# “Node” facilitated thermostable mechanophores for rapid self-strengthening in double network materials†

**DOI:** 10.1039/d5sc00151j

**Published:** 2025-07-10

**Authors:** Julong Jiang, Zhi Jian Wang, Ruben Staub, Yu Harabuchi, Alexandre Varnek, Jian Ping Gong, Satoshi Maeda

**Affiliations:** a Institute for Chemical Reaction Design and Discovery (WPI-ICReDD), Hokkaido University Kita 21, Nishi 10, Kita-ku Sapporo Hokkaido 001-0021 Japan gong@sci.hokudai.ac.jp smaeda@eis.hokudai.ac.jp; b Department of Chemistry, Faculty of Science, Hokkaido University Sapporo 060-8628 Japan; c Faculty of Advanced Life Science, Hokkaido University Sapporo 001-0021 Japan; d Laboratory of Chemoinformatics, UMR 7140, CNRS, University of Strasbourg 67081 Strasbourg France

## Abstract

Mechanophores are force-sensitive compounds that undergo chemical reactions under force stimuli. The design and discovery of efficient yet thermally stable mechanophores are crucial for developing self-strengthening materials. However, conventional mechanophores are often chemically unstable due to the presence of highly strained rings or weak covalent bonds, making the material sensitive to the change of temperature or UV irradiation. In this study, a comprehensive computational exploration was conducted to discover thermally stable, unconventional mechanophores for self-strengthening materials based on mechanoradical polymerisation. Notably, the computational procedure presented here serves as a general strategy for designing mechanophores intended for various applications. First, a conformational motif called a “node” along the force transduction direction was identified, significantly enhancing the force effect. Molecules with bridged rings emerged as ideal candidates for possessing a “node,” as the bridged structure helps to fix the key dihedral angle. Simulations predicted that polymers containing camphanediol and pinanediol could readily undergo C–C bond homolysis under force. Subsequently, automated reaction path exploration revealed the fate of the mechanoradicals and suggested that camphanediol could generate long-lived radicals, a crucial feature for self-strengthening materials. Following these computational predictions, we successfully prepared double-network hydrogels containing the camphanediol moiety. Careful experiments were then performed to quantify the concentration of mechanoradicals, and enhanced self-strengthening performance was demonstrated through loading–unloading tests.

## Abstract 2

A conformational motif called “node” was identified, and molecules with it were studied using EX-AFIR and NNP methods. The results guided the experimental design of thermostable, self-strengthening materials.

## Graphical abstract

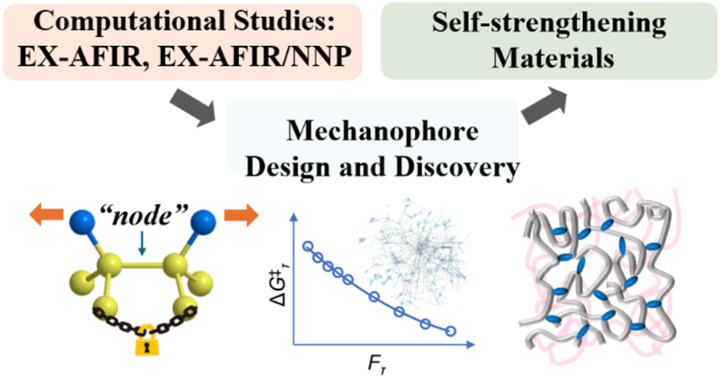

## Introduction

**Fig. 1:**
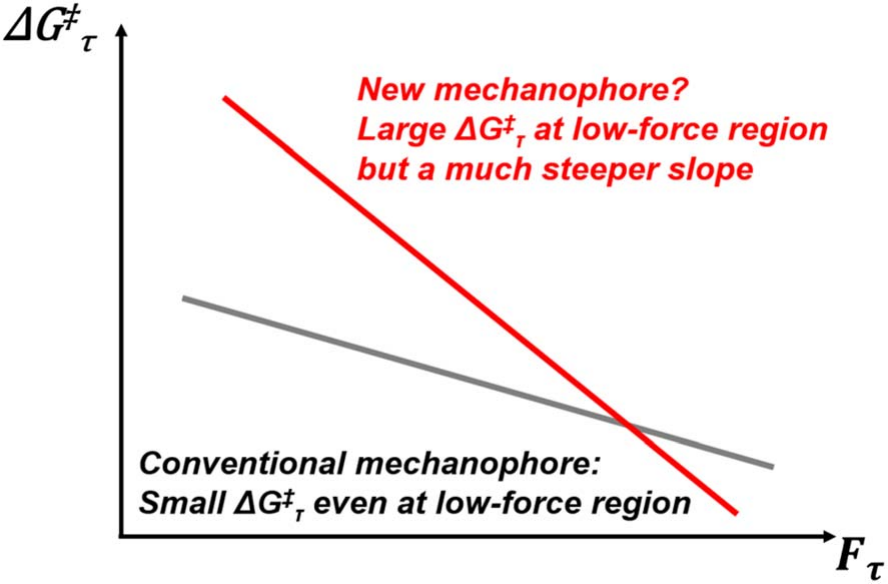
Illustration showing the free energy barrier as a function of external force (*i.e.*, Δ*G*^‡^_τ_–*F*_τ_ graph) for both a conventional mechanophore and a new mechanophore. Typically, the conventional mechanophore exhibits a small Δ*G*^‡^_τ_ even in the low-force region, indicating its high sensitivity to the change of temperature or UV irradiation. An ideal thermostable mechanophore should exhibit a large Δ*G*^‡^_τ_ in the low-force region with a much steeper slope, ensuring that it only evolves rapidly with an increasing external force.

Mechanophores are force-sensitive compounds that undergo chemical reactions when subjected to external force, making them highly valuable for enabling mechanoresponsive functions in advanced functional materials.^1–8^ The design of diverse mechanophores is pivotal for addressing specific needs in various applications. For instance, mechanophores can be incorporated into polymer chains, enabling smooth mechanodegradation.^9^ Recently, however, rather than focusing on polymer degradation, mechanophores have been employed to prepare self-strengthening materials based on mechanoradical polymerisation.^10–12^ Most conventional mechanophores rely on highly strained rings or weak covalent bonds (including those connecting two conjugated systems) which are typically chemically unstable.^13–19^ This means that the materials containing these compounds are sensitive to temperature, or UV irradiation. As illustrated in Fig. 1, conventional mechanophores exhibit a low activation barrier Δ*G*^‡^_τ_ even at small force *F*_τ_, making them prone to thermal activation without force stimuli. To achieve thermostable mechanophores, research on finding chemical motifs with a high Δ*G*^‡^_τ_ in the low-force region and a strong force sensitivity is required. Nevertheless, the discovery of new thermostable mechanophores is highly desirable but often considered challenging and time-intensive. While experimental advancements have been significant, there remains a high demand for computational tools to study mechanochemical reactions. From early methods like constrained geometry to simulate forces (CoGEF)^20,21^ to later approaches such as external force explicitly included (EFEI),^22–24^*ab initio* steered molecular dynamics (AISMD),^25–30^ single-ended growing string method (SE-GSM),^31,32^ and OpenMechanochem,^33,34^ efforts to develop new computational techniques continue to evolve.^35–38^ Recently, our group introduced the extended artificial force-induced reaction (EX-AFIR) method for computational exploration in mechanochemistry, providing a robust tool for this field.^39–45^

To design a new mechanophore, it is essential to understand the factors that promote mechanoreactivity. Craig *et al.* reported that stereochemistry (*e.g.*, *cis* and *trans*) plays a critical role in determining the activation force level (*F*_act_).^46^ However, the presence of a highly strained cyclopropane ring was initially believed to contribute most significantly to mechanoreactivity. Recently, the Boydston group confirmed the mechanochemical reactivity of VA-PNB (vinyl-addition polynorbornene) and attributed its reactivity to the strained rings.^47^ Nevertheless, the mechanoreactivity of VA-PNB is actually driven by a specific conformation called a “node,” rather than ring strain. We first proposed this concept in 2022 during a study of the retro-Diels–Alder reaction using the EX-AFIR method.^39^ Notably, Moore and co-workers have recently independently proposed a similar concept using their own theory called the restoring force triangle (RFT),^48,49^ which, in contrast to our fully *ab initio* EX-AFIR, identifies ideal mechanophores that have a strong covalent bond (Δ*E*) and a good mechanochemical coupling (*k*_eff_) in a highly efficient and accurate way using experimental data. The multivariable regression was performed by correlating *F*_act_, Δ*E* and *k*_eff_. It is also a milestone in computational polymer mechanochemistry, given that the RFT has a simple form and can be further improved in the future (*e.g.*, increasing the samples of the experimental data and introducing a machine-learning algorithm for a better correlation between *F*_act_, Δ*E* and *k*_eff_). Given this perspective of a “node”, we investigated the reactivity of both *cis*-VA-PNB and *trans*-VA-PNB under various force levels, as shown in Fig. S1 of the ESI.† The results demonstrate that *cis*-VA-PNB is significantly more reactive than *trans*-VA-PNB, despite both having the same level of ring strain. For a timescale of 0.1 s, the computed *F*_act_ values for *cis*-VA-PNB and *trans*-VA-PNB were 2160 and 2740 pN, respectively.

**Fig. 2:**
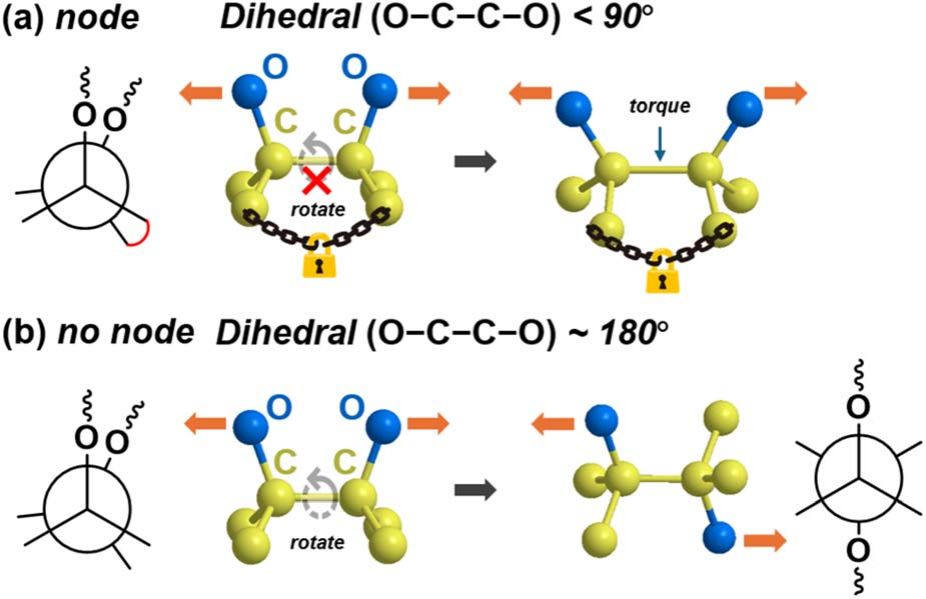
Conformation of a “node” on the breaking C–C bond: (a) the dihedral angle of the designated C–C bond is less than 90° (*i.e.*, with a node), inhibiting force transduction and generating torque on the bond. This results in severe distortion of bond angles and elongation of the bond length. (b) The dihedral angle is 180° (*i.e.*, without a node), allowing force to pass freely. No significant distortion of bond angles or bond length is observed in this case.

Following our previous work,^39^ we found that molecules with low *F*_act_ tend to possess a “node” structure, as illustrated in Fig. 2. While the presence of a highly strained ring is often emphasised, the dihedral angle conformation plays a crucial role in determining mechanoreactivity. A “node” refers to a dihedral angle smaller than 90°, with an ideal “node” having an angle close to 0°. At the node, force transduction is inhibited, encountering resistance that causes severe structural distortion and energy buildup. This generates significant stress at the node, ultimately leading to bond cleavage (see Fig. S2 in the ESI†).

Conventional mechanophores typically feature either a highly strained ring (*e.g.*, cyclopropane and cyclobutane) or an intrinsically weak covalent bond (*e.g.*, O–O bond, S–S bond, or bonds connecting conjugated systems). However, our findings suggest that these features are sufficient but not necessary. Molecules with a “node” conformation can also exhibit reactivity under tensile force, leading to the formation of mechanoradicals. To create such a node, the presence of a bond that cannot rotate freely is required.

## Results and discussion

Following this, our focus shifted to natural products, as many possess cyclic structures and are commercially available. Camphanediol and pinanediol, natural products used either in the cosmetics industry^50,51^ or as auxiliary ligands for synthesising chiral boron compounds,^52–54^ were selected as candidate mechanophores. Both compounds feature a “node,” with their bridged rings securing the crucial dihedral angle.

**Fig. 3:**
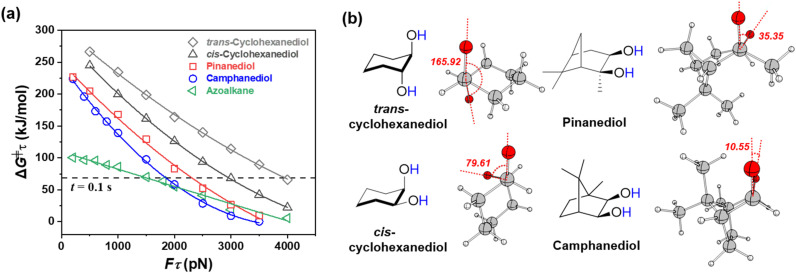
(a) Computed Δ*G*^‡^_τ_–*F*_τ_ graph at the UB3LYP-D3/6-311G(d,p)//UB3LYP-D3/6-311G(d,p) level of theory for the homolytic cleavage of the C–C bond in a series of vicinal diols. The *x*-axis represents the level of simulated tensile force, while the *y*-axis indicates the force-coupled free energy barrier for C–C bond cleavage. The Δ*G*^‡^_τ_–*F*_τ_ curve of a conventional mechanophore, azoalkane, is also included as a reference. (b) Optimised structures of the force-coupled transition states under *F*_τ_ = 2000 pN, highlighting the presence of a “node” in camphanediol, characterised by an O–C–C–O dihedral angle of 10.55°.

A series of EX-AFIR calculations were conducted to provide reliable *F*_act_ values for camphanediol, pinanediol, and their analogues, *trans*- and *cis*-cyclohexanediol. Fig. 3a shows the computed Δ*G*^‡^_τ_–*F*_τ_ curves for all these compounds, where Δ*G*^‡^_τ_ represents the force-coupled free energy barrier for C–C bond homolysis and *F*_τ_ is the applied force level. Camphanediol was identified as the most reactive species under external forces. For a timescale of 0.1 s, the computed *F*_act_ values for camphanediol, pinanediol, *cis*-cyclohexanediol, and *trans*-cyclohexanediol are 1850, 2320, 2970, and 3970 pN, respectively, confirming camphanediol as a reactive mechanophore. In addition, a comparison with azoalkane highlighted the distinctive feature of camphanediol described in Fig. 1.

Further analysis of the force-coupled transition state (at *F*_τ_ = 2000 pN) revealed O–C–C–O dihedral angles of 10.55° for camphanediol, 35.35° for pinanediol, 79.61° for *cis*-cyclohexanediol, and 165.92° for *trans*-cyclohexanediol (see Fig. 3b). Unlike conventional mechanophores, camphanediol neither exhibits substantial ring strain compared to cyclopropane or cyclobutane^55^ nor contains weak covalent bond (also see Fig. S3 in the ESI†). Instead, its bridged rings secure a preferred conformation, ultimately resulting in high mechanoreactivity. Despite the ability to generate radicals under tensile force, the further utilisation of these mechanoradicals requires them to exhibit relative longevity. To investigate the evolving processes within the reaction system, an automated reaction path exploration was conducted using the single-component EX-AFIR (SC/EX-AFIR) method on the potential energy surface calculated at the UB3LYP-D3/Def2-SV(P) level. The exploration was significantly accelerated by an iterative framework employing a DFT-xTB Δ-learning scheme (see the Computational methods section in the ESI†).^56^ During the reaction network exploration, a tensile force was consistently applied between the terminal –Me groups.

**Scheme 1:**
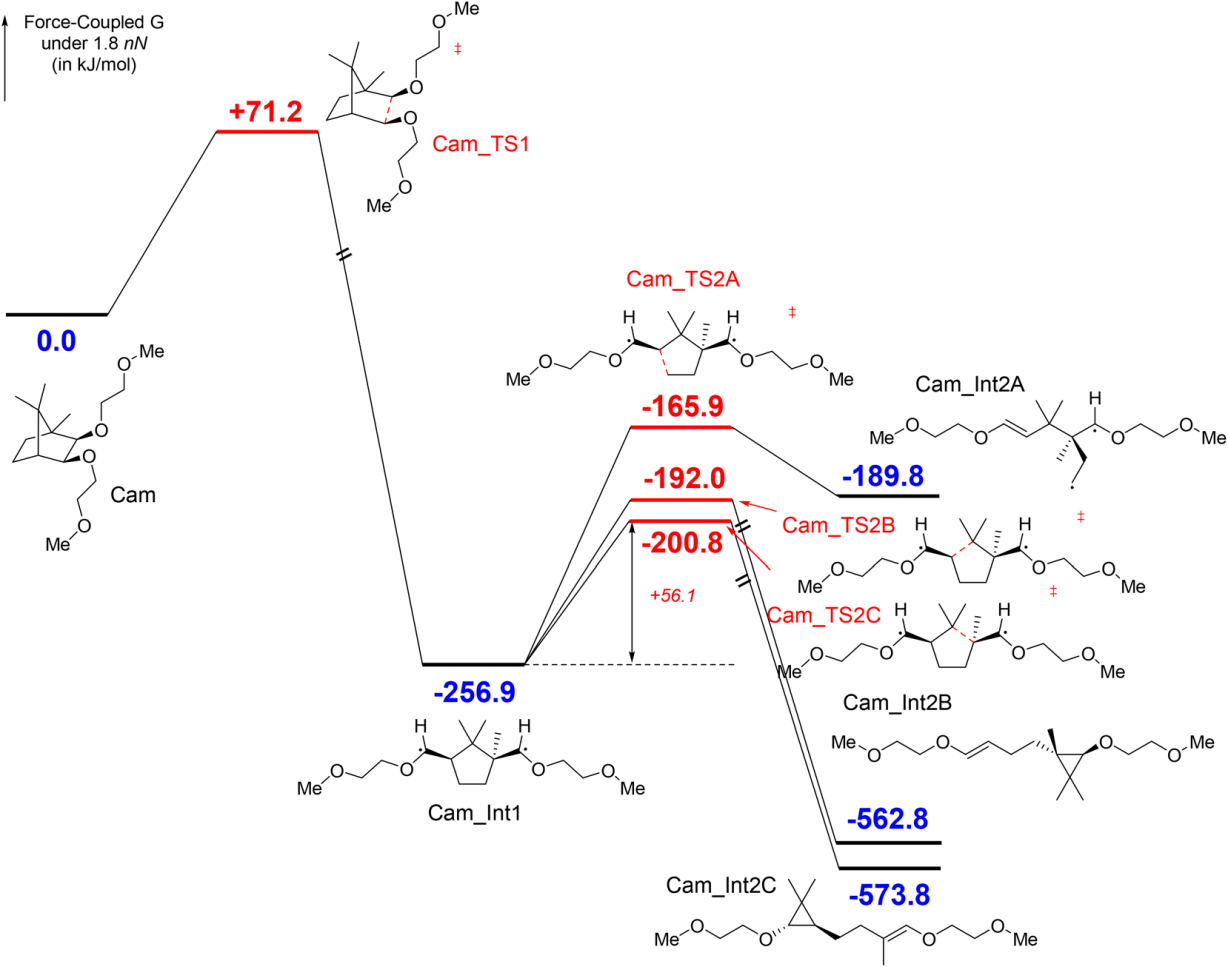
Mechanodegradation of camphanediol under *F*_τ_ = 1800 pN, showing the formation of the diradical Cam_Int1 with a relatively long half-life. The subsequent ring-opening reaction proceeds *via*Cam_TS2C with a force-coupled free energy barrier of +56.1 kJ mol^−1^. Calculations were performed at the UB3LYP-D3/6-311G(d,p)//UB3LYP-D3/6-311G(d,p) level of theory, including solvation effects.

Scheme 1 illustrates the computed reaction pathways of camphanediol under a tensile force of 1800 pN. The energetically accessible pathways identified by the automated search were further optimised at the UB3LYP-D3/6-311G(d,p)//UB3LYP-D3/6-311G(d,p) level under *F*_τ_ = 1800 pN. The initial step involves the force-promoted C–C bond homolysis, leading to the formation of Cam_Int1, a 1,5-diradical species with a five-membered ring separating the two radicals. Although a five-membered ring is generally stable, its proximity to a carbon radical in this case results in a radical ring-opening reaction through three possible pathways. Cam_TS2A exhibited the highest energy, with a barrier of +91.0 kJ mol^−1^, as the breaking C–C bond in Cam_TS2A does not directly align with the applied force. The force-coupled transition state Cam_TS2B had a slightly higher energy than Cam_TS2C, as the latter generates a tertiary carbon radical. Consequently, the effective barrier for the ring-opening of Cam_Int1 is calculated as +56.1 kJ mol^−1^ (Cam_Int1 → Cam_TS2C) under 1800 pN, indicating a relatively long half-life. The ring-opening produces a 1,3-diradical species, which cannot further break down into alkene molecules. Instead, it forms a cyclopropane derivative, labelled as Cam_Int2C. Owing to the force effects, the resulting three-membered ring lacks a “node”. EX-AFIR calculations suggest that the subsequent ring-opening of this three-membered structure requires a significantly higher activation force of 2700 pN (see Fig. S4 in the ESI†).

Reaction path explorations for the degradation of pinanediol under force were also conducted (see Scheme S1 in the ESI†). Although pinanediol exhibits a relatively low *F*_act_, the mechanoradicals generated from it are notably short-lived. The force-induced ring-opening reaction of Pin_Int1 has a barrier of only 31.1 kJ mol^−1^ under *F*_τ_ = 2300 pN, resulting in the formation of a 1,4-diradical species. This 1,4-diradical species, Pin_Int2C, readily undergoes scissile decomposition to produce two alkene molecules (see Fig. S5† for a stability comparison with Cam_Int1). Consequently, the mechanoradicals derived from pinanediol are unlikely to be experimentally observed or practically utilised.

**Fig. 4:**
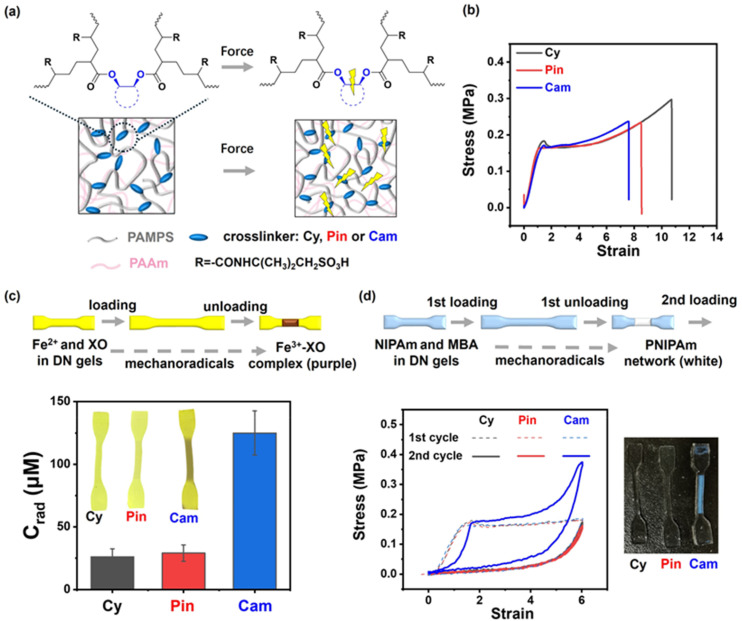
Applications of *cis*-cyclohexanediol (Cy), pinanediol (Pin), and camphanediol (Cam) mechanophores as diacrylate crosslinkers in the first network of double-network hydrogels. (a) Schematic illustration of the double-network hydrogel structure and force-induced bond cleavage in the first network. (b) Stress–strain curves of DN gels with varying crosslinkers under uniaxial tensile testing. (c) Mechanoradical concentration (*C*_rad_) in different DN gels after stretching, evaluated by the Fenton-related colour reaction. DN gels containing ferrous ions (Fe^2+^) and xylenol orange (XO) were stretched to a preset strain of 6 and then unloaded for UV measurement. The images show the stretched DN gels with Fe^2+^ and XO. (d) Stress–strain curves illustrating the cyclic loading and unloading behaviour of different DN gels containing the monomer NIPAm and crosslinker MBA. DN gels were first stretched to a strain of 6 and then unloaded (dashed line). After 3 min, the stretched gels were re-stretched and unloaded (solid line). The images show the stretched DN gels with NIPAm and MBA.

To confirm the mechanoreactivity of camphanediol and to demonstrate its application in self-strengthening materials, we covalently incorporated these molecules into promising polymer-double network (DN) hydrogels and investigated their capacity to generate more mechanoradicals and strengthen the materials. We modified *cis*-cyclohexanediol, pinanediol, and camphanediol into diacrylate crosslinkers and used them to synthesise poly(2-acrylamido-2-methyl-1-propanesulfonic acid) (PAMPS) single network (SN) hydrogels. These PAMPS networks, incorporating different crosslinkers, serve as the first network in the DN hydrogel, with the second network identically consisting of polyacrylamide (PAAm) crosslinked with *N*,*N*′-methylenebis(acrylamide) (MBA) (Fig. 4a).^57^ The resultant DN gels are denoted as DN-Cy, DN-Pin, and DN-Cam, respectively. Previous research has shown that DN systems are well-suited for studying mechanophore activation, where mechanophore crosslinkers with lower activation forces in the first network can be more efficiently and selectively activated.^41,45^Fig. 4b and S6† show that the three SN gels and DN hydrogels exhibited similar swelling and mechanical properties, indicating comparable network structures. However, they exhibited significant differences in generating reactive mechanoradicals. As shown in Fig. 4c and S7,† when the gels, loaded with ferrous ions and xylenol orange (XO), were stretched to a strain of 6 and then unloaded in air, DN-Cam exhibited a noticeable colour change in the necked region, while DN-Cy and DN-Pin showed much weaker changes (see the ESI† for a video clip). This colour change can be attributed to the fact that the generated mechanoradicals oxidise ferrous ions to ferric ions in the presence of oxygen and water, and the ferric ions subsequently react with XO to form a purple complex. Quantitative UV spectroscopic analysis revealed that DN-Cam generated approximately 130 μM mechanoradicals, over four times the amount produced by DN gels crosslinked with pinanediol and *cis*-cyclohexanediol, each of which generated only approximately 30 μM. Moreover, we investigated whether these mechanoradicals could trigger the polymerisation of monomers and crosslinkers, thereby endowing the gel with mechanoresponsive self-strengthening properties (Fig. 4d). After oxygen extraction in a glove box for 2 h, DN gels containing 2.0 M monomer *N*-isopropylacrylamide (NIPAm) and 0.15 M crosslinker MBA were stretched to a strain of 6 and unloaded. After 3 min, DN-Cam turned white in the necked region and became stronger during a second cycle of loading–unloading tests, while DN-Cy and DN-Pin did not. This strongly suggests that a high concentration of long-lived mechanoradicals was generated in DN-Cam, facilitating the formation of a new PNIPAm network that strengthened the material.

These results strongly indicate that camphanediol is a mechanophore, capable of generating reactive radicals upon activation. These radicals likely originate from the first-step C–C bond cleavage of camphanediol (*i.e.*, Cam_Int1) and the subsequent ring opening of Cam_Int2C, leading to the formation of long-lived radicals (although the latter requires a high activation force, *F*_act_). By contrast, despite having a similarly low *F*_act_, pinanediol cannot generate long-lived radicals suitable for application, as its intermediate (Pin_Int1) is a short-lived diradical that undergoes rapid scissile cleavage into two alkenes (*via*Pin_TS3CC, see Scheme S1 in the ESI†). Additionally, compared to the conventional azoalkane mechanophore, which is inherently reactive owing to weak C–N bonds, camphanediol exhibits excellent thermal and UV stability. After heating at 80 °C or exposing it to UV light for 10 h, the NMR spectra of all three diols remained unchanged (see Fig. S8 in the ESI†). This stability allows the camphanediol mechanophore to be incorporated into polymer materials *via* high-temperature or UV polymerisation, imparting superior stability to the resultant material and broadening its potential applications.

## Conclusion

**Fig. 5:**
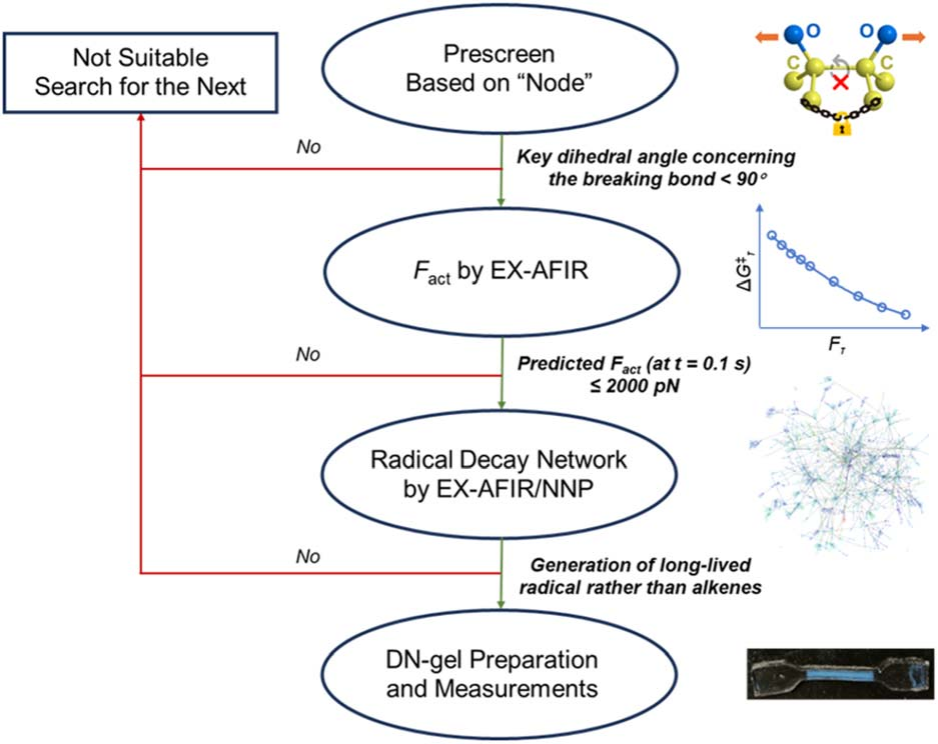
A general, theory-driven procedure to identify radical-type mechanophores suitable for thermally stable self-strengthening materials.

In conclusion, we have presented a computational design for radical-type mechanophores to be used in self-strengthening materials. This computational approach is both general and time-efficient, facilitating the discovery of new mechanoreactive compounds tailored for specific applications (as shown in Fig. 5). The procedure itself incorporates the concept of a “node,” which refers to a bond that cannot rotate freely, used during the pre-screening stage. Subsequently, those molecules with a “node” were subjected to EX-AFIR calculations to derive their *F*_act_ values over a given timescale. In our study, camphanediol and pinanediol were both identified as qualified mechanophores with relatively low *F*_act_. As economical natural products, these two compounds are both affordable and readily accessible. However, for self-strengthening materials, mechanoradicals with a relatively long half-life are essential. The EX-AFIR/NNP calculations revealed the plausible decay pathways of the mechanoradicals generated from camphanediol and pinanediol. These calculations revealed that a long-lived 1,5-diradical intermediate is formed upon the mechanoactivation of camphanediol. This relatively long-lived diradical species allows it to react with the monomers present in the system, ensuring excellent self-strengthening performance. By contrast, for pinanediol, two alkene molecules are formed rapidly from the mechanoradical. Based on these insights, DN hydrogels containing the aforementioned molecules were successfully prepared, and subsequent tests were performed. Quantitative experimental investigations showed that, compared to DN-Cy and DN-Pin, DN-Cam generates more than four times the number of mechanoradicals. Additionally, according to the loading–unloading tests, DN-Cam exhibited a satisfactory self-strengthening behaviour. Importantly, our selection guidelines do not require strained rings or weak covalent bonds. Therefore, camphanediol shows satisfactory thermal and UV stability, evolving rapidly only under mechanical force. Given the easy accessibility of camphanediol, we believe this type of mechanophore can be further utilised in materials science. So far, we focused on the formation of mechanoradicals. Moreover, we are expecting to pay more attention to the polymerisation steps, such as the radical migration and diffusion, as well as the distribution of forces in the hydrogels. In addition, the combination of EX-AFIR and NNP for further development of mechanoreactive species with specific purposes (other than the generation of radicals for polymerisation) is currently ongoing in our groups.

## Author contributions

J. J. and S. M. performed all the calculations and analysed the results involved in this study. Z. W. prepared the DN hydrogels containing the predicted mechanophores and subsequently tested the self-strengthening properties of the material. Y. H., R. S. and A. V. developed the NNP method used in this study. J. P. G. and S. M. directed the project. All authors contributed to the discussion and preparation of the manuscript.

## Conflicts of interest

There are no conflicts to declare.

## Supplementary Material

SC-OLF-D5SC00151J-s001

SC-OLF-D5SC00151J-s002

## Data Availability

The data supporting this article have been included as part of the ESI.†
