# Supplementary material for: “Node” facilitated thermostable mechanophores for rapid self-strengthening in double network materials
Source: Chem Sci. 2025 Jul 10;16(31):14278–85. doi: 10.1039/d5sc00151j (PMC12242834; doi:10.1039/d5sc00151j)
Supplement: SC-016-D5SC00151J-s002 [file SC-016-D5SC00151J-s002.pdf]

Supplementary Information (SI) for Chemical Science.
This journal is © The Royal Society of Chemistry 2025

*Electronic Supplementary Information*

*for*

# "Node" Facilitated Thermostable Mechanophores for Rapid Self-strengthening in Double Network Materials

Julong Jiang,‡[a] Zhi Jian Wang,‡[a] Ruben Staub,[a] Yu Harabuchi,[a] Alexandre Varnek,[a,d]

Jian Ping Gong,*[a,c] Satoshi Maeda*[a,b]

[a]*Institute for Chemical Reaction Design and Discovery (WPI-ICReDD), Hokkaido University, Kita 21, Nishi 10, Kita-ku, Sapporo, Hokkaido 001-0021, Japan*
[b]*Department of Chemistry, Faculty of Science, Hokkaido University, Sapporo 060-8628, Japan*
[c]*Faculty of Advanced Life Science, Hokkaido University, Sapporo 001-0021, Japan*
[d]*Laboratory of Chemoinformatics, UMR 7140, CNRS, University of Strasbourg, 67081 Strasbourg, France*

‡: contributed equally
*Email: gong@sci.hokudai.ac.jp; smaeda@eis.hokudai.ac.jp

**This file includes：**

1. Computational Section
    1.1. Computational Methods
    1.2. Supporting Computational Results (Figs. S1-S5, Scheme S1, Tables S1-S5)
2. Experiment Section
    2.1. Experiment Materials and Methods
    2.2. Supporting Experiment Results (Figs. S6-S8)
    2.3. NMR Spectra
3. Cartesian Coordinates of Optimized Structures

## 1. Computational Section

### 1.1. Computational Methods

All the calculations were performed at the DFT level of theory with the B3LYP hybrid functional[1] as implemented in Gaussian 16.[2] To describe the dispersion properly, an explicit dispersion correction term called GD3,[3] was also employed in the DFT calculations. The 6-311G(d,p) basis set[4] was used for all the atoms involved in this study during both the geometry optimization and the single-point calculation processes. To describe the open-shell singlet species involved in the homolysis process, the unrestricted DFT, as well as the procedure to test and optimize the wavefunction, was used in this work.[5] To describe the solvation environment, the implicit solvation model, IEF-PCM,[6] is applied to all the calculations involved in this study and toluene ($\varepsilon$ = 2.3741) was used as the solvent. All the structures were fully optimized with the consideration of external tensile force when necessary. The external tensile force $F_\tau$ is properly simulated through our extended artificial force induced reaction method (i.e., EX-AFIR).[7] The EX-AFIR method can provide a series of TSs under different $F_\tau$ efficiently. In this study, $F_\tau$ was applied to the terminal methyl groups in all cases. For the computation of hydrogenation enthalpies, it was conducted at the level of B3LYP-D3/6-311G(d,p)//B3LYP-D3/6-311G(d,p) in gas phase without any constraints.

The automated explorations of degradation pathways of mechanoradicals generated from camphanediol and pinanediol were done using the single component (SC) algorithm of the EX-AFIR method. During the explorations, the constant tensile force of 3000 pN and 4000 pN was applied between the two terminal methyl groups for camphanediol and pinanediol, respectively, in order to trigger all possible reactions. Such an exploration is denoted as SC/EX-AFIR. The collision energy parameter $\gamma$ of the AFIR calculations was set as 400 kJ/mol throughout the search. In order to efficiently obtain energy and force of the quality at the B3LYP-D3/Def2-SV(P) level, we adopted a machine learning framework with a Neural Network Potential (NNP), using an iterative scheme based on continual learning and Δ-learning. In this scheme, the SC/EX-AFIR exploration and machine learning of the energy difference $\Delta E$ between the GFN0-xTB[8] and B3LYP-D3/Def2-SV(P) levels are done alternately. Each iteration of the incremental scheme consists of the following three steps. First, $n$ paths are explored by the SC/EX-AFIR method on the PES of GFN0-xTB ($E^{\mathrm{xTB}}$) augmented by the neural network representation of $\Delta E$ ($\Delta E^{\mathrm{NN}}$), where $\Delta E^{\mathrm{NN}} = 0$ at any configuration in the first iteration. Second, B3LYP-D3/Def2-SV(P) energy and force calculations are done at all local minima and approximate transition states obtained in the first step. Third, $\Delta E^{\mathrm{NN}}$ is trained using SpookyNet[9] for all the data (xTB and DFT energy and force) acquired in the second step of the present and previous iterations. In the present applications, for camphanediol, 5 iterations were performed with $n$ = 1000, 1000, 1000, 1000 and 1000, while 3 iterations with $n$ = 300, 1000, 1000 were performed for pinanediol. Finally, the final exploration was performed by SC/EX-AFIR for 1500 paths on $E^{\mathrm{xTB}} + \Delta E^{\mathrm{NN}}$ using the final $\Delta E^{\mathrm{NN}}$. Important paths were extracted from the reaction pathway network obtained by the final SC/EX-AFIR exploration and

recalculated at the UB3LYP-D3/6-311G(d,p)//UB3LYP-D3/6-311G(d,p) level of theory under the stretching force of 1800 pN for camphanediol and 2300 pN for pinanediol. Therefore, the energetics shown in **Scheme 1** are not affected at all by the accuracy of $\Delta E^{\mathrm{NN}}$. See ref. 10 for details of how we trained $\Delta E^{\mathrm{NN}}$.

## References:


1. (a) A. D. Becke, *J. Chem. Phys.* **1993**, *98*, 1372–1377. (b) C. Lee, W. Yang, R. G. Parr, *Phys. Rev. B* **1988**, *37*, 785-789.
2. Gaussian 16, Revision C.01, M. J. Frisch, G. W. Trucks, H. B. Schlegel, G. E. Scuseria, M. A. Robb, J. R. Cheeseman, G. Scalmani, V. Barone, G. A. Petersson, H. Nakatsuji, X. Li, M. Caricato, A. V. Marenich, J. Bloino, B. G. Janesko, R. Gomperts, B. Mennucci, H. P. Hratchian, J. V. Ortiz, A. F. Izmaylov, J. L. Sonnenberg, D. Williams-Young, F. Ding, F. Lipparini, F. Egidi, J. Goings, B. Peng, A. Petrone, T. Henderson, D. Ranasinghe, V. G. Zakrzewski, J. Gao, N. Rega, G. Zheng, W. Liang, M. Hada, M. Ehara, K. Toyota, R. Fukuda, J. Hasegawa, M. Ishida, T. Nakajima, Y. Honda, O. Kitao, H. Nakai, T. Vreven, K. Throssell, J. A. Montgomery, Jr., J. E. Peralta, F. Ogliaro, M. J. Bearpark, J. J. Heyd, E. N. Brothers, K. N. Kudin, V. N. Staroverov, T. A. Keith, R. Kobayashi, J. Normand, K. Raghavachari, A. P. Rendell, J. C. Burant, S. S. Iyengar, J. Tomasi, M. Cossi, J. M. Millam, M. Klene, C. Adamo, R. Cammi, J. W. Ochterski, R. L. Martin, K. Morokuma, O. Farkas, J. B. Foresman, and D. J. Fox, Gaussian, Inc., Wallingford CT, 2016.
3. (a) S. Grimme, J. Antony, S. Ehrlich, H. Krieg, *J. Chem. Phys.* **2010**, *132*, 154014. (b) S. Grimme, S. Ehrlich, L. Goerigk, *J. Comput. Chem.* **2011**, *32*, 1456–1465.
4. (a) A. D. McLean, *J. Chem. Phys.* **1980**, *72*, 5639–5648. (b) K. Raghavachari, J. S. Binkley, R. Seeger, J. A. Pople, *J. Chem. Phys.* **1980**, *72*, 650–654.
5. R. Bauernschmitt, R. Ahlrichs, *J. Chem. Phys.* **1996**, *104*, 9047–9052.
6. G. Scalmani, M. J. Frisch, *J. Chem. Phys.* **2010**, *132*, 114110.
7. J. Jiang, K. Kubota, M. Jin, Z. Wang, T. Nakajima, H. Ito, J. P. Gong, S. Maeda, *ChemRxiv* **2022**, DOI: 10.26434/chemrxiv-2022-fr09l.
8. (a) P. Pracht, E. Caldeweyher, S. Ehlert, S. Grimme, *ChemRxiv* **2019**, DOI: 10.26434/chemrxiv.8326202.v1. (b) C. Bannwarth, E. Caldeweyher, S. Ehlert, A. Hansen, P. Pracht, J. Seibert, S. Spicher, S. Grimme, *WIREs Comput. Mol. Sci.* **2021**, *11*, e1493.
9. O. T. Unke, S. Chmiela, M. Gastegger, K. T. Schütt, H. E. Sauceda, K. -R. Müller, *Nat. Commun.* **2021**, *12*, 7273.
10. R. Staub, P. Gantzer, Y. Harabuchi, S. Maeda, A. Varnek, *Molecules* **2023**, *28*, 4477.

## 1.2. Supporting Computational Results

### Mechano-reactivities of *cis*- and *trans*- VA-PNB under forces

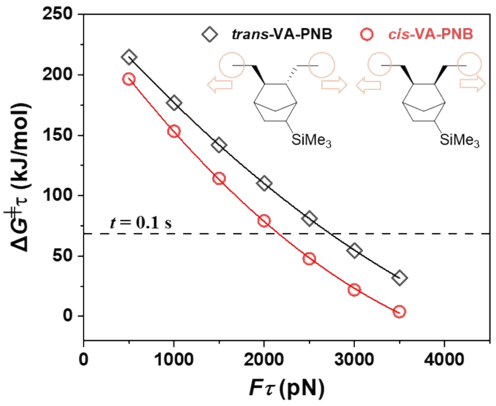


**Figure S1.** $\Delta G^{\ddagger}{}_{\tau} - F_{\tau}$ graph of *cis*-VA-PNB and *trans*-VA-PNB, suggesting ring strain is not the major reason for the mechanochemical reactivity observed in the sonication reaction.

### Distortion of cis-pulling and trans-pulling based on a tetramethyl norbornane model

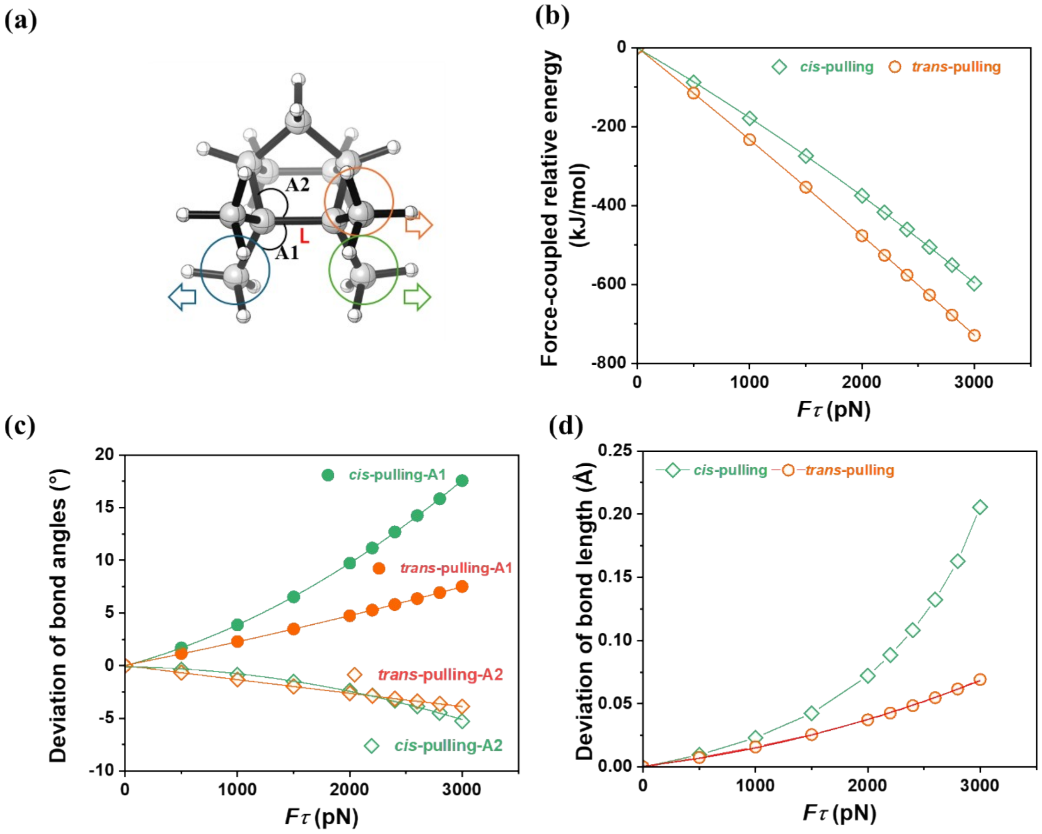

**Figure S2.** Here we used a model molecule (tetramethyl norbornene, on which repulsive force can be readily added to the specific Me groups to simulate the tensile force) for the illustration of how the pulling direction (a) affects: (b) Energy; (c) bond angles; (d) bond length. Note that the dihedral angle of *cis*-pulling is 0° (i.e., having an ideal node) and it is 120° for the *trans*-pulling.

## Computed Hydrogenation Enthalpies of Cyclic Diols

The hydrogenation enthalpies of a series of cyclic diols were computed, suggesting that the ring strain in camphanediol and pinanediol is much smaller than that in cyclobutane-1,2-diol and cyclopropane-1,2-diol. The calculation results therefore indicate that the ring strain does not contribute significantly to the mechanochemical reactivity observed for camphanediol and pinanediol.

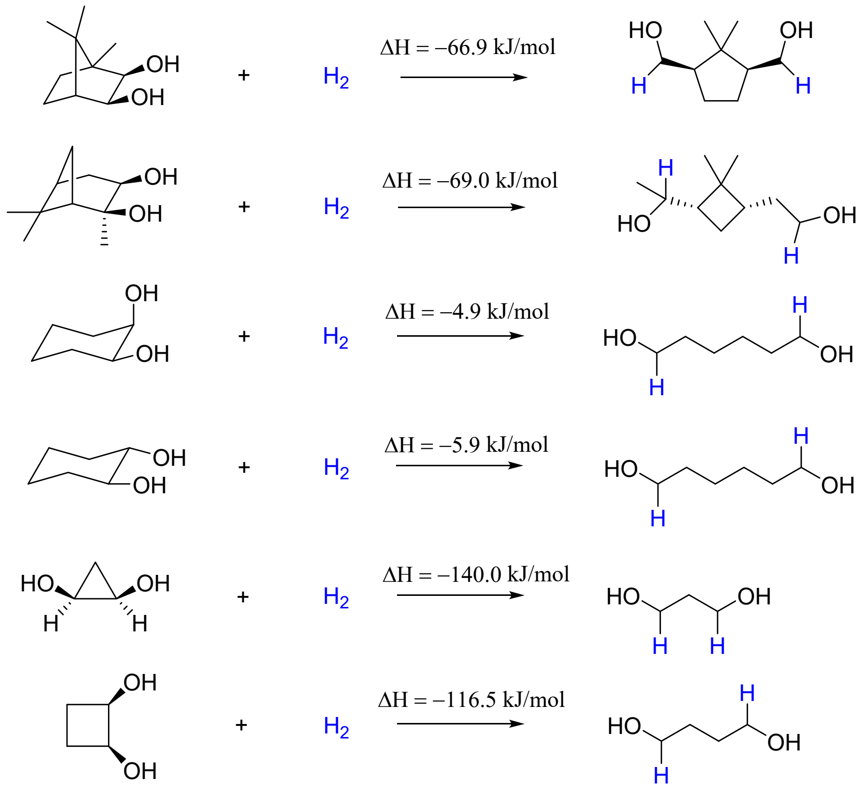


**Figure S3.** Computed hydrogenation enthalpies of a series of diols, from which we can find that hydrogenation enthalpies of camphanediol and pinanediol are much smaller than those of cyclopropane-1,2-diol and cyclobutane-1,2-diol.

## Degradation Pathways of Pinanediol under Tensile Force

Calculations regarding the degradation of pinanediol were also performed with the assistance of the neutral network potential (NNP) developed by Varnek and co-workers. Shown in **Scheme S1** is the SC-AFIR derived pathways under $F_{\tau}$ = 2300 pN which have accessible barriers. Pinanediol shows a slightly low reactivity towards the force-triggered homolytic cleavage. Once the six-membered ring is opened, the resultant intermediate **Pin_Int1** has three plausible pathways to follow, and each TS has been successfully located. **Pin_TS2C** was found to have the lowest barrier, which is only +31.4 kJ/mol, since an alkene and a tertiary carbon radical are generated upon the opening of the cyclobutane ring. **Pin_Int3C**, as the product connecting to **TS3C**, is actually a 1,4-diradical. Therefore, the following C-C cleavage through **TS3CC** is almost barrierless (i.e., 13.8 kJ/mol), given the fact that two stable alkenes can be generated. Based on the computational studies illustrated above, pinanediol, though has a relatively low $F_{\mathrm{act}}$, from which the diradical species generated are very short-lived. Therefore, it is very unlikely that these mechanoradicals can be experimentally observed or be further utilized.

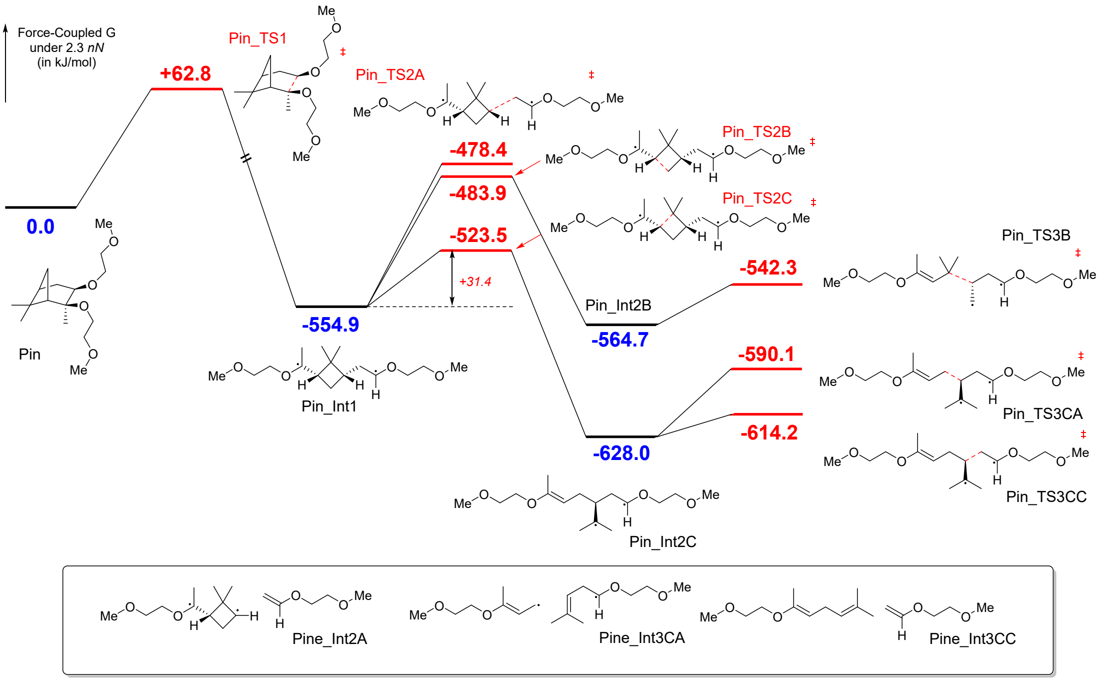


**Scheme S1.** Mechanodegradation of pinanediol under $F_{\tau}$ = 2300 pN, from which we can see the resultant intermediate **Pin_Int1** undergoes a rapid decomposition (via **Pin_TS2C** and **Pin_TS3CC**) leading to two alkene molecules.

**Further Stretching of Intermediate Cam_Int2C**

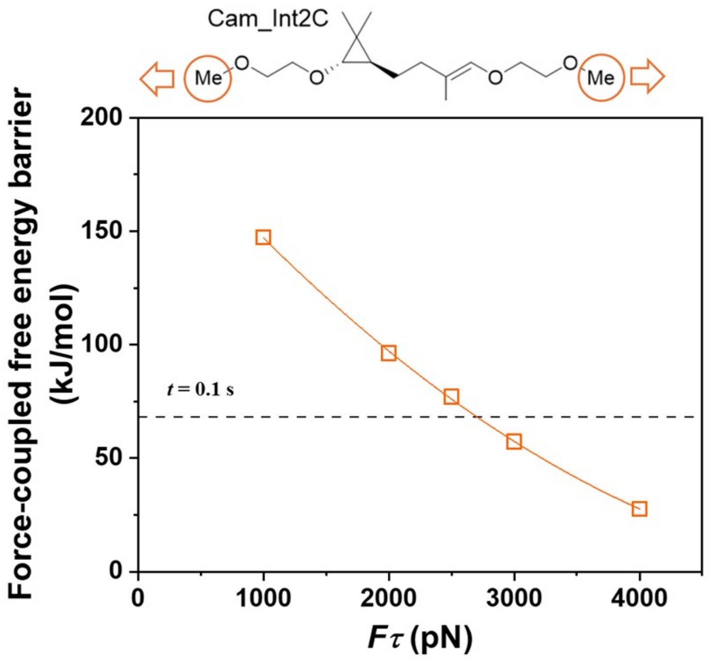


**Figure S4.** $\Delta G^{\ddagger}{}_{\tau} - F_{\tau}$ graph for the ring-opening reaction of **Cam_Int2C** via **TS3C**, which generates a 1,3-diradical that cannot further decompose at $F_{\text{act}}$ = 2700 pN.

**Stability of Two Different Cyclic Diradicals**

Two different cyclic diradical species were generated from the stretching experiments of camphanediol and pinanediol, respectively. Once generated, both cyclic diradical can undergo a ring-opening reaction via a transition state called **TS2C**. However, the reactivity of these two species is completely different, with **Pin_Int1** being much more reactive than **Cam_Int1**. Shown in **Figure S4** is how the barrier of ring-opening reaction evolves with an increasing force level. The high reactivity of Pin_Int1 can be owed to the ring-strain of the four-membered ring. In addition, **Pin_TS2C** will lead to a 1,4-diradical species, which will immediately turn into two alkene molecules via the C2−C3 bond cleavage process.

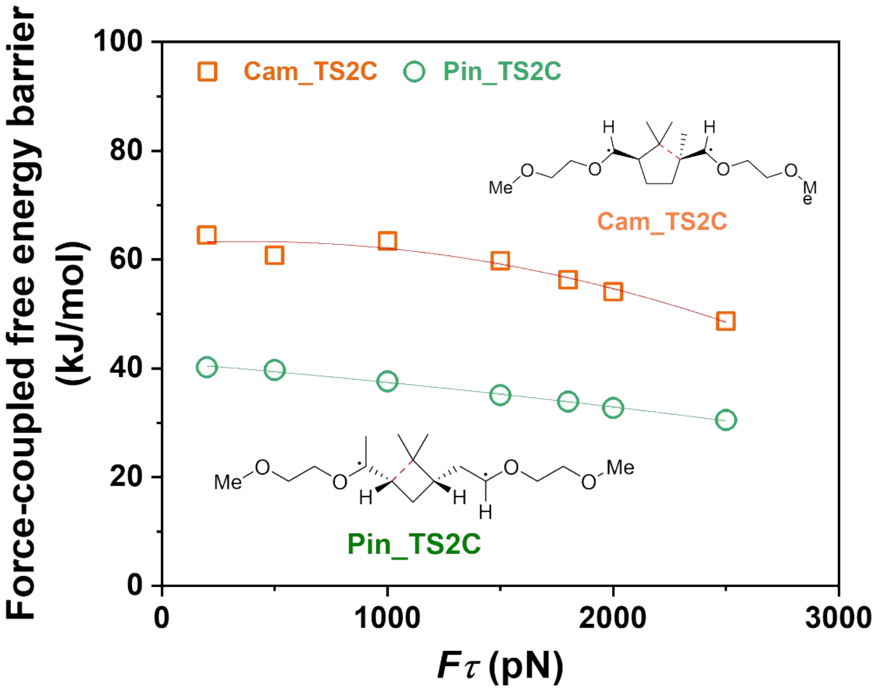


**Figure S5**. Stability of two cyclic mechanoradicals generated from the stretching experiments of camphanediol and pinanediol, respectively. From which we can figure out that the mechanoradical generated from camphanediol is relatively long-lived, and its stability is not significantly affected by the external tensile force.

### Tables of All $\Delta G^{\ddagger}_{\tau} - F_{\tau}$ Data

**Table S1.** $\Delta G^{\ddagger}_{\tau} - F_{\tau}$ Data of Figure S1

| $F_{\tau}$ (in pN) | $\Delta G^{\ddagger}_{\tau}$ (cis-VA-PNB) (in kJ/mol) | $\Delta G^{\ddagger}_{\tau}$ (trans-VA-PNB) (in kJ/mol) |
|---|---|---|
| 3500 | 3.8 | 31.9 |
| 3000 | 21.9 | 54.5 |
| 2500 | 47.7 | 81.0 |
| 2000 | 79.1 | 110.2 |
| 1500 | 114.2 | 141.9 |
| 1000 | 153.3 | 176.9 |
| 500 | 196.5 | 214.7 |

**Table S2.** $\Delta G^{\ddagger}_{\tau} - F_{\tau}$ Data of Figure 3a

| $F_{\tau}$ (in pN) | $\Delta G^{\ddagger}_{\tau}$ (Cam) (in kJ/mol) | $\Delta G^{\ddagger}_{\tau}$ (Pin) (in kJ/mol) | $\Delta G^{\ddagger}_{\tau}$ (cis-Cy) (in kJ/mol) | $\Delta G^{\ddagger}_{\tau}$ (trans-Cy) (in kJ/mol) |
|---|---|---|---|---|
| 4000 | – | – | 22.1 | 65.7 |
| 3500 | 0.2 | 9.8 | 42.1 | 89.7 |
| 3000 | 9.0 | 26.5 | 68.1 | 114.3 |
| 2500 | 28.5 | 52.3 | 93.7 | 140.0 |
| 2000 | 58.4 | 83.2 | 126.2 | 163.9 |
| 1500 | 97.6 | 129.4 | 161.6 | 198.6 |
| 1000 | 139.0 | 168.0 | 199.5 | 234.5 |
| 800 | 156.8 | – | – | – |
| 600 | 173.0 | – | – | – |
| 500 | – | 204.6 | 244.8 | 266.5 |
| 400 | 196.0 | – | – | – |
| 200 | 223.9 | 226.3 | – | – |

**Table S3.** $\Delta G^{\ddagger}_{\tau} - F_{\tau}$ Data of Figure S4

| $F_{\tau}$ (in pN) | $\Delta G^{\ddagger}_{\tau}$ (Cam_TS3C) (in kJ/mol) |
|---|---|
| 4000 | 27.6 |
| 3000 | 57.3 |
| 2500 | 77.0 |
| 2000 | 96.3 |
| 1000 | 147.3 |

**Table S4.** $\Delta G^{\ddagger}_{\tau} - F_{\tau}$ Data of Figure S5

| $F_{\tau}$ (in pN) | $\Delta G^{\ddagger}_{\tau}$ (Cam_TS2C) (in kJ/mol) | $\Delta G^{\ddagger}_{\tau}$ (Pin_TS2C) (in kJ/mol) |
|---|---|---|
| 2500 | 48.2 | 30.5 |
| 2000 | 53.7 | 32.7 |
| 1800 | 56.1 | 33.9 |
| 1500 | 59.7 | 35.1 |
| 1000 | 63.5 | 37.6 |
| 500 | 60.8 | 39.7 |
| 200 | 64.4 | 40.2 |

**Table S5.** $\Delta G^{\ddagger}_{\tau} - F_{\tau}$ Data of Azoalkane (shown in Figure 3a as reference)

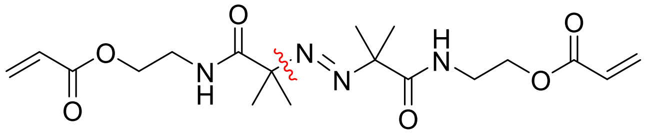

| $F_{\tau}$ (in pN) | $\Delta G^{\ddagger}_{\tau}$ (Azoalkane) (in kJ/mol) |
|---|---|
| 4000 | 5.7 |
| 3000 | 18.8 |
| 2500 | 41.2 |
| 2000 | 55.1 |
| 1800 | 63.7 |
| 1500 | 70.0 |
| 1000 | 85.9 |
| 800 | 88.9 |
| 600 | 95.9 |
| 400 | 96.5 |
| 200 | 100.4 |

## 2. Experimental Section

### 2.1. Experiment Materials and Methods

### Materials

Cis-1,2-cyclohexanediol, (1S,2S,3R,5S)-(+)-2,3-pinanediol, acryloyl chloride are purchased from Tokyo Chemical Industry. (±)-exo,exo-2,3-camphanediol is purchased from Sigma-Aldrich. Triethylamine is purchased from Wako Pure Chemical Corporation. 2-Acrylamido-2-methylpropanesulfonic acid (AMPS) was provided by Toagosei. Acrylamide (AAm) was purchased from Junsei Chemical. *N*, N'-methylenebis (acrylamide) (MBA), 2,2'-azobis(2,4-dimethyl) valeronitrile (ABVN), *N,N*-dimethylformamide (DMF), 2,2'-azobis[2-(2-imidazolin-2-yl)propane] dihydrochloride (VA-044) were purchased from Wako Pure Chemical Industry.

### General Process for the Synthesis of Diacrylate Linkers

Diacrylate linkers were synthesized by the treatment of diol with acryloyl chloride. To a 250 ml three-necked round-bottomed flask equipped with a magnetic stirrer and purged with Argon gas, diol (1 equiv.) and triethylamine (TEA, 8 equiv.) were added sequentially to $CH_2Cl_2$ (5 ml/mmol diol). The resulting solution was cooled in an ice

bath. After that, a solution of acryloyl chloride (6 equiv.) in $CH_2Cl_2$ (1 ml/mmol) was added dropwise into the solution. The reaction mixture was stirred overnight, and it was allowed to warm slowly to room temperature. After the reaction was completed, the solution was poured onto saturated $NaHCO_3$ to quench the reaction and $CH_2Cl_2$ to extract the organic compound. The layers were separated, and the aqueous layer was extracted with $CH_2Cl_2$ twice. The combined organics were washed with saturated $NaHCO_3$ and pure water, and then dried over $Na_2SO_4$. After removal of solvent by evaporation, the crude product was then purified by silica gel chromatography (10% ~ 20% EtOAc / Hexane gradient eluent) to give the product.

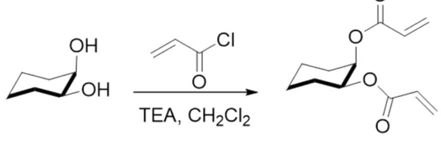


cis-cyclohexanediol 1

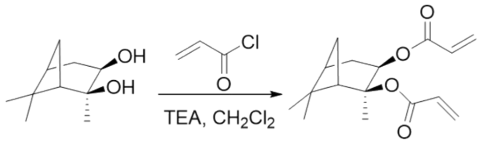


(1S,2S,3R,5S)-
(+)-2,3-pinanediol 2

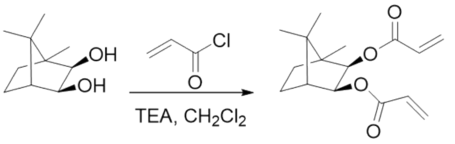


(+)-exo,exo-
2,3-camphanediol 3

Compound **1**: $^1$HNMR (400 MHz, $CDCl_3$): δ 6.35 (dd, J = 17.3, 1.5 Hz, 2H), 6.08 (dd, J = 17.3, 10.6 Hz, 2H), 5.79 (dd, J = 10.5, 1.5 Hz, 2H), 5.15 – 5.07 (m, 2H), 1.95 – 1.81 (m, 2H), 1.74 – 1.57 (m, 4H), 1.52 - 1.38 (m, 2H). $^{13}$C NMR (100 MHz, $CDCl_3$) δ 165.44, 130.78, 128.72, 71.29, 27.79, 21.76. FTMS-ESI (m/z): $[M + Na]^+$ calculated for $C_{12}H_{16}O_4Na$, 247.0946; observed 247.0941.

Compound **2**: $^1$HNMR (400 MHz, $CDCl_3$): δ 6.41 (dd, J = 17.3, 1.5 Hz, 1H), 6.28 (dd, J = 17.3, 1.7 Hz, 1H), 6.14 (dd, J = 17.3, 10.4 Hz, 1H), 6.01 (dd, J = 17.2, 10.3 Hz, 1H), 5.83 (dd, J = 10.4, 1.5 Hz, 1H), 5.72 (dd, J = 10.4, 1.7 Hz, 1H), 5.39 (dd, J = 9.4, 6.0 Hz, 1H), 2.82 (t, J = 5.8 Hz, 1H), 2.53 – 2.41 (m, 1H), 2.27 – 2.16 (m, 1H), 2.04 – 1.94 (m, 1H), 1.82 – 1.75 (m, 1H), 1.69 (s, 3H), 1.39 (d, J = 10.5 Hz, 1H), 1.29 (s, 3H), 1.04 (s, 3H). $^{13}$C NMR (100 MHz, $CDCl_3$) δ 165.77, 164.69, 130.85, 130.27, 129.76, 128.74, 85.39, 71.31, 52.06, 40.11, 38.70, 34.54, 28.20, 27.92, 25.51, 24.10. FTMS-ESI (m/z): $[M + Na]^+$ calculated for $C_{16}H_{22}O_4Na$, 301.1416; observed 301.1410.

Compound **3**: $^1$HNMR (400 MHz, $CDCl_3$): δ 6.38 – 6.15 (m, 2H), 6.10 – 5.87 (m, 2H), 5.83 – 5.64 (m, 2H), 4.89 (s, 2H), 1.89 (d, J = 4.9 Hz, 1H), 1.82 – 1.67 (m, 1H), 1.65 – 1.48 (m, 1H), 1.30 – 1.16 (m, 2H), 1.14 (s, 3H), 0.85 (s, 3H), 0.83 (s, 3H). $^{13}$C NMR

(100 MHz, $CDCl_3$) δ 165.14, 165.04, 130.74, 130.66, 128.58, 128.45, 79.87, 77.31, 49.67, 48.90, 47.58, 32.96, 23.93, 21.01, 20.53, 11.06. FTMS-ESI (m/z): $[M + Na]^+$ calculated for $C_{16}H_{22}O_4Na$, 301.1416; observed 301.1410.

## Gel Preparation

DN gels were prepared by the conventional two-step sequential polymerization as shown in Figure S5. The precursor N,N-dimethylformamide solution containing AMPS (1 mol/L, monomer), ABVN (0.01 mol/L, initiator) and different diacrylate crosslinkers (16 mol% in relative to monomer) was injected into the mold with two parallel glass plants and 0.5-mm silicone spacers. Then the mold was kept at 44°C for 10 hours to form PAMPS single network (SN) hydrogel. The resulting PAMPS SN hydrogel was then immersed into aqueous solution containing AAM (2 mol/L, monomer), MBA (0.0002 mol/L, crosslinker), VA-044 (0.001 mol/L, initiator) for 1 day until equilibrium was reached. The equilibrated PAMPS SN hydrogel was sandwiched by two glass substrates and then kept at 44°C for 10 hours to form DN hydrogels. The as-prepared DN gel was then swelled in a large amount of water to remove the residual and to reach the equilibrium state.

## Mechanical Test

Uniaxial tensile tests in air were performed using a commercial tensile tester (Instron 5965, Instron Co.). The gels, with a thickness of 1.7 mm, were cut into dumbbell-shaped specimens using a metal cutter. Two gauge dimensions were used: one with a gauge length of 17 mm and a gauge width of 4 mm, and another with a gauge length of 12 mm and a gauge width of 2 mm. These specimens were then stretched in air at a crosshead velocity of 100 mm/min, corresponding to a strain rate of approximately 0.1 $s^{-1}$.

Uniaxial tensile tests in glove box were performed using a commercial tensile tester (RTC-1150A, Orientec Co.).

## Evaluation of Mechanoradicals by Fenton-related Chemical Reaction

The mechanoradical concentration was estimated by the Fenton color reaction as reported in our previous work. DN gels were cut into dumbbell-shaped strips (17 mm gauge length and 4 mm width). The cut gels were immersed in a large amount of aqueous solution of 250 μM $(NH_4)_2Fe^{II}(SO_4)_2$, 250 μM XO and 20 mM $H_2SO_4$ for 1 day. Then gels were stretched in air to a preset strain at a velocity of 100 mm/min and then immediately recovered to the initial position. After 30 mins, when no further color change was observed, the gels were cut with scissors to obtain the gel in the necked region. The gel sample was then placed in a quartz cuvette and UV–Vis absorption

spectroscopy (UV-1800, Shimadzu Co.) was performed.

To quantify the $Fe^{3+}$ concentration, a calibration experiment was done by immersing DN gels in a large amount of aqueous solution of 0 - 200 μM $NH_4Fe^{III}(SO_4)_2$ (as a $Fe^{3+}$ source), 250 μM $(NH_4)^2Fe^{II}(SO_4)_2$, 250 μM XO and 20 mM $H_2SO_4$ to get the calibration curve of normalized absorbance (measured absorbance divided by the gel thickness) at 580 nm as a function of $Fe^{3+}$ concentration (Figure S6). Here, we also assume the concentration of $NH_4Fe^{III}(SO_4)_2$, $(NH_4)_2Fe^{II}(SO_4)_2$ and XO in the equilibrated gel was the same as that in the solution. By comparing the normalized absorbance at 580 nm of DN gels after stretching with the calibration curve, we can evaluate the $Fe^{3+}$ concentration, which is assumed to be equal to the mechanoradical concentration.

**Application of Mechanoradicals for Fast Mechanoresponsive Strengthening of DN gels**

Various DN gels with different crosslinkers were immersed in an aqueous solution containing 2.0 M NIPAm and 0.15 M MBA for one day. After immersion, the samples were cut into dumbbell-shaped strips with a gauge length of 12 mm and a width of 2 mm. The strips were then placed in an argon glove box for 2 hours to remove oxygen. Following this, the gels underwent a cyclic tensile test with a preset strain of 6 at a velocity of 300 mm/min. After a 3-minute waiting period, the gels were subjected to a second cyclic tensile test under the same conditions.

**Evaluation of Thermal and UV Stability of Diols**

15 mg of diols were dissolved in ~0.5 ml of $d_6$-DMSO. The solution was then either heated at 80°C or exposed to UV light (3.9 Mw/$cm^2$) for 10 hours. After the treatment, NMR was used to analyze if any reaction has occurred.

## 2.2. Supporting Experiment Results (Figs. S5-S7, Scheme 2)

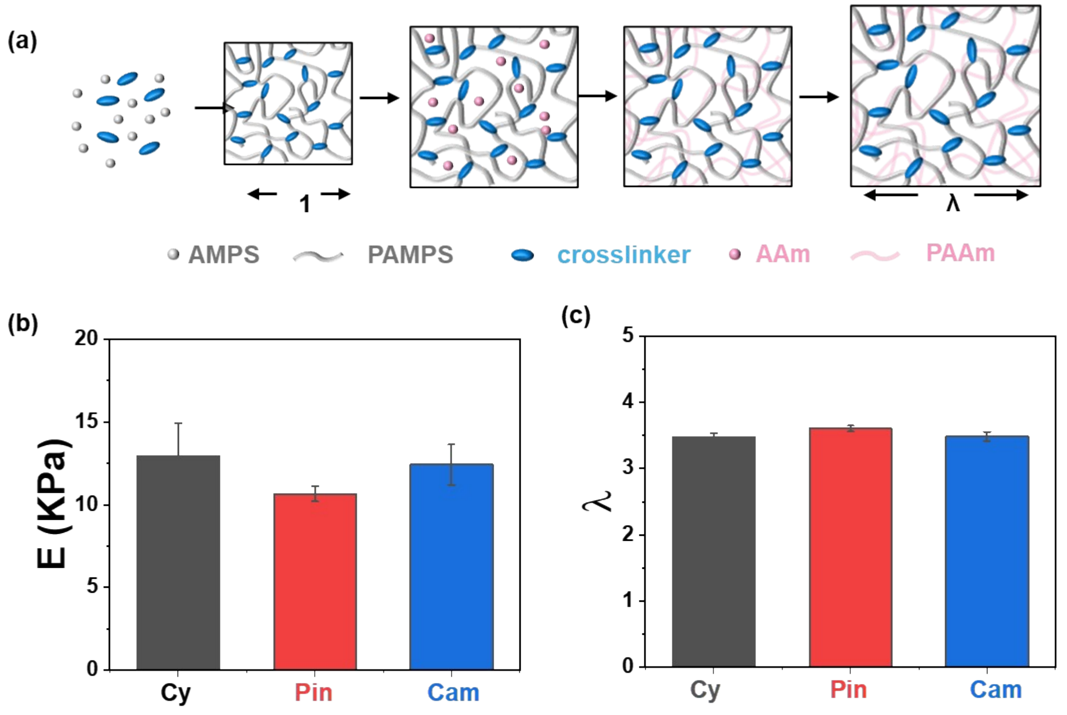


**Figure S6.** (a) Illustration of preparation of DN gels with different crosslinkers. (b) Modulus, E, of as-prepared single network PAMPS hydrogels. (c) Length swelling ratio, λ, of PAMPS network in equilibrated DN gels.

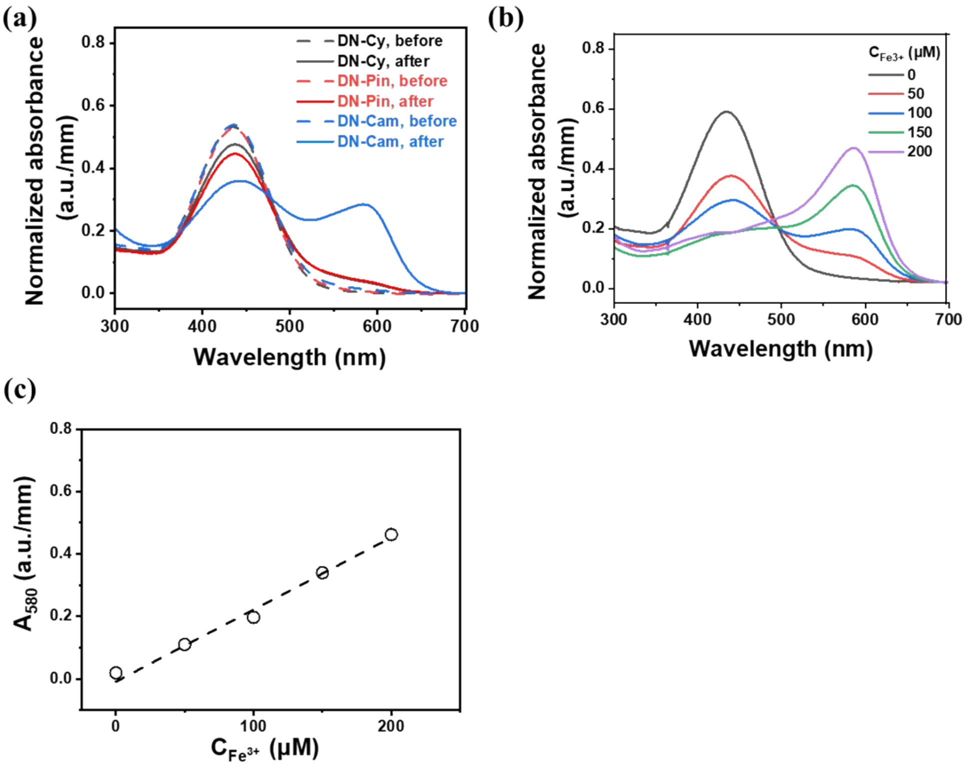


**Figure S7**. (a) UV spectrums of DN-Cy, DN-Pin, and DN-Cam gels fed with 250 μM $(NH_4)_2Fe^{II}(SO_4)_2$ and 250 μM xylenol orange (XO) before and after stretching. (b) UV spectra of equilibrated DN gels after immersion into a large amount of aqueous solution of 0 - 200 μM $NH_4Fe^{III}(SO_4)_2$ (as a $Fe^{3+}$ source), 250 μM $(NH_4)_2Fe^{II}(SO_4)_2$, 250 μM XO and 20 mM $H_2SO_4$. (c) The calibration curve of the normalized absorbance at 580 nm ($A_{580}$) as a function of the $Fe^{3+}$ concentration ($C_{Fe}{}^{3+}$).

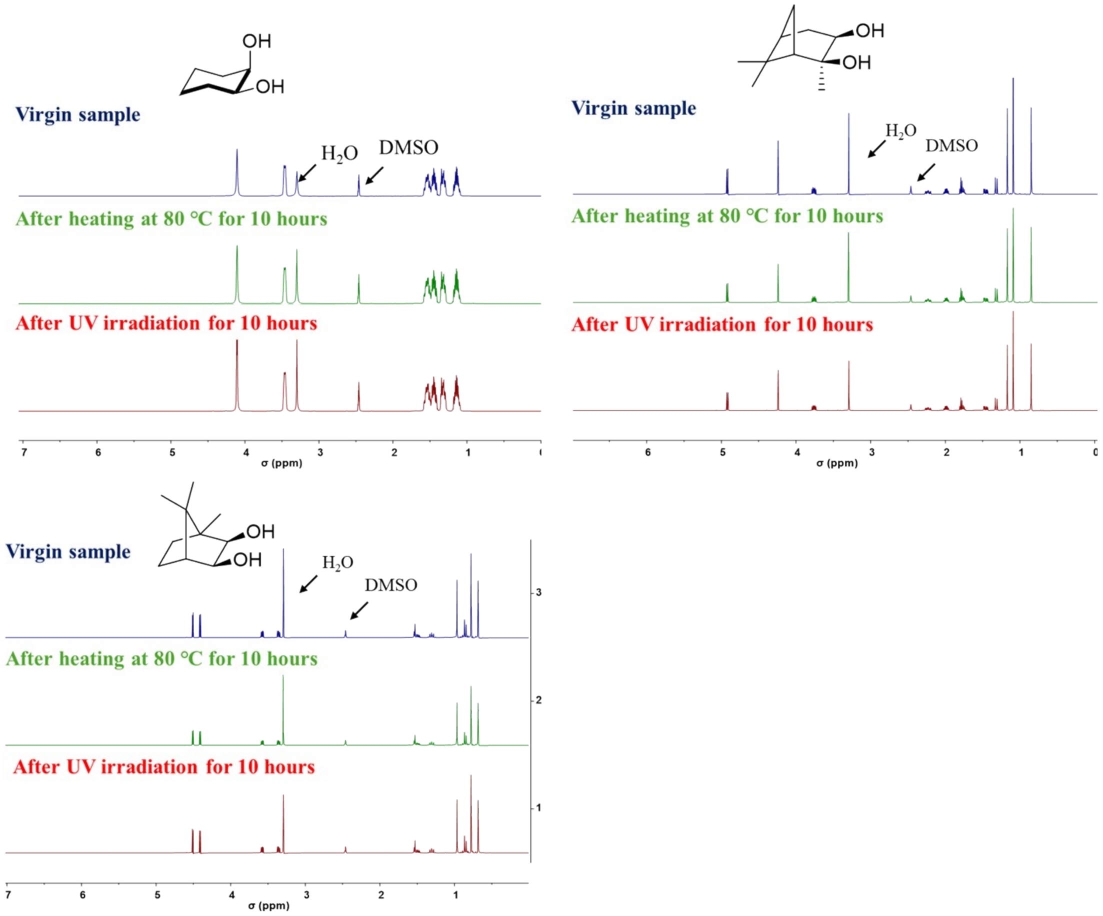


**Figure S8.** NMR spectra of three diols before and after thermal and UV treatment. After heating or UV irradiation, NMR of three diols remains unchanged.

## 2.3. NMR Spectra

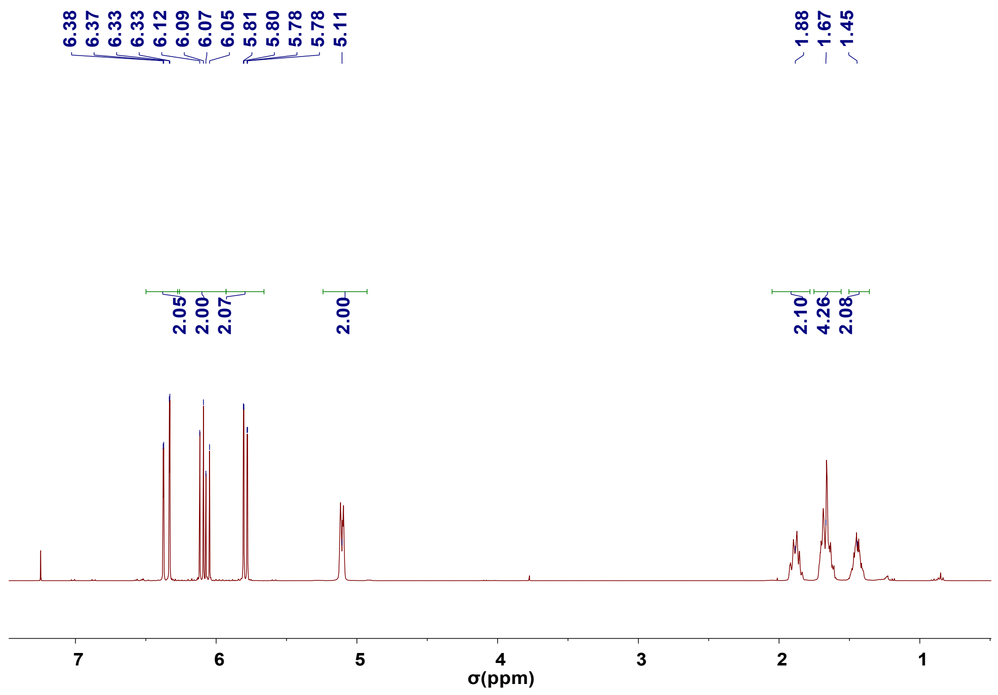


$^{1}$HNMR of compound **1**

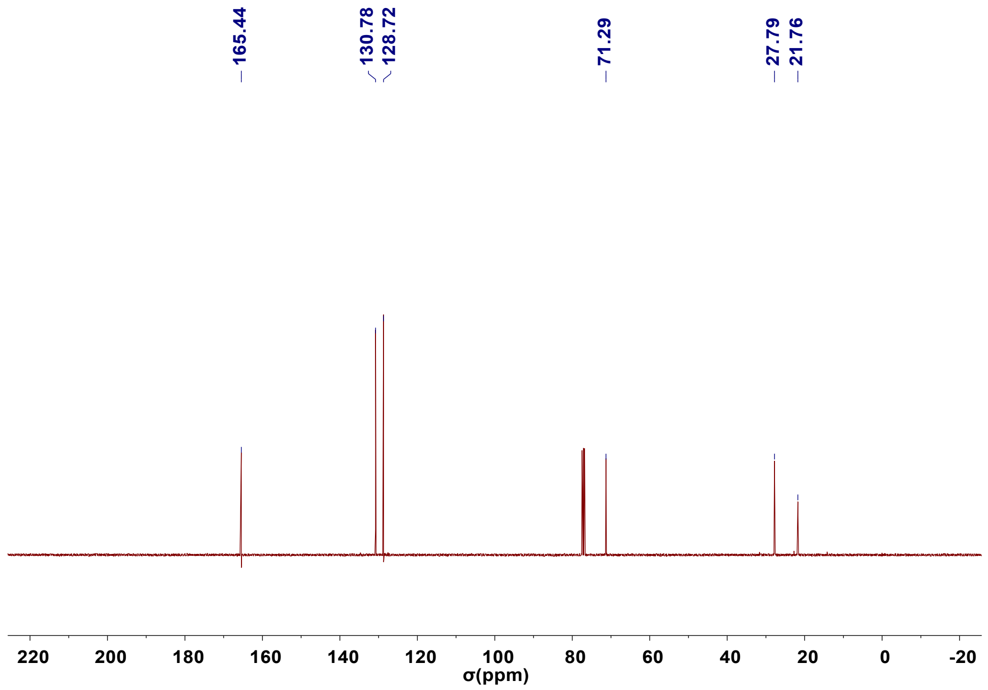


$^{13}$CNMR of compound **1**

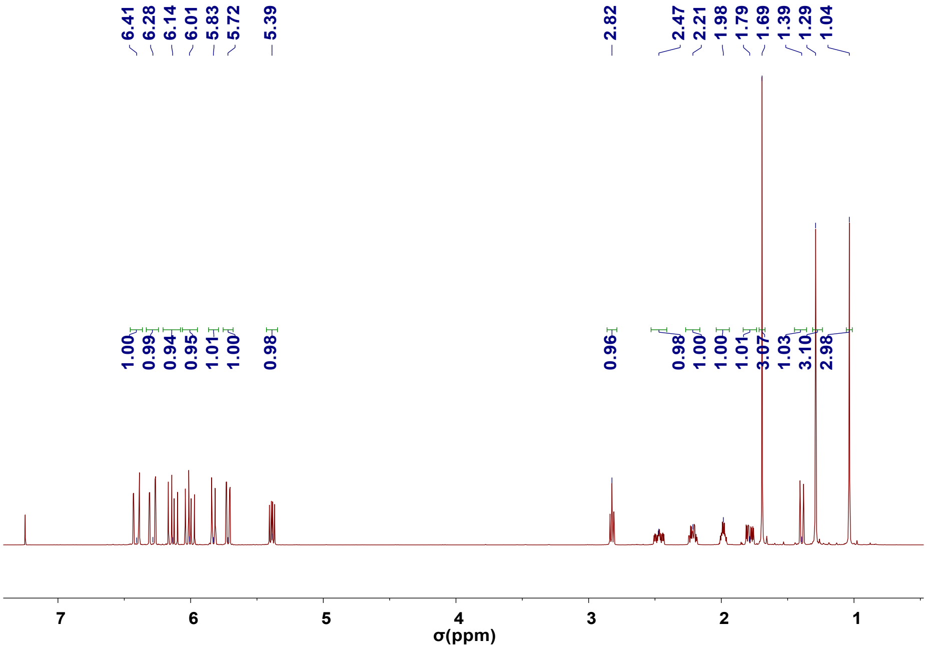


$^{1}$HNMR of compound **2**

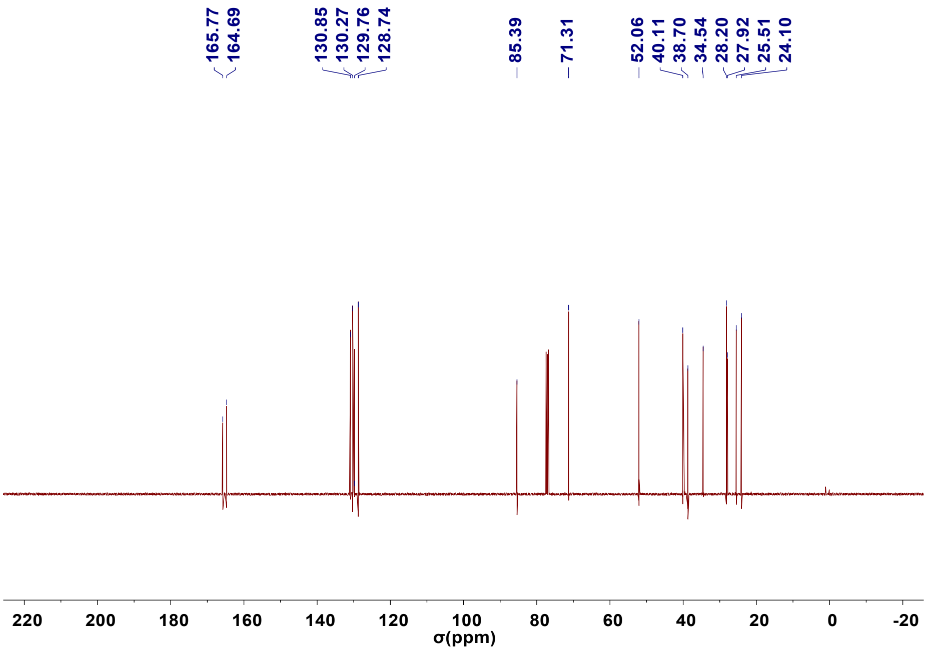


$^{13}$CNMR of compound **2**

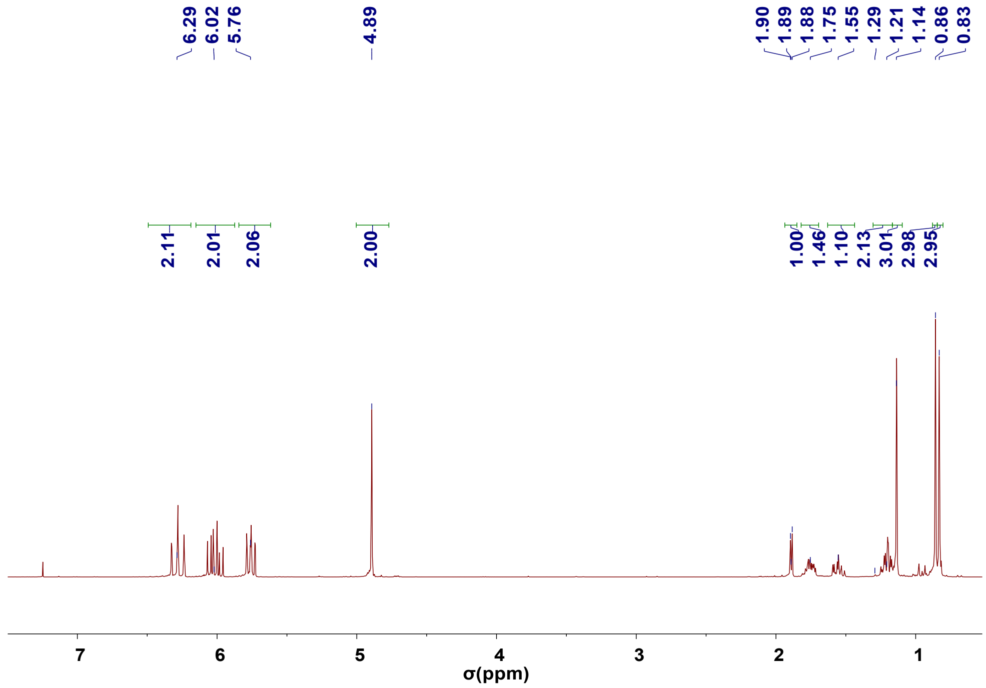


$^{1}$HNMR of compound **3**

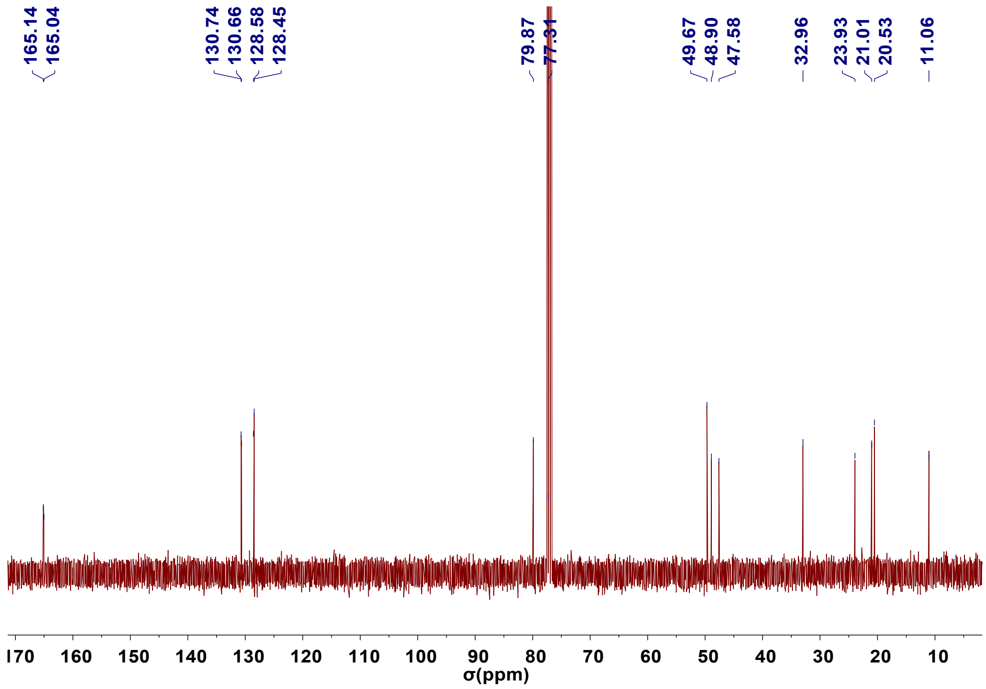


$^{13}$CNMR of compound **3**

**Cartesian Coordinates of Optimized Structures**

($E_F$: Electronic Energy on the FMPES at $F = F_\tau$ pN; $G_F$: Gibbs Free Energy at $F = F_\tau$ pN, 298.15 K and 1 atm)

cisVAPNB_TS-F3500

($E_F$ = -840.62520779 a.u.; $G_F$ = -840.28835867 a.u.)

0 1

| | | | |
|---|---|---|---|
| C | 2.127266544069 | 0.088711514564 | -5.321031576641 |
| C | 3.161680866472 | 0.565836335188 | -4.252049699899 |
| C | 1.697319120388 | -0.777681512228 | -3.083898159789 |
| C | 1.239310127482 | -0.937895968043 | -4.547005976103 |
| H | 1.515570067732 | 0.945370082690 | -5.627055908275 |
| H | 0.170834635117 | -0.748241287459 | -4.675469676274 |
| H | 1.422407838442 | -1.967866882133 | -4.874742717338 |
| C | 3.216705876631 | -0.611000203104 | -3.258880460433 |
| H | 3.727629422068 | -0.348707746788 | -2.329152414556 |
| H | 3.686317111293 | -1.504680698557 | -3.682093573747 |
| C | 2.667634841721 | 1.767909488835 | -3.455813644856 |
| H | 1.918346122229 | 2.354615123842 | -3.987086717525 |
| C | 1.162833587499 | 0.512059874021 | -2.458004403291 |
| H | 0.363122452904 | 0.958608239893 | -3.049910171299 |
| H | 4.146363174649 | 0.783497643665 | -4.678890417133 |
| H | 1.438855556756 | -1.642959715288 | -2.466078341015 |
| C | 3.841433213702 | 2.712082633857 | -2.990125126474 |
| H | 3.430978609846 | 3.488649118023 | -2.337876880140 |
| H | 4.536051731283 | 2.131183580691 | -2.373773002296 |
| C | 0.657393064501 | 0.313613067103 | -0.980120025200 |

| | | | |
|---|---|---|---|
| H | 1.489216302040 | -0.059761225590 | -0.372781214228 |
| H | 0.387889104887 | 1.289243828958 | -0.564463935635 |
| C | 4.775009581602 | 3.494721287249 | -4.103229522847 |
| H | 5.490869140102 | 4.105166036266 | -3.550895354251 |
| H | 5.316211601305 | 2.785316878939 | -4.731382072698 |
| H | 4.164374391600 | 4.138961403561 | -4.739314732012 |
| C | -0.615886466401 | -0.686230565380 | -0.663588385315 |
| H | -0.394520085771 | -1.711270183676 | -0.965595756287 |
| H | -0.789173845526 | -0.656966431861 | 0.413184911292 |
| H | -1.514043605297 | -0.347326890452 | -1.184289632047 |
| Si | 2.901615243446 | -0.575207541489 | -6.918469428515 |
| C | 4.117067793965 | -1.971809432202 | -6.538029013702 |
| H | 3.626903666217 | -2.799881933770 | -6.017498458499 |
| H | 4.550106914490 | -2.369548306251 | -7.461148623082 |
| H | 4.939691823855 | -1.620721355959 | -5.908124689796 |
| C | 3.818711210902 | 0.830979788941 | -7.786141845080 |
| H | 4.615538183112 | 1.234534731049 | -7.154096330876 |
| H | 4.274841694047 | 0.490837050474 | -8.720995123578 |
| H | 3.137007403216 | 1.653015336950 | -8.026110367328 |
| C | 1.528675738980 | -1.226590137945 | -8.040552704770 |
| H | 1.935301033832 | -1.619461117236 | -8.977572705154 |
| H | 0.971402426704 | -2.032956897069 | -7.554058548954 |
| H | 0.817212759876 | -0.433384969573 | -8.290917872917 |

cisVAPNB_P-F3500

($E_F$ = -840.84782002 a.u.; $G_F$ = -840.52107887 a.u.)

0 1

| | | | |
|---|---|---|---|
| C | 2.189857255169 | 0.483266945730 | -4.925869785244 |
| C | 3.155804520085 | 1.153445152356 | -3.876247854764 |
| C | 1.418323669275 | -0.195990591010 | -2.586218434269 |
| C | 1.261068994217 | -0.495702553376 | -4.127221115189 |
| H | 1.560434413153 | 1.295853156122 | -5.312665325579 |
| H | 0.222548997110 | -0.356861630505 | -4.438591868337 |
| H | 1.499902473144 | -1.546753601621 | -4.315436930634 |
| C | 2.163385024475 | 1.209450191319 | -2.621428991410 |
| H | 1.435233054735 | 2.012552799512 | -2.785742665106 |
| H | 2.686477929846 | 1.419809012902 | -1.685197052083 |
| C | 3.858923991891 | 2.436187466025 | -4.407722302792 |
| H | 3.216977089032 | 3.298549769850 | -4.576767613337 |
| C | 0.108526867231 | -0.405887154496 | -1.770291410676 |
| H | -0.608213888641 | 0.413516040186 | -1.782819645071 |
| H | 3.940698178512 | 0.431263976837 | -3.615183176735 |
| H | 2.150802019612 | -0.916514063780 | -2.199727529752 |
| C | 5.374979305087 | 2.576771568167 | -4.729151221731 |
| H | 5.898660605020 | 2.662058604138 | -3.763230941618 |
| H | 5.717101705354 | 1.612350231699 | -5.127638659611 |
| C | -0.383485754493 | -1.809174155028 | -1.306399617534 |
| H | -0.471317766544 | -2.428876392183 | -2.214241286107 |
| H | 0.445243432369 | -2.271910772658 | -0.752305019739 |
| C | 6.049142138524 | 3.723903409350 | -5.674702150203 |
| H | 7.126258813780 | 3.552316526115 | -5.671361982735 |
| H | 5.678342893450 | 3.662809243960 | -6.699955618160 |

| | | | |
|---|---|---|---|
| H | 5.845004817214 | 4.723513500811 | -5.285327708363 |
| C | -1.733085040278 | -2.123317194723 | -0.447115167457 |
| H | -2.626793692307 | -1.793029587440 | -0.981027578454 |
| H | -1.781377224631 | -3.203807373673 | -0.305544757639 |
| H | -1.704517710574 | -1.634876607252 | 0.529215918364 |
| Si | 2.999658590154 | -0.335005805442 | -6.438841943941 |
| C | 4.375052642994 | -1.494189886718 | -5.859323811036 |
| H | 4.000079934634 | -2.219760010044 | -5.130681340657 |
| H | 4.796171260037 | -2.053891563470 | -6.700046853332 |
| H | 5.190219682625 | -0.938676535144 | -5.386597857291 |
| C | 3.700035310919 | 0.954689463507 | -7.627148751629 |
| H | 4.498149302748 | 1.541527968208 | -7.169365272109 |
| H | 4.104703897051 | 0.472400139783 | -8.522928959759 |
| H | 2.918014917898 | 1.650110022685 | -7.947350492722 |
| C | 1.687630834438 | -1.332381476552 | -7.366228628653 |
| H | 2.112590980110 | -1.790009304111 | -8.265152447238 |
| H | 1.273448837904 | -2.132891988450 | -6.747105924272 |
| H | 0.858241901804 | -0.690731537606 | -7.680310839061 |

cisVAPNB_R-F3500

($E_F$ = -840.62838971 a.u.; $G_F$ = -840.28979007 a.u.)

0 1

| | | | |
|---|---|---|---|
| C | 2.116845506219 | 0.059541295413 | -5.352661739672 |
| C | 3.134360234130 | 0.565338009285 | -4.281481115871 |
| C | 1.746002970187 | -0.775508683535 | -3.109492984522 |
| C | 1.255304118487 | -0.976588732525 | -4.554828803448 |

| | | | |
|---|---|---|---|
| H | 1.484944188320 | 0.900102280358 | -5.664605462156 |
| H | 0.181919497126 | -0.801696576925 | -4.663018135413 |
| H | 1.447304735650 | -2.007825591882 | -4.874020295302 |
| C | 3.258943712142 | -0.630970895399 | -3.322894583854 |
| H | 3.803538058891 | -0.384228926753 | -2.407475889867 |
| H | 3.714161500634 | -1.515454809185 | -3.775354932092 |
| C | 2.503753613283 | 1.651452227426 | -3.374238972318 |
| H | 1.874896905929 | 2.316369001598 | -3.973244118704 |
| C | 1.300435281355 | 0.620017895183 | -2.580688987966 |
| H | 0.405037219028 | 0.930776138266 | -3.127781538820 |
| H | 4.078806935817 | 0.901454743396 | -4.711548231133 |
| H | 1.453572808783 | -1.586424640261 | -2.440622243105 |
| C | 3.654844991098 | 2.581725649634 | -2.785379371079 |
| H | 3.199080518966 | 3.326282434585 | -2.125002176707 |
| H | 4.316619725106 | 1.980875980692 | -2.152408401633 |
| C | 0.825202742981 | 0.504228999704 | -1.070043906450 |
| H | 1.664015751844 | 0.154192228264 | -0.457837703733 |
| H | 0.572074535871 | 1.502661551815 | -0.698957764444 |
| C | 4.626437246661 | 3.407083661538 | -3.820960768827 |
| H | 5.265918650403 | 4.058363743640 | -3.223183583653 |
| H | 5.253973849300 | 2.730659139694 | -4.402558428990 |
| H | 4.034290453086 | 4.018019329335 | -4.506537948615 |
| C | -0.451259793040 | -0.463913988812 | -0.715179101290 |
| H | -0.235372035864 | -1.507466476663 | -0.948894301752 |
| H | -0.649530330343 | -0.371727685397 | 0.353767245604 |
| H | -1.338152862804 | -0.152810348963 | -1.272395907106 |

| | | | |
|---|---|---|---|
| Si | 2.895031353332 | -0.600018260924 | -6.949602007651 |
| C | 4.141178025674 | -1.972660153151 | -6.581980707614 |
| H | 3.674615290548 | -2.808717956462 | -6.052755562801 |
| H | 4.567882493230 | -2.364052591976 | -7.510797930475 |
| H | 4.966081934446 | -1.604275784569 | -5.965231488693 |
| C | 3.778325873034 | 0.818783288447 | -7.831851866080 |
| H | 4.573896150084 | 1.238623542219 | -7.208868527652 |
| H | 4.231682813438 | 0.483063068438 | -8.769650612087 |
| H | 3.079928721310 | 1.627494774725 | -8.068655067921 |
| C | 1.523734630010 | -1.281276734942 | -8.056185118853 |
| H | 1.928391714027 | -1.672014865906 | -8.994932138252 |
| H | 0.985436600385 | -2.094656200369 | -7.560242521632 |
| H | 0.795986713932 | -0.502254787976 | -8.303816120260 |

cisVAPNB_TS-F3000

($E_F$ = -840.53661626 a.u.; $G_F$ = -840.19998312 a.u.)

0 1

| | | | |
|---|---|---|---|
| C | 2.130022190758 | 0.098617668217 | -5.307901632880 |
| C | 3.171047638638 | 0.569902772875 | -4.242458190161 |
| C | 1.675634425824 | -0.779704638346 | -3.072027309877 |
| C | 1.236796079367 | -0.928122827675 | -4.543184923505 |
| H | 1.522182049028 | 0.958987819681 | -5.610440449097 |
| H | 0.169627754267 | -0.739735780328 | -4.683148141254 |
| H | 1.423983209227 | -1.956908162223 | -4.871922999074 |
| C | 3.197843720079 | -0.593365691578 | -3.228138396482 |
| H | 3.690884400597 | -0.318179872780 | -2.293060450431 |

| | | | |
|---|---|---|---|
| H | 3.678918326378 | -1.490595340876 | -3.633282301811 |
| C | 2.732643076439 | 1.815885107006 | -3.503815016305 |
| H | 1.945732521577 | 2.380185340004 | -4.000909328196 |
| C | 1.093636475268 | 0.459998651506 | -2.416729271703 |
| H | 0.328569624484 | 0.953637689043 | -3.014414201927 |
| H | 4.169841625879 | 0.732775967277 | -4.665936444677 |
| H | 1.440245265361 | -1.667936167336 | -2.475234042808 |
| C | 3.896872831194 | 2.744386036575 | -3.048129923154 |
| H | 3.494756120477 | 3.523414317385 | -2.393412771120 |
| H | 4.596651557690 | 2.162359128301 | -2.438193394971 |
| C | 0.613599320170 | 0.258714408962 | -0.950610538941 |
| H | 1.449176840599 | -0.125143276552 | -0.354653403837 |
| H | 0.353268995596 | 1.232200826637 | -0.523988545591 |
| C | 4.800312911928 | 3.506628418356 | -4.173726633328 |
| H | 5.541614041063 | 4.113121984469 | -3.650638513232 |
| H | 5.314566914399 | 2.789305993539 | -4.816042453650 |
| H | 4.180129728317 | 4.155012328411 | -4.796528493365 |
| C | -0.650138872716 | -0.729785107853 | -0.651714875221 |
| H | -0.436109345820 | -1.749103018552 | -0.978692968897 |
| H | -0.826029776447 | -0.729176885020 | 0.425425913546 |
| H | -1.548606790560 | -0.376471913351 | -1.162595961828 |
| Si | 2.904801127247 | -0.562969019253 | -6.906271068468 |
| C | 4.113910006522 | -1.963570672029 | -6.521045440344 |
| H | 3.617976600192 | -2.790663349337 | -6.004551754919 |
| H | 4.551364780585 | -2.361596336990 | -7.441897029706 |
| H | 4.933692696638 | -1.616109976854 | -5.885512366094 |

| | | | |
|---|---|---|---|
| C | 3.828176384998 | 0.842545313628 | -7.767706228959 |
| H | 4.621941160026 | 1.244694980699 | -7.131133854455 |
| H | 4.288642389766 | 0.502852550655 | -8.700545003471 |
| H | 3.148672701473 | 1.665484044838 | -8.010241790281 |
| C | 1.533981856379 | -1.208601330051 | -8.033902846913 |
| H | 1.942464749281 | -1.599025931787 | -8.971119766601 |
| H | 0.974292427582 | -2.015613721023 | -7.551535088205 |
| H | 0.824486236207 | -0.413570285562 | -8.283562396368 |

cisVAPNB_P-F3000

($E_F$ = -840.72167371 a.u.; $G_F$ = -840.39413000 a.u.)

0 1

| | | | |
|---|---|---|---|
| C | 2.173472439021 | 0.509732895006 | -4.962869387480 |
| C | 3.138999202612 | 1.179578367552 | -3.910918739309 |
| C | 1.442897455011 | -0.184148001108 | -2.625629031008 |
| C | 1.198493972271 | -0.421186961182 | -4.161396862470 |
| H | 1.578892857652 | 1.327446624867 | -5.390206555856 |
| H | 0.162853012571 | -0.181379030320 | -4.416747184921 |
| H | 1.333939528938 | -1.481808434327 | -4.392879244136 |
| C | 2.176014364014 | 1.216200359876 | -2.643438372667 |
| H | 1.440713247260 | 2.018464709088 | -2.779489920211 |
| H | 2.717440252056 | 1.413208557114 | -1.714645934297 |
| C | 3.801246749966 | 2.473290299559 | -4.431219836684 |
| H | 3.134480062340 | 3.313647516438 | -4.616082168551 |
| C | 0.185110762112 | -0.415203643987 | -1.758785267187 |
| H | -0.525865609456 | 0.406763584352 | -1.695235427813 |

| | | | |
|---|---|---|---|
| H | 3.940752704114 | 0.470103965554 | -3.666687858385 |
| H | 2.193987803087 | -0.920296197873 | -2.308508615778 |
| C | 5.310247567561 | 2.677196893679 | -4.679886957698 |
| H | 5.778320324253 | 2.877710569814 | -3.701393449943 |
| H | 5.733914939087 | 1.710066553207 | -4.981032357853 |
| C | -0.285319103903 | -1.829267799096 | -1.348998552372 |
| H | -0.422413550261 | -2.406013845011 | -2.279431122344 |
| H | 0.562992373620 | -2.325258944776 | -0.855835169195 |
| C | 5.917637460319 | 3.787892966021 | -5.679389770602 |
| H | 7.005766726415 | 3.722593181351 | -5.632290628492 |
| H | 5.593749547557 | 3.614525734053 | -6.707954564942 |
| H | 5.610899799155 | 4.793297136180 | -5.381693441757 |
| C | -1.586608971950 | -2.134835661674 | -0.451215101213 |
| H | -2.493965017226 | -1.759614707503 | -0.930532211140 |
| H | -1.669785744052 | -3.216856504340 | -0.337744635050 |
| H | -1.501486787959 | -1.680234564275 | 0.538529370475 |
| Si | 2.995164028076 | -0.382821921899 | -6.424732854682 |
| C | 4.198230513354 | -1.680286403068 | -5.760237416423 |
| H | 3.699544977304 | -2.366924958311 | -5.069168355748 |
| H | 4.620602327503 | -2.276661118731 | -6.574737142256 |
| H | 5.029521763752 | -1.213234860649 | -5.223961667572 |
| C | 3.913533013852 | 0.820003079243 | -7.554040873727 |
| H | 4.771600127804 | 1.273392729422 | -7.054666867565 |
| H | 4.277870522596 | 0.302434048201 | -8.447453258317 |
| H | 3.254037961702 | 1.629281890357 | -7.881724891094 |
| C | 1.647695245075 | -1.235809672961 | -7.440683302836 |

| | | | |
|---|---|---|---|
| H | 2.078978799796 | -1.751294554411 | -8.304545554652 |
| H | 1.101542536703 | -1.975214447749 | -6.848308320477 |
| H | 0.921901150674 | -0.507496956212 | -7.816232366596 |

cisVAPNB_R-F3000

($E_F$ = -840.54788301 a.u.; $G_F$ = -840.20833049 a.u.)

| | | | |
|---|---|---|---|
| C | 2.118423115186 | 0.062231494258 | -5.368712806837 |
| C | 3.126020413645 | 0.565459205439 | -4.287680171343 |
| C | 1.732127074334 | -0.773590927175 | -3.127058068361 |
| C | 1.243377407815 | -0.966314515114 | -4.574288053605 |
| H | 1.494232746622 | 0.905265402934 | -5.689744255718 |
| H | 0.172257307628 | -0.779204280998 | -4.684581185942 |
| H | 1.424510067234 | -1.998969344028 | -4.895069173798 |
| C | 3.246733094429 | -0.637156062454 | -3.336124391330 |
| H | 3.791226214814 | -0.399112724507 | -2.418360099647 |
| H | 3.698575000439 | -1.520898808697 | -3.792759608991 |
| C | 2.465738554430 | 1.632625367199 | -3.373784780610 |
| H | 1.858122452456 | 2.308317531673 | -3.984022181435 |
| C | 1.305898509918 | 0.631905283172 | -2.602194072583 |
| H | 0.384505727567 | 0.926459307633 | -3.115589328190 |
| H | 4.069838057387 | 0.914725160091 | -4.707491926918 |
| H | 1.428654715164 | -1.582108716827 | -2.460792241909 |
| C | 3.580076939947 | 2.549767063960 | -2.729096853700 |
| H | 3.098700885740 | 3.271618085513 | -2.061073767403 |
| H | 4.234923740073 | 1.942003552449 | -2.095083860360 |
| C | 0.903818572068 | 0.550060027147 | -1.080265079447 |

| | | | |
|---|---|---|---|
| H | 1.762619529664 | 0.196652807811 | -0.497800980015 |
| H | 0.680719064264 | 1.557651379827 | -0.713630670677 |
| C | 4.535729601989 | 3.392140338216 | -3.732376208034 |
| H | 5.160362927170 | 4.056455247236 | -3.132511504156 |
| H | 5.183223337930 | 2.734932777497 | -4.314475810000 |
| H | 3.938907604935 | 3.995136980119 | -4.421721418097 |
| C | -0.359556725554 | -0.388148680406 | -0.694025202613 |
| H | -0.164173936430 | -1.436503504035 | -0.925071229021 |
| H | -0.544049060304 | -0.293199236809 | 0.377559742163 |
| H | -1.254287213057 | -0.071767960149 | -1.236487362897 |
| Si | 2.905209954606 | -0.606556616815 | -6.957730407781 |
| C | 4.131901355674 | -1.994572689722 | -6.582399190881 |
| H | 3.652213333395 | -2.823764660188 | -6.054115028744 |
| H | 4.557044971580 | -2.392712885590 | -7.509084109706 |
| H | 4.959215101357 | -1.636396503058 | -5.962913276182 |
| C | 3.812547070204 | 0.802011438472 | -7.831947848224 |
| H | 4.610139276616 | 1.209741069717 | -7.203547794354 |
| H | 4.266857939254 | 0.461875808248 | -8.767692112233 |
| H | 3.126624876639 | 1.620587207144 | -8.071298509775 |
| C | 1.535224898643 | -1.271175929533 | -8.076076817192 |
| H | 1.943108388585 | -1.667115380044 | -9.011236454613 |
| H | 0.982716063942 | -2.077779977780 | -7.584685863201 |
| H | 0.819238928165 | -0.483344994734 | -8.330034149471 |

cisVAPNB_TS-F2500

($E_F$ = -840.44717461 a.u.; $G_F$ = -840.11095188 a.u.)

0 1

| | | | |
|---|---|---|---|
| C | 2.130342235671 | 0.101011849029 | -5.303953607466 |
| C | 3.174667847639 | 0.571927981944 | -4.241329067002 |
| C | 1.664048271833 | -0.783711816688 | -3.068523369937 |
| C | 1.237467594587 | -0.928629831126 | -4.543904907599 |
| H | 1.521856942313 | 0.961796961301 | -5.603711165251 |
| H | 0.170484907853 | -0.744882380066 | -4.691130246119 |
| H | 1.430911964672 | -1.956295014236 | -4.872318860481 |
| C | 3.187136388965 | -0.581398182217 | -3.212819118631 |
| H | 3.668744037950 | -0.296202066873 | -2.275001057433 |
| H | 3.677625751519 | -1.479911975814 | -3.605080648704 |
| C | 2.764545057603 | 1.840372429519 | -3.535630725021 |
| H | 1.961055375012 | 2.396678327545 | -4.013340474183 |
| C | 1.053139408265 | 0.428790900867 | -2.401918433642 |
| H | 0.304476862348 | 0.945220268055 | -3.000015964744 |
| H | 4.179468621468 | 0.705562427308 | -4.663589496306 |
| H | 1.442816159744 | -1.683684208764 | -2.482525709665 |
| C | 3.917514108297 | 2.751599674321 | -3.064821701474 |
| H | 3.516319973618 | 3.526128228698 | -2.403862761442 |
| H | 4.616106100901 | 2.162480692639 | -2.459866126587 |
| C | 0.603519154252 | 0.240298036598 | -0.939087739749 |
| H | 1.444327768069 | -0.149761664837 | -0.354132680083 |
| H | 0.355600276073 | 1.216178033617 | -0.510033975047 |
| C | 4.799515083227 | 3.504187753231 | -4.188604294871 |
| H | 5.556603010083 | 4.108331493225 | -3.684723557389 |
| H | 5.298005680917 | 2.785613267771 | -4.842569924851 |

| | | | |
|---|---|---|---|
| H | 4.173316418823 | 4.156599922920 | -4.801652972055 |
| C | -0.652567449850 | -0.731906398747 | -0.649283103884 |
| H | -0.447973267769 | -1.748828718704 | -0.990754228489 |
| H | -0.833434565972 | -0.749459191953 | 0.427392857768 |
| H | -1.549965548747 | -0.367481014776 | -1.154913373142 |
| Si | 2.905785171529 | -0.557865550696 | -6.903126921247 |
| C | 4.113866732312 | -1.958622308751 | -6.515067115481 |
| H | 3.616117059965 | -2.786148903859 | -6.001015433771 |
| H | 4.554899385432 | -2.355985417928 | -7.434482269998 |
| H | 4.931158196820 | -1.611584838840 | -5.876079683527 |
| C | 3.830391131262 | 0.848283870870 | -7.762013120194 |
| H | 4.622654517635 | 1.250452476195 | -7.123584323996 |
| H | 4.292859642346 | 0.509128814049 | -8.694069801977 |
| H | 3.151098302016 | 1.671111437439 | -8.005520714397 |
| C | 1.536783757032 | -1.203115628683 | -8.033111893634 |
| H | 1.946737358650 | -1.593133398329 | -8.969858544972 |
| H | 0.976413475079 | -2.010368624287 | -7.551943527730 |
| H | 0.827637076502 | -0.408015668266 | -8.283550514147 |

cisVAPNB_P-F2500

($E_F$ = -840.59720961 a.u.; $G_F$ = -840.26903833 a.u.)

0 1

| | | | |
|---|---|---|---|
| C | 2.165580183221 | 0.507797334661 | -4.976829272948 |
| C | 3.126445827340 | 1.177002084553 | -3.920776444602 |
| C | 1.451522512666 | -0.180769878285 | -2.641909070478 |
| C | 1.185714868938 | -0.413311296140 | -4.173890181066 |

| | | | |
|---|---|---|---|
| H | 1.577385784005 | 1.325141795302 | -5.413308370785 |
| H | 0.150488186458 | -0.158312128667 | -4.416130177217 |
| H | 1.303935158483 | -1.475131764191 | -4.409032331949 |
| C | 2.173631903763 | 1.215388405444 | -2.656408670470 |
| H | 1.435136163245 | 2.015495560202 | -2.789301125785 |
| H | 2.717964388372 | 1.413472826853 | -1.729478174655 |
| C | 3.775989511310 | 2.469725504708 | -4.425570136777 |
| H | 3.108369542686 | 3.308514576802 | -4.614258056784 |
| C | 0.213381039074 | -0.407258536371 | -1.766119483815 |
| H | -0.494972896971 | 0.415179936935 | -1.683325534477 |
| H | 3.931089658511 | 0.469801651492 | -3.678879374465 |
| H | 2.206971332094 | -0.918496023078 | -2.337806559062 |
| C | 5.278088488095 | 2.676785085526 | -4.645474661787 |
| H | 5.734321174058 | 2.892019646979 | -3.663926069761 |
| H | 5.715724036882 | 1.711989836302 | -4.934575309473 |
| C | -0.249437732836 | -1.818170576234 | -1.377765023391 |
| H | -0.406713986610 | -2.382585152697 | -2.313133960384 |
| H | 0.601439174912 | -2.328216869205 | -0.903041834455 |
| C | 5.848441725586 | 3.781286838527 | -5.645738097107 |
| H | 6.938918647418 | 3.754765099564 | -5.607775671095 |
| H | 5.525384228824 | 3.590247240785 | -6.671700150084 |
| H | 5.512997412710 | 4.780999608020 | -5.358548785408 |
| C | -1.530586448125 | -2.089687131802 | -0.471439366381 |
| H | -2.434772281089 | -1.688119432952 | -0.936050230884 |
| H | -1.649650131180 | -3.167968493791 | -0.351435094413 |
| H | -1.420123182990 | -1.636667186849 | 0.516910858987 |

| | | | |
|---|---|---|---|
| Si | 2.997498375942 | -0.395878065474 | -6.425399324863 |
| C | 4.177155320989 | -1.705157720074 | -5.742407455505 |
| H | 3.662283494242 | -2.385071113484 | -5.056612835464 |
| H | 4.603686910175 | -2.307586622693 | -6.550262195552 |
| H | 5.006457259548 | -1.245891721774 | -5.196401856098 |
| C | 3.946846731740 | 0.795811637037 | -7.540633263172 |
| H | 4.803745682939 | 1.236538594570 | -7.027871730764 |
| H | 4.317403385929 | 0.274831052205 | -8.429463588189 |
| H | 3.303306120088 | 1.614548354943 | -7.876325126366 |
| C | 1.655045216143 | -1.235479884637 | -7.458764734935 |
| H | 2.092527908068 | -1.760178737900 | -8.313909454541 |
| H | 1.090654700439 | -1.965339209515 | -6.871596558620 |
| H | 0.944341478741 | -0.499560191572 | -7.848061212034 |

cisVAPNB_R-F2500

($E_F$ = -840.46961471 a.u.; $G_F$ = -840.12912793 a.u.)

0 1

| | | | |
|---|---|---|---|
| C | 2.120762735026 | 0.065802691244 | -5.387385811859 |
| C | 3.117788245237 | 0.563792489373 | -4.294964597498 |
| C | 1.712511899410 | -0.772987293035 | -3.148035395074 |
| C | 1.227560559230 | -0.952439872218 | -4.598562121015 |
| H | 1.507648456149 | 0.912990254527 | -5.718879787871 |
| H | 0.159755002907 | -0.748939690468 | -4.712050700226 |
| H | 1.394108324697 | -1.986571996313 | -4.922223069273 |
| C | 3.229398083250 | -0.645316922534 | -3.349940254196 |
| H | 3.771728796738 | -0.416766097557 | -2.428529543891 |

| | | | |
|---|---|---|---|
| H | 3.677967489139 | -1.528702490595 | -3.809968216798 |
| C | 2.432503706921 | 1.617147304615 | -3.380464121585 |
| H | 1.839811588510 | 2.299048568532 | -3.999330128714 |
| C | 1.304024318681 | 0.637195599022 | -2.620276968463 |
| H | 0.359993756631 | 0.923384525995 | -3.097006591279 |
| H | 4.063742468492 | 0.921252397235 | -4.702460532249 |
| H | 1.397526376696 | -1.582060023117 | -2.488160554226 |
| C | 3.509456073094 | 2.523266889012 | -2.685479997124 |
| H | 3.002720948756 | 3.219681742188 | -2.008786933520 |
| H | 4.160836415709 | 1.909084150658 | -2.053717387049 |
| C | 0.981688503350 | 0.584452659601 | -1.088614462783 |
| H | 1.862447263366 | 0.226642961955 | -0.542306796003 |
| H | 0.790270376962 | 1.600160261211 | -0.725762649911 |
| C | 4.447505596301 | 3.387798235196 | -3.656789094130 |
| H | 5.063880414772 | 4.055421497727 | -3.051380913299 |
| H | 5.107161717074 | 2.751943814517 | -4.249231930302 |
| H | 3.844652132151 | 3.992442490875 | -4.340040950754 |
| C | -0.265867917566 | -0.325565537198 | -0.660462677990 |
| H | -0.093465498608 | -1.377877254408 | -0.892610413395 |
| H | -0.423562459757 | -0.229473210477 | 0.415690882369 |
| H | -1.173570445931 | -0.001891569401 | -1.177232577328 |
| Si | 2.916873350817 | -0.614803035882 | -6.966959121741 |
| C | 4.117113501604 | -2.023220267646 | -6.582186285903 |
| H | 3.619887961529 | -2.843259659964 | -6.055834313874 |
| H | 4.540274539695 | -2.429820391345 | -7.506124916595 |
| H | 4.947254578923 | -1.679089770012 | -5.958577248826 |

| | | | |
|---|---|---|---|
| C | 3.855728901420 | 0.779878744303 | -7.830040824337 |
| H | 4.655274651861 | 1.171782279268 | -7.194103387974 |
| H | 4.311678257628 | 0.433960921026 | -8.762863217241 |
| H | 3.186419144815 | 1.611061721156 | -8.072936002389 |
| C | 1.548253974979 | -1.256700157361 | -8.100246695507 |
| H | 1.959541102287 | -1.658840129402 | -9.031255638528 |
| H | 0.977680519210 | -2.054518701680 | -7.615158262987 |
| H | 0.847729138715 | -0.457363889418 | -8.361254992425 |

cisVAPNB_TS-F2000

($E_F$ = -840.35810044 a.u.; $G_F$ = -840.02194448 a.u.)

0 1

| | | | |
|---|---|---|---|
| C | 2.129311445020 | 0.099182855734 | -5.306103861533 |
| C | 3.175137811186 | 0.571255373029 | -4.245209059342 |
| C | 1.654484217490 | -0.789395322350 | -3.070849669775 |
| C | 1.237628369818 | -0.933550444044 | -4.549034866179 |
| H | 1.519190698703 | 0.959436033728 | -5.603869625170 |
| H | 0.170656736744 | -0.754562865639 | -4.702129440737 |
| H | 1.437106624157 | -1.960259852216 | -4.876693916285 |
| C | 3.177519269953 | -0.573540433516 | -3.205553086267 |
| H | 3.650410937934 | -0.280327574905 | -2.265800868224 |
| H | 3.676112631675 | -1.472545215938 | -3.587051669879 |
| C | 2.781196141623 | 1.854005259409 | -3.562022135758 |
| H | 1.969363292159 | 2.407697731328 | -4.027737585517 |
| C | 1.023509772339 | 0.406339118941 | -2.399533372488 |
| H | 0.283356798129 | 0.936321161010 | -2.995733281891 |

| | | | |
|---|---|---|---|
| H | 4.183082515294 | 0.686612015435 | -4.666965245480 |
| H | 1.441842971608 | -1.696540076365 | -2.491823507271 |
| C | 3.920624414333 | 2.747447723232 | -3.062641164293 |
| H | 3.515375442536 | 3.513336710779 | -2.393762156832 |
| H | 4.614615996787 | 2.148467863902 | -2.461700616894 |
| C | 0.610638464219 | 0.239419968799 | -0.935191344093 |
| H | 1.458793850300 | -0.154541891970 | -0.363188355640 |
| H | 0.378261256170 | 1.220334022705 | -0.508407636516 |
| C | 4.787565014127 | 3.497850045506 | -4.176330993742 |
| H | 5.554299632598 | 4.100345462224 | -3.684191212333 |
| H | 5.277367513295 | 2.782353336302 | -4.840672475168 |
| H | 4.157700673716 | 4.154498516813 | -4.781569922512 |
| C | -0.638750436564 | -0.714115356851 | -0.644201259133 |
| H | -0.445802527590 | -1.730877083046 | -0.993843963938 |
| H | -0.821657106789 | -0.742356887664 | 0.432393311840 |
| H | -1.536003107964 | -0.340881710801 | -1.144286252806 |
| Si | 2.906391414895 | -0.556917920443 | -6.905489150898 |
| C | 4.115579716371 | -1.956036164949 | -6.514725719759 |
| H | 3.617380250629 | -2.784708687484 | -6.002961942833 |
| H | 4.560655918683 | -2.352098948503 | -7.432736130746 |
| H | 4.929925800901 | -1.608068884649 | -5.872470812891 |
| C | 3.830419398639 | 0.850825131599 | -7.762286303147 |
| H | 4.620323853463 | 1.254497680079 | -7.121896053835 |
| H | 4.295783383883 | 0.512518737450 | -8.693197021137 |
| H | 3.150163370114 | 1.672353919904 | -8.007421525897 |
| C | 1.540101249719 | -1.203934754523 | -8.037702931550 |

| | | | |
|---|---|---|---|
| H | 1.952068829335 | -1.593398882811 | -8.973793549264 |
| H | 0.980004786900 | -2.011929446303 | -7.557467685226 |
| H | 0.830338689418 | -0.409748220229 | -8.289276237527 |

cisVAPNB_R-F2000

($E_F$ = -840.39338548 a.u.; $G_F$ = -840.05209056 a.u.)

0 1

| | | | |
|---|---|---|---|
| C | 2.122648082342 | 0.068048587828 | -5.407042143059 |
| C | 3.109706663593 | 0.560361884994 | -4.303703296053 |
| C | 1.691307758519 | -0.773842461546 | -3.170058861452 |
| C | 1.210986312292 | -0.939482791475 | -4.624217013120 |
| H | 1.521047819186 | 0.919572856791 | -5.748527122018 |
| H | 0.146870046728 | -0.719274918369 | -4.741275901019 |
| H | 1.362732649983 | -1.974819071895 | -4.950986800026 |
| C | 3.210597238425 | -0.654904222749 | -3.364535846589 |
| H | 3.750610781543 | -0.435916460830 | -2.439546153749 |
| H | 3.655736157971 | -1.538171056548 | -3.827655609521 |
| C | 2.402712516596 | 1.602846713471 | -3.391771223252 |
| H | 1.823088156830 | 2.290157144853 | -4.017586287822 |
| C | 1.298638784348 | 0.638952450169 | -2.638702659349 |
| H | 0.335225600259 | 0.919945500499 | -3.079074678528 |
| H | 4.058789192405 | 0.923388150402 | -4.698822873156 |
| H | 1.365084712473 | -1.583925521414 | -2.517050436082 |
| C | 3.442425311839 | 2.496268154370 | -2.646992009568 |
| H | 2.910065725269 | 3.163724781267 | -1.960514676481 |
| H | 4.090821226633 | 1.873918907800 | -2.019928909023 |

| | | | |
|---|---|---|---|
| C | 1.056888049015 | 0.615758847421 | -1.100393980873 |
| H | 1.958800771633 | 0.254109960526 | -0.592223616113 |
| H | 0.897968561177 | 1.639868580472 | -0.745038948462 |
| C | 4.364595994014 | 3.387574952806 | -3.581714585604 |
| H | 4.976115172704 | 4.050053347191 | -2.965146864025 |
| H | 5.032407613385 | 2.775887780934 | -4.190677471556 |
| H | 3.756845333478 | 4.002247604740 | -4.252065483004 |
| C | -0.172871525370 | -0.265332018786 | -0.623310299059 |
| H | -0.026075390291 | -1.321424037250 | -0.856876890217 |
| H | -0.295404009324 | -0.167647579481 | 0.457655391449 |
| H | -1.095540686878 | 0.067184501004 | -1.107616951828 |
| Si | 2.927661774428 | -0.624103244139 | -6.977311901612 |
| C | 4.104455748296 | -2.049544122876 | -6.582954521546 |
| H | 3.591831653009 | -2.861497992671 | -6.058830176361 |
| H | 4.526923605679 | -2.463438790874 | -7.503985433911 |
| H | 4.936017093570 | -1.717227570835 | -5.954875871391 |
| C | 3.894107033808 | 0.757786199616 | -7.830365851038 |
| H | 4.693370729223 | 1.137461558956 | -7.186693334781 |
| H | 4.353625619271 | 0.406076373326 | -8.759259328569 |
| H | 3.239152324416 | 1.598758368126 | -8.078607181589 |
| C | 1.561544993408 | -1.246970640626 | -8.124251162767 |
| H | 1.976433664081 | -1.654551499304 | -9.051284967735 |
| H | 0.975467542159 | -2.037064865753 | -7.645016773799 |
| H | 0.874512040672 | -0.438164742243 | -8.391849790399 |

cisVAPNB_P-F2000

($E_F$ = -840.47430989 a.u.; $G_F$ = -840.14447886 a.u.)

0 1

| | | | |
|---|---|---|---|
| C | 2.152460428764 | 0.513179751713 | -5.003306994116 |
| C | 3.113619598704 | 1.178862439666 | -3.945062144703 |
| C | 1.464397167996 | -0.179715709119 | -2.671918700664 |
| C | 1.154065646369 | -0.382746515412 | -4.197645079131 |
| H | 1.581885192918 | 1.332273333448 | -5.458732453260 |
| H | 0.124351219685 | -0.081755883404 | -4.409238862848 |
| H | 1.222966938595 | -1.444809560368 | -4.450354585223 |
| C | 2.180380771694 | 1.210343877248 | -2.675561966940 |
| H | 1.439691934035 | 2.011221189696 | -2.791801798105 |
| H | 2.735899224317 | 1.399479209753 | -1.753373143880 |
| C | 3.742493545132 | 2.475486345242 | -4.433704062691 |
| H | 3.066180424825 | 3.305946991494 | -4.628621996048 |
| C | 0.257232411953 | -0.408280418239 | -1.772467116624 |
| H | -0.446380769519 | 0.414129101140 | -1.656606292386 |
| H | 3.925361869861 | 0.476343818699 | -3.713254407995 |
| H | 2.225369092756 | -0.925731767727 | -2.402461504220 |
| C | 5.236455694311 | 2.707608977231 | -4.611668613433 |
| H | 5.664025696111 | 2.969916486046 | -3.628129283369 |
| H | 5.708582752761 | 1.747940715718 | -4.860807472973 |
| C | -0.193705145413 | -1.817281835432 | -1.401855815065 |
| H | -0.377651372915 | -2.369721641499 | -2.339899049230 |
| H | 0.663193965769 | -2.339744964456 | -0.951503863548 |
| C | 5.763617989380 | 3.792502413479 | -5.633176881940 |
| H | 6.854061807667 | 3.826386830440 | -5.589872812658 |

| | | | |
|---|---|---|---|
| H | 5.459937756376 | 3.551991679511 | -6.654810856956 |
| H | 5.376402982353 | 4.784342485063 | -5.385156293230 |
| C | -1.447891961188 | -2.057328796897 | -0.476156494678 |
| H | -2.351968751479 | -1.631677621129 | -0.919821786279 |
| H | -1.598839979413 | -3.130857036601 | -0.346912236345 |
| H | -1.304890621925 | -1.605098154122 | 0.508714480770 |
| Si | 2.995459504177 | -0.420770946714 | -6.425190422700 |
| C | 4.098156921587 | -1.775574189037 | -5.703235998180 |
| H | 3.530823430398 | -2.441712677437 | -5.045958985362 |
| H | 4.538500368016 | -2.386927070389 | -6.496830811100 |
| H | 4.918386160383 | -1.349335060875 | -5.117941388159 |
| C | 4.032650321003 | 0.734958554506 | -7.498998636863 |
| H | 4.890786785170 | 1.132182916451 | -6.953355514239 |
| H | 4.409759473048 | 0.204871552925 | -8.379601479663 |
| H | 3.438626939635 | 1.584889075834 | -7.847899084856 |
| C | 1.657075625583 | -1.205284485287 | -7.505488412642 |
| H | 2.101589251449 | -1.755813094151 | -8.340514254346 |
| H | 1.037807386248 | -1.904169064330 | -6.936054030749 |
| H | 0.996357016593 | -0.440204600621 | -7.925373551931 |

cisVAPNB_TS-F1500

($E_F$ = -840.26931535 a.u.; $G_F$ = -839.93348166 a.u.)

0 1

| | | | |
|---|---|---|---|
| C | 2.127254066616 | 0.095500460106 | -5.307796729935 |
| C | 3.175415990593 | 0.569396475651 | -4.249453471528 |
| C | 1.642027575604 | -0.797203943257 | -3.072849638066 |

| | | | |
|---|---|---|---|
| C | 1.238769438266 | -0.942528688491 | -4.554655942193 |
| H | 1.513849899953 | 0.954659288847 | -5.601716446031 |
| H | 0.171648349122 | -0.771992567569 | -4.716176310368 |
| H | 1.448460095042 | -1.967785609996 | -4.880377624979 |
| C | 3.164671311130 | -0.563500805138 | -3.194806758106 |
| H | 3.625771621046 | -0.259135103787 | -2.252791244599 |
| H | 3.673936930087 | -1.463044580904 | -3.561483840515 |
| C | 2.803485797351 | 1.870403332485 | -3.595603490830 |
| H | 1.981585153613 | 2.421118031399 | -4.045911054705 |
| C | 0.984767333929 | 0.377979765243 | -2.398091896250 |
| H | 0.257022915610 | 0.924347111879 | -2.993976149581 |
| H | 4.186967009857 | 0.661039193062 | -4.670315693551 |
| H | 1.440380048564 | -1.712233609390 | -2.501199703369 |
| C | 3.930227154129 | 2.743663814718 | -3.065199253718 |
| H | 3.520894129476 | 3.500858874015 | -2.388585619833 |
| H | 4.617414821047 | 2.132997244558 | -2.467894106897 |
| C | 0.611073314696 | 0.236315499279 | -0.931566825966 |
| H | 1.467366452563 | -0.160312383678 | -0.373381102039 |
| H | 0.395304583001 | 1.223374803886 | -0.509798234219 |
| C | 4.787505912100 | 3.493002082120 | -4.166229796359 |
| H | 5.563440096515 | 4.091174005019 | -3.682212440539 |
| H | 5.268782189494 | 2.780680390044 | -4.840634211346 |
| H | 4.156932067807 | 4.156232504140 | -4.763898833311 |
| C | -0.632069209414 | -0.698290712181 | -0.632040358694 |
| H | -0.452020626388 | -1.715746451430 | -0.987407137988 |
| H | -0.812831011384 | -0.733553919509 | 0.445191966396 |

| | | | |
|---|---|---|---|
| H | -1.530573503730 | -0.317364966118 | -1.124464437647 |
| Si | 2.906102930417 | -0.554599214321 | -6.908599857180 |
| C | 4.120768608866 | -1.948600438940 | -6.516194921836 |
| H | 3.624667277317 | -2.780453941680 | -6.007553614898 |
| H | 4.571384696806 | -2.341130994232 | -7.432997364548 |
| H | 4.930878612280 | -1.597688657046 | -5.870173957573 |
| C | 3.824748075728 | 0.858070628652 | -7.762948793926 |
| H | 4.610421962400 | 1.266084952693 | -7.120105686790 |
| H | 4.294685749823 | 0.522464567295 | -8.692529047483 |
| H | 3.140599094014 | 1.675802508751 | -8.009925885029 |
| C | 1.543911707199 | -1.207108321109 | -8.042553920636 |
| H | 1.958582699736 | -1.593908199434 | -8.978555093748 |
| H | 0.987216291704 | -2.018131810582 | -7.563465829129 |
| H | 0.830648363376 | -0.416088572335 | -8.294199939023 |

cisVAPNB_R-F1500

($E_F$ = -840.31908329 a.u.; $G_F$ = -839.97699563 a.u.)

0 1

| | | | |
|---|---|---|---|
| C | 2.123545697035 | 0.068336797986 | -5.424372649159 |
| C | 3.102660163721 | 0.557574988288 | -4.312934496689 |
| C | 1.674792411973 | -0.776002552498 | -3.189071929403 |
| C | 1.198773798995 | -0.931893200528 | -4.646156118429 |
| H | 1.529836339699 | 0.922403537519 | -5.773355136432 |
| H | 0.137458751428 | -0.700057700491 | -4.766301749819 |
| H | 1.340443738999 | -1.967826000487 | -4.975244498306 |
| C | 3.195960315991 | -0.662476083015 | -3.378225430856 |

| | | | |
|---|---|---|---|
| H | 3.734738148440 | -0.451210775477 | -2.450889474596 |
| H | 3.638648161585 | -1.545257788687 | -3.844234119721 |
| C | 2.377297121313 | 1.590934140229 | -3.404604959297 |
| H | 1.809811159282 | 2.284305261610 | -4.035200153252 |
| C | 1.292068738193 | 0.638491122771 | -2.657492110267 |
| H | 0.313644009574 | 0.913842244911 | -3.067713811628 |
| H | 4.053964266753 | 0.925502832436 | -4.698403174507 |
| H | 1.340596896724 | -1.586569845580 | -2.540691633314 |
| C | 3.381948917309 | 2.468859775260 | -2.611254209783 |
| H | 2.825438041902 | 3.108033827867 | -1.917007915844 |
| H | 4.025255470482 | 1.836754856244 | -1.988719195871 |
| C | 1.120170436095 | 0.646119630341 | -1.116895469421 |
| H | 2.039433538933 | 0.284366683437 | -0.640943153019 |
| H | 0.987076201845 | 1.678133576258 | -0.774054100541 |
| C | 4.292947345575 | 3.386563274671 | -3.505063334203 |
| H | 4.900365783401 | 4.038698425082 | -2.872957522805 |
| H | 4.967807465871 | 2.799641764986 | -4.130955034563 |
| H | 3.683595005380 | 4.015767150049 | -4.160764606637 |
| C | -0.090633032816 | -0.207770551802 | -0.593163578724 |
| H | 0.032634523119 | -1.267643847536 | -0.824496158593 |
| H | -0.179408549665 | -0.106046060742 | 0.491101845234 |
| H | -1.026553552651 | 0.131612207329 | -1.046950175951 |
| Si | 2.935337436059 | -0.632224928227 | -6.987616545662 |
| C | 4.096765257336 | -2.068193792167 | -6.586031686959 |
| H | 3.574169728576 | -2.874802064677 | -6.063490383508 |
| H | 4.519264187436 | -2.486938718049 | -7.504867784558 |

| | | | |
|---|---|---|---|
| H | 4.928745831761 | -1.743151736717 | -5.954730479844 |
| C | 3.919653518440 | 0.740719229839 | -7.834523551488 |
| H | 4.718317956613 | 1.113057973977 | -7.185836021005 |
| H | 4.381795458142 | 0.384600162027 | -8.760430451142 |
| H | 3.274043599347 | 1.587616282093 | -8.087026212053 |
| C | 1.571501747811 | -1.243591944923 | -8.143504631579 |
| H | 1.988996507700 | -1.655084820155 | -9.067633454727 |
| H | 0.975500964217 | -2.028473190740 | -7.667938379070 |
| H | 0.893188628668 | -0.429038539052 | -8.415868865329 |

cisVAPNB_P-F1500

($E_F$ = -840.35298938 a.u.; $G_F$ = -840.02396995 a.u.)

0 1

| | | | |
|---|---|---|---|
| C | 2.134205767367 | 0.533579625143 | -5.047332309151 |
| C | 3.100518959037 | 1.190769815313 | -3.986654352592 |
| C | 1.479206788460 | -0.183705772658 | -2.727600693076 |
| C | 1.094403256340 | -0.309988544338 | -4.240600445374 |
| H | 1.601877728240 | 1.355551457493 | -5.540938603334 |
| H | 0.086243255977 | 0.085719892001 | -4.393531222509 |
| H | 1.061004229256 | -1.363485560635 | -4.533172134880 |
| C | 2.201312703779 | 1.194334055362 | -2.700409711144 |
| H | 1.462777125238 | 2.002372528736 | -2.773730634110 |
| H | 2.780655562866 | 1.351743168380 | -1.786964067834 |
| C | 3.691835017617 | 2.502582069106 | -4.450140880785 |
| H | 2.992934136997 | 3.312444916842 | -4.653341770929 |
| C | 0.319039856665 | -0.426006100182 | -1.787894214588 |

| | | | |
|---|---|---|---|
| H | -0.373133483655 | 0.395986573048 | -1.615016528602 |
| H | 3.928548797303 | 0.499110829419 | -3.781318519672 |
| H | 2.241885459570 | -0.950217414226 | -2.528104245880 |
| C | 5.172015397827 | 2.795945050370 | -4.563935028152 |
| H | 5.539565872468 | 3.147851288393 | -3.583692497641 |
| H | 5.706525683663 | 1.852213813087 | -4.732359109758 |
| C | -0.117356737451 | -1.835710736700 | -1.439628144381 |
| H | -0.338858421148 | -2.369821208096 | -2.380389628586 |
| H | 0.749109979639 | -2.373438463825 | -1.026524748801 |
| C | 5.652518245898 | 3.838717521036 | -5.631861565996 |
| H | 6.735289142718 | 3.965072858779 | -5.563541981466 |
| H | 5.402745056395 | 3.506893011416 | -6.642583339583 |
| H | 5.182294698877 | 4.812095794466 | -5.466628749147 |
| C | -1.334013678717 | -2.050639168644 | -0.481430983179 |
| H | -2.241479955179 | -1.598700102851 | -0.891595024113 |
| H | -1.514234789491 | -3.119193659615 | -0.345955927219 |
| H | -1.146971924846 | -1.605145144203 | 0.499425609922 |
| Si | 2.992783247232 | -0.460840761958 | -6.416443085320 |
| C | 3.937469174595 | -1.899007387078 | -5.633654821268 |
| H | 3.276586178114 | -2.526464973398 | -5.027925170969 |
| H | 4.388030758457 | -2.535092504912 | -6.401691847181 |
| H | 4.742531998401 | -1.539994726006 | -4.985727015508 |
| C | 4.188837778045 | 0.618520791848 | -7.400696160563 |
| H | 5.028157456734 | 0.951862334585 | -6.786378291806 |
| H | 4.595290021828 | 0.065065380161 | -8.253331706925 |
| H | 3.686321150468 | 1.510140038933 | -7.786977660259 |

| | | | |
|---|---|---|---|
| C | 1.673369886317 | -1.138944223790 | -7.587590941547 |
| H | 2.126035302866 | -1.731128233213 | -8.389023277562 |
| H | 0.961188819534 | -1.780001823118 | -7.059918930302 |
| H | 1.108298760745 | -0.325070428478 | -8.052774958387 |

cisVAPNB_TS-F1000

($E_F$ = -840.18052539 a.u.; $G_F$ = -839.84543400 a.u.)

0 1

| | | | |
|---|---|---|---|
| C | 2.125387198889 | 0.084186094899 | -5.304547986836 |
| C | 3.177131465646 | 0.565125114317 | -4.251731557433 |
| C | 1.629012776392 | -0.811223723891 | -3.069543293458 |
| C | 1.249799399527 | -0.967473891979 | -4.556464916297 |
| H | 1.501064700239 | 0.938738067175 | -5.588496658792 |
| H | 0.181663604406 | -0.819971128589 | -4.733312739894 |
| H | 1.485224805801 | -1.989333704027 | -4.875137330052 |
| C | 3.149561830775 | -0.545888600811 | -3.171172175384 |
| H | 3.589831783994 | -0.219070102374 | -2.226715038711 |
| H | 3.678432538192 | -1.445170567561 | -3.510811579172 |
| C | 2.836671547096 | 1.892851924470 | -3.644153081222 |
| H | 2.000911464034 | 2.437849276013 | -4.074008828143 |
| C | 0.928497335611 | 0.336057571703 | -2.399343488291 |
| H | 0.220779411085 | 0.900390397082 | -3.001508927106 |
| H | 4.192827444401 | 0.622505095809 | -4.671006906363 |
| H | 1.445461382973 | -1.734117060137 | -2.503055475610 |
| C | 3.950821548480 | 2.743725290667 | -3.080134887510 |
| H | 3.536460844238 | 3.494548877239 | -2.399019418837 |

| | | | |
|---|---|---|---|
| H | 4.626489349851 | 2.120305083613 | -2.482543961376 |
| C | 0.595037169388 | 0.230795220852 | -0.930250140818 |
| H | 1.460948203871 | -0.161546224334 | -0.383613326563 |
| H | 0.393604190674 | 1.226897635019 | -0.522238338948 |
| C | 4.807923279487 | 3.488973857585 | -4.165166974913 |
| H | 5.592183870379 | 4.080152847172 | -3.684780333166 |
| H | 5.281836729774 | 2.777420911045 | -4.846123084564 |
| H | 4.182418497822 | 4.160543041898 | -4.759183251138 |
| C | -0.639533979477 | -0.686204394848 | -0.610536577673 |
| H | -0.470106780625 | -1.706919126970 | -0.962835753389 |
| H | -0.814833250506 | -0.718798956377 | 0.468226474650 |
| H | -1.541464394792 | -0.304394828119 | -1.096409200696 |
| Si | 2.903453177305 | -0.549069518611 | -6.912116728333 |
| C | 4.127049835515 | -1.937378779590 | -6.527060217844 |
| H | 3.635122950649 | -2.776937968442 | -6.027096244626 |
| H | 4.584425278589 | -2.319283894129 | -7.444982996675 |
| H | 4.931860842936 | -1.585708984251 | -5.874806728341 |
| C | 3.812067361739 | 0.874779231427 | -7.758369921587 |
| H | 4.594462223698 | 1.284623980434 | -7.112710798699 |
| H | 4.284641621265 | 0.547968251693 | -8.689740483709 |
| H | 3.122138024675 | 1.689024879764 | -8.000687564235 |
| C | 1.544124090470 | -1.203976058541 | -8.048084093956 |
| H | 1.959895217658 | -1.582898285212 | -8.986813788063 |
| H | 0.993322368791 | -2.021196946178 | -7.572709734727 |
| H | 0.825469015036 | -0.416137862193 | -8.294332240063 |

cisVAPNB_R-F1000

($E_F$ = -840.24651443 a.u.; $G_F$ = -839.90384164 a.u.)

0 1

| | | | |
|---|---|---|---|
| C | 2.122599388008 | 0.066399514172 | -5.438677874831 |
| C | 3.096016358073 | 0.554768047081 | -4.321932506577 |
| C | 1.662662876233 | -0.779796466694 | -3.204269353501 |
| C | 1.190140524925 | -0.930029374724 | -4.663489548547 |
| H | 1.533113230922 | 0.921454506028 | -5.792450967203 |
| H | 0.130529396378 | -0.691728210036 | -4.786099505455 |
| H | 1.326497033785 | -1.966147768969 | -4.993978405628 |
| C | 3.185115505345 | -0.668718859592 | -3.390380546995 |
| H | 3.723766171053 | -0.463178499063 | -2.461892798151 |
| H | 3.626228433351 | -1.550726360621 | -3.859047467693 |
| C | 2.355986054101 | 1.580601653256 | -3.417250621300 |
| H | 1.799284203864 | 2.280300855178 | -4.050635709298 |
| C | 1.284970624995 | 0.635966047999 | -2.675121939749 |
| H | 0.295304798669 | 0.905268799082 | -3.061903262693 |
| H | 4.048468441506 | 0.927232834828 | -4.700530524874 |
| H | 1.323599153597 | -1.590421574937 | -2.558381604989 |
| C | 3.330290680306 | 2.441982682115 | -2.580778511141 |
| H | 2.753700915715 | 3.057142941426 | -1.881643827676 |
| H | 3.967551725266 | 1.800162251296 | -1.962163062433 |
| C | 1.170027194579 | 0.673587874702 | -1.135287639979 |
| H | 2.102898636051 | 0.314696128298 | -0.684655528877 |
| H | 1.054995273159 | 1.712218087666 | -0.806976532374 |
| C | 4.233195984650 | 3.381988193330 | -3.434583794367 |

| | | | |
|---|---|---|---|
| H | 4.837981623297 | 4.021191536152 | -2.786333917201 |
| H | 4.913428047639 | 2.818356282346 | -4.076557657573 |
| H | 3.624259965580 | 4.027385243187 | -4.075189807058 |
| C | -0.021684559723 | -0.155696069576 | -0.570648766444 |
| H | 0.081571616007 | -1.219308792094 | -0.796159492236 |
| H | -0.081198329979 | -0.047744054088 | 0.515374237568 |
| H | -0.968560006579 | 0.187951840022 | -0.998392112795 |
| Si | 2.939739302611 | -0.638334159569 | -6.997393802216 |
| C | 4.094868406453 | -2.078070543601 | -6.591141201399 |
| H | 3.567867105033 | -2.882847303349 | -6.070184193746 |
| H | 4.518955675880 | -2.498423883548 | -7.508518898617 |
| H | 4.925905571069 | -1.755626029614 | -5.957269623677 |
| C | 3.932054215029 | 0.731026507981 | -7.840718552126 |
| H | 4.729317881649 | 1.100846264467 | -7.188850451167 |
| H | 4.396825584213 | 0.372986402255 | -8.764564827728 |
| H | 3.290371023787 | 1.580017645908 | -8.096210668107 |
| C | 1.578696654107 | -1.245781118214 | -8.158678621718 |
| H | 1.998789651412 | -1.658978665596 | -9.080865551696 |
| H | 0.978206002723 | -2.028594695808 | -7.685344304828 |
| H | 0.904128089226 | -0.429188257488 | -8.434238491151 |

cisVAPNB_P-F1000

($E_F$ = -840.23314106 a.u.; $G_F$ = -839.90404154 a.u.)

0 1

| | | | |
|---|---|---|---|
| C | 2.123094541887 | 0.535061534111 | -5.078052810685 |
| C | 3.093619991789 | 1.177685783093 | -4.013288429455 |

| | | | |
|---|---|---|---|
| C | 1.486284412327 | -0.197176359525 | -2.766950719624 |
| C | 1.071063625500 | -0.291236296116 | -4.273008372072 |
| H | 1.605900192659 | 1.361987002489 | -5.579016332527 |
| H | 0.072649957420 | 0.137072346119 | -4.400691174916 |
| H | 1.001131666245 | -1.338434538006 | -4.581304777525 |
| C | 2.214025647712 | 1.169580378411 | -2.721603702532 |
| H | 1.479825101279 | 1.983354457981 | -2.772501328389 |
| H | 2.805229134998 | 1.307706138092 | -1.812667606190 |
| C | 3.668328188219 | 2.496533939305 | -4.446776818679 |
| H | 2.962879740244 | 3.300482192279 | -4.651035436379 |
| C | 0.348575342209 | -0.429630701508 | -1.812748845268 |
| H | -0.337739523750 | 0.394698717963 | -1.628349187932 |
| H | 3.926478554570 | 0.487061519180 | -3.824623098518 |
| H | 2.243444336193 | -0.975756426592 | -2.594641634518 |
| C | 5.138827446308 | 2.812263359441 | -4.513492326753 |
| H | 5.473872637721 | 3.194302685289 | -3.532731260036 |
| H | 5.699128126504 | 1.878977063232 | -4.652885680593 |
| C | -0.080695321180 | -1.829906520807 | -1.452950813532 |
| H | -0.321383762013 | -2.374206950174 | -2.383339563029 |
| H | 0.786275629522 | -2.369770713597 | -1.043331210636 |
| C | 5.590384458449 | 3.839966370042 | -5.591738437807 |
| H | 6.666567701314 | 4.015391303231 | -5.521042290448 |
| H | 5.363496153692 | 3.475611317237 | -6.596742019161 |
| H | 5.079732112859 | 4.797626058649 | -5.455493164608 |
| C | -1.278570195616 | -1.991366585935 | -0.480314650031 |
| H | -2.180311268093 | -1.526524862498 | -0.889312401212 |

| | | | |
|---|---|---|---|
| H | -1.489172539025 | -3.049391358034 | -0.308411859067 |
| H | -1.066792634608 | -1.522929652806 | 0.484955238925 |
| Si | 2.986848599184 | -0.472179191066 | -6.433782458964 |
| C | 3.876951393878 | -1.939338964101 | -5.640643390899 |
| H | 3.185736190925 | -2.559337769663 | -5.061725494696 |
| H | 4.335669022619 | -2.576193854235 | -6.403223342358 |
| H | 4.670758821736 | -1.605623378697 | -4.965863819429 |
| C | 4.234883354968 | 0.587840115117 | -7.373397912912 |
| H | 5.056892289401 | 0.905326293159 | -6.727575441483 |
| H | 4.662828057819 | 0.030024288059 | -8.212516036162 |
| H | 3.761791838710 | 1.488993702127 | -7.774370825317 |
| C | 1.680629934959 | -1.108932250801 | -7.641943662658 |
| H | 2.136681824229 | -1.710485412243 | -8.434396501226 |
| H | 0.938353323490 | -1.732241399375 | -7.134727367456 |
| H | 1.149657976202 | -0.278166039059 | -8.117236636749 |

cisVAPNB_TS-F500

($E_F$ = -840.09104935 a.u.; $G_F$ = -839.75739529 a.u.)

0 1

| | | | |
|---|---|---|---|
| C | 2.126836165269 | 0.044371964162 | -5.275017958634 |
| C | 3.186903342682 | 0.546505261096 | -4.237661051258 |
| C | 1.617115188524 | -0.845628642109 | -3.040309909752 |
| C | 1.299528822034 | -1.048312440071 | -4.535619216329 |
| H | 1.465708804172 | 0.882331514120 | -5.523101751783 |
| H | 0.230736997589 | -0.975314282900 | -4.751260762523 |
| H | 1.614580838963 | -2.056827801668 | -4.826986812799 |

| | | | |
|---|---|---|---|
| C | 3.128224323935 | -0.508725653358 | -3.097297960935 |
| H | 3.516573924815 | -0.122811067738 | -2.152463173120 |
| H | 3.702960154924 | -1.403992450601 | -3.367005279342 |
| C | 2.907422243850 | 1.924028178991 | -3.728393200748 |
| H | 2.049966956040 | 2.457543515789 | -4.126937448026 |
| C | 0.824846170580 | 0.256910522656 | -2.409522416081 |
| H | 0.158245760025 | 0.835575808255 | -3.043013919997 |
| H | 4.207364504487 | 0.536335366935 | -4.651714108134 |
| H | 1.469052732397 | -1.771188110572 | -2.466168354516 |
| C | 4.013988401335 | 2.749440550976 | -3.137268265312 |
| H | 3.597792852075 | 3.511208640386 | -2.468844128211 |
| H | 4.664221725996 | 2.115647602152 | -2.522091094965 |
| C | 0.519751896325 | 0.225151107996 | -0.940386446953 |
| H | 1.396189462830 | -0.135787597335 | -0.388360518814 |
| H | 0.316370216823 | 1.238339890466 | -0.576733863242 |
| C | 4.893226411167 | 3.461927746740 | -4.209067083764 |
| H | 5.687176122850 | 4.044007585182 | -3.732199254989 |
| H | 5.358857864510 | 2.733302833361 | -4.878197651962 |
| H | 4.288756847797 | 4.138981494748 | -4.818666628155 |
| C | -0.696476397987 | -0.680437110294 | -0.579799084823 |
| H | -0.521567007657 | -1.712075923385 | -0.897050715405 |
| H | -0.871182551931 | -0.679647876055 | 0.500168883247 |
| H | -1.604231741159 | -0.324763561952 | -1.074899518268 |
| Si | 2.891692570785 | -0.526981441945 | -6.911300975461 |
| C | 4.146963078522 | -1.900096246853 | -6.575934082835 |
| H | 3.674284159891 | -2.767269336667 | -6.105385259994 |

| | | | |
|---|---|---|---|
| H | 4.611848260104 | -2.239818561274 | -7.506556126260 |
| H | 4.944722083224 | -1.553914581153 | -5.912118823734 |
| C | 3.760634688294 | 0.938038088285 | -7.728172568996 |
| H | 4.539229697703 | 1.348138823578 | -7.078131006560 |
| H | 4.231753628055 | 0.646531801051 | -8.671933141783 |
| H | 3.050680731642 | 1.742895466811 | -7.942496273742 |
| C | 1.531476007024 | -1.180273484703 | -8.047114074445 |
| H | 1.942159679810 | -1.525090927782 | -9.001161934830 |
| H | 1.004885533457 | -2.021628731478 | -7.586374644577 |
| H | 0.792804824175 | -0.401865891136 | -8.262582689759 |

cisVAPNB_R-F500

($E_F$ = -840.17547689 a.u.; $G_F$ = -839.83225324 a.u.)

0 1

| | | | |
|---|---|---|---|
| C | 2.119628114703 | 0.062295223190 | -5.449458943348 |
| C | 3.088650278244 | 0.552781565862 | -4.329813993805 |
| C | 1.656609310158 | -0.787608836613 | -3.215331173155 |
| C | 1.185661341756 | -0.934805179944 | -4.675765703551 |
| H | 1.529936467462 | 0.916262136038 | -5.805566336140 |
| H | 0.126405652398 | -0.695245658977 | -4.799064729711 |
| H | 1.321279421745 | -1.970462493850 | -5.007781307780 |
| C | 3.179478746267 | -0.673514140324 | -3.401298880460 |
| H | 3.719039044167 | -0.471495452531 | -2.472786830352 |
| H | 3.621211130064 | -1.553319787095 | -3.873277210521 |
| C | 2.335193806924 | 1.570098073639 | -3.427339758720 |
| H | 1.785069554495 | 2.274520460045 | -4.061265535158 |

| | | | |
|---|---|---|---|
| C | 1.278294482741 | 0.627809378851 | -2.688314646395 |
| H | 0.278969587348 | 0.889140990299 | -3.055239395010 |
| H | 4.040379517275 | 0.931915847862 | -4.703995346811 |
| H | 1.317272602584 | -1.599343025309 | -2.570815409625 |
| C | 3.282530132734 | 2.417725074894 | -2.555046484467 |
| H | 2.689298703797 | 3.009110514425 | -1.850211701554 |
| H | 3.918663458292 | 1.768660693199 | -1.943268989309 |
| C | 1.211260219708 | 0.691641093602 | -1.150824879158 |
| H | 2.155828694950 | 0.338304114181 | -0.721269106539 |
| H | 1.106812804878 | 1.735040877254 | -0.836037855139 |
| C | 4.172240982415 | 3.381484522169 | -3.372307794210 |
| H | 4.775096446546 | 4.007890292401 | -2.709358540833 |
| H | 4.856645359172 | 2.843701876476 | -4.032506410040 |
| H | 3.559958800451 | 4.041935633209 | -3.994538368440 |
| C | 0.040018965271 | -0.117711959639 | -0.550444782834 |
| H | 0.127394637339 | -1.184524890049 | -0.769339928082 |
| H | 0.007027006090 | -0.003643050974 | 0.536427180551 |
| H | -0.916955983154 | 0.227664124394 | -0.954294272404 |
| Si | 2.941216833117 | -0.641814308126 | -7.006226840417 |
| C | 4.097120596929 | -2.080558673076 | -6.598778804823 |
| H | 3.570252940733 | -2.886110703701 | -6.078896767405 |
| H | 4.522748121680 | -2.500066764652 | -7.515833582300 |
| H | 4.927067196052 | -1.757577857610 | -5.963772359723 |
| C | 3.933635090072 | 0.728808580958 | -7.847265934232 |
| H | 4.729473173259 | 1.099028337196 | -7.193896260014 |
| H | 4.400267928224 | 0.371525540272 | -8.770466757056 |

| | | | |
|---|---|---|---|
| H | 3.291627427431 | 1.577313475230 | -8.103512807658 |
| C | 1.582959200073 | -1.250346540937 | -8.170186955336 |
| H | 2.005234367281 | -1.662955963159 | -9.091638111612 |
| H | 0.982327228411 | -2.033851304626 | -7.698188179419 |
| H | 0.908115215103 | -0.434377249773 | -8.446879184880 |

cisVAPNB_P-F500

($E_F$ = -840.11484851 a.u.; $G_F$ = -839.78553423 a.u.)

0 1

| | | | |
|---|---|---|---|
| C | 2.070645453494 | 0.555071846260 | -5.187452568239 |
| C | 3.062057274648 | 1.114017084849 | -4.097319337958 |
| C | 1.414016887495 | -0.267158510915 | -2.921611629158 |
| C | 0.968795175975 | -0.230158980142 | -4.413972123153 |
| H | 1.609076364641 | 1.417865127804 | -5.681599464904 |
| H | 0.007870956411 | 0.287953359396 | -4.486963824339 |
| H | 0.801353658264 | -1.245475555905 | -4.785128452705 |
| C | 2.203747553369 | 1.064213287065 | -2.797354209665 |
| H | 1.502547913995 | 1.907150644433 | -2.785701037370 |
| H | 2.810795106286 | 1.117829673427 | -1.889919594861 |
| C | 3.647452271893 | 2.443648480928 | -4.438124576685 |
| H | 2.949805712189 | 3.253561230998 | -4.646263360750 |
| C | 0.293237155079 | -0.469871425337 | -1.957946238555 |
| H | -0.488780021119 | 0.287474332513 | -1.935160328702 |
| H | 3.884341212218 | 0.400172702149 | -3.953868909800 |
| H | 2.136827671109 | -1.085257356820 | -2.804260052259 |
| C | 5.107846029520 | 2.774651212128 | -4.382396209977 |

| | | | |
|---|---|---|---|
| H | 5.365245331714 | 3.173887797468 | -3.385369323794 |
| H | 5.693464465101 | 1.851755511570 | -4.478020414130 |
| C | 0.233392588267 | -1.580467306776 | -0.952952662697 |
| H | 0.799821767064 | -2.441011574199 | -1.331886421034 |
| H | 0.759441923275 | -1.279418775864 | -0.029234278551 |
| C | 5.585244068008 | 3.797518529602 | -5.440002624098 |
| H | 6.649183853168 | 4.015997137604 | -5.316298466016 |
| H | 5.427213249803 | 3.413001001833 | -6.450802398763 |
| H | 5.034240063254 | 4.738137639634 | -5.347693328993 |
| C | -1.190990504767 | -2.036277559007 | -0.562785487409 |
| H | -1.731487593395 | -2.414950232242 | -1.434652704445 |
| H | -1.153320058099 | -2.828637358761 | 0.189258874908 |
| H | -1.766768920029 | -1.203776208212 | -0.147480671282 |
| Si | 2.923723421244 | -0.462670579787 | -6.540889567477 |
| C | 3.651037312729 | -2.028382426927 | -5.769830173611 |
| H | 2.878755426170 | -2.621743249831 | -5.271158909686 |
| H | 4.114428248974 | -2.660357528241 | -6.533637110736 |
| H | 4.418355886294 | -1.788330989556 | -5.027962640716 |
| C | 4.305573370376 | 0.541554127295 | -7.344931808393 |
| H | 5.084664529935 | 0.794033285330 | -6.621042987574 |
| H | 4.772676549003 | -0.018895164026 | -8.160953719305 |
| H | 3.921007986666 | 1.479318053960 | -7.756483515729 |
| C | 1.648265093755 | -0.943047267460 | -7.849161867229 |
| H | 2.100138905301 | -1.553689428575 | -8.637021966554 |
| H | 0.827527351910 | -1.518263223874 | -7.410272240947 |
| H | 1.217753574880 | -0.054206205762 | -8.320868327890 |

transVAPNB_TS-F3500

($E_F$ = -840.63341093 a.u.; $G_F$ = -840.29655712 a.u.)

0 1

| | | | |
|---|---|---|---|
| C | 2.053015792263 | -0.041472096172 | -5.567086882517 |
| C | 2.950640173645 | 0.658190352006 | -4.499214022221 |
| C | 1.561370717772 | -0.755337234920 | -3.251805609431 |
| C | 1.149742231124 | -0.998303071642 | -4.719555178207 |
| H | 1.428330537198 | 0.703855825476 | -6.067222715324 |
| H | 0.084870534462 | -0.810819971703 | -4.880116572864 |
| H | 1.322619022230 | -2.050444773676 | -4.971777119042 |
| C | 3.059973294910 | -0.397511079963 | -3.391117932074 |
| H | 3.493167492964 | 0.000819011356 | -2.469822004081 |
| H | 3.649937599951 | -1.263630292946 | -3.703058901360 |
| C | 2.297275951626 | 1.893736257887 | -3.922278312464 |
| H | 2.856474986872 | 2.296753760246 | -3.077700526020 |
| C | 0.854415427198 | 0.415848014919 | -2.615595062071 |
| H | -0.098368615284 | 0.678452796263 | -3.070593630183 |
| H | 3.942715586701 | 0.929411534978 | -4.890160857231 |
| H | 1.421526423171 | -1.656802332834 | -2.642930102495 |
| C | 0.792184535775 | 0.372004622259 | -1.049842182439 |
| H | 1.817653471802 | 0.331755717297 | -0.666130881195 |
| H | 0.372636360467 | 1.317478640791 | -0.693762325879 |
| C | 1.871842431927 | 3.021924247410 | -4.927604075242 |
| H | 1.038834682517 | 2.659855213578 | -5.538357177635 |
| H | 1.467252898463 | 3.849830929822 | -4.338210251305 |

| | | | |
|---|---|---|---|
| C | 2.921324326614 | 3.710381555445 | -6.004152299139 |
| H | 3.764604170127 | 4.172807018581 | -5.487178350583 |
| H | 3.296998115429 | 2.968583073770 | -6.709590183814 |
| H | 2.362328215447 | 4.476116612518 | -6.543726362348 |
| C | -0.019375979295 | -0.815845805913 | -0.232258655695 |
| H | -1.078587038793 | -0.817984689637 | -0.496815988080 |
| H | 0.405148682012 | -1.798299621523 | -0.445340516329 |
| H | 0.089262145670 | -0.597997528377 | 0.831070332750 |
| Si | 2.984379793832 | -0.905824656252 | -6.976137440011 |
| C | 4.201053104563 | -2.194425294149 | -6.318086257277 |
| H | 3.700793273356 | -2.936761849987 | -5.689075953251 |
| H | 4.677052188178 | -2.728289676738 | -7.146691547971 |
| H | 4.992740240836 | -1.729320717380 | -5.723566349972 |
| C | 3.933650452784 | 0.375052099425 | -7.990757283464 |
| H | 4.669413926525 | 0.905293754527 | -7.378800188336 |
| H | 4.470563915169 | -0.100762725180 | -8.817299490515 |
| H | 3.255043344351 | 1.119146190736 | -8.418996586971 |
| C | 1.715357569576 | -1.762625578789 | -8.083765717620 |
| H | 2.202067063467 | -2.271346327385 | -8.921604519561 |
| H | 1.143012681563 | -2.510488354752 | -7.526672680351 |
| H | 1.005020075760 | -1.040092428819 | -8.497887997452 |

transVAPNB_P-F3500

($E_F$ = -840.84621719 a.u.; $G_F$ = -840.51788396 a.u.)

0 1

| | | | |
|---|---|---|---|
| C | 1.711592843439 | 0.469420580825 | -5.395451558639 |

| | | | |
|---|---|---|---|
| C | 2.667760176335 | 1.518375206410 | -4.658017449290 |
| C | 1.657048723469 | -0.083817067979 | -2.944295467545 |
| C | 0.873426534672 | -0.251402290004 | -4.279793130755 |
| H | 1.028343621210 | 1.021703806053 | -6.049588628856 |
| H | -0.104396862601 | 0.229160818750 | -4.168624520546 |
| H | 0.677740476368 | -1.305168832866 | -4.499089541226 |
| C | 2.151631049493 | 1.414067442578 | -3.149610607236 |
| H | 1.300736711700 | 2.090985015803 | -3.007608772158 |
| H | 2.928054574764 | 1.704016282981 | -2.437846333353 |
| C | 2.759064362546 | 2.916784654144 | -5.343593172742 |
| H | 1.937604595877 | 3.601681132905 | -5.137875623733 |
| C | 0.866823039806 | -0.534486081334 | -1.681770565542 |
| H | 0.130224805006 | 0.162556358954 | -1.286799542995 |
| H | 3.686106575875 | 1.106964273797 | -4.659056088461 |
| H | 2.549126151233 | -0.720227405108 | -3.007487215135 |
| C | 0.822154444258 | -2.015350091732 | -1.200979043323 |
| H | 0.456377716549 | -2.612907864629 | -2.052287128700 |
| H | 1.864378670171 | -2.343413921193 | -1.080482293355 |
| C | 3.674706641412 | 3.222808201360 | -6.565829700636 |
| H | 4.666751184760 | 2.811986095556 | -6.335704546601 |
| H | 3.311903060178 | 2.589545683984 | -7.388363938906 |
| C | 3.935075639949 | 4.674765918282 | -7.261821253251 |
| H | 3.002613454530 | 5.110042111260 | -7.627918808071 |
| H | 4.402203489449 | 5.375400508900 | -6.566448535079 |
| H | 4.605262863219 | 4.506932897818 | -8.105934449083 |
| C | 0.017611103505 | -2.585589189891 | 0.097084279954 |

| | | | |
|---|---|---|---|
| H | 0.380326084006 | -2.128874073623 | 1.020347073365 |
| H | -1.055215874213 | -2.400735519166 | 0.009440896148 |
| H | 0.191916938311 | -3.661577333599 | 0.137653240763 |
| Si | 2.590522485337 | -0.836657948485 | -6.470195913113 |
| C | 3.581376288526 | -1.998853631819 | -5.354333881378 |
| H | 2.939804996639 | -2.515174227598 | -4.634954195185 |
| H | 4.089157599001 | -2.760236007381 | -5.954607598077 |
| H | 4.346862262295 | -1.456424496081 | -4.791386262994 |
| C | 3.774204274641 | -0.109669985801 | -7.748726683692 |
| H | 4.579424810472 | 0.465525802774 | -7.286079368740 |
| H | 4.231562029512 | -0.925083511444 | -8.318754868121 |
| H | 3.257737150410 | 0.541418524398 | -8.459408823849 |
| C | 1.273333872790 | -1.830076259812 | -7.394405572968 |
| H | 1.735358097349 | -2.608203347042 | -8.010087554218 |
| H | 0.574473855362 | -2.318711133279 | -6.709806954718 |
| H | 0.691796613501 | -1.182462395690 | -8.058156363725 |

transVAPNB_R-F3500

($E_F$ = -840.64935795 a.u.; $G_F$ = -840.30872135 a.u.)

0 1

| | | | |
|---|---|---|---|
| C | 2.092921271957 | -0.042936922749 | -5.596376780670 |
| C | 2.939759420596 | 0.561966956119 | -4.435541747293 |
| C | 1.456735120752 | -0.747151980665 | -3.342354303599 |
| C | 1.077037054525 | -0.953546832980 | -4.823525917851 |
| H | 1.549150112976 | 0.750573401220 | -6.116243120124 |
| H | 0.037657069487 | -0.678649108062 | -5.023006849123 |

| | | | |
|---|---|---|---|
| H | 1.182400128752 | -2.011735875833 | -5.088130575956 |
| C | 2.988529562546 | -0.601011390918 | -3.430981160547 |
| H | 3.456178208100 | -0.326551032933 | -2.482041891986 |
| H | 3.482108689512 | -1.495380419853 | -3.815282552756 |
| C | 2.106549951488 | 1.634537816267 | -3.677517377457 |
| H | 2.756500627213 | 2.096206275115 | -2.924717787224 |
| C | 1.016825982968 | 0.649606184391 | -2.839244127594 |
| H | 0.001361572771 | 0.876763563726 | -3.181279587014 |
| H | 3.910445328618 | 0.953389417300 | -4.749328509105 |
| H | 1.097528892440 | -1.555587347817 | -2.704430815873 |
| C | 1.015619403658 | 0.675240233810 | -1.235737470259 |
| H | 2.060004032179 | 0.681253103863 | -0.905437288886 |
| H | 0.605347464193 | 1.641977766073 | -0.924862185049 |
| C | 1.597447010559 | 2.799680302432 | -4.647974683891 |
| H | 0.709512882135 | 2.435510485631 | -5.174937775228 |
| H | 1.239788427825 | 3.617844279077 | -4.014298198625 |
| C | 2.550729698241 | 3.483718293283 | -5.788885949549 |
| H | 3.431015759031 | 3.949444791830 | -5.339739106432 |
| H | 2.881206964165 | 2.747367331361 | -6.522285980097 |
| H | 1.959054637162 | 4.250966611484 | -6.290336905768 |
| C | 0.269487927933 | -0.460838324351 | -0.321534196843 |
| H | -0.789109049342 | -0.546585976692 | -0.578209143598 |
| H | 0.741909301813 | -1.437428567420 | -0.438067082908 |
| H | 0.358668397818 | -0.146624044825 | 0.719523989479 |
| Si | 3.055091841138 | -0.905634467666 | -6.985471881808 |
| C | 4.148914080415 | -2.311811409171 | -6.353707695706 |

| | | | |
|---|---|---|---|
| H | 3.570971910466 | -3.054314845107 | -5.795653956004 |
| H | 4.625142201047 | -2.826086057038 | -7.194588902622 |
| H | 4.942220727587 | -1.940809708486 | -5.698544992411 |
| C | 4.141317114761 | 0.363973550166 | -7.869117978726 |
| H | 4.844420203341 | 0.837098405128 | -7.177101284066 |
| H | 4.724805173541 | -0.106045511437 | -8.666883408509 |
| H | 3.535650276913 | 1.154702350844 | -8.322180528339 |
| C | 1.796059643386 | -1.611807188738 | -8.205267526709 |
| H | 2.292509691108 | -2.106620883041 | -9.045701151190 |
| H | 1.146907214120 | -2.347729114694 | -7.721184922210 |
| H | 1.157784253469 | -0.821050649917 | -8.611679509717 |

transVAPNB_TS-F3000

($E_F$ = -840.54255008 a.u.; $G_F$ = -840.20598992 a.u.)

0 1

| | | | |
|---|---|---|---|
| C | 2.048432281517 | -0.042904171302 | -5.562830111072 |
| C | 2.952401080177 | 0.665395650934 | -4.505369445285 |
| C | 1.571943869340 | -0.759138607317 | -3.242267169369 |
| C | 1.158630138235 | -1.006530942934 | -4.708891398881 |
| H | 1.413940884498 | 0.697165293882 | -6.058371725624 |
| H | 0.091343275523 | -0.830463532893 | -4.866135561835 |
| H | 1.341125888258 | -2.057159268004 | -4.960463355050 |
| C | 3.065274941649 | -0.379136310375 | -3.386397505373 |
| H | 3.493707044407 | 0.030960545720 | -2.468092835536 |
| H | 3.665456576848 | -1.241224953519 | -3.690853472408 |
| C | 2.318288653882 | 1.915446663029 | -3.945939862350 |

| | | | |
|---|---|---|---|
| H | 2.862681863670 | 2.313688817753 | -3.089879815166 |
| C | 0.840555917784 | 0.388590706090 | -2.597126876341 |
| H | -0.103951010914 | 0.661282792864 | -3.062507140352 |
| H | 3.946783051498 | 0.920273634544 | -4.903589120329 |
| H | 1.454616394620 | -1.667016415235 | -2.637081186390 |
| C | 0.787961981415 | 0.365572688235 | -1.045013618630 |
| H | 1.813863692392 | 0.321338401704 | -0.662000972425 |
| H | 0.373657669347 | 1.315605444618 | -0.694228465260 |
| C | 1.877949623113 | 3.028632371918 | -4.938446563180 |
| H | 1.053182396249 | 2.657536923310 | -5.555603769461 |
| H | 1.460645916574 | 3.851799285283 | -4.350864398418 |
| C | 2.934916228340 | 3.703628820935 | -5.983757311805 |
| H | 3.770805153986 | 4.164329382684 | -5.452578647389 |
| H | 3.324804531996 | 2.958930958219 | -6.678918592247 |
| H | 2.398260629710 | 4.471802591654 | -6.543085036447 |
| C | -0.028108095755 | -0.809030713880 | -0.255496609927 |
| H | -1.085231048488 | -0.801926613878 | -0.529764860664 |
| H | 0.388253892217 | -1.793535958394 | -0.476987367660 |
| H | 0.066919064391 | -0.613453739756 | 0.813923289792 |
| Si | 2.975415152895 | -0.904465052409 | -6.976497577306 |
| C | 4.201600567546 | -2.184134459385 | -6.319221736012 |
| H | 3.707846142849 | -2.927118350255 | -5.686002714872 |
| H | 4.678144734411 | -2.717957146174 | -7.147470077023 |
| H | 4.992232942878 | -1.712278020534 | -5.728748140687 |
| C | 3.913400650113 | 0.377975368999 | -7.999207815677 |
| H | 4.656663427453 | 0.906253683351 | -7.394778396549 |

| | | | |
|---|---|---|---|
| H | 4.440414013464 | -0.096763478510 | -8.832697838443 |
| H | 3.230713911853 | 1.123558127558 | -8.418048252862 |
| C | 1.705271427432 | -1.770661929983 | -8.075173215911 |
| H | 2.190209853158 | -2.278811869262 | -8.914375133414 |
| H | 1.139998653071 | -2.520126841643 | -7.513235133755 |
| H | 0.988937841330 | -1.053018657094 | -8.487200819681 |

transVAPNB_P-F3000

($E_F$ = -840.71602844 a.u.; $G_F$ = -840.38820955 a.u.)

0 1

| | | | |
|---|---|---|---|
| C | 1.794497352371 | 0.434743041601 | -5.404554503083 |
| C | 2.711670252268 | 1.429132349540 | -4.603479701903 |
| C | 1.612860544874 | -0.119429269172 | -2.954042319552 |
| C | 0.918129104560 | -0.321761331279 | -4.337294069967 |
| H | 1.131975093311 | 1.006705074540 | -6.062723222231 |
| H | -0.081906753288 | 0.121441699205 | -4.289282885901 |
| H | 0.776956997407 | -1.382760684652 | -4.563370179154 |
| C | 2.092079093851 | 1.374307924400 | -3.140073222142 |
| H | 1.225799695707 | 2.043150347662 | -3.078145421341 |
| H | 2.812429576918 | 1.686901911196 | -2.379788839332 |
| C | 3.037925301998 | 2.812858493713 | -5.227169026991 |
| H | 3.461201622117 | 3.526138700413 | -4.521785028406 |
| C | 0.760116905390 | -0.540788352273 | -1.738242542243 |
| H | -0.046628889099 | 0.127738592941 | -1.443628066787 |
| H | 3.698346845220 | 0.946759488702 | -4.481648826609 |
| H | 2.516163926217 | -0.743847821778 | -2.949579504030 |

| | | | |
|---|---|---|---|
| C | 0.778928167960 | -1.976410331466 | -1.166608353152 |
| H | 0.518807170854 | -2.658060185955 | -1.993866154659 |
| H | 1.826790080073 | -2.224521556070 | -0.943645199340 |
| C | 2.871358043007 | 3.308270180109 | -6.677376455375 |
| H | 2.810431777162 | 2.442992869209 | -7.341877377289 |
| H | 1.875343989445 | 3.777334358512 | -6.746520317997 |
| C | 3.877055625661 | 4.366082250200 | -7.372446554798 |
| H | 3.929165060266 | 5.293904547079 | -6.797920169309 |
| H | 4.884025845704 | 3.952136928102 | -7.461825509522 |
| H | 3.492715099544 | 4.588413747444 | -8.369286087744 |
| C | -0.090548221448 | -2.467303801847 | 0.096339671187 |
| H | 0.170450558799 | -1.908789776463 | 0.998256073065 |
| H | -1.160503839074 | -2.348332440202 | -0.090263965844 |
| H | 0.123151476921 | -3.524586020118 | 0.260673193144 |
| Si | 2.759170117768 | -0.810963618212 | -6.474291633960 |
| C | 3.668499537475 | -2.018630242603 | -5.336763091038 |
| H | 2.978207684166 | -2.556877326784 | -4.681379580321 |
| H | 4.213328849388 | -2.760586333510 | -5.928757006577 |
| H | 4.396130770328 | -1.501907106347 | -4.703988011492 |
| C | 4.043385241758 | 0.030244216298 | -7.576448324572 |
| H | 4.670799070446 | 0.721671100861 | -7.006745239409 |
| H | 4.697914354518 | -0.722401304376 | -8.027473791030 |
| H | 3.577575140171 | 0.592351402389 | -8.390507933345 |
| C | 1.542212657749 | -1.768680925369 | -7.556691221757 |
| H | 2.060395370139 | -2.515931224174 | -8.165696557318 |
| H | 0.794991518738 | -2.291347565445 | -6.952258611085 |

| | | | |
|---|---|---|---|
| H | 1.010004738182 | -1.095332616751 | -8.235909511352 |

transVAPNB_R-F3000

($E_F$ = -840.56819503 a.u.; $G_F$ = -840.22676368 a.u.)

0 1

| | | | |
|---|---|---|---|
| C | 2.090768144744 | -0.051975396784 | -5.595002110826 |
| C | 2.941304922554 | 0.557019042004 | -4.439010948317 |
| C | 1.467728006620 | -0.753071586001 | -3.337338464513 |
| C | 1.084325582912 | -0.968346347299 | -4.816247188305 |
| H | 1.539007262349 | 0.738615686251 | -6.111168815126 |
| H | 0.042222827654 | -0.702392410044 | -5.013601906193 |
| H | 1.197667681316 | -2.026514618322 | -5.077591253949 |
| C | 2.998740354620 | -0.604829394813 | -3.432981354245 |
| H | 3.470805951481 | -0.327741842850 | -2.486976497026 |
| H | 3.492193196660 | -1.498765650809 | -3.818459021443 |
| C | 2.104218868181 | 1.621538612080 | -3.671730452820 |
| H | 2.758419456318 | 2.083983752233 | -2.922460761881 |
| C | 1.028757938987 | 0.650879607627 | -2.848427417968 |
| H | 0.012023738765 | 0.871186957054 | -3.192585944278 |
| H | 3.908557023957 | 0.952937694786 | -4.756965556995 |
| H | 1.110456212326 | -1.557168207373 | -2.693233884518 |
| C | 1.032657584377 | 0.710049798193 | -1.258625147992 |
| H | 2.076804713875 | 0.707551140150 | -0.926659729115 |
| H | 0.635228139272 | 1.687771040259 | -0.964636719263 |
| C | 1.574454570217 | 2.778843246897 | -4.618662690884 |
| H | 0.694077915170 | 2.406765342060 | -5.153289091504 |

| | | | |
|---|---|---|---|
| H | 1.201847188779 | 3.585249328883 | -3.978045077786 |
| C | 2.532801317272 | 3.461547279800 | -5.726429438804 |
| H | 3.409817430901 | 3.919312534852 | -5.261748752900 |
| H | 2.873854386580 | 2.730683394699 | -6.460974753833 |
| H | 1.960232349346 | 4.237885453508 | -6.236844823785 |
| C | 0.270795928181 | -0.399535081176 | -0.361763171531 |
| H | -0.784111314332 | -0.478966687244 | -0.637113750411 |
| H | 0.733688855387 | -1.382427327418 | -0.464882414380 |
| H | 0.338635459399 | -0.089730950708 | 0.682636985370 |
| Si | 3.050785408899 | -0.906700710169 | -6.990181637668 |
| C | 4.158419629351 | -2.304926600544 | -6.365013566090 |
| H | 3.588558042147 | -3.053207792975 | -5.806351772977 |
| H | 4.636124436022 | -2.813772697506 | -7.208349124178 |
| H | 4.950739662760 | -1.928472345358 | -5.711762902534 |
| C | 4.122140777421 | 0.373075483862 | -7.877236090918 |
| H | 4.830702271182 | 0.844450636859 | -7.189601551219 |
| H | 4.698923310530 | -0.088799305012 | -8.684562132852 |
| H | 3.508075492812 | 1.164379633819 | -8.317874512348 |
| C | 1.790367922869 | -1.620870964779 | -8.203802803665 |
| H | 2.285655803957 | -2.112802397714 | -9.046602422747 |
| H | 1.148149050759 | -2.360670300383 | -7.716365819311 |
| H | 1.145230478359 | -0.834136874679 | -8.607172733218 |

transVAPNB_TS-F2500

($E_F$ = -840.45223840 a.u.; $G_F$ = -840.11555874 a.u.)

0 1

| | | | |
|---|---|---|---|
| C | 2.045011231266 | -0.046424852074 | -5.558707295335 |
| C | 2.955284152148 | 0.666903128866 | -4.509088863525 |
| C | 1.579509792995 | -0.765805531194 | -3.234961570217 |
| C | 1.167161161427 | -1.017647618736 | -4.701115600991 |
| H | 1.401927329506 | 0.690229227105 | -6.048161281123 |
| H | 0.097878847623 | -0.853528276883 | -4.857287939120 |
| H | 1.360618699984 | -2.066416901678 | -4.952016422385 |
| C | 3.068543357811 | -0.369529167598 | -3.381680999307 |
| H | 3.493210019667 | 0.048697318838 | -2.465351715884 |
| H | 3.676081440462 | -1.228779537551 | -3.680291262545 |
| C | 2.337565693928 | 1.928641231021 | -3.962319056327 |
| H | 2.866200032684 | 2.320499017410 | -3.093766610792 |
| C | 0.828854823042 | 0.365039770888 | -2.585536873122 |
| H | -0.107061743082 | 0.649035556158 | -3.061020589447 |
| H | 3.951446009619 | 0.907449173216 | -4.913221553730 |
| H | 1.477585214918 | -1.677570061527 | -2.632112065145 |
| C | 0.785604572457 | 0.366961941826 | -1.045275627972 |
| H | 1.811842711141 | 0.318760156236 | -0.662911851490 |
| H | 0.377647941843 | 1.323130095710 | -0.703089347096 |
| C | 1.882711512553 | 3.030575121829 | -4.941059958999 |
| H | 1.063658920133 | 2.653335465646 | -5.562602894584 |
| H | 1.456642523265 | 3.849178397982 | -4.353075149311 |
| C | 2.946038746888 | 3.692403048867 | -5.958846576477 |
| H | 3.776825703330 | 4.148764529683 | -5.415381094208 |
| H | 3.345818205924 | 2.944905362427 | -6.645702727083 |
| H | 2.431580264668 | 4.464691426164 | -6.533989413056 |

| | | | |
|---|---|---|---|
| C | -0.036622691993 | -0.791821550868 | -0.277662161001 |
| H | -1.091334185249 | -0.775878139690 | -0.562008882557 |
| H | 0.371459523331 | -1.779202804953 | -0.503164933322 |
| H | 0.044484115442 | -0.613157288431 | 0.796316366034 |
| Si | 2.967794300020 | -0.902392678916 | -6.978496539682 |
| C | 4.207261582572 | -2.170770938478 | -6.324253646975 |
| H | 3.722048627722 | -2.917683702677 | -5.689053171501 |
| H | 4.686791690359 | -2.700869117318 | -7.153159749741 |
| H | 4.994855521102 | -1.690928269381 | -5.736104302932 |
| C | 3.890750692526 | 0.384433043799 | -8.009314105659 |
| H | 4.640287123270 | 0.912610910934 | -7.412644555881 |
| H | 4.408766605234 | -0.087636555504 | -8.849909560789 |
| H | 3.201959043805 | 1.129786474741 | -8.418419389147 |
| C | 1.697500029937 | -1.780000288575 | -8.067821178397 |
| H | 2.181182687063 | -2.286139652382 | -8.908957999608 |
| H | 1.141120501803 | -2.532530165917 | -7.501115958084 |
| H | 0.973469471789 | -1.068406178469 | -8.476932248761 |

transVAPNB_P-F2500

($E_F$ = -840.59186379 a.u.; $G_F$ = -840.26331745 a.u.)

0 1

| | | | |
|---|---|---|---|
| C | 1.802103672030 | 0.415912876036 | -5.404269960251 |
| C | 2.715511831536 | 1.401045790574 | -4.592009538302 |
| C | 1.612036445288 | -0.128257581664 | -2.958393519836 |
| C | 0.929174698257 | -0.349896903441 | -4.344241910703 |
| H | 1.137741248636 | 0.994220816197 | -6.054874606004 |

| | | | |
|---|---|---|---|
| H | -0.078384165213 | 0.076778519558 | -4.306261509176 |
| H | 0.807847051797 | -1.414615360736 | -4.564111037005 |
| C | 2.081749278319 | 1.360025725070 | -3.141813861122 |
| H | 1.212037080835 | 2.025893123243 | -3.095783792377 |
| H | 2.791327771868 | 1.680378898262 | -2.374586530358 |
| C | 3.050610357586 | 2.772451575577 | -5.207394115774 |
| H | 3.536311935599 | 3.463254476168 | -4.519942924852 |
| C | 0.756133598658 | -0.533320688335 | -1.753737053717 |
| H | -0.059143980757 | 0.130571414703 | -1.472549603510 |
| H | 3.697173482399 | 0.911721324131 | -4.456032441473 |
| H | 2.518178139949 | -0.749064976586 | -2.940992104630 |
| C | 0.781794838677 | -1.956913885353 | -1.181210923497 |
| H | 0.524826740521 | -2.648411426883 | -2.001933965641 |
| H | 1.828690979603 | -2.202694976476 | -0.949959589123 |
| C | 2.810917650263 | 3.290250223900 | -6.627708449925 |
| H | 2.663990400154 | 2.441429199564 | -7.300487262225 |
| H | 1.840999986771 | 3.816616899894 | -6.631923731515 |
| C | 3.842368046582 | 4.289381676494 | -7.331518127459 |
| H | 3.983451220067 | 5.192771010806 | -6.732799548510 |
| H | 4.815069413233 | 3.812722687101 | -7.475012633773 |
| H | 3.440698570458 | 4.574082686294 | -8.305828757523 |
| C | -0.095300555229 | -2.400579063703 | 0.071676132813 |
| H | 0.162929576939 | -1.820307125682 | 0.960993345230 |
| H | -1.162148069206 | -2.270805926457 | -0.127340803307 |
| H | 0.095969665072 | -3.455869845388 | 0.274137330289 |
| Si | 2.777320773331 | -0.806102498329 | -6.490075465142 |

| | | | |
|---|---|---|---|
| C | 3.695597589143 | -2.019929352616 | -5.366527544770 |
| H | 3.008761126221 | -2.567921077559 | -4.715476334593 |
| H | 4.243903954736 | -2.753521301671 | -5.965667195291 |
| H | 4.420805954979 | -1.505212707946 | -4.729391193173 |
| C | 4.052456672891 | 0.073337321344 | -7.572768966594 |
| H | 4.663272291889 | 0.765851652076 | -6.986462247380 |
| H | 4.722980453201 | -0.658318867396 | -8.034524068201 |
| H | 3.580462425467 | 0.643265720010 | -8.377786246907 |
| C | 1.571707976232 | -1.759961147558 | -7.588100006275 |
| H | 2.097346023374 | -2.494056136422 | -8.206614054767 |
| H | 0.828710377043 | -2.297591874744 | -6.991528919112 |
| H | 1.033999465945 | -1.082268545652 | -8.258598376582 |

transVAPNB_R-F2500

($E_F$ = -840.48847611 a.u.; $G_F$ = -840.14641945 a.u.)

0 1

| | | | |
|---|---|---|---|
| C | 2.088753741356 | -0.062912058034 | -5.593379253139 |
| C | 2.943868310928 | 0.551389725404 | -4.443678570688 |
| C | 1.482326949861 | -0.761332144894 | -3.331122490885 |
| C | 1.094320947961 | -0.986581223388 | -4.807382635367 |
| H | 1.527401129339 | 0.724354499350 | -6.104647761303 |
| H | 0.048896109391 | -0.731689886723 | -5.001657152336 |
| H | 1.217490995746 | -2.044469790277 | -5.065360309975 |
| C | 3.012174430507 | -0.608356541884 | -3.435309830564 |
| H | 3.488949680844 | -0.327112934866 | -2.492864896331 |
| H | 3.506577564860 | -1.501439449602 | -3.821618028222 |

| | | | |
|---|---|---|---|
| C | 2.103823303238 | 1.607998651887 | -3.667546197361 |
| H | 2.761965317612 | 2.070833210116 | -2.921427153279 |
| C | 1.041229978640 | 0.648394126329 | -2.856555652516 |
| H | 0.023447056648 | 0.860694470746 | -3.203816141041 |
| H | 3.907163988431 | 0.951916242739 | -4.767201908553 |
| H | 1.129491058409 | -1.561673295072 | -2.680177829028 |
| C | 1.047775844822 | 0.740946761390 | -1.280033280991 |
| H | 2.090660139240 | 0.723731354824 | -0.943836841361 |
| H | 0.668073358770 | 1.731302204918 | -1.004327670270 |
| C | 1.554155405185 | 2.759288299057 | -4.590616008502 |
| H | 0.682200187729 | 2.380865654666 | -5.134920571039 |
| H | 1.166580434940 | 3.552276770298 | -3.941760179776 |
| C | 2.518597636588 | 3.444804823309 | -5.664885596727 |
| H | 3.392796591177 | 3.891146987662 | -5.183064639066 |
| H | 2.868286898192 | 2.721733022575 | -6.403551863820 |
| H | 1.965311098858 | 4.232860473102 | -6.179396715762 |
| C | 0.261672568097 | -0.338406362488 | -0.399847540794 |
| H | -0.789635006789 | -0.402889940009 | -0.693986847841 |
| H | 0.707238208472 | -1.330689604843 | -0.490657477634 |
| H | 0.311017622999 | -0.033011468880 | 0.647277180472 |
| Si | 3.045906831310 | -0.908140131570 | -6.995902646748 |
| C | 4.169758128011 | -2.296812876091 | -6.378590214424 |
| H | 3.609594478223 | -3.051847752933 | -5.819193321818 |
| H | 4.649110362683 | -2.799200891341 | -7.224852005260 |
| H | 4.960888689503 | -1.913862264940 | -5.727663962569 |
| C | 4.099777802505 | 0.383293789396 | -7.886954650961 |

| | | | |
|---|---|---|---|
| H | 4.813935370996 | 0.853659282542 | -7.204421842744 |
| H | 4.669394340532 | -0.069431185197 | -8.704470318373 |
| H | 3.476012524873 | 1.174428376473 | -8.314142287364 |
| C | 1.784063289906 | -1.631723968616 | -8.202354433740 |
| H | 2.278091363899 | -2.119865096584 | -9.048087152060 |
| H | 1.150249218506 | -2.376345169035 | -7.711252271797 |
| H | 1.130715175940 | -0.849810487238 | -8.601865743972 |

transVAPNB_TS-F2000

($E_F$ = -840.36231289 a.u.; $G_F$ = -840.02562786 a.u.)

0 1

| | | | |
|---|---|---|---|
| C | 2.040731622917 | -0.050285973321 | -5.553435339033 |
| C | 2.958149541622 | 0.670165254368 | -4.513620735103 |
| C | 1.589147763656 | -0.773724495674 | -3.225922211757 |
| C | 1.178430395742 | -1.031506668171 | -4.691498379023 |
| H | 1.386631854837 | 0.681559555966 | -6.035450987153 |
| H | 0.106670680839 | -0.882983068797 | -4.846377895268 |
| H | 1.386214520711 | -2.077744628140 | -4.941560843075 |
| C | 3.072334039496 | -0.355437583869 | -3.375280481380 |
| H | 3.491013647084 | 0.073969079657 | -2.461386081254 |
| H | 3.690258207276 | -1.210435227667 | -3.665656402679 |
| C | 2.359937543372 | 1.946172790741 | -3.983638997229 |
| H | 2.869450396300 | 2.332352177635 | -3.101578095553 |
| C | 0.813957031627 | 0.335133891004 | -2.571194435191 |
| H | -0.112015674252 | 0.631719258190 | -3.057695866023 |
| H | 3.956040658552 | 0.893516949080 | -4.924904834233 |

| | | | |
|---|---|---|---|
| H | 1.507300359991 | -1.689882932296 | -2.625730209658 |
| C | 0.781084833318 | 0.362397272306 | -1.042173234577 |
| H | 1.807769147041 | 0.308229726710 | -0.661137611378 |
| H | 0.381103656421 | 1.325148778523 | -0.708375054022 |
| C | 1.890591493881 | 3.035646216436 | -4.951466275714 |
| H | 1.080296079331 | 2.649977813114 | -5.579792588733 |
| H | 1.452439014320 | 3.849223576190 | -4.365034957467 |
| C | 2.962251830494 | 3.687727214681 | -5.940625907805 |
| H | 3.783672242328 | 4.143660250084 | -5.382075508782 |
| H | 3.376957824798 | 2.937389130140 | -6.615966800871 |
| H | 2.468971736632 | 4.460756565676 | -6.533959850367 |
| C | -0.049285093852 | -0.780467172297 | -0.294051304197 |
| H | -1.101960251172 | -0.751873072138 | -0.586087409669 |
| H | 0.347463622810 | -1.771352983742 | -0.526219761802 |
| H | 0.021386568572 | -0.619455312221 | 0.783913004761 |
| Si | 2.958573865360 | -0.899804347779 | -6.980225441856 |
| C | 4.209509943692 | -2.158288807650 | -6.328754154506 |
| H | 3.731584087660 | -2.908598469010 | -5.692043683154 |
| H | 4.692019725194 | -2.685042210332 | -7.158031348873 |
| H | 4.994050587915 | -1.671455018716 | -5.742215580699 |
| C | 3.868116297078 | 0.390920380834 | -8.017992537338 |
| H | 4.625113902697 | 0.916963882555 | -7.428915216657 |
| H | 4.375792824200 | -0.078488832822 | -8.866393382922 |
| H | 3.174449571800 | 1.137727893494 | -8.416101413831 |
| C | 1.687716205019 | -1.786928849399 | -8.061066924513 |
| H | 2.169965502496 | -2.292334925385 | -8.903467919490 |

| | | | |
|---|---|---|---|
| H | 1.138265335202 | -2.541148707066 | -7.489861980460 |
| H | 0.957808661932 | -1.080207250344 | -8.468221718738 |

transVAPNB_P-F2000

($E_F$ = -840.46931877 a.u.; $G_F$ = -840.14007743 a.u.)

0 1

| | | | |
|---|---|---|---|
| C | 1.810910694508 | 0.398979760990 | -5.405138543033 |
| C | 2.722120959214 | 1.375854189465 | -4.583048645455 |
| C | 1.615709311361 | -0.138191147882 | -2.964665602658 |
| C | 0.940592778762 | -0.373611931822 | -4.351353388307 |
| H | 1.145288571065 | 0.983040327879 | -6.049060033384 |
| H | -0.072166778370 | 0.041018270700 | -4.320187307117 |
| H | 0.833594452626 | -1.440816160133 | -4.566397746224 |
| C | 2.080015820013 | 1.344081953763 | -3.143674159336 |
| H | 1.209005060172 | 2.009080892023 | -3.107841200463 |
| H | 2.782585756296 | 1.667871716193 | -2.371407375247 |
| C | 3.054791879439 | 2.741059897224 | -5.186176253611 |
| H | 3.573167341018 | 3.419654802591 | -4.510506789276 |
| C | 0.757379012562 | -0.527144744440 | -1.769756947427 |
| H | -0.062129337676 | 0.135529588828 | -1.498262255817 |
| H | 3.700982411938 | 0.882670160205 | -4.440130061236 |
| H | 2.522569054891 | -0.758135053051 | -2.937509055834 |
| C | 0.783168093834 | -1.941022822256 | -1.198964691190 |
| H | 0.522383770188 | -2.639220290147 | -2.013371276001 |
| H | 1.828136517988 | -2.190995834574 | -0.962595957358 |
| C | 2.766727201283 | 3.272050215380 | -6.582520133388 |

| | | | |
|---|---|---|---|
| H | 2.573703357784 | 2.435920162861 | -7.259724311401 |
| H | 1.814153145515 | 3.828801847367 | -6.549175710859 |
| C | 3.813672538781 | 4.233963618917 | -7.280827656998 |
| H | 4.004991541465 | 5.117207783079 | -6.665587466696 |
| H | 4.763635172890 | 3.721018651537 | -7.450962988362 |
| H | 3.414197103448 | 4.561158581232 | -8.243065315128 |
| C | -0.100630251632 | -2.336193898215 | 0.045969518753 |
| H | 0.161330326068 | -1.737539382067 | 0.922342086643 |
| H | -1.163796838403 | -2.189609239811 | -0.162878246286 |
| H | 0.063138102786 | -3.389262927768 | 0.283001456002 |
| Si | 2.790760547859 | -0.805487287324 | -6.505026768974 |
| C | 3.721018038430 | -2.022228924237 | -5.394779005734 |
| H | 3.039747904642 | -2.577021967104 | -4.743528525252 |
| H | 4.269519412509 | -2.749954416255 | -6.000835666890 |
| H | 4.447002918843 | -1.508119942361 | -4.758063242791 |
| C | 4.053193166299 | 0.101208060226 | -7.579796946052 |
| H | 4.657359795141 | 0.791922081897 | -6.984493603270 |
| H | 4.730623357714 | -0.615207829796 | -8.055044301571 |
| H | 3.571710753888 | 0.678546002142 | -8.373879325116 |
| C | 1.589738195644 | -1.757894981820 | -7.609111903125 |
| H | 2.119189163000 | -2.481351200169 | -8.236833530947 |
| H | 0.853258034693 | -2.307603210766 | -7.015428763795 |
| H | 1.044187081391 | -1.077837812313 | -8.270821743293 |

transVAPNB_R-F2000

($E_F$ = -840.41011198 a.u.; $G_F$ = -840.06758921 a.u.)

0 1

| | | | |
|---|---|---|---|
| C | 2.086503938910 | -0.075889364775 | -5.590943144015 |
| C | 2.947696638810 | 0.544666929463 | -4.449275107339 |
| C | 1.500673044801 | -0.771608898994 | -3.323094312905 |
| C | 1.107104610002 | -1.008540679470 | -4.796125905270 |
| H | 1.513660220903 | 0.707721175888 | -6.095535294534 |
| H | 0.057730746389 | -0.767293364227 | -4.986344034344 |
| H | 1.242384037478 | -2.065845980891 | -5.050385360467 |
| C | 3.028974703744 | -0.611961643593 | -3.437703789408 |
| H | 3.511095902690 | -0.325104867165 | -2.499629657109 |
| H | 3.524944187669 | -1.504000384732 | -3.824551817967 |
| C | 2.106118830873 | 1.593529623028 | -3.664142540614 |
| H | 2.768362666027 | 2.055752402607 | -2.920774449671 |
| C | 1.055062707552 | 0.643094329935 | -2.863912188455 |
| H | 0.036668847274 | 0.846478361302 | -3.215712754927 |
| H | 3.906493578194 | 0.949643341591 | -4.780000829770 |
| H | 1.154507897870 | -1.568135496500 | -2.664065757211 |
| C | 1.061196950603 | 0.769528752365 | -1.300497715994 |
| H | 2.101120358806 | 0.729036916740 | -0.956575702130 |
| H | 0.706740985416 | 1.774880184091 | -1.045095111793 |
| C | 1.537531903984 | 2.741034369219 | -4.562240764022 |
| H | 0.674910320088 | 2.358227998209 | -5.118518108386 |
| H | 1.134768339659 | 3.518451331876 | -3.903518861635 |
| C | 2.509057197505 | 3.433674749072 | -5.601488877616 |
| H | 3.380184515146 | 3.865700908398 | -5.100557172767 |
| H | 2.866697354109 | 2.721109357447 | -6.347031469574 |

| | | | |
|---|---|---|---|
| H | 1.975058962969 | 4.235628395247 | -6.115681300147 |
| C | 0.239391590632 | -0.272639551516 | -0.437560955912 |
| H | -0.808168952455 | -0.309663029292 | -0.750644870630 |
| H | 0.655958958312 | -1.278527678694 | -0.517971079697 |
| H | 0.273920345172 | 0.027549911878 | 0.612038806817 |
| Si | 3.039361075809 | -0.909587861204 | -7.002780109559 |
| C | 4.185076138714 | -2.284425208292 | -6.394987909783 |
| H | 3.638158044433 | -3.048136582747 | -5.834249881900 |
| H | 4.666745020927 | -2.778535005127 | -7.244795631459 |
| H | 4.974310282665 | -1.892116646655 | -5.747316873772 |
| C | 4.069324052553 | 0.396093091823 | -7.900971642391 |
| H | 4.788789691702 | 0.868721022814 | -7.225599628997 |
| H | 4.631418559541 | -0.046293663261 | -8.729264709677 |
| H | 3.432360499960 | 1.184248348033 | -8.314057237743 |
| C | 1.775816653240 | -1.647308641516 | -8.198749713858 |
| H | 2.268206190240 | -2.130579317494 | -9.048224418378 |
| H | 1.153941041703 | -2.398390080612 | -7.702243535234 |
| H | 1.110984074705 | -0.872610615275 | -8.593342794163 |

transVAPNB_TS-F1500

($E_F$ = -840.27251916 a.u.; $G_F$ = -839.93611987 a.u.)

0 1

| | | | |
|---|---|---|---|
| C | 2.035516827464 | -0.054302105757 | -5.546726754249 |
| C | 2.961296234090 | 0.673830954087 | -4.518091500237 |
| C | 1.598148739736 | -0.783329613396 | -3.215278065427 |
| C | 1.190670148307 | -1.046953741830 | -4.680773203578 |

| | | | |
|---|---|---|---|
| H | 1.369369846158 | 0.672454162576 | -6.019729739286 |
| H | 0.116541362853 | -0.916228243414 | -4.834979733128 |
| H | 1.414916619320 | -2.089860187940 | -4.930261943173 |
| C | 3.074127444671 | -0.338427557502 | -3.366196552439 |
| H | 3.484302797052 | 0.104076912521 | -2.454749936560 |
| H | 3.704535808938 | -1.188274742930 | -3.645806942004 |
| C | 2.388488963568 | 1.968502670290 | -4.010790257591 |
| H | 2.874824817932 | 2.348872715952 | -3.113603883063 |
| C | 0.793452683519 | 0.297103001039 | -2.553053076572 |
| H | -0.119499583024 | 0.610797287594 | -3.052477349584 |
| H | 3.961660420152 | 0.874696547808 | -4.936802142473 |
| H | 1.540076725407 | -1.704617641714 | -2.619078085823 |
| C | 0.771387960244 | 0.350532331798 | -1.034959499316 |
| H | 1.798483373255 | 0.291165685681 | -0.655352920943 |
| H | 0.378961978114 | 1.319808185454 | -0.710515329807 |
| C | 1.906258499504 | 3.044598579486 | -4.970582549194 |
| H | 1.106401762644 | 2.649680055162 | -5.606875123003 |
| H | 1.454703883476 | 3.853265035045 | -4.387219149292 |
| C | 2.986503820535 | 3.689618862859 | -5.931467897835 |
| H | 3.797631103024 | 4.145719229428 | -5.357745860006 |
| H | 3.416221669106 | 2.937598651583 | -6.595776451254 |
| H | 2.513329874727 | 4.463124715603 | -6.541197741231 |
| C | -0.066390093759 | -0.776472521953 | -0.302262631949 |
| H | -1.117012645168 | -0.736990839456 | -0.601186215037 |
| H | 0.320310122405 | -1.770502990081 | -0.539044185381 |
| H | -0.005609292494 | -0.629681215993 | 0.778845217517 |

| | | | |
|---|---|---|---|
| Si | 2.947658598459 | -0.896328174844 | -6.981570211360 |
| C | 4.211609822053 | -2.143546942257 | -6.333682594241 |
| H | 3.741997794850 | -2.899004823402 | -5.696938209074 |
| H | 4.698613911039 | -2.664810376506 | -7.163754429885 |
| H | 4.991730361382 | -1.649242010226 | -5.747469465000 |
| C | 3.841985145076 | 0.398929123982 | -8.026719506463 |
| H | 4.607501629744 | 0.922252478364 | -7.446366419595 |
| H | 4.337509335459 | -0.067236397649 | -8.884041698780 |
| H | 3.143051819201 | 1.147562648263 | -8.411870435260 |
| C | 1.676512575829 | -1.794029594754 | -8.053122339646 |
| H | 2.157190273207 | -2.298414838676 | -8.897021389962 |
| H | 1.134943482615 | -2.550206660312 | -7.477091033911 |
| H | 0.940045184264 | -1.092817493435 | -8.457889122170 |

transVAPNB_R-F1500

($E_F$ = -840.33306422 a.u.; $G_F$ = -839.99015163 a.u.)

0 1

| | | | |
|---|---|---|---|
| C | 2.084075762259 | -0.091558086620 | -5.587676708755 |
| C | 2.953081131198 | 0.536500108407 | -4.456165200214 |
| C | 1.523731636621 | -0.784419479033 | -3.313165186658 |
| C | 1.123420316715 | -1.035153803589 | -4.782186348783 |
| H | 1.497429928967 | 0.687746460431 | -6.083609590798 |
| H | 0.069441390490 | -0.810598684463 | -4.967090164177 |
| H | 1.273503065391 | -2.091440962659 | -5.032357628126 |
| C | 3.050005056448 | -0.615962773822 | -3.440550438485 |
| H | 3.538282489943 | -0.321874479069 | -2.507846934607 |

| | | | |
|---|---|---|---|
| H | 3.548205898089 | -1.506695154070 | -3.827726456854 |
| C | 2.111458544634 | 1.577382186963 | -3.661255121856 |
| H | 2.778210727157 | 2.037760736393 | -2.920278951332 |
| C | 1.071207221390 | 0.634558463864 | -2.870819231965 |
| H | 0.052735811602 | 0.828005781573 | -3.228870016676 |
| H | 3.906593072442 | 0.945961215716 | -4.795992777610 |
| H | 1.186683829018 | -1.576745325670 | -2.644393279433 |
| C | 1.073539202437 | 0.795663031982 | -1.320555930312 |
| H | 2.107638284273 | 0.721354799977 | -0.964347456192 |
| H | 0.754287190040 | 1.818423859554 | -1.087720024690 |
| C | 1.523862659932 | 2.723360167773 | -4.532106824105 |
| H | 0.672382364408 | 2.337627094924 | -5.103521535910 |
| H | 1.104026761206 | 3.481401369715 | -3.861270034506 |
| C | 2.502606315926 | 3.429869124692 | -5.532870687723 |
| H | 3.368567311868 | 3.846777124445 | -5.009915773587 |
| H | 2.870560979623 | 2.731836130109 | -6.287639416897 |
| H | 1.986377911479 | 4.246552307917 | -6.042772810203 |
| C | 0.202415873153 | -0.199201131184 | -0.477170436848 |
| H | -0.840100962593 | -0.192829243516 | -0.809782418405 |
| H | 0.574793053849 | -1.223054388039 | -0.550257186283 |
| H | 0.225930348209 | 0.093917325676 | 0.575097988618 |
| Si | 3.030843549428 | -0.911472164099 | -7.011071472519 |
| C | 4.203653278925 | -2.268258891389 | -6.414805416231 |
| H | 3.673521777553 | -3.042164479978 | -5.851916890572 |
| H | 4.687856863280 | -2.752522670727 | -7.268816434339 |
| H | 4.990431432944 | -1.863873078937 | -5.771555495166 |

| | | | |
|---|---|---|---|
| C | 4.030608367624 | 0.410851653545 | -7.918950424375 |
| H | 4.755540085489 | 0.888090085972 | -7.252700107668 |
| H | 4.584139991650 | -0.019838080406 | -8.759072434591 |
| H | 3.377450875836 | 1.193847488836 | -8.316371506485 |
| C | 1.765424598482 | -1.667821905544 | -8.193242054806 |
| H | 2.255685527371 | -2.144731473335 | -9.047532663151 |
| H | 1.159493936190 | -2.427244942705 | -7.689749900128 |
| H | 1.085600064840 | -0.902922578115 | -8.581349929863 |

transVAPNB_P-F1500

($E_F$ = -840.34830740 a.u.; $G_F$ = -840.01913832 a.u.)

0 1

| | | | |
|---|---|---|---|
| C | 1.825012768664 | 0.375477967499 | -5.406383826865 |
| C | 2.731685005239 | 1.343886621750 | -4.572352746676 |
| C | 1.618361496778 | -0.155178898099 | -2.970559838082 |
| C | 0.959363088031 | -0.409628698245 | -4.361121757749 |
| H | 1.155782083975 | 0.965834778003 | -6.040508676197 |
| H | -0.061572181823 | -0.015047678009 | -4.341717062285 |
| H | 0.875743483233 | -1.479964899044 | -4.570672780870 |
| C | 2.075435510134 | 1.322103495472 | -3.146044457239 |
| H | 1.201872189424 | 1.984563255632 | -3.125177246680 |
| H | 2.767104062891 | 1.651816405012 | -2.366462431804 |
| C | 3.064705392878 | 2.703812136160 | -5.161769378413 |
| H | 3.620217669692 | 3.365557311612 | -4.499033187489 |
| C | 0.750251740516 | -0.524923722631 | -1.789119096442 |
| H | -0.077651640569 | 0.135534330376 | -1.538602005793 |

| | | | |
|---|---|---|---|
| H | 3.706748343700 | 0.846756169604 | -4.418600826551 |
| H | 2.526738240292 | -0.772314636731 | -2.925883383620 |
| C | 0.775548093325 | -1.924999720996 | -1.207811629746 |
| H | 0.516667883450 | -2.637230544617 | -2.011036128998 |
| H | 1.817307134808 | -2.175000226595 | -0.957367981626 |
| C | 2.721526076466 | 3.257705641180 | -6.527406785492 |
| H | 2.470768903983 | 2.440159294419 | -7.208409648593 |
| H | 1.793998776751 | 3.850938885355 | -6.446253982348 |
| C | 3.784476195135 | 4.176856525078 | -7.229084806104 |
| H | 4.036847521762 | 5.034317207118 | -6.599091610815 |
| H | 4.703768418105 | 3.622609059895 | -7.435255355280 |
| H | 3.383352406259 | 4.550325760132 | -8.174090430162 |
| C | -0.121957710289 | -2.263498007719 | 0.026830780579 |
| H | 0.138141919922 | -1.639138540109 | 0.885892242948 |
| H | -1.179698574179 | -2.103904396998 | -0.200415388366 |
| H | 0.014286233048 | -3.310055478104 | 0.307409007125 |
| Si | 2.810901451168 | -0.803853829258 | -6.526461417172 |
| C | 3.765186078281 | -2.019263849311 | -5.435630825510 |
| H | 3.096158308229 | -2.582301184709 | -4.778645337240 |
| H | 4.311630655025 | -2.739950860583 | -6.051839636736 |
| H | 4.494965104297 | -1.502687126254 | -4.805311728991 |
| C | 4.049400522063 | 0.138833575654 | -7.597629729748 |
| H | 4.647712978465 | 0.827920818430 | -6.994547342987 |
| H | 4.733404958315 | -0.557220867159 | -8.093155852794 |
| H | 3.550694382947 | 0.724021054045 | -8.375229033865 |
| C | 1.614529764080 | -1.759979190524 | -7.632225285200 |

| | | | |
|---|---|---|---|
| H | 2.148870622949 | -2.467675412353 | -8.273612757335 |
| H | 0.891618023573 | -2.327840494447 | -7.038928851620 |
| H | 1.053105804152 | -1.079906256383 | -8.280501980305 |

transVAPNB_TS-F1000

($E_F$ = -840.18242944 a.u.; $G_F$ = -839.84686569 a.u.)

0 1

| | | | |
|---|---|---|---|
| C | 2.024777070281 | -0.065637988457 | -5.531248527656 |
| C | 2.964254441155 | 0.677381483194 | -4.522755548958 |
| C | 1.613706166737 | -0.798208377225 | -3.192927551856 |
| C | 1.215214273503 | -1.079806033683 | -4.657310157008 |
| H | 1.334855836985 | 0.651195267214 | -5.984630007590 |
| H | 0.137309823366 | -0.986805413450 | -4.812351184439 |
| H | 1.474769296499 | -2.115629085335 | -4.901830755127 |
| C | 3.076500267959 | -0.309083563704 | -3.346505228569 |
| H | 3.470381715324 | 0.158342703049 | -2.440377371886 |
| H | 3.728694771865 | -1.149684396504 | -3.605061365314 |
| C | 2.429378723300 | 2.001083831325 | -4.056137154131 |
| H | 2.881711090900 | 2.378707231369 | -3.140634584517 |
| C | 0.762524664404 | 0.240157261300 | -2.526531188987 |
| H | -0.130447436269 | 0.574517589433 | -3.047199951498 |
| H | 3.966800163060 | 0.843689572739 | -4.953513644259 |
| H | 1.592948775941 | -1.722203532398 | -2.597211347714 |
| C | 0.744280851491 | 0.323904550813 | -1.019502859506 |
| H | 1.770123641104 | 0.260453779831 | -0.636620845424 |
| H | 0.357654792610 | 1.300269880787 | -0.708692858542 |

| | | | |
|---|---|---|---|
| C | 1.947200149263 | 3.059519448840 | -5.020290104506 |
| H | 1.175099243496 | 2.649188332074 | -5.680861746272 |
| H | 1.464639129944 | 3.860801149612 | -4.451225807202 |
| C | 3.050598632298 | 3.706675185721 | -5.930984936814 |
| H | 3.833177201860 | 4.171983521530 | -5.325483046180 |
| H | 3.516586325074 | 2.956173747227 | -6.572425159807 |
| H | 2.603915468625 | 4.474812885605 | -6.567832039834 |
| C | -0.104044800786 | -0.786140681800 | -0.301589753388 |
| H | -1.151474110730 | -0.735528530233 | -0.610592806419 |
| H | 0.273033625930 | -1.783718854866 | -0.540578493009 |
| H | -0.056064641083 | -0.651163380567 | 0.782257028487 |
| Si | 2.925100334272 | -0.889858960535 | -6.983521081805 |
| C | 4.216136482794 | -2.115653914785 | -6.348073511319 |
| H | 3.764181518876 | -2.884643962479 | -5.714769129031 |
| H | 4.712180712922 | -2.621524795478 | -7.182272507192 |
| H | 4.987067451411 | -1.608647632812 | -5.760397674810 |
| C | 3.787135216089 | 0.418063112719 | -8.039872543676 |
| H | 4.568649897905 | 0.935409835822 | -7.475817098450 |
| H | 4.258565864911 | -0.038317409517 | -8.915876349436 |
| H | 3.077127738938 | 1.170363614327 | -8.396577891222 |
| C | 1.653419626411 | -1.806640624775 | -8.037910752723 |
| H | 2.130607964974 | -2.307346066140 | -8.885970800965 |
| H | 1.128015720793 | -2.567770176375 | -7.453475040087 |
| H | 0.903668120524 | -1.115769482865 | -8.436092978627 |

transVAPNB_P-F1000

($E_F$ = -840.22886855 a.u.; $G_F$ = -839.89911672 a.u.)

0 1

| | | | |
|---|---|---|---|
| C | 1.844218364515 | 0.306068535220 | -5.446526293296 |
| C | 2.776624447077 | 1.191136341648 | -4.558326525046 |
| C | 1.554174484334 | -0.279296720371 | -3.029772206447 |
| C | 0.964487747494 | -0.520090603642 | -4.448472447710 |
| H | 1.184882729983 | 0.959658256380 | -6.026840270134 |
| H | -0.068686176072 | -0.159059944771 | -4.467011698881 |
| H | 0.921481545886 | -1.589502334337 | -4.673886882894 |
| C | 2.053972780868 | 1.191938425484 | -3.167604765964 |
| H | 1.197700115722 | 1.875877098492 | -3.188771745558 |
| H | 2.714186051604 | 1.497357965287 | -2.351688058727 |
| C | 3.212964902368 | 2.530666287786 | -5.095283588739 |
| H | 3.913967156749 | 3.074352566993 | -4.463962743858 |
| C | 0.612032584863 | -0.609552214399 | -1.910248960969 |
| H | -0.344828360026 | -0.090541562538 | -1.889785541951 |
| H | 3.707182893842 | 0.628894026648 | -4.368086592804 |
| H | 2.449261116535 | -0.906934335763 | -2.924734300880 |
| C | 0.960152180651 | -1.513045506798 | -0.756096852855 |
| H | 1.685522867402 | -2.262225897857 | -1.099822906132 |
| H | 1.502230984172 | -0.932031935900 | 0.011473725055 |
| C | 2.748276156874 | 3.242107702360 | -6.337977991913 |
| H | 2.328674781799 | 2.524707639869 | -7.048870498786 |
| H | 1.910707027708 | 3.915692852048 | -6.085311246370 |
| C | 3.827832698229 | 4.096021625516 | -7.069387308791 |
| H | 4.250793285084 | 4.849260886084 | -6.398475092429 |

| | | | |
|---|---|---|---|
| H | 4.644548213174 | 3.464466309716 | -7.428121145846 |
| H | 3.386872053154 | 4.611453160321 | -7.926215031761 |
| C | -0.217184663682 | -2.241298839610 | -0.046921671344 |
| H | -0.938516954516 | -1.522570622214 | 0.352727631265 |
| H | -0.746311988411 | -2.898164937860 | -0.742624208355 |
| H | 0.155805955918 | -2.845723986489 | 0.783348316970 |
| Si | 2.785073718447 | -0.803300257110 | -6.669481622270 |
| C | 3.777277028105 | -2.081731778874 | -5.691112302043 |
| H | 3.132864915337 | -2.672517180677 | -5.033644453435 |
| H | 4.287266532589 | -2.774327299828 | -6.367769488227 |
| H | 4.540543375904 | -1.604398361319 | -5.069593225616 |
| C | 3.976132755197 | 0.213884935907 | -7.724726207799 |
| H | 4.623019842527 | 0.832643743246 | -7.096177195653 |
| H | 4.613783329327 | -0.439608634002 | -8.328181004339 |
| H | 3.440942287772 | 0.880342762473 | -8.406949520821 |
| C | 1.544782201519 | -1.689401554515 | -7.785093481074 |
| H | 2.052107031086 | -2.353962312217 | -8.491285916946 |
| H | 0.845787237522 | -2.294737334482 | -7.200288686572 |
| H | 0.958433091285 | -0.970273324519 | -8.365626927393 |

transVAPNB_R-F1000

($E_F$ = -840.25735855 a.u.; $G_F$ = -839.91424893 a.u.)

0 1

| | | | |
|---|---|---|---|
| C | 2.080035716855 | -0.111699318954 | -5.583212932118 |
| C | 2.960173787644 | 0.526285502130 | -4.465953895725 |
| C | 1.554627489338 | -0.800912864314 | -3.300037918708 |

| | | | |
|---|---|---|---|
| C | 1.144528554855 | -1.068994138262 | -4.763365186280 |
| H | 1.475572687054 | 0.661816429557 | -6.067409331005 |
| H | 0.084900037813 | -0.865702457126 | -4.940081969473 |
| H | 1.313195604266 | -2.123588271370 | -5.008887554042 |
| C | 3.077980953694 | -0.620451274913 | -3.445160784986 |
| H | 3.574436330830 | -0.316658200862 | -2.519820866525 |
| H | 3.579110326372 | -1.509430330241 | -3.832826084662 |
| C | 2.120948057958 | 1.558394362753 | -3.658857856622 |
| H | 2.793670532359 | 2.015288170639 | -2.920593997743 |
| C | 1.092229462204 | 0.621587645949 | -2.876683041171 |
| H | 0.074147547461 | 0.803311124582 | -3.242749526138 |
| H | 3.906640880132 | 0.940690164060 | -4.818764232287 |
| H | 1.230849380433 | -1.588121108414 | -2.618521959947 |
| C | 1.087549977559 | 0.819186439617 | -1.339943828888 |
| H | 2.110941702580 | 0.697186327737 | -0.965800129604 |
| H | 0.817880908841 | 1.861720752553 | -1.132267345781 |
| C | 1.511812492343 | 2.705306148017 | -4.497493200347 |
| H | 0.674679258311 | 2.317658168754 | -5.088602200925 |
| H | 1.070583106378 | 3.436694824169 | -3.810673988929 |
| C | 2.495862284592 | 3.437530082180 | -5.453131532124 |
| H | 3.352466939945 | 3.840336012607 | -4.903774707648 |
| H | 2.879927536776 | 2.761284709733 | -6.220239288776 |
| H | 1.993282687976 | 4.268947337517 | -5.953535078489 |
| C | 0.150591561662 | -0.112505032901 | -0.520145614957 |
| H | -0.883397997707 | -0.040221050897 | -0.871875471268 |
| H | 0.456933345812 | -1.158410894769 | -0.592093737220 |

| | | | |
|---|---|---|---|
| H | 0.168736813661 | 0.169208377594 | 0.535705259037 |
| Si | 3.016816635848 | -0.914948930388 | -7.022017577808 |
| C | 4.223958352083 | -2.247902074522 | -6.441147665605 |
| H | 3.715873719905 | -3.033261899222 | -5.873802705551 |
| H | 4.709423486176 | -2.721034269455 | -7.300641786049 |
| H | 5.008501355592 | -1.827715180209 | -5.805303114032 |
| C | 3.976722560888 | 0.426975795097 | -7.943931720728 |
| H | 4.708710065193 | 0.911203668008 | -7.290535204698 |
| H | 4.518315867385 | 0.009988218775 | -8.798598140664 |
| H | 3.303119920981 | 1.202376298717 | -8.321726827118 |
| C | 1.748070971962 | -1.696102435945 | -8.184220233087 |
| H | 2.234164344533 | -2.164513945740 | -9.045570689449 |
| H | 1.164543872519 | -2.466386244748 | -7.670846646588 |
| H | 1.047892625183 | -0.944735948248 | -8.562442638308 |

transVAPNB_TS-F500

($E_F$ = -840.09107784 a.u.; $G_F$ = -839.75788261 a.u.)

0 1

| | | | |
|---|---|---|---|
| C | 2.015094823460 | -0.155598662728 | -5.436085381058 |
| C | 2.993476627645 | 0.640629222072 | -4.493478376692 |
| C | 1.686960055798 | -0.850568062349 | -3.084035776022 |
| C | 1.371917522151 | -1.256115497618 | -4.536146229076 |
| H | 1.224742974708 | 0.520548320023 | -5.774098271441 |
| H | 0.298636735079 | -1.357583301397 | -4.716593504033 |
| H | 1.817219866007 | -2.238696160358 | -4.728482575882 |
| C | 3.108127833283 | -0.247294552191 | -3.237126152466 |

| | | | |
|---|---|---|---|
| H | 3.436783092200 | 0.310390819034 | -2.356431072456 |
| H | 3.826641071256 | -1.056461438932 | -3.409271068334 |
| C | 2.519022526123 | 2.023767749434 | -4.161095638421 |
| H | 2.862556259012 | 2.433738355478 | -3.213419122351 |
| C | 0.710672685019 | 0.128488891640 | -2.509753033401 |
| H | -0.120863734254 | 0.457803766593 | -3.125648990589 |
| H | 3.994251676234 | 0.726860531318 | -4.952890055299 |
| H | 1.734857619720 | -1.733928577328 | -2.430561643978 |
| C | 0.601925440854 | 0.305914736169 | -1.023924550098 |
| H | 1.602997750057 | 0.282100326227 | -0.575424766335 |
| H | 0.180077532445 | 1.290246010093 | -0.792995196777 |
| C | 2.138972077437 | 3.024837960134 | -5.214293015044 |
| H | 1.555388515412 | 2.543647652641 | -6.006580303478 |
| H | 1.487823729346 | 3.787324272148 | -4.771728108015 |
| C | 3.348174230228 | 3.748356862290 | -5.879178712348 |
| H | 3.933221980180 | 4.294435136606 | -5.133958049851 |
| H | 4.010083360067 | 3.032430939912 | -6.370886643195 |
| H | 2.999472689360 | 4.461666637795 | -6.631484743839 |
| C | -0.270448222314 | -0.779738999526 | -0.323864257372 |
| H | -1.294753928434 | -0.759817187460 | -0.705761875046 |
| H | 0.135615849473 | -1.779530214485 | -0.499544660876 |
| H | -0.302832462383 | -0.609232198326 | 0.756200040367 |
| Si | 2.826145253499 | -0.860305251965 | -6.997381740081 |
| C | 4.263361212089 | -1.987659181084 | -6.509732777329 |
| H | 3.924505871192 | -2.832490335394 | -5.903116771483 |
| H | 4.758423177417 | -2.393783146996 | -7.397261649579 |

| | | | |
|---|---|---|---|
| H | 5.013969459565 | -1.440907265238 | -5.930970581616 |
| C | 3.480901983542 | 0.540391032042 | -8.082368078727 |
| H | 4.302485624501 | 1.070670980535 | -7.593044019030 |
| H | 3.857741950530 | 0.152689694450 | -9.033934770777 |
| H | 2.697481015355 | 1.271283204041 | -8.304732799678 |
| C | 1.539412403567 | -1.854019563624 | -7.959561064304 |
| H | 1.973610894930 | -2.296207281810 | -8.861586028938 |
| H | 1.132567961065 | -2.667240400539 | -7.350982943959 |
| H | 0.703538822499 | -1.218134700771 | -8.268061398375 |

transVAPNB_R-F500

($E_F$ = -840.18312030 a.u.; $G_F$ = -839.83966797 a.u.)

0 1

| | | | |
|---|---|---|---|
| C | 2.067164242607 | -0.143509888152 | -5.573763654433 |
| C | 2.968424240486 | 0.516989943043 | -4.486667618008 |
| C | 1.616024361240 | -0.825260931588 | -3.273675367204 |
| C | 1.180515493820 | -1.122122828998 | -4.724033150760 |
| H | 1.429878299807 | 0.616507694243 | -6.037610451788 |
| H | 0.111411256289 | -0.954003664422 | -4.878870559201 |
| H | 1.377832327700 | -2.173129959333 | -4.963670173577 |
| C | 3.132026375808 | -0.618410738636 | -3.458061734233 |
| H | 3.645567369992 | -0.295858396595 | -2.548397706067 |
| H | 3.639553438658 | -1.502326485382 | -3.849247412546 |
| C | 2.135487519964 | 1.535116222591 | -3.658255149190 |
| H | 2.820022624360 | 1.992503065823 | -2.930649514102 |
| C | 1.133682308137 | 0.597322541290 | -2.871406102336 |

| | | | |
|---|---|---|---|
| H | 0.112283274693 | 0.756561061253 | -3.239448844835 |
| H | 3.898548154966 | 0.942892229555 | -4.867862909955 |
| H | 1.322792925085 | -1.605901226040 | -2.570540372734 |
| C | 1.129189443273 | 0.837821620695 | -1.349338371267 |
| H | 2.137815814363 | 0.659862527821 | -0.957320334931 |
| H | 0.921377723190 | 1.899847064547 | -1.170077945659 |
| C | 1.484633806508 | 2.679580720398 | -4.452740113766 |
| H | 0.665006283794 | 2.284265552727 | -5.063092372955 |
| H | 1.015402585740 | 3.369392433331 | -3.741520274197 |
| C | 2.455980232259 | 3.462161251986 | -5.360623928499 |
| H | 3.298347320575 | 3.859904190000 | -4.785684938527 |
| H | 2.863952103773 | 2.822188617914 | -6.147101964779 |
| H | 1.949340148741 | 4.303540827824 | -5.840647360037 |
| C | 0.120664267193 | -0.013603128750 | -0.550572114744 |
| H | -0.899773540091 | 0.143754590725 | -0.914369904240 |
| H | 0.340807829964 | -1.080977271427 | -0.629947072521 |
| H | 0.143719221334 | 0.252061883533 | 0.509693231083 |
| Si | 2.979709063931 | -0.924035173007 | -7.039818790742 |
| C | 4.244653337280 | -2.215085338152 | -6.488598882335 |
| H | 3.776804200735 | -3.014724759477 | -5.906786166839 |
| H | 4.723964432392 | -2.674800102741 | -7.358721518390 |
| H | 5.030234214421 | -1.767203816633 | -5.873258375919 |
| C | 3.867576238958 | 0.444705492764 | -7.993745364255 |
| H | 4.611845531665 | 0.945583846010 | -7.367284466465 |
| H | 4.385761808434 | 0.045525628873 | -8.871088923618 |
| H | 3.159337619005 | 1.203610755286 | -8.340541404615 |

| | | | |
|---|---|---|---|
| C | 1.700812394177 | -1.750156041695 | -8.158897406157 |
| H | 2.174248617728 | -2.206109448758 | -9.033879851214 |
| H | 1.159488023844 | -2.536864144971 | -7.624618396630 |
| H | 0.964730411754 | -1.023598232418 | -8.516869649643 |

transVAPNB_P-F500

($E_F$ = -840.11170128 a.u.; $G_F$ = -839.78118002 a.u.)

0 1

| | | | |
|---|---|---|---|
| C | 1.895471520401 | 0.223337957081 | -5.467105524373 |
| C | 2.868871851829 | 1.024826472334 | -4.547999420662 |
| C | 1.523166028911 | -0.355347749792 | -3.060505945288 |
| C | 0.995273837620 | -0.612445762085 | -4.498941001339 |
| H | 1.253993400239 | 0.936274413117 | -5.995320766096 |
| H | -0.044279407129 | -0.277484820532 | -4.566269448663 |
| H | 0.987207256689 | -1.684594344411 | -4.715152096177 |
| C | 2.098246344653 | 1.086470962443 | -3.189459141127 |
| H | 1.280050162538 | 1.812774820984 | -3.254092999444 |
| H | 2.740300881553 | 1.366222219359 | -2.350359855989 |
| C | 3.403572894071 | 2.329359891325 | -5.052063922495 |
| H | 4.224922452012 | 2.754309999634 | -4.477927106897 |
| C | 0.519987338907 | -0.586925616853 | -1.983832963798 |
| H | -0.459119703237 | -0.124992447752 | -2.103111034586 |
| H | 3.745428341683 | 0.393365997384 | -4.331134994105 |
| H | 2.382907843781 | -1.016658992934 | -2.886819819370 |
| C | 0.828668823937 | -1.240218763819 | -0.670230819101 |
| H | 1.716357368572 | -1.874997768146 | -0.781504372716 |

| | | | |
|---|---|---|---|
| H | 1.113242874853 | -0.473764656402 | 0.072202835143 |
| C | 2.833901835952 | 3.193282966699 | -6.136482573877 |
| H | 2.289202994100 | 2.582462351441 | -6.862824791827 |
| H | 2.078784173367 | 3.880703787629 | -5.716104291448 |
| C | 3.890849499499 | 4.040350074605 | -6.884030453771 |
| H | 4.438841380764 | 4.683582480788 | -6.189129853448 |
| H | 4.615190082346 | 3.396615167211 | -7.389577230495 |
| H | 3.416933317966 | 4.679210819869 | -7.633864047474 |
| C | -0.326164779782 | -2.075442843533 | -0.068092635332 |
| H | -1.216059997231 | -1.457501458810 | 0.084219857272 |
| H | -0.599894122418 | -2.898308880472 | -0.733970476376 |
| H | -0.037620687206 | -2.496026083400 | 0.898809687028 |
| Si | 2.765266404128 | -0.837399296317 | -6.779574314842 |
| C | 3.796679415348 | -2.162623762209 | -5.910867082692 |
| H | 3.183293209138 | -2.770831205170 | -5.239505762573 |
| H | 4.258130089223 | -2.835110112923 | -6.640549458493 |
| H | 4.600073618494 | -1.716685648582 | -5.317243285471 |
| C | 3.900024637055 | 0.226670483109 | -7.849578713172 |
| H | 4.612108038872 | 0.778883970421 | -7.229726906079 |
| H | 4.467454372380 | -0.391183708214 | -8.552487732367 |
| H | 3.330712324713 | 0.957802950020 | -8.430805520256 |
| C | 1.461058544032 | -1.666578892368 | -7.865965857186 |
| H | 1.925617337584 | -2.300404206210 | -8.627654500903 |
| H | 0.790612349152 | -2.294909894247 | -7.272072453431 |
| H | 0.848961564725 | -0.919660794566 | -8.381212796348 |

Cam_TS1-F3500

($E_F$ = -929.94992891 a.u.; $G_F$ = -929.56046475 a.u.)

0 1

| | | | |
|---|---|---|---|
| C | -1.822153526586 | 1.024127495599 | -2.610955382130 |
| C | -1.199171812584 | 2.323666140222 | -1.991367338219 |
| C | -1.975812923301 | 2.135437820212 | -0.175663528773 |
| C | -2.517658523680 | 0.734876950933 | -0.447632267995 |
| C | -1.373689633913 | -0.296108656584 | -0.613329397866 |
| C | -0.957308391500 | -0.156565621389 | -2.100474551518 |
| H | -0.185602794946 | 2.137817567580 | -1.630346564944 |
| H | -1.035318953506 | 2.157239293707 | 0.379285188679 |
| H | -3.223526676726 | 0.456707101565 | 0.338303545183 |
| H | -0.550262802348 | -0.091046396399 | 0.075218588303 |
| H | -1.728185043299 | -1.303992828392 | -0.389347450633 |
| H | -1.181423444574 | -1.063653923726 | -2.666274523602 |
| H | 0.109259409205 | 0.040607824085 | -2.231803849728 |
| C | -3.171842100026 | 0.801859025314 | -1.852039958148 |
| C | -3.904911255555 | -0.502418991362 | -2.208327639568 |
| H | -4.288447702834 | -0.467754455420 | -3.231517987139 |
| H | -3.285558453419 | -1.395194389674 | -2.115645469352 |
| H | -4.765032190207 | -0.628224914933 | -1.543802370731 |
| C | -4.163181425222 | 1.960541526357 | -2.040486158126 |
| H | -4.550634446206 | 1.954802725024 | -3.063580914582 |
| H | -5.010631315284 | 1.851038589359 | -1.358691095322 |
| H | -3.714861211128 | 2.935503659266 | -1.854164697925 |
| O | -1.120499471930 | 3.407820765178 | -2.947418998591 |

| | | | |
|---|---|---|---|
| O | -2.974248467580 | 2.873870204238 | 0.602073379160 |
| C | -1.940390123504 | 1.058601420337 | -4.131259737663 |
| H | -0.957343230699 | 1.083101360252 | -4.606449594101 |
| H | -2.446356863922 | 0.156636788904 | -4.484146713982 |
| H | -2.500050139287 | 1.930005192052 | -4.470097195150 |
| C | 0.143199547593 | 3.476271973277 | -3.741038208192 |
| H | 0.651972910247 | 2.510324932581 | -3.700274334974 |
| H | 0.787141769018 | 4.223657252348 | -3.270164501532 |
| C | -0.005541831246 | 3.862108116225 | -5.280051536511 |
| H | -0.666711362093 | 3.142078518049 | -5.774799743418 |
| H | -0.455880859420 | 4.859017364216 | -5.361238218971 |
| C | -2.959684122104 | 2.543465082364 | 2.054218976452 |
| H | -2.345625890980 | 3.292541697734 | 2.562487017687 |
| H | -2.495853021764 | 1.564758762698 | 2.204964376961 |
| C | -4.373055425216 | 2.496347490980 | 2.783594262475 |
| H | -4.873576800069 | 3.466307384392 | 2.674244469579 |
| H | -4.997571180018 | 1.728637728713 | 2.308688405818 |
| O | 1.318371620862 | 3.844844915585 | -5.894574309572 |
| C | 1.535967605376 | 4.162497886020 | -7.338694432125 |
| H | 0.998265968203 | 3.455601039974 | -7.979163521120 |
| H | 1.221315649747 | 5.186499820547 | -7.565107272030 |
| H | 2.609882538129 | 4.057111780024 | -7.476290923761 |
| O | -4.156138471811 | 2.183787109066 | 4.192676887471 |
| C | -5.273989283578 | 2.069684875252 | 5.177311334754 |
| H | -5.820523174950 | 3.014868759472 | 5.259800294145 |
| H | -5.960694216076 | 1.263867828816 | 4.897722694557 |

| | | | |
|---|---|---|---|
| H | -4.778641972400 | 1.835726601679 | 6.117126879210 |

Cam-F3500

($E_F$ = -929.95033850 a.u.; $G_F$ = -929.56055311 a.u.)

0 1

| | | | |
|---|---|---|---|
| C | -1.856188723878 | 1.002742275394 | -2.601119112226 |
| C | -1.220880653807 | 2.279769894042 | -1.921748001820 |
| C | -1.905623309765 | 2.125849387389 | -0.249708022841 |
| C | -2.485142124511 | 0.717227635094 | -0.435798428944 |
| C | -1.359492381095 | -0.328753802998 | -0.632020615700 |
| C | -0.990671862987 | -0.193234263736 | -2.134344493504 |
| H | -0.190810939345 | 2.056602378034 | -1.633862378465 |
| H | -0.988867268585 | 2.139439568622 | 0.344796180622 |
| H | -3.151177244274 | 0.472936728380 | 0.393677609656 |
| H | -0.511974747416 | -0.129057254042 | 0.028616040790 |
| H | -1.715876832689 | -1.332410186766 | -0.393264995668 |
| H | -1.247020391407 | -1.094780477210 | -2.695292488232 |
| H | 0.073425392712 | -0.009244463257 | -2.301107972270 |
| C | -3.187729232308 | 0.774883927281 | -1.816859072684 |
| C | -3.929815894469 | -0.529681091464 | -2.150422038589 |
| H | -4.314463249965 | -0.508496902604 | -3.173786037151 |
| H | -3.317906705874 | -1.425842327519 | -2.044203982025 |
| H | -4.790086478606 | -0.638510517498 | -1.482959131803 |
| C | -4.183605223601 | 1.932880470238 | -1.993438538533 |
| H | -4.609522563469 | 1.899930624808 | -3.000728153056 |
| H | -5.004178468128 | 1.844333954451 | -1.277011117687 |

| | | | |
|---|---|---|---|
| H | -3.730496296434 | 2.913219385791 | -1.852621758886 |
| O | -1.138313840635 | 3.408251490032 | -2.842322193710 |
| O | -2.900764936641 | 2.918506591355 | 0.494139160848 |
| C | -1.998015513073 | 1.090560611948 | -4.114380673674 |
| H | -1.023822100664 | 1.105043995824 | -4.608032155924 |
| H | -2.534598244109 | 0.214191601460 | -4.487043801470 |
| H | -2.541148830926 | 1.986794516936 | -4.413448615713 |
| C | 0.109487005903 | 3.453662268397 | -3.658848276157 |
| H | 0.582702314388 | 2.468963611263 | -3.658696266161 |
| H | 0.791228503996 | 4.159243277845 | -3.176093494099 |
| C | -0.038490273133 | 3.892824362698 | -5.183331282447 |
| H | -0.736923089216 | 3.219899607032 | -5.692923894098 |
| H | -0.441270385114 | 4.911959983230 | -5.231316641678 |
| C | -2.907459210075 | 2.652844768805 | 1.956513244124 |
| H | -2.432735026219 | 3.508079646164 | 2.445308350476 |
| H | -2.309528231916 | 1.763906853875 | 2.175694421305 |
| C | -4.325673655992 | 2.438208118534 | 2.649924974625 |
| H | -4.962514224358 | 3.309258548213 | 2.452026682725 |
| H | -4.810137836708 | 1.550901861851 | 2.221724943190 |
| O | 1.278684949699 | 3.831257179422 | -5.810708033187 |
| C | 1.502873858443 | 4.185837473981 | -7.244903286714 |
| H | 0.927451423821 | 3.527876243608 | -7.904460017686 |
| H | 1.237549780439 | 5.230949959597 | -7.435219928834 |
| H | 2.569483981831 | 4.032835902128 | -7.394138198080 |
| O | -4.116890846479 | 2.266972130843 | 4.084378446213 |
| C | -5.239334551105 | 2.058681788969 | 5.047990632174 |

| | | | |
|---|---|---|---|
| H | -5.923346646410 | 2.913677749382 | 5.042364182648 |
| H | -5.787891254064 | 1.139584332703 | 4.816814699498 |
| H | -4.748363670457 | 1.973242342920 | 6.014943485833 |

Cam_Int1-F3500

($E_F$ = -930.19722043 a.u.; $G_F$ = -929.81668258 a.u.)

0 1

| | | | |
|---|---|---|---|
| C | -1.473137592157 | 1.774175419490 | -2.576446993270 |
| C | -0.879673868771 | 2.715608822960 | -3.673021566664 |
| C | -3.308106029862 | 1.956925378027 | 1.002858858517 |
| C | -2.647312550180 | 1.450802212650 | -0.302211225196 |
| C | -1.137388867688 | 1.048455059740 | -0.159331110312 |
| C | -0.406816133155 | 1.359627116243 | -1.503614452514 |
| H | -0.413664063771 | 3.647700537213 | -3.360344159751 |
| H | -2.906307165126 | 2.849999467234 | 1.478471052933 |
| H | -3.213717573687 | 0.563845861363 | -0.601185116788 |
| H | -0.680219386012 | 1.619664387673 | 0.652697058129 |
| H | -1.053230187334 | -0.005014416486 | 0.116455359703 |
| H | 0.179293617934 | 0.508626347190 | -1.859698171380 |
| H | 0.297642049123 | 2.182034422786 | -1.358080295792 |
| C | -2.607833632315 | 2.432686474634 | -1.587922224699 |
| C | -3.983974877238 | 2.528417299441 | -2.248197730600 |
| H | -3.931683158107 | 3.123542337196 | -3.165236443687 |
| H | -4.384480810131 | 1.544948873579 | -2.502391395433 |
| H | -4.694290134767 | 3.007735137190 | -1.568123916526 |
| C | -2.169934932611 | 3.843246791499 | -1.155091445906 |

| | | | |
|---|---|---|---|
| H | -2.120873901260 | 4.512435217998 | -2.017497726090 |
| H | -2.895051132529 | 4.265049997417 | -0.454676794950 |
| H | -1.192297734430 | 3.848026668314 | -0.666608310831 |
| O | -0.383983971358 | 2.211240180501 | -4.892405810429 |
| O | -3.954475786847 | 1.075282397575 | 1.892103318137 |
| C | -2.011820382889 | 0.511356016409 | -3.272604690221 |
| H | -1.201970339855 | 0.018609564551 | -3.814379728517 |
| H | -2.419775126671 | -0.200415146224 | -2.551645450687 |
| H | -2.790654837168 | 0.753762000891 | -3.997688441847 |
| C | 0.273110055747 | 3.165171263620 | -5.817112627870 |
| H | 1.127126384484 | 3.617433162842 | -5.303243494318 |
| H | -0.441809779893 | 3.953560968215 | -6.068384685685 |
| C | 0.794237599252 | 2.513300454600 | -7.168384891708 |
| H | 1.512975151152 | 1.719403728785 | -6.929000191938 |
| H | -0.054633967709 | 2.063772957137 | -7.698651456172 |
| C | -4.431635290578 | 1.638973614345 | 3.179039297201 |
| H | -5.131858372578 | 2.450824980727 | 2.963421798204 |
| H | -3.572832886643 | 2.051619397261 | 3.717615302358 |
| C | -5.156949261438 | 0.594922965990 | 4.130598884487 |
| H | -6.028720124408 | 0.180262780865 | 3.609203208754 |
| H | -4.464917508478 | -0.226234745264 | 4.356029351838 |
| O | 1.420933403669 | 3.547219083497 | -7.981729297723 |
| C | 2.011169732836 | 3.260336241868 | -9.324835376771 |
| H | 2.829585707695 | 2.537463790909 | -9.245155977130 |
| H | 1.247526399386 | 2.887270798207 | -10.014979433910 |
| H | 2.387936379982 | 4.223586875472 | -9.661423267625 |

| | | | |
|---|---|---|---|
| O | -5.563512733734 | 1.276184460526 | 5.353112225801 |
| C | -6.281726447457 | 0.574083534335 | 6.460191788711 |
| H | -7.242743471881 | 0.180165605504 | 6.113869331174 |
| H | -5.670253118580 | -0.236455462379 | 6.869890489547 |
| H | -6.437020637005 | 1.347296181480 | 7.209315837864 |

Cam_TS1-F3000

($E_F$ = -929.78387267 a.u.; $G_F$ = -929.39490480 a.u.)

0 1

| | | | |
|---|---|---|---|
| C | -1.787140257251 | 1.031932398354 | -2.621702779311 |
| C | -1.161045123019 | 2.351238965685 | -2.095697255632 |
| C | -2.059428732484 | 2.120686108674 | -0.070572115027 |
| C | -2.567536016740 | 0.746272736185 | -0.455234922316 |
| C | -1.411767451889 | -0.276557805179 | -0.585253490835 |
| C | -0.933439640479 | -0.132474519753 | -2.050385698803 |
| H | -0.172105332730 | 2.214768874623 | -1.656228788187 |
| H | -1.089874673457 | 2.148480316085 | 0.430671205989 |
| H | -3.322604415260 | 0.432722835306 | 0.271948229213 |
| H | -0.618198361496 | -0.074799427723 | 0.138141631769 |
| H | -1.772637022596 | -1.286819777718 | -0.382491761258 |
| H | -1.119546139047 | -1.045350575534 | -2.620590918786 |
| H | 0.135725753454 | 0.075702957101 | -2.134554994558 |
| C | -3.159731375284 | 0.831437794788 | -1.889167385536 |
| C | -3.891320184572 | -0.467554410230 | -2.272192441919 |
| H | -4.268206293712 | -0.416170118236 | -3.296899094011 |
| H | -3.270745914514 | -1.360869509831 | -2.190502579914 |

| | | | |
|---|---|---|---|
| H | -4.755517233475 | -0.604918897146 | -1.615516528694 |
| C | -4.136617150260 | 1.997154158635 | -2.097281800314 |
| H | -4.478107317676 | 2.019960618866 | -3.136368423706 |
| H | -5.015300014997 | 1.873683660414 | -1.458243579918 |
| H | -3.687816864193 | 2.961639573350 | -1.863986206441 |
| O | -1.106030934532 | 3.380685626418 | -3.075915159659 |
| O | -3.041208572214 | 2.813100010501 | 0.725708582140 |
| C | -1.877386401668 | 0.992515793119 | -4.147829316371 |
| H | -0.884215776506 | 1.037671651250 | -4.600428864344 |
| H | -2.339654963344 | 0.057409151277 | -4.471688979342 |
| H | -2.461348863547 | 1.826272407545 | -4.536704111991 |
| C | 0.172149390610 | 3.524217408713 | -3.817660102563 |
| H | 0.751916981083 | 2.602248566229 | -3.727215052371 |
| H | 0.740610133486 | 4.337498855102 | -3.358903044921 |
| C | 0.014205646917 | 3.836562840715 | -5.358704551216 |
| H | -0.575973233402 | 3.044626881674 | -5.834193454102 |
| H | -0.518420338179 | 4.787673357182 | -5.485748142671 |
| C | -3.005660711207 | 2.435159021373 | 2.158045554738 |
| H | -2.288531343222 | 3.084049138278 | 2.670489408897 |
| H | -2.660750879914 | 1.401637765918 | 2.255871821623 |
| C | -4.388049524527 | 2.526781350857 | 2.910875367120 |
| H | -4.764448070301 | 3.557028702854 | 2.874888299346 |
| H | -5.115965463469 | 1.875079096594 | 2.409757260620 |
| O | 1.340233407028 | 3.907083648393 | -5.944495713283 |
| C | 1.522750213642 | 4.171655407460 | -7.388550273925 |
| H | 1.051830595707 | 3.389026528831 | -7.994027818531 |

| | | | |
|---|---|---|---|
| H | 1.112186216818 | 5.149419933064 | -7.664346057929 |
| H | 2.600715553671 | 4.162741354767 | -7.537602870651 |
| O | -4.174853623848 | 2.102741542098 | 4.282905203879 |
| C | -5.284180658081 | 2.066164189290 | 5.259711605171 |
| H | -5.712734987888 | 3.063806072836 | 5.406926118256 |
| H | -6.068438604560 | 1.371145560229 | 4.939152254267 |
| H | -4.827998943997 | 1.715140373029 | 6.183287648448 |

Cam-F3000

($E_F$ = -929.78942143 a.u.; $G_F$ = -929.39834475 a.u.)

0 1

| | | | |
|---|---|---|---|
| C | -1.856660201833 | 0.947443898013 | -2.584926295314 |
| C | -1.215381442169 | 2.209984416961 | -1.879461193308 |
| C | -1.866510083233 | 2.071890018886 | -0.284831265289 |
| C | -2.516810076455 | 0.685085017462 | -0.432687325541 |
| C | -1.429459955302 | -0.402213702781 | -0.609095042699 |
| C | -1.026280801237 | -0.267930871313 | -2.103656481833 |
| H | -0.172770852828 | 1.980182529762 | -1.644947922351 |
| H | -0.966930266992 | 2.041038164446 | 0.336531045888 |
| H | -3.189529361354 | 0.487720225014 | 0.403246811579 |
| H | -0.588755925273 | -0.238553805677 | 0.070085165457 |
| H | -1.827582889533 | -1.393564202685 | -0.385584592241 |
| H | -1.288903001340 | -1.161898774946 | -2.673720544966 |
| H | 0.044867340610 | -0.105368051222 | -2.246515348229 |
| C | -3.205232633877 | 0.751725671655 | -1.821462010965 |
| C | -3.973417074704 | -0.534565122511 | -2.165190255889 |

| | | | |
|---|---|---|---|
| H | -4.339422152276 | -0.504230108386 | -3.195286483637 |
| H | -3.385739079799 | -1.445533763556 | -2.049089009833 |
| H | -4.847645896536 | -0.622101703469 | -1.512790426687 |
| C | -4.170266163950 | 1.933465115413 | -2.015516759673 |
| H | -4.588690944336 | 1.902072028444 | -3.026060268162 |
| H | -4.999328181930 | 1.870480646002 | -1.305847713840 |
| H | -3.695391025021 | 2.903908265984 | -1.880220248534 |
| O | -1.174057822014 | 3.373684435425 | -2.749505142364 |
| O | -2.811747766673 | 2.950138829668 | 0.403155867970 |
| C | -1.961036989680 | 1.044709953889 | -4.098938986305 |
| H | -0.974162944472 | 1.062346144859 | -4.567750571922 |
| H | -2.489877638373 | 0.172218351800 | -4.491649441004 |
| H | -2.496631653951 | 1.943527038531 | -4.404054497562 |
| C | 0.060897513380 | 3.480542024893 | -3.558380161986 |
| H | 0.578258374731 | 2.518024140434 | -3.572130361196 |
| H | 0.715564431519 | 4.209035907113 | -3.070968991022 |
| C | -0.111501514419 | 3.922171723809 | -5.066234134781 |
| H | -0.792013982952 | 3.234703448264 | -5.580845524191 |
| H | -0.539791954250 | 4.931533479438 | -5.111489211072 |
| C | -2.846961205345 | 2.767959601290 | 1.865730890172 |
| H | -2.530984866263 | 3.715233848011 | 2.310491705003 |
| H | -2.127672862258 | 1.999848256192 | 2.164688952598 |
| C | -4.235496871626 | 2.389357225717 | 2.524932850543 |
| H | -4.988067748870 | 3.139678690204 | 2.250396247022 |
| H | -4.573674867915 | 1.411691089272 | 2.156418514554 |
| O | 1.203558083120 | 3.894482905801 | -5.683969089900 |

| | | | |
|---|---|---|---|
| C | 1.391147154582 | 4.252217653361 | -7.106216534309 |
| H | 0.825511833527 | 3.578337440307 | -7.759575277882 |
| H | 1.089385682068 | 5.288548003880 | -7.295118331186 |
| H | 2.458644272769 | 4.135615760938 | -7.282809543361 |
| O | -4.042961977145 | 2.353685454436 | 3.963821771838 |
| C | -5.155921956883 | 2.047161532469 | 4.887257181583 |
| H | -5.959072734327 | 2.786945808746 | 4.795577613119 |
| H | -5.555901640623 | 1.043645607990 | 4.703075886336 |
| H | -4.713531638897 | 2.096731682616 | 5.880324727555 |

Cam_Int1-F3000

($E_F$ = -929.99127034 a.u.; $G_F$ = -929.60949123 a.u.)

0 1

| | | | |
|---|---|---|---|
| C | -1.479451458139 | 1.779389435354 | -2.563294275566 |
| C | -0.906631389372 | 2.726241531896 | -3.649268361066 |
| C | -3.297579237195 | 1.968947002298 | 0.984546649733 |
| C | -2.638698416994 | 1.456160853475 | -0.305339933343 |
| C | -1.129102363419 | 1.056424254778 | -0.155720542711 |
| C | -0.402740845557 | 1.371628001861 | -1.498242243316 |
| H | -0.451277282015 | 3.664063118586 | -3.338142154995 |
| H | -2.899390923711 | 2.863988033852 | 1.459575512278 |
| H | -3.203016426062 | 0.565894182731 | -0.598755352265 |
| H | -0.675316551897 | 1.627501434432 | 0.658269107036 |
| H | -1.044457711897 | 0.002681457773 | 0.118683168650 |
| H | 0.186843445715 | 0.524974893359 | -1.858591687161 |
| H | 0.296670829527 | 2.198791460286 | -1.355482824578 |

| | | | |
|---|---|---|---|
| C | -2.609231559124 | 2.432955562198 | -1.583201263681 |
| C | -3.987691173014 | 2.521431933413 | -2.241253597752 |
| H | -3.939232354681 | 3.114117692918 | -3.160017952438 |
| H | -4.385082508318 | 1.535774247943 | -2.491757253274 |
| H | -4.698254448673 | 2.999993623202 | -1.561035721245 |
| C | -2.180584369100 | 3.848208639978 | -1.152935109769 |
| H | -2.140457910683 | 4.517451981145 | -2.015515090372 |
| H | -2.906936305753 | 4.265034020583 | -0.451125488971 |
| H | -1.201422708019 | 3.861414921702 | -0.667578652775 |
| O | -0.401903136551 | 2.220092945101 | -4.856236738161 |
| O | -3.928536695883 | 1.081706828451 | 1.868619404788 |
| C | -2.009896097049 | 0.511662891498 | -3.258519255710 |
| H | -1.197042629073 | 0.024284630252 | -3.800320170328 |
| H | -2.411841801362 | -0.202456034198 | -2.536636684001 |
| H | -2.791086049782 | 0.747798341166 | -3.983072699751 |
| C | 0.229696217629 | 3.181338974776 | -5.774174873932 |
| H | 1.073662137571 | 3.657121984517 | -5.263871348887 |
| H | -0.500554669648 | 3.953480064851 | -6.033648855302 |
| C | 0.759461050537 | 2.526225023012 | -7.104966651232 |
| H | 1.494215150694 | 1.748520602693 | -6.858810854408 |
| H | -0.078328265559 | 2.054373973536 | -7.634471798879 |
| C | -4.408591717974 | 1.648434567085 | 3.140831873753 |
| H | -5.119675440754 | 2.450763206731 | 2.922858924659 |
| H | -3.557168671418 | 2.075314331658 | 3.681049633048 |
| C | -5.113416271075 | 0.599471806216 | 4.080450559075 |
| H | -5.980863679330 | 0.171788878162 | 3.561301433815 |

| | | | |
|---|---|---|---|
| H | -4.411853850634 | -0.213570414947 | 4.308392909724 |
| O | 1.361694733761 | 3.566212944558 | -7.915532495298 |
| C | 1.947914482678 | 3.259164158013 | -9.238506635999 |
| H | 2.776503243036 | 2.548226462172 | -9.146055703467 |
| H | 1.190314577889 | 2.857507452368 | -9.920555299770 |
| H | 2.314341917780 | 4.215156583219 | -9.607170756753 |
| O | -5.522554678239 | 1.282287263207 | 5.292257033838 |
| C | -6.225336214953 | 0.561071043585 | 6.375729133145 |
| H | -7.178367169259 | 0.152120123374 | 6.022192816481 |
| H | -5.603472746352 | -0.245924172107 | 6.778971216434 |
| H | -6.402142132069 | 1.315008431399 | 7.140231194241 |

Cam_TS1-F2500

($E_F$ = -929.61668918 a.u.; $G_F$ = -929.22889556 a.u.)

0 1

| | | | |
|---|---|---|---|
| C | -1.768102660351 | 1.026261026725 | -2.631854463160 |
| C | -1.125408508509 | 2.350390589644 | -2.175354806327 |
| C | -2.104511324255 | 2.096519233059 | 0.000947143912 |
| C | -2.601261639653 | 0.749048714426 | -0.459160332327 |
| C | -1.447931229152 | -0.278682030319 | -0.571391693010 |
| C | -0.929559966089 | -0.131319432365 | -2.020601261552 |
| H | -0.152101007898 | 2.246017498169 | -1.696499630432 |
| H | -1.119000620032 | 2.124475719110 | 0.468973958284 |
| H | -3.387204645028 | 0.418186682592 | 0.228450354241 |
| H | -0.672166307056 | -0.089326189420 | 0.174111046456 |
| H | -1.821573412048 | -1.287681135883 | -0.385975867355 |

| | | | |
|---|---|---|---|
| H | -1.094214147239 | -1.046429027592 | -2.593716670373 |
| H | 0.141061575764 | 0.079639465314 | -2.073355977018 |
| C | -3.154418762141 | 0.854378368732 | -1.910820462272 |
| C | -3.899126033580 | -0.433161245146 | -2.310635150338 |
| H | -4.270550599150 | -0.367778029430 | -3.336381404234 |
| H | -3.287086018633 | -1.333200925165 | -2.235160049147 |
| H | -4.767652703204 | -0.568346270818 | -1.659417512599 |
| C | -4.108806158076 | 2.035462540462 | -2.129587713440 |
| H | -4.426400291520 | 2.075940916871 | -3.175694565023 |
| H | -5.003907765276 | 1.917301705901 | -1.512379910137 |
| H | -3.647558979095 | 2.988233304286 | -1.873353908280 |
| O | -1.096216941094 | 3.347600470677 | -3.163565402447 |
| O | -3.071414933703 | 2.768610319490 | 0.804490693285 |
| C | -1.845082165576 | 0.934071421327 | -4.159311396357 |
| H | -0.846426795102 | 0.983322130114 | -4.599903927089 |
| H | -2.288168664867 | -0.017935445970 | -4.458723789139 |
| H | -2.437249011813 | 1.745808615867 | -4.581169107860 |
| C | 0.185040068651 | 3.585254103734 | -3.854476818601 |
| H | 0.847767396711 | 2.729525787727 | -3.701746153100 |
| H | 0.653698151564 | 4.471807388515 | -3.419378731418 |
| C | 0.030743364023 | 3.806263721781 | -5.399849234900 |
| H | -0.458551132293 | 2.933840453802 | -5.849085671889 |
| H | -0.599275097772 | 4.686403110388 | -5.583920850810 |
| C | -3.021612274376 | 2.372405656716 | 2.222514574650 |
| H | -2.257400891642 | 2.969185740919 | 2.731471151410 |
| H | -2.737890249293 | 1.318160221391 | 2.298501221027 |

| | | | |
|---|---|---|---|
| C | -4.379520662421 | 2.535655291377 | 2.982187768128 |
| H | -4.691091297027 | 3.588421885173 | 2.977106555245 |
| H | -5.153271743947 | 1.944588778222 | 2.473754290216 |
| O | 1.350771689722 | 3.992721022214 | -5.954151048157 |
| C | 1.508862542439 | 4.189430826667 | -7.398320344049 |
| H | 1.133902332977 | 3.324530604861 | -7.958225623704 |
| H | 0.989320444014 | 5.094714123423 | -7.733568188130 |
| H | 2.580366713109 | 4.297208166064 | -7.556848584374 |
| O | -4.174016431389 | 2.064334262106 | 4.332509415529 |
| C | -5.282346380152 | 2.071587665076 | 5.290281879901 |
| H | -5.653210357285 | 3.089375881141 | 5.460227893723 |
| H | -6.106975692320 | 1.435520770791 | 4.946796175978 |
| H | -4.865484296007 | 1.670963739568 | 6.212758039512 |

Cam_Int1-F2500

($E_F$ = -929.78756119 a.u.; $G_F$ = -929.40479269 a.u.)

0 1

| | | | |
|---|---|---|---|
| C | -1.483986500494 | 1.784607492901 | -2.551238754895 |
| C | -0.930838539765 | 2.736851439237 | -3.627268807264 |
| C | -3.286749914409 | 1.980371114879 | 0.966862215220 |
| C | -2.629295540506 | 1.461334085041 | -0.308880284938 |
| C | -1.119883167021 | 1.065013047936 | -0.152633223269 |
| C | -0.397443487741 | 1.384560203769 | -1.493359224119 |
| H | -0.486208707802 | 3.680135992778 | -3.317293227116 |
| H | -2.891463479823 | 2.876704537066 | 1.441942320448 |
| H | -3.191258541959 | 0.567690722919 | -0.596750846733 |

| | | | |
|---|---|---|---|
| H | -0.669764699027 | 1.636121090352 | 0.663341802528 |
| H | -1.034163762877 | 0.011025849346 | 0.120327441821 |
| H | 0.196310850675 | 0.542579280006 | -1.857475525955 |
| H | 0.296284683021 | 2.216900668378 | -1.353094999113 |
| C | -2.610039325910 | 2.433108204997 | -1.579553131523 |
| C | -3.990594521546 | 2.513108628812 | -2.235753527568 |
| H | -3.946097121439 | 3.103101049230 | -3.156389127494 |
| H | -4.384076157118 | 1.524931093706 | -2.482396605142 |
| H | -4.701935647042 | 2.990618499637 | -1.555727691660 |
| C | -2.191625479478 | 3.853240950147 | -1.152017265475 |
| H | -2.160570080612 | 4.522312116758 | -2.014867315176 |
| H | -2.919583685397 | 4.264702811728 | -0.448997655654 |
| H | -1.211149939782 | 3.875509075617 | -0.669557258578 |
| O | -0.416456933088 | 2.229377410997 | -4.822082265502 |
| O | -3.903744973090 | 1.087670850323 | 1.845298304571 |
| C | -2.005540196391 | 0.511898526467 | -3.245544481899 |
| H | -1.189419748517 | 0.030069310639 | -3.787085124491 |
| H | -2.401527658993 | -0.204551758060 | -2.522791237199 |
| H | -2.788889663762 | 0.741457593957 | -3.969821487581 |
| C | 0.191393951274 | 3.197556724940 | -5.733578351133 |
| H | 1.025346389882 | 3.694876102662 | -5.226357083164 |
| H | -0.553043461881 | 3.953994897418 | -6.000590810235 |
| C | 0.729623931734 | 2.539117817416 | -7.045182040651 |
| H | 1.478412617034 | 1.776528745691 | -6.791975185013 |
| H | -0.097593535008 | 2.046951039177 | -7.573763528000 |
| C | -4.387265961364 | 1.656435287769 | 3.104159302330 |

| | | | |
|---|---|---|---|
| H | -5.108459476000 | 2.449687368485 | 2.884151318349 |
| H | -3.543116173474 | 2.096047303277 | 3.646515465131 |
| C | -5.073158270334 | 0.601314236729 | 4.030973807464 |
| H | -5.935567446501 | 0.161778112888 | 3.512346439644 |
| H | -4.362243375264 | -0.203797579542 | 4.260418635767 |
| O | 1.310124135137 | 3.583285363251 | -7.854032110756 |
| C | 1.893068403675 | 3.254553229738 | -9.157890032767 |
| H | 2.729054315561 | 2.553031999450 | -9.052068590702 |
| H | 1.140580910321 | 2.827532842542 | -9.831357771493 |
| H | 2.252514814961 | 4.201389529356 | -9.557012145068 |
| O | -5.486638505752 | 1.282781824937 | 5.233898697130 |
| C | -6.174848195738 | 0.539672906421 | 6.293045710052 |
| H | -7.118858699251 | 0.116258476633 | 5.930111676839 |
| H | -5.542008729928 | -0.263719265572 | 6.688389012092 |
| H | -6.373451125369 | 1.272430958576 | 7.073208488023 |

Cam-F2500

($E_F$ = -929.63223347 a.u.; $G_F$ = -929.23976291 a.u.)

0 1

| | | | |
|---|---|---|---|
| C | -1.824607515276 | 0.874892560160 | -2.564488726035 |
| C | -1.199955639735 | 2.126486572057 | -1.833695704025 |
| C | -1.885110891100 | 1.992360600415 | -0.299908384565 |
| C | -2.638404315922 | 0.655741005317 | -0.463166668638 |
| C | -1.611990005495 | -0.495504996514 | -0.557876931779 |
| C | -1.072981641453 | -0.362716870723 | -2.009715983915 |
| H | -0.149032951034 | 1.914086266660 | -1.618822528451 |

| | | | |
|---|---|---|---|
| H | -1.021711319906 | 1.878911947597 | 0.364761550421 |
| H | -3.372651767862 | 0.522257927554 | 0.330859476955 |
| H | -0.826458851849 | -0.395679726169 | 0.195605115669 |
| H | -2.088783926782 | -1.463099265379 | -0.391732779244 |
| H | -1.302957186328 | -1.248255302143 | -2.606295005874 |
| H | 0.010519624048 | -0.225915490067 | -2.053080874911 |
| C | -3.229934891237 | 0.742852870223 | -1.893740094907 |
| C | -4.026717398141 | -0.508859215484 | -2.294372734802 |
| H | -4.339615986481 | -0.447704356277 | -3.340504693123 |
| H | -3.477910363718 | -1.442468113106 | -2.168140181843 |
| H | -4.934798577714 | -0.575596573453 | -1.687232963184 |
| C | -4.130278109730 | 1.965220918747 | -2.140648531296 |
| H | -4.476364105414 | 1.965198083352 | -3.178648251137 |
| H | -5.011235386933 | 1.923152754062 | -1.493801628712 |
| H | -3.629678521983 | 2.913573632451 | -1.955747553635 |
| O | -1.208874298254 | 3.317081046616 | -2.657679736531 |
| O | -2.748230426434 | 2.977620404915 | 0.310104951054 |
| C | -1.816248989549 | 0.948725574715 | -4.082289708387 |
| H | -0.797248683648 | 0.975258360427 | -4.477396823212 |
| H | -2.304475345842 | 0.064756212864 | -4.501057151253 |
| H | -2.341863808948 | 1.834920172945 | -4.437304621564 |
| C | 0.016333290805 | 3.520396139611 | -3.442017662669 |
| H | 0.614151446280 | 2.605105504637 | -3.446355990252 |
| H | 0.603832263281 | 4.303297030215 | -2.952512364663 |
| C | -0.182983440821 | 3.935696142712 | -4.942820330162 |
| H | -0.805484446384 | 3.197541787397 | -5.459950763695 |

| | | | |
|---|---|---|---|
| H | -0.684505419907 | 4.910690842560 | -5.000089789475 |
| C | -2.814948091869 | 2.952783332875 | 1.768808042107 |
| H | -2.844462749973 | 4.000361162249 | 2.075508286756 |
| H | -1.900688325235 | 2.507572679094 | 2.176736389958 |
| C | -4.048425099064 | 2.254905254889 | 2.461527366538 |
| H | -4.978438448231 | 2.648277484250 | 2.029423246935 |
| H | -4.025528683043 | 1.169145216687 | 2.301702254199 |
| O | 1.131221699319 | 3.999108960485 | -5.543918147145 |
| C | 1.268344050057 | 4.331148888250 | -6.963992877826 |
| H | 0.752649685553 | 3.596343282727 | -7.593958211217 |
| H | 0.876257728944 | 5.332785567562 | -7.177088011379 |
| H | 2.338333655694 | 4.302973520153 | -7.162960098169 |
| O | -3.968410211571 | 2.551087622238 | 3.873539378261 |
| C | -4.976556206639 | 2.017065347864 | 4.792626463930 |
| H | -5.977586229793 | 2.387233323300 | 4.541066588145 |
| H | -4.980186937532 | 0.920415740093 | 4.782923520850 |
| H | -4.685663391238 | 2.377287457423 | 5.777939018180 |

Cam_TS1-F2000

($E_F$ = -929.45027302 a.u.; $G_F$ = -929.06281588 a.u.)

0 1

| | | | |
|---|---|---|---|
| C | -1.799579650618 | 1.000658272109 | -2.666252445279 |
| C | -1.108537028115 | 2.306997079283 | -2.245649490031 |
| C | -2.075351839500 | 2.049742689346 | 0.017143645732 |
| C | -2.604536547437 | 0.726839342733 | -0.469699112978 |
| C | -1.475979327955 | -0.323760910021 | -0.613071336156 |

| | | | |
|---|---|---|---|
| C | -0.976518176981 | -0.175064233891 | -2.068649625819 |
| H | -0.123097525505 | 2.197632764061 | -1.794173144041 |
| H | -1.079632090505 | 2.055401749161 | 0.464259556966 |
| H | -3.391279554899 | 0.396564926742 | 0.218546886048 |
| H | -0.684076013570 | -0.158941798195 | 0.121297367151 |
| H | -1.867794238791 | -1.326618912389 | -0.431679688639 |
| H | -1.166206167361 | -1.083273373421 | -2.644901198477 |
| H | 0.097428039017 | 0.014929937656 | -2.135257931824 |
| C | -3.174498301320 | 0.867692078553 | -1.913311090770 |
| C | -3.966946493443 | -0.392372658083 | -2.310791660557 |
| H | -4.361849680866 | -0.304079206845 | -3.325944730919 |
| H | -3.379120683179 | -1.310480960514 | -2.260833390473 |
| H | -4.822356212917 | -0.510644296605 | -1.639290763901 |
| C | -4.093516159525 | 2.080948305360 | -2.102918120441 |
| H | -4.424425789239 | 2.146238279388 | -3.143714587237 |
| H | -4.982884856006 | 1.983859902639 | -1.473933803264 |
| H | -3.596781813097 | 3.014043797792 | -1.840500750488 |
| O | -1.135690538976 | 3.314614569917 | -3.200095933110 |
| O | -3.014533018019 | 2.743049402366 | 0.821390652314 |
| C | -1.913145408506 | 0.897540293515 | -4.191591842110 |
| H | -0.921693374784 | 0.908223689712 | -4.650089541133 |
| H | -2.394660789704 | -0.040603128948 | -4.474351627778 |
| H | -2.484007186198 | 1.726301287779 | -4.610142269222 |
| C | 0.110511426677 | 3.780604283722 | -3.804373309752 |
| H | 0.954113726040 | 3.207282547445 | -3.409660254656 |
| H | 0.243606475641 | 4.831110742671 | -3.535893853408 |

| | | | |
|---|---|---|---|
| C | 0.098022442630 | 3.667775537920 | -5.360794835312 |
| H | 0.028068130035 | 2.614513726586 | -5.658889140205 |
| H | -0.783930683296 | 4.193022129485 | -5.752473661624 |
| C | -2.960676563179 | 2.352043886585 | 2.230505021650 |
| H | -2.187732711812 | 2.938559916424 | 2.739372934964 |
| H | -2.692948724875 | 1.293587621551 | 2.311236460500 |
| C | -4.312608043546 | 2.539997544985 | 2.972841977660 |
| H | -4.610130410671 | 3.597249561299 | 2.962228516610 |
| H | -5.092892346788 | 1.958295850814 | 2.462721472255 |
| O | 1.310460561162 | 4.252642256411 | -5.861134708115 |
| C | 1.540004736852 | 4.219676005056 | -7.297250522349 |
| H | 1.579426731829 | 3.188084461834 | -7.668949536424 |
| H | 0.758512980224 | 4.768872616670 | -7.836848367252 |
| H | 2.503074486836 | 4.703296967315 | -7.453364121790 |
| O | -4.117443032827 | 2.071874092582 | 4.319221531363 |
| C | -5.241693512184 | 2.095174864362 | 5.238602243150 |
| H | -5.609475731970 | 3.117227119725 | 5.393551995456 |
| H | -6.065191083591 | 1.466598351233 | 4.876822659003 |
| H | -4.862021942274 | 1.696379218457 | 6.178229387166 |

Cam_Int1-F2000

($E_F$ = -929.58595229 a.u.; $G_F$ = -929.20247217 a.u.)

0 1

| | | | |
|---|---|---|---|
| C | -1.489687581727 | 1.799620812996 | -2.542446204988 |
| C | -0.957017412393 | 2.760581573137 | -3.606857007219 |
| C | -3.274465798592 | 1.989753175337 | 0.950086553038 |

| | | | |
|---|---|---|---|
| C | -2.619131976099 | 1.469025366510 | -0.314707497890 |
| C | -1.109417779827 | 1.077123897105 | -0.154823193098 |
| C | -0.392409251627 | 1.405417508478 | -1.493656404084 |
| H | -0.523360180422 | 3.708435963000 | -3.295445136079 |
| H | -2.881421519087 | 2.885462060087 | 1.428253945686 |
| H | -3.178357954160 | 0.572511206107 | -0.599089561322 |
| H | -0.662539858773 | 1.646330134573 | 0.664231619433 |
| H | -1.021248367997 | 0.022206262002 | 0.113578485626 |
| H | 0.205947149011 | 0.569678760960 | -1.864266429168 |
| H | 0.294958677098 | 2.243112390406 | -1.354035878308 |
| C | -2.612178711040 | 2.439577171262 | -1.575913836243 |
| C | -3.995443995470 | 2.511348787009 | -2.228678114289 |
| H | -3.956437426056 | 3.101183745986 | -3.149601916287 |
| H | -4.384148851272 | 1.520918597988 | -2.473778457845 |
| H | -4.707416225734 | 2.985182131400 | -1.546848148012 |
| C | -2.204982776573 | 3.863626710795 | -1.147493665097 |
| H | -2.184596328023 | 4.534929596960 | -2.008696237424 |
| H | -2.934100319017 | 4.266891688307 | -0.441209733033 |
| H | -1.222767154667 | 3.894526765931 | -0.668965422212 |
| O | -0.433289369809 | 2.255738754059 | -4.791596273313 |
| O | -3.877436634548 | 1.088556116523 | 1.820127882580 |
| C | -2.002043698767 | 0.523742674346 | -3.239078731223 |
| H | -1.182861737015 | 0.049582012061 | -3.782471733883 |
| H | -2.391006738650 | -0.197332615212 | -2.517207086414 |
| H | -2.788274574775 | 0.748311309697 | -3.961743406558 |
| C | 0.150900077121 | 3.233084670498 | -5.695019391310 |

| | | | |
|---|---|---|---|
| H | 0.975065454383 | 3.749488276536 | -5.189967860443 |
| H | -0.607383552692 | 3.974519872067 | -5.966574336896 |
| C | 0.696516222052 | 2.574389380169 | -6.990442816138 |
| H | 1.458227370180 | 1.825770020867 | -6.732242823668 |
| H | -0.120885444472 | 2.064282348237 | -7.518160697904 |
| C | -4.364544365070 | 1.653877238518 | 3.069024037510 |
| H | -5.096498043963 | 2.438111211172 | 2.850436723392 |
| H | -3.527746528375 | 2.104310241854 | 3.614723210894 |
| C | -5.030372858321 | 0.587363256668 | 3.979046080266 |
| H | -5.886669169172 | 0.137380637228 | 3.458364106327 |
| H | -4.308734699191 | -0.209552715646 | 4.205683867200 |
| O | 1.256421392815 | 3.622992654127 | -7.796964629322 |
| C | 1.835479577092 | 3.273981583683 | -9.083590404110 |
| H | 2.677074501618 | 2.579991606421 | -8.965964570647 |
| H | 1.086762569691 | 2.825112949359 | -9.748126907876 |
| H | 2.189875058806 | 4.211000982703 | -9.510481310351 |
| O | -5.450094520443 | 1.259707786547 | 5.177466783522 |
| C | -6.121560476020 | 0.487051923237 | 6.209098972468 |
| H | -7.054995419288 | 0.049194240457 | 5.834044235054 |
| H | -5.475134492603 | -0.312673165893 | 6.591634513243 |
| H | -6.342932242640 | 1.193170072894 | 7.008004997682 |

Cam-F2000

($E_F$ = -929.47876953 a.u.; $G_F$ = -929.08504818 a.u.)

0 1

| | | | |
|---|---|---|---|
| C | -1.810237173614 | 0.827807612085 | -2.554364235501 |

| | | | |
|---|---|---|---|
| C | -1.189471622765 | 2.061109963358 | -1.799965003383 |
| C | -1.902671424677 | 1.933631000872 | -0.314726951288 |
| C | -2.743603515678 | 0.648184415488 | -0.499891547023 |
| C | -1.778415675664 | -0.556011560412 | -0.536836606955 |
| C | -1.133699811333 | -0.430857686951 | -1.946722552724 |
| H | -0.134295554291 | 1.855226816665 | -1.597728356964 |
| H | -1.076102100738 | 1.753458942403 | 0.383123754185 |
| H | -3.524447528941 | 0.568782331578 | 0.255498783265 |
| H | -1.043147598616 | -0.509067649662 | 0.270517293008 |
| H | -2.316222187733 | -1.498665909145 | -0.421996549764 |
| H | -1.340601990228 | -1.309184957562 | -2.561880804476 |
| H | -0.046711839705 | -0.321272583945 | -1.909936845737 |
| C | -3.254761492388 | 0.752763958017 | -1.959778766926 |
| C | -4.071309023453 | -0.469267969321 | -2.410413446605 |
| H | -4.342540595786 | -0.379313110958 | -3.466104430682 |
| H | -3.553207366758 | -1.420202817584 | -2.284107089255 |
| H | -5.003031417409 | -0.521977645377 | -1.838742970152 |
| C | -4.103358498926 | 2.004235252508 | -2.242112063452 |
| H | -4.373584011492 | 2.039235413200 | -3.301817571591 |
| H | -5.030730588336 | 1.963996114033 | -1.662747059066 |
| H | -3.598485961935 | 2.934965438368 | -1.994661611512 |
| O | -1.230302346175 | 3.275140865143 | -2.577268879851 |
| O | -2.690451955677 | 2.997190498529 | 0.237254209373 |
| C | -1.710606595027 | 0.886478366373 | -4.068891924207 |
| H | -0.669547631345 | 0.918272460953 | -4.402539666433 |
| H | -2.168546167047 | -0.003026363514 | -4.509568182706 |

| | | | |
|---|---|---|---|
| H | -2.220767219223 | 1.765536913026 | -4.461775859460 |
| C | -0.014715744334 | 3.552268522188 | -3.333387006886 |
| H | 0.630117983813 | 2.669225103659 | -3.351687363257 |
| H | 0.530032926117 | 4.355114165587 | -2.826075182950 |
| C | -0.236936927931 | 3.978327323235 | -4.816681761477 |
| H | -0.823573285430 | 3.219429496646 | -5.345726657812 |
| H | -0.784414835229 | 4.929457359620 | -4.861687851954 |
| C | -2.763266432615 | 3.053129996849 | 1.683117011782 |
| H | -2.966461190494 | 4.099952203867 | 1.915460343257 |
| H | -1.790176657614 | 2.792252719962 | 2.117026131693 |
| C | -3.868086188841 | 2.214105333854 | 2.412853129486 |
| H | -4.838928314857 | 2.390254461234 | 1.928706629374 |
| H | -3.646387605360 | 1.139757345109 | 2.361513739971 |
| O | 1.068760867066 | 4.109640534113 | -5.410267114229 |
| C | 1.158158622679 | 4.437425479498 | -6.822670702545 |
| H | 0.670501482522 | 3.672372759863 | -7.440105578074 |
| H | 0.705815052520 | 5.414221265940 | -7.035328109188 |
| H | 2.222440630678 | 4.469422183195 | -7.051632189542 |
| O | -3.886481270311 | 2.649324132508 | 3.783519704951 |
| C | -4.800463308148 | 1.997569722410 | 4.706189363426 |
| H | -5.844015016324 | 2.134813084794 | 4.396267774007 |
| H | -4.586298437681 | 0.924278138369 | 4.787084087644 |
| H | -4.638503681500 | 2.477140770639 | 5.670475962914 |

Cam_TS1-F1500

($E_F$ = -929.28538410 a.u.; $G_F$ = -928.89760082 a.u.)

0 1

| | | | |
|---|---|---|---|
| C | -1.817826213712 | 0.991956800967 | -2.708014913435 |
| C | -1.097829431719 | 2.284604644643 | -2.318316016341 |
| C | -2.057966188073 | 1.997884533878 | 0.050418820690 |
| C | -2.583914328983 | 0.695496910110 | -0.483201063346 |
| C | -1.454887107918 | -0.349019137748 | -0.668464877305 |
| C | -0.993091593251 | -0.194208594328 | -2.136141554903 |
| H | -0.102545594080 | 2.184562492864 | -1.887457259777 |
| H | -1.052932855820 | 2.008789410690 | 0.475416576716 |
| H | -3.364127930776 | 0.338198056396 | 0.200452399666 |
| H | -0.643054041984 | -0.184820332285 | 0.044105896242 |
| H | -1.836512811383 | -1.354715349501 | -0.481005972148 |
| H | -1.206702223567 | -1.097059639398 | -2.712367291339 |
| H | 0.080936723455 | -0.015333472514 | -2.228881231045 |
| C | -3.175300791924 | 0.872816683952 | -1.916233463443 |
| C | -3.997578573855 | -0.368600070028 | -2.313632818445 |
| H | -4.411412910465 | -0.262189491617 | -3.319361377972 |
| H | -3.423414814797 | -1.296568738213 | -2.285388362875 |
| H | -4.840924202893 | -0.480951614454 | -1.626113194719 |
| C | -4.076361312780 | 2.104282133361 | -2.066441150234 |
| H | -4.429641311447 | 2.192449229877 | -3.098187279270 |
| H | -4.952404264016 | 2.013975555763 | -1.418208956070 |
| H | -3.555445776244 | 3.023208971679 | -1.800819297970 |
| O | -1.190135530523 | 3.313921607601 | -3.229640633660 |
| O | -2.989560234229 | 2.690191361097 | 0.846705012539 |
| C | -1.981166280727 | 0.893077370913 | -4.230979703396 |

| | | | |
|---|---|---|---|
| H | -1.005960988981 | 0.909744717065 | -4.722790608181 |
| H | -2.470071053069 | -0.044189754117 | -4.503073132008 |
| H | -2.567407939059 | 1.722897037506 | -4.625965980880 |
| C | 0.009457126353 | 3.908353112883 | -3.787367342281 |
| H | 0.893029604042 | 3.567541822463 | -3.239291949155 |
| H | -0.078442247620 | 4.990499205712 | -3.673383072694 |
| C | 0.167119212033 | 3.585885423604 | -5.296221660127 |
| H | 0.369333388124 | 2.514995640382 | -5.434196768595 |
| H | -0.773544280166 | 3.824142724447 | -5.812165148194 |
| C | -2.925584393889 | 2.318561090474 | 2.251589662760 |
| H | -2.140183093859 | 2.897826893371 | 2.750718379739 |
| H | -2.677083657867 | 1.256087973222 | 2.345612763142 |
| C | -4.269379797354 | 2.544265461746 | 2.976814376222 |
| H | -4.547719476450 | 3.606888973260 | 2.948744592006 |
| H | -5.058471411894 | 1.970015837980 | 2.471047358611 |
| O | 1.247718631726 | 4.370264046642 | -5.806758449764 |
| C | 1.557179655992 | 4.207326101521 | -7.207999667073 |
| H | 1.850027969318 | 3.173598464163 | -7.433825487679 |
| H | 0.702453188607 | 4.483603981538 | -7.839011396417 |
| H | 2.392678665990 | 4.875451741404 | -7.413487546661 |
| O | -4.087332475719 | 2.093403659220 | 4.324666528776 |
| C | -5.230264819039 | 2.141166478801 | 5.202039434027 |
| H | -5.595697737612 | 3.168657525067 | 5.329267486578 |
| H | -6.049854421323 | 1.514798161513 | 4.825609265154 |
| H | -4.890413563645 | 1.755572548755 | 6.162681986981 |

Cam-F1500

($E_F$ = -929.32878687 a.u.; $G_F$ = -928.93475824 a.u.)

0 1

| | | | |
|---|---|---|---|
| C | -1.905812273574 | 0.800365679167 | -2.619634570981 |
| C | -1.228113931543 | 2.009221939878 | -1.875822189473 |
| C | -1.841029921827 | 1.864127503426 | -0.372435991944 |
| C | -2.713957560179 | 0.595162900803 | -0.512668857272 |
| C | -1.760846253242 | -0.614677249305 | -0.626791549596 |
| C | -1.203092525643 | -0.471949535191 | -2.072301712225 |
| H | -0.153581713417 | 1.815281726269 | -1.787331003171 |
| H | -0.993466879164 | 1.674946733192 | 0.298755314546 |
| H | -3.446627106665 | 0.509112597462 | 0.288299029755 |
| H | -0.976047190265 | -0.583420920465 | 0.133392302651 |
| H | -2.295868377170 | -1.556085775165 | -0.491810407703 |
| H | -1.452595066219 | -1.338953541290 | -2.687684830530 |
| H | -0.115006110661 | -0.369057968365 | -2.100594813947 |
| C | -3.312809302650 | 0.720143168537 | -1.938310792541 |
| C | -4.155721709237 | -0.499205692782 | -2.349310717833 |
| H | -4.504821259450 | -0.393524117486 | -3.380300295194 |
| H | -3.628787221708 | -1.450783849356 | -2.276921636741 |
| H | -5.042919371159 | -0.563812099685 | -1.711813235470 |
| C | -4.182586962225 | 1.969257448288 | -2.161017547551 |
| H | -4.505529615715 | 2.012657394544 | -3.205768911012 |
| H | -5.080336292828 | 1.913379476011 | -1.537445973310 |
| H | -3.672892113726 | 2.899746130476 | -1.927700950105 |
| O | -1.382116077550 | 3.258722528133 | -2.549859879610 |

| | | | |
|---|---|---|---|
| O | -2.571373154064 | 2.970011505310 | 0.161068753489 |
| C | -1.895354258743 | 0.882073266190 | -4.134630524626 |
| H | -0.879694213878 | 0.792193059448 | -4.527943613201 |
| H | -2.479822709495 | 0.061678392234 | -4.559562641253 |
| H | -2.316702676070 | 1.824603668256 | -4.484902643473 |
| C | -0.221962579078 | 3.851811965379 | -3.167722689786 |
| H | 0.692873520642 | 3.514795686783 | -2.666677041993 |
| H | -0.313078904167 | 4.929218198258 | -3.012180264209 |
| C | -0.076828508504 | 3.619253825169 | -4.698304558242 |
| H | 0.180282587768 | 2.574847314621 | -4.910618959624 |
| H | -1.033745333956 | 3.843083270179 | -5.190698142495 |
| C | -2.626202431501 | 3.062504895415 | 1.596717670387 |
| H | -2.807566051571 | 4.117409591199 | 1.811208252026 |
| H | -1.656215921501 | 2.790121768686 | 2.031979798177 |
| C | -3.742903729679 | 2.259157295638 | 2.326333638753 |
| H | -4.705302760364 | 2.434460183787 | 1.824391188694 |
| H | -3.534950080885 | 1.180978967499 | 2.304733631832 |
| O | 0.958402915262 | 4.484494039737 | -5.177678844094 |
| C | 1.287739390950 | 4.371817879472 | -6.578331102045 |
| H | 1.642515877833 | 3.362261307579 | -6.824867081494 |
| H | 0.423575066826 | 4.610969404286 | -7.211863786072 |
| H | 2.084065808494 | 5.092041712862 | -6.762944715774 |
| O | -3.773303985970 | 2.725058567567 | 3.680330213484 |
| C | -4.701853661207 | 2.078396712455 | 4.574910135032 |
| H | -5.738181354552 | 2.205098991740 | 4.235342446874 |
| H | -4.485369643285 | 1.005944165247 | 4.668846095691 |

| | | | |
|---|---|---|---|
| H | -4.575700515429 | 2.560475542413 | 5.543717741774 |

Cam_Int1-F1500

($E_F$ = -929.38634125 a.u.; $G_F$ = -929.00240061 a.u.)

0 1

| | | | |
|---|---|---|---|
| C | -1.503899880917 | 1.805616759888 | -2.539989446161 |
| C | -0.976830341708 | 2.772741329318 | -3.588367629279 |
| C | -3.252694627764 | 1.997200448966 | 0.937654279188 |
| C | -2.614797504957 | 1.472419090634 | -0.323966172407 |
| C | -1.111758694014 | 1.055233803680 | -0.170281723301 |
| C | -0.399522578248 | 1.388673387876 | -1.507847431562 |
| H | -0.535538866365 | 3.714848614208 | -3.270461295099 |
| H | -2.841256950251 | 2.879323251078 | 1.425576179708 |
| H | -3.189597466693 | 0.586095102361 | -0.609203495948 |
| H | -0.653795210635 | 1.607874445387 | 0.653966171170 |
| H | -1.039941563782 | -0.004058939744 | 0.085321892259 |
| H | 0.186007493351 | 0.550795425662 | -1.893527814445 |
| H | 0.297855402518 | 2.217575303608 | -1.365349952106 |
| C | -2.606955597246 | 2.451235595023 | -1.568822070382 |
| C | -3.994201954435 | 2.545707251870 | -2.211394534540 |
| H | -3.953805893176 | 3.141839617235 | -3.128132882009 |
| H | -4.397634837679 | 1.562313513429 | -2.460894827955 |
| H | -4.694854012023 | 3.023669283324 | -1.520862715934 |
| C | -2.182133565063 | 3.867968350113 | -1.130482973500 |
| H | -2.163138524585 | 4.547638576133 | -1.984907240418 |
| H | -2.901724107604 | 4.271349338250 | -0.414814164514 |

| | | | |
|---|---|---|---|
| H | -1.195756899081 | 3.885178527970 | -0.659794044188 |
| O | -0.456105378799 | 2.268326495181 | -4.767828302183 |
| O | -3.858140300066 | 1.092351901499 | 1.793092261084 |
| C | -2.034493769505 | 0.541989809483 | -3.246440612348 |
| H | -1.223832874477 | 0.064088938560 | -3.798977374416 |
| H | -2.427294835177 | -0.181784773926 | -2.529433032471 |
| H | -2.822224980487 | 0.782448477776 | -3.962262746157 |
| C | 0.119069812905 | 3.248967789929 | -5.660837993505 |
| H | 0.943419801655 | 3.767779206796 | -5.157741693878 |
| H | -0.641057610745 | 3.989253829579 | -5.931613729889 |
| C | 0.657345398532 | 2.585223611732 | -6.944737970127 |
| H | 1.419476836026 | 1.836764014452 | -6.685788655964 |
| H | -0.160682035024 | 2.072941309934 | -7.470117205993 |
| C | -4.332746002770 | 1.651502584349 | 3.040328905622 |
| H | -5.059054860201 | 2.444700787594 | 2.833888323371 |
| H | -3.491724214002 | 2.088303556382 | 3.591371001729 |
| C | -4.998825207920 | 0.577163883418 | 3.923612744936 |
| H | -5.859748666247 | 0.141456168297 | 3.397667356907 |
| H | -4.282487429470 | -0.229223338107 | 4.134890686333 |
| O | 1.212891464810 | 3.630013144597 | -7.748979949949 |
| C | 1.778837028757 | 3.252975203617 | -9.021240397739 |
| H | 2.613987533047 | 2.551735576411 | -8.894982845765 |
| H | 1.024028510595 | 2.796956451232 | -9.674992643651 |
| H | 2.143196652999 | 4.174094037700 | -9.474436736492 |
| O | -5.407760281526 | 1.232998705155 | 5.127976537367 |
| C | -6.075068064118 | 0.430970999874 | 6.124014305695 |

| | | | |
|---|---|---|---|
| H | -7.007583590532 | 0.003102617084 | 5.733513613829 |
| H | -5.429632641959 | -0.382369074749 | 6.480491443203 |
| H | -6.302024552398 | 1.103415361578 | 6.950426957224 |

Cam_TS1-F1000

($E_F$ = -929.12165882 a.u.; $G_F$ = -928.73480771 a.u.)

0 1

| | | | |
|---|---|---|---|
| C | -1.817704561741 | 0.975147392682 | -2.753863071649 |
| C | -1.074999625479 | 2.256908222285 | -2.405473670831 |
| C | -2.051287202282 | 1.921252740332 | 0.108619576699 |
| C | -2.557521279455 | 0.648881576970 | -0.500305495348 |
| C | -1.418569242247 | -0.378611724335 | -0.724007044328 |
| C | -0.991425301749 | -0.217667249013 | -2.201461610304 |
| H | -0.068420714637 | 2.176097029101 | -1.999099505388 |
| H | -1.039009984840 | 1.949556924738 | 0.513073828060 |
| H | -3.336745950722 | 0.251645446135 | 0.163709382113 |
| H | -0.590559623864 | -0.205290866206 | -0.032384553606 |
| H | -1.780157346310 | -1.390275839655 | -0.529016468455 |
| H | -1.224882147074 | -1.116798716444 | -2.775793061545 |
| H | 0.081863025136 | -0.047576428781 | -2.317309142558 |
| C | -3.158990049871 | 0.870961620244 | -1.926389184229 |
| C | -4.011892549873 | -0.348295071965 | -2.329553889016 |
| H | -4.437755311538 | -0.221079315162 | -3.327666112964 |
| H | -3.453506648200 | -1.286693362648 | -2.320797875945 |
| H | -4.847180363197 | -0.454583184823 | -1.631389048678 |
| C | -4.036633651064 | 2.122568907567 | -2.035833922874 |

| | | | |
|---|---|---|---|
| H | -4.405107801584 | 2.241082298925 | -3.059167059280 |
| H | -4.902658225179 | 2.037668348326 | -1.373690783084 |
| H | -3.489866169292 | 3.023285203549 | -1.759984695197 |
| O | -1.250453207870 | 3.314887449170 | -3.260839312831 |
| O | -2.987811167630 | 2.601824713748 | 0.888265286902 |
| C | -2.024838131337 | 0.872029947015 | -4.273764208308 |
| H | -1.063569821547 | 0.897656210702 | -4.792581624321 |
| H | -2.514438281133 | -0.068089134228 | -4.533823271866 |
| H | -2.627982569694 | 1.698331340148 | -4.650822949043 |
| C | -0.097773654734 | 3.999971605641 | -3.790554639039 |
| H | 0.780764866752 | 3.819862358749 | -3.162924409064 |
| H | -0.324348172558 | 5.067305173942 | -3.777161100939 |
| C | 0.189011494418 | 3.572731831919 | -5.243123897679 |
| H | 0.506475627838 | 2.520304945033 | -5.272181631659 |
| H | -0.734801380147 | 3.664599087635 | -5.831686251165 |
| C | -2.904029850992 | 2.279125327716 | 2.296229015439 |
| H | -2.110420714909 | 2.868977305423 | 2.770231692220 |
| H | -2.664537243857 | 1.217767498876 | 2.423864717149 |
| C | -4.241357952773 | 2.545971885385 | 2.999062779662 |
| H | -4.507470837406 | 3.610359873818 | 2.931028532649 |
| H | -5.033844620758 | 1.963948267728 | 2.507063396753 |
| O | 1.211026907328 | 4.422002628393 | -5.754716144512 |
| C | 1.570668746799 | 4.191529626027 | -7.124159705555 |
| H | 1.959744478269 | 3.174868786171 | -7.270334645372 |
| H | 0.712911566769 | 4.343030767517 | -7.793000416247 |
| H | 2.349605504661 | 4.913981311984 | -7.366272350486 |

| | | | |
|---|---|---|---|
| O | -4.073551067968 | 2.141220841183 | 4.357308735652 |
| C | -5.239388954092 | 2.218724140946 | 5.185436319921 |
| H | -5.611799099580 | 3.249410421593 | 5.261411137105 |
| H | -6.046113127061 | 1.578270863956 | 4.803248017445 |
| H | -4.940882128790 | 1.870519164310 | 6.174076248044 |

Cam_Int1-F1000

($E_F$ = -929.18866883 a.u.; $G_F$ = -928.80461945 a.u.)

0 1

| | | | |
|---|---|---|---|
| C | -1.513514528988 | 1.793151485933 | -2.546314771907 |
| C | -0.970322362258 | 2.758825300759 | -3.575439771785 |
| C | -3.210166148668 | 2.005217621574 | 0.925162718051 |
| C | -2.605779076801 | 1.471069720855 | -0.339516202607 |
| C | -1.120268601813 | 0.993135268538 | -0.200076583962 |
| C | -0.410088853451 | 1.319892998042 | -1.538009522183 |
| H | -0.496953772755 | 3.681716921253 | -3.247588350985 |
| H | -2.758193670186 | 2.858294126398 | 1.428736849777 |
| H | -3.217235351690 | 0.611107285209 | -0.628986384763 |
| H | -0.635668149949 | 1.515019226662 | 0.628980881859 |
| H | -1.088893767782 | -0.071934331310 | 0.038999843016 |
| H | 0.138646463400 | 0.467605896834 | -1.945343024505 |
| H | 0.319011086009 | 2.120410230812 | -1.391925079019 |
| C | -2.577500784343 | 2.464942112463 | -1.562495602809 |
| C | -3.967434707950 | 2.619239853184 | -2.188625725945 |
| H | -3.914320408146 | 3.225016835100 | -3.098311625312 |
| H | -4.410142867211 | 1.654861354741 | -2.445568725712 |

| | | | |
|---|---|---|---|
| H | -4.642090396626 | 3.114150020317 | -1.484386192391 |
| C | -2.099143337612 | 3.860118612429 | -1.107937935003 |
| H | -2.068328545973 | 4.552708926213 | -1.951402235395 |
| H | -2.796022407789 | 4.275945262878 | -0.377287546862 |
| H | -1.107006133545 | 3.836840744338 | -0.649670425593 |
| O | -0.467665997887 | 2.246676628725 | -4.752934506325 |
| O | -3.838614341418 | 1.104554167709 | 1.759464234310 |
| C | -2.096172793519 | 0.560825275115 | -3.268158036540 |
| H | -1.308530801640 | 0.062954406047 | -3.835810243344 |
| H | -2.508803016192 | -0.159894204600 | -2.559344747298 |
| H | -2.880817105326 | 0.841687029543 | -3.972507945927 |
| C | 0.126282767563 | 3.217258751063 | -5.632916839430 |
| H | 0.969258763125 | 3.708698612114 | -5.132500225260 |
| H | -0.612768321901 | 3.981742797649 | -5.895770486269 |
| C | 0.629667377021 | 2.536413178824 | -6.910904824341 |
| H | 1.366745532525 | 1.761230027849 | -6.656487401546 |
| H | -0.208532166033 | 2.052257370441 | -7.431906432463 |
| C | -4.286489391149 | 1.655128109642 | 3.011883052215 |
| H | -4.988934904466 | 2.475078824692 | 2.827107178995 |
| H | -3.431187524232 | 2.052636231249 | 3.571259191794 |
| C | -4.976418616474 | 0.577791745300 | 3.856256337010 |
| H | -5.849974605702 | 0.181573189375 | 3.319287487965 |
| H | -4.282699327749 | -0.254905612617 | 4.041223681371 |
| O | 1.213785980384 | 3.559562447773 | -7.713184000958 |
| C | 1.745352156952 | 3.138039278278 | -8.975234776959 |
| H | 2.549010306269 | 2.400587902096 | -8.845780817812 |

| | | | |
|---|---|---|---|
| H | 0.964761557598 | 2.703697323607 | -9.614302827621 |
| H | 2.148383479433 | 4.029827193474 | -9.454472279055 |
| O | -5.362475614665 | 1.208136393970 | 5.075307147174 |
| C | -6.042376597850 | 0.376886498688 | 6.023648855523 |
| H | -6.981469913051 | -0.016500706796 | 5.611661645857 |
| H | -5.413887879577 | -0.465912097450 | 6.341750719538 |
| H | -6.262826855385 | 1.007008869800 | 6.884895957937 |

Cam-F1000

($E_F$ = -929.18259484 a.u.; $G_F$ = -928.78773634 a.u.)

0 1

| | | | |
|---|---|---|---|
| C | -1.940303159443 | 0.761735964549 | -2.638732774057 |
| C | -1.257074869623 | 1.961290326067 | -1.892837639396 |
| C | -1.848843114279 | 1.819659815935 | -0.401230678210 |
| C | -2.748520302882 | 0.566135204037 | -0.528811011102 |
| C | -1.806273282064 | -0.652869743245 | -0.642441136321 |
| C | -1.244225458263 | -0.513191037728 | -2.087785236979 |
| H | -0.177174107189 | 1.784473734663 | -1.839846604960 |
| H | -1.009739710645 | 1.620035435281 | 0.278079252261 |
| H | -3.480444573733 | 0.491121240041 | 0.273951868308 |
| H | -1.022012604033 | -0.630419318516 | 0.118606421543 |
| H | -2.350123205726 | -1.589423355748 | -0.509853958426 |
| H | -1.494511314324 | -1.379885732708 | -2.703101805063 |
| H | -0.155622053593 | -0.414199588149 | -2.112319777097 |
| C | -3.348283261361 | 0.688278507277 | -1.955389776386 |
| C | -4.192002117721 | -0.531610330597 | -2.365077441410 |

| | | | |
|---|---|---|---|
| H | -4.543621006264 | -0.423546761141 | -3.394993291552 |
| H | -3.665409302959 | -1.483541580890 | -2.296535380350 |
| H | -5.077604201444 | -0.597429791701 | -1.725497239961 |
| C | -4.222166653521 | 1.934588323014 | -2.178225187976 |
| H | -4.540454098151 | 1.980676801847 | -3.224346198650 |
| H | -5.123237672094 | 1.869461418933 | -1.560003838208 |
| H | -3.718265357457 | 2.865541695936 | -1.937529056181 |
| O | -1.464169220057 | 3.219843063280 | -2.524117279107 |
| O | -2.540203988910 | 2.964974926539 | 0.085647163912 |
| C | -1.925467015776 | 0.849977174565 | -4.153392559299 |
| H | -0.907693595359 | 0.770433158770 | -4.544446736920 |
| H | -2.504070146899 | 0.029174357783 | -4.585620998295 |
| H | -2.351152207455 | 1.792646939248 | -4.498446529444 |
| C | -0.324806144130 | 3.898165396963 | -3.066734439841 |
| H | 0.581859557201 | 3.651155316869 | -2.501562299152 |
| H | -0.515724491318 | 4.966727646019 | -2.944381103794 |
| C | -0.082437327041 | 3.630187502021 | -4.567777977542 |
| H | 0.239500591674 | 2.593143564989 | -4.728931966532 |
| H | -1.021854797727 | 3.783486285096 | -5.117975915703 |
| C | -2.571575929274 | 3.124664090114 | 1.507590071668 |
| H | -2.801355437498 | 4.178116348386 | 1.678768159834 |
| H | -1.583132788526 | 2.918203696151 | 1.939260552649 |
| C | -3.634557296399 | 2.296757171160 | 2.267594182963 |
| H | -4.606403109403 | 2.400087322837 | 1.762829323232 |
| H | -3.369277590956 | 1.230618303517 | 2.285229421762 |
| O | 0.924375469608 | 4.534263401390 | -5.017488906610 |

| | | | |
|---|---|---|---|
| C | 1.278588830793 | 4.407816863155 | -6.400874979779 |
| H | 1.679125584208 | 3.409034271238 | -6.622490304645 |
| H | 0.415373254883 | 4.595449750180 | -7.053761631621 |
| H | 2.047171783190 | 5.155523952735 | -6.595739170682 |
| O | -3.686801813288 | 2.809975963551 | 3.597551661194 |
| C | -4.586703713338 | 2.135482138616 | 4.485529431560 |
| H | -5.623034783614 | 2.192868607372 | 4.125879046255 |
| H | -4.312529659420 | 1.078619710419 | 4.608120949800 |
| H | -4.511008364846 | 2.640975830310 | 5.448055383896 |

Cam_TS1-F800

($E_F$ = -929.05840319 a.u.; $G_F$ = -928.67120573 a.u.)

0 1

| | | | |
|---|---|---|---|
| C | -1.821943025863 | 0.929812769732 | -2.709530378270 |
| C | -1.003253548659 | 2.161137178321 | -2.372757722874 |
| C | -2.253549539404 | 1.890357324745 | 0.192099588114 |
| C | -2.814688370353 | 0.700568815141 | -0.518105863550 |
| C | -1.761671980698 | -0.430122912158 | -0.630940306719 |
| C | -1.150273646160 | -0.303548939441 | -2.045894486320 |
| H | 0.001554813872 | 2.030453441142 | -1.975201993587 |
| H | -1.312811793400 | 1.806852569893 | 0.733124849838 |
| H | -3.700763785938 | 0.373400679127 | 0.043165631313 |
| H | -1.006398166080 | -0.349828630144 | 0.154689499132 |
| H | -2.242263829904 | -1.401791805064 | -0.500259933030 |
| H | -1.362490141431 | -1.194776112034 | -2.640746686279 |
| H | -0.062434216721 | -0.199639364857 | -2.025817774789 |

| | | | |
|---|---|---|---|
| C | -3.234229141721 | 0.978827938680 | -2.001489538653 |
| C | -4.167277272320 | -0.149954885500 | -2.485341409047 |
| H | -4.493465552841 | 0.021195873578 | -3.513791272659 |
| H | -3.702639901158 | -1.137706346223 | -2.442036330988 |
| H | -5.064330631163 | -0.177724441580 | -1.860026879803 |
| C | -3.963356172543 | 2.313486859860 | -2.188057418722 |
| H | -4.202715075807 | 2.472959013276 | -3.243574975466 |
| H | -4.901293902270 | 2.314303469515 | -1.625392198063 |
| H | -3.361267597607 | 3.152371987050 | -1.843176229846 |
| O | -1.146803457709 | 3.238212062729 | -3.216195152739 |
| O | -3.181636777601 | 2.730059481575 | 0.768332609702 |
| C | -1.909338519570 | 0.766213405114 | -4.239226436043 |
| H | -0.906473962842 | 0.696772510143 | -4.668943970809 |
| H | -2.445897698735 | -0.145740070513 | -4.506863112170 |
| H | -2.411198303668 | 1.617491782232 | -4.700433679953 |
| C | 0.023934038436 | 3.895129803714 | -3.729648702903 |
| H | 0.909812809686 | 3.624743216437 | -3.145985284355 |
| H | -0.136838712939 | 4.971490734801 | -3.641029693835 |
| C | 0.238731828476 | 3.545386340673 | -5.210795153971 |
| H | 0.479052058502 | 2.477691925097 | -5.316279846390 |
| H | -0.692302193416 | 3.738637513092 | -5.762903529988 |
| C | -3.071314553105 | 3.025151243602 | 2.170689333890 |
| H | -3.335245618183 | 4.076020089791 | 2.303810452362 |
| H | -2.045019250044 | 2.875118543326 | 2.522008184929 |
| C | -4.040474188381 | 2.158873570023 | 2.990253416564 |
| H | -5.056434934346 | 2.282078440416 | 2.588426019412 |

| | | | |
|---|---|---|---|
| H | -3.765874634055 | 1.096915489344 | 2.898565238487 |
| O | 1.302149331356 | 4.353746048612 | -5.700117634078 |
| C | 1.598974518839 | 4.178082700722 | -7.088428080935 |
| H | 1.907887801751 | 3.146306449368 | -7.305421815235 |
| H | 0.733459981108 | 4.427528781747 | -7.717296812294 |
| H | 2.420282213355 | 4.855484886911 | -7.321595138639 |
| O | -3.968232773949 | 2.590718042877 | 4.344110901484 |
| C | -4.887684441531 | 1.952157045324 | 5.233955244059 |
| H | -5.928155378825 | 2.129932646372 | 4.929555439425 |
| H | -4.713762411369 | 0.868367742043 | 5.282048344393 |
| H | -4.722247810108 | 2.386103283673 | 6.219966602240 |

Cam_Int1-F800

($E_F$ = -929.11014652 a.u.; $G_F$ = -928.72608852 a.u.)

0 1

| | | | |
|---|---|---|---|
| C | -1.524807480740 | 1.808952852885 | -2.540412250944 |
| C | -0.969760821707 | 2.756361350777 | -3.575503997082 |
| C | -3.204920125327 | 2.081718540673 | 0.923184078927 |
| C | -2.613018127746 | 1.526241435738 | -0.334631178990 |
| C | -1.137172821024 | 1.021324504774 | -0.191833864976 |
| C | -0.425403604994 | 1.322437017427 | -1.534069103862 |
| H | -0.484203123711 | 3.676245420524 | -3.257252077791 |
| H | -2.734066736658 | 2.925493101550 | 1.425186126295 |
| H | -3.241104839948 | 0.675540800804 | -0.615588124547 |
| H | -0.641384958756 | 1.541714666863 | 0.631536592829 |
| H | -1.125916349407 | -0.041855686372 | 0.057202457588 |

| | | | |
|---|---|---|---|
| H | 0.104233093339 | 0.456195447823 | -1.937137597385 |
| H | 0.320031334886 | 2.109550004017 | -1.397890731805 |
| C | -2.570658435697 | 2.509259437557 | -1.562221939945 |
| C | -3.959599292648 | 2.685454377257 | -2.185214656715 |
| H | -3.897718643558 | 3.282254846280 | -3.100235257514 |
| H | -4.422071840324 | 1.727817273513 | -2.432338510757 |
| H | -4.621892411061 | 3.199479225526 | -1.482969098625 |
| C | -2.065030918951 | 3.898843280026 | -1.119376828038 |
| H | -2.026031541309 | 4.585474486359 | -1.967305543814 |
| H | -2.751017545402 | 4.331795428426 | -0.388435881043 |
| H | -1.071341585529 | 3.860880241758 | -0.665487597990 |
| O | -0.468752027733 | 2.220005084087 | -4.740436443826 |
| O | -3.851393265023 | 1.195916482606 | 1.756154504066 |
| C | -2.132838780269 | 0.582109984826 | -3.251230648608 |
| H | -1.356326861096 | 0.065298973603 | -3.817071723396 |
| H | -2.556877893966 | -0.125415265889 | -2.535932649724 |
| H | -2.914079144365 | 0.872354795115 | -3.955563709688 |
| C | 0.138262223912 | 3.169565732578 | -5.629961807273 |
| H | 0.986173264452 | 3.658497513764 | -5.135174959272 |
| H | -0.590076591304 | 3.939020771670 | -5.908218448199 |
| C | 0.633480666093 | 2.457170023901 | -6.889595368943 |
| H | 1.358977775743 | 1.676785992811 | -6.617824728153 |
| H | -0.210265539664 | 1.974037192135 | -7.402780930185 |
| C | -4.289777562110 | 1.757310404736 | 3.003887049885 |
| H | -4.975605270877 | 2.591026749780 | 2.817644635118 |
| H | -3.428653762098 | 2.137445523376 | 3.566641388077 |

| | | | |
|---|---|---|---|
| C | -5.000636890843 | 0.690916287493 | 3.838393843645 |
| H | -5.879408383940 | 0.313086764857 | 3.296424748540 |
| H | -4.322975018853 | -0.155517736218 | 4.021385845456 |
| O | 1.232544459471 | 3.454707518379 | -7.709184114526 |
| C | 1.755732328075 | 2.989912565297 | -8.955347400567 |
| H | 2.546081810776 | 2.241785166804 | -8.805142091714 |
| H | 0.968071144126 | 2.551047428410 | -9.582909265832 |
| H | 2.175414002011 | 3.859158868610 | -9.461280669404 |
| O | -5.378427676979 | 1.321908426478 | 5.057245720522 |
| C | -6.074530416349 | 0.490917346072 | 5.988468194998 |
| H | -7.016994779571 | 0.116326023653 | 5.566227002999 |
| H | -5.461031270813 | -0.365843776950 | 6.299186105736 |
| H | -6.291946764403 | 1.110955036395 | 6.857929286021 |

Cam-F800

($E_F$ = -929.12507898 a.u.; $G_F$ = -928.73091641 a.u.)

0 1

| | | | |
|---|---|---|---|
| C | -1.996123749111 | 0.765136894369 | -2.650966951203 |
| C | -1.276330255512 | 1.942345466148 | -1.907904574203 |
| C | -1.855247235674 | 1.817692952710 | -0.416772129795 |
| C | -2.794726650684 | 0.592018650452 | -0.534492019778 |
| C | -1.886133675438 | -0.652184293927 | -0.654906912371 |
| C | -1.330587999738 | -0.527954829793 | -2.104696700947 |
| H | -0.199377495722 | 1.744312442558 | -1.873849810906 |
| H | -1.021145725631 | 1.595143139298 | 0.261690831422 |
| H | -3.522241027187 | 0.537152110246 | 0.273952718110 |

| | | | |
|---|---|---|---|
| H | -1.095870666019 | -0.651068124147 | 0.100244263217 |
| H | -2.454001325009 | -1.573711386061 | -0.518137135578 |
| H | -1.607745330679 | -1.387838158901 | -2.717950310047 |
| H | -0.239896806701 | -0.457865994484 | -2.136374867993 |
| C | -3.401355974131 | 0.727873642865 | -1.957488551734 |
| C | -4.276779636708 | -0.471771575218 | -2.361177469261 |
| H | -4.631434382240 | -0.355374828999 | -3.389156713474 |
| H | -3.773317575784 | -1.436261250037 | -2.295402211453 |
| H | -5.160049179718 | -0.515809711965 | -1.716532772440 |
| C | -4.248657446561 | 1.993534519175 | -2.174602043329 |
| H | -4.572716477022 | 2.046953802902 | -3.218612835969 |
| H | -5.147100812185 | 1.947162574470 | -1.550783549022 |
| H | -3.722828606523 | 2.912718344174 | -1.936611320373 |
| O | -1.474662265423 | 3.207477819150 | -2.524574243656 |
| O | -2.500321300343 | 2.994564235936 | 0.053256184045 |
| C | -1.986348751165 | 0.859768411087 | -4.165436408338 |
| H | -0.971742260079 | 0.763366761457 | -4.561294602687 |
| H | -2.582923304037 | 0.052972113263 | -4.599651604771 |
| H | -2.394351656031 | 1.812961884332 | -4.503234180162 |
| C | -0.325124437378 | 3.895064963277 | -3.022846378924 |
| H | 0.560279004826 | 3.672582668758 | -2.415171454482 |
| H | -0.537309110800 | 4.962267645202 | -2.927823871105 |
| C | -0.023669628167 | 3.596902552501 | -4.503049818746 |
| H | 0.300971429643 | 2.554951435140 | -4.628997033127 |
| H | -0.940741342147 | 3.738783481067 | -5.092666321721 |
| C | -2.515008979996 | 3.181241427320 | 1.469074037995 |

| | | | |
|---|---|---|---|
| H | -2.717729959227 | 4.242706228309 | 1.624618376312 |
| H | -1.529282646995 | 2.957091470840 | 1.898620699884 |
| C | -3.592850547279 | 2.390237133843 | 2.239139691905 |
| H | -4.562812568519 | 2.510076503517 | 1.734085966391 |
| H | -3.352954570481 | 1.318203868174 | 2.269004667229 |
| O | 0.999772574923 | 4.489133700588 | -4.930969751251 |
| C | 1.379476044664 | 4.343271652819 | -6.301917226843 |
| H | 1.775464532711 | 3.338366160638 | -6.504289535268 |
| H | 0.531049857330 | 4.529752863943 | -6.974565917035 |
| H | 2.158547667284 | 5.081251421261 | -6.493002223714 |
| O | -3.631042436624 | 2.919911847652 | 3.560803668828 |
| C | -4.550865271337 | 2.273894611448 | 4.443817844823 |
| H | -5.583232502211 | 2.353579242802 | 4.076445756070 |
| H | -4.303801083577 | 1.211060585812 | 4.574117976242 |
| H | -4.471803789112 | 2.781135590241 | 5.405326831933 |

Cam_TS1-F600

($E_F$ = -928.99354045 a.u.; $G_F$ = -928.60751483 a.u.)

0 1

| | | | |
|---|---|---|---|
| C | -1.834652763254 | 0.935297598912 | -2.758804335002 |
| C | -0.987058195125 | 2.150724979483 | -2.452439973942 |
| C | -2.294968034703 | 1.851938756882 | 0.246828060085 |
| C | -2.790611642993 | 0.679538040469 | -0.531861408819 |
| C | -1.690165274011 | -0.405521059247 | -0.674261905389 |
| C | -1.159615162468 | -0.307426113437 | -2.122897648617 |
| H | 0.029124411619 | 2.017385757975 | -2.086603722896 |

| | | | |
|---|---|---|---|
| H | -1.360972605740 | 1.797658703836 | 0.801396554167 |
| H | -3.667406949365 | 0.288092937489 | 0.003632339723 |
| H | -0.895038805227 | -0.255779077690 | 0.060211512749 |
| H | -2.108955333903 | -1.394742162182 | -0.478344361813 |
| H | -1.433558593184 | -1.196763769936 | -2.694900327646 |
| H | -0.069775636310 | -0.237005584246 | -2.167891219231 |
| C | -3.226233273398 | 0.992642970265 | -2.007837924007 |
| C | -4.183515343145 | -0.119889178410 | -2.483843614096 |
| H | -4.515157204843 | 0.052914055881 | -3.510081649289 |
| H | -3.735044424893 | -1.115400548329 | -2.438193519612 |
| H | -5.075615582204 | -0.130024367224 | -1.851113851847 |
| C | -3.943792345980 | 2.337323733278 | -2.160867065579 |
| H | -4.209374948730 | 2.510467677163 | -3.207921711765 |
| H | -4.864940272748 | 2.344668515117 | -1.571833116247 |
| H | -3.321403001155 | 3.164643570376 | -1.824566202726 |
| O | -1.175767346645 | 3.252506292590 | -3.250546427059 |
| O | -3.263146995479 | 2.658520572396 | 0.795673560460 |
| C | -1.971107857797 | 0.780406358622 | -4.287438206367 |
| H | -0.981990432666 | 0.714634521275 | -4.748566837932 |
| H | -2.516216388369 | -0.129068980476 | -4.545721448771 |
| H | -2.486371274138 | 1.635692892830 | -4.726289739073 |
| C | -0.026573760475 | 3.947807839498 | -3.752359384584 |
| H | 0.844424499036 | 3.778930208670 | -3.110355928914 |
| H | -0.264228599471 | 5.012984045887 | -3.742694480841 |
| C | 0.282250048804 | 3.518477510524 | -5.191576979194 |
| H | 0.584112904472 | 2.460889419293 | -5.214377791005 |

| | | | |
|---|---|---|---|
| H | -0.627938848966 | 3.622985146864 | -5.799566350759 |
| C | -3.148880651302 | 3.028530439212 | 2.176610108979 |
| H | -3.477411129204 | 4.065634536966 | 2.265748545343 |
| H | -2.110367447338 | 2.960218299578 | 2.517309305078 |
| C | -4.049411529763 | 2.138333770230 | 3.040443179085 |
| H | -5.075738183897 | 2.181829861133 | 2.648188901940 |
| H | -3.709241615273 | 1.092874864119 | 2.983505653106 |
| O | 1.322826310539 | 4.353340948138 | -5.679228919752 |
| C | 1.668471633525 | 4.124647633366 | -7.044753940679 |
| H | 2.029543881434 | 3.098756345606 | -7.201773268850 |
| H | 0.812108008144 | 4.302355299633 | -7.709761100216 |
| H | 2.465542969411 | 4.826208124715 | -7.290896034629 |
| O | -3.989527581790 | 2.624046371668 | 4.374154198556 |
| C | -4.864206920306 | 1.960146866122 | 5.284992172608 |
| H | -5.914663221913 | 2.065802232969 | 4.980586209373 |
| H | -4.626237725686 | 0.890259256607 | 5.364291360555 |
| H | -4.723735280153 | 2.430812077836 | 6.258094647680 |

Cam-F600

($E_F$ = -929.06817690 a.u.; $G_F$ = -928.67341716 a.u.)

0 1

| | | | |
|---|---|---|---|
| C | -2.031341422664 | 0.750574194466 | -2.675828101817 |
| C | -1.307674581571 | 1.926134004226 | -1.938831176991 |
| C | -1.859169599210 | 1.797712278514 | -0.444130921344 |
| C | -2.799935864725 | 0.571009255346 | -0.547793076505 |
| C | -1.886925455765 | -0.668957704225 | -0.683461333453 |

| | | | |
|---|---|---|---|
| C | -1.352452520947 | -0.540409968535 | -2.141326489698 |
| H | -0.227980781945 | 1.741140487396 | -1.931288813812 |
| H | -1.018594003142 | 1.577343712784 | 0.227058938363 |
| H | -3.515265461475 | 0.511689200537 | 0.271277108897 |
| H | -1.085673975861 | -0.665420695022 | 0.060017127388 |
| H | -2.448436398218 | -1.593271377597 | -0.539799659792 |
| H | -1.634172255810 | -1.400710695419 | -2.751832890432 |
| H | -0.262625863827 | -0.465239771966 | -2.187943395939 |
| C | -3.427243632873 | 0.703951690360 | -1.962745425767 |
| C | -4.299124223900 | -0.501665065124 | -2.356932483082 |
| H | -4.666056894148 | -0.386416237964 | -3.380752103101 |
| H | -3.788830023330 | -1.462941955740 | -2.298052812531 |
| H | -5.174699212185 | -0.552183334542 | -1.702358304473 |
| C | -4.288706723145 | 1.962434552354 | -2.165601766151 |
| H | -4.626082174796 | 2.016235710722 | -3.205382200814 |
| H | -5.179238423582 | 1.904719526041 | -1.531340092299 |
| H | -3.768481546942 | 2.885273463336 | -1.930463195082 |
| O | -1.541259645139 | 3.190840676468 | -2.540198205280 |
| O | -2.491003978727 | 2.984064392339 | 0.015707319264 |
| C | -2.038692006463 | 0.856062102538 | -4.189864410981 |
| H | -1.026321733497 | 0.780229926634 | -4.596373762996 |
| H | -2.627077191025 | 0.043767431948 | -4.625086593752 |
| H | -2.464003788727 | 1.806136355874 | -4.515173978538 |
| C | -0.403993837550 | 3.928377174696 | -2.982336206120 |
| H | 0.454251856864 | 3.770407773014 | -2.317957609503 |
| H | -0.678275322040 | 4.984092846266 | -2.929924488833 |

| | | | |
|---|---|---|---|
| C | -0.008794002667 | 3.600141845434 | -4.429469612337 |
| H | 0.352741051006 | 2.564385196024 | -4.504046804697 |
| H | -0.895026293444 | 3.694252517216 | -5.073287344265 |
| C | -2.484619543413 | 3.194045521921 | 1.425394220977 |
| H | -2.684834064671 | 4.257792887649 | 1.568730183251 |
| H | -1.494023148004 | 2.974794754510 | 1.846576586641 |
| C | -3.553791944586 | 2.412669787279 | 2.209182960731 |
| H | -4.527066297610 | 2.527413094952 | 1.709091923259 |
| H | -3.313831918709 | 1.340776260138 | 2.247148423360 |
| O | 1.007628078274 | 4.511072462259 | -4.825995909914 |
| C | 1.435383374005 | 4.352578480938 | -6.177630245040 |
| H | 1.859806357539 | 3.353631562916 | -6.350872339960 |
| H | 0.606138812636 | 4.508762057216 | -6.881500351964 |
| H | 2.204421708514 | 5.103999342258 | -6.357473633211 |
| O | -3.583458474662 | 2.954182335144 | 3.524025969069 |
| C | -4.504709680620 | 2.314379491159 | 4.404558975540 |
| H | -5.536171044059 | 2.394319663038 | 4.034147343450 |
| H | -4.260756000445 | 1.251133841451 | 4.538947191253 |
| H | -4.429194570906 | 2.822902656196 | 5.365842426237 |

Cam_Int1-F600

($E_F$ = -929.03196324 a.u.; $G_F$ = -928.64781319 a.u.)

0 1

| | | | |
|---|---|---|---|
| C | -1.576647695422 | 1.811194683486 | -2.563282847205 |
| C | -0.941303781640 | 2.745985151985 | -3.558363193134 |
| C | -3.123198488603 | 2.074740957940 | 0.952014523382 |

| | | | |
|---|---|---|---|
| C | -2.627925817467 | 1.530714077732 | -0.348379455084 |
| C | -1.219066440055 | 0.851958696400 | -0.276397992293 |
| C | -0.515670285309 | 1.135975355281 | -1.626216739143 |
| H | -0.353455668020 | 3.590690102508 | -3.206164053433 |
| H | -2.541191187022 | 2.815629792031 | 1.497974682856 |
| H | -3.357446408011 | 0.776405345275 | -0.658005795991 |
| H | -0.643942293552 | 1.271313157932 | 0.553034129552 |
| H | -1.325276902546 | -0.216276686914 | -0.076225311505 |
| H | -0.113293558596 | 0.234488411136 | -2.093689691321 |
| H | 0.327497158524 | 1.814845538754 | -1.478126850380 |
| C | -2.500430489204 | 2.572856700482 | -1.515979431508 |
| C | -3.873895319870 | 2.952755208320 | -2.079718308866 |
| H | -3.764128643833 | 3.591009906011 | -2.961600872105 |
| H | -4.457365907003 | 2.074689950492 | -2.363526785387 |
| H | -4.448159263094 | 3.500588396316 | -1.327330628798 |
| C | -1.817501082395 | 3.862233395822 | -1.011126468415 |
| H | -1.724449366489 | 4.593500643847 | -1.816420886086 |
| H | -2.422565758994 | 4.324226645236 | -0.228395631486 |
| H | -0.821150067769 | 3.678551206264 | -0.600763134769 |
| O | -0.515305596906 | 2.212063386078 | -4.751486048307 |
| O | -3.866190097430 | 1.223197133368 | 1.735193044866 |
| C | -2.347965116147 | 0.710466192734 | -3.321444952075 |
| H | -1.655513573204 | 0.141167433995 | -3.943203109109 |
| H | -2.832816783793 | 0.015396774841 | -2.633125600631 |
| H | -3.108883229588 | 1.133388475442 | -3.979414515810 |
| C | 0.181045335960 | 3.128544535520 | -5.605227251846 |

| | | | |
|---|---|---|---|
| H | 1.085208054859 | 3.496895472761 | -5.105339039776 |
| H | -0.459763754235 | 3.986309623517 | -5.837019518828 |
| C | 0.572572047859 | 2.418493999973 | -6.898108433784 |
| H | 1.206599956366 | 1.549056117377 | -6.670905348014 |
| H | -0.328342490068 | 2.056867185177 | -7.414426522933 |
| C | -4.219888597509 | 1.749537169481 | 3.021313506646 |
| H | -4.803281441577 | 2.669285727278 | 2.902789567541 |
| H | -3.314473051174 | 1.985752176388 | 3.593528101384 |
| C | -5.042897211306 | 0.717923968454 | 3.787512704437 |
| H | -5.962933590108 | 0.483500842478 | 3.232870865477 |
| H | -4.467164530346 | -0.212264443154 | 3.900360540901 |
| O | 1.270451377643 | 3.372849089622 | -7.686831845511 |
| C | 1.710797034511 | 2.893663197577 | -8.955440170299 |
| H | 2.407976726337 | 2.051260127345 | -8.847305382704 |
| H | 0.865297098573 | 2.573519839900 | -9.579891943295 |
| H | 2.223396283253 | 3.722149297521 | -9.444172764881 |
| O | -5.336639378310 | 1.298774244026 | 5.051188158427 |
| C | -6.114375736079 | 0.479920616105 | 5.921311540162 |
| H | -7.094302033629 | 0.244865421903 | 5.483337259242 |
| H | -5.598271234917 | -0.461366230387 | 6.155960302152 |
| H | -6.259936295329 | 1.047129314579 | 6.840597408118 |

Cam_TS1-F400

($E_F$ = -928.92862315 a.u.; $G_F$ = -928.54274974 a.u.)

0 1

| | | | |
|---|---|---|---|
| C | -1.866278971849 | 0.927921664088 | -2.833685623414 |

| | | | |
|---|---|---|---|
| C | -0.981060859141 | 2.117657124927 | -2.545173541908 |
| C | -2.361126014234 | 1.807332399054 | 0.290918982206 |
| C | -2.745717304737 | 0.646843649706 | -0.559775506653 |
| C | -1.548280984188 | -0.330441278803 | -0.739615217085 |
| C | -1.193084946105 | -0.330340512526 | -2.240142566481 |
| H | 0.048207061651 | 1.965313472490 | -2.226639734081 |
| H | -1.442805023748 | 1.804564483886 | 0.872396962458 |
| H | -3.585171217313 | 0.146799132759 | -0.054575798781 |
| H | -0.695828612303 | 0.000890288562 | -0.141055727356 |
| H | -1.803907748004 | -1.332151019266 | -0.387815128462 |
| H | -1.594978665649 | -1.218028528511 | -2.733821565228 |
| H | -0.114502276540 | -0.337666805576 | -2.419207320994 |
| C | -3.223923402456 | 0.995921294000 | -2.021605848521 |
| C | -4.220055031641 | -0.091812066377 | -2.475759968286 |
| H | -4.566915212396 | 0.083090226524 | -3.496573706939 |
| H | -3.798612880857 | -1.099176045095 | -2.428519279841 |
| H | -5.099315173473 | -0.073174314683 | -1.825552900726 |
| C | -3.924234621898 | 2.354021043700 | -2.132020497910 |
| H | -4.228689226873 | 2.540922111227 | -3.166043231031 |
| H | -4.818358660320 | 2.372350402365 | -1.504067057967 |
| H | -3.274263874831 | 3.167313930890 | -1.814798195309 |
| O | -1.206931779559 | 3.254699251674 | -3.278881636963 |
| O | -3.399628612937 | 2.537384794886 | 0.810631764579 |
| C | -2.066644580354 | 0.800324249222 | -4.357880160894 |
| H | -1.097443355027 | 0.740598879488 | -4.860533372801 |
| H | -2.626171074770 | -0.102105087940 | -4.610362790890 |

| | | | |
|---|---|---|---|
| H | -2.595848466350 | 1.665634036925 | -4.759393740675 |
| C | -0.070964665762 | 3.981083241635 | -3.757765589363 |
| H | 0.775115229191 | 3.886251436964 | -3.068226617244 |
| H | -0.362451400571 | 5.031231220137 | -3.806761517110 |
| C | 0.325979613781 | 3.501076987132 | -5.154904569777 |
| H | 0.651149443211 | 2.450404866983 | -5.114031225122 |
| H | -0.550326927134 | 3.556995051132 | -5.816847686823 |
| C | -3.288886721661 | 3.025666142894 | 2.151030063232 |
| H | -3.728133726509 | 4.024663351684 | 2.174728543046 |
| H | -2.240903660093 | 3.097924915387 | 2.460240341332 |
| C | -4.062759854040 | 2.107822172778 | 3.098162653510 |
| H | -5.095917931111 | 2.010064568657 | 2.734233346416 |
| H | -3.609251108087 | 1.104522366473 | 3.100779717398 |
| O | 1.372221789904 | 4.339243670728 | -5.617855977953 |
| C | 1.778239307385 | 4.067020706383 | -6.955053696980 |
| H | 2.159632136634 | 3.041532901392 | -7.059114511202 |
| H | 0.948820449399 | 4.206790848463 | -7.662194601165 |
| H | 2.575717916971 | 4.770319729618 | -7.195417476015 |
| O | -4.028693456626 | 2.688519789570 | 4.391845792872 |
| C | -4.810879089458 | 1.993065082961 | 5.356716220691 |
| H | -5.871158883310 | 1.965808476276 | 5.068922399293 |
| H | -4.457527925037 | 0.961437131098 | 5.494287960314 |
| H | -4.709662538205 | 2.533176756353 | 6.298274752948 |

Cam-F400

($E_F$ = -929.01194947 a.u.; $G_F$ = -928.61742012 a.u.)

0 1

| | | | |
|---|---|---|---|
| C | -2.069756228100 | 0.718872960495 | -2.701551314977 |
| C | -1.347079330447 | 1.895600530713 | -1.972375277030 |
| C | -1.883053573004 | 1.771573952978 | -0.477474869930 |
| C | -2.831868020733 | 0.548950594150 | -0.569472820080 |
| C | -1.920753085263 | -0.692877173409 | -0.702878082303 |
| C | -1.388091025234 | -0.568931718106 | -2.162269627901 |
| H | -0.265410202954 | 1.722982250382 | -1.981802096656 |
| H | -1.041560338582 | 1.549660274239 | 0.191946044236 |
| H | -3.544071798835 | 0.495701152838 | 0.252947511863 |
| H | -1.118244666724 | -0.688935642395 | 0.039216999287 |
| H | -2.483575312315 | -1.615848502668 | -0.556090504313 |
| H | -1.668275791542 | -1.432233316696 | -2.769139107621 |
| H | -0.298444326761 | -0.491569233247 | -2.209869957854 |
| C | -3.464087512878 | 0.673514252119 | -1.983804898881 |
| C | -4.333178909551 | -0.536139145010 | -2.372357957605 |
| H | -4.703264214965 | -0.423287948951 | -3.395347591260 |
| H | -3.819656070233 | -1.495674608018 | -2.314015009757 |
| H | -5.206648069477 | -0.588715925324 | -1.715163687595 |
| C | -4.331982652090 | 1.927521151908 | -2.186809449234 |
| H | -4.666747258513 | 1.982178015951 | -3.227386376871 |
| H | -5.224165280479 | 1.861874354714 | -1.555527125033 |
| H | -3.817798988171 | 2.852415950034 | -1.947436306562 |
| O | -1.610004851762 | 3.155188241987 | -2.569326872899 |
| O | -2.498036004271 | 2.971231592699 | -0.033252309318 |
| C | -2.074101106656 | 0.824570843855 | -4.215740449173 |

| | | | |
|---|---|---|---|
| H | -1.058170606274 | 0.765366575273 | -4.617142530171 |
| H | -2.648938734264 | 0.004730210119 | -4.654962956169 |
| H | -2.510148759901 | 1.769830368968 | -4.541045098278 |
| C | -0.484235101886 | 3.943685513471 | -2.937601759013 |
| H | 0.339517626585 | 3.824559405398 | -2.223287918023 |
| H | -0.806674139781 | 4.986486075195 | -2.905785955246 |
| C | 0.000185619568 | 3.619185857336 | -4.353946993570 |
| H | 0.378758874526 | 2.587168958963 | -4.402457859133 |
| H | -0.848676161201 | 3.696881006684 | -5.048640049124 |
| C | -2.463654412281 | 3.211935314100 | 1.368357608110 |
| H | -2.669171036803 | 4.276844587458 | 1.494968809307 |
| H | -1.463916857852 | 3.007493936893 | 1.775596833735 |
| C | -3.512082899170 | 2.435117036693 | 2.176365856957 |
| H | -4.493854950075 | 2.538793376521 | 1.690427795857 |
| H | -3.265408882303 | 1.364747147558 | 2.220656131434 |
| O | 1.022986891147 | 4.542599646796 | -4.693436252875 |
| C | 1.511206690832 | 4.389632407755 | -6.021411310341 |
| H | 1.950935333255 | 3.394160220819 | -6.177014292613 |
| H | 0.713356947318 | 4.539624807123 | -6.762286125674 |
| H | 2.282004114936 | 5.146628557021 | -6.168271621771 |
| O | -3.524233264763 | 2.989468467658 | 3.483891401095 |
| C | -4.439468671173 | 2.355409481765 | 4.369571133555 |
| H | -5.472933665010 | 2.434107340770 | 4.003916967783 |
| H | -4.196310105818 | 1.292168577204 | 4.507061655740 |
| H | -4.360710020367 | 2.866160833619 | 5.329577768360 |

Cam_Int1-F400

($E_F$ = -928.95123648 a.u.; $G_F$ = -928.56778312 a.u.)

0 1

| | | | |
|---|---|---|---|
| C | -1.379517911107 | 1.500170164814 | -2.528102564848 |
| C | -0.486460501616 | 2.243771337860 | -3.477937878544 |
| C | -3.171721950858 | 2.133275526311 | 0.809395778068 |
| C | -2.633771493179 | 1.479377494559 | -0.418378917439 |
| C | -1.334945459637 | 0.643487797944 | -0.182849186911 |
| C | -0.513240355568 | 0.730064645102 | -1.492506236199 |
| H | 0.561110750074 | 1.976075997742 | -3.599123930913 |
| H | -2.602979460644 | 2.908167742026 | 1.321367505594 |
| H | -3.416336973441 | 0.797734088104 | -0.767396223218 |
| H | -0.769967588228 | 1.060229948258 | 0.654894765760 |
| H | -1.586291638396 | -0.383513052597 | 0.089042430439 |
| H | -0.235066060309 | -0.254366134091 | -1.876576366098 |
| H | 0.418870865441 | 1.273147159440 | -1.314263398104 |
| C | -2.275111775678 | 2.436195962421 | -1.603125055690 |
| C | -3.530884485631 | 2.972847009135 | -2.299887347351 |
| H | -3.259414483865 | 3.549341554422 | -3.187356093169 |
| H | -4.202288516967 | 2.168485305026 | -2.607721181125 |
| H | -4.087091200320 | 3.621815924363 | -1.617359133791 |
| C | -1.443901715209 | 3.636905340408 | -1.108068352354 |
| H | -1.125717534574 | 4.250533218396 | -1.953142907535 |
| H | -2.040379235060 | 4.266783972517 | -0.443461068200 |
| H | -0.546315929267 | 3.328631133787 | -0.566181385181 |
| O | -1.089875670482 | 2.855178451567 | -4.547712862386 |

| | | | |
|---|---|---|---|
| O | -3.919295619467 | 1.319242372321 | 1.625471028841 |
| C | -2.245059945036 | 0.496320552318 | -3.333660561190 |
| H | -1.592972239125 | -0.221816570793 | -3.838489155967 |
| H | -2.926894230224 | -0.066046108153 | -2.690823219765 |
| H | -2.830512504943 | 1.008908106443 | -4.097100055510 |
| C | -0.239013806938 | 3.600848092953 | -5.425767376854 |
| H | 0.629052132220 | 3.987375358978 | -4.882782645951 |
| H | -0.825460865170 | 4.448219282822 | -5.784650702523 |
| C | 0.220053788337 | 2.770905179032 | -6.624762059501 |
| H | 0.828593125646 | 1.916366533706 | -6.291953133631 |
| H | -0.657480666745 | 2.375014579459 | -7.156424763250 |
| C | -4.308045650405 | 1.900033911407 | 2.873653184341 |
| H | -4.931593929691 | 2.784550184388 | 2.701986417074 |
| H | -3.421621302513 | 2.206115833580 | 3.442702495046 |
| C | -5.088103420210 | 0.864025156045 | 3.672203007791 |
| H | -5.986715193441 | 0.560399244018 | 3.115672284447 |
| H | -4.467046648880 | -0.030467723991 | 3.827395986904 |
| O | 0.972759858777 | 3.645922169841 | -7.450454969042 |
| C | 1.425426828916 | 3.065083871106 | -8.667672101312 |
| H | 2.089822354150 | 2.209247103224 | -8.483538217802 |
| H | 0.584246835821 | 2.729394936923 | -9.289995152175 |
| H | 1.978867237365 | 3.837719170496 | -9.201581790089 |
| O | -5.429347016381 | 1.473891500252 | 4.907356203835 |
| C | -6.167205327747 | 0.634376305245 | 5.787359912897 |
| H | -7.123191663499 | 0.325373485173 | 5.341958412112 |
| H | -5.597632430681 | -0.266229846699 | 6.056486428421 |

| | | | |
|---|---|---|---|
| H | -6.364514656828 | 1.214576252660 | 6.688891843705 |

Cam_TS1-F200

($E_F$ = -928.86369367 a.u.; $G_F$ = -928.47748199 a.u.)

0 1

| | | | |
|---|---|---|---|
| C | -1.890142282754 | 0.894425976752 | -2.905430126653 |
| C | -0.961122658624 | 2.059792771233 | -2.673006219212 |
| C | -2.521317714781 | 1.746730093744 | 0.350960701555 |
| C | -2.748601580793 | 0.624684361260 | -0.597502211296 |
| C | -1.452375847275 | -0.215428323959 | -0.777980923609 |
| C | -1.235068064298 | -0.360322281973 | -2.291231822229 |
| H | 0.084258583469 | 1.886340804250 | -2.425434318883 |
| H | -1.630572844088 | 1.806656721818 | 0.970318780256 |
| H | -3.546303684844 | 0.001161390162 | -0.167580928698 |
| H | -0.604650018640 | 0.315389649485 | -0.335361263080 |
| H | -1.521781356860 | -1.180896982046 | -0.271976657606 |
| H | -1.734955898240 | -1.253641649374 | -2.673682158048 |
| H | -0.179246697679 | -0.450240854500 | -2.562427110654 |
| C | -3.225162399672 | 1.020626283975 | -2.059628228307 |
| C | -4.283320252329 | -0.006526022747 | -2.515066529050 |
| H | -4.643734461551 | 0.208680824538 | -3.523590112692 |
| H | -3.910661114534 | -1.033992291156 | -2.499022096936 |
| H | -5.145167265620 | 0.039791892950 | -1.843254363507 |
| C | -3.862895460358 | 2.411829628305 | -2.144098375395 |
| H | -4.143555497998 | 2.637427488306 | -3.176965958549 |
| H | -4.762426059699 | 2.453697009740 | -1.525978442930 |

| | | | |
|---|---|---|---|
| H | -3.182741029068 | 3.188785807154 | -1.800044237077 |
| O | -1.224315853970 | 3.212455576386 | -3.365661789936 |
| O | -3.650678754425 | 2.344735074165 | 0.844622449793 |
| C | -2.129485660518 | 0.735926229360 | -4.421518974905 |
| H | -1.172369147262 | 0.664207617766 | -4.945950816768 |
| H | -2.698623255736 | -0.168733306837 | -4.643288081352 |
| H | -2.665618430619 | 1.595925358936 | -4.826244118550 |
| C | -0.103699165864 | 3.985279320900 | -3.797607506030 |
| H | 0.710984416109 | 3.940158682709 | -3.066135460385 |
| H | -0.442232863729 | 5.019153307147 | -3.875808419256 |
| C | 0.380730045017 | 3.498692359788 | -5.160636241592 |
| H | 0.713989376116 | 2.451960451265 | -5.087369259727 |
| H | -0.453716451150 | 3.536336579724 | -5.876150542719 |
| C | -3.565970856766 | 2.989095898426 | 2.115936022847 |
| H | -4.196066213606 | 3.879309197095 | 2.074208800203 |
| H | -2.539239832424 | 3.300666552867 | 2.334597220000 |
| C | -4.083032127954 | 2.047613055662 | 3.199374285240 |
| H | -5.095720756861 | 1.713954542271 | 2.929267741978 |
| H | -3.439309131673 | 1.156297493916 | 3.259561838764 |
| O | 1.442187356019 | 4.344017907963 | -5.565904405222 |
| C | 1.929469009614 | 4.051025825306 | -6.867706982055 |
| H | 2.325032650750 | 3.027263964368 | -6.928883502810 |
| H | 1.143292464846 | 4.168741032992 | -7.626645348192 |
| H | 2.734362651247 | 4.756420016201 | -7.075592986015 |
| O | -4.088178508508 | 2.759376618490 | 4.423916765661 |
| C | -4.654902523159 | 2.025729845215 | 5.499975190351 |

| | | | |
|---|---|---|---|
| H | -5.702596323131 | 1.761810727893 | 5.298112313266 |
| H | -4.093055084209 | 1.102073961038 | 5.698746406222 |
| H | -4.611906938967 | 2.664842003386 | 6.382263916117 |

Cam_Int1-F200

($E_F$ = -928.87803746 a.u.; $G_F$ = -928.49289924 a.u.)

0 1

| | | | |
|---|---|---|---|
| C | -1.332263774510 | 1.310134031651 | -2.501813692475 |
| C | -0.566558065269 | 2.194718768321 | -3.432198309796 |
| C | -2.908386578901 | 1.467309689882 | 1.001775103429 |
| C | -2.428102324627 | 1.005423345367 | -0.323694978848 |
| C | -1.069607754409 | 0.268332255665 | -0.245953549575 |
| C | -0.351886749385 | 0.512114206759 | -1.596815714866 |
| H | 0.323544962742 | 2.724275540969 | -3.094347505106 |
| H | -2.254312583548 | 2.002631173594 | 1.689411857938 |
| H | -3.190656794173 | 0.320919842844 | -0.710145535114 |
| H | -0.473582756663 | 0.666113096036 | 0.580490425862 |
| H | -1.217720500622 | -0.793276247569 | -0.038678351171 |
| H | -0.053068383272 | -0.418769291535 | -2.083362066382 |
| H | 0.564801466263 | 1.086777131923 | -1.438734000519 |
| C | -2.216447159884 | 2.104617314273 | -1.436039753447 |
| C | -3.547795432400 | 2.616982840069 | -1.992085421195 |
| H | -3.376052460431 | 3.285659995531 | -2.839601221744 |
| H | -4.191820037834 | 1.800731951153 | -2.325344074310 |
| H | -4.090606115183 | 3.162330163658 | -1.215855739556 |
| C | -1.432169702680 | 3.302549405594 | -0.868324230842 |

| | | | |
|---|---|---|---|
| H | -1.261360164475 | 4.052346599491 | -1.644128687166 |
| H | -2.001031914178 | 3.778180360041 | -0.065545906694 |
| H | -0.460051603967 | 3.013697685824 | -0.460712409110 |
| O | -1.295440816061 | 2.826262993690 | -4.408123358195 |
| O | -4.244097134754 | 1.769126673069 | 1.081890335176 |
| C | -2.189918177593 | 0.332231632797 | -3.337889159126 |
| H | -1.533237370407 | -0.284420506365 | -3.957102240244 |
| H | -2.780435391780 | -0.335592125357 | -2.707533158879 |
| H | -2.866426962288 | 0.868719524823 | -4.002818461326 |
| C | -0.567252781459 | 3.688727523214 | -5.281617027066 |
| H | 0.165577869857 | 4.280410932409 | -4.720041374111 |
| H | -1.296227250176 | 4.368936398223 | -5.723431405876 |
| C | 0.135842244275 | 2.906533369641 | -6.387424362315 |
| H | 0.870327347033 | 2.213783124243 | -5.950388093930 |
| H | -0.603873433831 | 2.310508340148 | -6.942190931837 |
| C | -4.705457519359 | 2.275515088329 | 2.334406712719 |
| H | -5.642689359804 | 2.794890253910 | 2.131368223325 |
| H | -3.990772979455 | 2.996650626021 | 2.748569492684 |
| C | -4.953334769056 | 1.154155714901 | 3.339703963882 |
| H | -5.661971881101 | 0.429544361638 | 2.911985168093 |
| H | -4.013638642449 | 0.623300502816 | 3.553379058952 |
| O | 0.763002624976 | 3.860220863241 | -7.227012277008 |
| C | 1.415935897310 | 3.288566306790 | -8.350812127704 |
| H | 2.212488271788 | 2.595683919391 | -8.044924859945 |
| H | 0.708515452669 | 2.746828241358 | -8.994329410718 |
| H | 1.856412554150 | 4.109330604500 | -8.917444829966 |

| | | | |
|---|---|---|---|
| O | -5.478564698868 | 1.760909424324 | 4.507518144498 |
| C | -5.824573448728 | 0.833641484190 | 5.525596210118 |
| H | -6.597416147526 | 0.130855721468 | 5.183726072160 |
| H | -4.950428452023 | 0.257346151325 | 5.860140152501 |
| H | -6.213480538415 | 1.409969203650 | 6.365446818266 |

Cam-F200

($E_F$ = -928.95670907 a.u.; $G_F$ = -928.56276360 a.u.)

0 1

| | | | |
|---|---|---|---|
| C | -2.161375283219 | 0.619478064568 | -2.756064885142 |
| C | -1.433651214442 | 1.810120370431 | -2.067464131177 |
| C | -1.939008624571 | 1.723823083177 | -0.565098499421 |
| C | -2.900149673793 | 0.506525828165 | -0.610903328263 |
| C | -1.994268170264 | -0.741468082364 | -0.722190102952 |
| C | -1.474136316407 | -0.654040242957 | -2.189339826678 |
| H | -0.350565772745 | 1.648776478600 | -2.102306831617 |
| H | -1.091133045355 | 1.511402683127 | 0.098965567512 |
| H | -3.602473971677 | 0.479809793778 | 0.221657639262 |
| H | -1.185003400464 | -0.723179293450 | 0.012286008605 |
| H | -2.559096420407 | -1.658498050691 | -0.548701882868 |
| H | -1.758208290013 | -1.532666022186 | -2.771739730608 |
| H | -0.385039247028 | -0.576806361415 | -2.247698388150 |
| C | -3.548746575459 | 0.592247521007 | -2.021932724199 |
| C | -4.418063853187 | -0.628757500954 | -2.373349293646 |
| H | -4.797138773109 | -0.539211948732 | -3.395410917432 |
| H | -3.901501270234 | -1.585455800922 | -2.298878109060 |

| | | | |
|---|---|---|---|
| H | -5.285442624470 | -0.669108374864 | -1.707313769693 |
| C | -4.424961870568 | 1.837252057172 | -2.242956346363 |
| H | -4.764369965744 | 1.873218792288 | -3.282825296528 |
| H | -5.314303375218 | 1.774944395277 | -1.607220059180 |
| H | -3.915302067416 | 2.768641159088 | -2.020587784623 |
| O | -1.734173039676 | 3.044204199546 | -2.694946873163 |
| O | -2.529792846942 | 2.947221218453 | -0.155409776164 |
| C | -2.168334047357 | 0.696999074786 | -4.272247609282 |
| H | -1.147736904452 | 0.673602442184 | -4.667663620034 |
| H | -2.709555869554 | -0.151464386076 | -4.699877153056 |
| H | -2.636506433145 | 1.620834602280 | -4.613813381658 |
| C | -0.644237562184 | 3.936328486121 | -2.882414821385 |
| H | 0.093530917969 | 3.851234254844 | -2.075938231025 |
| H | -1.049105387998 | 4.950196856562 | -2.858649669708 |
| C | 0.020099715174 | 3.684674125155 | -4.234741203456 |
| H | 0.422117910376 | 2.660047522600 | -4.274226626692 |
| H | -0.738070734268 | 3.773993414868 | -5.026684363644 |
| C | -2.433676098042 | 3.253443817633 | 1.228010712076 |
| H | -2.659437573321 | 4.317899467098 | 1.318391736181 |
| H | -1.411432983489 | 3.089421914504 | 1.596241232634 |
| C | -3.423317599910 | 2.483830425670 | 2.105927896696 |
| H | -4.428999260403 | 2.554920357657 | 1.664933349023 |
| H | -3.153554243130 | 1.419478826852 | 2.166448589585 |
| O | 1.054552996413 | 4.637537722949 | -4.408692434351 |
| C | 1.692470446200 | 4.544240484875 | -5.673620438283 |
| H | 2.163086377107 | 3.560812158111 | -5.816580987827 |

| | | | |
|---|---|---|---|
| H | 0.982743095283 | 4.714276629070 | -6.495673527667 |
| H | 2.463596881435 | 5.314657084786 | -5.703874572505 |
| O | -3.384340791088 | 3.074461763591 | 3.394625438227 |
| C | -4.259581715152 | 2.453731718765 | 4.324129568250 |
| H | -5.306301576545 | 2.515026383776 | 3.994035819447 |
| H | -4.002379576301 | 1.395585457223 | 4.475975805839 |
| H | -4.153208756317 | 2.984744405055 | 5.270543492969 |

Pin_TS1-F3500

($E_F$ = -929.91038704 a.u.; $G_F$ = -929.52340277 a.u.)

0 1

| | | | |
|---|---|---|---|
| C | -1.788859080878 | 1.972638183711 | -0.150762451333 |
| C | -1.660790665608 | 3.442146390478 | 0.415498481783 |
| C | -2.116036173898 | 3.886724214271 | 1.807071338883 |
| C | -1.740216687769 | 2.004217386963 | 2.797999062802 |
| C | -0.614079574645 | 1.310063022351 | 2.042247919281 |
| C | -0.431298749235 | 1.697616873777 | 0.573998446770 |
| H | -2.030874220241 | 4.173476305205 | -0.307150647331 |
| H | -2.723672194965 | 1.742594768603 | 2.437588535967 |
| H | 0.317876972344 | 1.485286389648 | 2.589189887948 |
| H | -0.812445655623 | 0.230194645411 | 2.118724736981 |
| H | 0.258405515502 | 1.007052276719 | 0.079107328401 |
| C | -0.121573861017 | 3.212086485932 | 0.473938979168 |
| H | 0.401674435784 | 3.496648727451 | -0.437559669751 |
| H | 0.378862751714 | 3.664683596080 | 1.329470213705 |
| C | -1.587301808355 | 2.051508482565 | -1.680095400838 |

| | | | |
|---|---|---|---|
| H | -0.779674213617 | 2.721044511659 | -1.977700645178 |
| H | -1.360490727558 | 1.058410526137 | -2.079427090256 |
| H | -2.504278263603 | 2.405496137245 | -2.162075102884 |
| C | -3.011303108796 | 1.068423628143 | 0.058285068590 |
| H | -2.928255391965 | 0.186198970588 | -0.583825332337 |
| H | -3.149479596736 | 0.703089414855 | 1.072258666323 |
| H | -3.924208941232 | 1.596327188165 | -0.235161840341 |
| C | -3.601686907254 | 4.017266525494 | 2.055221493318 |
| H | -4.018599544901 | 4.806625277509 | 1.418657059786 |
| H | -4.141277122119 | 3.095301857663 | 1.833271917866 |
| H | -3.782681487653 | 4.292314965046 | 3.095859043352 |
| O | -1.514844229182 | 5.228772982216 | 2.047595216971 |
| O | -1.662007607723 | 1.548857363911 | 4.177542261967 |
| C | -1.543935005429 | 6.193693468261 | 0.901252633374 |
| H | -2.519562131739 | 6.143771006741 | 0.411151310236 |
| H | -0.780478544586 | 5.903875436983 | 0.176534299871 |
| C | -1.292791701093 | 7.729989577250 | 1.207799431444 |
| H | -2.062697162314 | 8.111370799613 | 1.889410560982 |
| H | -0.310223769016 | 7.866391957877 | 1.675312035913 |
| C | -2.436302974544 | 0.315504355370 | 4.493023149441 |
| H | -2.761117523822 | -0.160344823930 | 3.563266556773 |
| H | -3.325035695109 | 0.615190655005 | 5.054349578262 |
| C | -1.677324462959 | -0.792909810028 | 5.349853722219 |
| H | -0.802569499518 | -1.146258241916 | 4.789019449063 |
| H | -1.329411690361 | -0.346007795895 | 6.289352329643 |
| O | -1.353097171614 | 8.419861380288 | -0.080861806175 |

| | | | |
|---|---|---|---|
| C | -1.159918707693 | 9.892328203934 | -0.238773267200 |
| H | -1.925563201049 | 10.449301327529 | 0.311182218888 |
| H | -0.163066712944 | 10.195218499505 | 0.097960954863 |
| H | -1.265068996302 | 10.060375272762 | -1.308412325130 |
| O | -2.598826141292 | -1.893418217217 | 5.612608578324 |
| C | -2.209820469309 | -3.098635871209 | 6.406091982666 |
| H | -1.387205207395 | -3.635816474060 | 5.922652724167 |
| H | -1.926177127539 | -2.819845015802 | 7.426213937770 |
| H | -3.108027658329 | -3.712191235904 | 6.417066809191 |

Pin_Int1-F3500

($E_F$ = -930.25646431 a.u.; $G_F$ = -929.87912396 a.u.)

0 1

| | | | |
|---|---|---|---|
| C | -2.024755013146 | 2.759570810052 | 0.914339921068 |
| C | -1.260729971035 | 4.185709415807 | 1.080739477610 |
| C | -1.886810529935 | 5.554774898302 | 0.747655872699 |
| C | -1.430237641554 | -0.343572432145 | 3.222833641250 |
| C | -1.764629872728 | 1.170196417570 | 3.274876290929 |
| C | -1.205449209407 | 2.194271070369 | 2.165811159454 |
| H | -0.334577200975 | 4.112719730983 | 0.502915455403 |
| H | -1.497610327026 | -0.885295519626 | 2.280045558150 |
| H | -2.857365834079 | 1.291890977267 | 3.330913237032 |
| H | -1.393250313797 | 1.535482379415 | 4.240112481869 |
| H | -0.261177725695 | 1.790016086381 | 1.786929187461 |
| C | -0.970242166395 | 3.697796424506 | 2.564064304122 |
| H | 0.003408653309 | 3.956201090845 | 2.987570781591 |

| | | | |
|---|---|---|---|
| H | -1.746022631258 | 4.043232310482 | 3.252662161840 |
| C | -1.765853609270 | 2.062569123736 | -0.416078501049 |
| H | -0.693832742845 | 1.983592611626 | -0.617870443930 |
| H | -2.186327800979 | 1.050736465780 | -0.416115997095 |
| H | -2.224692704500 | 2.619521157629 | -1.239948745266 |
| C | -3.526321354713 | 2.850502242816 | 1.180696453883 |
| H | -3.980076243200 | 1.855650737549 | 1.135546411055 |
| H | -3.755493126420 | 3.268683871585 | 2.163233432841 |
| H | -4.007820833343 | 3.479765837241 | 0.426862093859 |
| C | -2.873346078650 | 6.185395620519 | 1.680056188343 |
| H | -3.686639690732 | 6.699207865847 | 1.157061150484 |
| H | -3.327908416282 | 5.429756077123 | 2.318458580905 |
| H | -2.399218618770 | 6.927921423106 | 2.342411267870 |
| O | -1.204770346591 | 6.432852383226 | -0.151631195350 |
| O | -1.683794078146 | -1.120026440338 | 4.372878476056 |
| C | -1.842248553379 | 7.718161359380 | -0.533390927082 |
| H | -2.045438549000 | 8.306085126818 | 0.364158250557 |
| H | -2.793404532121 | 7.503249279157 | -1.029041661686 |
| C | -0.986229006007 | 8.640435491882 | -1.500851591162 |
| H | -0.018993474474 | 8.858287665448 | -1.030843363460 |
| H | -0.803363377726 | 8.114658380675 | -2.446027086224 |
| C | -1.478781788554 | -2.584677612848 | 4.297565091689 |
| H | -0.441623820209 | -2.779205146361 | 4.010689382331 |
| H | -2.138364763159 | -2.992788027803 | 3.525125983176 |
| C | -1.773121430202 | -3.348153645915 | 5.659474082858 |
| H | -1.112855949827 | -2.949693344682 | 6.440009369615 |

| | | | |
|---|---|---|---|
| H | -2.812630130400 | -3.159873618564 | 5.956418007732 |
| O | -1.737129297114 | 9.870099240912 | -1.731432418794 |
| C | -1.233480873016 | 10.974070430650 | -2.603107007083 |
| H | -0.295191082338 | 11.382541430312 | -2.213749554116 |
| H | -1.092101201303 | 10.626624954307 | -3.631748750526 |
| H | -2.019111681990 | 11.725294643971 | -2.562162297110 |
| O | -1.542818812124 | -4.774173032030 | 5.468726717425 |
| C | -1.728708145342 | -5.767227810667 | 6.570289445192 |
| H | -1.049925518870 | -5.557541065658 | 7.403370855463 |
| H | -2.765206821113 | -5.769413218352 | 6.923091006359 |
| H | -1.482466222590 | -6.722688180086 | 6.112376534068 |

Pin-F3500

($E_F$ = -929.91631340 a.u.; $G_F$ = -929.52715028 a.u.)

0 1

| | | | |
|---|---|---|---|
| C | -1.755403063013 | 1.831923798647 | -0.152824005252 |
| C | -1.793449873457 | 3.325614213053 | 0.389680747893 |
| C | -2.135265050144 | 3.653325345819 | 1.877666079295 |
| C | -1.711439996733 | 2.199472714086 | 2.622848391838 |
| C | -0.453534126966 | 1.548705895811 | 2.027132771990 |
| C | -0.337722716649 | 1.776092049336 | 0.509647413332 |
| H | -2.343467384141 | 4.003310176347 | -0.264126970712 |
| H | -2.565547341760 | 1.670878884356 | 2.219289981350 |
| H | 0.436188220505 | 1.930575212388 | 2.536659320446 |
| H | -0.513170047057 | 0.478494551436 | 2.255564119519 |
| H | 0.414906340611 | 1.125081665700 | 0.055492217922 |

| | | | |
|---|---|---|---|
| C | -0.233594480143 | 3.300381992114 | 0.286742981488 |
| H | 0.142534935614 | 3.582796302804 | -0.694222841506 |
| H | 0.298563502095 | 3.865413270452 | 1.050040977158 |
| C | -1.674390876833 | 1.896731674422 | -1.693111742808 |
| H | -1.027944691869 | 2.688560053016 | -2.070705962238 |
| H | -1.304580414630 | 0.945781046427 | -2.088607964407 |
| H | -2.673839727286 | 2.064035652516 | -2.107332678267 |
| C | -2.816352157673 | 0.756978680800 | 0.163124065542 |
| H | -2.844204683915 | 0.015321606222 | -0.639539209532 |
| H | -2.632581990029 | 0.201894057715 | 1.082014674179 |
| H | -3.816204158316 | 1.197574078537 | 0.224174115018 |
| C | -3.618454941216 | 3.861569150675 | 2.171481870864 |
| H | -4.007681952337 | 4.730227150245 | 1.638016499500 |
| H | -4.213348459368 | 2.994374990175 | 1.874251807653 |
| H | -3.753749778138 | 4.023736157486 | 3.242792104914 |
| O | -1.473814614108 | 5.002079494530 | 2.151586220832 |
| O | -1.759948349222 | 1.875511685958 | 4.057602425385 |
| C | -1.631630545683 | 6.018973225642 | 1.073969406607 |
| H | -2.678200232846 | 6.067522911573 | 0.761927496798 |
| H | -1.037637142731 | 5.724220433745 | 0.207181541779 |
| C | -1.196585284753 | 7.516091801866 | 1.373844215361 |
| H | -1.812162759233 | 7.943029621661 | 2.174759041081 |
| H | -0.144986456184 | 7.547426508200 | 1.684138828592 |
| C | -2.485399818675 | 0.634371405029 | 4.421569161306 |
| H | -2.959993945122 | 0.210896503607 | 3.531267434713 |
| H | -3.276825536298 | 0.932985292949 | 5.112954893287 |

| | | | |
|---|---|---|---|
| C | -1.683107559889 | -0.547625198821 | 5.135822909463 |
| H | -0.920639870095 | -0.945966006157 | 4.453851430086 |
| H | -1.179254954826 | -0.154010956882 | 6.027280737636 |
| O | -1.389014745854 | 8.253956260259 | 0.124294886268 |
| C | -1.089619487134 | 9.707899155630 | -0.031912413622 |
| H | -1.707751559037 | 10.310692099833 | 0.641598431345 |
| H | -0.029509255162 | 9.912186384368 | 0.151213389091 |
| H | -1.341871210164 | 9.921185426321 | -1.068453505507 |
| O | -2.637905272001 | -1.590291793567 | 5.500375548563 |
| C | -2.241075706724 | -2.840630091119 | 6.215539729017 |
| H | -1.531772891182 | -3.423964498186 | 5.619152884977 |
| H | -1.806204932072 | -2.610170552038 | 7.193634148466 |
| H | -3.173957371798 | -3.387214452182 | 6.335389510170 |

Pin_TS1-F3000

($E_F$ = -929.74250747 a.u.; $G_F$ = -929.35546060 a.u.)

0 1

| | | | |
|---|---|---|---|
| C | -1.780589962779 | 1.993924435061 | -0.162134778523 |
| C | -1.605184197573 | 3.458423937546 | 0.394899654051 |
| C | -2.122710380079 | 3.940356106535 | 1.743280996882 |
| C | -1.753000134428 | 1.932410670540 | 2.846380130726 |
| C | -0.673494617922 | 1.221685894463 | 2.046369398920 |
| C | -0.457530969530 | 1.655413736058 | 0.597991369481 |
| H | -1.895204939111 | 4.195640212195 | -0.359322823414 |
| H | -2.764911733632 | 1.733381640848 | 2.523809763024 |
| H | 0.266250251418 | 1.325193066690 | 2.598629495276 |

| | | | |
|---|---|---|---|
| H | -0.924936513604 | 0.148774849487 | 2.078635659185 |
| H | 0.217472545954 | 0.954680284237 | 0.096832354608 |
| C | -0.084024412345 | 3.159026080089 | 0.533032663742 |
| H | 0.499417022061 | 3.428233822232 | -0.346607845640 |
| H | 0.388418598268 | 3.581181056153 | 1.419746746899 |
| C | -1.526568648976 | 2.044430088160 | -1.684285279133 |
| H | -0.659571749867 | 2.648483674168 | -1.955355117368 |
| H | -1.361422132087 | 1.034124930811 | -2.070660810742 |
| H | -2.396638607312 | 2.463688954989 | -2.199682421202 |
| C | -3.047051092822 | 1.152054027427 | 0.024894191167 |
| H | -2.944141956361 | 0.210045374749 | -0.523112551321 |
| H | -3.280921088702 | 0.893753727642 | 1.053703755755 |
| H | -3.912481877293 | 1.676734891919 | -0.392074803058 |
| C | -3.612325574411 | 4.028293409719 | 1.961182699513 |
| H | -4.039428639805 | 4.787934747819 | 1.292467724473 |
| H | -4.122084316141 | 3.086952519582 | 1.756379198751 |
| H | -3.822319693361 | 4.331878970036 | 2.988330884655 |
| O | -1.552521661156 | 5.265471861948 | 2.004148981811 |
| O | -1.623536135044 | 1.508713963098 | 4.209718894482 |
| C | -1.523778460846 | 6.216505328923 | 0.859467254355 |
| H | -2.458582749056 | 6.138893539995 | 0.297892970885 |
| H | -0.700233700695 | 5.949545226159 | 0.193315397927 |
| C | -1.348682329468 | 7.743741811991 | 1.195493920912 |
| H | -2.175168303503 | 8.092406327843 | 1.827400123580 |
| H | -0.404482433572 | 7.913499612513 | 1.727714043314 |
| C | -2.448720526275 | 0.357507797892 | 4.635446211696 |

| | | | |
|---|---|---|---|
| H | -2.999471029207 | -0.039009989912 | 3.777430089416 |
| H | -3.170687571226 | 0.720680513932 | 5.370999198053 |
| C | -1.650807631631 | -0.838518566479 | 5.295223999535 |
| H | -0.951821715446 | -1.261395528091 | 4.561467352397 |
| H | -1.070329856637 | -0.458537327406 | 6.145647662723 |
| O | -1.350703014216 | 8.429572189467 | -0.087997007996 |
| C | -1.204339887142 | 9.895732948721 | -0.200439195276 |
| H | -2.024317771925 | 10.414779537000 | 0.308395762055 |
| H | -0.244965555845 | 10.229612992448 | 0.210361713767 |
| H | -1.242433690338 | 10.094844487946 | -1.269670409337 |
| O | -2.607609237442 | -1.839459970878 | 5.729281396648 |
| C | -2.168142865756 | -3.078489424872 | 6.407190879665 |
| H | -1.513675632348 | -3.671317065317 | 5.758264979348 |
| H | -1.649850208796 | -2.850724177744 | 7.345366343294 |
| H | -3.087490905200 | -3.624245645353 | 6.610371524224 |

Pin-F3000

($E_F$ = -929.75639076 a.u.; $G_F$ = -929.36554571 a.u.)

0 1

| | | | |
|---|---|---|---|
| C | -1.736922912855 | 1.787207352425 | -0.158617569132 |
| C | -1.802775098964 | 3.288611689327 | 0.361098079066 |
| C | -2.149263340135 | 3.611182569245 | 1.851129810905 |
| C | -1.706382106964 | 2.209224171799 | 2.603478735772 |
| C | -0.430577288225 | 1.570225567244 | 2.028472073431 |
| C | -0.318866938610 | 1.768639163582 | 0.505771509583 |
| H | -2.366624922724 | 3.950144026014 | -0.297950702797 |

| | | | |
|---|---|---|---|
| H | -2.541937132722 | 1.630892357420 | 2.227200890849 |
| H | 0.448977031441 | 1.986182149018 | 2.528812115148 |
| H | -0.464572198194 | 0.504939321677 | 2.280999211193 |
| H | 0.445766402368 | 1.123141542157 | 0.064034734192 |
| C | -0.242692214080 | 3.290235311249 | 0.255248307352 |
| H | 0.125605663560 | 3.561956070942 | -0.731649383725 |
| H | 0.282689438181 | 3.876144691102 | 1.007349421752 |
| C | -1.656715353972 | 1.826580847319 | -1.699702831322 |
| H | -1.027156576679 | 2.625134298703 | -2.091262260642 |
| H | -1.266901101541 | 0.876875311501 | -2.078730929277 |
| H | -2.659374813412 | 1.965494163670 | -2.116635205016 |
| C | -2.776518017581 | 0.696703622677 | 0.175387976840 |
| H | -2.780882999530 | -0.063963855883 | -0.609846361407 |
| H | -2.589021409444 | 0.167692907940 | 1.109001810053 |
| H | -3.786229971795 | 1.116499313968 | 0.217514182501 |
| C | -3.639055218977 | 3.803169949997 | 2.132713929112 |
| H | -4.033649365675 | 4.672570502465 | 1.604874114392 |
| H | -4.221251677176 | 2.932726652949 | 1.820329859227 |
| H | -3.784949624005 | 3.952591785116 | 3.204591513079 |
| O | -1.493631553802 | 4.938590140219 | 2.154319975210 |
| O | -1.748604842887 | 1.981481775663 | 4.042575247161 |
| C | -1.659640459954 | 5.965146314339 | 1.104706990570 |
| H | -2.709883282153 | 6.031627986641 | 0.807461781057 |
| H | -1.082346195510 | 5.686288122298 | 0.220999220708 |
| C | -1.205627912780 | 7.435555839521 | 1.438078791468 |
| H | -1.804242281926 | 7.852768832825 | 2.257407388767 |

| | | | |
|---|---|---|---|
| H | -0.148854697951 | 7.450083660681 | 1.733511980938 |
| C | -2.493363627065 | 0.802639067949 | 4.505603569860 |
| H | -3.189604746743 | 0.474510874830 | 3.726324983062 |
| H | -3.083660347522 | 1.143134597304 | 5.358877596145 |
| C | -1.689405202532 | -0.472864894679 | 5.000362626581 |
| H | -1.143177399677 | -0.929228976801 | 4.163915810197 |
| H | -0.961289054599 | -0.168109643684 | 5.763137386721 |
| O | -1.410196100641 | 8.191593032851 | 0.210195257324 |
| C | -1.090033548141 | 9.629138080151 | 0.102307029730 |
| H | -1.687257766613 | 10.221639211603 | 0.804571669914 |
| H | -0.024153270994 | 9.811552295942 | 0.280046219410 |
| H | -1.347984052576 | 9.892865720961 | -0.921600286281 |
| O | -2.652518557017 | -1.411012692598 | 5.548996641353 |
| C | -2.231039459310 | -2.705400545237 | 6.125926954294 |
| H | -1.734560018145 | -3.329163258879 | 5.374001338472 |
| H | -1.562399023927 | -2.556070654569 | 6.981100053531 |
| H | -3.155611459375 | -3.177796402641 | 6.452055464496 |

Pin_Int1-F3000

($E_F$ = -930.03841463 a.u.; $G_F$ = -929.65981688 a.u.)

0 1

| | | | |
|---|---|---|---|
| C | -2.009451465239 | 2.752724406610 | 0.926491946305 |
| C | -1.263511801911 | 4.182119311808 | 1.094015421074 |
| C | -1.907093503713 | 5.532207207769 | 0.765207645891 |
| C | -1.448561669085 | -0.306287354864 | 3.194842097569 |
| C | -1.743182752444 | 1.201798467141 | 3.286618507021 |

| | | | |
|---|---|---|---|
| C | -1.188735877500 | 2.206599986082 | 2.179948880026 |
| H | -0.337249737272 | 4.123312454286 | 0.514658298988 |
| H | -1.570957683381 | -0.835470329111 | 2.250392196087 |
| H | -2.829989412289 | 1.353929995226 | 3.379818145923 |
| H | -1.330617455984 | 1.540879302902 | 4.245049148815 |
| H | -0.241033477041 | 1.804229071971 | 1.807127128710 |
| C | -0.965628699816 | 3.708035241960 | 2.575024494549 |
| H | 0.006753099432 | 3.976485142680 | 2.995110399677 |
| H | -1.742945882205 | 4.048441107997 | 3.264498894573 |
| C | -1.736266993890 | 2.054734579808 | -0.400812374844 |
| H | -0.662401446161 | 1.986573276162 | -0.596779840993 |
| H | -2.145917624877 | 1.038480896261 | -0.400658749463 |
| H | -2.196570423902 | 2.604579468419 | -1.228644253113 |
| C | -3.513417366009 | 2.825072195680 | 1.185369073714 |
| H | -3.953104237768 | 1.823662646649 | 1.145689801026 |
| H | -3.753122411338 | 3.247283482988 | 2.163582634553 |
| H | -4.000068629960 | 3.442329799100 | 0.424961237686 |
| C | -2.897971737381 | 6.157223048362 | 1.697175675010 |
| H | -3.712850241649 | 6.668342211506 | 1.174318722858 |
| H | -3.350016347903 | 5.398290294405 | 2.333243004003 |
| H | -2.427623954010 | 6.900232116665 | 2.361828254235 |
| O | -1.220341691388 | 6.405499789200 | -0.123062857912 |
| O | -1.671459815167 | -1.078994382556 | 4.344982181320 |
| C | -1.864911467348 | 7.671490530110 | -0.513399886536 |
| H | -2.086405803328 | 8.265456364807 | 0.376322943827 |
| H | -2.807835345248 | 7.448383611028 | -1.022201072884 |

| | | | |
|---|---|---|---|
| C | -0.998873073668 | 8.579226334924 | -1.464479797311 |
| H | -0.038475550040 | 8.802514735048 | -0.981923641934 |
| H | -0.799624890541 | 8.051774636400 | -2.405990363289 |
| C | -1.504314469236 | -2.536149725209 | 4.243553165256 |
| H | -0.486102738636 | -2.755715894568 | 3.909034670605 |
| H | -2.207466927030 | -2.925698213392 | 3.499779775939 |
| C | -1.756122741797 | -3.283084829232 | 5.607938638185 |
| H | -1.050648198015 | -2.906076694485 | 6.359781494822 |
| H | -2.775701289774 | -3.068227989583 | 5.953883925240 |
| O | -1.752590076886 | 9.796478882330 | -1.704005963947 |
| C | -1.224340746783 | 10.875298687201 | -2.565859615323 |
| H | -0.288908746016 | 11.279196172516 | -2.162763827603 |
| H | -1.062033775404 | 10.518515208441 | -3.589081282181 |
| H | -1.997433624561 | 11.641078175502 | -2.555305563301 |
| O | -1.572970824026 | -4.705257689636 | 5.396846187957 |
| C | -1.736788681304 | -5.666792139456 | 6.508896089419 |
| H | -1.016361281317 | -5.467687635173 | 7.310082108432 |
| H | -2.755528305713 | -5.633297478090 | 6.910987273600 |
| H | -1.541962662518 | -6.640542546366 | 6.063877800067 |

Pin_TS1-F2500

($E_F$ = -929.57531037 a.u.; $G_F$ = -929.18840772 a.u.)

0 1

| | | | |
|---|---|---|---|
| C | -1.760991427530 | 1.988152154243 | -0.171371315278 |
| C | -1.581781605626 | 3.457462480313 | 0.369367136940 |
| C | -2.138636934646 | 3.959979834325 | 1.689802077818 |

| | | | |
|---|---|---|---|
| C | -1.745925099792 | 1.883213255044 | 2.869399530913 |
| C | -0.677921320757 | 1.182333953194 | 2.049191542916 |
| C | -0.452566975153 | 1.640703282245 | 0.609987923324 |
| H | -1.835866262150 | 4.186852179140 | -0.406657023447 |
| H | -2.768909408235 | 1.703362689709 | 2.567663986538 |
| H | 0.263560268800 | 1.267680680062 | 2.601827543170 |
| H | -0.935002456218 | 0.109577304002 | 2.059171890848 |
| H | 0.225685570379 | 0.943566319879 | 0.108050553466 |
| C | -0.068657072432 | 3.142718169992 | 0.554920806274 |
| H | 0.544134806711 | 3.404482777043 | -0.307227607971 |
| H | 0.379526121332 | 3.562656752039 | 1.455141384592 |
| C | -1.482086237244 | 2.015089946144 | -1.689279351714 |
| H | -0.599183866602 | 2.600406375701 | -1.951051871576 |
| H | -1.328340269459 | 0.997523064046 | -2.061213454987 |
| H | -2.335249584891 | 2.444238386866 | -2.224460261037 |
| C | -3.036462784522 | 1.161018517523 | 0.012451563191 |
| H | -2.927057174286 | 0.201082925768 | -0.502422846566 |
| H | -3.293171677706 | 0.938323396435 | 1.044121351182 |
| H | -3.889423655084 | 1.676705903575 | -0.439826201689 |
| C | -3.629339874741 | 4.014178216250 | 1.895146952397 |
| H | -4.070469810547 | 4.753952655341 | 1.211330150568 |
| H | -4.115910611280 | 3.058810393713 | 1.702176036993 |
| H | -3.854773230242 | 4.330152443403 | 2.915414797932 |
| O | -1.588523907372 | 5.272702142998 | 1.969715356595 |
| O | -1.582883712107 | 1.509823560224 | 4.225417836412 |
| C | -1.526278692298 | 6.215830627678 | 0.833982170500 |

| | | | |
|---|---|---|---|
| H | -2.437505905146 | 6.131408432521 | 0.235016725815 |
| H | -0.674149776505 | 5.963769278705 | 0.197758270895 |
| C | -1.386722344284 | 7.728078365825 | 1.198801228204 |
| H | -2.240744332890 | 8.056795153457 | 1.805108832855 |
| H | -0.465037073675 | 7.905927831908 | 1.767564801879 |
| C | -2.442276834274 | 0.449130028866 | 4.763995860468 |
| H | -3.194379297559 | 0.163746794085 | 4.021915948856 |
| H | -2.955852160936 | 0.856779788895 | 5.637629573201 |
| C | -1.671813643392 | -0.842775254036 | 5.219392398079 |
| H | -1.193941393023 | -1.316327597057 | 4.350709105452 |
| H | -0.885787819074 | -0.559183316681 | 5.931767951879 |
| O | -1.353669479450 | 8.421581567975 | -0.072252375089 |
| C | -1.230909193584 | 9.878899666211 | -0.134211868170 |
| H | -2.077779745542 | 10.370681801520 | 0.359110368819 |
| H | -0.294504924704 | 10.218951283970 | 0.323875054784 |
| H | -1.231328815861 | 10.119373686501 | -1.196015475985 |
| O | -2.624517653907 | -1.738403670378 | 5.831262954655 |
| C | -2.172271113411 | -3.019862132660 | 6.380614030296 |
| H | -1.734059787822 | -3.652902986813 | 5.599744758507 |
| H | -1.439700856732 | -2.869629539923 | 7.182558532496 |
| H | -3.067878657728 | -3.492108016808 | 6.780705007987 |

Pin_Int1-F2500

($E_F$ = -929.82282428 a.u.; $G_F$ = -929.44321691 a.u.)

0 1

| | | | |
|---|---|---|---|
| C | -2.390584824247 | 2.918391305804 | 1.166995473015 |

| | | | |
|---|---|---|---|
| C | -1.357035090368 | 4.139023425239 | 0.939184210768 |
| C | -1.747454947108 | 5.607866643490 | 1.019279280021 |
| C | -1.562167476308 | -0.408828711813 | 2.767457882638 |
| C | -1.335120996717 | 1.081229386329 | 3.022376842484 |
| C | -1.200879390332 | 2.074257209742 | 1.800550748574 |
| H | -0.896826613597 | 3.985838537626 | -0.041552649700 |
| H | -2.313327133853 | -0.743039661802 | 2.052398165237 |
| H | -2.114444407150 | 1.462113616899 | 3.701598857228 |
| H | -0.401962725513 | 1.161565188486 | 3.594343177801 |
| H | -0.697559099404 | 1.541771624648 | 0.986603506684 |
| C | -0.464838262152 | 3.434420340867 | 2.034438171919 |
| H | 0.616890978378 | 3.453325474954 | 1.881299443667 |
| H | -0.677361656133 | 3.825454815280 | 3.033197719528 |
| C | -2.993874331176 | 2.358592085460 | -0.116302698406 |
| H | -3.570219746426 | 1.448848781488 | 0.085477477194 |
| H | -3.666654216934 | 3.089257823644 | -0.577828630811 |
| H | -2.214782304521 | 2.111259202369 | -0.843047061319 |
| C | -3.495418680884 | 3.241375750071 | 2.171667534745 |
| H | -4.109098062545 | 2.354492717833 | 2.357687767451 |
| H | -3.104822025642 | 3.574896682576 | 3.135321586698 |
| H | -4.144044943928 | 4.030741447350 | 1.781534117895 |
| C | -1.948543425488 | 6.286104749841 | 2.338866401921 |
| H | -2.765002133288 | 7.015153953446 | 2.324683766691 |
| H | -2.185148716065 | 5.553672046639 | 3.108501436255 |
| H | -1.044041566147 | 6.823285392386 | 2.668365969746 |
| O | -1.410124469865 | 6.428456497795 | -0.083567861316 |

| | | | |
|---|---|---|---|
| O | -1.329616226072 | -1.271854341221 | 3.840645134008 |
| C | -1.849546391389 | 7.823867186691 | -0.073568461644 |
| H | -1.454792565458 | 8.337466992853 | 0.806562322922 |
| H | -2.943018479989 | 7.857692915907 | -0.029261687543 |
| C | -1.382201301394 | 8.636692998070 | -1.323723684897 |
| H | -0.287438565285 | 8.596391390577 | -1.401010269450 |
| H | -1.809559358375 | 8.204443008704 | -2.238016428332 |
| C | -1.635033396314 | -2.687664811674 | 3.651740806886 |
| H | -1.053826371545 | -3.073654793685 | 2.808536303400 |
| H | -2.700049540471 | -2.801733618174 | 3.421258423144 |
| C | -1.303871629615 | -3.537387517515 | 4.922069728788 |
| H | -0.236049124505 | -3.436384847337 | 5.158098750807 |
| H | -1.881480745478 | -3.156943034024 | 5.775344573212 |
| O | -1.837831143835 | 9.994211660841 | -1.132041485459 |
| C | -1.555272809081 | 11.016484188941 | -2.142246769888 |
| H | -0.475669487002 | 11.155305172456 | -2.274721274191 |
| H | -2.011065504465 | 10.758786284661 | -3.105638465044 |
| H | -2.001043504057 | 11.931172317795 | -1.755174521103 |
| O | -1.642257514509 | -4.911398402265 | 4.642566398608 |
| C | -1.422692406544 | -5.934343993577 | 5.668936246068 |
| H | -0.362117450114 | -6.002835279350 | 5.938574377567 |
| H | -2.015539359630 | -5.726767403641 | 6.567667442083 |
| H | -1.750776204673 | -6.868565592197 | 5.216566954971 |

Pin-F2500

($E_F$ = -929.59973253 a.u.; $G_F$ = -929.20834560 a.u.)

0 1

| | | | |
|---|---|---|---|
| C | -1.713355699509 | 1.742462388975 | -0.166603075874 |
| C | -1.810963627847 | 3.250585768532 | 0.328473390846 |
| C | -2.167471224828 | 3.568137253622 | 1.817602530384 |
| C | -1.702421943532 | 2.215088665099 | 2.585549547633 |
| C | -0.407461445224 | 1.594062097028 | 2.030077251589 |
| C | -0.297337985335 | 1.764143651710 | 0.502879218681 |
| H | -2.387948312587 | 3.894182285261 | -0.337555044144 |
| H | -2.518704375402 | 1.587182020249 | 2.244958658115 |
| H | 0.458230465413 | 2.047870997431 | 2.521445920846 |
| H | -0.410101771626 | 0.534709425224 | 2.306576391918 |
| H | 0.481308094896 | 1.126101141431 | 0.075073075458 |
| C | -0.251165009125 | 3.282150121084 | 0.223868758337 |
| H | 0.111818530753 | 3.542532166158 | -0.768006907874 |
| H | 0.263600797903 | 3.890813144788 | 0.965054475573 |
| C | -1.629171317339 | 1.754940672335 | -1.707843517204 |
| H | -1.015637368116 | 2.559314608127 | -2.112625800514 |
| H | -1.218294557014 | 0.806856677639 | -2.068321809898 |
| H | -2.633324711765 | 1.864789297899 | -2.129851128747 |
| C | -2.731073644759 | 0.636674742704 | 0.184707261854 |
| H | -2.716699557933 | -0.138220690722 | -0.586398111905 |
| H | -2.535248567313 | 0.129072151956 | 1.128545029923 |
| H | -3.749657342605 | 1.035237739074 | 0.216269585774 |
| C | -3.664423281412 | 3.738292204053 | 2.083188611245 |
| H | -4.066853572405 | 4.605410201214 | 1.557844508074 |
| H | -4.228963692966 | 2.861782774576 | 1.756055748634 |

| | | | |
|---|---|---|---|
| H | -3.824196429119 | 3.876300290230 | 3.154661074487 |
| O | -1.521731015182 | 4.876947271390 | 2.150373065324 |
| O | -1.730505774600 | 2.087496846229 | 4.026712354551 |
| C | -1.698910854732 | 5.915981332369 | 1.131571507774 |
| H | -2.754808213752 | 6.007738483923 | 0.860893027399 |
| H | -1.149072127804 | 5.651413395938 | 0.225724250185 |
| C | -1.210561512868 | 7.354582313550 | 1.498104206282 |
| H | -1.779655535316 | 7.761010811581 | 2.344023307831 |
| H | -0.146169843315 | 7.341807060369 | 1.766927964215 |
| C | -2.484747389759 | 0.966882911706 | 4.574003900319 |
| H | -3.328272202119 | 0.727123602406 | 3.916418438377 |
| H | -2.891414536754 | 1.327715952584 | 5.520616613094 |
| C | -1.724710120350 | -0.375452855829 | 4.896587863425 |
| H | -1.376922226516 | -0.856510097080 | 3.971865701724 |
| H | -0.848656671345 | -0.156643350636 | 5.521721183941 |
| O | -1.432752474150 | 8.138659698858 | 0.299342902482 |
| C | -1.076669277427 | 9.556282897133 | 0.243338419529 |
| H | -1.637704390566 | 10.138023056538 | 0.984585880737 |
| H | -0.001713704693 | 9.703536437938 | 0.403041216728 |
| H | -1.348005462195 | 9.878534904285 | -0.760639010375 |
| O | -2.656773225167 | -1.234046841186 | 5.590778498047 |
| C | -2.239282421949 | -2.563386134467 | 6.042790086039 |
| H | -1.939511875255 | -3.193935682572 | 5.197051412343 |
| H | -1.411531656989 | -2.496281195640 | 6.758798844649 |
| H | -3.115518399558 | -2.988987740812 | 6.528919378599 |

Pin_TS1-F2000

($E_F$ = -929.40981328 a.u.; $G_F$ = -929.02270349 a.u.)

0 1

| | | | |
|---|---|---|---|
| C | -1.750396410461 | 1.970868170324 | -0.189423670530 |
| C | -1.573588127293 | 3.446250526174 | 0.334222170765 |
| C | -2.159457601259 | 3.960684745862 | 1.634115047511 |
| C | -1.734155938913 | 1.846733857914 | 2.875839137771 |
| C | -0.676312330064 | 1.150968830188 | 2.040716079537 |
| C | -0.449947906399 | 1.624937797919 | 0.606253107189 |
| H | -1.804983306033 | 4.167421444419 | -0.457734933057 |
| H | -2.765861306299 | 1.671678256239 | 2.598300746368 |
| H | 0.268186493433 | 1.227167866685 | 2.589551184267 |
| H | -0.934483694224 | 0.078129153844 | 2.035912832764 |
| H | 0.232794328574 | 0.932280052690 | 0.104133569223 |
| C | -0.065512460826 | 3.127313693993 | 0.553549637788 |
| H | 0.566162991046 | 3.384000115076 | -0.296699076837 |
| H | 0.364024868841 | 3.550411316454 | 1.461343136114 |
| C | -1.456886721810 | 1.975197361261 | -1.704277219257 |
| H | -0.564766256924 | 2.548244372231 | -1.962824532894 |
| H | -1.309573975133 | 0.951586402320 | -2.062156265061 |
| H | -2.299629971868 | 2.406858858197 | -2.253759696903 |
| C | -3.028229926059 | 1.149950341664 | -0.000740206903 |
| H | -2.908858096771 | 0.172700298371 | -0.479780823640 |
| H | -3.301681912416 | 0.964250907374 | 1.033894358317 |
| H | -3.873827763575 | 1.648457491248 | -0.484935457534 |
| C | -3.650228633275 | 3.990259450279 | 1.832882587461 |

| | | | |
|---|---|---|---|
| H | -4.102022088191 | 4.721897680117 | 1.146054888670 |
| H | -4.121009732865 | 3.027270582194 | 1.640719730269 |
| H | -3.884956655454 | 4.305482317873 | 2.851492434389 |
| O | -1.619727592077 | 5.258563683276 | 1.938933077899 |
| O | -1.536770408107 | 1.536407785648 | 4.230324358661 |
| C | -1.529744986399 | 6.201467426417 | 0.818654382830 |
| H | -2.422797738925 | 6.121491544977 | 0.191537401854 |
| H | -0.658140671507 | 5.962958186535 | 0.202940577817 |
| C | -1.411990206374 | 7.694967522124 | 1.221049901589 |
| H | -2.285362995251 | 8.004982536863 | 1.810211865183 |
| H | -0.508230187446 | 7.867732229481 | 1.820210901531 |
| C | -2.408167665989 | 0.551388691278 | 4.858629053561 |
| H | -3.282319239063 | 0.361160516791 | 4.227621990218 |
| H | -2.749640613607 | 0.978335464927 | 5.803896393971 |
| C | -1.698048004491 | -0.804004217819 | 5.169152437022 |
| H | -1.397979540262 | -1.294493042648 | 4.232258771357 |
| H | -0.791585218841 | -0.606953262898 | 5.757556142436 |
| O | -1.348989143196 | 8.408306926554 | -0.030429280060 |
| C | -1.245439049418 | 9.856006355024 | -0.030493307882 |
| H | -2.112887967546 | 10.318956538441 | 0.456584857145 |
| H | -0.328838706742 | 10.191548575522 | 0.470673822450 |
| H | -1.216266006065 | 10.147407659917 | -1.079495884230 |
| O | -2.621063239047 | -1.625486395446 | 5.902553077227 |
| C | -2.177499434422 | -2.935965673368 | 6.349397136525 |
| H | -1.908925587325 | -3.576310363610 | 5.499812908655 |
| H | -1.317949070717 | -2.854599116209 | 7.026374509004 |

| | | | |
|---|---|---|---|
| H | -3.023772286151 | -3.368359909706 | 6.881218483629 |

Pin_Int1-F2000

($E_F$ = -929.60961342 a.u.; $G_F$ = -929.22906870 a.u.)

0 1

| | | | |
|---|---|---|---|
| C | -2.367085839042 | 2.906132668206 | 1.175997683440 |
| C | -1.352143475307 | 4.135808735261 | 0.953241117040 |
| C | -1.763020784343 | 5.589279659332 | 1.048558676628 |
| C | -1.577900342604 | -0.363259395143 | 2.755648150953 |
| C | -1.288166454587 | 1.104080980979 | 3.020859393266 |
| C | -1.164891800459 | 2.082600516378 | 1.802742715706 |
| H | -0.895622136568 | 3.998749208808 | -0.031563866714 |
| H | -2.372227241452 | -0.664332754856 | 2.073181121706 |
| H | -2.027732647498 | 1.509382044373 | 3.730253413179 |
| H | -0.332102813199 | 1.144184522038 | 3.558546798747 |
| H | -0.659400202940 | 1.551294302988 | 0.989231505342 |
| C | -0.442139229888 | 3.445209226253 | 2.036431182442 |
| H | 0.637663657060 | 3.477511221504 | 1.872858396090 |
| H | -0.650196380037 | 3.830506609964 | 3.038391684045 |
| C | -2.963769144753 | 2.342112270653 | -0.108803748610 |
| H | -3.529016522610 | 1.424860374904 | 0.090206077874 |
| H | -3.645537918436 | 3.065700627712 | -0.568318832681 |
| H | -2.181808517054 | 2.106305495188 | -0.836321594934 |
| C | -3.476260548844 | 3.208401670007 | 2.182571778670 |
| H | -4.072919752018 | 2.310104614307 | 2.369258404581 |
| H | -3.090708982111 | 3.548885473895 | 3.145718379670 |

| | | | |
|---|---|---|---|
| H | -4.139793524545 | 3.985947990278 | 1.793853977235 |
| C | -1.961705689279 | 6.262254574809 | 2.371380458090 |
| H | -2.779101955768 | 6.990105800264 | 2.362147999642 |
| H | -2.195334077633 | 5.526453788450 | 3.138625294915 |
| H | -1.056638942726 | 6.799052771770 | 2.700282488838 |
| O | -1.423833637598 | 6.404917294900 | -0.048890880645 |
| O | -1.324324570139 | -1.235549380229 | 3.808093195035 |
| C | -1.877234059352 | 7.786029012090 | -0.042204527775 |
| H | -1.493213626150 | 8.310570413891 | 0.836695089210 |
| H | -2.971670899224 | 7.812091731310 | -0.006009951879 |
| C | -1.403199077434 | 8.577124436169 | -1.290553988966 |
| H | -0.307366379683 | 8.538350991521 | -1.359382674781 |
| H | -1.821892172628 | 8.135122864555 | -2.204706863896 |
| C | -1.677531729154 | -2.630960131634 | 3.617203888710 |
| H | -1.146020732527 | -3.027902939128 | 2.746294148118 |
| H | -2.754937702901 | -2.716433349612 | 3.435171615673 |
| C | -1.309278780757 | -3.479711546606 | 4.864527595674 |
| H | -0.229108633460 | -3.405598528992 | 5.051231340681 |
| H | -1.836293352247 | -3.085888792057 | 5.744626487874 |
| O | -1.863104457125 | 9.928240806102 | -1.112157863958 |
| C | -1.559743246274 | 10.914683693684 | -2.134284750568 |
| H | -0.476791949189 | 11.042133213459 | -2.256239511140 |
| H | -2.001683221928 | 10.637653556588 | -3.099564280547 |
| H | -2.000990446060 | 11.846529721370 | -1.783575453331 |
| O | -1.694309373364 | -4.837289124736 | 4.598390246724 |
| C | -1.441832081831 | -5.837854778184 | 5.622178062437 |

| | | | |
|---|---|---|---|
| H | -0.369910859283 | -5.921265488065 | 5.840712614791 |
| H | -1.982462592577 | -5.604746399164 | 6.548019177170 |
| H | -1.806562278156 | -6.778127487359 | 5.211405977803 |

Pin-F2000

($E_F$ = -929.44642381 a.u.; $G_F$ = -929.05440689 a.u.)

0 1

| | | | |
|---|---|---|---|
| C | -1.695780545593 | 1.703410210708 | -0.187094134289 |
| C | -1.824805938286 | 3.214608306429 | 0.288982775359 |
| C | -2.190436146717 | 3.520848250184 | 1.777398128610 |
| C | -1.699677364916 | 2.213323616367 | 2.561081178684 |
| C | -0.389249094086 | 1.612335826260 | 2.017016735377 |
| C | -0.282207518685 | 1.762384106943 | 0.486538440541 |
| H | -2.415676918844 | 3.842006378557 | -0.380922159023 |
| H | -2.498058289862 | 1.539932551154 | 2.264901701558 |
| H | 0.463031491140 | 2.099311944316 | 2.499829978795 |
| H | -0.361291808453 | 0.558352203723 | 2.311148684543 |
| H | 0.509953238867 | 1.134502273673 | 0.068721367794 |
| C | -0.266272449972 | 3.277377973065 | 0.185172093862 |
| H | 0.091679569599 | 3.529658618839 | -0.810566381632 |
| H | 0.236745836426 | 3.906662109054 | 0.916999464614 |
| C | -1.607274353062 | 1.694767119731 | -1.728095484005 |
| H | -1.009470384191 | 2.505589601620 | -2.143351035949 |
| H | -1.176040797919 | 0.750123005676 | -2.073572507905 |
| H | -2.612324019450 | 1.777554018551 | -2.154159636937 |
| C | -2.693131780203 | 0.583870333631 | 0.176960676940 |

| | | | |
|---|---|---|---|
| H | -2.651913549425 | -0.207523170732 | -0.576275367102 |
| H | -2.498858842174 | 0.102104961476 | 1.134225400410 |
| H | -3.720337529682 | 0.960644362974 | 0.186985066100 |
| C | -3.693689177005 | 3.664219761190 | 2.029426135043 |
| H | -4.108849006845 | 4.525004617647 | 1.503841335156 |
| H | -4.237087073339 | 2.778303968800 | 1.692503876481 |
| H | -3.865144766126 | 3.793721831129 | 3.100143887974 |
| O | -1.555472978406 | 4.811379547876 | 2.143344402419 |
| O | -1.706121365338 | 2.186097376553 | 4.000233322687 |
| C | -1.756642168541 | 5.871753857607 | 1.167251868182 |
| H | -2.822359092681 | 6.007216382267 | 0.959222301304 |
| H | -1.267993334187 | 5.619855121510 | 0.223178717553 |
| C | -1.196283130395 | 7.265387507871 | 1.558053655331 |
| H | -1.700294132803 | 7.661517315437 | 2.449413092711 |
| H | -0.119374392140 | 7.203798461400 | 1.765161685590 |
| C | -2.462664002764 | 1.119465041934 | 4.621001188830 |
| H | -3.390487955706 | 0.940563901557 | 4.064379572781 |
| H | -2.733091611328 | 1.494874214796 | 5.609525558855 |
| C | -1.752329929714 | -0.258092495519 | 4.837047276804 |
| H | -1.537871935104 | -0.743263069193 | 3.874126995009 |
| H | -0.799819769074 | -0.101283189606 | 5.361862371460 |
| O | -1.455772469757 | 8.092897253029 | 0.404555809659 |
| C | -1.035128900881 | 9.480914834561 | 0.398667311525 |
| H | -1.523109408050 | 10.051492873248 | 1.198988782113 |
| H | 0.053456951982 | 9.569882115393 | 0.505499900045 |
| H | -1.340528031370 | 9.875542204261 | -0.569521176612 |

| | | | |
|---|---|---|---|
| O | -2.648316670729 | -1.068587042960 | 5.617349551584 |
| C | -2.241192816623 | -2.415581006358 | 5.978460032077 |
| H | -2.073486656525 | -3.033747311481 | 5.087354744381 |
| H | -1.328638756191 | -2.405764298049 | 6.587538041536 |
| H | -3.065267342804 | -2.827024121447 | 6.559577394180 |

Pin_TS1-F1500

($E_F$ = -929.24611715 a.u.; $G_F$ = -928.85894930 a.u.)

0 1

| | | | |
|---|---|---|---|
| C | -1.742957882035 | 1.947630127753 | -0.207689270893 |
| C | -1.571469364046 | 3.432142017352 | 0.291367211361 |
| C | -2.189055441895 | 3.964044743809 | 1.564920130495 |
| C | -1.711889308955 | 1.803757112317 | 2.885638270433 |
| C | -0.667181336285 | 1.116971686686 | 2.030599686552 |
| C | -0.447500003129 | 1.609547427575 | 0.600349259538 |
| H | -1.779229925279 | 4.140447628785 | -0.520195949430 |
| H | -2.753275693736 | 1.635772156654 | 2.637839014254 |
| H | 0.282746172505 | 1.186394483771 | 2.570939399091 |
| H | -0.922125094883 | 0.043175774797 | 2.009832848482 |
| H | 0.241300805206 | 0.924677580512 | 0.095765368700 |
| C | -0.068512738239 | 3.113690896421 | 0.547611021821 |
| H | 0.582355324107 | 3.366119343140 | -0.289699282585 |
| H | 0.339111297424 | 3.543257862911 | 1.462462432424 |
| C | -1.439041833406 | 1.923587036399 | -1.719661607640 |
| H | -0.540697430263 | 2.486861750948 | -1.979280104469 |
| H | -1.295186704177 | 0.893242972148 | -2.059349307414 |

| | | | |
|---|---|---|---|
| H | -2.274325412759 | 2.351811291887 | -2.283067925369 |
| C | -3.020125634012 | 1.130076710505 | -0.006183065050 |
| H | -2.891449261412 | 0.136815835798 | -0.448673486305 |
| H | -3.303223637118 | 0.981292035008 | 1.031844364644 |
| H | -3.862296126853 | 1.607654363271 | -0.516702942944 |
| C | -3.678195837050 | 3.965925901243 | 1.762609264786 |
| H | -4.145597432062 | 4.684684455101 | 1.071247213529 |
| H | -4.131009639916 | 2.993312177293 | 1.578202648947 |
| H | -3.918947602819 | 4.284724025454 | 2.778923857725 |
| O | -1.659051964449 | 5.246114796178 | 1.895017563241 |
| O | -1.472106579383 | 1.564017557107 | 4.236768376350 |
| C | -1.534937997432 | 6.190464343908 | 0.791923865454 |
| H | -2.407536475000 | 6.119441532059 | 0.134859450466 |
| H | -0.643300049954 | 5.963315485046 | 0.200065309288 |
| C | -1.436609617204 | 7.662245522241 | 1.239030597296 |
| H | -2.330652494753 | 7.952124379806 | 1.807891390051 |
| H | -0.554506342637 | 7.820673672108 | 1.874187004462 |
| C | -2.347477463818 | 0.656656802541 | 4.950385596758 |
| H | -3.311511166295 | 0.570642833415 | 4.437989302088 |
| H | -2.516947574112 | 1.087311148232 | 5.939025433270 |
| C | -1.728757797629 | -0.754514003186 | 5.131123962649 |
| H | -1.602971364915 | -1.240478095578 | 4.152433857353 |
| H | -0.735841957659 | -0.653443232380 | 5.591141669165 |
| O | -1.335283719787 | 8.406390915846 | 0.015439400708 |
| C | -1.254757631271 | 9.842525829576 | 0.091620227793 |
| H | -2.146537972956 | 10.269902431318 | 0.568748026999 |

| | | | |
|---|---|---|---|
| H | -0.363770529076 | 10.166415644742 | 0.645413328949 |
| H | -1.189863015089 | 10.195186205786 | -0.937224775229 |
| O | -2.609223631572 | -1.512133736436 | 5.966247728327 |
| C | -2.192958962484 | -2.849412369275 | 6.314872813773 |
| H | -2.084143861175 | -3.479661113690 | 5.422468544368 |
| H | -1.242719950750 | -2.840272262277 | 6.864527354335 |
| H | -2.978089832703 | -3.254620131650 | 6.952217265592 |

Pin-F1500

($E_F$ = -929.30036002 a.u.; $G_F$ = -928.90823334 a.u.)

0 1

| | | | |
|---|---|---|---|
| C | -1.524270734859 | 1.993233611409 | -0.288044493284 |
| C | -1.708717441858 | 3.413086623107 | 0.385948880723 |
| C | -2.241224937794 | 3.454489295872 | 1.845407386923 |
| C | -1.754698451811 | 2.116153666859 | 2.561024385581 |
| C | -0.423582404557 | 1.559687087235 | 2.009900363697 |
| C | -0.188777838160 | 1.936803732734 | 0.534503199235 |
| H | -2.233410988221 | 4.158835106644 | -0.218634938367 |
| H | -2.534490172819 | 1.416322155481 | 2.270475035114 |
| H | 0.398060518217 | 1.950116658188 | 2.617327771118 |
| H | -0.430560050074 | 0.472681188549 | 2.143930263903 |
| H | 0.644221197992 | 1.374242939131 | 0.103298042335 |
| C | -0.154234036143 | 3.482636822927 | 0.464911470209 |
| H | 0.308555282140 | 3.875113258693 | -0.438372081250 |
| H | 0.256118639617 | 4.000503941104 | 1.329400522162 |
| C | -1.257992659907 | 2.181121596449 | -1.796312783346 |

| | | | |
|---|---|---|---|
| H | -0.589765573411 | 3.010910077517 | -2.025281892800 |
| H | -0.816185928303 | 1.272375089812 | -2.216740150051 |
| H | -2.201597573811 | 2.365231763201 | -2.319780669774 |
| C | -2.558992102013 | 0.862764952009 | -0.190261658675 |
| H | -2.254942061987 | 0.034902162096 | -0.838250160305 |
| H | -2.693684775031 | 0.445308217882 | 0.804384262496 |
| H | -3.535379392610 | 1.208104114473 | -0.544572384572 |
| C | -3.772228461309 | 3.476820093724 | 1.925175935228 |
| H | -4.201211001565 | 4.348105577708 | 1.429658476069 |
| H | -4.191807552541 | 2.592779399114 | 1.441151129923 |
| H | -4.081065569474 | 3.484880431319 | 2.973224158299 |
| O | -1.687510018418 | 4.637867845373 | 2.485781064788 |
| O | -1.747260653287 | 2.125664358309 | 3.994618450034 |
| C | -2.088220601645 | 5.944683515441 | 2.022707611001 |
| H | -2.190197655101 | 6.556433688763 | 2.922103407955 |
| H | -3.064470401387 | 5.923673344777 | 1.534551491974 |
| C | -1.122200139502 | 6.702986456086 | 1.069111764130 |
| H | -0.129876443896 | 6.803216018371 | 1.530484122364 |
| H | -1.004998913271 | 6.172605403218 | 0.115290339719 |
| C | -2.530511714303 | 1.117750149283 | 4.656212663797 |
| H | -3.453981765296 | 0.917808917455 | 4.099046964010 |
| H | -2.810017684394 | 1.542906119115 | 5.622336102113 |
| C | -1.820760939323 | -0.235554408163 | 4.932854282925 |
| H | -1.576612177871 | -0.747875678791 | 3.990267562457 |
| H | -0.882346547132 | -0.053432794484 | 5.475286584967 |
| O | -1.724532854532 | 7.988705262673 | 0.860220824960 |

| | | | |
|---|---|---|---|
| C | -1.047856267117 | 8.914462353917 | -0.011923162148 |
| H | -0.049283249802 | 9.167221627586 | 0.368300122372 |
| H | -0.950226273938 | 8.509894370797 | -1.028242003785 |
| H | -1.663928412339 | 9.812828009773 | -0.037150629400 |
| O | -2.728035601082 | -1.020849022716 | 5.715152597079 |
| C | -2.281437242787 | -2.319422182235 | 6.154089910294 |
| H | -2.056528194441 | -2.975179552149 | 5.302486432112 |
| H | -1.389800542903 | -2.241637483091 | 6.789886378360 |
| H | -3.102836986603 | -2.743288042772 | 6.730837085751 |

Pin_Int1-F1500

($E_F$ = -929.39872210 a.u.; $G_F$ = -929.01764381 a.u.)

0 1

| | | | |
|---|---|---|---|
| C | -2.335266995172 | 2.891635889888 | 1.188484430558 |
| C | -1.344710580325 | 4.134314175799 | 0.969841924818 |
| C | -1.781752818462 | 5.570873903057 | 1.081263570381 |
| C | -1.591318981222 | -0.312379827002 | 2.746154498394 |
| C | -1.225620248694 | 1.127205588082 | 3.019387355916 |
| C | -1.116231552109 | 2.093416035256 | 1.805385926631 |
| H | -0.893718900336 | 4.015750719574 | -0.019818452357 |
| H | -2.433665256729 | -0.570246148703 | 2.104795581116 |
| H | -1.915802923539 | 1.559313942126 | 3.762410288160 |
| H | -0.246249557901 | 1.118579708218 | 3.515151212377 |
| H | -0.608392139936 | 1.565290872631 | 0.991187752710 |
| C | -0.411543685730 | 3.461200697932 | 2.038393955502 |
| H | 0.665335295650 | 3.511678102471 | 1.861412862778 |

| | | | |
|---|---|---|---|
| H | -0.613964147140 | 3.839398586160 | 3.044241869261 |
| C | -2.925511116320 | 2.322722262979 | -0.097421260744 |
| H | -3.476910819978 | 1.396464777007 | 0.098461253835 |
| H | -3.618738620576 | 3.037705987856 | -0.553298472151 |
| H | -2.141241735365 | 2.101318873998 | -0.826990198731 |
| C | -3.447600922242 | 3.167197083472 | 2.199522738808 |
| H | -4.022262832832 | 2.254916787535 | 2.387519914586 |
| H | -3.066784298779 | 3.515747394450 | 3.161541285757 |
| H | -4.130342728518 | 3.929618919934 | 1.814211236682 |
| C | -1.979367254723 | 6.237787560855 | 2.407384378312 |
| H | -2.799523931110 | 6.962372318911 | 2.403069223286 |
| H | -2.207978081256 | 5.497972670974 | 3.172223961917 |
| H | -1.075019794776 | 6.776543802186 | 2.735390201032 |
| O | -1.443644258706 | 6.383335680569 | -0.010795339799 |
| O | -1.317171165474 | -1.199898878478 | 3.772327703677 |
| C | -1.913987880032 | 7.749736738781 | -0.009114704486 |
| H | -1.541583629240 | 8.287030902118 | 0.867547509184 |
| H | -3.009272188655 | 7.765542032627 | 0.018868967663 |
| C | -1.433408052389 | 8.516422106132 | -1.258089791027 |
| H | -0.336494505228 | 8.479848316955 | -1.316782808162 |
| H | -1.841694412615 | 8.059646774675 | -2.170164907956 |
| C | -1.732740727302 | -2.570064815904 | 3.583386900369 |
| H | -1.267413891502 | -2.978219504105 | 2.680005253830 |
| H | -2.821638515935 | -2.616521484669 | 3.464437760930 |
| C | -1.319351430684 | -3.424809758071 | 4.799764959844 |
| H | -0.227701889180 | -3.386618214973 | 4.921482299258 |

| | | | |
|---|---|---|---|
| H | -1.778338021482 | -3.015321369250 | 5.710769523221 |
| O | -1.898428444522 | 9.862116909210 | -1.101279724036 |
| C | -1.569484567394 | 10.807652720574 | -2.138660085586 |
| H | -0.482880347704 | 10.922666461516 | -2.245401655012 |
| H | -1.993738890670 | 10.505746771505 | -3.105155371070 |
| H | -2.006541534419 | 11.757057457416 | -1.831339195947 |
| O | -1.763834639732 | -4.761925950670 | 4.555696771670 |
| C | -1.470553686869 | -5.743448449932 | 5.571886502205 |
| H | -0.388549330545 | -5.849674328257 | 5.723836602197 |
| H | -1.942331176371 | -5.481686366948 | 6.527979397901 |
| H | -1.880964765278 | -6.685391061247 | 5.209998548626 |

Pin_TS1-F1000

($E_F$ = -929.08430440 a.u.; $G_F$ = -928.69748796 a.u.)

0 1

| | | | |
|---|---|---|---|
| C | -1.735529465071 | 1.918607208484 | -0.233489086273 |
| C | -1.578466726654 | 3.415253738792 | 0.234447735020 |
| C | -2.237533232030 | 3.968025154513 | 1.472097558833 |
| C | -1.665481971409 | 1.741588783994 | 2.895370099848 |
| C | -0.640803617851 | 1.073986491173 | 2.007451429196 |
| C | -0.440504579077 | 1.598171785557 | 0.583923758902 |
| H | -1.759860720039 | 4.104944885719 | -0.601200823907 |
| H | -2.718073946028 | 1.584934901556 | 2.687397143125 |
| H | 0.318886830762 | 1.135271967034 | 2.531374992014 |
| H | -0.888852168412 | -0.001159777540 | 1.961886823461 |
| H | 0.259019089131 | 0.930944671671 | 0.070426292506 |

| | | | |
|---|---|---|---|
| C | -0.080478467472 | 3.107392893863 | 0.535294350133 |
| H | 0.591809902276 | 3.362968038908 | -0.284463028112 |
| H | 0.296450947434 | 3.544692295787 | 1.459624054485 |
| C | -1.425381604694 | 1.862299860584 | -1.742469456089 |
| H | -0.524798706309 | 2.420599274965 | -2.006633631597 |
| H | -1.279403030131 | 0.825300079363 | -2.060518620038 |
| H | -2.257050019930 | 2.279748551057 | -2.319185810056 |
| C | -3.005693303465 | 1.097470132557 | -0.010072895044 |
| H | -2.862641379769 | 0.088197692117 | -0.410058377455 |
| H | -3.292964452820 | 0.990136513663 | 1.031991095126 |
| H | -3.849510212778 | 1.544289478623 | -0.545106540350 |
| C | -3.723040509786 | 3.931590456644 | 1.673216935205 |
| H | -4.215395097804 | 4.626819134391 | 0.973532887280 |
| H | -4.149863192120 | 2.944446575434 | 1.507298306255 |
| H | -3.969064745420 | 4.260534792677 | 2.685312328151 |
| O | -1.721296653480 | 5.234047490120 | 1.831909662965 |
| O | -1.367026752580 | 1.581629204103 | 4.237269091855 |
| C | -1.551207686069 | 6.183184610685 | 0.750839642708 |
| H | -2.399696097034 | 6.130113312580 | 0.060543020285 |
| H | -0.638687821980 | 5.963612128123 | 0.187635195950 |
| C | -1.466921096542 | 7.627446645305 | 1.255006223082 |
| H | -2.382198692807 | 7.896087487648 | 1.800883963555 |
| H | -0.610882811239 | 7.755950055734 | 1.932034645956 |
| C | -2.244452632984 | 0.770375702173 | 5.042669984152 |
| H | -3.268757504722 | 0.802805680320 | 4.655551016031 |
| H | -2.238286896607 | 1.196704678252 | 6.047087876019 |

| | | | |
|---|---|---|---|
| C | -1.756902258130 | -0.690088937472 | 5.117456168119 |
| H | -1.796791308736 | -1.153031818567 | 4.119828037229 |
| H | -0.710241879314 | -0.699950129590 | 5.453614701147 |
| O | -1.316559764182 | 8.418239144385 | 0.073542407472 |
| C | -1.255771632672 | 9.836917955117 | 0.248394245542 |
| H | -2.173351964761 | 10.225313029722 | 0.711242044172 |
| H | -0.396231244458 | 10.130815783152 | 0.866245732817 |
| H | -1.145755267852 | 10.266174731536 | -0.747399699697 |
| O | -2.598148964909 | -1.384066992748 | 6.034068140975 |
| C | -2.238844797914 | -2.748017156962 | 6.292254261700 |
| H | -2.280179011459 | -3.353955384765 | 5.376952756528 |
| H | -1.230267035710 | -2.821128968581 | 6.720834817214 |
| H | -2.964653537579 | -3.129722278890 | 7.009910887821 |

Pin_Int1-F1000

($E_F$ = -929.19018418 a.u.; $G_F$ = -928.80869475 a.u.)

0 1

| | | | |
|---|---|---|---|
| C | -2.289602026866 | 2.876583714511 | 1.211289482776 |
| C | -1.331235319348 | 4.137157514295 | 0.995739486787 |
| C | -1.801900043663 | 5.554294847553 | 1.123708105356 |
| C | -1.591520958183 | -0.253053840713 | 2.738149907241 |
| C | -1.141746917301 | 1.151973940506 | 3.021850034278 |
| C | -1.048376612102 | 2.110356707112 | 1.814483558691 |
| H | -0.887670720840 | 4.039606634153 | 0.000587759743 |
| H | -2.476911494129 | -0.456630642385 | 2.136519081124 |
| H | -1.776822438619 | 1.607938859374 | 3.799360331056 |

| | | | |
|---|---|---|---|
| H | -0.142594700856 | 1.087895836163 | 3.471990949002 |
| H | -0.537203171459 | 1.589315646425 | 0.997781042691 |
| C | -0.368946392610 | 3.486893573692 | 2.047181341572 |
| H | 0.703576117230 | 3.562267146644 | 1.854179421452 |
| H | -0.565389939383 | 3.856293840976 | 3.057487745741 |
| C | -2.873569267534 | 2.300201224869 | -0.074416769062 |
| H | -3.403093406183 | 1.360833775458 | 0.119191667623 |
| H | -3.584973636678 | 3.001524704368 | -0.523498274315 |
| H | -2.088211108744 | 2.099827729649 | -0.808889720498 |
| C | -3.403130467720 | 3.118847250071 | 2.229973130509 |
| H | -3.951130123765 | 2.190642938995 | 2.419468458956 |
| H | -3.026407198566 | 3.475788508963 | 3.190408668966 |
| H | -4.108982507068 | 3.863030801797 | 1.850786939957 |
| C | -1.999489348619 | 6.215310170844 | 2.452715366774 |
| H | -2.824289688667 | 6.934438662519 | 2.452797929664 |
| H | -2.220794805277 | 5.471483084362 | 3.215800077719 |
| H | -1.097187790152 | 6.758789308483 | 2.778897248329 |
| O | -1.469796534716 | 6.364707645354 | 0.036077415831 |
| O | -1.309531157143 | -1.163243485676 | 3.733847652746 |
| C | -1.960564660068 | 7.715580390833 | 0.029509878654 |
| H | -1.599204306680 | 8.269328413436 | 0.901060405822 |
| H | -3.056479393522 | 7.718385676188 | 0.051123522315 |
| C | -1.475359524167 | 8.451129490418 | -1.225269378854 |
| H | -0.377439514026 | 8.417453774477 | -1.273089482166 |
| H | -1.872118287873 | 7.970756302728 | -2.130720233566 |
| C | -1.796014479659 | -2.502213380340 | 3.544037808128 |

| | | | |
|---|---|---|---|
| H | -1.400536174780 | -2.917380345677 | 2.610738799274 |
| H | -2.891241983717 | -2.501623652990 | 3.487774156816 |
| C | -1.345333283566 | -3.371823503561 | 4.724515645657 |
| H | -0.247356369899 | -3.374536281455 | 4.781208949385 |
| H | -1.732617754766 | -2.949615917002 | 5.663048502716 |
| O | -1.946066670213 | 9.792433636797 | -1.103972011201 |
| C | -1.585834641932 | 10.686390753139 | -2.163118297043 |
| H | -0.495473494967 | 10.788276311084 | -2.248990927878 |
| H | -1.987262158709 | 10.348882769027 | -3.128279970668 |
| H | -2.019228750538 | 11.654206187795 | -1.911946632848 |
| O | -1.852252025717 | -4.684523468210 | 4.503345590744 |
| C | -1.520806673717 | -5.652123309842 | 5.507591793332 |
| H | -0.433854508769 | -5.785194022788 | 5.592442494412 |
| H | -1.920742079936 | -5.364044366910 | 6.489198184761 |
| H | -1.975656002542 | -6.591605794330 | 5.194705615556 |

Pin-F1000

($E_F$ = -929.15396719 a.u.; $G_F$ = -928.76146386 a.u.)

0 1

| | | | |
|---|---|---|---|
| C | -1.498087097637 | 1.981447376163 | -0.313784991221 |
| C | -1.702164062315 | 3.395772230347 | 0.362283003919 |
| C | -2.262540745458 | 3.406025408121 | 1.809703658353 |
| C | -1.751742145570 | 2.098568062577 | 2.547143319263 |
| C | -0.419878031400 | 1.540901930664 | 1.993059425535 |
| C | -0.173062137564 | 1.931388809515 | 0.524734710377 |
| H | -2.224454637318 | 4.141627867785 | -0.244265194756 |

| | | | |
|---|---|---|---|
| H | -2.525370453716 | 1.367208110657 | 2.319910171117 |
| H | 0.397742387573 | 1.926669445408 | 2.608567240274 |
| H | -0.426233079683 | 0.452902013166 | 2.117307810736 |
| H | 0.669646641064 | 1.379470812532 | 0.098724289747 |
| C | -0.150807714623 | 3.478295602108 | 0.467438242879 |
| H | 0.323078104226 | 3.882185282523 | -0.425138447648 |
| H | 0.241237430078 | 3.992275085594 | 1.343010856181 |
| C | -1.211659352060 | 2.168084010405 | -1.817713921647 |
| H | -0.524086460829 | 2.985337770267 | -2.034809500328 |
| H | -0.781502510139 | 1.252499084296 | -2.235480495662 |
| H | -2.144603476127 | 2.371938604435 | -2.352774940037 |
| C | -2.526039585370 | 0.846568155391 | -0.222054909321 |
| H | -2.171046872057 | -0.012511840628 | -0.800063717956 |
| H | -2.725747319338 | 0.487412057666 | 0.784105557532 |
| H | -3.479238560247 | 1.159805260366 | -0.659640599679 |
| C | -3.795521281383 | 3.394090588153 | 1.852315857224 |
| H | -4.230139178973 | 4.258139258893 | 1.348503372069 |
| H | -4.184143959918 | 2.503085454369 | 1.355928928780 |
| H | -4.130654571824 | 3.393618732360 | 2.892273287162 |
| O | -1.749574183001 | 4.575248912816 | 2.495327942032 |
| O | -1.699843300113 | 2.206394956186 | 3.971231059670 |
| C | -2.153468646012 | 5.878598571654 | 2.050805581082 |
| H | -2.299024990017 | 6.474578517135 | 2.955241968523 |
| H | -3.108107830227 | 5.857786280396 | 1.520710682239 |
| C | -1.148949945994 | 6.639913347667 | 1.157466781081 |
| H | -0.177640632405 | 6.729219026730 | 1.664407882389 |

| | | | |
|---|---|---|---|
| H | -0.989720718720 | 6.120677602453 | 0.203656733132 |
| C | -2.493590443208 | 1.273498664680 | 4.708955475098 |
| H | -3.479433927872 | 1.142432252569 | 4.244951770690 |
| H | -2.645347037210 | 1.719830062420 | 5.693678044023 |
| C | -1.851642640826 | -0.115414037270 | 4.908424374984 |
| H | -1.716740416169 | -0.623904625015 | 3.941471514594 |
| H | -0.861110901937 | 0.000873878492 | 5.371364275559 |
| O | -1.733306653743 | 7.925664499040 | 0.936742999309 |
| C | -1.003379207159 | 8.816577684387 | 0.087315012329 |
| H | -0.011570438430 | 9.045369160108 | 0.500763603124 |
| H | -0.877416773850 | 8.398299338287 | -0.920990489431 |
| H | -1.587088239549 | 9.734964024300 | 0.025061813228 |
| O | -2.732315072460 | -0.857883640950 | 5.749805446992 |
| C | -2.298902648925 | -2.173926156002 | 6.112204746230 |
| H | -2.174339002911 | -2.814221461200 | 5.228083254858 |
| H | -1.350127215977 | -2.145675944547 | 6.665003591199 |
| H | -3.076176510464 | -2.591645093746 | 6.751861642760 |

Pin_TS1-F500

($E_F$ = -928.92458323 a.u.; $G_F$ = -928.53922463 a.u.)

0 1

| | | | |
|---|---|---|---|
| C | -1.730214795313 | 1.888279548111 | -0.275237432370 |
| C | -1.616133043485 | 3.403291690159 | 0.149861745674 |
| C | -2.343409103103 | 3.980517481200 | 1.328505662201 |
| C | -1.533592654005 | 1.632202706573 | 2.904671392095 |
| C | -0.554942158525 | 1.017842828051 | 1.940379776930 |

| | | | |
|---|---|---|---|
| C | -0.418274864673 | 1.613930023310 | 0.534953931234 |
| H | -1.767568377084 | 4.060747194828 | -0.719709751605 |
| H | -2.601733547411 | 1.513615050069 | 2.759026261412 |
| H | 0.428494214437 | 1.065084814959 | 2.420247399154 |
| H | -0.788518356167 | -0.057789472973 | 1.843970593587 |
| H | 0.302960040673 | 0.997719658782 | -0.011377546451 |
| C | -0.120646916334 | 3.137232222304 | 0.509264583272 |
| H | 0.575100406326 | 3.425375483619 | -0.280351363965 |
| H | 0.201631482557 | 3.579203536946 | 1.452025946954 |
| C | -1.432336088264 | 1.796149503272 | -1.783508677200 |
| H | -0.543656437652 | 2.366237523172 | -2.064972879140 |
| H | -1.266609968169 | 0.754387331646 | -2.075549047195 |
| H | -2.275224996090 | 2.182722382620 | -2.365491886396 |
| C | -2.970603765770 | 1.039239567377 | -0.002125877770 |
| H | -2.793740353223 | 0.014866018912 | -0.346562107599 |
| H | -3.245604297353 | 0.982248688943 | 1.047560448122 |
| H | -3.832806468620 | 1.426238610140 | -0.554074202619 |
| C | -3.819874252081 | 3.870129424388 | 1.541534957590 |
| H | -4.362475952410 | 4.507081463105 | 0.821890718131 |
| H | -4.191736794035 | 2.854719029663 | 1.423083287045 |
| H | -4.075392868641 | 4.226513114793 | 2.542383231578 |
| O | -1.862704242785 | 5.233753715787 | 1.728139655992 |
| O | -1.137665840150 | 1.533977268939 | 4.219102345018 |
| C | -1.586665565814 | 6.187083973471 | 0.684128371920 |
| H | -2.379649752585 | 6.170936328344 | -0.071592406010 |
| H | -0.636179758420 | 5.961895356509 | 0.189134993698 |

| | | | |
|---|---|---|---|
| C | -1.521110005021 | 7.592811171648 | 1.267771011472 |
| H | -2.474766683887 | 7.843125218824 | 1.754241742157 |
| H | -0.723920110014 | 7.661623932819 | 2.021768645283 |
| C | -2.026328920556 | 0.880834404396 | 5.135887919536 |
| H | -3.072746158556 | 1.085394401846 | 4.883177500830 |
| H | -1.820009983130 | 1.290778660985 | 6.125481621615 |
| C | -1.765692776189 | -0.628705665071 | 5.142228010715 |
| H | -1.983969377715 | -1.050234940834 | 4.148511922430 |
| H | -0.701534474621 | -0.805457786955 | 5.356342002731 |
| O | -1.263444608095 | 8.452461225456 | 0.163123286925 |
| C | -1.228179511695 | 9.840096138153 | 0.474096671042 |
| H | -2.187402799261 | 10.185933820676 | 0.884442169525 |
| H | -0.433392872092 | 10.072460208986 | 1.196770950273 |
| H | -1.027559426062 | 10.368221070575 | -0.458327123737 |
| O | -2.592693540759 | -1.213874117274 | 6.135654830385 |
| C | -2.380217738537 | -2.611411417757 | 6.319700437347 |
| H | -2.593740581710 | -3.175268873671 | 5.400874369392 |
| H | -1.347388914430 | -2.822051168657 | 6.629994423776 |
| H | -3.063464136685 | -2.935656798194 | 7.104769829230 |

Pin_Int1-F500

($E_F$ = -928.98417295 a.u.; $G_F$ = -928.60279607 a.u.)

0 1

| | | | |
|---|---|---|---|
| C | -2.207957428035 | 2.865167530733 | 1.256344960958 |
| C | -1.292139895840 | 4.149692515934 | 1.045694825849 |
| C | -1.807806891123 | 5.542621936042 | 1.190636939874 |

| | | | |
|---|---|---|---|
| C | -1.561461484517 | -0.179038957872 | 2.732086247170 |
| C | -1.013318460218 | 1.178890342883 | 3.034216824273 |
| C | -0.938208510949 | 2.139029990986 | 1.840015745874 |
| H | -0.857187751710 | 4.077260899206 | 0.044744993163 |
| H | -2.482431714003 | -0.309603217012 | 2.164723273410 |
| H | -1.584337257924 | 1.652686861886 | 3.850166433800 |
| H | 0.001118655404 | 1.047039556844 | 3.432210198850 |
| H | -0.421495770631 | 1.633284398821 | 1.017218313043 |
| C | -0.295095722147 | 3.528612269583 | 2.077275109861 |
| H | 0.771062493679 | 3.638890601077 | 1.867454793936 |
| H | -0.487354263665 | 3.884527526855 | 3.093176689051 |
| C | -2.786214638577 | 2.282550350153 | -0.029287387192 |
| H | -3.289261026565 | 1.328024814102 | 0.159908228407 |
| H | -3.519253175483 | 2.968302968352 | -0.467559789333 |
| H | -2.001339279185 | 2.108801016402 | -0.771038865386 |
| C | -3.319415235016 | 3.064758626156 | 2.287200735968 |
| H | -3.833556939750 | 2.117847725436 | 2.478663020952 |
| H | -2.945904017058 | 3.431286720741 | 3.245217960074 |
| H | -4.053903771370 | 3.785782493359 | 1.917873879588 |
| C | -2.000924664756 | 6.201865475736 | 2.520770265341 |
| H | -2.835470703755 | 6.909526095380 | 2.527256164967 |
| H | -2.204395369782 | 5.455815741480 | 3.286807490588 |
| H | -1.102675233799 | 6.758277408249 | 2.836601176710 |
| O | -1.500106463219 | 6.348870707796 | 0.100523438227 |
| O | -1.295599770894 | -1.123453239007 | 3.691501930495 |
| C | -2.022845896028 | 7.679839111946 | 0.078296977989 |

| | | | |
|---|---|---|---|
| H | -1.667043853404 | 8.262013257674 | 0.934053595236 |
| H | -3.118772927582 | 7.660706012402 | 0.106125711369 |
| C | -1.550100136497 | 8.369397281401 | -1.197103537260 |
| H | -0.451658748503 | 8.341291142232 | -1.244604454583 |
| H | -1.942207884517 | 7.848872711008 | -2.082623641510 |
| C | -1.867842415088 | -2.419382289279 | 3.489197588014 |
| H | -1.538161788972 | -2.835500526137 | 2.530833389812 |
| H | -2.962978603369 | -2.356737405853 | 3.483665638067 |
| C | -1.407363520029 | -3.319676519417 | 4.631254164109 |
| H | -0.309023434678 | -3.375604756512 | 4.635069376195 |
| H | -1.727101398079 | -2.890067705520 | 5.592004812676 |
| O | -2.028517028614 | 9.705879077042 | -1.130706786456 |
| C | -1.647313701857 | 10.528083923604 | -2.228561226359 |
| H | -0.554523381244 | 10.616954223292 | -2.304283027129 |
| H | -2.034772237797 | 10.136441289166 | -3.179469532382 |
| H | -2.074499653830 | 11.514673819817 | -2.048317483706 |
| O | -1.986111320182 | -4.598623918316 | 4.422691152809 |
| C | -1.634008705164 | -5.565069945868 | 5.408708858974 |
| H | -0.548987527688 | -5.737239491110 | 5.433144227202 |
| H | -1.963986335720 | -5.256077156622 | 6.410343052914 |
| H | -2.137393350538 | -6.492848639864 | 5.136724882270 |

Pin-F500

($E_F$ = -929.01075085 a.u.; $G_F$ = -928.61714900 a.u.)

0 1

| | | | |
|---|---|---|---|
| C | -1.472453332155 | 1.964037340577 | -0.345173516328 |

| | | | |
|---|---|---|---|
| C | -1.704681726894 | 3.372815110432 | 0.331512897747 |
| C | -2.295632854975 | 3.353295662890 | 1.764009558986 |
| C | -1.750427538018 | 2.080757893113 | 2.526802986767 |
| C | -0.417751615417 | 1.522646604812 | 1.968662918367 |
| C | -0.158208014304 | 1.928546252062 | 0.508577053548 |
| H | -2.225155510819 | 4.115905853262 | -0.280070848139 |
| H | -2.514992930332 | 1.321887172013 | 2.362405460067 |
| H | 0.396405067156 | 1.900845844171 | 2.593102700365 |
| H | -0.425640299408 | 0.433424633324 | 2.081073997987 |
| H | 0.697533223669 | 1.391925978529 | 0.089046130310 |
| C | -0.157335950931 | 3.475736453057 | 0.465009423441 |
| H | 0.325973885011 | 3.894729318994 | -0.415713070833 |
| H | 0.212770701176 | 3.986284776086 | 1.352468770630 |
| C | -1.171351103701 | 2.152148922070 | -1.844909876814 |
| H | -0.478881558762 | 2.968240923381 | -2.051736207537 |
| H | -0.738699886479 | 1.236560305868 | -2.260194728565 |
| H | -2.097872967244 | 2.359869949215 | -2.389573740298 |
| C | -2.483413874521 | 0.815483536191 | -0.253697790838 |
| H | -2.091826741805 | -0.056165563146 | -0.787815047617 |
| H | -2.713480078545 | 0.489889681619 | 0.757617339775 |
| H | -3.425360237574 | 1.095505627427 | -0.735781941472 |
| C | -3.828731495438 | 3.294096575597 | 1.769976366864 |
| H | -4.274946977882 | 4.146574834900 | 1.256058791690 |
| H | -4.181964272827 | 2.392710979031 | 1.266804371239 |
| H | -4.188211241373 | 3.285953593642 | 2.801654293729 |
| O | -1.840331003427 | 4.520211559887 | 2.482819193089 |

| | | | |
|---|---|---|---|
| O | -1.654023287057 | 2.280198209087 | 3.935665925040 |
| C | -2.254210897647 | 5.810815889803 | 2.031906007404 |
| H | -2.515939097863 | 6.387705876646 | 2.923212654847 |
| H | -3.144102375665 | 5.769690641010 | 1.399470307181 |
| C | -1.174353917158 | 6.590235886297 | 1.269762191142 |
| H | -0.262645140861 | 6.671705637535 | 1.879094850551 |
| H | -0.911710838855 | 6.086315812238 | 0.330542421900 |
| C | -2.458901059024 | 1.427294168182 | 4.742477990480 |
| H | -3.477341276680 | 1.341368650206 | 4.342149703064 |
| H | -2.524335838321 | 1.904906502839 | 5.721943170164 |
| C | -1.872192768385 | 0.021060376199 | 4.919553797947 |
| H | -1.795697223742 | -0.491087567487 | 3.947564838633 |
| H | -0.858815401259 | 0.095815612533 | 5.340705815025 |
| O | -1.733337679376 | 7.873556766737 | 1.007880166648 |
| C | -0.903867157536 | 8.738936110135 | 0.241724547606 |
| H | 0.046713779018 | 8.945217296444 | 0.753535116882 |
| H | -0.683313412509 | 8.314317381090 | -0.748090000723 |
| H | -1.450469834424 | 9.673817715844 | 0.114961363646 |
| O | -2.742576248586 | -0.686594941937 | 5.791721546066 |
| C | -2.324404495568 | -2.012511034960 | 6.098598897938 |
| H | -2.260392856194 | -2.634497388479 | 5.194479169996 |
| H | -1.346312465588 | -2.020114360982 | 6.599580211528 |
| H | -3.072916631207 | -2.434468634175 | 6.769609220591 |

Pin_TS1-F200

($E_F$ = -928.83008680 a.u.; $G_F$ = -928.44725113 a.u.)

0 1

| | | | |
|---|---|---|---|
| C | -1.727006764590 | 1.836510251124 | -0.323768191807 |
| C | -1.695877003783 | 3.378267505959 | 0.024133233290 |
| C | -2.544202522865 | 3.974493572929 | 1.102235138193 |
| C | -1.243349158009 | 1.512489699261 | 2.898660577490 |
| C | -0.359166658743 | 0.978869951249 | 1.815511260741 |
| C | -0.368447013450 | 1.669142684856 | 0.444938521307 |
| H | -1.817543247417 | 3.978964737890 | -0.892898753221 |
| H | -2.324709692162 | 1.501880796165 | 2.831336157432 |
| H | 0.665783618198 | 1.022830763319 | 2.200805875511 |
| H | -0.571176468075 | -0.095202164655 | 1.663439219572 |
| H | 0.379933696350 | 1.155170342093 | -0.166460755496 |
| C | -0.207603469637 | 3.211962059023 | 0.463338125431 |
| H | 0.509221246993 | 3.584287380034 | -0.270801915906 |
| H | 0.015820633838 | 3.647748777277 | 1.437272807094 |
| C | -1.488604125388 | 1.667294708544 | -1.832690268082 |
| H | -0.634140602217 | 2.257665005025 | -2.175397773197 |
| H | -1.288878585355 | 0.618222383931 | -2.073772635449 |
| H | -2.367484853872 | 1.987709292313 | -2.401578794820 |
| C | -2.896787124001 | 0.941916026175 | 0.078974932019 |
| H | -2.630958603579 | -0.105647134034 | -0.097615494977 |
| H | -3.175007215711 | 1.037472196110 | 1.126149235791 |
| H | -3.782497956140 | 1.160764016915 | -0.525282549424 |
| C | -4.033754491261 | 3.865942259385 | 1.162328161608 |
| H | -4.501400805303 | 4.555329963033 | 0.437620706760 |
| H | -4.396820693244 | 2.864541608616 | 0.940711027417 |

| | | | |
|---|---|---|---|
| H | -4.389212707026 | 4.160151566814 | 2.153807074154 |
| O | -2.088654743194 | 5.188736669090 | 1.608702769277 |
| O | -0.729022617276 | 1.374149841523 | 4.163179522022 |
| C | -1.692748907651 | 6.181084811109 | 0.647877966445 |
| H | -2.427645219002 | 6.248403168238 | -0.162518846906 |
| H | -0.718107664168 | 5.940938432285 | 0.210020716824 |
| C | -1.613706871202 | 7.524130494056 | 1.352287908004 |
| H | -2.593658002195 | 7.777934375133 | 1.782003543861 |
| H | -0.884026304215 | 7.478774807255 | 2.173957742831 |
| C | -1.628687932266 | 0.947033362652 | 5.189297625118 |
| H | -2.622877588977 | 1.386953949112 | 5.052079692625 |
| H | -1.220037042136 | 1.299063055096 | 6.137261050041 |
| C | -1.714566863730 | -0.576915239812 | 5.195037766975 |
| H | -2.117863863478 | -0.930042832359 | 4.232898254083 |
| H | -0.702036512862 | -0.991927246769 | 5.307503896758 |
| O | -1.223287257918 | 8.468912197780 | 0.367839105525 |
| C | -1.157480775631 | 9.802459662088 | 0.846483633350 |
| H | -2.134165544742 | 10.147614603365 | 1.214912884990 |
| H | -0.422193262060 | 9.905299395026 | 1.657293443765 |
| H | -0.851819342756 | 10.429412196203 | 0.008297028033 |
| O | -2.549687960096 | -0.965733058498 | 6.270130932407 |
| C | -2.632465251891 | -2.374463216645 | 6.434312079993 |
| H | -3.050800993280 | -2.860241708336 | 5.541369443169 |
| H | -1.646291916270 | -2.812908500924 | 6.642114849809 |
| H | -3.291634689727 | -2.560935913051 | 7.282484413793 |

Pin-F200

($E_F$ = -928.92669706 a.u.; $G_F$ = -928.53345353 a.u.)

0 1

| | | | |
|---|---|---|---|
| C | -1.675512517115 | 1.544645402611 | -0.498636647667 |
| C | -1.788226582595 | 3.047816829452 | -0.028927453079 |
| C | -2.250000080362 | 3.269562666438 | 1.433969889931 |
| C | -1.707853850751 | 2.096119314045 | 2.327494905998 |
| C | -0.451372337686 | 1.388270179828 | 1.759883549810 |
| C | -0.294716288315 | 1.563981604160 | 0.241470051896 |
| H | -2.329741183649 | 3.712196629117 | -0.707002598146 |
| H | -2.519032943640 | 1.368410241204 | 2.347936401292 |
| H | 0.425545998516 | 1.806356955282 | 2.261461431786 |
| H | -0.496769453607 | 0.328134121829 | 2.029276461834 |
| H | 0.498340517248 | 0.924911879034 | -0.157208178541 |
| C | -0.231313934679 | 3.086090218593 | -0.037580648836 |
| H | 0.189352375994 | 3.342727465483 | -1.007770407419 |
| H | 0.237873807653 | 3.698921831446 | 0.731158784407 |
| C | -1.500099424102 | 1.493104487959 | -2.029000312205 |
| H | -0.793789738644 | 2.229593411976 | -2.412185376568 |
| H | -1.145739137010 | 0.503399974105 | -2.333727506937 |
| H | -2.460932588626 | 1.669984280934 | -2.522662908505 |
| C | -2.724215595882 | 0.480830118301 | -0.154089218061 |
| H | -2.417796177139 | -0.481590721668 | -0.576296843934 |
| H | -2.884532832397 | 0.323879194171 | 0.909480430744 |
| H | -3.689717015903 | 0.738539661739 | -0.600733598280 |
| C | -3.775878263680 | 3.339288438561 | 1.562572444668 |

| | | | |
|---|---|---|---|
| H | -4.184674307728 | 4.176804343551 | 0.994124116282 |
| H | -4.242403662460 | 2.426660531642 | 1.186569031481 |
| H | -4.047556368604 | 3.469383201318 | 2.612718143357 |
| O | -1.678669074884 | 4.490782709500 | 1.952319173190 |
| O | -1.478304763594 | 2.513281854705 | 3.671983244010 |
| C | -1.830943067680 | 5.653761694394 | 1.150674245558 |
| H | -2.877769890723 | 5.836369118434 | 0.881726410557 |
| H | -1.254183609336 | 5.585582533206 | 0.220688294767 |
| C | -1.315930169834 | 6.846310789185 | 1.940346858332 |
| H | -1.905646865482 | 6.979764644951 | 2.858724675738 |
| H | -0.268821092620 | 6.677737130617 | 2.231492956392 |
| C | -2.218219920995 | 1.811212120263 | 4.661156600458 |
| H | -3.283361813448 | 1.752740968276 | 4.400265426817 |
| H | -2.129916314638 | 2.392073383094 | 5.581196038621 |
| C | -1.688374897040 | 0.397662505458 | 4.907167815705 |
| H | -1.746866508515 | -0.199924107159 | 3.984525385776 |
| H | -0.631149977478 | 0.445135380051 | 5.208034025628 |
| O | -1.436180436384 | 7.971501441383 | 1.082137773907 |
| C | -0.964696645460 | 9.181337359761 | 1.650155464030 |
| H | -1.523532654898 | 9.446943367753 | 2.559098628400 |
| H | 0.103455223775 | 9.119741333428 | 1.903655233164 |
| H | -1.107993512600 | 9.964047997145 | 0.904401529688 |
| O | -2.487675934075 | -0.184129946349 | 5.923374579644 |
| C | -2.130303596277 | -1.524230468328 | 6.225769477189 |
| H | -2.239833827332 | -2.178046619694 | 5.348540369917 |
| H | -1.094182446452 | -1.593973873607 | 6.586139879606 |

| | | | |
|---|---|---|---|
| H | -2.803987470786 | -1.867673592548 | 7.011391795500 |

Pin_Int1-F200

($E_F$ = -928.86202465 a.u.; $G_F$ = -928.48106209 a.u.)

0 1

| | | | |
|---|---|---|---|
| C | -2.095585786346 | 2.881130513083 | 1.322360611852 |
| C | -1.199686530751 | 4.177133359919 | 1.125436426942 |
| C | -1.743821793127 | 5.554082107162 | 1.279170743716 |
| C | -1.498655515408 | -0.121668395684 | 2.733492035935 |
| C | -0.889523392417 | 1.197385192893 | 3.070065889826 |
| C | -0.813615653749 | 2.172499658560 | 1.895219369839 |
| H | -0.763746774942 | 4.120942084893 | 0.123840688782 |
| H | -2.423219700018 | -0.193745401378 | 2.162095734049 |
| H | -1.428683565929 | 1.670317052566 | 3.908106049172 |
| H | 0.126385503806 | 1.016997209624 | 3.444309125416 |
| H | -0.290152815179 | 1.683699753284 | 1.066448404538 |
| C | -0.192091322478 | 3.567101121446 | 2.149668524334 |
| H | 0.871573524191 | 3.697997879240 | 1.939522743315 |
| H | -0.389173873307 | 3.908439938051 | 3.169541479532 |
| C | -2.670936320572 | 2.305381208553 | 0.032461624179 |
| H | -3.169255115567 | 1.346663283499 | 0.212141781204 |
| H | -3.407657031225 | 2.991278665964 | -0.399457306648 |
| H | -1.885295935219 | 2.142725723032 | -0.711019751482 |
| C | -3.205912031412 | 3.061359241500 | 2.358551742543 |
| H | -3.707066570043 | 2.107818885659 | 2.550577943819 |
| H | -2.833665030446 | 3.432393865701 | 3.315431494359 |

| | | | |
|---|---|---|---|
| H | -3.951159997244 | 3.773319479677 | 1.993235149563 |
| C | -1.912564548006 | 6.227914409787 | 2.604438397588 |
| H | -2.759355416396 | 6.920747992151 | 2.623095170720 |
| H | -2.082336589769 | 5.488790398018 | 3.385696572180 |
| H | -1.016534622359 | 6.804987615525 | 2.888344328993 |
| O | -1.491771905132 | 6.344031198366 | 0.168101511458 |
| O | -1.278932234651 | -1.098731151074 | 3.666498136988 |
| C | -2.059284464200 | 7.651791884946 | 0.124242640750 |
| H | -1.687095277967 | 8.278496237597 | 0.941168542224 |
| H | -3.152214359302 | 7.600958396253 | 0.197328368469 |
| C | -1.652788294214 | 8.283561913803 | -1.197584581624 |
| H | -0.556237961319 | 8.274993646396 | -1.284322980812 |
| H | -2.063267791726 | 7.705255345297 | -2.038077536739 |
| C | -1.918052248635 | -2.353243115096 | 3.429013548292 |
| H | -1.610909356093 | -2.761821438977 | 2.459835806766 |
| H | -3.008643283655 | -2.234247890416 | 3.427691648092 |
| C | -1.499609978926 | -3.294219629876 | 4.547617580370 |
| H | -0.405129778163 | -3.403554420192 | 4.546029036817 |
| H | -1.794365856325 | -2.868416137029 | 5.518214227040 |
| O | -2.156794742194 | 9.608583695766 | -1.189721066713 |
| C | -1.819013362627 | 10.352487892793 | -2.350137151737 |
| H | -0.729865322111 | 10.448476383852 | -2.464146661251 |
| H | -2.226437886628 | 9.888955157994 | -3.259814413924 |
| H | -2.253428697490 | 11.345526616322 | -2.232051153881 |
| O | -2.139744619720 | -4.536348915491 | 4.316648214105 |
| C | -1.819904837963 | -5.522494908749 | 5.287163097244 |

| | | | |
|---|---|---|---|
| H | -0.742533786931 | -5.739804401631 | 5.300241662202 |
| H | -2.126503460358 | -5.209679359834 | 6.295323235957 |
| H | -2.362582703106 | -6.428016940166 | 5.014914542560 |

Cam_TS3C-F4000

($E_F$ = -930.58403049 a.u.; $G_F$ = -930.20874739 a.u.)

0 1

| | | | |
|---|---|---|---|
| C | -0.780299288590 | 1.707264054645 | -3.865086228052 |
| C | -0.474488327989 | 2.504210952205 | -4.933192192959 |
| C | -3.531912062041 | 1.447094577525 | 1.698003143281 |
| C | -3.161972080592 | 1.452537418061 | -0.353389911819 |
| C | -1.859655719231 | 1.418655871246 | -1.254969222920 |
| C | -1.934587618843 | 2.006061196585 | -2.793282729619 |
| H | -0.941999048134 | 3.478027127505 | -5.064104632208 |
| H | -2.588223387964 | 1.251714803515 | 2.195669059154 |
| H | -4.005463194793 | 0.911254094018 | -0.772090976759 |
| H | -1.044249555665 | 1.938773447763 | -0.746075641249 |
| H | -1.547532636970 | 0.374386455891 | -1.342274681784 |
| H | -2.100567840998 | 3.085656265800 | -2.734756553028 |
| H | -2.849302243468 | 1.579112139470 | -3.221814307350 |
| C | -3.580695506261 | 2.517412124700 | 0.650131086038 |
| C | -4.972574474586 | 3.105462713048 | 0.409696685066 |
| H | -4.954857086084 | 3.740450387969 | -0.479967013526 |
| H | -5.712996919933 | 2.317189440545 | 0.260785962530 |
| H | -5.292681852907 | 3.706973339628 | 1.265895460134 |
| C | -2.548836182460 | 3.622913027167 | 0.884040852988 |

| | | | |
|---|---|---|---|
| H | -2.441469182390 | 4.228790791251 | -0.022259851116 |
| H | -2.861794928665 | 4.273007645513 | 1.705649049140 |
| H | -1.565137538530 | 3.214387731420 | 1.128838214717 |
| O | 0.447183497489 | 2.166323357077 | -5.970884321209 |
| O | -4.686368452322 | 1.094229829803 | 2.431089785749 |
| C | -0.126188846027 | 0.353545253461 | -3.739160496345 |
| H | 0.447112982859 | 0.276669140205 | -2.808847265388 |
| H | -0.880509703108 | -0.442057303249 | -3.710101510478 |
| H | 0.547846844999 | 0.158729541323 | -4.573381703302 |
| C | 0.630679004832 | 3.076821091275 | -7.132243145150 |
| H | 0.977768467227 | 4.049250595993 | -6.770701666190 |
| H | -0.331454224727 | 3.210283458708 | -7.635242974359 |
| C | 1.686692797138 | 2.544344433695 | -8.215869358329 |
| H | 2.655667854623 | 2.408262817113 | -7.720528553719 |
| H | 1.345246297984 | 1.569645788230 | -8.584823972434 |
| C | -4.592009016514 | 0.506887081217 | 3.801286709048 |
| H | -3.755165008977 | 0.975831787135 | 4.325656693464 |
| H | -4.400141810032 | -0.564521476386 | 3.707967279454 |
| C | -5.918326135967 | 0.710603046072 | 4.683479214311 |
| H | -6.107518224105 | 1.786539856403 | 4.780595124890 |
| H | -6.762456085146 | 0.256001076082 | 4.151178385582 |
| O | 1.803507635486 | 3.500140886436 | -9.320553625826 |
| C | 2.716093482493 | 3.295840297626 | -10.509407013969 |
| H | 3.760729115721 | 3.237785397261 | -10.189587867353 |
| H | 2.436785182522 | 2.393112732722 | -11.060764276305 |
| H | 2.549036750605 | 4.182921103652 | -11.115582968277 |

| | | | |
|---|---|---|---|
| O | -5.748802500925 | 0.095955249076 | 6.001380795183 |
| C | -6.818455422499 | 0.107419315598 | 7.072551244124 |
| H | -7.061286920641 | 1.133341938756 | 7.364279263652 |
| H | -7.716397090628 | -0.412859756325 | 6.726798250682 |
| H | -6.357762160638 | -0.428501542426 | 7.898728897124 |

Cam_Int2C-F4000

($E_F$ = -930.59651628 a.u.; $G_F$ = -930.21925561 a.u.)

0 1

| | | | |
|---|---|---|---|
| C | -0.901297224606 | 1.654193239991 | -3.737783921864 |
| C | -0.518585867341 | 2.482752710383 | -4.755774143868 |
| C | -3.337557601637 | 1.345045188299 | 1.385386086038 |
| C | -3.255995441123 | 1.480383584733 | -0.209644677537 |
| C | -1.974665827639 | 1.311825821475 | -1.153684821973 |
| C | -1.994788702044 | 2.014371157687 | -2.622267660030 |
| H | -0.869417851788 | 3.511463236740 | -4.808795312953 |
| H | -2.378890814028 | 1.197639719561 | 1.882032968066 |
| H | -4.161046447121 | 1.116559586962 | -0.691554034725 |
| H | -1.087569876529 | 1.661833258054 | -0.619787104880 |
| H | -1.835513093074 | 0.237567538947 | -1.302379081461 |
| H | -2.015819833458 | 3.099076777486 | -2.482219798810 |
| H | -2.962170849388 | 1.747283201671 | -3.065670494896 |
| C | -3.527892463888 | 2.677265893822 | 0.698290584610 |
| C | -4.936001782427 | 3.245691948480 | 0.670474751986 |
| H | -5.071127177887 | 3.895394409183 | -0.199797680524 |
| H | -5.681271728758 | 2.449827060284 | 0.631704225536 |

| | | | |
|---|---|---|---|
| H | -5.128977734765 | 3.840222538643 | 1.569649210277 |
| C | -2.445146159757 | 3.727172951886 | 0.876832070969 |
| H | -2.433628340900 | 4.418480266907 | 0.027454190370 |
| H | -2.626004008712 | 4.310995915082 | 1.784779560784 |
| H | -1.450302192989 | 3.284195166742 | 0.958255027556 |
| O | 0.342307561133 | 2.113993689564 | -5.832544474851 |
| O | -4.424193400880 | 0.688466659180 | 2.110923237177 |
| C | -0.412329468499 | 0.227215240613 | -3.717652317962 |
| H | 0.156769356707 | 0.016747776145 | -2.805526307080 |
| H | -1.255720770390 | -0.473829523935 | -3.731034690665 |
| H | 0.225474765825 | 0.013897913715 | -4.575444883776 |
| C | 0.611883948697 | 3.074669696580 | -6.936078796987 |
| H | 1.066901823520 | 3.977402342918 | -6.518144599272 |
| H | -0.335556463446 | 3.344323391931 | -7.411413537624 |
| C | 1.591087704117 | 2.503992909765 | -8.071153855591 |
| H | 2.546087013727 | 2.234245073221 | -7.604290077380 |
| H | 1.142444102681 | 1.596801247119 | -8.493621565895 |
| C | -4.566664588012 | 1.055521512215 | 3.547167945302 |
| H | -4.810953497835 | 2.119703681319 | 3.620925642465 |
| H | -3.610553524722 | 0.884373266161 | 4.053721795926 |
| C | -5.693355453310 | 0.232454624912 | 4.338708011339 |
| H | -6.660349960729 | 0.401805995380 | 3.849401232091 |
| H | -5.455127176413 | -0.836232902043 | 4.274815382720 |
| O | 1.795194744396 | 3.511208132654 | -9.115494929509 |
| C | 2.667534843130 | 3.289837103420 | -10.331408342681 |
| H | 3.704001426290 | 3.103840170967 | -10.034895312866 |

| | | | |
|---|---|---|---|
| H | 2.289020653987 | 2.457893744384 | -10.932571063685 |
| H | 2.586689551934 | 4.226290636403 | -10.877924766621 |
| O | -5.746923477496 | 0.663743339149 | 5.740113909178 |
| C | -6.704608910936 | 0.086331738783 | 6.757110347081 |
| H | -7.741861958442 | 0.264890392594 | 6.458011756866 |
| H | -6.528000987894 | -0.985105317694 | 6.891237598091 |
| H | -6.469710759196 | 0.630517277496 | 7.668768453220 |

Cam_Int3C-F4000

($E_F$ = -930.62424940 a.u.; $G_F$ = -930.25416985 a.u.)

0 1

| | | | |
|---|---|---|---|
| C | -0.618246038777 | 2.055842513113 | -4.068919877770 |
| C | -0.634976082287 | 2.239025735250 | -5.423734102346 |
| C | -4.354572060289 | 0.902489133168 | 1.648325257745 |
| C | -3.108803500773 | 1.408984957196 | -0.701056115291 |
| C | -1.980939964297 | 2.027489569883 | -1.613504239222 |
| C | -1.882876093216 | 1.626685715716 | -3.183494734123 |
| H | -1.565763198751 | 2.207147521792 | -5.986458597181 |
| H | -4.251699016765 | -0.177983318993 | 1.549344996525 |
| H | -3.886376498111 | 0.827128862436 | -1.190673845644 |
| H | -2.049004246907 | 3.123249759184 | -1.554887098209 |
| H | -1.015491652098 | 1.785049715535 | -1.153777738256 |
| H | -2.796425105672 | 1.963863063443 | -3.681909990830 |
| H | -1.905069100703 | 0.530450580699 | -3.214984730237 |
| C | -3.394706137381 | 1.840915419730 | 0.804526600985 |
| C | -4.044536883646 | 3.248744848824 | 0.769675281830 |

| | | | |
|---|---|---|---|
| H | -3.376472424658 | 3.973089154499 | 0.296744508438 |
| H | -4.983084527640 | 3.225551056688 | 0.210747718021 |
| H | -4.261149578947 | 3.587986927917 | 1.785814386564 |
| C | -2.050689231513 | 1.928870858766 | 1.572116286814 |
| H | -1.375131079202 | 2.651246364457 | 1.104359825973 |
| H | -2.230728732258 | 2.244628528132 | 2.602330451194 |
| H | -1.551401176797 | 0.956542100843 | 1.591157900649 |
| O | 0.517315604343 | 2.472341653249 | -6.232274777580 |
| O | -4.762353730522 | 1.342713415877 | 2.932473159075 |
| C | 0.698605328045 | 2.082895695104 | -3.333020870230 |
| H | 0.710518161767 | 2.870714424627 | -2.571823072302 |
| H | 0.871629579108 | 1.135228609852 | -2.808655621839 |
| H | 1.530962627698 | 2.255095622054 | -4.015327470962 |
| C | 0.385269472768 | 2.575456182475 | -7.710636684153 |
| H | -0.284776169100 | 3.406139827520 | -7.950464052449 |
| H | -0.050389440644 | 1.648239406353 | -8.093771550730 |
| C | 1.775342596416 | 2.822180954530 | -8.472211418042 |
| H | 2.219039736302 | 3.751500039561 | -8.095026034830 |
| H | 2.453218517824 | 1.992929499197 | -8.236426193675 |
| C | -5.540846619144 | 0.423570464630 | 3.814218996078 |
| H | -4.931517834664 | -0.463452891017 | 4.009836196425 |
| H | -6.444998710679 | 0.120680557233 | 3.280429974739 |
| C | -5.976876485454 | 1.055048235413 | 5.222224085881 |
| H | -5.074321062566 | 1.362797794294 | 5.764060671808 |
| H | -6.588553201097 | 1.945103148867 | 5.031669055893 |
| O | 1.551596409121 | 2.908568531910 | -9.917632054512 |

| | | | |
|---|---|---|---|
| C | 2.658813152003 | 3.136361828335 | -10.922850562336 |
| H | 3.152594405802 | 4.095918805247 | -10.743409492546 |
| H | 3.387575831151 | 2.321250565709 | -10.886757457274 |
| H | 2.139285609300 | 3.144711381131 | -11.877949767931 |
| O | -6.731618776583 | 0.068564503471 | 5.999243240627 |
| C | -7.297102356350 | 0.341512837442 | 7.375809711962 |
| H | -6.495994702993 | 0.573488933891 | 8.083697055599 |
| H | -8.022263197932 | 1.159798483512 | 7.340530123041 |
| H | -7.785305275572 | -0.593032618904 | 7.641112301050 |

Cam_TS3C-F3000

($E_F$ = -930.12314988 a.u.; $G_F$ = -929.74694894 a.u.)

0 1

| | | | |
|---|---|---|---|
| C | -0.814268652954 | 1.673428512697 | -3.844660348746 |
| C | -0.485458316296 | 2.501772038767 | -4.869984404646 |
| C | -3.510716689548 | 1.492369385319 | 1.714903434792 |
| C | -3.121865268048 | 1.461769030954 | -0.419195587348 |
| C | -1.829392539787 | 1.437075079592 | -1.282456282824 |
| C | -1.954424704112 | 1.974037922157 | -2.803304797962 |
| H | -0.931844765763 | 3.489342687425 | -4.968883572569 |
| H | -2.563988346816 | 1.263485751659 | 2.191781219418 |
| H | -3.940913488406 | 0.856069032038 | -0.796002114043 |
| H | -1.031460969570 | 2.000784149499 | -0.791698998728 |
| H | -1.478106518408 | 0.402342190278 | -1.343176622680 |
| H | -2.153128407176 | 3.049780765075 | -2.773934870483 |
| H | -2.862429040308 | 1.507824152304 | -3.207451497088 |

| | | | |
|---|---|---|---|
| C | -3.552146223458 | 2.498164353811 | 0.603739551780 |
| C | -4.950713644494 | 3.071057972450 | 0.348473850388 |
| H | -4.931857938847 | 3.695720102569 | -0.548209483922 |
| H | -5.681340919335 | 2.273343644057 | 0.201220289268 |
| H | -5.283686729117 | 3.678102848716 | 1.195784841031 |
| C | -2.538640198850 | 3.627432424946 | 0.820022014428 |
| H | -2.432361324525 | 4.213278329033 | -0.099558490908 |
| H | -2.870265730446 | 4.290907658540 | 1.623190721734 |
| H | -1.551144036358 | 3.240622454335 | 1.084176251727 |
| O | 0.440770787153 | 2.181122309604 | -5.886074493595 |
| O | -4.658161767264 | 1.169308955228 | 2.431240392274 |
| C | -0.194405745121 | 0.300487176283 | -3.761841317059 |
| H | 0.411312699605 | 0.191947835813 | -2.855505208346 |
| H | -0.970070410166 | -0.473328372285 | -3.716659371227 |
| H | 0.443760849977 | 0.100778159065 | -4.622552100926 |
| C | 0.561928359727 | 3.076193835880 | -7.040925350571 |
| H | 0.846047532893 | 4.077737589835 | -6.700834556059 |
| H | -0.401647423896 | 3.142692899289 | -7.556425557614 |
| C | 1.643192047861 | 2.575912281514 | -8.072639041708 |
| H | 2.615299180405 | 2.507429444677 | -7.567045218344 |
| H | 1.365728862385 | 1.572995674357 | -8.422576129324 |
| C | -4.561510881282 | 0.498960630376 | 3.738278321739 |
| H | -3.682414073948 | 0.873776455417 | 4.270725798979 |
| H | -4.448482008449 | -0.576904878033 | 3.580410932212 |
| C | -5.838136271003 | 0.758088475733 | 4.628185736119 |
| H | -5.950154020777 | 1.839757067387 | 4.779235462096 |

| | | | |
|---|---|---|---|
| H | -6.725200772204 | 0.391252545747 | 4.095689552169 |
| O | 1.702799186177 | 3.509171302103 | -9.179780375150 |
| C | 2.632217702742 | 3.287735805940 | -10.308928170511 |
| H | 3.673119394418 | 3.283655005363 | -9.966530947588 |
| H | 2.408681031906 | 2.346414890745 | -10.823402555259 |
| H | 2.461585206813 | 4.129226864097 | -10.977573198246 |
| O | -5.676724204541 | 0.074126613007 | 5.893871985323 |
| C | -6.730123190518 | 0.143718820344 | 6.931574207063 |
| H | -6.893324518737 | 1.177292281418 | 7.256181121716 |
| H | -7.670509360492 | -0.282396254789 | 6.565131124287 |
| H | -6.345212106414 | -0.451983298313 | 7.756750350230 |

Cam_Int2C-F3000

($E_F$ = -930.14871828 a.u.; $G_F$ = -929.76876579 a.u.)

0 1

| | | | |
|---|---|---|---|
| C | -0.967892225288 | 1.613766092632 | -3.685694868208 |
| C | -0.543193996566 | 2.468417913676 | -4.652427038987 |
| C | -3.283335708620 | 1.382428251346 | 1.327676706489 |
| C | -3.238955294754 | 1.492618926581 | -0.232343926748 |
| C | -1.975135221325 | 1.303191842128 | -1.148852099498 |
| C | -2.033511882575 | 1.989701808189 | -2.591034568891 |
| H | -0.846272948698 | 3.513645525547 | -4.659695009334 |
| H | -2.318772286998 | 1.249222808178 | 1.818718539269 |
| H | -4.145953902021 | 1.113151786477 | -0.698709944384 |
| H | -1.082869190933 | 1.658259853524 | -0.625976441886 |
| H | -1.837099589246 | 0.226764965306 | -1.286468734286 |

| | | | |
|---|---|---|---|
| H | -2.053771096702 | 3.076058192622 | -2.460021261764 |
| H | -3.009639634321 | 1.718737570171 | -3.015770933252 |
| C | -3.503352039428 | 2.714940814246 | 0.644815561202 |
| C | -4.916912643699 | 3.268363983143 | 0.633217613074 |
| H | -5.076888290712 | 3.895643583273 | -0.249396631555 |
| H | -5.654738393564 | 2.464595084249 | 0.627108225549 |
| H | -5.098946305281 | 3.883420448040 | 1.520840883020 |
| C | -2.426725784713 | 3.775622609556 | 0.786811262422 |
| H | -2.435844992072 | 4.455345515269 | -0.071969362419 |
| H | -2.596358995485 | 4.371714481907 | 1.689101527205 |
| H | -1.426445199808 | 3.343479705155 | 0.857713851040 |
| O | 0.306447634591 | 2.104784620408 | -5.718057697220 |
| O | -4.349067298142 | 0.712114798735 | 2.034590750379 |
| C | -0.549920353662 | 0.164947267324 | -3.726602186886 |
| H | 0.035595846544 | -0.105048228882 | -2.840938962253 |
| H | -1.427600520532 | -0.492578264754 | -3.736522792384 |
| H | 0.051712498374 | -0.049500108018 | -4.609649047519 |
| C | 0.552029369609 | 3.074886565136 | -6.790258773111 |
| H | 0.996794407749 | 3.982422609898 | -6.368671546710 |
| H | -0.396239450457 | 3.340082204256 | -7.268673269766 |
| C | 1.524072734023 | 2.510615967709 | -7.895034381039 |
| H | 2.481752525204 | 2.246116471203 | -7.428034092366 |
| H | 1.086726247094 | 1.599435804647 | -8.323637384533 |
| C | -4.496398108762 | 1.107675262769 | 3.440073275635 |
| H | -4.762123329026 | 2.168707446662 | 3.497306175610 |
| H | -3.541897608250 | 0.966041506707 | 3.961050847546 |

| | | | |
|---|---|---|---|
| C | -5.596420195291 | 0.277498166929 | 4.204408155468 |
| H | -6.566028154513 | 0.420697135895 | 3.709408755062 |
| H | -5.339766403205 | -0.788855030298 | 4.155339846277 |
| O | 1.710386151671 | 3.521788564551 | -8.916028317089 |
| C | 2.577591023841 | 3.266470076104 | -10.086917550506 |
| H | 3.610453830267 | 3.073290103915 | -9.776370133179 |
| H | 2.204298952877 | 2.421132363944 | -10.675691109935 |
| H | 2.528033525888 | 4.181811845832 | -10.673325068300 |
| O | -5.652482879358 | 0.730473629707 | 5.581698453724 |
| C | -6.601060541258 | 0.128189228973 | 6.542192053374 |
| H | -7.637491814680 | 0.277079199181 | 6.218617031001 |
| H | -6.405267105841 | -0.942368679729 | 6.670824545299 |
| H | -6.422368429348 | 0.655617915348 | 7.477252745791 |

Cam_Int3C-F3000

($E_F$ = -930.14753855 a.u.; $G_F$ = -929.77416771 a.u.)

0 1

| | | | |
|---|---|---|---|
| C | -0.632428836752 | 1.988473111901 | -3.988118336734 |
| C | -0.674116278576 | 2.268409771911 | -5.316767205477 |
| C | -4.286109613886 | 0.933332358049 | 1.574251751075 |
| C | -3.078737319320 | 1.438755556525 | -0.707176172011 |
| C | -1.954673009071 | 2.035592311604 | -1.592284266474 |
| C | -1.894594493249 | 1.613456181003 | -3.128959392323 |
| H | -1.618650530757 | 2.350681780389 | -5.851053127153 |
| H | -4.137359163875 | -0.142587509946 | 1.482078534161 |
| H | -3.845226408714 | 0.840002158840 | -1.193647747228 |

| | | | |
|---|---|---|---|
| H | -1.998811799125 | 3.133878993987 | -1.540526591837 |
| H | -0.991594055206 | 1.777673504269 | -1.133852912624 |
| H | -2.797273173130 | 1.978445116416 | -3.628718423347 |
| H | -1.958538146741 | 0.517183961528 | -3.147395816677 |
| C | -3.372700202619 | 1.898604430034 | 0.758658991457 |
| C | -4.079807837570 | 3.280325008471 | 0.694330102261 |
| H | -3.439658820396 | 4.019748329603 | 0.206456133185 |
| H | -5.015378793225 | 3.207293169223 | 0.134731873213 |
| H | -4.311245451140 | 3.632267400849 | 1.702698829857 |
| C | -2.039595496330 | 2.059562328220 | 1.537108635900 |
| H | -1.394088787837 | 2.806765704421 | 1.066431816597 |
| H | -2.244990812603 | 2.379659983159 | 2.560946680044 |
| H | -1.496043893398 | 1.111707200001 | 1.573643052890 |
| O | 0.466127617765 | 2.477111083051 | -6.120083550961 |
| O | -4.713219236214 | 1.369489439793 | 2.836982645750 |
| C | 0.699006585017 | 1.853658129473 | -3.291428458145 |
| H | 0.824177803201 | 2.621251037127 | -2.519731731019 |
| H | 0.776887377326 | 0.883042028864 | -2.787052797239 |
| H | 1.525567381827 | 1.944077494507 | -3.995946350394 |
| C | 0.299850311860 | 2.581066401356 | -7.573722659087 |
| H | -0.375656831876 | 3.410763826741 | -7.806948326038 |
| H | -0.137809180823 | 1.654799024271 | -7.959491644231 |
| C | 1.668571808651 | 2.829631718571 | -8.314570808959 |
| H | 2.114763074088 | 3.759823653212 | -7.939236139450 |
| H | 2.353462223556 | 2.002615981011 | -8.086099278414 |
| C | -5.451604481068 | 0.428854202894 | 3.692007920814 |

| | | | |
|---|---|---|---|
| H | -4.815588592221 | -0.437622954977 | 3.900483354165 |
| H | -6.343043945836 | 0.088010601509 | 3.157674569655 |
| C | -5.899380694134 | 1.065342062992 | 5.062123170916 |
| H | -5.010950428288 | 1.411132146280 | 5.606655559227 |
| H | -6.540572173800 | 1.933619375553 | 4.861891319658 |
| O | 1.421730464598 | 2.914840966191 | -9.739838097607 |
| C | 2.532875777105 | 3.141284151896 | -10.689692955655 |
| H | 3.029239421795 | 4.098158716255 | -10.493216455343 |
| H | 3.265123907000 | 2.327724581163 | -10.639242451324 |
| H | 2.060354974646 | 3.159187906313 | -11.669796873793 |
| O | -6.613653196509 | 0.062629765077 | 5.826333433012 |
| C | -7.164753293926 | 0.375389767466 | 7.163154222399 |
| H | -6.368150499194 | 0.657089478247 | 7.860709461344 |
| H | -7.906318021349 | 1.179812009412 | 7.104188654916 |
| H | -7.639184959311 | -0.547790619042 | 7.489898307759 |

Cam_TS3C-F2500

($E_F$ = -929.89597364 a.u.; $G_F$ = -929.51844085 a.u.)

0 1

| | | | |
|---|---|---|---|
| C | -0.836415979691 | 1.652952864922 | -3.841103235643 |
| C | -0.490983010774 | 2.500675793071 | -4.839150334392 |
| C | -3.496843513428 | 1.514846261472 | 1.723304716048 |
| C | -3.108751865785 | 1.470143250554 | -0.451194008554 |
| C | -1.821767538641 | 1.442438079669 | -1.300170985299 |
| C | -1.971312205387 | 1.953533801704 | -2.813836299276 |
| H | -0.926696151904 | 3.494782172232 | -4.919814115134 |

| | | | |
|---|---|---|---|
| H | -2.550424636197 | 1.259093441781 | 2.186853603261 |
| H | -3.917894013388 | 0.836779468486 | -0.803373318477 |
| H | -1.029340358879 | 2.024285797619 | -0.821655769209 |
| H | -1.456873545594 | 0.411144598445 | -1.347261016128 |
| H | -2.185675151627 | 3.026689683310 | -2.798520754459 |
| H | -2.875259209025 | 1.468132589601 | -3.205505325192 |
| C | -3.533011653926 | 2.492409184585 | 0.586043247721 |
| C | -4.932818091219 | 3.066161360603 | 0.330920121466 |
| H | -4.914088267580 | 3.688231235522 | -0.567475051043 |
| H | -5.663823482208 | 2.268376192897 | 0.185843156323 |
| H | -5.264792828259 | 3.675480717712 | 1.176965256166 |
| C | -2.521159067204 | 3.626860980666 | 0.791848978808 |
| H | -2.415647567554 | 4.205130886721 | -0.132619239438 |
| H | -2.854974866036 | 4.296285933947 | 1.589032213061 |
| H | -1.533086533141 | 3.244641293147 | 1.060561918027 |
| O | 0.446351357324 | 2.195064037706 | -5.839194269550 |
| O | -4.643481135702 | 1.206378203917 | 2.431932904883 |
| C | -0.234591209808 | 0.271028231343 | -3.781539918095 |
| H | 0.389240888556 | 0.146082174380 | -2.889675313417 |
| H | -1.020516975695 | -0.491475141024 | -3.725789502444 |
| H | 0.382841110157 | 0.069670114967 | -4.656910801002 |
| C | 0.517038251837 | 3.065913961388 | -7.005706665176 |
| H | 0.735516421562 | 4.092373568285 | -6.690604910651 |
| H | -0.443191545669 | 3.060529995656 | -7.532314463797 |
| C | 1.632292008820 | 2.601241038149 | -7.999014791397 |
| H | 2.601288234626 | 2.605606500750 | -7.481991364690 |

| | | | |
|---|---|---|---|
| H | 1.421103272077 | 1.573334634995 | -8.323194578552 |
| C | -4.545746051716 | 0.501275226028 | 3.709903785118 |
| H | -3.653750748475 | 0.839919905273 | 4.245770390141 |
| H | -4.460178300841 | -0.573110648944 | 3.524343063982 |
| C | -5.803068848290 | 0.775561871988 | 4.601465542703 |
| H | -5.887650245539 | 1.856797468045 | 4.775323823306 |
| H | -6.703754783009 | 0.442494290830 | 4.068625541258 |
| O | 1.644163292730 | 3.503431288124 | -9.124167447902 |
| C | 2.598679526577 | 3.285044218341 | -10.214607428677 |
| H | 3.632341818274 | 3.346307883114 | -9.853891207524 |
| H | 2.436070549756 | 2.312249376712 | -10.693712235106 |
| H | 2.407299342840 | 4.085322996940 | -10.927394318148 |
| O | -5.646121285105 | 0.063655918126 | 5.844231815255 |
| C | -6.693714782517 | 0.159285501808 | 6.866301102569 |
| H | -6.824418633949 | 1.194817340269 | 7.201803882951 |
| H | -7.648328969304 | -0.227114125912 | 6.490462825263 |
| H | -6.343861387453 | -0.456880817893 | 7.692607275361 |

Cam_Int2C-F2500

($E_F$ = -929.92889740 a.u.; $G_F$ = -929.54776088 a.u.)

0 1

| | | | |
|---|---|---|---|
| C | -1.006541453771 | 1.591217899080 | -3.669356580345 |
| C | -0.549381202252 | 2.461752333089 | -4.600508843097 |
| C | -3.254127721576 | 1.400759924559 | 1.304408005827 |
| C | -3.229830317557 | 1.495046720480 | -0.241871258962 |
| C | -1.975807650322 | 1.296492639418 | -1.149751116296 |

| | | | |
|---|---|---|---|
| C | -2.056326582904 | 1.975052603984 | -2.581006192266 |
| H | -0.819979841674 | 3.515688575696 | -4.576749455059 |
| H | -2.285824383767 | 1.276855310165 | 1.791239049996 |
| H | -4.138115687312 | 1.104432323982 | -0.696450453746 |
| H | -1.080102102381 | 1.655823921072 | -0.635372040264 |
| H | -1.837474584107 | 0.219167910749 | -1.281805050600 |
| H | -2.075040232880 | 3.062099875037 | -2.454375599139 |
| H | -3.037606097028 | 1.702276141032 | -2.993976861287 |
| C | -3.491387742942 | 2.730074570101 | 0.618699558571 |
| C | -4.908126011191 | 3.274710566835 | 0.615848223521 |
| H | -5.082519824028 | 3.887441983505 | -0.274301071848 |
| H | -5.641113824383 | 2.466574827813 | 0.629987338373 |
| H | -5.084013961929 | 3.902603501591 | 1.495701526052 |
| C | -2.419247538678 | 3.797626573626 | 0.738456235174 |
| H | -2.442054388167 | 4.470374335712 | -0.125570852956 |
| H | -2.581795609002 | 4.400602864076 | 1.637540210037 |
| H | -1.415600919796 | 3.372243373168 | 0.801626650375 |
| O | 0.300501401240 | 2.104081039174 | -5.658155337864 |
| O | -4.308340929377 | 0.724173522386 | 2.005898672227 |
| C | -0.636547674779 | 0.131270268535 | -3.751189408108 |
| H | -0.037345021939 | -0.177120093708 | -2.887435723630 |
| H | -1.535311027047 | -0.497004064673 | -3.751977881225 |
| H | -0.064097296300 | -0.082581517623 | -4.653578742195 |
| C | 0.519103735909 | 3.073489512719 | -6.724790307945 |
| H | 0.938798139761 | 3.995763568299 | -6.308101234737 |
| H | -0.432980486140 | 3.312335128236 | -7.210310202553 |

| | | | |
|---|---|---|---|
| C | 1.504250291932 | 2.520900022310 | -7.806673974750 |
| H | 2.465459790601 | 2.281917309559 | -7.332114242984 |
| H | 1.091730790016 | 1.595690784754 | -8.231029009185 |
| C | -4.457670566307 | 1.134161245989 | 3.397380455886 |
| H | -4.733654419722 | 2.193388285887 | 3.445730295493 |
| H | -3.503998178203 | 1.006641400855 | 3.924465431021 |
| C | -5.545867361997 | 0.300115933142 | 4.149676363430 |
| H | -6.515784417357 | 0.430265429169 | 3.650641486469 |
| H | -5.279467746149 | -0.764644838695 | 4.108058634631 |
| O | 1.668801744546 | 3.526612681695 | -8.826978762775 |
| C | 2.544310040655 | 3.257794158825 | -9.971323113181 |
| H | 3.576838001681 | 3.082491500610 | -9.646701940848 |
| H | 2.190166762265 | 2.393606998270 | -10.545793492737 |
| H | 2.496535990250 | 4.155368036746 | -10.585445018655 |
| O | -5.606399341726 | 0.762701159465 | 5.516195288309 |
| C | -6.551971864776 | 0.145461881698 | 6.449016145539 |
| H | -7.586745369936 | 0.279114697385 | 6.111227917853 |
| H | -6.344782517842 | -0.924172924539 | 6.573876736184 |
| H | -6.402160065139 | 0.663788296025 | 7.394658452986 |

Cam_Int3C-F2500

($E_F$ = -929.91339329 a.u.; $G_F$ = -929.53890838 a.u.)

0 1

| | | | |
|---|---|---|---|
| C | -0.638186448349 | 1.958151637530 | -3.952836621089 |
| C | -0.692794577695 | 2.268973178110 | -5.269791633207 |
| C | -4.262975114653 | 0.952643429340 | 1.536383715256 |

| | | | |
|---|---|---|---|
| C | -3.068118996402 | 1.457446963969 | -0.711632394814 |
| C | -1.945754032821 | 2.042641030699 | -1.586457861262 |
| C | -1.893662740396 | 1.592993352237 | -3.103115250039 |
| H | -1.641768011486 | 2.376581043825 | -5.791673219732 |
| H | -4.108764363448 | -0.121993651053 | 1.437350296700 |
| H | -3.839525835965 | 0.865499249700 | -1.198638159914 |
| H | -1.989192036185 | 3.142225376932 | -1.552596295963 |
| H | -0.983612798225 | 1.791718370676 | -1.121816342424 |
| H | -2.796782234226 | 1.949104817242 | -3.608865356440 |
| H | -1.957176685026 | 0.496052310503 | -3.102410790607 |
| C | -3.357567083967 | 1.924134384950 | 0.740493994594 |
| C | -4.070660690640 | 3.303691146361 | 0.674810263900 |
| H | -3.433011516248 | 4.044631896142 | 0.186011603870 |
| H | -5.005855005807 | 3.226238109051 | 0.115168555079 |
| H | -4.303268352700 | 3.655943067484 | 1.682739652752 |
| C | -2.027219810010 | 2.092059103519 | 1.523425544902 |
| H | -1.385830592295 | 2.845795649252 | 1.057754365778 |
| H | -2.238455516258 | 2.406819599818 | 2.547630187080 |
| H | -1.477018325418 | 1.147952807204 | 1.558184075624 |
| O | 0.438974924054 | 2.484113936068 | -6.070467208456 |
| O | -4.690470545901 | 1.381381777332 | 2.793930834351 |
| C | 0.697113794585 | 1.787843256023 | -3.271860823045 |
| H | 0.853225134129 | 2.552250210194 | -2.502639570056 |
| H | 0.754169766329 | 0.815754272052 | -2.767754721261 |
| H | 1.517123619020 | 1.856130324654 | -3.986414551888 |
| C | 0.257231758246 | 2.569785181718 | -7.514506368484 |

| | | | |
|---|---|---|---|
| H | -0.434711074192 | 3.384721518566 | -7.753918459151 |
| H | -0.164514970591 | 1.632279144258 | -7.891874490325 |
| C | 1.613877351649 | 2.835553475136 | -8.246234320075 |
| H | 2.044395766903 | 3.776725129603 | -7.878666067859 |
| H | 2.314975602491 | 2.023158815287 | -8.012265441700 |
| C | -5.423189074594 | 0.433632330474 | 3.629486467762 |
| H | -4.787526804107 | -0.434617183240 | 3.834014723260 |
| H | -6.314612712912 | 0.094615110367 | 3.093139243995 |
| C | -5.863708716183 | 1.067182931192 | 4.989692412385 |
| H | -4.975100842045 | 1.411710603356 | 5.535712351330 |
| H | -6.504993216131 | 1.936940083663 | 4.793632516407 |
| O | 1.359798553566 | 2.903784140968 | -9.664079735408 |
| C | 2.474111628491 | 3.139374549720 | -10.586576066882 |
| H | 2.955130371494 | 4.104386374490 | -10.387551176220 |
| H | 3.219850304121 | 2.338440802084 | -10.518552398950 |
| H | 2.027534624351 | 3.146113688868 | -11.579346218835 |
| O | -6.573517538176 | 0.062820140936 | 5.742700708563 |
| C | -7.110173398047 | 0.394472408808 | 7.065792330318 |
| H | -6.308919053494 | 0.680994084378 | 7.757227395156 |
| H | -7.845558180822 | 1.205230248668 | 7.002324554902 |
| H | -7.592691480575 | -0.516574711412 | 7.415318201569 |

Cam_TS3C-F2000

($E_F$ = -929.67067114 a.u.; $G_F$ = -929.29277164 a.u.)

0 1

| | | | |
|---|---|---|---|
| C | -0.857220537238 | 1.630387411891 | -3.845199597933 |

| | | | |
|---|---|---|---|
| C | -0.505104978314 | 2.494868439768 | -4.820754087758 |
| C | -3.483990178247 | 1.536275042617 | 1.739678846627 |
| C | -3.099641293437 | 1.483095656789 | -0.489266734982 |
| C | -1.818393592587 | 1.451542900321 | -1.325999453441 |
| C | -1.995179584351 | 1.922315835437 | -2.837116616547 |
| H | -0.945910864898 | 3.487524622680 | -4.892233939819 |
| H | -2.540887770922 | 1.231556305323 | 2.179597947521 |
| H | -3.896244045955 | 0.817635615883 | -0.810130011000 |
| H | -1.034494815751 | 2.059565591821 | -0.866404641278 |
| H | -1.434557835034 | 0.425622889715 | -1.351094577131 |
| H | -2.239230314326 | 2.989286706313 | -2.844186425502 |
| H | -2.886870056186 | 1.403211862637 | -3.214391565442 |
| C | -3.510636874747 | 2.482591394987 | 0.573689201592 |
| C | -4.910107545518 | 3.064363466248 | 0.323376315234 |
| H | -4.890244396369 | 3.688509606143 | -0.573409948664 |
| H | -5.644856587338 | 2.270169262883 | 0.177353423461 |
| H | -5.237384773959 | 3.672687422834 | 1.171899860264 |
| C | -2.496074773024 | 3.618472498056 | 0.771216240855 |
| H | -2.390473388639 | 4.191949803034 | -0.156269424258 |
| H | -2.828963788302 | 4.292199472169 | 1.564967886193 |
| H | -1.508451573071 | 3.236559693782 | 1.042185779488 |
| O | 0.447901537081 | 2.209379723711 | -5.801942047741 |
| O | -4.631670482846 | 1.246763143309 | 2.440837085720 |
| C | -0.250710252792 | 0.250523936582 | -3.794423230846 |
| H | 0.380850308087 | 0.124311629192 | -2.908188692652 |
| H | -1.034051369338 | -0.514238081313 | -3.735180774575 |

| | | | |
|---|---|---|---|
| H | 0.361264710777 | 0.054495931316 | -4.674811477528 |
| C | 0.478203742615 | 3.061555598540 | -6.974271061884 |
| H | 0.647510641773 | 4.103161361396 | -6.677551953291 |
| H | -0.477553490100 | 3.003236666379 | -7.506459374784 |
| C | 1.615051569317 | 2.625166757957 | -7.938401094943 |
| H | 2.578867945144 | 2.680569805826 | -7.413813270370 |
| H | 1.452002327214 | 1.582718706013 | -8.244842891474 |
| C | -4.534653680793 | 0.509420541266 | 3.690953457341 |
| H | -3.634226493244 | 0.817459723317 | 4.231702147674 |
| H | -4.469889250089 | -0.561958831726 | 3.479043600481 |
| C | -5.777196972375 | 0.792078543745 | 4.581229810330 |
| H | -5.840715238574 | 1.871583710209 | 4.776203798904 |
| H | -6.687236921121 | 0.487301968675 | 4.046458606932 |
| O | 1.594605324250 | 3.504174646638 | -9.073603384796 |
| C | 2.568554402955 | 3.284378189337 | -10.129957974941 |
| H | 3.593901875415 | 3.388099666120 | -9.753457675480 |
| H | 2.448078928681 | 2.291677847964 | -10.581463208953 |
| H | 2.372561423070 | 4.052822671826 | -10.876301677354 |
| O | -5.625725522340 | 0.055247963352 | 5.802752504499 |
| C | -6.669693228538 | 0.174936926349 | 6.808608588394 |
| H | -6.772297135711 | 1.211680350818 | 7.152335043268 |
| H | -7.634743700464 | -0.175944411658 | 6.422668015634 |
| H | -6.353859795236 | -0.457115584452 | 7.637125150258 |

Cam_Int2C-F2000

($E_F$ = -929.71157193 a.u.; $G_F$ = -929.32946528 a.u.)

0 1

| | | | |
|---|---|---|---|
| C | -1.048243960458 | 1.568395123853 | -3.657715087462 |
| C | -0.562170835793 | 2.453465510148 | -4.553885092623 |
| C | -3.225635625150 | 1.419829184709 | 1.282420616340 |
| C | -3.223818458628 | 1.500118499128 | -0.251472021116 |
| C | -1.982239392932 | 1.288820198966 | -1.155180321175 |
| C | -2.087702574287 | 1.955922620661 | -2.577915156239 |
| H | -0.809798075388 | 3.512210277652 | -4.503899401623 |
| H | -2.253219363776 | 1.300096145592 | 1.762680212451 |
| H | -4.136035715093 | 1.102538102885 | -0.691907858076 |
| H | -1.081253878560 | 1.653147780249 | -0.653436179815 |
| H | -1.844982377309 | 0.210195113598 | -1.279427345093 |
| H | -2.111074880859 | 3.043737994581 | -2.457853741814 |
| H | -3.071839829609 | 1.674765463241 | -2.979467814442 |
| C | -3.475448337185 | 2.747151836779 | 0.595109856695 |
| C | -4.892658263432 | 3.289825329021 | 0.604656853209 |
| H | -5.080097754826 | 3.890463555201 | -0.291138111696 |
| H | -5.624347640608 | 2.481057514648 | 0.638685678085 |
| H | -5.058015240430 | 3.929492301868 | 1.478058592946 |
| C | -2.402910919176 | 3.816302127621 | 0.691910382402 |
| H | -2.437292728397 | 4.482528306578 | -0.176789263098 |
| H | -2.554577938189 | 4.426395399969 | 1.588147575828 |
| H | -1.397949485538 | 3.392850776640 | 0.745928455973 |
| O | 0.294876507653 | 2.104501912413 | -5.599520148923 |
| O | -4.270443411750 | 0.740944586960 | 1.980235898498 |
| C | -0.712137704333 | 0.102811073359 | -3.773000070506 |

| | | | |
|---|---|---|---|
| H | -0.105546047732 | -0.235860854172 | -2.925894553084 |
| H | -1.624761899601 | -0.504944915277 | -3.770723866950 |
| H | -0.158563796914 | -0.105343957559 | -4.688417288751 |
| C | 0.483971356137 | 3.071624806433 | -6.663152131276 |
| H | 0.875199915968 | 4.009686788623 | -6.252762488311 |
| H | -0.471909691113 | 3.279916641465 | -7.155975762278 |
| C | 1.484673940090 | 2.532410547706 | -7.721549996325 |
| H | 2.448527815890 | 2.321939634821 | -7.237941847250 |
| H | 1.099763514328 | 1.592396698846 | -8.140371959250 |
| C | -4.417193963748 | 1.161762099362 | 3.359982326988 |
| H | -4.695355279259 | 2.220970906721 | 3.403129829940 |
| H | -3.464804922779 | 1.039059001282 | 3.891393441658 |
| C | -5.499950310138 | 0.328739024104 | 4.098461889477 |
| H | -6.469023188772 | 0.455212063989 | 3.595890523335 |
| H | -5.231522014120 | -0.736051598183 | 4.059496255528 |
| O | 1.627424537769 | 3.530757827312 | -8.743465667627 |
| C | 2.514050214082 | 3.248652206344 | -9.860403533230 |
| H | 3.545404905698 | 3.093370211913 | -9.519914618853 |
| H | 2.180846340056 | 2.365105736644 | -10.419046037338 |
| H | 2.468148985091 | 4.127137104762 | -10.502317340222 |
| O | -5.562406812161 | 0.794626242599 | 5.457006380473 |
| C | -6.509632585266 | 0.163778464101 | 6.359759322854 |
| H | -7.541253374106 | 0.291717930345 | 6.008118170141 |
| H | -6.298173769067 | -0.906853460391 | 6.474699955328 |
| H | -6.384592425096 | 0.666508432681 | 7.317781766557 |

Cam_Int3C-F2000

($E_F$ = -929.68183678 a.u.; $G_F$ = -929.30625486 a.u.)

0 1

| | | | |
|---|---|---|---|
| C | -0.641949090196 | 1.919467173335 | -3.920325194778 |
| C | -0.709753329713 | 2.276725463268 | -5.220549799828 |
| C | -4.236869581042 | 0.974782490192 | 1.502371953041 |
| C | -3.055211845608 | 1.477751270285 | -0.714496383378 |
| C | -1.932034744182 | 2.046875680766 | -1.580188320218 |
| C | -1.892053400502 | 1.570512499889 | -3.077880875977 |
| H | -1.663008562351 | 2.423447562818 | -5.725024338139 |
| H | -4.071773031906 | -0.097956395234 | 1.399430434158 |
| H | -3.829963764228 | 0.889734414686 | -1.200892747818 |
| H | -1.969475510161 | 3.147557965911 | -1.563911612303 |
| H | -0.971445282508 | 1.797952386282 | -1.110745454323 |
| H | -2.794115410521 | 1.923838814396 | -3.587750422077 |
| H | -1.961574843596 | 0.473691451206 | -3.058873944049 |
| C | -3.342750292410 | 1.954547573303 | 0.723281904226 |
| C | -4.067362427650 | 3.328515568328 | 0.651593197977 |
| H | -3.435180989010 | 4.071788695226 | 0.159222978828 |
| H | -5.001807910799 | 3.240935744846 | 0.092196463399 |
| H | -4.302598361635 | 3.683629690915 | 1.657876109130 |
| C | -2.016283801581 | 2.136761935776 | 1.510371377949 |
| H | -1.381404816353 | 2.897174001433 | 1.046765626781 |
| H | -2.234614926280 | 2.449513517138 | 2.533622228409 |
| H | -1.456352091679 | 1.198456852340 | 1.547369197921 |
| O | 0.413263024320 | 2.500929094734 | -6.018961535350 |

| | | | |
|---|---|---|---|
| O | -4.668237872215 | 1.396950874031 | 2.753610966037 |
| C | 0.696031662562 | 1.694341734623 | -3.261453539579 |
| H | 0.896495217952 | 2.450453175201 | -2.494257965509 |
| H | 0.721269266921 | 0.719867191996 | -2.759696594715 |
| H | 1.506214628804 | 1.731601280430 | -3.989478033694 |
| C | 0.217253994706 | 2.557653611331 | -7.454607887518 |
| H | -0.499953090095 | 3.347804197691 | -7.704822920914 |
| H | -0.177037087226 | 1.602468440029 | -7.818013463375 |
| C | 1.559660173488 | 2.852333818363 | -8.178250472599 |
| H | 1.962327542227 | 3.810891991645 | -7.822973036944 |
| H | 2.285457842481 | 2.064687081154 | -7.933225551550 |
| C | -5.390019219079 | 0.439315255990 | 3.572473473019 |
| H | -4.748449762554 | -0.425208318575 | 3.776477879086 |
| H | -6.278126108542 | 0.094263898631 | 3.033755811531 |
| C | -5.829822549152 | 1.071627520882 | 4.921012942812 |
| H | -4.943936999080 | 1.423504251352 | 5.467660232095 |
| H | -6.477248582616 | 1.937339500135 | 4.724863380030 |
| O | 1.301468078136 | 2.892823897379 | -9.589983246230 |
| C | 2.418416182588 | 3.145968890755 | -10.485235659242 |
| H | 2.871809363730 | 4.125445486031 | -10.288201678108 |
| H | 3.185816522006 | 2.367258390816 | -10.393230174249 |
| H | 1.999790466680 | 3.131291241190 | -11.490440251914 |
| O | -6.528949377217 | 0.063952363457 | 5.667458512372 |
| C | -7.055046101340 | 0.416794935435 | 6.975933425644 |
| H | -6.251432517338 | 0.717004001635 | 7.659883913637 |
| H | -7.789985102197 | 1.228106390739 | 6.903152276792 |

| | | | |
|---|---|---|---|
| H | -7.538822152187 | -0.483655178261 | 7.351399742608 |

Cam_TS3C-F1000

($E_F$ = -929.22296996 a.u.; $G_F$ = -928.84506730 a.u.)

0 1

| | | | |
|---|---|---|---|
| C | -0.826513967476 | 1.607914831308 | -3.924447132299 |
| C | -0.623396867060 | 2.441231006145 | -4.957149127608 |
| C | -3.630313450028 | 1.263766176572 | 1.668527435184 |
| C | -3.104720509710 | 1.627797023258 | -0.694115398947 |
| C | -1.823276321061 | 1.676903801060 | -1.489137727042 |
| C | -2.058777290647 | 1.730812197636 | -3.033394927852 |
| H | -1.294826661560 | 3.272527727042 | -5.165421372722 |
| H | -2.801410282559 | 0.625566026612 | 1.963145212860 |
| H | -3.892243072676 | 0.979878742837 | -1.071880219439 |
| H | -1.207906085415 | 2.530962138684 | -1.192435694666 |
| H | -1.214815593993 | 0.784577490041 | -1.271753652484 |
| H | -2.607232377234 | 2.647325960131 | -3.272731344069 |
| H | -2.729754186882 | 0.896855076082 | -3.284659090503 |
| C | -3.432142014689 | 2.333473835944 | 0.618954878504 |
| C | -4.740292413757 | 3.138846892795 | 0.427116488639 |
| H | -4.590910990724 | 3.906450212593 | -0.335523082043 |
| H | -5.556152696845 | 2.486146913736 | 0.107896258798 |
| H | -5.040597610644 | 3.619562294789 | 1.360829741065 |
| C | -2.311718243861 | 3.298833341396 | 1.067132516903 |
| H | -2.142342691957 | 4.078092941682 | 0.316413486139 |
| H | -2.593887086836 | 3.782345964068 | 2.005604191352 |

| | | | |
|---|---|---|---|
| H | -1.368538230154 | 2.769485917513 | 1.226253681992 |
| O | 0.452878122603 | 2.329748234493 | -5.819463561534 |
| O | -4.639690067243 | 1.430954121455 | 2.587279566198 |
| C | 0.096826921660 | 0.444927189259 | -3.668013735741 |
| H | 0.693235848609 | 0.599301749227 | -2.761961369283 |
| H | -0.476626343433 | -0.476831509353 | -3.516437965061 |
| H | 0.784936255208 | 0.296252881768 | -4.500279156915 |
| C | 0.291816683539 | 2.935411948525 | -7.112508500425 |
| H | 0.082839415528 | 4.007315045744 | -7.009648242399 |
| H | -0.540930278724 | 2.470471881747 | -7.652327727993 |
| C | 1.583813366391 | 2.747706533384 | -7.917405026944 |
| H | 2.424327289885 | 3.208636400865 | -7.379009929603 |
| H | 1.797427299083 | 1.674784023093 | -8.026460208826 |
| C | -4.739770341068 | 0.462761898425 | 3.644255637791 |
| H | -3.770823195414 | 0.348102681015 | 4.144051557573 |
| H | -5.039496106436 | -0.509135985878 | 3.237583660105 |
| C | -5.780382109157 | 0.948943051976 | 4.661581259051 |
| H | -5.481422060524 | 1.935012596942 | 5.045609079150 |
| H | -6.756893530575 | 1.058733530929 | 4.168542900364 |
| O | 1.384952147494 | 3.363365700802 | -9.186732156871 |
| C | 2.487444517390 | 3.273015361351 | -10.098033475828 |
| H | 3.383744644797 | 3.761796658701 | -9.692803425854 |
| H | 2.728898230079 | 2.227436888382 | -10.332434069325 |
| H | 2.178854812632 | 3.784670765876 | -11.009318000866 |
| O | -5.838134979394 | -0.014422303714 | 5.708619938976 |
| C | -6.756792942671 | 0.281875246147 | 6.768720905637 |

| | | | |
|---|---|---|---|
| H | -6.497791755159 | 1.223542347433 | 7.271293686445 |
| H | -7.788309363934 | 0.349464050417 | 6.397686608609 |
| H | -6.682952200742 | -0.539316899465 | 7.481143127249 |

Cam_Int2C-F1000

($E_F$ = -929.28407320 a.u.; $G_F$ = -928.90116516 a.u.)

0 1

| | | | |
|---|---|---|---|
| C | -1.186860304741 | 1.513413832766 | -3.675448563883 |
| C | -0.600298790794 | 2.426247589478 | -4.465440880128 |
| C | -3.158197869160 | 1.463429566231 | 1.232801854569 |
| C | -3.226229002703 | 1.527683773154 | -0.277739043084 |
| C | -2.035502273317 | 1.257392566594 | -1.201402096476 |
| C | -2.196384185727 | 1.913764490807 | -2.602812459409 |
| H | -0.771542190103 | 3.493869922837 | -4.337499706905 |
| H | -2.174200642635 | 1.315098573656 | 1.681397545830 |
| H | -4.167139820499 | 1.149546380243 | -0.672290095220 |
| H | -1.103469977891 | 1.602099904015 | -0.744142093851 |
| H | -1.935495197704 | 0.173256845761 | -1.317455409830 |
| H | -2.210987699236 | 3.002255337861 | -2.486345149241 |
| H | -3.192837747791 | 1.633472518166 | -2.974831076428 |
| C | -3.400253089125 | 2.798504997341 | 0.553605128012 |
| C | -4.797505349928 | 3.386162438088 | 0.617547238285 |
| H | -5.007484663293 | 3.975835751753 | -0.280658750990 |
| H | -5.551883832239 | 2.601976977557 | 0.699359564219 |
| H | -4.903392850361 | 4.047379782594 | 1.484129725880 |
| C | -2.290594871968 | 3.832342959250 | 0.590492369256 |

| | | | |
|---|---|---|---|
| H | -2.341083661593 | 4.491328635632 | -0.282945672816 |
| H | -2.383237381518 | 4.456182154506 | 1.485395216430 |
| H | -1.298120806082 | 3.377628381513 | 0.606175959465 |
| O | 0.279355779203 | 2.093117094505 | -5.480435424089 |
| O | -4.196087415984 | 0.809002384551 | 1.936098814202 |
| C | -0.960318135339 | 0.038845133170 | -3.889861414100 |
| H | -0.360260613248 | -0.396081294960 | -3.082964508201 |
| H | -1.914592529689 | -0.500395633942 | -3.900640596221 |
| H | -0.441725464971 | -0.147744662472 | -4.830239271694 |
| C | 0.421076723333 | 3.056369486843 | -6.537014071877 |
| H | 0.759475429215 | 4.019276016161 | -6.134802037833 |
| H | -0.537255195024 | 3.211085004775 | -7.045704796738 |
| C | 1.452649269203 | 2.540076308198 | -7.547843873797 |
| H | 2.415932407265 | 2.379744186620 | -7.042666509147 |
| H | 1.117922899182 | 1.575338871579 | -7.955283440585 |
| C | -4.305898886411 | 1.238622776581 | 3.301174179440 |
| H | -4.544010479351 | 2.307786399449 | 3.350497706712 |
| H | -3.356933298104 | 1.077720116398 | 3.829972272503 |
| C | -5.408288242501 | 0.439742723552 | 4.007163573929 |
| H | -6.370749439718 | 0.605973260796 | 3.501763288225 |
| H | -5.177872059028 | -0.634012945747 | 3.953248835527 |
| O | 1.566379568505 | 3.520044997808 | -8.575617602770 |
| C | 2.478864672918 | 3.203068194422 | -9.634508170026 |
| H | 3.503001124356 | 3.078935445887 | -9.257522318700 |
| H | 2.180360348433 | 2.285547319148 | -10.159373131665 |
| H | 2.449966647944 | 4.041630099009 | -10.329790562497 |

| | | | |
|---|---|---|---|
| O | -5.456074126142 | 0.888227843626 | 5.360088813726 |
| C | -6.430596194116 | 0.248447156971 | 6.192279563203 |
| H | -7.449558867509 | 0.409981431767 | 5.814764400740 |
| H | -6.247995412797 | -0.832288801027 | 6.266414905552 |
| H | -6.337818301593 | 0.697734713306 | 7.180829393765 |

Cam_Int3C-F1000

($E_F$ = -929.22614616 a.u.; $G_F$ = -928.84969605 a.u.)

0 1

| | | | |
|---|---|---|---|
| C | -0.620991525431 | 1.836621172204 | -3.855236626108 |
| C | -0.732554136045 | 2.270059033254 | -5.120686752707 |
| C | -4.174312502848 | 1.026057297983 | 1.434407368253 |
| C | -3.012642908026 | 1.521230680587 | -0.720680923525 |
| C | -1.889720255202 | 2.056860707517 | -1.574464896150 |
| C | -1.845761030112 | 1.481099941085 | -3.017320950173 |
| H | -1.700709278763 | 2.440455526781 | -5.588550651894 |
| H | -3.990234489577 | -0.042570390455 | 1.319880940713 |
| H | -3.795925527836 | 0.942608000695 | -1.204718585052 |
| H | -1.942651490055 | 3.156638439978 | -1.632747209120 |
| H | -0.931886568849 | 1.851963173780 | -1.078698520263 |
| H | -2.759948669845 | 1.770979739080 | -3.545793779552 |
| H | -1.876009623860 | 0.385650452114 | -2.927486838799 |
| C | -3.299846033667 | 2.022738030546 | 0.688734619305 |
| C | -4.046963705952 | 3.384521169951 | 0.601022789170 |
| H | -3.424130477141 | 4.131836390901 | 0.102754288178 |
| H | -4.977852189372 | 3.276209563698 | 0.039336949898 |

| | | | |
|---|---|---|---|
| H | -4.290849003690 | 3.746346599742 | 1.602848765884 |
| C | -1.983817943279 | 2.233497288544 | 1.486584925229 |
| H | -1.361048517723 | 3.006605168856 | 1.027732937076 |
| H | -2.218358251920 | 2.542975111913 | 2.507111378983 |
| H | -1.404891775433 | 1.307045167115 | 1.530308401824 |
| O | 0.361338968536 | 2.556953107839 | -5.917443586139 |
| O | -4.612336015249 | 1.431073685504 | 2.675484448828 |
| C | 0.730636788080 | 1.580799173332 | -3.238723108247 |
| H | 0.971690816052 | 2.329272518991 | -2.475682251349 |
| H | 0.749492163364 | 0.603913524280 | -2.742025786460 |
| H | 1.518586033573 | 1.606595389743 | -3.991451652022 |
| C | 0.138799937545 | 2.533198271428 | -7.336638282908 |
| H | -0.645814612596 | 3.248041553439 | -7.613503439957 |
| H | -0.173664578457 | 1.533481952259 | -7.659183612848 |
| C | 1.442600503408 | 2.911382952234 | -8.050623231366 |
| H | 1.763114754600 | 3.911980293619 | -7.726727954052 |
| H | 2.232248605457 | 2.196741995022 | -7.777612888720 |
| C | -5.317272939120 | 0.452597675639 | 3.458125053516 |
| H | -4.668354255770 | -0.409211174947 | 3.653719687705 |
| H | -6.201920509207 | 0.105391774056 | 2.913958818188 |
| C | -5.748295628521 | 1.079323070026 | 4.789919477386 |
| H | -4.862919769837 | 1.435963497485 | 5.335847936582 |
| H | -6.400376479820 | 1.943402812748 | 4.597924492538 |
| O | 1.184386831883 | 2.880954862263 | -9.451251686708 |
| C | 2.302320314260 | 3.191076949782 | -10.292906734144 |
| H | 2.676830436794 | 4.206248366995 | -10.104235133837 |

| | | | |
|---|---|---|---|
| H | 3.124006263629 | 2.476978704480 | -10.146891535072 |
| H | 1.944813493290 | 3.124036355070 | -11.320105455901 |
| O | -6.434135690722 | 0.070255077987 | 5.525225538823 |
| C | -6.930071610426 | 0.468089864621 | 6.809841145683 |
| H | -6.114481813782 | 0.784689057252 | 7.473882181677 |
| H | -7.655544254584 | 1.288092589744 | 6.722764022042 |
| H | -7.422889078885 | -0.405540378520 | 7.235570039407 |

cisCydiol_TS1-F4000

($E_F$ = -774.03158332 a.u.; $G_F$ = -773.73146081 a.u.)

0 1

| | | | |
|---|---|---|---|
| C | -4.490401945921 | -1.746770169603 | -2.885138039478 |
| C | -3.209892617966 | -0.919130794918 | -1.231525430977 |
| C | -4.115581589692 | -1.196906658452 | -0.048374299388 |
| C | -4.385742188383 | -2.699649759710 | 0.144710971906 |
| C | -5.059210744743 | -3.392472265499 | -1.047008720001 |
| C | -4.328109888785 | -3.170822186615 | -2.379785929979 |
| H | -3.882198828487 | -1.479210156934 | -3.749854828900 |
| H | -2.511811783173 | -1.724996770397 | -1.459230341166 |
| H | -3.631037374016 | -0.805615691475 | 0.855961653802 |
| H | -5.052840610953 | -0.642853904500 | -0.159771657723 |
| H | -5.002814004139 | -2.838881638038 | 1.038767729222 |
| H | -3.428110701237 | -3.195149989546 | 0.350276850148 |
| H | -5.127899989004 | -4.465932831152 | -0.842962065922 |
| H | -6.082652324777 | -3.023563089232 | -1.159213777946 |
| H | -4.747629709753 | -3.846748065106 | -3.137528478538 |

| | | | |
|---|---|---|---|
| H | -3.271229485512 | -3.440431155530 | -2.293820606122 |
| O | -5.905917928476 | -1.519083633062 | -3.146311077971 |
| O | -2.442029723375 | 0.302040031809 | -1.040265536155 |
| C | -6.462716026173 | -2.051493710798 | -4.439094756978 |
| H | -5.842668229500 | -2.881141764056 | -4.786785367616 |
| H | -6.408773174382 | -1.245917128679 | -5.175438242396 |
| C | -7.981090176116 | -2.574123725721 | -4.395064362546 |
| H | -8.046223747105 | -3.412366464176 | -3.690443191623 |
| H | -8.623988116449 | -1.763390022046 | -4.032185966628 |
| C | -1.133600134113 | 0.104600808222 | -0.325158625421 |
| H | -0.425857447717 | -0.348312257304 | -1.025862080597 |
| H | -1.289958531524 | -0.587652868023 | 0.507197569959 |
| C | -0.454757119453 | 1.422139135364 | 0.286787935793 |
| H | -0.253921970186 | 2.132390235745 | -0.523948993101 |
| H | -1.157697299900 | 1.884961373515 | 0.989760811644 |
| O | -8.395696836520 | -2.998890374482 | -5.736745152150 |
| C | -9.769559114695 | -3.541490859221 | -6.062948588060 |
| H | -9.963354251723 | -4.463411343255 | -5.506677214227 |
| H | -10.541290843591 | -2.797245127379 | -5.845436164707 |
| H | -9.718388219650 | -3.740723697152 | -7.130579388633 |
| O | 0.791090508570 | 1.055294533413 | 0.969736796180 |
| C | 1.695501901589 | 2.048520946697 | 1.665405544477 |
| H | 2.074786144669 | 2.789613602387 | 0.955687551489 |
| H | 1.167676932796 | 2.543534099022 | 2.485864002844 |
| H | 2.505845246810 | 1.431906086695 | 2.046568739175 |

cisCydiol-F4000

($E_F$ = -774.04373482 a.u.; $G_F$ = -773.73988006 a.u.)

0 1

| | | | |
|---|---|---|---|
| C | -4.182446739614 | -1.695069230042 | -2.691073956405 |
| C | -3.400981053448 | -1.123781998859 | -1.349107637029 |
| C | -4.368942663389 | -1.261237061921 | -0.174064122450 |
| C | -4.637509341307 | -2.757808663633 | 0.083752568451 |
| C | -5.096432138456 | -3.510171642433 | -1.177088331655 |
| C | -4.224216451589 | -3.216391470763 | -2.412241504981 |
| H | -3.584628034908 | -1.453779571559 | -3.575416491264 |
| H | -2.636644305832 | -1.901761853944 | -1.256591505749 |
| H | -3.938936887156 | -0.801877141602 | 0.719484321092 |
| H | -5.295959696782 | -0.729843241741 | -0.406460425582 |
| H | -5.383433304223 | -2.875907937826 | 0.875755648431 |
| H | -3.710237859037 | -3.210620783654 | 0.457297920508 |
| H | -5.106919067104 | -4.587268263446 | -0.983328761075 |
| H | -6.122327581817 | -3.216517324211 | -1.411796756082 |
| H | -4.640246604925 | -3.729947143099 | -3.283313620693 |
| H | -3.211758174490 | -3.610635055593 | -2.279582638106 |
| O | -5.599343478699 | -1.308631534565 | -2.953307474847 |
| O | -2.602832860927 | 0.140425174741 | -1.279079243607 |
| C | -6.225549693284 | -1.769279926204 | -4.226008128740 |
| H | -5.527539546162 | -2.400985679566 | -4.781197559166 |
| H | -6.409762418235 | -0.870185871240 | -4.818602963592 |
| C | -7.630800214006 | -2.554550924983 | -4.139635491896 |
| H | -7.478069901383 | -3.519446530353 | -3.639899366033 |

| | | | |
|---|---|---|---|
| H | -8.326916919753 | -1.957800128006 | -3.537774457262 |
| C | -1.418316990010 | 0.003516488466 | -0.380266223360 |
| H | -0.708045915657 | -0.688157090205 | -0.847272262126 |
| H | -1.736691132376 | -0.431268401060 | 0.572696594402 |
| C | -0.610993510510 | 1.339039610977 | -0.016784750113 |
| H | -0.232607775836 | 1.796689128181 | -0.938543133604 |
| H | -1.292224347681 | 2.047130042831 | 0.470098653117 |
| O | -8.164467344523 | -2.760349840631 | -5.491141935267 |
| C | -9.480268755919 | -3.444378672255 | -5.786363231804 |
| H | -9.467300147381 | -4.479080620770 | -5.430838940806 |
| H | -10.311524598021 | -2.897407098069 | -5.331563030675 |
| H | -9.547607947175 | -3.412264970486 | -6.871113561703 |
| O | 0.500840294233 | 0.995440243667 | 0.879687798236 |
| C | 1.485745163744 | 2.000786282641 | 1.431166830208 |
| H | 2.036857665876 | 2.488203026136 | 0.621356014736 |
| H | 0.974182527301 | 2.748051025827 | 2.045131565536 |
| H | 2.153738604732 | 1.396301382353 | 2.040019616867 |

cisCydiol_Int1-F4000

($E_F$ = -774.43982816 a.u.; $G_F$ = -774.14960896 a.u.)

0 1

| | | | |
|---|---|---|---|
| C | -6.805104652048 | -2.262905237891 | -3.786537041002 |
| C | -1.392835819100 | -0.377795358526 | 0.079202271071 |
| C | -2.677584757406 | -0.145246763812 | -0.786041657611 |
| C | -3.483819203117 | -1.438864299196 | -1.338455078980 |
| C | -4.782861737045 | -1.200441315834 | -2.273288033965 |

| | | | |
|---|---|---|---|
| C | -5.575435799929 | -2.494361964844 | -2.844407369968 |
| H | -6.773240586789 | -1.438869779659 | -4.498530685356 |
| H | -0.735055472417 | -1.209479695351 | -0.171026677881 |
| H | -3.370205097391 | 0.471152174144 | -0.201836242959 |
| H | -2.404523253399 | 0.467469065170 | -1.659605558302 |
| H | -3.791661571476 | -2.034325962211 | -0.473245661504 |
| H | -2.775626320617 | -2.056616922839 | -1.901287084899 |
| H | -5.498876667885 | -0.591588907365 | -1.712426274784 |
| H | -4.470627243432 | -0.596061421167 | -3.131870412773 |
| H | -5.911717930947 | -3.097747537914 | -1.993634595758 |
| H | -4.840713594812 | -3.119667173881 | -3.375654663561 |
| O | -7.604044053867 | -3.360153284069 | -4.192829957173 |
| O | -0.743358640320 | 0.720876651783 | 0.694296304234 |
| C | -8.695023786857 | -3.122666194556 | -5.185653861681 |
| H | -8.246204263994 | -2.729145517979 | -6.102518423253 |
| H | -9.374468030302 | -2.371160877976 | -4.775567743401 |
| C | -9.556467449183 | -4.419327847786 | -5.569081431798 |
| H | -8.883011014664 | -5.180484428625 | -5.981284644735 |
| H | -10.015281134664 | -4.818446438363 | -4.656519663561 |
| C | 0.546238326824 | 0.481574591106 | 1.409833452393 |
| H | 1.267892025182 | 0.078708417295 | 0.693013447894 |
| H | 0.372195928389 | -0.263146251629 | 2.190610010156 |
| C | 1.193486777161 | 1.779836690798 | 2.092476489390 |
| H | 1.370448784053 | 2.534637376879 | 1.316734883550 |
| H | 0.479643691210 | 2.187798930400 | 2.818134846261 |
| O | -10.586355767778 | -4.059544690840 | -6.547720856217 |

| | | | |
|---|---|---|---|
| C | -11.580324400818 | -5.046205878567 | -7.119700557403 |
| H | -11.064991212332 | -5.840842087390 | -7.667184384992 |
| H | -12.202060903244 | -5.472308655738 | -6.326862700738 |
| H | -12.180507012634 | -4.441435675648 | -7.795083920188 |
| O | 2.450167466950 | 1.418333902570 | 2.754178058708 |
| C | 3.317087107292 | 2.405985611860 | 3.503634140433 |
| H | 3.670044260261 | 3.196221145777 | 2.834491182511 |
| H | 2.770446688927 | 2.837930457508 | 4.347032213968 |
| H | 4.148898273643 | 1.800293755404 | 3.854908597303 |

cisCydiol_TS1-F3500

($E_F$ = -773.86050841 a.u.; $G_F$ = -773.56061157 a.u.)

0 1

| | | | |
|---|---|---|---|
| C | -4.534959298223 | -1.781021047460 | -2.912525466766 |
| C | -3.174723179298 | -0.904115445503 | -1.193874457684 |
| C | -4.087037907872 | -1.194472864351 | -0.021545837301 |
| C | -4.374428354499 | -2.695265130388 | 0.158290253772 |
| C | -5.080236518823 | -3.374382545619 | -1.022190169091 |
| C | -4.359498479094 | -3.183082776012 | -2.365103828961 |
| H | -3.911230884546 | -1.513034532923 | -3.765650848678 |
| H | -2.492441263031 | -1.711959799755 | -1.458927639007 |
| H | -3.597238317370 | -0.819647012123 | 0.888433519883 |
| H | -5.018015415941 | -0.627502358016 | -0.121463153150 |
| H | -4.976776520873 | -2.832787710624 | 1.062762069020 |
| H | -3.420731189697 | -3.206323005573 | 0.343288047606 |
| H | -5.174468576868 | -4.444207528179 | -0.808825232004 |

| | | | |
|---|---|---|---|
| H | -6.095772889731 | -2.980789678557 | -1.124333481175 |
| H | -4.778455832042 | -3.884827552594 | -3.101462041342 |
| H | -3.299692481596 | -3.440685723497 | -2.278224871661 |
| O | -5.933228742010 | -1.552913895629 | -3.175491804056 |
| O | -2.424286636175 | 0.306753376407 | -0.998997001980 |
| C | -6.470362315581 | -2.066438478275 | -4.471419393502 |
| H | -5.855915533150 | -2.902271385882 | -4.815644231240 |
| H | -6.402560129669 | -1.260659410045 | -5.207154955082 |
| C | -7.979272844508 | -2.569253622136 | -4.424953469682 |
| H | -8.056594727349 | -3.401102030195 | -3.713135401874 |
| H | -8.619963602557 | -1.751188532484 | -4.073028371155 |
| C | -1.097070308519 | 0.109677118272 | -0.342995108713 |
| H | -0.396957688376 | -0.285176029569 | -1.085661145081 |
| H | -1.205046434295 | -0.626999060463 | 0.458693676205 |
| C | -0.459821865361 | 1.412923293141 | 0.305055832044 |
| H | -0.303091641564 | 2.168748736929 | -0.474383032646 |
| H | -1.159037969880 | 1.818775392313 | 1.046802010097 |
| O | -8.377511306332 | -2.997789322276 | -5.760929473255 |
| C | -9.742997640709 | -3.525895028950 | -6.061883215253 |
| H | -9.944516573344 | -4.436656801544 | -5.488411067935 |
| H | -10.508634867684 | -2.772656541663 | -5.849257586557 |
| H | -9.714673881579 | -3.747077586642 | -7.126542986177 |
| O | 0.805788066914 | 1.049788735132 | 0.932791847707 |
| C | 1.665640349165 | 2.044599424867 | 1.643684942724 |
| H | 1.995282848558 | 2.834389745773 | 0.960894443074 |
| H | 1.133671659171 | 2.482952869645 | 2.494312014308 |

| | | | |
|---|---|---|---|
| H | 2.517116951580 | 1.462196495248 | 1.988573886254 |

cisCydiol-F3500

($E_F$ = -773.88178717 a.u.; $G_F$ = -773.57666459 a.u.)

0 1

| | | | |
|---|---|---|---|
| C | -4.162029960583 | -1.718384822953 | -2.672450283504 |
| C | -3.406191258280 | -1.162064758653 | -1.342032110271 |
| C | -4.382585008016 | -1.282390719317 | -0.171040736939 |
| C | -4.677224223467 | -2.773766696979 | 0.086222165440 |
| C | -5.140458830125 | -3.516409626907 | -1.178684178091 |
| C | -4.248713516843 | -3.240189353691 | -2.403768770703 |
| H | -3.546165375787 | -1.492287250288 | -3.549383384539 |
| H | -2.642323775129 | -1.939104897258 | -1.227517047873 |
| H | -3.949382562236 | -0.828701258367 | 0.723873612832 |
| H | -5.298768676572 | -0.735790663613 | -0.410249761114 |
| H | -5.430087248461 | -2.879548140626 | 0.873321110268 |
| H | -3.760281765110 | -3.242838746831 | 0.465260614442 |
| H | -5.176816532345 | -4.593096390337 | -0.985932748162 |
| H | -6.157673289325 | -3.200145046009 | -1.421887507736 |
| H | -4.668352457654 | -3.737542180300 | -3.282613532486 |
| H | -3.248084450682 | -3.660748018650 | -2.261766798648 |
| O | -5.550248301204 | -1.279598478742 | -2.937482757967 |
| O | -2.624346475125 | 0.100076944631 | -1.299592666320 |
| C | -6.177130615612 | -1.713958876474 | -4.204869183574 |
| H | -5.469634010911 | -2.301799151570 | -4.796254280307 |
| H | -6.403273584840 | -0.802754408729 | -4.763936835239 |

| | | | |
|---|---|---|---|
| C | -7.542159645111 | -2.539254536717 | -4.111435383605 |
| H | -7.352521748059 | -3.519435568094 | -3.654790836648 |
| H | -8.243375624497 | -1.989189272229 | -3.471053305534 |
| C | -1.446419098163 | -0.022842279753 | -0.409494287362 |
| H | -0.730900549673 | -0.715609328992 | -0.868240055700 |
| H | -1.756252316926 | -0.448083889442 | 0.551125095845 |
| C | -0.660340079890 | 1.314392499595 | -0.071892386060 |
| H | -0.283543821794 | 1.765731992009 | -0.998071564938 |
| H | -1.343231311730 | 2.025930759391 | 0.408843811456 |
| O | -8.089235831754 | -2.701455015450 | -5.454180920982 |
| C | -9.377131525278 | -3.410435594337 | -5.720281800654 |
| H | -9.321495616104 | -4.455995748752 | -5.399508857964 |
| H | -10.210329167125 | -2.908953602088 | -5.216987500641 |
| H | -9.496489218500 | -3.351712786639 | -6.799935718642 |
| O | 0.444399661252 | 0.975907274023 | 0.821796159791 |
| C | 1.403179378069 | 1.993941682604 | 1.346010243305 |
| H | 1.946525994733 | 2.477976769506 | 0.527751211877 |
| H | 0.883980498258 | 2.747708554172 | 1.947109192069 |
| H | 2.087591521253 | 1.420423902828 | 1.967342900837 |

cisCydiol_Int1-F3500

($E_F$ = -774.21441073 a.u.; $G_F$ = -773.92265363 a.u.)

0 1

| | | | |
|---|---|---|---|
| C | -6.773931766614 | -2.248672740813 | -3.757986447562 |
| C | -1.428002584659 | -0.403200154277 | 0.053550541480 |
| C | -2.706153498299 | -0.160486270930 | -0.794541969371 |

| | | | |
|---|---|---|---|
| C | -3.489003056044 | -1.449450421185 | -1.346441432215 |
| C | -4.780859276285 | -1.202693263771 | -2.262510245266 |
| C | -5.550051933124 | -2.491612456699 | -2.833387966022 |
| H | -6.750306877241 | -1.415032548514 | -4.459281650004 |
| H | -0.767222935248 | -1.228706353211 | -0.209593665008 |
| H | -3.400437020269 | 0.444213566938 | -0.199444549686 |
| H | -2.442632347084 | 0.465222292803 | -1.662616857472 |
| H | -3.787264159969 | -2.057676531400 | -0.486313428237 |
| H | -2.778348680330 | -2.055522778652 | -1.919584225887 |
| H | -5.499412524882 | -0.605505379400 | -1.691739306248 |
| H | -4.477834140409 | -0.585629485220 | -3.115881886434 |
| H | -5.878367581024 | -3.107700198305 | -1.988126107354 |
| H | -4.812541954242 | -3.106033075017 | -3.374708209088 |
| O | -7.558719979124 | -3.344171442166 | -4.169966174470 |
| O | -0.788964948980 | 0.690349792425 | 0.671142633756 |
| C | -8.647570297585 | -3.094267039395 | -5.144065732257 |
| H | -8.211081746484 | -2.681137316592 | -6.058957065646 |
| H | -9.333542183296 | -2.356119472022 | -4.719442713598 |
| C | -9.483461455797 | -4.388182522404 | -5.531828035920 |
| H | -8.805011865620 | -5.137161306343 | -5.959474312523 |
| H | -9.933369475237 | -4.807442699518 | -4.623172321131 |
| C | 0.492342309326 | 0.442995199814 | 1.373963034821 |
| H | 1.215759460281 | 0.046598006060 | 0.654423352091 |
| H | 0.324970101067 | -0.307466923765 | 2.151424573391 |
| C | 1.122030803620 | 1.733739149561 | 2.052731047633 |
| H | 1.294340802511 | 2.495232039872 | 1.281643189352 |

| | | | |
|---|---|---|---|
| H | 0.409056173549 | 2.136061725466 | 2.783269612692 |
| O | -10.514069756646 | -4.018415756831 | -6.493248663302 |
| C | -11.478951037820 | -5.013604839956 | -7.052589312968 |
| H | -10.953415192019 | -5.797454327430 | -7.607715642978 |
| H | -12.084382624541 | -5.459825227919 | -6.256943560162 |
| H | -12.105550185238 | -4.429740448973 | -7.723155525565 |
| O | 2.373583059262 | 1.366877738790 | 2.702485752521 |
| C | 3.211135679996 | 2.360213659406 | 3.441477714486 |
| H | 3.550716238209 | 3.158315275022 | 2.773269873919 |
| H | 2.657611913243 | 2.787238882560 | 4.284062433391 |
| H | 4.058620693617 | 1.780185023064 | 3.800017654830 |

cisCydiol_TS1-F3000

($E_F$ = -773.69059800 a.u.; $G_F$ = -773.39025005 a.u.)

0 1

| | | | |
|---|---|---|---|
| C | -4.543337403197 | -1.808373181465 | -2.920160407861 |
| C | -3.160995696064 | -0.910555091997 | -1.168037696438 |
| C | -4.090701648274 | -1.199133093394 | -0.010448961850 |
| C | -4.386247552206 | -2.698319028827 | 0.167820031327 |
| C | -5.097935891617 | -3.375528613163 | -1.010324388199 |
| C | -4.374410568259 | -3.201843920422 | -2.354332773170 |
| H | -3.905499679761 | -1.540216964043 | -3.762794772312 |
| H | -2.484752165736 | -1.721671736792 | -1.438794598043 |
| H | -3.610784642309 | -0.825129376470 | 0.905681669029 |
| H | -5.018677831911 | -0.628869264453 | -0.121492286684 |
| H | -4.988979673214 | -2.832039762137 | 1.072631365894 |

| | | | |
|---|---|---|---|
| H | -3.435730891112 | -3.215294782050 | 0.352894670004 |
| H | -5.204466297333 | -4.442862717512 | -0.790162963323 |
| H | -6.109265892275 | -2.971593715346 | -1.115290826643 |
| H | -4.795116376875 | -3.911570757706 | -3.083011293551 |
| H | -3.315354527621 | -3.460992326014 | -2.262570886459 |
| O | -5.926820863331 | -1.562463100110 | -3.182917677775 |
| O | -2.423656581982 | 0.296096873686 | -0.979557588480 |
| C | -6.452114736534 | -2.046680105086 | -4.482353842661 |
| H | -5.833399599628 | -2.871549486489 | -4.846124996471 |
| H | -6.393735638088 | -1.226867494599 | -5.203920091298 |
| C | -7.944448890449 | -2.556470991176 | -4.426876706168 |
| H | -8.011171461967 | -3.400260577606 | -3.727308698146 |
| H | -8.590979058136 | -1.749994146916 | -4.057607577183 |
| C | -1.090424907016 | 0.100743406126 | -0.361235805317 |
| H | -0.396920924962 | -0.268795854005 | -1.123544406475 |
| H | -1.169677518908 | -0.652356148193 | 0.428973924178 |
| C | -0.477640743042 | 1.396855594829 | 0.292702916728 |
| H | -0.343576102895 | 2.170652877812 | -0.474000351215 |
| H | -1.172701463539 | 1.778523128727 | 1.051773522124 |
| O | -8.338992122029 | -2.963404744262 | -5.762294664291 |
| C | -9.692972727794 | -3.493700191574 | -6.032851249949 |
| H | -9.876793503325 | -4.411972913861 | -5.463782983989 |
| H | -10.461750912105 | -2.750572871231 | -5.793289681424 |
| H | -9.698561769076 | -3.707212853379 | -7.099914438231 |
| O | 0.794594334468 | 1.037171488730 | 0.890748742112 |
| C | 1.618988660857 | 2.038467728099 | 1.601831730738 |

| | | | |
|---|---|---|---|
| H | 1.917293402340 | 2.851327523529 | 0.930307960339 |
| H | 1.081729891284 | 2.449552179084 | 2.463880162675 |
| H | 2.496238030862 | 1.487527760470 | 1.935323190149 |

cisCydiol-F3000

($E_F$ = -773.72245034 a.u.; $G_F$ = -773.41619818 a.u.)

0 1

| | | | |
|---|---|---|---|
| C | -4.143746032874 | -1.743949062318 | -2.652919729028 |
| C | -3.407992646647 | -1.203366370557 | -1.331019323918 |
| C | -4.389826902299 | -1.310196296199 | -0.162517429267 |
| C | -4.712185631066 | -2.796183675822 | 0.089960527778 |
| C | -5.185691843868 | -3.523327270757 | -1.179910299400 |
| C | -4.276490499865 | -3.264494403014 | -2.395612472490 |
| H | -3.509923369236 | -1.533484451387 | -3.521803626713 |
| H | -2.643566411672 | -1.978701313248 | -1.200363436656 |
| H | -3.950964578425 | -0.865725298789 | 0.734321990048 |
| H | -5.294831219128 | -0.746631949058 | -0.404261620413 |
| H | -5.469028785754 | -2.890628043299 | 0.874622340961 |
| H | -3.805506885186 | -3.284638058289 | 0.469306210447 |
| H | -5.251270939926 | -4.599387214712 | -0.991574413344 |
| H | -6.193065618521 | -3.180117034327 | -1.427818156919 |
| H | -4.699176683211 | -3.745063373812 | -3.282377357640 |
| H | -3.288519309807 | -3.711557062273 | -2.245416043971 |
| O | -5.501269506796 | -1.250278316817 | -2.920584741799 |
| O | -2.643863842899 | 0.058159881706 | -1.313601506113 |
| C | -6.129084069962 | -1.656841897367 | -4.184157530316 |

| | | | |
|---|---|---|---|
| H | -5.416212666067 | -2.206645338608 | -4.805915212263 |
| H | -6.387936447206 | -0.735199534601 | -4.711313461228 |
| C | -7.460418386066 | -2.511981678213 | -4.087730882956 |
| H | -7.243200274636 | -3.502970484354 | -3.667346719892 |
| H | -8.164017270525 | -2.000409494309 | -3.417737202447 |
| C | -1.468483908879 | -0.050115472667 | -0.435980682199 |
| H | -0.746568128181 | -0.738918879655 | -0.891968433392 |
| H | -1.765392406765 | -0.470636478383 | 0.531285054446 |
| C | -0.707574595200 | 1.291607820020 | -0.121001773226 |
| H | -0.338161079748 | 1.741469822215 | -1.051456875847 |
| H | -1.394310614574 | 2.001071217686 | 0.358443022624 |
| O | -8.018257319477 | -2.638177031644 | -5.421512667328 |
| C | -9.280568663764 | -3.370020043363 | -5.660175020511 |
| H | -9.187831939183 | -4.421765278172 | -5.366456516294 |
| H | -10.112365237610 | -2.907400849071 | -5.117120169573 |
| H | -9.446472153712 | -3.294798472347 | -6.733090887489 |
| O | 0.395921416563 | 0.961849814269 | 0.765401796374 |
| C | 1.325576633058 | 1.996781587930 | 1.263894378262 |
| H | 1.855447529325 | 2.482735603495 | 0.436870799233 |
| H | 0.795442678868 | 2.751217360580 | 1.856076868039 |
| H | 2.030265722761 | 1.455870634446 | 1.892535010711 |

cisCydiol_Int1-F3000

($E_F$ = -773.99178424 a.u.; $G_F$ = -773.69875552 a.u.)

0 1

| | | | |
|---|---|---|---|
| C | -6.745089983075 | -2.223100626335 | -3.733233529164 |

| | | | |
|---|---|---|---|
| C | -1.455505142809 | -0.419947305047 | 0.027125778714 |
| C | -2.731162441043 | -0.166697935626 | -0.799665166904 |
| C | -3.488946872727 | -1.450693606550 | -1.358790388209 |
| C | -4.778379633834 | -1.195176689086 | -2.251978438254 |
| C | -5.522344208404 | -2.478586334789 | -2.830694696888 |
| H | -6.734447297277 | -1.375605340894 | -4.418277355853 |
| H | -0.789407410167 | -1.234576259905 | -0.256327201667 |
| H | -3.427528415085 | 0.419490243696 | -0.188105852887 |
| H | -2.481428407831 | 0.478225610345 | -1.658394442464 |
| H | -3.773114173120 | -2.078098288793 | -0.507328433281 |
| H | -2.775346536321 | -2.038227772317 | -1.948017249880 |
| H | -5.500041885106 | -0.616764741412 | -1.665439763469 |
| H | -4.489187352834 | -0.558797471445 | -3.096362770593 |
| H | -5.837955939213 | -3.114143342700 | -1.994652298177 |
| H | -4.781533671265 | -3.075057022323 | -3.388453809276 |
| O | -7.513229406655 | -3.316968693065 | -4.156483451699 |
| O | -0.828882894252 | 0.666852665399 | 0.653092412083 |
| C | -8.604831281202 | -3.052983838063 | -5.107505555999 |
| H | -8.184905806011 | -2.612430956099 | -6.017969951188 |
| H | -9.298556616936 | -2.334541426764 | -4.661047838849 |
| C | -9.411952985252 | -4.345524763500 | -5.506995743920 |
| H | -8.726507773078 | -5.075536316001 | -5.957090406866 |
| H | -9.847458084279 | -4.792672089235 | -4.603998177091 |
| C | 0.447793879861 | 0.411929701937 | 1.339240454527 |
| H | 1.172189999161 | 0.029057097985 | 0.612604410608 |
| H | 0.291090731077 | -0.349385004853 | 2.108977858986 |

| | | | |
|---|---|---|---|
| C | 1.057939617014 | 1.694227609986 | 2.021214087364 |
| H | 1.220743722406 | 2.467092952484 | 1.258653899590 |
| H | 0.345696493241 | 2.083599635195 | 2.760224476430 |
| O | -10.449121998718 | -3.965646717756 | -6.445681728611 |
| C | -11.382459153188 | -4.971486738435 | -6.998107359389 |
| H | -10.843626397764 | -5.737783993868 | -7.566473604227 |
| H | -11.966976365715 | -5.444843028989 | -6.201370135427 |
| H | -12.038009720478 | -4.407486801192 | -7.658638516532 |
| O | 2.308031053393 | 1.324489262117 | 2.655012164421 |
| C | 3.114963519512 | 2.323818370142 | 3.388671888150 |
| H | 3.436167065757 | 3.134077784784 | 2.724678240369 |
| H | 2.553942226013 | 2.738528044186 | 4.233514821339 |
| H | 3.979793908993 | 1.770462024390 | 3.749267325990 |

cisCydiol_TS1-F2500

($E_F$ = -773.52204696 a.u.; $G_F$ = -773.22266781 a.u.)

0 1

| | | | |
|---|---|---|---|
| C | -4.548166773530 | -1.844892916212 | -2.924201546941 |
| C | -3.147142865307 | -0.918593950022 | -1.127866874161 |
| C | -4.107700471174 | -1.196003773656 | 0.005454755827 |
| C | -4.417689104170 | -2.691847228258 | 0.186856563469 |
| C | -5.129792109813 | -3.371486375113 | -0.989757970905 |
| C | -4.394717887500 | -3.226469554777 | -2.331081135809 |
| H | -3.891202192081 | -1.583949427573 | -3.754362642840 |
| H | -2.478377255899 | -1.736505070207 | -1.395663749492 |
| H | -3.646064896339 | -0.819240725003 | 0.930218439047 |

| | | | |
|---|---|---|---|
| H | -5.029778495763 | -0.620900370955 | -0.129546872783 |
| H | -5.027434410655 | -2.814778283162 | 1.088528984322 |
| H | -3.473564687484 | -3.217017476393 | 0.381563736959 |
| H | -5.254003199073 | -4.434277629391 | -0.756915564163 |
| H | -6.134541562725 | -2.954287451656 | -1.106693051591 |
| H | -4.814874294988 | -3.947894893067 | -3.049645384386 |
| H | -3.337786319115 | -3.489769402115 | -2.226000487177 |
| O | -5.915392443967 | -1.575650949631 | -3.195087015080 |
| O | -2.423591418357 | 0.282770387427 | -0.944476713529 |
| C | -6.426356336534 | -2.035983984657 | -4.497538477299 |
| H | -5.807187489750 | -2.856179098566 | -4.872028891069 |
| H | -6.368462271783 | -1.207366316657 | -5.209669854710 |
| C | -7.906907894374 | -2.542835188380 | -4.436551424827 |
| H | -7.971098240699 | -3.392781741083 | -3.743463885476 |
| H | -8.554785047988 | -1.740982826114 | -4.058191738657 |
| C | -1.059700068279 | 0.102232654424 | -0.419954814760 |
| H | -0.389599667537 | -0.167581598821 | -1.242812352630 |
| H | -1.057592937769 | -0.713083484854 | 0.310324869159 |
| C | -0.496546529220 | 1.372154735356 | 0.299346238796 |
| H | -0.440932240297 | 2.209833665134 | -0.408317800665 |
| H | -1.174767560633 | 1.654781462452 | 1.115512042566 |
| O | -8.297379752260 | -2.935854380004 | -5.769771467621 |
| C | -9.643030436877 | -3.463953284812 | -6.010015245798 |
| H | -9.815190450090 | -4.383810005109 | -5.438412767403 |
| H | -10.410533018162 | -2.724709432269 | -5.751666819581 |
| H | -9.680046658259 | -3.678543182612 | -7.076645448458 |

| | | | |
|---|---|---|---|
| O | 0.813021957609 | 1.037657849722 | 0.807450867630 |
| C | 1.584047224290 | 2.034242975936 | 1.555797182776 |
| H | 1.796456795664 | 2.915578546159 | 0.939100526443 |
| H | 1.054245109852 | 2.343501025762 | 2.464643591167 |
| H | 2.514413958279 | 1.535099449580 | 1.821109471344 |

cisCydiol_Int1-F2500

($E_F$ = -773.77171783 a.u.; $G_F$ = -773.47774159 a.u.)

0 1

| | | | |
|---|---|---|---|
| C | -6.723192484512 | -2.205985467453 | -3.701961026591 |
| C | -1.476065193777 | -0.443872221369 | -0.000461452633 |
| C | -2.755801321676 | -0.181364921054 | -0.797526821528 |
| C | -3.484489046019 | -1.459887895450 | -1.373282670980 |
| C | -4.779172592943 | -1.196711112265 | -2.234519134275 |
| C | -5.493320045438 | -2.473702662078 | -2.831547089566 |
| H | -6.736086476023 | -1.337837958862 | -4.360818112314 |
| H | -0.801064715695 | -1.239579215734 | -0.315357606267 |
| H | -3.454309635271 | 0.375449845875 | -0.161000543341 |
| H | -2.528261367709 | 0.491735986403 | -1.641263733658 |
| H | -3.745489911711 | -2.115931066905 | -0.535784931173 |
| H | -2.767904228374 | -2.017702744600 | -1.987771677266 |
| H | -5.504262674395 | -0.649071596811 | -1.622747967491 |
| H | -4.513164527440 | -0.530862383462 | -3.064046033346 |
| H | -5.786260069561 | -3.139404635083 | -2.010514972328 |
| H | -4.748959353922 | -3.039952849971 | -3.416352278902 |
| O | -7.469288307909 | -3.298922107911 | -4.144557746729 |

| | | | |
|---|---|---|---|
| O | -0.865378123271 | 0.634048905583 | 0.641509554810 |
| C | -8.573138492454 | -3.020054394408 | -5.061981883950 |
| H | -8.180130033550 | -2.537832851874 | -5.963857996258 |
| H | -9.276974085020 | -2.334194594870 | -4.580495105247 |
| C | -9.343243409155 | -4.313428648999 | -5.484537865473 |
| H | -8.647490459869 | -5.011227047486 | -5.969720566893 |
| H | -9.753005808161 | -4.802567755717 | -4.590737927873 |
| C | 0.412730153518 | 0.374323699096 | 1.302476153835 |
| H | 1.136423411866 | 0.017051628568 | 0.561543590740 |
| H | 0.275515291842 | -0.405682774571 | 2.057654661166 |
| C | 0.998523718268 | 1.646647310336 | 1.997053398254 |
| H | 1.141941951756 | 2.438294034931 | 1.249316547055 |
| H | 0.287782282455 | 2.010668730245 | 2.750978637537 |
| O | -10.397787866928 | -3.924589960933 | -6.388966665291 |
| C | -11.293430372708 | -4.944447166139 | -6.941776045097 |
| H | -10.737402192403 | -5.679623459393 | -7.535583433292 |
| H | -11.845435571620 | -5.459313438793 | -6.146545722815 |
| H | -11.986467702757 | -4.400277784791 | -7.580996548468 |
| O | 2.253637198174 | 1.279459196425 | 2.606239389058 |
| C | 3.026309018816 | 2.285330291097 | 3.340483907883 |
| H | 3.319091448825 | 3.114880152907 | 2.685979536678 |
| H | 2.458301451111 | 2.675598457000 | 4.193252319052 |
| H | 3.913033533029 | 1.762385043137 | 3.694452665622 |

cisCydiol-F2500

($E_F$ = -773.56559010 a.u.; $G_F$ = -773.25835727 a.u.)

0 1

| | | | |
|---|---|---|---|
| C | -4.124342984837 | -1.779613032290 | -2.631786966715 |
| C | -3.405859366492 | -1.249637272818 | -1.318114215513 |
| C | -4.392993138929 | -1.336358926291 | -0.151886147093 |
| C | -4.748850935961 | -2.814151097106 | 0.100551208970 |
| C | -5.236119875497 | -3.527240193048 | -1.171768218033 |
| C | -4.308293589484 | -3.295152918200 | -2.378272092415 |
| H | -3.471003138863 | -1.592069805203 | -3.492234040368 |
| H | -2.641934781856 | -2.023508112461 | -1.169722813608 |
| H | -3.946897896065 | -0.899994785979 | 0.745461400875 |
| H | -5.284380119460 | -0.753370046134 | -0.397550666136 |
| H | -5.509217212065 | -2.891448248606 | 0.883626956391 |
| H | -3.854744306492 | -3.324515466820 | 0.480983510260 |
| H | -5.336498468111 | -4.600759442106 | -0.984419596189 |
| H | -6.230877305779 | -3.152880908173 | -1.425810161918 |
| H | -4.732866805938 | -3.762851762600 | -3.270904633515 |
| H | -3.335163309205 | -3.770053427074 | -2.216170934849 |
| O | -5.447106543501 | -1.226987724073 | -2.906990435828 |
| O | -2.657898152141 | 0.010772192357 | -1.330121682081 |
| C | -6.074258614509 | -1.608364438578 | -4.167601317397 |
| H | -5.361248839402 | -2.136454130180 | -4.808685674960 |
| H | -6.348658452201 | -0.678832113613 | -4.672832857528 |
| C | -7.385016050827 | -2.473844442763 | -4.067947156805 |
| H | -7.157080694402 | -3.470961359551 | -3.667792777215 |
| H | -8.088205215895 | -1.981812823724 | -3.382204312885 |
| C | -1.483352196193 | -0.079466145748 | -0.465824608106 |

| | | | |
|---|---|---|---|
| H | -0.755134642430 | -0.764910761978 | -0.918027455544 |
| H | -1.766072785793 | -0.492002551799 | 0.509559799433 |
| C | -0.748484686451 | 1.269211136697 | -0.178973728089 |
| H | -0.389474832327 | 1.714280130693 | -1.116296682923 |
| H | -1.439835111544 | 1.976748969570 | 0.297650144427 |
| O | -7.947096810012 | -2.578220358300 | -5.394217777081 |
| C | -9.189633481710 | -3.325318753945 | -5.604028878693 |
| H | -9.071718787679 | -4.378209365389 | -5.320709662933 |
| H | -10.017154362017 | -2.885208669483 | -5.034844756434 |
| H | -9.393123857709 | -3.250054389390 | -6.670873591281 |
| O | 0.356968943654 | 0.955107932826 | 0.699668102233 |
| C | 1.255717351307 | 2.011547867212 | 1.167993507052 |
| H | 1.768593151164 | 2.497634998646 | 0.329402092110 |
| H | 0.712697550869 | 2.766105358865 | 1.749680407682 |
| H | 1.982164359402 | 1.507584963527 | 1.803277759616 |

cisCydiol_TS1-F2000

($E_F$ = -773.35580468 a.u.; $G_F$ = -773.05503275 a.u.)

0 1

| | | | |
|---|---|---|---|
| C | -4.506901489860 | -1.924630542046 | -2.895120576850 |
| C | -3.152921121109 | -0.875538046528 | -1.022617688717 |
| C | -4.250498893635 | -1.058971726139 | -0.001447664808 |
| C | -4.570538412255 | -2.540028819300 | 0.269137059474 |
| C | -5.202291310764 | -3.313731619709 | -0.896238214249 |
| C | -4.396461530015 | -3.261962015476 | -2.203319054346 |
| H | -3.811607613717 | -1.725670853299 | -3.711778160149 |

| | | | |
|---|---|---|---|
| H | -2.480398941125 | -1.718103970988 | -1.179190741278 |
| H | -3.911828383766 | -0.594080939902 | 0.935556212452 |
| H | -5.154153061358 | -0.520197295280 | -0.303962218998 |
| H | -5.244063206950 | -2.595155072141 | 1.131278308539 |
| H | -3.643928671965 | -3.044062668091 | 0.573385655074 |
| H | -5.332999541513 | -4.357444294839 | -0.590876683304 |
| H | -6.201601549622 | -2.917840079631 | -1.100698552592 |
| H | -4.780213809515 | -4.037808027982 | -2.886480820145 |
| H | -3.346590278532 | -3.515652268170 | -2.023818175072 |
| O | -5.851837021887 | -1.632770636770 | -3.226446983702 |
| O | -2.469408875342 | 0.322170828358 | -0.857012526317 |
| C | -6.322086238804 | -2.151831247117 | -4.509912648054 |
| H | -5.754730904371 | -3.046023941607 | -4.785684804390 |
| H | -6.165878464326 | -1.393774259522 | -5.284630110891 |
| C | -7.828710944880 | -2.534497841587 | -4.473710723424 |
| H | -7.988602398546 | -3.311688445267 | -3.713278886211 |
| H | -8.428820824008 | -1.656633376487 | -4.197699591990 |
| C | -1.015324887192 | 0.343562682596 | -0.794985660675 |
| H | -0.631974247026 | 0.822635628030 | -1.699403043625 |
| H | -0.624254969041 | -0.677095432739 | -0.744745720764 |
| C | -0.519318995605 | 1.144053007754 | 0.446711200372 |
| H | -0.956055493236 | 2.151744853184 | 0.421687744635 |
| H | -0.864186002641 | 0.645021896405 | 1.363335154241 |
| O | -8.187573229434 | -3.013879943056 | -5.780873088237 |
| C | -9.551225934887 | -3.462673905928 | -6.004774913054 |
| H | -9.803843898967 | -4.309046618946 | -5.353518908923 |

| | | | |
|---|---|---|---|
| H | -10.270481257369 | -2.651161664398 | -5.836917066031 |
| H | -9.590891134568 | -3.777528590096 | -7.046615700160 |
| O | 0.914119436702 | 1.201705399258 | 0.401654089452 |
| C | 1.605360243989 | 1.916588131100 | 1.463124716491 |
| H | 1.310953017746 | 2.973122973777 | 1.486656359237 |
| H | 1.405898903973 | 1.463479536629 | 2.442217009496 |
| H | 2.666119992693 | 1.836021956778 | 1.230582689232 |

cisCydiol_Int1-F2000

($E_F$ = -773.55403352 a.u.; $G_F$ = -773.25932649 a.u.)

0 1

| | | | |
|---|---|---|---|
| C | -6.699822476624 | -2.206478962166 | -3.655507660401 |
| C | -1.480651336883 | -0.446185176514 | -0.042081910966 |
| C | -2.777059298741 | -0.186405668042 | -0.792976220591 |
| C | -3.463977141782 | -1.462346618598 | -1.395328703200 |
| C | -4.777374955812 | -1.203172450135 | -2.207924122529 |
| C | -5.448917264341 | -2.475821112672 | -2.834138588539 |
| H | -6.756901080344 | -1.311981940235 | -4.275838959597 |
| H | -0.787952958596 | -1.205051864781 | -0.406040826257 |
| H | -3.478088233566 | 0.320752493362 | -0.118509310914 |
| H | -2.591448709090 | 0.527414660197 | -1.613721055390 |
| H | -3.681989532774 | -2.160084525068 | -0.579246174677 |
| H | -2.743886180520 | -1.969987495841 | -2.048435557871 |
| H | -5.506480758020 | -0.707490051049 | -1.557480366125 |
| H | -4.554565961706 | -0.494339789310 | -3.014568677409 |
| H | -5.698976720731 | -3.185738443225 | -2.036033576982 |

| | | | |
|---|---|---|---|
| H | -4.700075491814 | -2.990385717947 | -3.460289551181 |
| O | -7.412051671063 | -3.303260886189 | -4.123871563197 |
| O | -0.896774017830 | 0.622972485600 | 0.625360139076 |
| C | -8.541589299105 | -3.018861647990 | -4.993463549804 |
| H | -8.192735679801 | -2.481010300854 | -5.882432431919 |
| H | -9.258945482668 | -2.383746810509 | -4.463906134157 |
| C | -9.259250436828 | -4.318856169447 | -5.446863700278 |
| H | -8.548241866715 | -4.966211559802 | -5.978592786713 |
| H | -9.625783698564 | -4.863477020876 | -4.565956548537 |
| C | 0.394699210899 | 0.373160817994 | 1.243923904320 |
| H | 1.114553389887 | 0.065749766807 | 0.476743539958 |
| H | 0.299129949176 | -0.437304526011 | 1.973543904350 |
| C | 0.941102643493 | 1.635828911272 | 1.963449683092 |
| H | 1.041451261579 | 2.457582709197 | 1.240965333687 |
| H | 0.234054312143 | 1.949851097853 | 2.743484593613 |
| O | -10.345563154763 | -3.933157513637 | -6.303764048613 |
| C | -11.190843652696 | -4.976193701182 | -6.860705108733 |
| H | -10.612021713856 | -5.663376967984 | -7.490402364079 |
| H | -11.692247868057 | -5.546798440130 | -6.069056424699 |
| H | -11.933590384374 | -4.460510170136 | -7.467523694868 |
| O | 2.214491924646 | 1.289566778599 | 2.530580341190 |
| C | 2.940761334575 | 2.306835995206 | 3.272386109750 |
| H | 3.180678944590 | 3.167921805635 | 2.636178438027 |
| H | 2.367768667017 | 2.649186926400 | 4.143114635024 |
| H | 3.861043003846 | 1.828414842772 | 3.604151620957 |

cisCydiol-F2000

($E_F$ = -773.41005530 a.u.; $G_F$ = -773.10308315 a.u.)

0 1

| | | | |
|---|---|---|---|
| C | -4.101596777786 | -1.868173657086 | -2.536361393004 |
| C | -3.408498811996 | -1.212369792564 | -1.282630122680 |
| C | -4.395597412708 | -1.172427047012 | -0.115354863020 |
| C | -4.814641601234 | -2.605139952842 | 0.263699364595 |
| C | -5.334978315623 | -3.401030735443 | -0.943769346250 |
| C | -4.372265118395 | -3.336880125669 | -2.141517269561 |
| H | -3.409841532774 | -1.805845326570 | -3.385832272660 |
| H | -2.621771272698 | -1.937510880949 | -1.035159850274 |
| H | -3.920567331881 | -0.675351154041 | 0.734766130337 |
| H | -5.264354951160 | -0.576355610150 | -0.407893698384 |
| H | -5.574844067999 | -2.578966160441 | 1.050276213818 |
| H | -3.944225576912 | -3.122881959062 | 0.687314622659 |
| H | -5.507263369497 | -4.443939914495 | -0.660604719289 |
| H | -6.299539541931 | -2.989018268489 | -1.252392757984 |
| H | -4.795864824080 | -3.871022303793 | -2.996800258734 |
| H | -3.427419562082 | -3.837306034413 | -1.902295400204 |
| O | -5.366050721080 | -1.261246803926 | -2.910749146463 |
| O | -2.750624645321 | 0.061608277071 | -1.463460814753 |
| C | -5.935369162120 | -1.683520471926 | -4.175050525160 |
| H | -5.229280391552 | -2.310094944172 | -4.730083321271 |
| H | -6.112247189620 | -0.780724177319 | -4.766198577689 |
| C | -7.295820639303 | -2.443931431068 | -4.076952270164 |
| H | -7.161394053611 | -3.409078265441 | -3.568883133546 |

| | | | |
|---|---|---|---|
| H | -8.004886419802 | -1.844559086384 | -3.489086101577 |
| C | -1.339913058777 | 0.143559918997 | -1.155305024321 |
| H | -0.852642301048 | 0.629534715907 | -2.005050389557 |
| H | -0.909399380962 | -0.859240054553 | -1.053325106258 |
| C | -0.964092990746 | 0.966364390244 | 0.117686797417 |
| H | -1.414540317021 | 1.966268620023 | 0.052780087732 |
| H | -1.351320245797 | 0.473677478567 | 1.021340423501 |
| O | -7.775599691112 | -2.639529399028 | -5.418364886267 |
| C | -9.049642882245 | -3.310050544422 | -5.611847031138 |
| H | -9.027081111200 | -4.331962269944 | -5.212417829808 |
| H | -9.867894698042 | -2.755037658596 | -5.135947177413 |
| H | -9.206386017262 | -3.341569281599 | -6.689140636512 |
| O | 0.471844804395 | 1.046893255482 | 0.155076624925 |
| C | 1.106808288149 | 1.803681851351 | 1.219601142494 |
| H | 0.799691959947 | 2.856879846736 | 1.197219304539 |
| H | 0.872343535358 | 1.380864572040 | 2.204775535274 |
| H | 2.177801663653 | 1.730510819669 | 1.035559278975 |

cisCydiol_TS1-F1500

($E_F$ = -773.19013179 a.u.; $G_F$ = -772.88971768 a.u.)

0 1

| | | | |
|---|---|---|---|
| C | -4.519338548648 | -1.981400361471 | -2.901823882657 |
| C | -3.135618839713 | -0.869200307078 | -0.962347366876 |
| C | -4.261493755221 | -1.054565205850 | 0.023053877491 |
| C | -4.583889701749 | -2.534531868715 | 0.296154803624 |
| C | -5.218471979272 | -3.314233548109 | -0.863911890564 |

| | | | |
|---|---|---|---|
| C | -4.408289963850 | -3.293432334080 | -2.169895796935 |
| H | -3.805946404399 | -1.780037884995 | -3.701654603373 |
| H | -2.457404550772 | -1.707225519358 | -1.117936422257 |
| H | -3.955224982282 | -0.580471634379 | 0.967199513805 |
| H | -5.159478116617 | -0.523027286179 | -0.309323923961 |
| H | -5.259260905964 | -2.584819796917 | 1.157126698364 |
| H | -3.659498363023 | -3.040004954026 | 0.604808669710 |
| H | -5.362919453912 | -4.351978111384 | -0.544534322043 |
| H | -6.212815298547 | -2.910166220403 | -1.077204132390 |
| H | -4.787208036870 | -4.091061683132 | -2.831689707505 |
| H | -3.357581661404 | -3.535734391426 | -1.979703949336 |
| O | -5.849274462375 | -1.679291289079 | -3.239280788264 |
| O | -2.482312429276 | 0.337833681443 | -0.808753601049 |
| C | -6.299457518367 | -2.161092274056 | -4.535375058397 |
| H | -5.736072328068 | -3.053853997718 | -4.824783749742 |
| H | -6.132380928089 | -1.387925146226 | -5.293165108039 |
| C | -7.799250874525 | -2.529355616230 | -4.501386352530 |
| H | -7.963994624083 | -3.315567903688 | -3.750865380548 |
| H | -8.393770266146 | -1.651494896760 | -4.212062328097 |
| C | -1.036487308254 | 0.395952495485 | -0.829902768412 |
| H | -0.718465506535 | 0.951342627013 | -1.715866809856 |
| H | -0.613081277272 | -0.612084421920 | -0.876290383044 |
| C | -0.514947486818 | 1.118338787694 | 0.436358192928 |
| H | -0.988406012358 | 2.107629441298 | 0.506488479322 |
| H | -0.798090183773 | 0.541935751216 | 1.329080323810 |
| O | -8.157858913128 | -2.986166366482 | -5.809994282423 |

| | | | |
|---|---|---|---|
| C | -9.517479914787 | -3.426939705318 | -6.003150641864 |
| H | -9.757784936517 | -4.278333881842 | -5.352811228063 |
| H | -10.232367665900 | -2.616563101570 | -5.809431138050 |
| H | -9.591133701595 | -3.734278795180 | -7.045725084677 |
| O | 0.905712684816 | 1.233437087998 | 0.329305657678 |
| C | 1.586764774182 | 1.899663265507 | 1.414204272785 |
| H | 1.241365774613 | 2.935806148120 | 1.524700325838 |
| H | 1.438367617790 | 1.369195790510 | 2.363900555374 |
| H | 2.645094105967 | 1.894326178123 | 1.156056601957 |

cisCydiol -F1500

($E_F$ = -773.25901380 a.u.; $G_F$ = -772.95127131 a.u.)

0 1

| | | | |
|---|---|---|---|
| C | -4.078138461281 | -1.909730225937 | -2.497032700029 |
| C | -3.402806568274 | -1.222037834777 | -1.268493788916 |
| C | -4.382861186977 | -1.166077271421 | -0.096077971466 |
| C | -4.808893851418 | -2.589157023736 | 0.307147278592 |
| C | -5.348755295133 | -3.392167560314 | -0.886502603579 |
| C | -4.381989837785 | -3.364199737109 | -2.081335288090 |
| H | -3.373685608900 | -1.879326670259 | -3.338445298042 |
| H | -2.586040295747 | -1.906643238078 | -1.000225390200 |
| H | -3.903656383629 | -0.655213277488 | 0.743637662214 |
| H | -5.249828879771 | -0.571313128099 | -0.396328309178 |
| H | -5.560402521483 | -2.545259424578 | 1.101209507001 |
| H | -3.939502784249 | -3.111187185927 | 0.727823476446 |
| H | -5.543649524897 | -4.426788655888 | -0.588224356700 |

| | | | |
|---|---|---|---|
| H | -6.304199763809 | -2.963391859361 | -1.201572204474 |
| H | -4.808560504320 | -3.904586459918 | -2.931280343490 |
| H | -3.446474306469 | -3.875676779550 | -1.827600354030 |
| O | -5.309255179208 | -1.264585416520 | -2.889456492016 |
| O | -2.819178096661 | 0.067223169139 | -1.522366202970 |
| C | -5.859921691254 | -1.665527246567 | -4.159602160803 |
| H | -5.158674961781 | -2.304143453742 | -4.707733377939 |
| H | -6.014414088329 | -0.758923378446 | -4.751695630346 |
| C | -7.223231892613 | -2.399076958641 | -4.063586125552 |
| H | -7.109220356327 | -3.359736467286 | -3.541536456139 |
| H | -7.930008059587 | -1.782488217671 | -3.490240377389 |
| C | -1.409859018812 | 0.219771397731 | -1.287431031172 |
| H | -1.031922964526 | 0.879674559400 | -2.072147646278 |
| H | -0.891686068872 | -0.742121738571 | -1.383297346933 |
| C | -1.023942542926 | 0.856135586989 | 0.074757057465 |
| H | -1.560494538118 | 1.807503075541 | 0.197168564102 |
| H | -1.307644970831 | 0.194355823748 | 0.906893099769 |
| O | -7.687331753985 | -2.602327212684 | -5.403071940240 |
| C | -8.962462404280 | -3.254508924401 | -5.569445079995 |
| H | -8.952492679974 | -4.267476182798 | -5.145637041268 |
| H | -9.770403631877 | -2.678821809787 | -5.099032503337 |
| H | -9.135863004033 | -3.312462723373 | -6.643473207256 |
| O | 0.393625188305 | 1.060606066405 | 0.047036633233 |
| C | 0.998085440632 | 1.710265518095 | 1.183068871005 |
| H | 0.590446410026 | 2.719021424238 | 1.330159148854 |
| H | 0.851619495780 | 1.128556410842 | 2.102815396819 |

| | | | |
|---|---|---|---|
| H | 2.062951730384 | 1.777590249072 | 0.962631685870 |

cisCydiol_Int1-F1500

($E_F$ = -773.33859758 a.u.; $G_F$ = -773.04324928 a.u.)

0 1

| | | | |
|---|---|---|---|
| C | -6.676931495362 | -2.189578772132 | -3.635682444540 |
| C | -1.505012705171 | -0.465156166782 | -0.057023603559 |
| C | -2.796966405525 | -0.196340213877 | -0.794159187343 |
| C | -3.467028817132 | -1.467541662933 | -1.399666182595 |
| C | -4.775592744233 | -1.201502022774 | -2.198351524474 |
| C | -5.429848746547 | -2.468934612901 | -2.828552593438 |
| H | -6.738244111306 | -1.289885317513 | -4.248245770789 |
| H | -0.808409488768 | -1.213476045599 | -0.435427343959 |
| H | -3.497910909164 | 0.299318751673 | -0.110865794093 |
| H | -2.618392380989 | 0.527654339988 | -1.607996449176 |
| H | -3.677784207202 | -2.174944601573 | -0.589797941659 |
| H | -2.745962191950 | -1.964008955593 | -2.060581441507 |
| H | -5.506062491070 | -0.717513455053 | -1.540394425529 |
| H | -4.559788111317 | -0.482623090237 | -2.998260819894 |
| H | -5.674105947981 | -3.189008529096 | -2.037577710652 |
| H | -4.679970744452 | -2.972465315476 | -3.462990869277 |
| O | -7.378903146061 | -3.283769838803 | -4.107964861676 |
| O | -0.932513142405 | 0.597974121012 | 0.617515246106 |
| C | -8.507437610203 | -2.992537580683 | -4.963497385431 |
| H | -8.168869459719 | -2.441171638456 | -5.848601261627 |
| H | -9.229384389651 | -2.370656476360 | -4.423908421698 |

| | | | |
|---|---|---|---|
| C | -9.200546648535 | -4.293241831934 | -5.418989326741 |
| H | -8.482280609193 | -4.927070995564 | -5.958004923772 |
| H | -9.556437350171 | -4.850652841481 | -4.541247895745 |
| C | 0.354791025502 | 0.344795655514 | 1.225044370716 |
| H | 1.076364144367 | 0.048931977332 | 0.454438885652 |
| H | 0.267508077647 | -0.472156852332 | 1.948927900860 |
| C | 0.878962821191 | 1.604526755696 | 1.944780567571 |
| H | 0.967415569962 | 2.432172405968 | 1.226884332235 |
| H | 0.169390410904 | 1.906086683418 | 2.727968597323 |
| O | -10.289639942651 | -3.913935699539 | -6.265411193210 |
| C | -11.099531893246 | -4.976380423630 | -6.810058293933 |
| H | -10.503540123850 | -5.651096985164 | -7.438353317943 |
| H | -11.580744106593 | -5.559894773716 | -6.014400569367 |
| H | -11.862884316545 | -4.492920600722 | -7.418504776357 |
| O | 2.152162559342 | 1.268912646625 | 2.503972848134 |
| C | 2.839565132714 | 2.301934221337 | 3.239940713339 |
| H | 3.056335166287 | 3.169452996809 | 2.603050884648 |
| H | 2.253080347640 | 2.632164015082 | 4.107176826802 |
| H | 3.774472158601 | 1.858858971355 | 3.580957310499 |

cisCydiol_TS1-F1000

($E_F$ = -773.02540082 a.u.; $G_F$ = -772.72663295 a.u.)

0 1

| | | | |
|---|---|---|---|
| C | -4.604682296866 | -2.146301779771 | -2.874882282955 |
| C | -3.106866196382 | -0.870542341796 | -0.925399877726 |
| C | -4.284889281573 | -0.960975685837 | 0.006368374955 |

| | | | |
|---|---|---|---|
| C | -4.643305459813 | -2.407941413919 | 0.390517152867 |
| C | -5.306619129454 | -3.260124324036 | -0.700731622574 |
| C | -4.513882904747 | -3.374369219171 | -2.013404992752 |
| H | -3.910948079446 | -2.037796457154 | -3.707217343647 |
| H | -2.441386853655 | -1.729582184189 | -1.001981351750 |
| H | -4.027011242473 | -0.406839474979 | 0.921540444235 |
| H | -5.159478890848 | -0.455433502687 | -0.419440133564 |
| H | -5.317309499150 | -2.376065735168 | 1.253468984369 |
| H | -3.731831562163 | -2.912787563721 | 0.736395831993 |
| H | -5.472159240541 | -4.264811134190 | -0.296817476318 |
| H | -6.294607300788 | -2.851987084235 | -0.934647184686 |
| H | -4.913945019360 | -4.228196873921 | -2.587947495732 |
| H | -3.463547500002 | -3.608538295375 | -1.811655146310 |
| O | -5.911723375832 | -1.779079411068 | -3.153276500076 |
| O | -2.451261056314 | 0.340200603176 | -0.856322884119 |
| C | -6.400742169440 | -1.841029373352 | -4.509456417642 |
| H | -5.639079376561 | -2.253493437069 | -5.178057301240 |
| H | -6.650506026477 | -0.830373720443 | -4.844544388012 |
| C | -7.663964103341 | -2.718584210498 | -4.561351195518 |
| H | -7.410853233713 | -3.747011479791 | -4.263898651711 |
| H | -8.401894452690 | -2.330969951480 | -3.844329465917 |
| C | -1.014180639379 | 0.380464439263 | -0.908859520162 |
| H | -0.709354709038 | 0.958423110671 | -1.785107951423 |
| H | -0.599043162702 | -0.628667267847 | -0.993792154645 |
| C | -0.475270331758 | 1.060190216594 | 0.362403849116 |
| H | -0.946833970589 | 2.047418222615 | 0.469704941053 |

| | | | |
|---|---|---|---|
| H | -0.744875949419 | 0.458901166750 | 1.243424395838 |
| O | -8.173219448493 | -2.681246272173 | -5.891619478926 |
| C | -9.374979913558 | -3.431193196227 | -6.112148940869 |
| H | -9.224760952142 | -4.499499253306 | -5.904859418690 |
| H | -10.198324326370 | -3.061049995246 | -5.486269215088 |
| H | -9.634095892223 | -3.301092154035 | -7.162653144229 |
| O | 0.938467624048 | 1.177020006719 | 0.235242811873 |
| C | 1.603013357595 | 1.820183884669 | 1.331140624243 |
| H | 1.244973268744 | 2.849514017178 | 1.467240818842 |
| H | 1.457017988716 | 1.266840752577 | 2.268676162517 |
| H | 2.664209365466 | 1.837049123297 | 1.084114416117 |

cisCydiol_Int1-F1000

($E_F$ = -773.12533226 a.u.; $G_F$ = -772.83038674 a.u.)

0 1

| | | | |
|---|---|---|---|
| C | -6.690001494503 | -2.139372314423 | -3.583142881179 |
| C | -1.529247101019 | -0.515551110631 | -0.050648290387 |
| C | -2.851356262149 | -0.240689858064 | -0.711178624529 |
| C | -3.421506949061 | -1.455121180615 | -1.487863128135 |
| C | -4.797726784271 | -1.199795656235 | -2.145953742065 |
| C | -5.367872939391 | -2.414223360255 | -2.922646958852 |
| H | -6.877116171519 | -1.173362008947 | -4.052646889727 |
| H | -1.342176909640 | -1.481542438735 | 0.418911097824 |
| H | -3.591612115343 | 0.077820919916 | 0.043405567969 |
| H | -2.738170351612 | 0.614206814589 | -1.389760674167 |
| H | -3.508445656766 | -2.306945945216 | -0.802065124345 |

| | | | |
|---|---|---|---|
| H | -2.701219883192 | -1.753746561221 | -2.257994556156 |
| H | -5.518014842280 | -0.901178337228 | -1.375819876527 |
| H | -4.710791387511 | -0.347965727696 | -2.831745861833 |
| H | -5.481029692388 | -3.269133726418 | -2.244076460828 |
| H | -4.627628063948 | -2.732709558447 | -3.677252412418 |
| O | -7.324703155782 | -3.218807703639 | -4.157077615545 |
| O | -0.894519159187 | 0.563892238378 | 0.523242745234 |
| C | -8.534929099380 | -2.920750780266 | -4.875633394982 |
| H | -8.322704703349 | -2.221943411643 | -5.693730525507 |
| H | -9.263945453093 | -2.455432434503 | -4.203554738655 |
| C | -9.127294229997 | -4.210480444323 | -5.456093583487 |
| H | -8.397102499501 | -4.686836329236 | -6.126057636852 |
| H | -9.353701798555 | -4.915120965201 | -4.643178262832 |
| C | 0.315656060123 | 0.265824094086 | 1.241879747700 |
| H | 1.044671318758 | -0.199609513405 | 0.569878279317 |
| H | 0.103348236828 | -0.432889095892 | 2.060036581595 |
| C | 0.908081045336 | 1.555570998552 | 1.822241497588 |
| H | 1.134589450977 | 2.260112445097 | 1.009265635684 |
| H | 0.177882208381 | 2.032050152704 | 2.492113420421 |
| O | -10.309033569080 | -3.840700545493 | -6.161543964547 |
| C | -11.026523580569 | -4.915289739133 | -6.781234258900 |
| H | -10.409668976940 | -5.429923525949 | -7.530289142313 |
| H | -11.369385171337 | -5.648981943498 | -6.039249996803 |
| H | -11.890692644100 | -4.468132476237 | -7.271804595988 |
| O | 2.089751361445 | 1.185775005041 | 2.527801865700 |
| C | 2.807290798313 | 2.260378923433 | 3.147410467154 |

| | | | |
|---|---|---|---|
| H | 3.150264416287 | 2.993956187134 | 2.405356569986 |
| H | 2.190427685329 | 2.775157377889 | 3.896366487644 |
| H | 3.671391740810 | 1.813203567539 | 3.638091792760 |

cisCydiol-F1000

($E_F$ = -773.11081334 a.u.; $G_F$ = -772.80261922 a.u.)

0 1

| | | | |
|---|---|---|---|
| C | -4.068581944945 | -1.950633682302 | -2.449442568005 |
| C | -3.405571992016 | -1.224655038251 | -1.251500385880 |
| C | -4.385980390910 | -1.119412044214 | -0.082827108431 |
| C | -4.843332686085 | -2.519557966583 | 0.361492518968 |
| C | -5.408325154752 | -3.337476223018 | -0.809863587463 |
| C | -4.432610191456 | -3.377731504054 | -1.996121902517 |
| H | -3.347629420619 | -1.975698523899 | -3.277427966711 |
| H | -2.573729527837 | -1.877272380009 | -0.950078303798 |
| H | -3.901229860628 | -0.590289846524 | 0.742337038185 |
| H | -5.239905558523 | -0.518110687466 | -0.406166920474 |
| H | -5.589733331845 | -2.435126783627 | 1.157050401844 |
| H | -3.985926072281 | -3.053260044336 | 0.792235879895 |
| H | -5.642319371134 | -4.355214425461 | -0.483149090398 |
| H | -6.345997709941 | -2.883227812248 | -1.143054755157 |
| H | -4.867908330277 | -3.927696114862 | -2.835577403633 |
| H | -3.517160494646 | -3.912216821187 | -1.716537422430 |
| O | -5.252271582328 | -1.255027786743 | -2.876859619503 |
| O | -2.869494863146 | 0.060689345584 | -1.590053868366 |
| C | -5.779951341140 | -1.638646025473 | -4.153558801682 |

| | | | |
|---|---|---|---|
| H | -5.095317886110 | -2.313789349360 | -4.679020251189 |
| H | -5.883768424729 | -0.733620157349 | -4.759511818215 |
| C | -7.163770375916 | -2.310522708099 | -4.060407934507 |
| H | -7.095663334135 | -3.260111033804 | -3.510224089210 |
| H | -7.854906485394 | -1.652118148737 | -3.514480767439 |
| C | -1.464454851770 | 0.246408531366 | -1.407311086656 |
| H | -1.153155022854 | 0.995099649570 | -2.139212142750 |
| H | -0.912100312054 | -0.677785390661 | -1.620222219750 |
| C | -1.060843987639 | 0.758497898406 | -0.009153861865 |
| H | -1.619962233487 | 1.677819590009 | 0.216886056350 |
| H | -1.304803193679 | 0.013678454348 | 0.764006652550 |
| O | -7.611775508313 | -2.530637682751 | -5.396801759005 |
| C | -8.902043205692 | -3.137279001329 | -5.532632287204 |
| H | -8.928185141319 | -4.133957572757 | -5.071001542783 |
| H | -9.687013248213 | -2.516861120232 | -5.079116118366 |
| H | -9.090460497667 | -3.231202807459 | -6.601970205220 |
| O | 0.343849069558 | 1.004451198002 | -0.051844247407 |
| C | 0.922993679872 | 1.571938260085 | 1.128465744304 |
| H | 0.478795571825 | 2.548771639994 | 1.363229183276 |
| H | 0.799427003142 | 0.909478423860 | 1.996225376700 |
| H | 1.985598245767 | 1.699676901652 | 0.922495795006 |

cisCydiol_TS1-F500

($E_F$ = -772.86221468 a.u.; $G_F$ = -772.56392193 a.u.)

0 1

| | | | |
|---|---|---|---|
| C | -4.655333936871 | -2.476305287491 | -2.792760622036 |

| | | | |
|---|---|---|---|
| C | -3.065377774146 | -0.764379937799 | -0.863556997650 |
| C | -4.251316325987 | -0.854602427347 | 0.048006183070 |
| C | -4.523696051303 | -2.279627366113 | 0.560661673901 |
| C | -5.179976155258 | -3.259895735790 | -0.422308044488 |
| C | -4.414147560776 | -3.510755520336 | -1.736753919120 |
| H | -3.968572735954 | -2.384218976067 | -3.631510315645 |
| H | -2.316476954023 | -1.555065463578 | -0.847840981737 |
| H | -4.059187037354 | -0.199807626462 | 0.913326765187 |
| H | -5.147855505005 | -0.450526567886 | -0.438213595134 |
| H | -5.172273228212 | -2.206064207466 | 1.440257998030 |
| H | -3.579016741711 | -2.709588406408 | 0.918855548674 |
| H | -5.305957111513 | -4.216762343068 | 0.095918384053 |
| H | -6.186839939156 | -2.911759958589 | -0.672211408780 |
| H | -4.732552459116 | -4.489903826791 | -2.135178743750 |
| H | -3.339604356799 | -3.589742976202 | -1.546589332714 |
| O | -5.991255510402 | -2.350866473892 | -3.099289801586 |
| O | -2.568572785513 | 0.505935405551 | -1.027362099782 |
| C | -6.386938512398 | -2.053316936540 | -4.441575505074 |
| H | -5.639127555871 | -2.412977091404 | -5.156334087873 |
| H | -6.507786218535 | -0.973157988385 | -4.573061529446 |
| C | -7.713637477799 | -2.773164351826 | -4.682387029750 |
| H | -7.566705624402 | -3.852126606638 | -4.526812403881 |
| H | -8.457212989377 | -2.426529900506 | -3.949858055214 |
| C | -1.148726409683 | 0.663722528352 | -1.123429429271 |
| H | -0.961258836601 | 1.496001104165 | -1.803911575001 |
| H | -0.679236329873 | -0.235268157694 | -1.536989048292 |

| | | | |
|---|---|---|---|
| C | -0.555597508871 | 0.987604498547 | 0.250659010517 |
| H | -1.063243092681 | 1.872786189291 | 0.660628638697 |
| H | -0.730459371869 | 0.147908081926 | 0.941117490449 |
| O | -8.137001573671 | -2.501800866623 | -6.008795599258 |
| C | -9.346521089955 | -3.163447307446 | -6.370861959088 |
| H | -9.239772702015 | -4.255409773972 | -6.310503383779 |
| H | -10.181379604071 | -2.855448920053 | -5.726352202308 |
| H | -9.566890968308 | -2.881703398569 | -7.400485701465 |
| O | 0.832615043830 | 1.226694015570 | 0.077193888099 |
| C | 1.507113757933 | 1.641289912436 | 1.262241074241 |
| H | 1.091653687279 | 2.580899317062 | 1.651949417179 |
| H | 1.445839652779 | 0.876380524288 | 2.048896213447 |
| H | 2.552533950528 | 1.795625574584 | 0.994788358319 |

cisCydiol_Int1-F500

($E_F$ = -772.90446825 a.u.; $G_F$ = -772.61078810 a.u.)

0 1

| | | | |
|---|---|---|---|
| C | -6.424661637136 | -1.609892373652 | -3.845554576189 |
| C | -1.774973998571 | -0.828912286505 | 0.058288504987 |
| C | -3.014100612530 | -0.646130392613 | -0.761193643627 |
| C | -3.775927668334 | -1.975940645163 | -0.939805824414 |
| C | -5.147229673396 | -1.877480740946 | -1.637564329738 |
| C | -5.091698168838 | -1.712296375402 | -3.176537524864 |
| H | -6.702353658921 | -0.745660505057 | -4.443985456529 |
| H | -1.736824745124 | -1.605526655975 | 0.822604035357 |
| H | -3.681714654807 | 0.086849736677 | -0.275932385031 |

| | | | |
|---|---|---|---|
| H | -2.754222414552 | -0.210076603234 | -1.734247196670 |
| H | -3.934015284902 | -2.407526036370 | 0.055469264732 |
| H | -3.139780790090 | -2.686989871543 | -1.480380916116 |
| H | -5.718754868674 | -2.785704843387 | -1.420597601149 |
| H | -5.718388974768 | -1.045549992981 | -1.209703058759 |
| H | -4.535670100149 | -2.564479310847 | -3.595245605873 |
| H | -4.520241755499 | -0.811543793782 | -3.424155643438 |
| O | -7.061672208531 | -2.785705299167 | -4.154690043803 |
| O | -1.035333441079 | 0.297548120490 | 0.315705338498 |
| C | -8.330314030262 | -2.629988804355 | -4.801394776660 |
| H | -8.237053998414 | -1.950398049756 | -5.657413717995 |
| H | -9.059708262279 | -2.206014892497 | -4.102608424267 |
| C | -8.816834301246 | -3.987246208331 | -5.296834767910 |
| H | -8.079234301114 | -4.416286225833 | -5.990676941379 |
| H | -8.932025837466 | -4.680923025760 | -4.451384783580 |
| C | 0.088320375820 | 0.095319600220 | 1.181361094343 |
| H | 0.810400048548 | -0.580869276274 | 0.710710868712 |
| H | -0.239231668369 | -0.352992733478 | 2.127449526142 |
| C | 0.750658022355 | 1.438525065331 | 1.466412783558 |
| H | 1.096735977517 | 1.894837931595 | 0.527665053381 |
| H | 0.023724157214 | 2.122201693230 | 1.928797685680 |
| O | -10.059107880597 | -3.753927273453 | -5.945014001638 |
| C | -10.662667569079 | -4.915436476574 | -6.505658814304 |
| H | -10.021049505370 | -5.371949682506 | -7.272121836665 |
| H | -10.880328044419 | -5.668043810852 | -5.735134269766 |
| H | -11.597206456533 | -4.595041191148 | -6.966234095221 |

| | | | |
|---|---|---|---|
| O | 1.837478909092 | 1.173672058710 | 2.341674689147 |
| C | 2.579638782714 | 2.325857709701 | 2.728482173083 |
| H | 3.027456069794 | 2.826813316604 | 1.858941394234 |
| H | 1.949549224921 | 3.048813959836 | 3.265090094432 |
| H | 3.373871242516 | 1.982533816607 | 3.391414851132 |

cisCydiol -F500

($E_F$ = -772.96547629 a.u.; $G_F$ = -772.65718030 a.u.)

0 1

| | | | |
|---|---|---|---|
| C | -4.056825416068 | -2.009990667709 | -2.408535952978 |
| C | -3.423684556316 | -1.234241196727 | -1.238142149830 |
| C | -4.406299556026 | -1.112546287575 | -0.073223515273 |
| C | -4.855309000180 | -2.503521646733 | 0.404633240377 |
| C | -5.423475838371 | -3.345341870178 | -0.747813871136 |
| C | -4.441114540698 | -3.420130632543 | -1.926196169281 |
| H | -3.321099151671 | -2.065483457507 | -3.222342926178 |
| H | -2.561419141564 | -1.832496296635 | -0.910426386465 |
| H | -3.929757411711 | -0.556636048679 | 0.739083567597 |
| H | -5.264249538467 | -0.527663660660 | -0.415314472482 |
| H | -5.598069086743 | -2.403595901788 | 1.201758031541 |
| H | -3.994917169599 | -3.026772319683 | 0.842394944212 |
| H | -5.666627006042 | -4.353213917157 | -0.398144982417 |
| H | -6.356420097318 | -2.891730258193 | -1.095669872365 |
| H | -4.872815381633 | -3.986030960216 | -2.757040692200 |
| H | -3.531392820888 | -3.953872723769 | -1.626000124699 |
| O | -5.212609024017 | -1.298133996840 | -2.866158860427 |

| | | | |
|---|---|---|---|
| O | -2.967039390369 | 0.057213965855 | -1.650159397743 |
| C | -5.707701247343 | -1.671662348267 | -4.151244932834 |
| H | -5.030163452326 | -2.371024096478 | -4.654217811675 |
| H | -5.774638934780 | -0.770481245405 | -4.768666281826 |
| C | -7.100038648150 | -2.303307900640 | -4.060142250511 |
| H | -7.059517465030 | -3.240858652961 | -3.486379688240 |
| H | -7.780461841258 | -1.616760705898 | -3.535199458857 |
| C | -1.568773831031 | 0.294351472762 | -1.538235079054 |
| H | -1.334037853042 | 1.106654477516 | -2.229326726423 |
| H | -0.986758970350 | -0.584791268977 | -1.845477332028 |
| C | -1.138004451168 | 0.716323658089 | -0.128174301469 |
| H | -1.711751383331 | 1.603728749586 | 0.176810633447 |
| H | -1.346818917264 | -0.085728718243 | 0.597166428964 |
| O | -7.539327037584 | -2.543098345488 | -5.390439819891 |
| C | -8.836566395686 | -3.121330713241 | -5.488011106852 |
| H | -8.879442490517 | -4.101327216350 | -4.991883979691 |
| H | -9.602244987714 | -2.469782629914 | -5.044000518222 |
| H | -9.048669046531 | -3.249356861715 | -6.549724993783 |
| O | 0.254126705070 | 0.996330634342 | -0.184198576188 |
| C | 0.811216395228 | 1.484077804848 | 1.031706398724 |
| H | 0.338656432422 | 2.426828817869 | 1.340975820108 |
| H | 0.703663853536 | 0.753963488402 | 1.846408085078 |
| H | 1.871453242439 | 1.659263721929 | 0.847697477407 |

transCydiol_TS1-F4000

($E_F$ = -774.04729761 a.u.; $G_F$ = -773.74861605 a.u.)

0 1

| | | | |
|---|---|---|---|
| C | -1.077780016770 | -0.419261289721 | 0.533447646962 |
| C | 1.077780664887 | -0.418966126486 | -0.533652019327 |
| C | 0.837625414371 | 0.846488367137 | -1.307942131504 |
| C | 0.653279052284 | 2.077890630278 | -0.400617568766 |
| C | -0.653329638670 | 2.077672786541 | 0.401712296026 |
| C | -0.837655776999 | 0.845796156714 | 1.308396391102 |
| H | -0.980964485150 | -1.357024795578 | 1.079046895692 |
| H | 1.691381593251 | 1.026349903003 | -1.980738151637 |
| H | -0.050327731656 | 0.703772750631 | -1.933377496912 |
| H | 0.688602907958 | 2.980339205791 | -1.019172154266 |
| H | 1.506593385127 | 2.129361493071 | 0.283896554822 |
| H | -0.688666165608 | 2.979798687967 | 1.020736684193 |
| H | -1.506645674241 | 2.129487011112 | -0.282773834817 |
| H | -1.691420358736 | 1.025290082198 | 1.981279868856 |
| H | 0.050295864222 | 0.702778484142 | 1.933764721247 |
| O | -2.310806138189 | -0.356836924887 | -0.224855925789 |
| C | -3.577924720516 | -0.796015163722 | 0.454008470081 |
| H | -3.587212656287 | -0.398051285795 | 1.472066391571 |
| H | -3.581044380284 | -1.888659136366 | 0.503867745446 |
| C | -4.932880972903 | -0.321922003668 | -0.262263461598 |
| H | -4.937558283517 | 0.773841365108 | -0.309077708119 |
| H | -4.947515707650 | -0.714001878068 | -1.286320910399 |
| O | -6.093378738494 | -0.808781503954 | 0.491138667860 |
| C | -7.527283782451 | -0.537264227424 | 0.092930673938 |
| H | -7.732481591118 | 0.537397367428 | 0.095307145431 |

| | | | |
|---|---|---|---|
| H | -7.742642379251 | -0.963002162861 | -0.891624410169 |
| H | -8.105909731234 | -1.040441759289 | 0.863790202534 |
| H | 0.980979600654 | -1.356447938909 | -1.079737586874 |
| O | 2.310808893089 | -0.356912869530 | 0.224678391006 |
| C | 3.577933861416 | -0.795718659762 | -0.454414218985 |
| H | 3.581076428251 | -1.888337637544 | -0.504831696659 |
| H | 3.587210550054 | -0.397234063072 | -1.472269309855 |
| C | 4.932882373778 | -0.321963819069 | 0.262097180418 |
| H | 4.937544802010 | 0.773777163362 | 0.309459566539 |
| H | 4.947520599522 | -0.714556026157 | 1.285959511791 |
| O | 6.093388052045 | -0.808431548982 | -0.491546689016 |
| C | 7.527288456017 | -0.537080746517 | -0.093208122366 |
| H | 7.732467211775 | 0.537587012153 | -0.095067166528 |
| H | 7.742655354059 | -0.963289574001 | 0.891143023737 |
| H | 8.105922864936 | -1.039877325296 | -0.864311465609 |

transCydiol_Int1-F4000

($E_F$ = -774.43983505 a.u.; $G_F$ = -774.14950871 a.u.)

0 1

| | | | |
|---|---|---|---|
| C | -3.449044744805 | -0.040988046767 | -0.228165692665 |
| C | 3.449038494052 | -0.041035306978 | 0.228158061716 |
| C | 2.068289552605 | 0.003589198553 | -0.510013227148 |
| C | 0.713790541848 | 0.015727943974 | 0.380859811137 |
| C | -0.713797231072 | 0.015835977021 | -0.380848775106 |
| C | -2.068296224814 | 0.003418060863 | 0.510020605816 |
| H | -3.537558766304 | -0.628496141594 | -1.141400094600 |

| | | | |
|---|---|---|---|
| H | 2.062602969468 | 0.885276627464 | -1.160849149244 |
| H | 2.006121615459 | -0.862164198237 | -1.188008037819 |
| H | 0.751572569566 | 0.893742389598 | 1.033248749246 |
| H | 0.741319211687 | -0.858974077882 | 1.039698758069 |
| H | -0.751586796784 | 0.894046096498 | -1.032974333000 |
| H | -0.741318340534 | -0.858668600438 | -1.039950390923 |
| H | -2.062614194100 | 0.884908021662 | 1.161125185826 |
| H | -2.006124037382 | -0.862541659618 | 1.187752322944 |
| O | -4.652064136624 | 0.083347210912 | 0.509773941997 |
| C | -5.952494416584 | -0.079997695216 | -0.207632589608 |
| H | -5.978752351051 | -1.080654491845 | -0.649122591927 |
| H | -5.996478297693 | 0.660295377888 | -1.010461137567 |
| C | -7.257688039791 | 0.095760394796 | 0.706672173781 |
| H | -7.222410747965 | -0.642700927713 | 1.516763118636 |
| H | -7.243578632433 | 1.098826534628 | 1.149675183601 |
| O | -8.462291149483 | -0.090594830311 | -0.107003025332 |
| C | -9.868478517585 | 0.014477625719 | 0.441042743778 |
| H | -10.039634333383 | -0.740412276334 | 1.214100415472 |
| H | -10.054066882419 | 1.016039051462 | 0.839987951367 |
| H | -10.495534596712 | -0.175447431943 | -0.426641119360 |
| H | 3.537559054509 | -0.628836429430 | 1.141202076369 |
| O | 4.652056443030 | 0.083548344070 | -0.509741371926 |
| C | 5.952488513978 | -0.080014620666 | 0.207613029719 |
| H | 5.996467169148 | 0.660024822034 | 1.010677979597 |
| H | 5.978754086990 | -1.080813181487 | 0.648785522167 |
| C | 7.257680190116 | 0.096044073578 | -0.706637898007 |

| | | | |
|---|---|---|---|
| H | 7.222408554993 | -0.642162953517 | -1.516965311249 |
| H | 7.243562248505 | 1.099253082214 | -1.149324840415 |
| O | 8.462284043556 | -0.090559046162 | 0.106981469439 |
| C | 9.868469448295 | 0.014695805462 | -0.441033492859 |
| H | 10.039633237861 | -0.739959605455 | -1.214325627576 |
| H | 10.054048433162 | 1.016385647549 | -0.839674665876 |
| H | 10.495528245316 | -0.175489178343 | 0.426596289664 |

transCydiol-F4000

($E_F$ = -774.07765103 a.u.; $G_F$ = -773.77362826 a.u.)

0 1

| | | | |
|---|---|---|---|
| C | -0.634834263967 | -0.467697573224 | 0.501663849848 |
| C | 0.634827262609 | -0.467446162216 | -0.501881275744 |
| C | 0.535895202028 | 0.782977851188 | -1.376094051780 |
| C | 0.561378927066 | 2.061078981085 | -0.524225367298 |
| C | -0.561386403732 | 2.060821750306 | 0.525263501282 |
| C | -0.535913825740 | 0.782295051409 | 1.376495790208 |
| H | -0.594062814367 | -1.376707141919 | 1.110932880166 |
| H | 1.333815172905 | 0.795029558643 | -2.123192675655 |
| H | -0.411535285846 | 0.717493926609 | -1.920583697339 |
| H | 0.473520573493 | 2.939509519961 | -1.170858387531 |
| H | 1.529884401656 | 2.130026460340 | -0.017659410199 |
| H | -0.473520374458 | 2.938928121540 | 1.172335670119 |
| H | -1.529891645249 | 2.130032228912 | 0.018732210615 |
| H | -1.333843201337 | 0.793976272038 | 2.123590269157 |
| H | 0.411509545958 | 0.716542233168 | 1.920965264569 |

| | | | |
|---|---|---|---|
| O | -1.926131161286 | -0.492560782362 | -0.268987956833 |
| C | -3.188571164267 | -0.546316937529 | 0.507394780188 |
| H | -3.256155152526 | 0.324727418268 | 1.165082461135 |
| H | -3.187873895747 | -1.447115591306 | 1.131439497485 |
| C | -4.530200467535 | -0.556496241253 | -0.372543055163 |
| H | -4.529616050409 | 0.329193608825 | -1.019846845302 |
| H | -4.536329474165 | -1.450634873758 | -1.007449361415 |
| O | -5.702541777911 | -0.545874941773 | 0.510304324180 |
| C | -7.130100953677 | -0.552067903923 | 0.013481939217 |
| H | -7.335107191978 | 0.338933461362 | -0.587582393934 |
| H | -7.334417427369 | -1.456527174818 | -0.567347110624 |
| H | -7.719162679135 | -0.542030125123 | 0.927371023457 |
| H | 0.594060432739 | -1.376154947858 | -1.111599094734 |
| O | 1.926127491081 | -0.492684563739 | 0.268754053289 |
| C | 3.188567697930 | -0.545955052519 | -0.507663104168 |
| H | 3.187872810205 | -1.446367139663 | -1.132271746131 |
| H | 3.256149938819 | 0.325502525641 | -1.164809339117 |
| C | 4.530197240815 | -0.556675768478 | 0.372271220941 |
| H | 4.529584836733 | 0.328588863046 | 1.020164377714 |
| H | 4.536355593549 | -1.451238284592 | 1.006587923129 |
| O | 5.702537497731 | -0.545433716112 | -0.510573687792 |
| C | 7.130097767086 | -0.551947422677 | -0.013755357873 |
| H | 7.335099075323 | 0.338651446537 | 0.587899022811 |
| H | 7.334422109693 | -1.456787363336 | 0.566469839980 |
| H | 7.719157241582 | -0.541298016867 | -0.927632782815 |

transCydiol_TS1-F3500

($E_F$ = -773.87511743 a.u.; $G_F$ = -773.57557655 a.u.)

0 1

| | | | |
|---|---|---|---|
| C | -1.095487674984 | -0.410227789141 | 0.542758208182 |
| C | 1.095483540487 | -0.409950380200 | -0.542939656127 |
| C | 0.836729952668 | 0.854587267681 | -1.311721768150 |
| C | 0.652330505686 | 2.084417886318 | -0.402239698655 |
| C | -0.652377877023 | 2.084216325098 | 0.403263889119 |
| C | -0.836762840293 | 0.853944282899 | 1.312151528508 |
| H | -0.989565317319 | -1.351400873645 | 1.080920500030 |
| H | 1.684404785503 | 1.041576640716 | -1.991084563728 |
| H | -0.054413140759 | 0.706682399939 | -1.931086492016 |
| H | 0.685544025439 | 2.987655033167 | -1.019849428550 |
| H | 1.507250677209 | 2.136492691456 | 0.280272825874 |
| H | -0.685601300155 | 2.987154048429 | 1.021310875835 |
| H | -1.507299181974 | 2.136611339004 | -0.279222687181 |
| H | -1.684450615341 | 1.040588441896 | 1.991593559307 |
| H | 0.054376329053 | 0.705762013497 | 1.931454998431 |
| O | -2.316569151257 | -0.342443475331 | -0.211724426551 |
| C | -3.564172258071 | -0.815686419515 | 0.453007007811 |
| H | -3.576818142809 | -0.455062957093 | 1.485439550348 |
| H | -3.562625894863 | -1.910007203361 | 0.464951852850 |
| C | -4.902587103691 | -0.320900773140 | -0.247331171543 |
| H | -4.910026646404 | 0.776392351552 | -0.258538639104 |
| H | -4.918032674602 | -0.677917557595 | -1.284776405764 |
| O | -6.049804959724 | -0.835391730488 | 0.491103458785 |

| | | | |
|---|---|---|---|
| C | -7.458593154199 | -0.540766743724 | 0.087914025343 |
| H | -7.656681468075 | 0.535681642850 | 0.119120552586 |
| H | -7.667879951802 | -0.930999886408 | -0.913475135580 |
| H | -8.062196189925 | -1.058637698303 | 0.829984248611 |
| H | 0.989572015236 | -1.350865487613 | -1.081556275132 |
| O | 2.316568600373 | -0.342518973819 | 0.211573149255 |
| C | 3.564184436905 | -0.815343895578 | -0.453427710389 |
| H | 3.562651351264 | -1.909652555010 | -0.466036567290 |
| H | 3.576836490299 | -0.454095880484 | -1.485636218566 |
| C | 4.902588158475 | -0.320969048724 | 0.247215590891 |
| H | 4.910007335675 | 0.776311257880 | 0.259103695352 |
| H | 4.918041464611 | -0.678627244468 | 1.284433398211 |
| O | 6.049815116297 | -0.834977566876 | -0.491534431279 |
| C | 7.458598460682 | -0.540576083767 | -0.088168897229 |
| H | 7.656663861519 | 0.535890480962 | -0.118692729216 |
| H | 7.667898274397 | -0.931437559636 | 0.912963869674 |
| H | 8.062209161477 | -1.057956319442 | -0.830567882891 |

transCydiol-F3500

($E_F$ = -773.91489982 a.u.; $G_F$ = -773.60973186 a.u.)

0 1

| | | | |
|---|---|---|---|
| C | -0.622932455391 | -0.460856238233 | 0.504847033538 |
| C | 0.622932294621 | -0.460589081572 | -0.505064540796 |
| C | 0.522736374987 | 0.790857477859 | -1.380191904503 |
| C | 0.556931800086 | 2.068917118287 | -0.528918092680 |
| C | -0.556973589683 | 2.068642289105 | 0.529984880862 |

| | | | |
|---|---|---|---|
| C | -0.522764114817 | 0.790149236108 | 1.380608457676 |
| H | -0.574230881305 | -1.368570554192 | 1.116669104241 |
| H | 1.316315386476 | 0.799999235171 | -2.131839519240 |
| H | -0.427356006952 | 0.727753095403 | -1.919723328752 |
| H | 0.464522409481 | 2.947626694398 | -1.174543459519 |
| H | 1.529628904470 | 2.137448805285 | -0.030210819001 |
| H | -0.464572013375 | 2.947023269660 | 1.176058447488 |
| H | -1.529671995330 | 2.137420824991 | 0.031313424969 |
| H | -1.316347691904 | 0.798895647071 | 2.132255959352 |
| H | 0.427325840168 | 0.726787972099 | 1.920114214088 |
| O | -1.902432336917 | -0.490683531862 | -0.261819753903 |
| C | -3.150033224504 | -0.553609578506 | 0.516187613615 |
| H | -3.223624563290 | 0.312976428960 | 1.179830255478 |
| H | -3.147171875314 | -1.457830340115 | 1.136370778877 |
| C | -4.472083297308 | -0.562729405879 | -0.366383866273 |
| H | -4.472703177687 | 0.325600512417 | -1.011078859748 |
| H | -4.477366519311 | -1.454166404478 | -1.006085761603 |
| O | -5.633434490041 | -0.557166330340 | 0.516837789943 |
| C | -7.034711502157 | -0.560999356107 | -0.000084565810 |
| H | -7.231692859166 | 0.331811988776 | -0.602806916865 |
| H | -7.231190398789 | -1.461637553640 | -0.591171458102 |
| H | -7.650356003151 | -0.555395870382 | 0.896754321909 |
| H | 0.574247613600 | -1.367995472546 | -1.117344326051 |
| O | 1.902435504466 | -0.490773408827 | 0.261581828469 |
| C | 3.150034050281 | -0.553307053914 | -0.516460007540 |
| H | 3.147195254466 | -1.457242758900 | -1.137055624090 |

| | | | |
|---|---|---|---|
| H | 3.223598537138 | 0.313582937382 | -1.179705787688 |
| C | 4.472087754252 | -0.562794312516 | 0.366100401933 |
| H | 4.472689192733 | 0.325243135908 | 1.011194531490 |
| H | 4.477393330316 | -1.454517543516 | 1.005399167095 |
| O | 5.633436484947 | -0.556806579177 | -0.517120089802 |
| C | 7.034714604156 | -0.560841718388 | -0.000203516050 |
| H | 7.231683177737 | 0.331710871183 | 0.602903666675 |
| H | 7.231208745023 | -1.461731203535 | 0.590492644671 |
| H | 7.650357208128 | -0.554840990644 | -0.897040221278 |

transCydiol_Int1-F3500

($E_F$ = -774.21441621 a.u.; $G_F$ = -773.92260216 a.u.)

0 1

| | | | |
|---|---|---|---|
| C | -3.402313054657 | -0.039586339567 | -0.229816028925 |
| C | 3.402306614679 | -0.038646224538 | 0.229907021885 |
| C | 2.038852882836 | 0.004601652809 | -0.512403161952 |
| C | 0.704786056708 | 0.018823195506 | 0.381376206761 |
| C | -0.704816716199 | 0.016915039768 | -0.381458448296 |
| C | -2.038875439348 | 0.006336298677 | 0.512365224422 |
| H | -3.488664667636 | -0.628569962867 | -1.142536167353 |
| H | 2.033831604946 | 0.885410636527 | -1.165080153237 |
| H | 1.975833025626 | -0.862328106678 | -1.189869660689 |
| H | 0.743359491506 | 0.898316715396 | 1.032324452983 |
| H | 0.732995081921 | -0.854390530345 | 1.042836938722 |
| H | -0.743616584083 | 0.893462363260 | -1.036359632524 |
| H | -0.732791078865 | -0.859275722023 | -1.038981262005 |

| | | | |
|---|---|---|---|
| H | -2.033732208503 | 0.889712811958 | 1.161583354305 |
| H | -1.975988267770 | -0.857916073427 | 1.193253067079 |
| O | -4.596492440123 | 0.083854006906 | 0.508042643347 |
| C | -5.881382434288 | -0.076490729791 | -0.213308753710 |
| H | -5.909870453911 | -1.075096718041 | -0.660712953420 |
| H | -5.927413996039 | 0.666940201857 | -1.013819132807 |
| C | -7.167763899537 | 0.095102846522 | 0.702669969708 |
| H | -7.132960049222 | -0.646813071353 | 1.510429989877 |
| H | -7.154790171113 | 1.096260315894 | 1.151443807667 |
| O | -8.360812067213 | -0.088158320593 | -0.113603561994 |
| C | -9.740214997644 | 0.018175609974 | 0.452358871198 |
| H | -9.903516407577 | -0.737748650304 | 1.227371626724 |
| H | -9.918288620225 | 1.018153406426 | 0.861023801387 |
| H | -10.392514298190 | -0.164839841243 | -0.398642304193 |
| H | 3.488935055767 | -0.625427217246 | 1.143984898829 |
| O | 4.596452394006 | 0.083726712344 | -0.508173124789 |
| C | 5.881356485919 | -0.077338939978 | 0.212994636401 |
| H | 5.926907968608 | 0.664496470076 | 1.015017659980 |
| H | 5.910319373072 | -1.076837645374 | 0.658357729088 |
| C | 7.167751848347 | 0.096679355230 | -0.702521704463 |
| H | 7.132239215636 | -0.642175898151 | -1.513077619652 |
| H | 7.155528285708 | 1.099544350212 | -1.147527711793 |
| O | 8.360755328943 | -0.090561717958 | 0.112913493604 |
| C | 9.740192335542 | 0.018249481473 | -0.452505069333 |
| H | 9.903257038361 | -0.733686402950 | -1.231466093301 |
| H | 9.918583423139 | 1.020310208507 | -0.855940632354 |

| | | | |
|---|---|---|---|
| H | 10.392431262385 | -0.169418046080 | 0.397546173599 |

transCydiol_TS1-F3000

($E_F$ = -773.70440642 a.u.; $G_F$ = -773.40471209 a.u.)

0 1

| | | | |
|---|---|---|---|
| C | -1.115341441689 | -0.398868283979 | 0.554228863411 |
| C | 1.115119085878 | -0.399348741753 | -0.553891596470 |
| C | 0.834645578319 | 0.863219198683 | -1.316946861685 |
| C | 0.650963390331 | 2.092013606221 | -0.405800430425 |
| C | -0.650738642395 | 2.092329137227 | 0.404693731893 |
| C | -0.834577529991 | 0.864074217962 | 1.316541468841 |
| H | -1.000828422202 | -1.343759133747 | 1.084045082607 |
| H | 1.674304743878 | 1.057748084251 | -2.004937994753 |
| H | -0.060943714064 | 0.708698261298 | -1.927954543190 |
| H | 0.680958998273 | 2.995614380824 | -1.023146879189 |
| H | 1.508448829752 | 2.145434098823 | 0.273415245916 |
| H | -0.680614722059 | 2.996279899784 | 1.021531958472 |
| H | -1.508223769699 | 2.145484807287 | -0.274543161874 |
| H | -1.674222794125 | 1.059077069436 | 2.004417790131 |
| H | 0.061023945529 | 0.709748613659 | 1.927579098092 |
| O | -2.326049023742 | -0.323464258664 | -0.195183901998 |
| C | -3.553745807389 | -0.840953801878 | 0.448355481355 |
| H | -3.568722097778 | -0.532190578291 | 1.497870515192 |
| H | -3.545880348596 | -1.934970618235 | 0.406980235250 |
| C | -4.878119099388 | -0.317724042248 | -0.228027841610 |
| H | -4.889928033937 | 0.779308346989 | -0.185961326474 |

| | | | |
|---|---|---|---|
| H | -4.892439558622 | -0.622545046408 | -1.282566206193 |
| O | -6.013489129099 | -0.872298997188 | 0.483840082600 |
| C | -7.398958342295 | -0.545538640874 | 0.081912102975 |
| H | -7.589354555428 | 0.530380187792 | 0.165488371439 |
| H | -7.600205294257 | -0.877846419780 | -0.942699801659 |
| H | -8.026802186955 | -1.092259397500 | 0.782713824819 |
| H | 1.000466280425 | -1.344498264322 | -1.083238065362 |
| O | 2.325835999108 | -0.323958592711 | 0.195648599467 |
| C | 3.553770090621 | -0.839447937006 | -0.449063548293 |
| H | 3.545761813036 | -1.933585510887 | -0.411285345443 |
| H | 3.569207269370 | -0.527289921123 | -1.497552343314 |
| C | 4.877994657765 | -0.318565115990 | 0.229365735712 |
| H | 4.889440940916 | 0.778614620877 | 0.191786435576 |
| H | 4.892563419545 | -0.627694193230 | 1.282624575177 |
| O | 6.013457600701 | -0.869819731244 | -0.484931100998 |
| C | 7.398889086837 | -0.544917125204 | -0.081393001425 |
| H | 7.588999049335 | 0.531490938593 | -0.158992886471 |
| H | 7.600356771735 | -0.882875272239 | 0.941315838134 |
| H | 8.026785962337 | -1.087545845243 | -0.785312200179 |

transCydiol-F3000

($E_F$ = -773.75424071 a.u.; $G_F$ = -773.44825176 a.u.)

0 1

| | | | |
|---|---|---|---|
| C | -0.611658230625 | -0.452691868837 | 0.508447085158 |
| C | 0.611663185663 | -0.452587615726 | -0.508542939921 |
| C | 0.510166686261 | 0.799766937194 | -1.384416049173 |

| | | | |
|---|---|---|---|
| C | 0.552625182304 | 2.077789223533 | -0.533776941873 |
| C | -0.552928705068 | 2.077691526720 | 0.533826534785 |
| C | -0.510316371299 | 0.799625039568 | 1.384391923283 |
| H | -0.554697430254 | -1.359086820244 | 1.122576102457 |
| H | 1.299665282687 | 0.806189473693 | -2.140302441594 |
| H | -0.442453389326 | 0.738966743381 | -1.919263113545 |
| H | 0.456077866735 | 2.956746383299 | -1.178466511172 |
| H | 1.529207334092 | 2.145746484538 | -0.042483171273 |
| H | -0.456487836634 | 2.956623003689 | 1.178567035729 |
| H | -1.529519038277 | 2.145559080294 | 0.042536630748 |
| H | -1.299817384402 | 0.805907902857 | 2.140277001599 |
| H | 0.442309916210 | 0.738910263353 | 1.919237725708 |
| O | -1.880981082126 | -0.487634575378 | -0.253798316592 |
| C | -3.114429570039 | -0.564405283205 | 0.526201646992 |
| H | -3.193990792671 | 0.294211186926 | 1.200067959835 |
| H | -3.108437230427 | -1.475360163130 | 1.137433980761 |
| C | -4.418348280477 | -0.568458263937 | -0.359679136564 |
| H | -4.420020194935 | 0.326142928712 | -0.996549405824 |
| H | -4.421159165675 | -1.453544295490 | -1.009026080022 |
| O | -5.571107784943 | -0.573839093962 | 0.522080648065 |
| C | -6.947217640481 | -0.571966393377 | -0.017417702661 |
| H | -7.134430083065 | 0.326553350934 | -0.616268036976 |
| H | -7.133927281301 | -1.465124389084 | -0.624334125134 |
| H | -7.589505513563 | -0.576154609137 | 0.861098313201 |
| H | 0.554813605187 | -1.358954618828 | -1.122723376569 |
| O | 1.880991169195 | -0.487415959482 | 0.253699015986 |

| | | | |
|---|---|---|---|
| C | 3.114448520603 | -0.563994544852 | -0.526305696593 |
| H | 3.108573561165 | -1.474924569435 | -1.137575355377 |
| H | 3.193898091304 | 0.294659572687 | -1.200136596900 |
| C | 4.418368289397 | -0.567917293571 | 0.359573457980 |
| H | 4.419934888438 | 0.326666659356 | 0.996468897907 |
| H | 4.421283545515 | -1.453021424673 | 1.008895980768 |
| O | 5.571128120090 | -0.573138198510 | -0.522188149428 |
| C | 6.947237894249 | -0.571094891903 | 0.017311141637 |
| H | 7.134338230732 | 0.327451568756 | 0.616165085417 |
| H | 7.134059198507 | -1.464234400081 | 0.624228969065 |
| H | 7.589526057207 | -0.575202085372 | -0.861209562211 |

transCydiol_Int1-F3000

($E_F$ = -773.99178846 a.u.; $G_F$ = -773.69873877 a.u.)

0 1

| | | | |
|---|---|---|---|
| C | -3.359982414398 | -0.037455694266 | -0.231825436934 |
| C | 3.359943672957 | -0.037666892589 | 0.231808304141 |
| C | 2.012472293199 | 0.008129223578 | -0.514705531059 |
| C | 0.696741541776 | 0.019935587914 | 0.382042533386 |
| C | -0.696783286979 | 0.020361717373 | -0.381987111045 |
| C | -2.012512829509 | 0.007442473428 | 0.514745188635 |
| H | -3.444383991990 | -0.626625578774 | -1.144740674140 |
| H | 2.008306846723 | 0.891509589018 | -1.164438572708 |
| H | 1.948779491657 | -0.856528722776 | -1.195895277062 |
| H | 0.736280673832 | 0.897476587933 | 1.036060457032 |
| H | 0.725430263294 | -0.855397343471 | 1.041251625668 |

| | | | |
|---|---|---|---|
| H | -0.736354806175 | 0.898675209915 | -1.034964437658 |
| H | -0.725439551416 | -0.854190179217 | -1.042232274651 |
| H | -2.008356424747 | 0.890030915489 | 1.165552868995 |
| H | -1.948810404295 | -0.858041026837 | 1.194882103572 |
| O | -4.545882361975 | 0.084519738699 | 0.506070230779 |
| C | -5.816602722650 | -0.074681736637 | -0.219006404591 |
| H | -5.847211979613 | -1.072737290469 | -0.668766984851 |
| H | -5.864005027372 | 0.669486937026 | -1.019384400617 |
| C | -7.085550213384 | 0.096200931801 | 0.698718488356 |
| H | -7.049910254362 | -0.645524878152 | 1.507348336767 |
| H | -7.073587264027 | 1.097500700573 | 1.148481707893 |
| O | -8.269077072481 | -0.088150884392 | -0.117659983009 |
| C | -9.623060084710 | 0.022252185430 | 0.467654943956 |
| H | -9.776303296465 | -0.730322698990 | 1.249156160937 |
| H | -9.792753655851 | 1.023154778928 | 0.879876973155 |
| H | -10.300112478625 | -0.159375557288 | -0.364688441528 |
| H | 3.444379859464 | -0.628064832716 | 1.143921593411 |
| O | 4.545837405914 | 0.085367202258 | -0.505918609356 |
| C | 5.816563635147 | -0.074885308036 | 0.218912369116 |
| H | 5.863967369799 | 0.668090493680 | 1.020387354214 |
| H | 5.847183584237 | -1.073599479148 | 0.667187083274 |
| C | 7.085504833888 | 0.097364343618 | -0.698554665901 |
| H | 7.049944071864 | -0.643255503951 | -1.508192734192 |
| H | 7.073453621945 | 1.099271305104 | -1.146946087870 |
| O | 8.269039189368 | -0.087991609744 | 0.117590071402 |
| C | 9.623016682900 | 0.023116173120 | -0.467625130721 |

| | | | |
|---|---|---|---|
| H | 9.776245614859 | -0.728531460524 | -1.250021356073 |
| H | 9.792716673379 | 1.024506091446 | -0.878666194847 |
| H | 10.300072332771 | -0.159508276239 | 0.364478473062 |

transCydiol_TS1-F2500

($E_F$ = -773.53498853 a.u.; $G_F$ = -773.23556733 a.u.)

0 1

| | | | |
|---|---|---|---|
| C | -1.136945867788 | -0.381663244128 | 0.568590128734 |
| C | 1.137270649903 | -0.380484319664 | -0.569415402395 |
| C | 0.825555568899 | 0.878700737524 | -1.324452570903 |
| C | 0.646316894574 | 2.106349665280 | -0.410620593384 |
| C | -0.646855571282 | 2.105562872845 | 0.413665046566 |
| C | -0.825809724331 | 0.876478259202 | 1.325612024601 |
| H | -1.016654050461 | -1.332451170642 | 1.086095625469 |
| H | 1.650653288298 | 1.083050404801 | -2.027861246837 |
| H | -0.078991366268 | 0.714476671699 | -1.919244189679 |
| H | 0.667381728633 | 3.010217381238 | -1.028079701027 |
| H | 1.510878115492 | 2.162872368844 | 0.259293896664 |
| H | -0.668155852311 | 3.008490449120 | 1.032491659194 |
| H | -1.511415467830 | 2.162870904058 | -0.256184451764 |
| H | -1.651067615730 | 1.079512326010 | 2.029216967861 |
| H | 0.078680249185 | 0.711682053595 | 1.920334740379 |
| O | -2.339645784902 | -0.289511920292 | -0.171736553635 |
| C | -3.544026327836 | -0.882525648732 | 0.424942621707 |
| H | -3.559955538728 | -0.674281158484 | 1.499206139764 |
| H | -3.525905659673 | -1.968002257678 | 0.280775569319 |

| | | | |
|---|---|---|---|
| C | -4.857884400865 | -0.306732212670 | -0.200272460243 |
| H | -4.876658069680 | 0.781735859876 | -0.054583793380 |
| H | -4.869413944218 | -0.508978560328 | -1.279701976916 |
| O | -5.981919082300 | -0.932856763014 | 0.453191528733 |
| C | -7.345462444629 | -0.555070377383 | 0.071265204765 |
| H | -7.527578069817 | 0.510148141691 | 0.257117220409 |
| H | -7.537461915142 | -0.779495317786 | -0.984704551024 |
| H | -7.996711795485 | -1.157580847168 | 0.702043611246 |
| H | 1.017215490209 | -1.330540053275 | -1.088291743454 |
| O | 2.339969382705 | -0.288865685469 | 0.170813656609 |
| C | 3.544096481429 | -0.883335612013 | -0.424898342569 |
| H | 3.525972068157 | -1.968478253767 | -0.278226301813 |
| H | 3.559717355924 | -0.677520112876 | -1.499638970227 |
| C | 4.858099025936 | -0.306130649132 | 0.198757489168 |
| H | 4.877034893613 | 0.781963847683 | 0.050211982909 |
| H | 4.869580350998 | -0.505542319218 | 1.278731259410 |
| O | 5.982037958326 | -0.934151309745 | -0.453045864886 |
| C | 7.345623266603 | -0.555322052657 | -0.072269494664 |
| H | 7.527836476119 | 0.509320420603 | -0.261411457748 |
| H | 7.537595308624 | -0.776511741459 | 0.984407451162 |
| H | 7.996812995610 | -1.159850776522 | -0.701198158055 |

transCydiol_Int1-F2500

($E_F$ = -773.77172343 a.u.; $G_F$ = -773.47767887 a.u.)

0 1

| | | | |
|---|---|---|---|
| C | -3.320975435747 | -0.036326544645 | -0.235941406384 |

| | | | |
|---|---|---|---|
| C | 3.321021612347 | -0.035665878794 | 0.235914688335 |
| C | 1.988853571711 | 0.009467783086 | -0.515934066728 |
| C | 0.689273912602 | 0.022815978411 | 0.382959681875 |
| C | -0.689228765989 | 0.022055168756 | -0.383119029795 |
| C | -1.988802733990 | 0.010293640423 | 0.515809357468 |
| H | -3.402859801811 | -0.629092196624 | -1.146902489067 |
| H | 1.986067948441 | 0.892603778896 | -1.166439341341 |
| H | 1.925036369055 | -0.855985630225 | -1.196868072796 |
| H | 0.729337229777 | 0.901390339883 | 1.035967125821 |
| H | 0.718084998192 | -0.851712954410 | 1.043714006590 |
| H | -0.729352614600 | 0.899411947574 | -1.037763331499 |
| H | -0.717982523226 | -0.853707310550 | -1.042245136039 |
| H | -1.986069821617 | 0.894646715654 | 1.164668650633 |
| H | -1.924924630969 | -0.853885639088 | 1.198361862593 |
| O | -4.499458385776 | 0.086510006087 | 0.501045238408 |
| C | -5.756894318363 | -0.072860261285 | -0.227970944305 |
| H | -5.788973175622 | -1.070943176678 | -0.678671246122 |
| H | -5.805470289426 | 0.671233701151 | -1.028888752846 |
| C | -7.009428265343 | 0.098218542142 | 0.691951417570 |
| H | -6.972379967390 | -0.642966523631 | 1.501666905772 |
| H | -6.997474692908 | 1.099861256599 | 1.142085657160 |
| O | -8.185407312112 | -0.087019270977 | -0.122916476136 |
| C | -9.514593943727 | 0.027971716425 | 0.483653604315 |
| H | -9.656422735044 | -0.721556242059 | 1.271279326506 |
| H | -9.674313327167 | 1.029802773254 | 0.899610724996 |
| H | -10.216295718211 | -0.151652996784 | -0.328980739255 |

| | | | |
|---|---|---|---|
| H | 3.402936158349 | -0.626785881249 | 1.147955665659 |
| O | 4.499505088759 | 0.085916630006 | -0.501298175866 |
| C | 5.756944676917 | -0.071985795562 | 0.228055155566 |
| H | 5.805475958892 | 0.673660823227 | 1.027565928061 |
| H | 5.789074649364 | -1.069224222110 | 0.680679473003 |
| C | 7.009475809516 | 0.097416407341 | -0.692190175708 |
| H | 6.972661743741 | -0.645506956279 | -1.500294287848 |
| H | 6.997279396051 | 1.098065171789 | -1.144476196208 |
| O | 8.185463243380 | -0.085736312623 | 0.123103920430 |
| C | 9.514646518204 | 0.027948904874 | -0.483691778959 |
| H | 9.656649319551 | -0.723592134023 | -1.269353553634 |
| H | 9.674176630640 | 1.028717091597 | -0.902254596286 |
| H | 10.216361494294 | -0.149412250869 | 0.329436978103 |

transCydiol-F2500

($E_F$ = -773.59551785 a.u.; $G_F$ = -773.28888056 a.u.)

0 1

| | | | |
|---|---|---|---|
| C | -0.601210865125 | -0.442966507251 | 0.511783892209 |
| C | 0.601218713162 | -0.442613561645 | -0.512083130339 |
| C | 0.499585264191 | 0.811320839657 | -1.387568857028 |
| C | 0.549464058347 | 2.088569018542 | -0.536464661322 |
| C | -0.549195302355 | 2.088204183262 | 0.538181316563 |
| C | -0.499484001349 | 0.810267239691 | 1.388265102369 |
| H | -0.536094784293 | -1.348481100857 | 1.127341708106 |
| H | 1.286053712663 | 0.815591458344 | -2.146580623636 |
| H | -0.454872325671 | 0.753536913799 | -1.919066540887 |

| | | | |
|---|---|---|---|
| H | 0.449995351555 | 2.968323849948 | -1.179629010912 |
| H | 1.529225014185 | 2.155099488485 | -0.051215450112 |
| H | -0.449618937638 | 2.967428438881 | 1.182054372153 |
| H | -1.528946863418 | 2.155247202462 | 0.052984453907 |
| H | -1.285991974558 | 0.814018324286 | 2.147239266574 |
| H | 0.454939712902 | 0.751966920306 | 1.919766692705 |
| O | -1.861234445666 | -0.482408644690 | -0.246430271163 |
| C | -3.081369373151 | -0.580673751893 | 0.534743272999 |
| H | -3.167412763317 | 0.263790282803 | 1.226022690278 |
| H | -3.071480341220 | -1.503646570752 | 1.128543269500 |
| C | -4.367578177879 | -0.572535980035 | -0.355881625414 |
| H | -4.369859701926 | 0.334149168760 | -0.976265187659 |
| H | -4.365517692214 | -1.444921942925 | -1.022963884402 |
| O | -5.514732463008 | -0.598248536509 | 0.521278250083 |
| C | -6.865505443552 | -0.584546418498 | -0.044388172358 |
| H | -7.040775926016 | 0.325969420042 | -0.630016841326 |
| H | -7.039860549827 | -1.463578607749 | -0.676477187724 |
| H | -7.535615363671 | -0.607236042207 | 0.813338392776 |
| H | 0.536022662409 | -1.347630937617 | -1.128363940746 |
| O | 1.861240213136 | -0.482792476701 | 0.246099699879 |
| C | 3.081392591934 | -0.580010150090 | -0.535173146719 |
| H | 3.071433280344 | -1.502074895303 | -1.130367261546 |
| H | 3.167542816340 | 0.265486438557 | -1.225164744294 |
| C | 4.367579702417 | -0.573338019824 | 0.355487011043 |
| H | 4.369886692009 | 0.332356689123 | 0.977298421822 |
| H | 4.365474270197 | -1.446770931048 | 1.021181548111 |

| | | | |
|---|---|---|---|
| O | 5.514746640904 | -0.597708000936 | -0.521688463070 |
| C | 6.865514823611 | -0.585009023856 | 0.044006541785 |
| H | 7.040845412139 | 0.324545304970 | 0.631091138789 |
| H | 7.039802570006 | -1.465058709894 | 0.674679664317 |
| H | 7.535630284215 | -0.606369727989 | -0.813741651550 |

transCydiol_TS1-F2000

($E_F$ = -773.36681842 a.u.; $G_F$ = -773.06897582 a.u.)

0 1

| | | | |
|---|---|---|---|
| C | -1.158026905571 | -0.338990330580 | 0.595828857541 |
| C | 1.157480586037 | -0.339806686702 | -0.595141184066 |
| C | 0.784657977424 | 0.904560458293 | -1.346828872101 |
| C | 0.629150730290 | 2.140576851360 | -0.439468463995 |
| C | -0.628382390030 | 2.141080870491 | 0.438605388890 |
| C | -0.784388261655 | 0.905637571809 | 1.346676453582 |
| H | -1.050532942742 | -1.298429381278 | 1.098430223729 |
| H | 1.569695639063 | 1.117239780221 | -2.092708444396 |
| H | -0.145268715280 | 0.716524592391 | -1.892986449012 |
| H | 0.616136654350 | 3.037559820423 | -1.067375975165 |
| H | 1.520475650210 | 2.213911459167 | 0.192408324459 |
| H | -0.614966056616 | 3.038386962504 | 1.066041822973 |
| H | -1.519693432341 | 2.214489146846 | -0.193281899206 |
| H | -1.569180499184 | 1.119133902870 | 2.092568095324 |
| H | 0.145556986685 | 0.717395977276 | 1.892720460697 |
| O | -2.358846352879 | -0.192766090280 | -0.116100655287 |
| C | -3.515309026162 | -0.990529611229 | 0.268440719796 |

| | | | |
|---|---|---|---|
| H | -3.508010633998 | -1.162279607870 | 1.349254430837 |
| H | -3.483979741509 | -1.959876111979 | -0.240041947880 |
| C | -4.834152387160 | -0.256708518894 | -0.108061674190 |
| H | -4.865624592603 | 0.711505168482 | 0.410581204491 |
| H | -4.849404387717 | -0.065113212016 | -1.189910984346 |
| O | -5.939593566956 | -1.085662177005 | 0.283172430365 |
| C | -7.285568275704 | -0.587001681893 | 0.052520356263 |
| H | -7.463111957495 | 0.346001223076 | 0.601975639538 |
| H | -7.469338782672 | -0.417689942252 | -1.015856421886 |
| H | -7.954930345966 | -1.362823097005 | 0.421248984462 |
| H | 1.049638830376 | -1.299470210010 | -1.097268760573 |
| O | 2.358297986070 | -0.193985933426 | 0.117108596286 |
| C | 3.515271362303 | -0.989635520239 | -0.270429166338 |
| H | 3.483969528758 | -1.961331922310 | 0.233590851606 |
| H | 3.508573284548 | -1.156483732228 | -1.352025901445 |
| C | 4.833793197130 | -0.257256122839 | 0.109896258174 |
| H | 4.865157782238 | 0.713418013364 | -0.404147753215 |
| H | 4.848813283534 | -0.070800981957 | 1.192654182480 |
| O | 5.939490740174 | -1.084120684946 | -0.285087805066 |
| C | 7.285335507978 | -0.586278007911 | -0.051972371374 |
| H | 7.462709664967 | 0.349406267027 | -0.596928901245 |
| H | 7.469024922089 | -0.422061539591 | 1.017224894196 |
| H | 7.954887939985 | -1.360176961213 | -0.424398544849 |

transCydiol_Int1-F2000

($E_F$ = -773.55404079 a.u.; $G_F$ = -773.25921514 a.u.)

0 1

| | | | |
|---|---|---|---|
| C | -3.283966762826 | -0.037167279191 | -0.254026689266 |
| C | 3.283876373699 | -0.036465141385 | 0.254059138727 |
| C | 1.969245071889 | 0.009030246159 | -0.508646951815 |
| C | 0.680657384001 | 0.022718836157 | 0.386950981119 |
| C | -0.680752857134 | 0.021969426803 | -0.387020858107 |
| C | -1.969339969386 | 0.009761411842 | 0.508598910275 |
| H | -3.359795805513 | -0.632681654243 | -1.163867118860 |
| H | 1.970691035911 | 0.892834973214 | -1.158586832243 |
| H | 1.908326777399 | -0.856346917782 | -1.190593625433 |
| H | 0.718495555272 | 0.901417565622 | 1.040259191015 |
| H | 0.706693165631 | -0.851993728254 | 1.047982354426 |
| H | -0.718668862635 | 0.899498468338 | -1.041894109700 |
| H | -0.706710802118 | -0.853922139289 | -1.046491746683 |
| H | -1.970853389735 | 0.894703514265 | 1.156985330995 |
| H | -1.908357378896 | -0.854412493910 | 1.192061115797 |
| O | -4.458377156931 | 0.085952540497 | 0.477539292327 |
| C | -5.700816110978 | -0.072205291793 | -0.260269085229 |
| H | -5.732548678446 | -1.069451409631 | -0.713842868584 |
| H | -5.747642110309 | 0.673596062821 | -1.060194856758 |
| C | -6.940512390931 | 0.097327540213 | 0.658950042590 |
| H | -6.903903719910 | -0.645258084960 | 1.467995324831 |
| H | -6.928840701625 | 1.098218751770 | 1.111785415895 |
| O | -8.108393749902 | -0.086180597146 | -0.156832343268 |
| C | -9.414777386822 | 0.031901240213 | 0.469275434290 |
| H | -9.546392300631 | -0.717616216023 | 1.259630274713 |

| | | | |
|---|---|---|---|
| H | -9.564130988220 | 1.033186974990 | 0.892134744574 |
| H | -10.138806826795 | -0.142222196169 | -0.325259545807 |
| H | 3.359747302077 | -0.630337921117 | 1.164966488340 |
| O | 4.458276908173 | 0.085453864276 | -0.477723246344 |
| C | 5.700727177968 | -0.071559594052 | 0.260324505363 |
| H | 5.747459178772 | 0.675342320610 | 1.059244978763 |
| H | 5.732569592485 | -1.068193063403 | 0.715260261192 |
| C | 6.940417939010 | 0.096846411038 | -0.659114693087 |
| H | 6.903706397479 | -0.646617986010 | -1.467350485212 |
| H | 6.928848174403 | 1.097250683099 | -1.113039446460 |
| O | 8.108297280516 | -0.085894266365 | 0.156836743217 |
| C | 9.414684284619 | 0.031727341372 | -0.469343563460 |
| H | 9.546298993650 | -0.718358746731 | -1.259183009961 |
| H | 9.564039618022 | 1.032733148577 | -0.892905691275 |
| H | 10.138710975103 | -0.141843903655 | 0.325333457551 |

transCydiol-F2000

($E_F$ = -773.43861637 a.u.; $G_F$ = -773.13140121 a.u.)

0 1

| | | | |
|---|---|---|---|
| C | -0.594070663638 | -0.433405745238 | 0.512021758435 |
| C | 0.593907273887 | -0.432977262635 | -0.512665675407 |
| C | 0.497385839400 | 0.822451050224 | -1.388173334338 |
| C | 0.549417456107 | 2.098954116440 | -0.536361970799 |
| C | -0.549054206933 | 2.098505598252 | 0.538413360189 |
| C | -0.497326644655 | 0.821077587203 | 1.388859690054 |
| H | -0.523669110337 | -1.337871072811 | 1.129361643024 |

| | | | |
|---|---|---|---|
| H | 1.285596532430 | 0.824588199006 | -2.145381605753 |
| H | -0.455666091513 | 0.767715985760 | -1.922206275941 |
| H | 0.451109634209 | 2.979457959795 | -1.178687157615 |
| H | 1.529313629993 | 2.163949730405 | -0.051069467665 |
| H | -0.450539067349 | 2.978294030058 | 1.181686716887 |
| H | -1.528934990548 | 2.164259498200 | 0.053191670271 |
| H | -1.285572951762 | 0.822572781971 | 2.146032899228 |
| H | 0.455687374559 | 0.765580347090 | 1.922880559938 |
| O | -1.841681817909 | -0.478075953860 | -0.248691697259 |
| C | -3.053045368423 | -0.602171110346 | 0.526750859271 |
| H | -3.150943491704 | 0.226064007623 | 1.236324556625 |
| H | -3.040181973975 | -1.538151201064 | 1.100596248483 |
| C | -4.316987491267 | -0.582063343178 | -0.376478785607 |
| H | -4.316874782490 | 0.337531402509 | -0.978317096367 |
| H | -4.303511603646 | -1.440027169491 | -1.062538778133 |
| O | -5.465858851886 | -0.631365222771 | 0.486684798369 |
| C | -6.787472800171 | -0.604497785662 | -0.115176347953 |
| H | -6.946552981370 | 0.319193666489 | -0.685906403160 |
| H | -6.942849230473 | -1.467314934638 | -0.775135937927 |
| H | -7.490778005468 | -0.647553417448 | 0.715236050418 |
| H | 0.523333547079 | -1.336774912066 | -1.130963347804 |
| O | 1.841514506917 | -0.478691503691 | 0.247993417856 |
| C | 3.052865128253 | -0.601977370976 | -0.527602810380 |
| H | 3.039840225390 | -1.537197552956 | -1.102699284081 |
| H | 3.150921848596 | 0.227191151505 | -1.236077350963 |
| C | 4.316802899921 | -0.583295557988 | 0.375670920103 |

| | | | |
|---|---|---|---|
| H | 4.316846386955 | 0.335503033063 | 0.978742174135 |
| H | 4.303175224000 | -1.442180939486 | 1.060589965514 |
| O | 5.465670135828 | -0.631641255919 | -0.487562184448 |
| C | 6.787287254518 | -0.605807981723 | 0.114342606581 |
| H | 6.946524480266 | 0.317097986359 | 0.686320278751 |
| H | 6.942515359877 | -1.469547728892 | 0.773147993248 |
| H | 7.490586404633 | -0.647866561424 | -0.716135226273 |

transCydiol_TS1-F1500

($E_F$ = -773.19996489 a.u.; $G_F$ = -772.90053071 a.u.)

0 1

| | | | |
|---|---|---|---|
| C | -1.187230746526 | -0.301787771311 | 0.629582798264 |
| C | 1.187476745125 | -0.300844499310 | -0.630006716527 |
| C | 0.753822931265 | 0.932226803675 | -1.365846146807 |
| C | 0.614036913948 | 2.170438341678 | -0.458176084327 |
| C | -0.614570157465 | 2.169812935521 | 0.460316905034 |
| C | -0.754015267576 | 0.930652943324 | 1.366734471040 |
| H | -1.085112898399 | -1.269184942678 | 1.116949774886 |
| H | 1.499187387688 | 1.160339431774 | -2.147469266312 |
| H | -0.194886560797 | 0.722312473454 | -1.869477192139 |
| H | 0.573390331820 | 3.064192192203 | -1.089721031798 |
| H | 1.524506070721 | 2.256057454829 | 0.143886152610 |
| H | -0.574181894082 | 3.062953558246 | 1.092745023912 |
| H | -1.525060791933 | 2.255765410478 | -0.141666606534 |
| H | -1.499507771036 | 1.157742017085 | 2.148538446442 |
| H | 0.194720772664 | 0.720534472057 | 1.870234656034 |

| | | | |
|---|---|---|---|
| O | -2.387910123830 | -0.115005543669 | -0.053283226386 |
| C | -3.494103223018 | -1.020592293575 | 0.163087902430 |
| H | -3.467083392518 | -1.416285311574 | 1.183410716376 |
| H | -3.438428693194 | -1.858723454615 | -0.539610168016 |
| C | -4.822213334455 | -0.257063331946 | -0.046398367487 |
| H | -4.871010441004 | 0.578165983795 | 0.666548948843 |
| H | -4.845416347189 | 0.162062528929 | -1.062312120670 |
| O | -5.903573509699 | -1.169810288378 | 0.157319084844 |
| C | -7.236814022499 | -0.631136797370 | 0.032620281906 |
| H | -7.415998969100 | 0.164013514268 | 0.768084168684 |
| H | -7.413832235528 | -0.232995129260 | -0.975035889092 |
| H | -7.917763314755 | -1.460408331974 | 0.220537641971 |
| H | 1.085559101902 | -1.267774139798 | -1.118337273373 |
| O | 2.388209559325 | -0.114365791719 | 0.052787763786 |
| C | 3.494129367908 | -1.020395897151 | -0.163143745195 |
| H | 3.438407602979 | -1.857999817330 | 0.540261641885 |
| H | 3.466800541987 | -1.416895433102 | -1.183204881070 |
| C | 4.822446120513 | -0.256916750468 | 0.045501942716 |
| H | 4.871301167968 | 0.577773033829 | -0.668154180653 |
| H | 4.845881046929 | 0.163038166877 | 1.061124150144 |
| O | 5.903597232745 | -1.170059793983 | -0.157651038025 |
| C | 7.236956587542 | -0.631540665827 | -0.033446360080 |
| H | 7.416271416885 | 0.162990565089 | -0.769574499823 |
| H | 7.414096443009 | -0.232618210155 | 0.973898559210 |
| H | 7.917725351648 | -1.461117631953 | -0.220730236642 |

transCydiol_Int1-F1500

($E_F$ = -773.33860086 a.u.; $G_F$ = -773.04326734 a.u.)

0 1

| | | | |
|---|---|---|---|
| C | -3.249203307697 | -0.035947384164 | -0.273075004132 |
| C | 3.249230059333 | -0.036201478756 | 0.273048949356 |
| C | 1.951527187957 | 0.009960990822 | -0.500668674381 |
| C | 0.672476106039 | 0.023136537016 | 0.391222290133 |
| C | -0.672450200278 | 0.023514428171 | -0.391184579170 |
| C | -1.951501601509 | 0.009458338601 | 0.500691253243 |
| H | -3.319405387384 | -0.633004813002 | -1.182486780259 |
| H | 1.957344250556 | 0.894945459124 | -1.149243085589 |
| H | 1.894005103961 | -0.854872433614 | -1.184152134645 |
| H | 0.707882049116 | 0.901238315395 | 1.045748452287 |
| H | 0.695289134892 | -0.852549335946 | 1.051425865697 |
| H | -0.707861253513 | 0.902255480185 | -1.044850930222 |
| H | -0.695257403836 | -0.851524582191 | -1.052244108597 |
| H | -1.957320017183 | 0.893807726422 | 1.150132520611 |
| H | -1.893979362614 | -0.856043907358 | 1.183328178981 |
| O | -4.419646740908 | 0.086968350590 | 0.453596669841 |
| C | -5.648623773871 | -0.067928468945 | -0.292081082918 |
| H | -5.680722572010 | -1.063299685766 | -0.750615883304 |
| H | -5.694302278457 | 0.681598190144 | -1.089028919371 |
| C | -6.874301041433 | 0.098441590174 | 0.629701546502 |
| H | -6.835650257679 | -0.647159145995 | 1.436410143180 |
| H | -6.861036366601 | 1.097620138467 | 1.087192628341 |
| O | -8.037676765277 | -0.081262183732 | -0.183119900573 |

| | | | |
|---|---|---|---|
| C | -9.319532067000 | 0.039067874054 | 0.467785514470 |
| H | -9.437608145334 | -0.711905482101 | 1.259799081461 |
| H | -9.456117140406 | 1.039129849692 | 0.899433434614 |
| H | -10.068037475983 | -0.128094595466 | -0.305795305272 |
| H | 3.319447480295 | -0.634193739765 | 1.181840301979 |
| O | 4.419670842782 | 0.087490931411 | -0.453493305294 |
| C | 5.648650590989 | -0.068195785961 | 0.292012188846 |
| H | 5.694297926570 | 0.680438534313 | 1.089799015961 |
| H | 5.680783756633 | -1.064078447681 | 0.749430062170 |
| C | 6.874324948954 | 0.099249384182 | -0.629580949046 |
| H | 6.835653851068 | -0.645392415302 | -1.437178239770 |
| H | 6.861077367603 | 1.098974953699 | -1.085884128784 |
| O | 8.037701425756 | -0.081438974057 | 0.183024575850 |
| C | 9.319557783224 | 0.039693773958 | -0.467728889046 |
| H | 9.437655694577 | -0.710337532699 | -1.260632718326 |
| H | 9.456122379524 | 1.040271504402 | -0.898189744456 |
| H | 10.068063180426 | -0.128367136397 | 0.305660251408 |

transCydiol-F1500

($E_F$ = -773.28345786 a.u.; $G_F$ = -772.97618395 a.u.)

0 1

| | | | |
|---|---|---|---|
| C | -0.585472310667 | -0.403415571743 | 0.514719341111 |
| C | 0.585517118508 | -0.403046336067 | -0.515026716943 |
| C | 0.492341041089 | 0.853791625931 | -1.390144188310 |
| C | 0.548195890100 | 2.129526665929 | -0.537660951452 |
| C | -0.548002794705 | 2.129152319348 | 0.539299867792 |

| | | | |
|---|---|---|---|
| C | -0.492262630181 | 0.852751415324 | 1.390797198617 |
| H | -0.507073100222 | -1.306646474379 | 1.133593352819 |
| H | 1.281228528960 | 0.853764792697 | -2.146703192712 |
| H | -0.460218506882 | 0.802303823123 | -1.925197574962 |
| H | 0.449911559724 | 3.010780183113 | -1.178971962187 |
| H | 1.529166006481 | 2.192744033445 | -0.054251390548 |
| H | -0.449646443528 | 3.009897158742 | 1.181298446480 |
| H | -1.528966399313 | 2.192831364622 | 0.055938278978 |
| H | -1.281190845518 | 0.852190607831 | 2.147314301521 |
| H | 0.460265249087 | 0.800800305732 | 1.925861340744 |
| O | -1.824091340412 | -0.453930695502 | -0.243928189387 |
| C | -3.023361502042 | -0.617586393410 | 0.528670426707 |
| H | -3.132446717903 | 0.183958383889 | 1.267146500120 |
| H | -3.001891280129 | -1.573412663718 | 1.069322904848 |
| C | -4.268510243320 | -0.576859836527 | -0.382857506586 |
| H | -4.270922260885 | 0.363806086834 | -0.951941231746 |
| H | -4.240630207847 | -1.409211925319 | -1.099982439164 |
| O | -5.418494577512 | -0.666814590214 | 0.465444173410 |
| C | -6.711439509933 | -0.618024578234 | -0.170566946452 |
| H | -6.856662905632 | 0.327617850291 | -0.709400892556 |
| H | -6.844927214220 | -1.452474860547 | -0.871592762958 |
| H | -7.446044445706 | -0.696011552874 | 0.630144633761 |
| H | 0.507082883958 | -1.305799996583 | -1.134592924535 |
| O | 1.824135963991 | -0.454198014744 | 0.243579487565 |
| C | 3.023423817296 | -0.616978311372 | -0.529176504715 |
| H | 3.001937580542 | -1.572156911838 | -1.070973403018 |

| | | | |
|---|---|---|---|
| H | 3.132558123549 | 0.185446094244 | -1.266690252667 |
| C | 4.268553238696 | -0.577377295667 | 0.382426467688 |
| H | 4.270960066873 | 0.362593558634 | 0.952662888235 |
| H | 4.240660006030 | -1.410609503827 | 1.098532458160 |
| O | 5.418548463622 | -0.666295404285 | -0.465974596553 |
| C | 6.711487627374 | -0.618302210638 | 0.170110050919 |
| H | 6.856696807956 | 0.326654794170 | 0.710154564915 |
| H | 6.844981840698 | -1.453648050537 | 0.870071978394 |
| H | 7.446099074589 | -0.695256083418 | -0.630696834988 |

transCydiol_TS1-F1000

($E_F$ = -773.03413633 a.u.; $G_F$ = -772.73421152 a.u.)

0 1

| | | | |
|---|---|---|---|
| C | -1.224887960702 | -0.239490620722 | 0.688485645207 |
| C | 1.224824816360 | -0.239240355534 | -0.688594943299 |
| C | 0.713658181664 | 0.979998750778 | -1.394014070776 |
| C | 0.596144258729 | 2.218472937954 | -0.482409136308 |
| C | -0.596085804974 | 2.218281616298 | 0.483355795747 |
| C | -0.713639334047 | 0.979414413140 | 1.394423867449 |
| H | -1.120389735906 | -1.215035793889 | 1.158326966448 |
| H | 1.404113259236 | 1.228289581380 | -2.219165320416 |
| H | -0.256455729942 | 0.744519223957 | -1.841678673423 |
| H | 0.524489397035 | 3.110072226160 | -1.114448676973 |
| H | 1.528350877757 | 2.314671293080 | 0.083335164966 |
| H | -0.524399296289 | 3.109603972809 | 1.115782236495 |
| H | -1.528289467875 | 2.314759266657 | -0.082345181087 |

| | | | |
|---|---|---|---|
| H | -1.404058951830 | 1.227380349784 | 2.219701537229 |
| H | 0.256476048495 | 0.743688480401 | 1.841953920753 |
| O | -2.439138683422 | -0.011144140085 | 0.062369582654 |
| C | -3.473163701816 | -1.007145104290 | 0.101966130594 |
| H | -3.427246830359 | -1.572536280947 | 1.038870619385 |
| H | -3.363733926774 | -1.705317265880 | -0.734930702192 |
| C | -4.825374162516 | -0.282579058642 | 0.008634832350 |
| H | -4.915231574188 | 0.414947797416 | 0.853895425564 |
| H | -4.860369893449 | 0.305920532554 | -0.919595919471 |
| O | -5.862524496665 | -1.257524612976 | 0.036569805823 |
| C | -7.195069710026 | -0.729122158036 | -0.004475188957 |
| H | -7.394740239580 | -0.080343777689 | 0.859063321752 |
| H | -7.371004314391 | -0.157500920751 | -0.925740163831 |
| H | -7.869533896758 | -1.584562636686 | 0.023346435754 |
| H | 1.120292082622 | -1.214571251389 | -1.158872063153 |
| O | 2.439053612408 | -0.011255253618 | -0.062290429832 |
| C | 3.473170522306 | -1.007131826313 | -0.102779452519 |
| H | 3.363625967635 | -1.706256628349 | 0.733307883863 |
| H | 3.427489709393 | -1.571470558469 | -1.040328594709 |
| C | 4.825315194940 | -0.282582897439 | -0.008405137586 |
| H | 4.915157920076 | 0.416065138610 | -0.852740250136 |
| H | 4.860234496654 | 0.304689877737 | 0.920603452850 |
| O | 5.862532340841 | -1.257421778627 | -0.037591523747 |
| C | 7.195042687290 | -0.728993986866 | 0.004225630509 |
| H | 7.394667702627 | -0.078930736547 | -0.858356023786 |
| H | 7.370944993117 | -0.158720162683 | 0.926330176257 |

| | | | |
|---|---|---|---|
| H | 7.869562642276 | -1.584347652342 | -0.024860979394 |

transCydiol-F1000

($E_F$ = -773.13000645 a.u.; $G_F$ = -772.82354313 a.u.)

0 1

| | | | |
|---|---|---|---|
| C | -0.575578918196 | -0.354471325432 | 0.519514763885 |
| C | 0.575492283218 | -0.355111937782 | -0.519538907168 |
| C | 0.484530810201 | 0.902652659901 | -1.394437545416 |
| C | 0.546205236904 | 2.177984832554 | -0.541965366594 |
| C | -0.546050046563 | 2.178720405981 | 0.538799128548 |
| C | -0.484802697228 | 0.904407189753 | 1.392847176988 |
| H | -0.486124133329 | -1.255711945210 | 1.140342844705 |
| H | 1.273134731487 | 0.899605469773 | -2.151410755419 |
| H | -0.468444626089 | 0.854748512192 | -1.929047491221 |
| H | 0.447385268501 | 3.059722020560 | -1.182539049338 |
| H | 1.529039116218 | 2.239056943038 | -0.062045664976 |
| H | -0.447101981532 | 3.061205356990 | 1.178323142759 |
| H | -1.528831116506 | 2.239383638726 | 0.058736763019 |
| H | -1.273842276997 | 0.902400504339 | 2.149385070239 |
| H | 0.467899180139 | 0.857124846778 | 1.928003864004 |
| O | -1.808636555730 | -0.412575452988 | -0.232216907123 |
| C | -2.991396878639 | -0.629329832215 | 0.539808493630 |
| H | -3.112165094602 | 0.136695353410 | 1.313810150085 |
| H | -2.953361242442 | -1.608296745234 | 1.037076640468 |
| C | -4.221349012791 | -0.565886677033 | -0.375515830788 |
| H | -4.234806396030 | 0.401129069589 | -0.899138461780 |

| | | | |
|---|---|---|---|
| H | -4.172825553425 | -1.361523682871 | -1.132702133684 |
| O | -5.371323253110 | -0.715331257395 | 0.454729093771 |
| C | -6.636041630629 | -0.639353978475 | -0.212980193872 |
| H | -6.773501930427 | 0.334538240371 | -0.702450181157 |
| H | -6.741480233876 | -1.431983614663 | -0.966280776677 |
| H | -7.399886923773 | -0.767985115509 | 0.553606951721 |
| H | 0.486074277616 | -1.257095682263 | -1.139312855227 |
| O | 1.808468352455 | -0.412555972473 | 0.232391946079 |
| C | 2.991875732106 | -0.624807944475 | -0.539861206538 |
| H | 2.954017436679 | -1.600525148558 | -1.043440170515 |
| H | 3.113594521560 | 0.146126561727 | -1.308781006224 |
| C | 4.221114519255 | -0.567505913662 | 0.376780473323 |
| H | 4.233711546610 | 0.395634151978 | 0.907476717859 |
| H | 4.172577855304 | -1.368695172366 | 1.128050355515 |
| O | 5.371658387816 | -0.710255794260 | -0.453870907179 |
| C | 6.635983034740 | -0.638780462721 | 0.215054438227 |
| H | 6.772806135367 | 0.331490943450 | 0.711838023182 |
| H | 6.741336279463 | -1.436958477789 | 0.962488712693 |
| H | 7.400283296922 | -0.761412640054 | -0.552066897436 |

transCydiol_Int1-F1000

($E_F$ = -773.12530289 a.u.; $G_F$ = -772.82975480 a.u.)

0 1

| | | | |
|---|---|---|---|
| C | -3.216668720311 | -0.032287577510 | -0.293851254117 |
| C | 3.216652327989 | -0.032474033784 | 0.293794308456 |
| C | 1.935358240213 | 0.012057588811 | -0.491225762102 |

| | | | |
|---|---|---|---|
| C | 0.664253691436 | 0.026020038672 | 0.396000290365 |
| C | -0.664270593027 | 0.026271913515 | -0.396016965018 |
| C | -1.935375671145 | 0.011742241977 | 0.491199144193 |
| H | -3.282412432624 | -0.633487830635 | -1.200991312030 |
| H | 1.945599858789 | 0.896928506402 | -1.140090071810 |
| H | 1.882149626648 | -0.853648959852 | -1.174420450166 |
| H | 0.696544868706 | 0.904614410493 | 1.050247056721 |
| H | 0.683266173489 | -0.849581905759 | 1.056709279581 |
| H | -0.696562425654 | 0.905283638094 | -1.049702913726 |
| H | -0.683281961893 | -0.848908173758 | -1.057284701508 |
| H | -1.945617353791 | 0.896198731827 | 1.140628609767 |
| H | -1.882168324186 | -0.854400655132 | 1.173840966519 |
| O | -4.382766704414 | 0.093733317634 | 0.428480747263 |
| C | -5.600686228426 | -0.057627933106 | -0.322224146781 |
| H | -5.635009808294 | -1.051482298954 | -0.784645155286 |
| H | -5.646604507944 | 0.695182083756 | -1.116452149513 |
| C | -6.809200691765 | 0.107390846897 | 0.607011803206 |
| H | -6.764888155531 | -0.640676305390 | 1.411640882485 |
| H | -6.790550608242 | 1.105077426910 | 1.068418858602 |
| O | -7.973626104463 | -0.067605709439 | -0.195685762239 |
| C | -9.226685364071 | 0.055795587691 | 0.488483688236 |
| H | -9.325982729771 | -0.696778080045 | 1.282363010742 |
| H | -9.346159509574 | 1.054612991788 | 0.929459153822 |
| H | -10.003983659974 | -0.103689509123 | -0.258363051082 |
| H | 3.282401103914 | -0.634265001218 | 1.200541896360 |
| O | 4.382748757810 | 0.094024700795 | -0.428456876289 |

| | | | |
|---|---|---|---|
| C | 5.600670103909 | -0.057841720118 | 0.322142710252 |
| H | 5.646584926261 | 0.694424938601 | 1.116885731328 |
| H | 5.635000247468 | -1.052012157604 | 0.783883041886 |
| C | 6.809182051912 | 0.107819103378 | -0.606982566431 |
| H | 6.764864912230 | -0.639690415121 | -1.412129869383 |
| H | 6.790533053522 | 1.105825465568 | -1.067697933114 |
| O | 7.973609084592 | -0.067736129589 | 0.195590570566 |
| C | 9.226667720675 | 0.056137420564 | -0.488494330857 |
| H | 9.325963676486 | -0.695886312452 | -1.282892879115 |
| H | 9.346142358613 | 1.055257868847 | -0.928778903275 |
| H | 10.003966636180 | -0.103864012649 | 0.258240053430 |

transCydiol_TS1-F500

($E_F$ = -772.86913597 a.u.; $G_F$ = -772.56951581 a.u.)

0 1

| | | | |
|---|---|---|---|
| C | -1.282826120240 | -0.140829940714 | 0.803463745623 |
| C | 1.282711486661 | -0.141049317520 | -0.804189585072 |
| C | 0.646215711156 | 1.053936609828 | -1.440176317375 |
| C | 0.569229308114 | 2.287798140058 | -0.515349234445 |
| C | -0.568773498281 | 2.287832488930 | 0.514636954270 |
| C | -0.645863807639 | 1.053924646860 | 1.439415118190 |
| H | -1.139030767909 | -1.133663504201 | 1.224652336955 |
| H | 1.235019975258 | 1.335509367136 | -2.330915764439 |
| H | -0.352600271905 | 0.776789029112 | -1.788985315800 |
| H | 0.459115816132 | 3.181593934229 | -1.138841038575 |
| H | 1.530590676355 | 2.388483325101 | -0.001755854288 |

| | | | |
|---|---|---|---|
| H | -0.458563035961 | 3.181599160985 | 1.138153516816 |
| H | -1.530129799699 | 2.388632904834 | 0.001055933610 |
| H | -1.234383958922 | 1.335578355913 | 2.330311270855 |
| H | 0.352965248123 | 0.776491194399 | 1.787950879949 |
| O | -2.546034057380 | 0.131126216385 | 0.328654058274 |
| C | -3.470390741110 | -0.943087702194 | 0.152567744686 |
| H | -3.423650083803 | -1.636513055417 | 1.000407110255 |
| H | -3.248855864493 | -1.497970098508 | -0.766008411035 |
| C | -4.861771415690 | -0.317464258644 | 0.078137340367 |
| H | -5.042157491125 | 0.258857869114 | 0.997407320818 |
| H | -4.906023804086 | 0.381078358175 | -0.770356551429 |
| O | -5.813131052592 | -1.359465209157 | -0.066652892362 |
| C | -7.163038200208 | -0.902416285688 | -0.096231466872 |
| H | -7.430127751569 | -0.380685830033 | 0.833290686429 |
| H | -7.339480988482 | -0.224886672806 | -0.943208152114 |
| H | -7.794418977785 | -1.784149667601 | -0.206066944125 |
| H | 1.139010987830 | -1.133738528948 | -1.225737460547 |
| O | 2.545635807081 | 0.130553172103 | -0.328405586143 |
| C | 3.470392283230 | -0.943663054077 | -0.154363721988 |
| H | 3.247897530432 | -1.501601048372 | 0.762119975463 |
| H | 3.425261358569 | -1.634400688871 | -1.004469125606 |
| C | 4.861323645564 | -0.317527294488 | -0.076143146588 |
| H | 5.042039930899 | 0.262747370721 | -0.992832657257 |
| H | 4.904566786289 | 0.377447552318 | 0.775306052609 |
| O | 5.813170719119 | -1.359581898880 | 0.064933639491 |
| C | 7.162799990287 | -0.901932241247 | 0.097435315454 |

| | | | |
|---|---|---|---|
| H | 7.430155894953 | -0.375823191818 | -0.829512353768 |
| H | 7.338347403946 | -0.228189684688 | 0.947592225544 |
| H | 7.794610128831 | -1.783790522396 | 0.203636354231 |

transCydiol-F500

($E_F$ = -772.97829547 a.u.; $G_F$ = -772.67101977 a.u.)

0 1

| | | | |
|---|---|---|---|
| C | -0.560029307848 | -0.276561122377 | 0.530125409116 |
| C | 0.560046861278 | -0.276354445372 | -0.530844607048 |
| C | 0.463316790279 | 0.984329424875 | -1.401404361727 |
| C | 0.539286539604 | 2.257422575408 | -0.547036968262 |
| C | -0.539431905777 | 2.257223038756 | 0.547099783968 |
| C | -0.463414561935 | 0.983865479608 | 1.401071855316 |
| H | -0.451483940840 | -1.175846975581 | 1.151402704473 |
| H | 1.243807596834 | 0.979280465356 | -2.166869972272 |
| H | -0.495590100819 | 0.941445629236 | -1.925764976465 |
| H | 0.434678259069 | 3.141034023494 | -1.184086341025 |
| H | 1.528154306544 | 2.315086048193 | -0.079052098333 |
| H | -0.434868914857 | 3.140638432578 | 1.184428593688 |
| H | -1.528300337976 | 2.314986269366 | 0.079129994288 |
| H | -1.243946691446 | 0.978531246786 | 2.166494446300 |
| H | 0.495468650902 | 0.940884439581 | 1.925467718794 |
| O | -1.795203530192 | -0.346441688543 | -0.201965342202 |
| C | -2.957628299163 | -0.582187458081 | 0.583431487302 |
| H | -3.102821000113 | 0.199061952684 | 1.338436844270 |
| H | -2.887081912153 | -1.547260168555 | 1.104491873829 |

| | | | |
|---|---|---|---|
| C | -4.171511798074 | -0.579696584823 | -0.341139287984 |
| H | -4.207738882896 | 0.373268642717 | -0.889491687214 |
| H | -4.088838548886 | -1.391311375006 | -1.078585409153 |
| O | -5.322105766554 | -0.743375978458 | 0.476435913112 |
| C | -6.556239860698 | -0.706957989534 | -0.232259172681 |
| H | -6.697821860991 | 0.255136178449 | -0.744507373190 |
| H | -6.618128689110 | -1.513287598049 | -0.976580517956 |
| H | -7.349035560145 | -0.839690777637 | 0.504168278131 |
| H | 0.451578060567 | -1.175457815623 | -1.152400722211 |
| O | 1.795219485198 | -0.346372670894 | 0.201234904725 |
| C | 2.957725872775 | -0.581359866751 | -0.584267525961 |
| H | 2.887299946658 | -1.545992938325 | -1.106162223879 |
| H | 3.102920047837 | 0.200550180087 | -1.338590591346 |
| C | 4.171539091681 | -0.579553907513 | 0.340398763126 |
| H | 4.207586981139 | 0.372910936171 | 0.889636476274 |
| H | 4.088934807739 | -1.391864660785 | 1.077088634903 |
| O | 5.322212783558 | -0.742307043425 | -0.477251592941 |
| C | 6.556293052757 | -0.706401497980 | 0.231562767926 |
| H | 6.697711587737 | 0.255222456830 | 0.744743221200 |
| H | 6.618239557453 | -1.513436427338 | 0.975117732941 |
| H | 7.349157166160 | -0.838322986609 | -0.504938495331 |

transCydiol_Int1-F500

($E_F$ = -772.91409918 a.u.; $G_F$ = -772.61871156 a.u.)

0 1

| | | | |
|---|---|---|---|
| C | -3.187070226421 | -0.023382409052 | -0.310600395099 |

| | | | |
|---|---|---|---|
| C | 3.187030096003 | -0.023022060881 | 0.310334872563 |
| C | 1.919727587032 | 0.016369170256 | -0.483234050850 |
| C | 0.656580941732 | 0.030237182232 | 0.400372538476 |
| C | -0.656626319038 | 0.030159119951 | -0.400642082425 |
| C | -1.919771059615 | 0.016166253210 | 0.482964852623 |
| H | -3.253885029085 | -0.631341935904 | -1.213278860393 |
| H | 1.932214378938 | 0.900407954345 | -1.133304600327 |
| H | 1.871516223759 | -0.851050778333 | -1.165022184827 |
| H | 0.685669373904 | 0.909340027490 | 1.054266565517 |
| H | 0.672482696766 | -0.845330326783 | 1.061429431392 |
| H | -0.685811116856 | 0.909248707759 | -1.054548828749 |
| H | -0.672432279479 | -0.845419827812 | -1.061685469600 |
| H | -1.932355939103 | 0.900214739727 | 1.133018721488 |
| H | -1.871462097767 | -0.851234899188 | 1.164768371229 |
| O | -4.345586789981 | 0.112083904729 | 0.411818780925 |
| C | -5.558694480894 | -0.032551507109 | -0.335409325935 |
| H | -5.601708574573 | -1.024929723558 | -0.800923669494 |
| H | -5.608721250989 | 0.723699570241 | -1.126472850513 |
| C | -6.740640132661 | 0.134956035812 | 0.613248137247 |
| H | -6.683055516451 | -0.615477144214 | 1.415286076094 |
| H | -6.707385339302 | 1.131044685763 | 1.078057847370 |
| O | -7.917406314950 | -0.031503213147 | -0.163662042403 |
| C | -9.130571655280 | 0.097582996857 | 0.571467995886 |
| H | -9.201196142701 | -0.657420183232 | 1.366841486306 |
| H | -9.222676818084 | 1.094829518938 | 1.023735096806 |
| H | -9.946488373628 | -0.050907759187 | -0.135913324661 |

| | | | |
|---|---|---|---|
| H | 3.253910399710 | -0.630952949032 | 1.213029748288 |
| O | 4.345532936448 | 0.112552919791 | -0.412086535602 |
| C | 5.558655446422 | -0.031882596573 | 0.335157337193 |
| H | 5.608597272691 | 0.724446475953 | 1.126153793046 |
| H | 5.601779242337 | -1.024215371430 | 0.800762412825 |
| C | 6.740583436068 | 0.135671673364 | -0.613514741303 |
| H | 6.683094925317 | -0.614851821766 | -1.415473854784 |
| H | 6.707206455443 | 1.131707190946 | -1.078427848019 |
| O | 7.917368090726 | -0.030562058030 | 0.163414219260 |
| C | 9.130519062283 | 0.098599037224 | -0.571726258907 |
| H | 9.201228636797 | -0.656465727656 | -1.367037593666 |
| H | 9.222511094489 | 1.095820601940 | -1.024078549456 |
| H | 9.946453493230 | -0.049740367332 | 0.135670770182 |

Cam_TS1

($E_F$ = -929.38447827 a.u.; $G_F$ = -928.99726281 a.u.)

0 1

| | | | |
|---|---|---|---|
| C | -1.794727207069 | 0.967238200281 | -2.633387759132 |
| C | -1.080788462354 | 2.250912988802 | -2.197308653341 |
| C | -2.183494124714 | 1.987796456883 | 0.068788197438 |
| C | -2.773446044563 | 0.723076325695 | -0.493895567929 |
| C | -1.708290885724 | -0.396505348891 | -0.571852752789 |
| C | -1.069326593882 | -0.239009089748 | -1.970980336946 |
| H | -0.103011678173 | 2.120026744387 | -1.734723666457 |
| H | -1.217169777992 | 1.916128835772 | 0.568293065239 |
| H | -3.628273651486 | 0.443792027258 | 0.132683315282 |

| | | | |
|---|---|---|---|
| H | -0.978116533836 | -0.313113234041 | 0.236638063143 |
| H | -2.183092866181 | -1.373746845280 | -0.464473758263 |
| H | -1.223857422379 | -1.135639002842 | -2.575284768144 |
| H | 0.010529554498 | -0.077570183209 | -1.928366032951 |
| C | -3.223801319584 | 0.912233599848 | -1.974568489883 |
| C | -4.058619364827 | -0.294185042296 | -2.445513541671 |
| H | -4.388455091557 | -0.161255034094 | -3.478821744058 |
| H | -3.521752387222 | -1.242876743941 | -2.388214604512 |
| H | -4.956438139906 | -0.382912900674 | -1.826755809253 |
| C | -4.053032451428 | 2.181316379782 | -2.205612429279 |
| H | -4.291177141161 | 2.291810956163 | -3.267656070769 |
| H | -4.996564649191 | 2.122192714247 | -1.655001297883 |
| H | -3.525407247379 | 3.075851991009 | -1.877842447174 |
| O | -1.085834221065 | 3.274746960113 | -3.142711906162 |
| O | -3.101174010055 | 2.765606826538 | 0.773779564608 |
| C | -1.812512804186 | 0.842645205167 | -4.163176113481 |
| H | -0.792674829401 | 0.832748668317 | -4.556703009178 |
| H | -2.291241975274 | -0.090474618091 | -4.466814348307 |
| H | -2.341473617655 | 1.675757472491 | -4.625842243741 |
| C | 0.172941776064 | 3.663918935172 | -3.766561834292 |
| H | 0.956971656393 | 2.946130541337 | -3.508895727805 |
| H | 0.462054571084 | 4.647997620872 | -3.387444767480 |
| C | 0.056995721059 | 3.740568634114 | -5.315232819702 |
| H | -0.212324779452 | 2.756083423079 | -5.717196429821 |
| H | -0.736760907397 | 4.449697941687 | -5.587976451833 |
| C | -2.998092439916 | 2.846066482094 | 2.222484183143 |

| | | | |
|---|---|---|---|
| H | -3.016898395461 | 3.903931606331 | 2.493737042366 |
| H | -2.050375942581 | 2.416088942384 | 2.560792442567 |
| C | -4.174110216374 | 2.137570435885 | 2.954979631401 |
| H | -5.125289849652 | 2.530347707436 | 2.569948252890 |
| H | -4.144661381256 | 1.056498254310 | 2.756155488390 |
| O | 1.326746830216 | 4.167282769399 | -5.829580125571 |
| C | 1.469439792210 | 4.261003775972 | -7.268992453282 |
| H | 1.305158731940 | 3.287924120085 | -7.749157485115 |
| H | 0.770172000594 | 4.993303234386 | -7.691921851846 |
| H | 2.492861276952 | 4.588225993276 | -7.446975348302 |
| O | -4.036662189058 | 2.403207443844 | 4.358021175500 |
| C | -5.055271409326 | 1.886946997202 | 5.250230484241 |
| H | -6.043455040314 | 2.296618299272 | 5.005153881719 |
| H | -5.100643816201 | 0.790990201253 | 5.212316604319 |
| H | -4.765211758974 | 2.205945280937 | 6.250461180186 |

Cam_Int1

($E_F$ = -929.50587285 a.u.; $G_F$ = -929.12223898 a.u.)

0 1

| | | | |
|---|---|---|---|
| C | -1.489993171916 | 1.814075943249 | -2.520171888190 |
| C | -0.986492587656 | 2.774155830448 | -3.594152083015 |
| C | -3.278531335944 | 2.013311060139 | 0.956500142539 |
| C | -2.608635465422 | 1.488727527392 | -0.294730673606 |
| C | -1.090072890162 | 1.138510154316 | -0.124838305250 |
| C | -0.381237975648 | 1.464629746456 | -1.467560676087 |
| H | -0.581469161999 | 3.738876526311 | -3.296341085046 |

| | | | |
|---|---|---|---|
| H | -2.908918805992 | 2.924891025453 | 1.423260438444 |
| H | -3.143965660555 | 0.573387406116 | -0.564795824798 |
| H | -0.659998107668 | 1.731866537117 | 0.686018526314 |
| H | -0.975492209763 | 0.090517791127 | 0.160156253824 |
| H | 0.238136817883 | 0.639249097816 | -1.826558592788 |
| H | 0.284193828020 | 2.321935195399 | -1.341388855032 |
| C | -2.627634084666 | 2.438773914438 | -1.567263674592 |
| C | -4.012029864771 | 2.463915865370 | -2.221571545469 |
| H | -3.987288803120 | 3.038969523040 | -3.152254234782 |
| H | -4.375049138436 | 1.459860930798 | -2.450037716490 |
| H | -4.736359814200 | 2.930742498795 | -1.548017767107 |
| C | -2.259730629924 | 3.880076174956 | -1.160302323833 |
| H | -2.259305143040 | 4.539179139743 | -2.031027418623 |
| H | -2.999168669451 | 4.273072556902 | -0.459078573315 |
| H | -1.278241252835 | 3.945477794307 | -0.683705922860 |
| O | -0.443417224677 | 2.264755460693 | -4.765435559985 |
| O | -3.856718678371 | 1.107930833068 | 1.835323728059 |
| C | -1.967094772101 | 0.514657491959 | -3.198674533090 |
| H | -1.134931790950 | 0.055151032549 | -3.734780289063 |
| H | -2.336698703743 | -0.206233041710 | -2.466548183619 |
| H | -2.758714410166 | 0.707152440364 | -3.924742249844 |
| C | 0.110380873301 | 3.244858075751 | -5.679784400077 |
| H | 0.916650804983 | 3.795739598903 | -5.182014581972 |
| H | -0.669803465954 | 3.957590347009 | -5.966242977571 |
| C | 0.677650976811 | 2.578981441714 | -6.957211866965 |
| H | 1.460871168691 | 1.858343184943 | -6.683520609161 |

| | | | |
|---|---|---|---|
| H | -0.122129224833 | 2.034611464973 | -7.477987455718 |
| C | -4.361506747147 | 1.678297711536 | 3.071032057915 |
| H | -5.115219632144 | 2.438161399727 | 2.840050897665 |
| H | -3.539877362000 | 2.160221893490 | 3.613155651044 |
| C | -4.996356968884 | 0.605038172383 | 3.988255628097 |
| H | -5.837856313468 | 0.123681549856 | 3.471218944540 |
| H | -4.252521769482 | -0.167956122159 | 4.226409253906 |
| O | 1.206898678888 | 3.628805275551 | -7.778493399291 |
| C | 1.795819428531 | 3.264789119250 | -9.051368608560 |
| H | 2.654567431629 | 2.594987621463 | -8.916210500107 |
| H | 1.060777659353 | 2.780379461779 | -9.706589489123 |
| H | 2.128357033542 | 4.198442985389 | -9.502897637881 |
| O | -5.437432659033 | 1.279954553712 | 5.174672817010 |
| C | -6.085755507572 | 0.494021237864 | 6.204753510684 |
| H | -7.002666037732 | 0.022736463280 | 5.828675429853 |
| H | -5.415644031360 | -0.282550817873 | 6.594919413428 |
| H | -6.335120935013 | 1.194698593526 | 7.000390362122 |

Cam

($E_F$ = -929.41833017 a.u.; $G_F$ = -929.02438381 a.u.)

0 1

| | | | |
|---|---|---|---|
| C | -1.837428138740 | 0.806105876940 | -2.546438018874 |
| C | -1.199680694235 | 2.024144040938 | -1.784887677487 |
| C | -1.919870042456 | 1.909581904095 | -0.314064832057 |
| C | -2.803125934358 | 0.652094247735 | -0.504621180487 |
| C | -1.872735431852 | -0.579215164815 | -0.527601212298 |

| | | | |
|---|---|---|---|
| C | -1.199449471310 | -0.466980687757 | -1.925667184622 |
| H | -0.145783553272 | 1.805540178044 | -1.588620497520 |
| H | -1.107927312897 | 1.700336353043 | 0.393188231839 |
| H | -3.595744696261 | 0.598896987345 | 0.240682193108 |
| H | -1.151039079103 | -0.556246153667 | 0.292903877435 |
| H | -2.439121502303 | -1.506548565867 | -0.426598474558 |
| H | -1.412582482140 | -1.340811065645 | -2.545001806285 |
| H | -0.111263822067 | -0.380069800177 | -1.867998753084 |
| C | -3.291746156789 | 0.766889113258 | -1.971622684473 |
| C | -4.129438959817 | -0.436211602700 | -2.434808999663 |
| H | -4.388152322491 | -0.335780940750 | -3.492713366623 |
| H | -3.632510418719 | -1.398329977616 | -2.308345171121 |
| H | -5.067665428475 | -0.472055955606 | -1.872550408786 |
| C | -4.109590246311 | 2.036527016623 | -2.263602032342 |
| H | -4.357357529659 | 2.083662234666 | -3.328346488770 |
| H | -5.049634054175 | 2.009142290528 | -1.704023565682 |
| H | -3.592783327539 | 2.955616260639 | -1.999207116757 |
| O | -1.235884493577 | 3.246656307827 | -2.544369747000 |
| O | -2.667870713008 | 3.007148531290 | 0.218746894515 |
| C | -1.712796372861 | 0.860318828983 | -4.059069398271 |
| H | -0.666021943100 | 0.880704813648 | -4.375780376665 |
| H | -2.173726758988 | -0.024361849175 | -4.506303566303 |
| H | -2.206942359539 | 1.744238697394 | -4.461418495017 |
| C | -0.020526008223 | 3.534850962166 | -3.288737038622 |
| H | 0.628094437854 | 2.654597613083 | -3.315202626895 |
| H | 0.520485947562 | 4.334894734168 | -2.772674468873 |

| | | | |
|---|---|---|---|
| C | -0.247329484035 | 3.972104302660 | -4.763641380179 |
| H | -0.830667179813 | 3.214567448104 | -5.298478913492 |
| H | -0.800162670926 | 4.920679826154 | -4.800458900446 |
| C | -2.736071745357 | 3.087050014550 | 1.659772414255 |
| H | -2.960370861110 | 4.133123455742 | 1.875111359910 |
| H | -1.756408613049 | 2.853418343998 | 2.095308644787 |
| C | -3.819032633211 | 2.236144517866 | 2.399188594732 |
| H | -4.793635379914 | 2.381661538944 | 1.911861781199 |
| H | -3.572325928559 | 1.166342511466 | 2.364581883967 |
| O | 1.055331428548 | 4.114349195396 | -5.355549136754 |
| C | 1.128847709212 | 4.441359835641 | -6.764448487213 |
| H | 0.643192129750 | 3.671471790820 | -7.377915612097 |
| H | 0.664363177599 | 5.413316994494 | -6.974473556960 |
| H | 2.190120413726 | 4.484828598543 | -7.005897094044 |
| O | -3.847962098266 | 2.691928126223 | 3.760249684932 |
| C | -4.747179911794 | 2.025740271134 | 4.680190442536 |
| H | -5.792308305904 | 2.129890336670 | 4.361887798477 |
| H | -4.504394476158 | 0.959270004855 | 4.773156637268 |
| H | -4.607886659608 | 2.517004728958 | 5.642297004159 |

Cam_TS2A

($E_F$ = -929.46746296 a.u.; $G_F$ = -929.08756704 a.u.)

0 1

| | | | |
|---|---|---|---|
| C | -1.542532888962 | 1.706036456951 | -2.548818528669 |
| C | -1.065478804891 | 2.673140466090 | -3.616134697419 |
| C | -3.367237047958 | 1.766108991271 | 0.874847875268 |

| | | | |
|---|---|---|---|
| C | -2.951808263213 | 1.380058384908 | -0.381019886402 |
| C | -0.786876324185 | 0.580174808576 | -0.387911320984 |
| C | -0.320912517029 | 1.267983859747 | -1.621603109649 |
| H | -0.703259766317 | 3.657029310235 | -3.327773078401 |
| H | -3.160905563749 | 2.750009373391 | 1.283686786698 |
| H | -3.328488858875 | 0.410730487671 | -0.693083424306 |
| H | -0.461717126900 | 0.938943555992 | 0.580055335426 |
| H | -1.033061590854 | -0.474495191508 | -0.430606022667 |
| H | 0.323462826388 | 0.630789720703 | -2.239406683117 |
| H | 0.261095077426 | 2.157942029786 | -1.372057038877 |
| C | -2.624142931140 | 2.359130900185 | -1.544407607858 |
| C | -3.941986961806 | 2.648060035543 | -2.298883644560 |
| H | -3.748667107196 | 3.251371684929 | -3.190796822499 |
| H | -4.441456387860 | 1.728655140447 | -2.609823471653 |
| H | -4.631805182672 | 3.192819029475 | -1.649365287965 |
| C | -2.099109765746 | 3.694430042932 | -0.979420352318 |
| H | -1.898112952802 | 4.409107627547 | -1.778572233676 |
| H | -2.849233653393 | 4.155577311149 | -0.333133894809 |
| H | -1.187931562402 | 3.563012245428 | -0.392666924861 |
| O | -0.471131600382 | 2.168013569523 | -4.763656835089 |
| O | -3.988981518116 | 0.895367962263 | 1.757897797022 |
| C | -2.095234641995 | 0.441347751631 | -3.237267179854 |
| H | -1.314705579580 | -0.011491520582 | -3.850731982028 |
| H | -2.425437466503 | -0.301574203476 | -2.509955858870 |
| H | -2.931951820229 | 0.676650300437 | -3.896477497340 |
| C | 0.081775190953 | 3.154793563379 | -5.672424318764 |

| | | | |
|---|---|---|---|
| H | 0.870701700370 | 3.719744098159 | -5.163087777213 |
| H | -0.705510716152 | 3.853548747705 | -5.973200703071 |
| C | 0.678507895417 | 2.496366354664 | -6.940106233547 |
| H | 1.472133414112 | 1.791857057656 | -6.654795103952 |
| H | -0.103987453192 | 1.935391764025 | -7.469440728761 |
| C | -4.356841906296 | 1.448244428733 | 3.047464593922 |
| H | -4.993366009011 | 2.328692880476 | 2.905874651376 |
| H | -3.456228930767 | 1.754840652500 | 3.590891936810 |
| C | -5.128199764138 | 0.414480111389 | 3.903661193571 |
| H | -6.038218354039 | 0.101991192178 | 3.373089174312 |
| H | -4.502716169612 | -0.474483825126 | 4.064259713149 |
| O | 1.197191264212 | 3.554890709952 | -7.756931584179 |
| C | 1.808829444675 | 3.200598660294 | -9.021905058695 |
| H | 2.679378350241 | 2.548818090930 | -8.874951475975 |
| H | 1.091762031059 | 2.700034557778 | -9.684828291762 |
| H | 2.127581017864 | 4.140070756100 | -9.471290964948 |
| O | -5.448655618809 | 1.052115385465 | 5.147475092412 |
| C | -6.187333218872 | 0.287306600537 | 6.132028303426 |
| H | -7.167872391230 | -0.018046465216 | 5.745272928562 |
| H | -5.629484255546 | -0.604650222395 | 6.444263264127 |
| H | -6.321480487624 | 0.951414940093 | 6.984767109779 |

Cam_Int2A

($E_F$ = -929.47416039 a.u.; $G_F$ = -929.09666943 a.u.)

0 1

| | | | |
|---|---|---|---|
| C | -1.540922724569 | 1.707325288460 | -2.528371734381 |

| | | | |
|---|---|---|---|
| C | -1.240163685554 | 2.631282762833 | -3.683258172547 |
| C | -3.435234000012 | 1.740204429020 | 0.880007653299 |
| C | -3.103821859669 | 1.413863925077 | -0.383602421989 |
| C | -0.185818617134 | 0.548362199173 | -0.612520491624 |
| C | -0.108263768979 | 1.393066210607 | -1.809499403257 |
| H | -1.150049860144 | 3.703012589986 | -3.522901985130 |
| H | -3.209655492819 | 2.714402437233 | 1.305223820459 |
| H | -3.396107803664 | 0.424180718446 | -0.719836331058 |
| H | -0.239781157755 | 0.983792668547 | 0.376415628757 |
| H | -0.400251113319 | -0.510668151300 | -0.692598665870 |
| H | 0.479768354524 | 0.922130607253 | -2.604389492864 |
| H | 0.358270183961 | 2.353706268316 | -1.584211482842 |
| C | -2.569381919327 | 2.383606493154 | -1.472838698213 |
| C | -3.826187472997 | 2.867117977889 | -2.247938009364 |
| H | -3.560452778064 | 3.569650347581 | -3.041436440483 |
| H | -4.362629468028 | 2.032944087425 | -2.704301058477 |
| H | -4.513535097208 | 3.361879321213 | -1.557457400912 |
| C | -1.918415124498 | 3.614315341383 | -0.808772953896 |
| H | -1.498999115598 | 4.297148476914 | -1.549193053975 |
| H | -2.661033354027 | 4.182235716042 | -0.243188000495 |
| H | -1.122038851124 | 3.327589778299 | -0.120140053636 |
| O | -0.471825670681 | 2.148577496755 | -4.729733643129 |
| O | -4.091795348631 | 0.883061826422 | 1.753862700297 |
| C | -2.073755771196 | 0.370987694218 | -3.080664293805 |
| H | -1.398524822210 | -0.001824450605 | -3.852686836430 |
| H | -2.142318852806 | -0.385308926602 | -2.297779784556 |

| | | | |
|---|---|---|---|
| H | -3.059164184620 | 0.489994384041 | -3.534609337998 |
| C | -0.076050784342 | 3.125238193039 | -5.729133326453 |
| H | 0.545450194953 | 3.895772911485 | -5.259679637941 |
| H | -0.969640287496 | 3.603437811562 | -6.142389360339 |
| C | 0.720921680415 | 2.473382760656 | -6.885071720390 |
| H | 1.626052971780 | 1.992484125816 | -6.488974037205 |
| H | 0.106337065961 | 1.702024002440 | -7.369272377814 |
| C | -4.424105468650 | 1.428591805410 | 3.056152573735 |
| H | -5.018027207007 | 2.341782467730 | 2.938591213226 |
| H | -3.507426356989 | 1.678224700302 | 3.601902472532 |
| C | -5.242097177287 | 0.415662826394 | 3.893881772741 |
| H | -6.166592306353 | 0.157670287692 | 3.359104694767 |
| H | -4.659266584244 | -0.505031402147 | 4.034508880240 |
| O | 1.053511245352 | 3.521837660228 | -7.804929118156 |
| C | 1.800987827592 | 3.170667525580 | -8.995895515602 |
| H | 2.778969807749 | 2.743331140322 | -8.740891421327 |
| H | 1.246603347561 | 2.456615643435 | -9.617886127585 |
| H | 1.940542015554 | 4.101356614857 | -9.543991734527 |
| O | -5.530280581977 | 1.041578254495 | 5.151462562767 |
| C | -6.301034565410 | 0.290818345563 | 6.122136779163 |
| H | -7.295521432387 | 0.039164941070 | 5.732383581224 |
| H | -5.784125904234 | -0.632274561216 | 6.413528120027 |
| H | -6.402387070036 | 0.942051030899 | 6.989196464145 |

Cam_TS2B

($E_F$ = -929.47716708 a.u.; $G_F$ = -929.09753813 a.u.)

0 1

| | | | |
|---|---|---|---|
| C | -1.512338165489 | 1.742751721394 | -2.646248500021 |
| C | -1.035707579880 | 2.731340920070 | -3.712324316139 |
| C | -2.855146469685 | 1.399218296266 | 1.228714600916 |
| C | -2.507972295819 | 1.010874612664 | -0.070745699391 |
| C | -1.013522959026 | 0.741849111831 | -0.345887151828 |
| C | -0.413840534055 | 1.464715804821 | -1.578134916398 |
| H | -0.756867079321 | 3.741367284530 | -3.418712866656 |
| H | -2.155827191922 | 1.955930453855 | 1.848884969521 |
| H | -3.198604889276 | 0.293254942421 | -0.507139573824 |
| H | -0.426330345478 | 1.004847015690 | 0.538770980428 |
| H | -0.895453305658 | -0.340365829848 | -0.465391887889 |
| H | 0.401304715426 | 0.881525687418 | -2.017018224723 |
| H | 0.023220584021 | 2.416845216850 | -1.265543468096 |
| C | -2.694893218450 | 2.335438175611 | -1.813579953775 |
| C | -4.098981320185 | 2.121602468132 | -2.319261175699 |
| H | -4.271358860988 | 2.683724677999 | -3.249039065287 |
| H | -4.320490586592 | 1.072744208382 | -2.525465417255 |
| H | -4.824533819156 | 2.475769731711 | -1.581357146798 |
| C | -2.487419026140 | 3.715356420620 | -1.233209339378 |
| H | -2.643023950342 | 4.493687100537 | -1.993953937134 |
| H | -3.208696444728 | 3.894797118015 | -0.430789469241 |
| H | -1.489623125656 | 3.856583017554 | -0.813447306340 |
| O | -0.404930859559 | 2.252919819170 | -4.851444167320 |
| O | -4.063875359924 | 1.102462285977 | 1.838956814177 |
| C | -1.893995868876 | 0.416125844506 | -3.332759324673 |

| | | | |
|---|---|---|---|
| H | -1.015637371369 | -0.012346677361 | -3.818362837004 |
| H | -2.274978099937 | -0.308787840238 | -2.610974958202 |
| H | -2.653842555492 | 0.567870589596 | -4.101383567581 |
| C | 0.051890105942 | 3.252292832122 | -5.797764762740 |
| H | 0.763942383206 | 3.925181505326 | -5.306210072186 |
| H | -0.801622502581 | 3.844211331191 | -6.143896260288 |
| C | 0.743802499718 | 2.598171322457 | -7.018906399514 |
| H | 1.599001491978 | 1.996553419860 | -6.680591861617 |
| H | 0.036674573502 | 1.930619531795 | -7.530453128316 |
| C | -4.134657403223 | 1.398153512922 | 3.259334791414 |
| H | -4.232354061094 | 2.479427478045 | 3.405145381386 |
| H | -3.215416257267 | 1.063607514980 | 3.751433359284 |
| C | -5.330363433426 | 0.687096049300 | 3.939191908237 |
| H | -6.274150666605 | 1.018998949484 | 3.485018183811 |
| H | -5.242675996269 | -0.398526936202 | 3.794400087892 |
| O | 1.170005688718 | 3.658873398768 | -7.884616559993 |
| C | 1.845508199333 | 3.299486949593 | -9.115186080520 |
| H | 2.774153998199 | 2.750348966699 | -8.914224970463 |
| H | 1.199905000325 | 2.691014637691 | -9.761104815622 |
| H | 2.077727726022 | 4.240069602587 | -9.612712126957 |
| O | -5.278887189006 | 1.029276266919 | 5.331266537357 |
| C | -6.285523653369 | 0.468243010276 | 6.209551178734 |
| H | -7.293875976040 | 0.784641503739 | 5.914011796218 |
| H | -6.240111235668 | -0.628160619466 | 6.218361937945 |
| H | -6.059293910115 | 0.851362380285 | 7.203629342246 |

Cam_Int2B

($E_F$ = -929.62117093 a.u.; $G_F$ = -929.23874163 a.u.)

0 1

| | | | |
|---|---|---|---|
| C | -1.366770168210 | 1.803631106717 | -2.768574035378 |
| C | -0.731772430714 | 2.827660185201 | -3.729551725411 |
| C | -3.504279210521 | 0.919137650698 | 1.837307968843 |
| C | -2.983580682877 | 1.758932469280 | 0.924743446124 |
| C | -2.716390673423 | 1.408440100453 | -0.546267904435 |
| C | -1.491070760732 | 2.096768159418 | -1.244393265007 |
| H | -0.400429879703 | 3.771894494840 | -3.294565298521 |
| H | -3.686903370025 | -0.128894081930 | 1.601061716443 |
| H | -2.840026718662 | 2.796890730644 | 1.218620377532 |
| H | -2.625138741391 | 0.322284073975 | -0.647008720561 |
| H | -3.606530357567 | 1.688309931679 | -1.124693017300 |
| H | -0.567794531821 | 1.764366029600 | -0.755935476485 |
| H | -1.553090634317 | 3.174013983078 | -1.069702242369 |
| C | -2.228592980900 | 2.585639166156 | -3.776797003573 |
| C | -2.806003241256 | 1.840054017818 | -4.969201013144 |
| H | -3.178199251464 | 2.550925437663 | -5.713592848145 |
| H | -2.067433239592 | 1.206997613964 | -5.458779601086 |
| H | -3.647776677512 | 1.211621423458 | -4.661104546607 |
| C | -3.143064890751 | 3.698389894662 | -3.289006194191 |
| H | -3.355141753129 | 4.392474995546 | -4.108793105235 |
| H | -4.099467068119 | 3.296580075594 | -2.938288262832 |
| H | -2.706965677455 | 4.277755834227 | -2.474263713555 |
| O | 0.107472168759 | 2.390628662352 | -4.795019930321 |

| | | | |
|---|---|---|---|
| O | -3.868669051021 | 1.294441657553 | 3.121646978974 |
| C | -1.086189516889 | 0.333306816697 | -3.052743890929 |
| H | -0.326130969509 | -0.038664383145 | -2.358821032873 |
| H | -1.983975233281 | -0.278287640997 | -2.924474122378 |
| H | -0.706793254397 | 0.179819183562 | -4.061400954825 |
| C | 0.182129342851 | 3.320533877390 | -5.899832498238 |
| H | 0.536584491011 | 4.297039198751 | -5.545837680796 |
| H | -0.809783821695 | 3.460407593014 | -6.344326341451 |
| C | 1.155290487489 | 2.809775838815 | -6.991255578981 |
| H | 2.152507278114 | 2.663687800121 | -6.553214279830 |
| H | 0.805412027381 | 1.840251728140 | -7.373224238837 |
| C | -4.465850455413 | 0.270876551384 | 3.957883564010 |
| H | -3.743438066963 | -0.532114627320 | 4.140880093908 |
| H | -5.342646412491 | -0.156236803772 | 3.458931938724 |
| C | -4.910846400061 | 0.863424357715 | 5.317607298736 |
| H | -4.041172792257 | 1.289676150069 | 5.836424935149 |
| H | -5.636293684087 | 1.670581587135 | 5.145696934041 |
| O | 1.191316032408 | 3.790222011309 | -8.038312076816 |
| C | 2.047216220643 | 3.531009067062 | -9.177535507349 |
| H | 3.098802707793 | 3.444769441272 | -8.875123901417 |
| H | 1.747875716165 | 2.614118089807 | -9.701470550443 |
| H | 1.927539278551 | 4.386097548000 | -9.841627343251 |
| O | -5.494547697333 | -0.202131458795 | 6.078545033896 |
| C | -5.997148409038 | 0.108464627753 | 7.401935740626 |
| H | -5.196594980308 | 0.477272511023 | 8.055590708433 |
| H | -6.799376971259 | 0.855918017261 | 7.357867113234 |

| | | | |
|---|---|---|---|
| H | -6.390430053507 | -0.826803988272 | 7.797849963312 |

Cam_TS2C

($E_F$ = -929.47941076 a.u.; $G_F$ = -929.10086023 a.u.)

0 1

| | | | |
|---|---|---|---|
| C | -1.153466665769 | 1.505628545480 | -2.694403688311 |
| C | -0.468363184895 | 2.368113610628 | -3.570520900139 |
| C | -3.385843187378 | 1.851432817989 | 0.969674200034 |
| C | -2.789349439583 | 1.345047566278 | -0.331144888923 |
| C | -1.328123816456 | 0.774507945170 | -0.200042424025 |
| C | -0.420762257439 | 1.206091358938 | -1.380667673303 |
| H | 0.210685583129 | 3.127788128355 | -3.193208358995 |
| H | -2.971114495988 | 2.740101599263 | 1.442803800790 |
| H | -3.436003648169 | 0.524305248323 | -0.658292186318 |
| H | -0.876504300856 | 1.107200355241 | 0.738511671576 |
| H | -1.384123235515 | -0.315280608206 | -0.138458231411 |
| H | 0.325374768705 | 0.421917065984 | -1.563450540071 |
| H | 0.149052542661 | 2.095544604281 | -1.096308543873 |
| C | -2.778502084831 | 2.401779389697 | -1.455638041575 |
| C | -4.002468205371 | 2.470078340523 | -2.327976874238 |
| H | -3.811907907813 | 3.078709303369 | -3.217803918984 |
| H | -4.329660275030 | 1.479993296462 | -2.654291552239 |
| H | -4.843266094772 | 2.931117258694 | -1.788057489381 |
| C | -2.218330078235 | 3.755988289052 | -1.092525469708 |
| H | -1.956237064076 | 4.316748245067 | -1.994589221112 |
| H | -2.958922327997 | 4.350095985976 | -0.539048049947 |

| | | | |
|---|---|---|---|
| H | -1.325920330458 | 3.685816473624 | -0.465661248466 |
| O | -0.550443376326 | 2.285671115862 | -4.952072648626 |
| O | -3.984112467575 | 0.945710288690 | 1.834052706570 |
| C | -1.861619087704 | 0.329124655110 | -3.339670507792 |
| H | -1.128334412503 | -0.377882495958 | -3.746722198216 |
| H | -2.481304798484 | -0.212550491045 | -2.623386201740 |
| H | -2.495794962841 | 0.653105865549 | -4.166346356586 |
| C | 0.343923707641 | 3.150551318689 | -5.698608562908 |
| H | 1.338986506333 | 3.135380039064 | -5.241729733146 |
| H | -0.033727984830 | 4.178354846381 | -5.674559876985 |
| C | 0.480069708797 | 2.693594777036 | -7.172100765209 |
| H | 0.851954268678 | 1.660063431919 | -7.201724173785 |
| H | -0.501540193355 | 2.719109425277 | -7.665008914574 |
| C | -4.473736359623 | 1.501568578124 | 3.082425689617 |
| H | -5.220038984488 | 2.273941091834 | 2.869638131518 |
| H | -3.643001961060 | 1.964426817388 | 3.627430426422 |
| C | -5.113771246726 | 0.417563981570 | 3.983475118197 |
| H | -5.963243011877 | -0.045073245557 | 3.462326529345 |
| H | -4.376054887664 | -0.367397748864 | 4.200754497025 |
| O | 1.399715584360 | 3.592495474461 | -7.806668301578 |
| C | 1.716831436209 | 3.367458815564 | -9.202657944158 |
| H | 2.179458783710 | 2.383742476951 | -9.352256878437 |
| H | 0.821322735486 | 3.443918602237 | -9.832154454511 |
| H | 2.423757074379 | 4.149275624531 | -9.476630809960 |
| O | -5.540843037246 | 1.071544734341 | 5.186588761309 |
| C | -6.189604511252 | 0.269239342218 | 6.203678987601 |

| | | | |
|---|---|---|---|
| H | -7.113087080793 | -0.185705904701 | 5.823517361981 |
| H | -5.524025272893 | -0.521562069689 | 6.572491025695 |
| H | -6.427629988430 | 0.954566433618 | 7.016000396102 |

Cam_Int2C

($E_F$ = -929.62530924 a.u.; $G_F$ = -929.24294992 a.u.)

0 1

| | | | |
|---|---|---|---|
| C | -1.068544298579 | 1.542510419622 | -3.677677554099 |
| C | -0.549825436730 | 2.432720615431 | -4.547635345420 |
| C | -3.201723202913 | 1.398049818637 | 1.256673778418 |
| C | -3.213217932789 | 1.495262701441 | -0.271250442618 |
| C | -1.987141638811 | 1.257451200899 | -1.182558773778 |
| C | -2.090702527152 | 1.942333564752 | -2.592351443141 |
| H | -0.761436692236 | 3.497757116937 | -4.471399613045 |
| H | -2.229424462588 | 1.245455676085 | 1.727997300656 |
| H | -4.139837393045 | 1.127216054466 | -0.707226336157 |
| H | -1.072085750804 | 1.592252711464 | -0.685655740990 |
| H | -1.880423558665 | 0.176746607107 | -1.318991585038 |
| H | -2.087969874400 | 3.028960636789 | -2.459652907190 |
| H | -3.084071891044 | 1.688301787232 | -2.989615077690 |
| C | -3.421812728013 | 2.741032885521 | 0.588972351562 |
| C | -4.823161572419 | 3.322523939204 | 0.617926814790 |
| H | -5.002914749598 | 3.936042800349 | -0.270712481650 |
| H | -5.576587355913 | 2.533986945624 | 0.652206960148 |
| H | -4.962138064243 | 3.958834650900 | 1.498349125321 |
| C | -2.319044731816 | 3.778734613218 | 0.686895534912 |

| | | | |
|---|---|---|---|
| H | -2.342826353428 | 4.455430337103 | -0.174044352661 |
| H | -2.445432985130 | 4.382851742123 | 1.591084529477 |
| H | -1.325741523983 | 3.327195773805 | 0.726855243928 |
| O | 0.300400892588 | 2.080891718889 | -5.594395814725 |
| O | -4.258797840282 | 0.737678765110 | 1.947869761655 |
| C | -0.783510985332 | 0.069443744216 | -3.827839267378 |
| H | -0.181743489525 | -0.308510301723 | -2.994008129375 |
| H | -1.716343078248 | -0.506712530544 | -3.830684920704 |
| H | -0.244503026012 | -0.136683587346 | -4.752361350646 |
| C | 0.492449721081 | 3.053507026418 | -6.648510664235 |
| H | 0.888566568202 | 3.987342316703 | -6.232651808086 |
| H | -0.462067982563 | 3.270066099429 | -7.140679788424 |
| C | 1.488689530593 | 2.512533494405 | -7.704195531963 |
| H | 2.450754415484 | 2.293467066334 | -7.220468583292 |
| H | 1.098662579779 | 1.576728889479 | -8.128096257849 |
| C | -4.386629375321 | 1.145533586975 | 3.330107119964 |
| H | -4.634567097468 | 2.211606143946 | 3.388653481066 |
| H | -3.436583168117 | 0.988825839646 | 3.857070560627 |
| C | -5.488328916540 | 0.333110099831 | 4.054736000064 |
| H | -6.454729753307 | 0.493888158956 | 3.556479063142 |
| H | -5.250058703223 | -0.738258475606 | 3.999115794797 |
| O | 1.637661809656 | 3.514093841555 | -8.718724666306 |
| C | 2.522456597434 | 3.221605699488 | -9.828653205348 |
| H | 3.551154002824 | 3.054839999348 | -9.484750936667 |
| H | 2.182317775556 | 2.340538678690 | -10.387538721334 |
| H | 2.490615228605 | 4.098321655411 | -10.474071594886 |

| | | | |
|---|---|---|---|
| O | -5.533634351915 | 0.780412431847 | 5.417640869790 |
| C | -6.496083360772 | 0.157017214325 | 6.302153530761 |
| H | -7.523588155116 | 0.317269937599 | 5.950881763551 |
| H | -6.312637472285 | -0.921131037402 | 6.395450642874 |
| H | -6.363646006873 | 0.634801527352 | 7.272039162774 |

Pin_TS1

($E_F$ = -929.50889790 a.u.; $G_F$ = -929.12186947 a.u.)

0 1

| | | | |
|---|---|---|---|
| C | -1.744912223217 | 1.978153366229 | -0.176808160212 |
| C | -1.573600382156 | 3.451221781437 | 0.355772264342 |
| C | -2.149297838989 | 3.958665934614 | 1.665021215870 |
| C | -1.743683786001 | 1.871344435717 | 2.873585572108 |
| C | -0.673427675007 | 1.174219309608 | 2.053972806490 |
| C | -0.442257939713 | 1.636545362856 | 0.616818488664 |
| H | -1.817599771673 | 4.175416465807 | -0.428793292248 |
| H | -2.768086471839 | 1.689341567508 | 2.576517495276 |
| H | 0.266313503263 | 1.259959871195 | 2.609519674335 |
| H | -0.927712824461 | 0.100647581643 | 2.059074930529 |
| H | 0.242114861095 | 0.942200428150 | 0.119308558047 |
| C | -0.062330448487 | 3.139726290119 | 0.561741008655 |
| H | 0.560886531180 | 3.399850982814 | -0.293506021074 |
| H | 0.373348131746 | 3.563738510928 | 1.466164035319 |
| C | -1.453144316960 | 1.994705332650 | -1.692250469562 |
| H | -0.567009464914 | 2.577271705102 | -1.949527943254 |
| H | -1.297327295679 | 0.974597785847 | -2.056361994079 |

| | | | |
|---|---|---|---|
| H | -2.300880012491 | 2.421652372716 | -2.237719438144 |
| C | -3.019696345930 | 1.149862572891 | 0.004055358496 |
| H | -2.900783751718 | 0.182386230740 | -0.494430788757 |
| H | -3.287680310362 | 0.942782373897 | 1.036087521595 |
| H | -3.868984247915 | 1.655446424752 | -0.466172986081 |
| C | -3.641235095038 | 3.998685672174 | 1.860444771338 |
| H | -4.085510689400 | 4.732932908029 | 1.172242909590 |
| H | -4.117596748731 | 3.038503752680 | 1.666688579565 |
| H | -3.876370093796 | 4.314990614356 | 2.878506538662 |
| O | -1.609088876104 | 5.267886175821 | 1.954813430541 |
| O | -1.572790771340 | 1.525421076188 | 4.230503354811 |
| C | -1.533755729853 | 6.208727620961 | 0.823632779586 |
| H | -2.434393716420 | 6.121245873689 | 0.208973048507 |
| H | -0.669696780097 | 5.964117035499 | 0.200320907285 |
| C | -1.410555630358 | 7.714915765677 | 1.200562286015 |
| H | -2.276898330151 | 8.034508958965 | 1.794492334452 |
| H | -0.499809116748 | 7.895920796095 | 1.786075793524 |
| C | -2.437395753416 | 0.493334868880 | 4.803763416026 |
| H | -3.238144386191 | 0.240713271225 | 4.101695323084 |
| H | -2.887947779663 | 0.911040900060 | 5.706682369696 |
| C | -1.684697165562 | -0.821507912072 | 5.205660067141 |
| H | -1.271368959735 | -1.304317307360 | 4.309222160658 |
| H | -0.850901678396 | -0.567399926125 | 5.873640335162 |
| O | -1.361088435071 | 8.412916623142 | -0.064518096351 |
| C | -1.251415406661 | 9.866882264146 | -0.105150913136 |
| H | -2.111899192721 | 10.345187924358 | 0.378487220093 |

| | | | |
|---|---|---|---|
| H | -0.327461044105 | 10.210629689873 | 0.375664554492 |
| H | -1.233536283796 | 10.124691970995 | -1.162919875649 |
| O | -2.628177450360 | -1.688527825858 | 5.864582690153 |
| C | -2.175186438362 | -2.979201794483 | 6.377373836456 |
| H | -1.798842537469 | -3.617576923881 | 5.568595563523 |
| H | -1.391698332142 | -2.851661237679 | 7.134346170374 |
| H | -3.054093679855 | -3.436266594050 | 6.829356703219 |

Pin

($E_F$ = -929.53801613 a.u.; $G_F$ = -929.14579117 a.u.)

0 1

| | | | |
|---|---|---|---|
| C | -1.694003121042 | 1.722684443251 | -0.171727178421 |
| C | -1.809889577692 | 3.232872697403 | 0.312185369975 |
| C | -2.177763545703 | 3.547914514666 | 1.798692659441 |
| C | -1.702389240736 | 2.216506260636 | 2.577895436971 |
| C | -0.397412832407 | 1.604861830311 | 2.034223281395 |
| C | -0.282083843808 | 1.763867555814 | 0.505847738116 |
| H | -2.390741773731 | 3.867438995040 | -0.359369421684 |
| H | -2.508745486189 | 1.567092765685 | 2.253000236844 |
| H | 0.459911212590 | 2.074859861354 | 2.524977965307 |
| H | -0.386908165003 | 0.548162438174 | 2.320018220086 |
| H | 0.505367670012 | 1.130773529520 | 0.086935380138 |
| C | -0.250139543130 | 3.280251406970 | 0.215103430854 |
| H | 0.114872268127 | 3.536476249913 | -0.777090570931 |
| H | 0.255132668502 | 3.899645935228 | 0.953884510635 |
| C | -1.601039156286 | 1.723900805175 | -1.712521955989 |

| | | | |
|---|---|---|---|
| H | -0.994019831017 | 2.531706233834 | -2.120259660577 |
| H | -1.177903643858 | 0.777527751668 | -2.063207095848 |
| H | -2.603904051165 | 1.819479313492 | -2.141031591785 |
| C | -2.702601396721 | 0.609601670445 | 0.182034249469 |
| H | -2.670675489083 | -0.174187410892 | -0.579537423951 |
| H | -2.511832177631 | 0.115624034519 | 1.133959011346 |
| H | -3.725919745833 | 0.996762162180 | 0.197461808555 |
| C | -3.679040949439 | 3.703044562687 | 2.051651677724 |
| H | -4.085999245700 | 4.568430035439 | 1.527081408419 |
| H | -4.231296151751 | 2.822358484216 | 1.715070424173 |
| H | -3.849161337518 | 3.833581533833 | 3.122429131291 |
| O | -1.542165003565 | 4.851990002767 | 2.145164520641 |
| O | -1.727388225940 | 2.133237788890 | 4.019028175769 |
| C | -1.724598124581 | 5.895767082718 | 1.138539308957 |
| H | -2.782745855359 | 5.997229504176 | 0.879714001702 |
| H | -1.186391059648 | 5.637135063850 | 0.223856714535 |
| C | -1.221987763234 | 7.321325541692 | 1.517751212466 |
| H | -1.778298931547 | 7.722542876474 | 2.374791394438 |
| H | -0.154681300745 | 7.297246893848 | 1.774772577830 |
| C | -2.481994694103 | 1.033830645773 | 4.596987497393 |
| H | -3.364317193453 | 0.820226905170 | 3.982311312697 |
| H | -2.829691260852 | 1.400993946969 | 5.564311125833 |
| C | -1.741236144090 | -0.324632626838 | 4.872003188869 |
| H | -1.453974908883 | -0.810076622129 | 3.928735357480 |
| H | -0.828347031969 | -0.131864988530 | 5.451752075513 |
| O | -1.451583856623 | 8.117681782035 | 0.331700472039 |

| | | | |
|---|---|---|---|
| C | -1.079654621712 | 9.526599941209 | 0.296543416969 |
| H | -1.624638728022 | 10.103067370368 | 1.054148779545 |
| H | -0.001143300021 | 9.658641958908 | 0.447452369404 |
| H | -1.356559285907 | 9.872944685409 | -0.698040849179 |
| O | -2.657378277998 | -1.160655311055 | 5.608053461021 |
| C | -2.243198662177 | -2.498158437238 | 6.021196166493 |
| H | -2.004025043107 | -3.125871826160 | 5.153922571369 |
| H | -1.374370330403 | -2.455907367143 | 6.689247756505 |
| H | -3.097130569800 | -2.914836266842 | 6.552952518044 |

Pin_Int1

($E_F$ = -929.73727343 a.u.; $G_F$ = -929.35714613 a.u.)

0 1

| | | | |
|---|---|---|---|
| C | -1.984663482483 | 2.744046715898 | 0.949974076263 |
| C | -1.267567394517 | 4.179854866297 | 1.114380564208 |
| C | -1.939877925370 | 5.501949325454 | 0.789450580815 |
| C | -1.474015481870 | -0.251072803742 | 3.158634369963 |
| C | -1.703375211196 | 1.248642634072 | 3.309734057217 |
| C | -1.157510345507 | 2.229238180959 | 2.204872529516 |
| H | -0.343020397622 | 4.142224764363 | 0.530571212793 |
| H | -1.676879793229 | -0.754814859885 | 2.213998869430 |
| H | -2.776949742039 | 1.446507675430 | 3.459294920038 |
| H | -1.229753210888 | 1.544395485319 | 4.254602475564 |
| H | -0.205295429232 | 1.830663794631 | 1.838965186661 |
| C | -0.953873415303 | 3.729090821793 | 2.592539892002 |
| H | 0.017238150166 | 4.014965672329 | 3.003753757197 |

| | | | |
|---|---|---|---|
| H | -1.731876585049 | 4.062010813505 | 3.285032480766 |
| C | -1.693232135564 | 2.043530167598 | -0.372459248447 |
| H | -0.617405053247 | 1.992713270590 | -0.563006346476 |
| H | -2.085367487387 | 1.020433377567 | -0.369317626332 |
| H | -2.158814478190 | 2.581083838048 | -1.205413969795 |
| C | -3.490985536659 | 2.786207106785 | 1.202629986102 |
| H | -3.907883541821 | 1.774681061564 | 1.173871741684 |
| H | -3.744089457256 | 3.214836785055 | 2.174521525826 |
| H | -3.988432735357 | 3.383462294947 | 0.433324666595 |
| C | -2.936963761296 | 6.118083437426 | 1.721135744620 |
| H | -3.754545083616 | 6.624559722630 | 1.198397933555 |
| H | -3.384532356263 | 5.354277264691 | 2.354295627930 |
| H | -2.472227877120 | 6.862385197969 | 2.388490861564 |
| O | -1.248536423367 | 6.370435561682 | -0.084290333319 |
| O | -1.655383531590 | -1.025134386864 | 4.303783989062 |
| C | -1.903912880024 | 7.609798052227 | -0.488913568777 |
| H | -2.150813890713 | 8.212565126378 | 0.388799136869 |
| H | -2.834502561711 | 7.373333249442 | -1.015572443637 |
| C | -1.022299990681 | 8.496564522363 | -1.418870359525 |
| H | -0.072237576648 | 8.726521259165 | -0.917791098758 |
| H | -0.799972290203 | 7.963755182028 | -2.352909920930 |
| C | -1.541301895033 | -2.471095386226 | 4.164724774359 |
| H | -0.551886121164 | -2.721642891660 | 3.769241616516 |
| H | -2.298820936014 | -2.830943540367 | 3.459426706783 |
| C | -1.738866180796 | -3.201096033276 | 5.528520675655 |
| H | -0.979237583632 | -2.852730079985 | 6.241383931215 |

| | | | |
|---|---|---|---|
| H | -2.729135127624 | -2.953877067620 | 5.934870942240 |
| O | -1.775791421350 | 9.698880121147 | -1.676597507741 |
| C | -1.207066440405 | 10.741263303978 | -2.525465905463 |
| H | -0.276988432948 | 11.136972876775 | -2.099622877519 |
| H | -1.012934483133 | 10.367469520492 | -3.538156402864 |
| H | -1.959542764724 | 11.527403702776 | -2.561111892450 |
| O | -1.616370350798 | -4.616581564931 | 5.295496290684 |
| C | -1.752382043593 | -5.536136750185 | 6.422171085771 |
| H | -0.982782836447 | -5.349823015442 | 7.181003982586 |
| H | -2.744497404359 | -5.455128270706 | 6.882645870178 |
| H | -1.621577524161 | -6.531534164267 | 6.000926212126 |

Pin_TS2A

($E_F$ = -929.70346814 a.u.; $G_F$ = -929.32799679 a.u.)

0 1

| | | | |
|---|---|---|---|
| C | -1.848076562277 | 2.847725521239 | 0.764869255736 |
| C | -1.197477255249 | 4.303286928535 | 1.038862886440 |
| C | -1.905003900091 | 5.610889961714 | 0.726401547789 |
| C | -2.096558757036 | -0.417894531203 | 3.497252889638 |
| C | -1.965768920043 | 0.997722173951 | 3.482708434389 |
| C | -1.090346884865 | 2.333227555185 | 2.029108249852 |
| H | -0.241139917191 | 4.324533428979 | 0.509429842572 |
| H | -2.805476348208 | -0.910837551721 | 2.836878735525 |
| H | -2.913764462660 | 1.489344019268 | 3.288887025551 |
| H | -1.413923866460 | 1.423646422962 | 4.316258483632 |
| H | -0.157608911054 | 1.812822907587 | 1.818263613057 |

| | | | |
|---|---|---|---|
| C | -0.942711393470 | 3.786826780663 | 2.519202431363 |
| H | 0.000112827605 | 4.076048930623 | 2.989258761321 |
| H | -1.761341552393 | 4.077368490691 | 3.186728293308 |
| C | -1.448126643500 | 2.215359589354 | -0.563292963038 |
| H | -0.361549281909 | 2.214270863418 | -0.687104234365 |
| H | -1.797467214309 | 1.178948165769 | -0.622153885799 |
| H | -1.884729253782 | 2.768998658043 | -1.401557205782 |
| C | -3.370692380038 | 2.836420298463 | 0.921532898487 |
| H | -3.749152358802 | 1.813820539655 | 0.832158301611 |
| H | -3.692940606899 | 3.223008823076 | 1.890777151026 |
| H | -3.839065423894 | 3.447678985278 | 0.144709808695 |
| C | -2.951122468777 | 6.171676100806 | 1.637949066872 |
| H | -3.769050176879 | 6.661495897915 | 1.099905855631 |
| H | -3.391253031136 | 5.378201589748 | 2.239690843130 |
| H | -2.534120347880 | 6.916492386657 | 2.335672181629 |
| O | -1.220034406337 | 6.515917775543 | -0.113696589205 |
| O | -1.398015337300 | -1.273311015614 | 4.338558275286 |
| C | -1.907521926835 | 7.735106539991 | -0.526457212399 |
| H | -2.195513141121 | 8.322965049779 | 0.349029436801 |
| H | -2.816575971573 | 7.470987991007 | -1.076967502720 |
| C | -1.032576401987 | 8.654414261092 | -1.430752495136 |
| H | -0.104230788331 | 8.914524701786 | -0.904381845718 |
| H | -0.767976481505 | 8.131254282951 | -2.359235736145 |
| C | -1.870731597795 | -2.657056518254 | 4.375241188690 |
| H | -1.557810123588 | -3.169033440669 | 3.459593021993 |
| H | -2.964075432812 | -2.664944131018 | 4.422849284281 |

| | | | |
|---|---|---|---|
| C | -1.329913396769 | -3.448976066524 | 5.604205025878 |
| H | -0.232622488733 | -3.482755306166 | 5.573823861716 |
| H | -1.633605958264 | -2.940220004029 | 6.529009928915 |
| O | -1.818018362570 | 9.831770300989 | -1.706887698232 |
| C | -1.260335015254 | 10.893622148717 | -2.538892419249 |
| H | -0.355149478149 | 11.317898372126 | -2.087673675197 |
| H | -1.027218188889 | 10.528224079424 | -3.546399704739 |
| H | -2.036311473548 | 11.655417133650 | -2.593866581898 |
| O | -1.889504642367 | -4.774310513237 | 5.534580423675 |
| C | -1.562136422386 | -5.746215179053 | 6.574775176597 |
| H | -0.484411169091 | -5.946738410120 | 6.605254018614 |
| H | -1.896141761992 | -5.399717826624 | 7.560208778543 |
| H | -2.098480844983 | -6.653338415363 | 6.301360668095 |

Pin_TS2B

($E_F$ = -929.70735797 a.u.; $G_F$ = -929.33010741 a.u.)

0 1

| | | | |
|---|---|---|---|
| C | -1.584497735490 | 2.814512200501 | 1.098620055691 |
| C | -1.348203397093 | 4.384852982751 | 1.132144359061 |
| C | -2.004328008086 | 5.430755755597 | 0.463890811064 |
| C | -1.425434438033 | -0.135258933699 | 3.360102101717 |
| C | -2.063999475720 | 1.245731723096 | 3.357032164559 |
| C | -1.361973358098 | 2.476480021317 | 2.631234838907 |
| H | -0.289599062883 | 4.600362642378 | 1.271750984315 |
| H | -1.051328945182 | -0.594640803946 | 2.446110655034 |
| H | -2.159813499264 | 1.538661775910 | 4.409945914064 |

| | | | |
|---|---|---|---|
| H | -3.101254285684 | 1.176103337280 | 2.994741646952 |
| H | -0.283000272605 | 2.374188416948 | 2.789174711079 |
| C | -1.798418731662 | 3.830196783803 | 3.166348378160 |
| H | -1.198358224530 | 4.345505647261 | 3.909998870833 |
| H | -2.868672610006 | 4.004776470688 | 3.243828967271 |
| C | -0.531104671118 | 2.163034576547 | 0.189237657104 |
| H | 0.480543988852 | 2.445758132208 | 0.493151454053 |
| H | -0.604924897667 | 1.071355184912 | 0.237288645569 |
| H | -0.670547539259 | 2.469958788856 | -0.851933179110 |
| C | -2.972216109972 | 2.369097792332 | 0.627764744995 |
| H | -3.174634583357 | 2.719386894795 | -0.386964571844 |
| H | -3.017810903610 | 1.276602875993 | 0.617285757994 |
| H | -3.772495074501 | 2.721205498395 | 1.281998019656 |
| C | -3.479484898901 | 5.503816803790 | 0.201031110551 |
| H | -3.720114657259 | 5.629678494363 | -0.859795357658 |
| H | -3.986970370257 | 4.615639994696 | 0.558205327382 |
| H | -3.911204689639 | 6.359908443796 | 0.733859525079 |
| O | -1.289012265542 | 6.629312545365 | 0.225886745121 |
| O | -1.898877504774 | -1.018415595835 | 4.330220841065 |
| C | -1.830682222395 | 7.595778343001 | -0.725188153548 |
| H | -2.828907153853 | 7.919454232242 | -0.421650537072 |
| H | -1.906422471210 | 7.131433863803 | -1.713810908467 |
| C | -0.961280826245 | 8.882163339519 | -0.857162831791 |
| H | -0.853543097915 | 9.358777856440 | 0.126407280134 |
| H | 0.039663392094 | 8.629268728556 | -1.230804717333 |
| C | -1.389437702874 | -2.382707869454 | 4.319475202195 |

| | | | |
|---|---|---|---|
| H | -0.301805417872 | -2.366890489473 | 4.441643259580 |
| H | -1.624689722303 | -2.854100518256 | 3.358637303775 |
| C | -2.015023868229 | -3.236621452760 | 5.464876079521 |
| H | -1.779451061509 | -2.774090600332 | 6.432899242826 |
| H | -3.107446965342 | -3.254179083613 | 5.350527562136 |
| O | -1.654695357236 | 9.747296715913 | -1.779603764552 |
| C | -1.088547469022 | 11.049328212392 | -2.118762821469 |
| H | -0.994242828410 | 11.685241982173 | -1.230182095687 |
| H | -0.106796099478 | 10.943768589947 | -2.596103346391 |
| H | -1.792299473894 | 11.496592791661 | -2.818883422858 |
| O | -1.467170549078 | -4.565310307341 | 5.381890025016 |
| C | -1.889846160947 | -5.568368818334 | 6.355903483332 |
| H | -1.630639439417 | -5.263315496934 | 7.376921600586 |
| H | -2.969488927710 | -5.750985686678 | 6.293869408239 |
| H | -1.346848221460 | -6.474590284863 | 6.092763625634 |

Pin_Int2B

($E_F$ = -929.73653376 a.u.; $G_F$ = -929.36088970 a.u.)

0 1

| | | | |
|---|---|---|---|
| C | -1.789187952283 | 2.958825577631 | 1.007588038429 |
| C | -1.290380002937 | 4.440206765462 | 0.825170565293 |
| C | -1.925287640995 | 5.609804298213 | 0.566660137716 |
| C | -1.344704921343 | -0.047731617923 | 3.742085579288 |
| C | -1.896562354456 | 0.735786230506 | 2.560190549398 |
| C | -1.414646419055 | 2.238907457864 | 2.432262007734 |
| H | -0.212792753688 | 4.492303572489 | 0.677620968704 |

| | | | |
|---|---|---|---|
| H | -0.308701130641 | 0.076904477882 | 4.054466231338 |
| H | -2.990360533451 | 0.726643285311 | 2.638128599811 |
| H | -1.663429123282 | 0.189594889637 | 1.635824365022 |
| H | -0.312947955763 | 2.236016804123 | 2.450316280835 |
| C | -1.915041650521 | 3.025740423627 | 3.589272988440 |
| H | -1.611206164381 | 4.057948655645 | 3.716383532298 |
| H | -2.665652228163 | 2.622794100778 | 4.257454605219 |
| C | -1.030920617458 | 2.203169270546 | -0.114263102605 |
| H | 0.050710240187 | 2.242991943276 | 0.044046343234 |
| H | -1.328172376968 | 1.153358387136 | -0.161828817302 |
| H | -1.244888882362 | 2.655693539340 | -1.086062397422 |
| C | -3.296373876019 | 2.778480905090 | 0.756597894973 |
| H | -3.609748610228 | 3.304813807805 | -0.147270277224 |
| H | -3.528023505575 | 1.720585360412 | 0.615977536406 |
| H | -3.897040366237 | 3.133325088710 | 1.595847699641 |
| C | -3.384842628830 | 5.911273952560 | 0.779100173266 |
| H | -3.937713487484 | 6.010179071747 | -0.160151071709 |
| H | -3.863341500294 | 5.145738353783 | 1.378980091024 |
| H | -3.477860917492 | 6.859046317270 | 1.317552077931 |
| O | -1.158681091933 | 6.733127919885 | 0.158220065410 |
| O | -1.894129500514 | -1.309066371318 | 3.951611680152 |
| C | -1.752455897653 | 7.681117367289 | -0.780019835510 |
| H | -2.718888736147 | 8.038514884369 | -0.417473644495 |
| H | -1.909237931572 | 7.187431493877 | -1.745132875283 |
| C | -0.863805179088 | 8.942543986392 | -1.000755230152 |
| H | -0.666333908583 | 9.422443760563 | -0.032527826644 |

| | | | |
|---|---|---|---|
| H | 0.098007256006 | 8.657514975092 | -1.447131769355 |
| C | -1.300891522668 | -2.147314114033 | 4.986346689662 |
| H | -1.370790109175 | -1.636863579414 | 5.951918153487 |
| H | -0.242855254609 | -2.315518391724 | 4.756998467319 |
| C | -2.018700151579 | -3.527835781424 | 5.090035612622 |
| H | -3.079916362112 | -3.369309484808 | 5.325134176640 |
| H | -1.955492528418 | -4.044976813593 | 4.122838922210 |
| O | -1.598177590904 | 9.822969370569 | -1.875094275181 |
| C | -1.014426501632 | 11.102319025900 | -2.266938210177 |
| H | -0.831499985876 | 11.739668363607 | -1.393271121036 |
| H | -0.075498064701 | 10.959432638620 | -2.815472911391 |
| H | -1.752653724455 | 11.570266090070 | -2.916209178932 |
| O | -1.369616580792 | -4.289272523923 | 6.124906187307 |
| C | -1.858257948443 | -5.631432282666 | 6.430932597984 |
| H | -2.901602422472 | -5.606127389308 | 6.767835357174 |
| H | -1.773050685827 | -6.292431404155 | 5.559923065232 |
| H | -1.220006505961 | -5.995734302650 | 7.234201549094 |

Pin_TS3B

($E_F$ = -929.72655743 a.u.; $G_F$ = -929.35237684 a.u.)

0 1

| | | | |
|---|---|---|---|
| C | -1.776130576749 | 3.082665751544 | 0.872872328677 |
| C | -1.274421515748 | 4.508718578089 | 0.653544836397 |
| C | -1.905830168104 | 5.702095457462 | 0.464342561984 |
| C | -1.355902898008 | -0.145108415935 | 3.877727471815 |
| C | -1.862631528353 | 0.680571540939 | 2.695544932544 |

| | | | |
|---|---|---|---|
| C | -1.316749676794 | 2.144611544646 | 2.640804362943 |
| H | -0.217633936680 | 4.546249030229 | 0.394780667708 |
| H | -0.348883173543 | 0.007893604148 | 4.262053302175 |
| H | -2.957875225290 | 0.715333934398 | 2.752247604780 |
| H | -1.631260667096 | 0.148785821356 | 1.761881077288 |
| H | -0.240126352411 | 2.182176133859 | 2.467070138960 |
| C | -1.779212440881 | 2.981757579910 | 3.688771555639 |
| H | -1.312305339938 | 3.940357528208 | 3.882457603674 |
| H | -2.718730103770 | 2.773567142190 | 4.188694309413 |
| C | -1.019073506458 | 2.195343916317 | -0.118622760841 |
| H | 0.062301409261 | 2.253518643884 | 0.035379622497 |
| H | -1.319525073749 | 1.149660622485 | -0.035598560434 |
| H | -1.221856037629 | 2.517938397428 | -1.147683969723 |
| C | -3.276399467765 | 2.854250188843 | 0.787707765579 |
| H | -3.697777098151 | 3.326767451659 | -0.107026419255 |
| H | -3.493429364880 | 1.786019578091 | 0.727494952253 |
| H | -3.801640892313 | 3.243148471044 | 1.661995949451 |
| C | -3.330455601600 | 6.030658312606 | 0.811507816888 |
| H | -3.940901773148 | 6.232604493361 | -0.074063783443 |
| H | -3.795196705706 | 5.229333474978 | 1.373646102562 |
| H | -3.352940089474 | 6.929257224009 | 1.435812954294 |
| O | -1.152673099362 | 6.790643169605 | -0.031037188035 |
| O | -1.872471099868 | -1.429778155526 | 4.022075848489 |
| C | -1.833452068662 | 7.845894292802 | -0.777847958590 |
| H | -2.608935355442 | 8.314480405607 | -0.167391260577 |
| H | -2.306388342722 | 7.417744211751 | -1.667513256927 |

| | | | |
|---|---|---|---|
| C | -0.865728670696 | 8.979875928170 | -1.231886328469 |
| H | -0.360257575525 | 9.404928738966 | -0.354272023204 |
| H | -0.101529279277 | 8.577224633358 | -1.909632603184 |
| C | -1.325152368927 | -2.251259686491 | 5.095799319306 |
| H | -1.470483689378 | -1.740449367238 | 6.052703763566 |
| H | -0.250197087617 | -2.389903527877 | 4.935426096976 |
| C | -2.006387422569 | -3.652089146594 | 5.163949289414 |
| H | -3.084316794308 | -3.525766466990 | 5.333141620888 |
| H | -1.868125233063 | -4.173425478532 | 4.206914875373 |
| O | -1.675062669950 | 9.971490252775 | -1.894926056848 |
| C | -1.045711860800 | 11.170378769744 | -2.441397469545 |
| H | -0.557780690649 | 11.756849812683 | -1.653519107781 |
| H | -0.310069963169 | 10.915801923023 | -3.213892981370 |
| H | -1.857396303841 | 11.747474332257 | -2.881340476084 |
| O | -1.397610526623 | -4.385739247784 | 6.242953631599 |
| C | -1.862124459512 | -5.739623087495 | 6.533459429033 |
| H | -2.923438792583 | -5.743908755606 | 6.809926402746 |
| H | -1.707062866467 | -6.404796608755 | 5.675308264258 |
| H | -1.260395982713 | -6.077699641497 | 7.375481901285 |

Pin_TS2C

($E_F$ = -929.72195533 a.u.; $G_F$ = -929.34517280 a.u.)

0 1

| | | | |
|---|---|---|---|
| C | -2.505246204207 | 2.705838272214 | 1.175186620126 |
| C | -1.200009461543 | 4.282708807425 | 0.933124965452 |
| C | -1.471400233660 | 5.675902481456 | 1.045715600589 |

| | | | |
|---|---|---|---|
| C | -1.591289731692 | -0.404011216189 | 2.784132505950 |
| C | -1.325477340663 | 1.075552691884 | 3.033824570794 |
| C | -1.244447988224 | 2.062498732412 | 1.801181679238 |
| H | -0.837984033277 | 4.013029293017 | -0.058472897860 |
| H | -2.382387185887 | -0.720729416482 | 2.105049540976 |
| H | -2.068719320061 | 1.472891535480 | 3.744540307727 |
| H | -0.365422139014 | 1.141077410651 | 3.561182913265 |
| H | -0.696522057461 | 1.548185368791 | 1.003877635248 |
| C | -0.553851728611 | 3.433105822746 | 2.061654331566 |
| H | 0.542307599413 | 3.421485471605 | 2.039219857860 |
| H | -0.848145056265 | 3.808703478395 | 3.045731451576 |
| C | -3.000017809377 | 2.160139484242 | -0.136984949077 |
| H | -3.522326051223 | 1.200423634363 | 0.005095987782 |
| H | -3.707954672863 | 2.849079799908 | -0.608860861258 |
| H | -2.177618747429 | 1.983588721337 | -0.835936868227 |
| C | -3.597278534502 | 3.123147032825 | 2.125459241559 |
| H | -4.209382376412 | 2.258618341030 | 2.423595193295 |
| H | -3.207064613377 | 3.572057687853 | 3.041850227989 |
| H | -4.264250812277 | 3.850049647264 | 1.652586508277 |
| C | -1.831303715775 | 6.347578393184 | 2.335994570971 |
| H | -2.801558573960 | 6.854864179428 | 2.281615996019 |
| H | -1.893662970120 | 5.615362681794 | 3.138712297960 |
| H | -1.092908678587 | 7.101872318206 | 2.636089766491 |
| O | -1.428813042728 | 6.477150188988 | -0.114809663987 |
| O | -1.324190131094 | -1.273139869188 | 3.841047205001 |
| C | -1.836955580682 | 7.876335358856 | -0.055281908415 |

| | | | |
|---|---|---|---|
| H | -1.369419480595 | 8.377275161395 | 0.795141959642 |
| H | -2.923338099789 | 7.939692318713 | 0.063510634259 |
| C | -1.435164536451 | 8.676510238426 | -1.331426336502 |
| H | -0.348482351848 | 8.612774970193 | -1.477594077935 |
| H | -1.928185560070 | 8.252804867821 | -2.216381158441 |
| C | -1.665518698787 | -2.678059553120 | 3.659394315865 |
| H | -1.130437841088 | -3.072847199625 | 2.789934836843 |
| H | -2.741941663256 | -2.771519587851 | 3.476767941502 |
| C | -1.293399609216 | -3.531315981853 | 4.910397099752 |
| H | -0.214143159374 | -3.450845349895 | 5.098307243445 |
| H | -1.824395005512 | -3.141464935429 | 5.789565639490 |
| O | -1.849519644021 | 10.039850789878 | -1.111856805930 |
| C | -1.598381064626 | 11.045498787975 | -2.140040965132 |
| H | -0.524522703193 | 11.155244804609 | -2.334197485644 |
| H | -2.113193878877 | 10.794553192569 | -3.075356745119 |
| H | -1.997155077590 | 11.975474098726 | -1.738006141840 |
| O | -1.669914568715 | -4.895324229989 | 4.644202706168 |
| C | -1.419578006414 | -5.912373489976 | 5.662225057166 |
| H | -0.348609373078 | -5.997030411223 | 5.883008631259 |
| H | -1.965288256658 | -5.690249817900 | 6.587337580813 |
| H | -1.782866903373 | -6.845502677555 | 5.234766140312 |

Pin_Int2C

($E_F$ = -929.75854409 a.u.; $G_F$ = -929.38499278 a.u.)

0 1

| | | | |
|---|---|---|---|
| C | -2.945408966763 | 1.798368542371 | 1.207824609181 |

| | | | |
|---|---|---|---|
| C | -1.431199102167 | 4.555399235942 | 0.879593726052 |
| C | -1.299985953680 | 5.899890373180 | 0.986673306168 |
| C | -1.510952357712 | -0.397334742350 | 2.829596189899 |
| C | -1.310415023996 | 1.097005248979 | 3.034678379478 |
| C | -1.553659720016 | 2.001388028638 | 1.746175339146 |
| H | -1.642207051711 | 4.155620282493 | -0.108134150517 |
| H | -2.233690436456 | -0.752421257327 | 2.096326192852 |
| H | -1.990026091496 | 1.457046118177 | 3.824464400334 |
| H | -0.297735882071 | 1.273972095014 | 3.419208287371 |
| H | -0.837895309870 | 1.668975075731 | 0.986177938360 |
| C | -1.251902567804 | 3.534034653014 | 2.039700895919 |
| H | -0.222373031584 | 3.591720370573 | 2.414580869692 |
| H | -1.890460367014 | 3.829093263930 | 2.880062856883 |
| C | -3.145698449479 | 1.415302785841 | -0.222812788769 |
| H | -4.101970683191 | 0.902241617629 | -0.374155535382 |
| H | -3.158870987448 | 2.297900443153 | -0.886236803351 |
| H | -2.344542423559 | 0.763074366562 | -0.584357721323 |
| C | -4.109483459724 | 2.351453169833 | 1.967006750003 |
| H | -5.035927506327 | 1.822027895273 | 1.723041852966 |
| H | -3.962377182061 | 2.295991982176 | 3.050077191704 |
| H | -4.277138811137 | 3.414843366589 | 1.726240576068 |
| C | -0.989720402942 | 6.591597316966 | 2.286402255100 |
| H | -1.761647064039 | 7.318780662299 | 2.554665779791 |
| H | -0.913471851850 | 5.871766764028 | 3.098758162565 |
| H | -0.038600081141 | 7.130653528330 | 2.226640045717 |
| O | -1.381862872872 | 6.725572075005 | -0.154853764641 |

| | | | |
|---|---|---|---|
| O | -1.281412978238 | -1.237731958653 | 3.915308722829 |
| C | -1.575558158029 | 8.164115267373 | -0.003642719447 |
| H | -0.825726873928 | 8.587821641900 | 0.668196891396 |
| H | -2.566764695432 | 8.362504304621 | 0.416827201341 |
| C | -1.453341705803 | 8.928564019931 | -1.356545818175 |
| H | -0.472609964578 | 8.722908455719 | -1.806227447566 |
| H | -2.230934284504 | 8.591596897343 | -2.054624390444 |
| C | -1.533337594725 | -2.659835371432 | 3.714026012394 |
| H | -0.899141268792 | -3.029079738768 | 2.901976859873 |
| H | -2.581382712291 | -2.806314403041 | 3.429783132920 |
| C | -1.242849465261 | -3.493712835527 | 4.998889705281 |
| H | -0.191987409670 | -3.362081802894 | 5.290308593919 |
| H | -1.874808909874 | -3.131492532168 | 5.821212062929 |
| O | -1.601643130800 | 10.330437346242 | -1.054656733648 |
| C | -1.529523267626 | 11.312931632592 | -2.132264804449 |
| H | -0.553403778302 | 11.283799611196 | -2.631434916346 |
| H | -2.321685826915 | 11.149188292360 | -2.872840163650 |
| H | -1.668878411421 | 12.279791448985 | -1.651374773642 |
| O | -1.526116047716 | -4.873767321110 | 4.700717632665 |
| C | -1.329109824187 | -5.880913609682 | 5.739886062307 |
| H | -0.282229100035 | -5.915719538925 | 6.064998056205 |
| H | -1.972895641021 | -5.687837074073 | 6.606599176746 |
| H | -1.604135926783 | -6.828800901403 | 5.280220396147 |

Pin_TS3CA

($E_F$ = -929.74236173 a.u.; $G_F$ = -929.37056219 a.u.)

0 1

| | | | |
|---|---|---|---|
| C | -1.564257565763 | 1.566081493135 | 0.930735763853 |
| C | -1.311616132819 | 5.101620811770 | 1.595332741718 |
| C | -1.886924539890 | 5.903818994892 | 0.648733694532 |
| C | -1.426339867241 | -0.252730739472 | 3.515401782342 |
| C | -1.886866013377 | 1.205070041532 | 3.451044189439 |
| C | -1.272954035062 | 1.993899418029 | 2.254594923269 |
| H | -0.323093582553 | 5.420203470782 | 1.920077767383 |
| H | -0.417809893860 | -0.509136800621 | 3.196007440265 |
| H | -1.626506550164 | 1.723871860096 | 4.386854662497 |
| H | -2.981733699076 | 1.228626231237 | 3.403077609595 |
| H | -0.217415662393 | 2.210168882631 | 2.419418826237 |
| C | -1.902451810638 | 3.959215408634 | 2.352739624764 |
| H | -1.691228740828 | 4.049958412426 | 3.417305861506 |
| H | -2.964047174971 | 3.794285276389 | 2.184909512970 |
| C | -0.728166139772 | 2.093414126545 | -0.196585719969 |
| H | 0.287852265665 | 2.336893882252 | 0.123881842983 |
| H | -0.669583130884 | 1.377573831316 | -1.023718505330 |
| H | -1.164674601628 | 3.019620746208 | -0.603222064944 |
| C | -2.895243440646 | 0.989937430439 | 0.548878721416 |
| H | -3.577644507955 | 1.781848248578 | 0.195646546070 |
| H | -2.791956820061 | 0.282745431171 | -0.280932897136 |
| H | -3.388280026814 | 0.470373613279 | 1.371018911316 |
| C | -3.230574663810 | 5.648288377612 | 0.031164158469 |
| H | -3.140414381960 | 5.553715529790 | -1.056430598939 |
| H | -3.659701990886 | 4.722959083621 | 0.411191929996 |

| | | | |
|---|---|---|---|
| H | -3.939826779661 | 6.457347789765 | 0.231374778958 |
| O | -1.173310355345 | 7.015462105840 | 0.161386333282 |
| O | -1.981077810469 | -1.084715872797 | 4.484253706467 |
| C | -1.896282582544 | 8.143592434495 | -0.417550625750 |
| H | -2.566453526227 | 8.576462413796 | 0.332091176009 |
| H | -2.496328339495 | 7.819275993728 | -1.271687117007 |
| C | -0.934238956247 | 9.261457167361 | -0.921659288179 |
| H | -0.344846264250 | 9.655031511999 | -0.082940279193 |
| H | -0.241304818075 | 8.841808523923 | -1.663404129042 |
| C | -1.446881348899 | -2.440780775476 | 4.559737504441 |
| H | -0.375016707445 | -2.395918605138 | 4.782760937969 |
| H | -1.579180255406 | -2.934976760370 | 3.592169916953 |
| C | -2.149136282268 | -3.294269459682 | 5.659119727975 |
| H | -2.024701679838 | -2.808941844614 | 6.636834323940 |
| H | -3.224043019128 | -3.358931811746 | 5.442190403889 |
| O | -1.755697985291 | 10.291733101923 | -1.505049983630 |
| C | -1.124231839453 | 11.482695623199 | -2.066602110324 |
| H | -0.562473125939 | 12.032200444374 | -1.301587634582 |
| H | -0.453281963409 | 11.223230671282 | -2.894453406836 |
| H | -1.943615462260 | 12.097730029142 | -2.435043525363 |
| O | -1.541686595954 | -4.600069356348 | 5.650743109282 |
| C | -2.021633334965 | -5.615732578915 | 6.584049559135 |
| H | -1.883405504691 | -5.295968227860 | 7.624096873151 |
| H | -3.080384095507 | -5.844350334571 | 6.411403699064 |
| H | -1.416664965839 | -6.499168456306 | 6.386467214593 |

Pin_TS3CC

($E_F$ = -929.75335703 a.u.; $G_F$ = -929.37974463 a.u.)

0 1

| | | | |
|---|---|---|---|
| C | -2.827665553100 | 1.830202412640 | 0.934812610608 |
| C | -1.410604074519 | 4.672638553213 | 0.817496926800 |
| C | -1.300012779696 | 6.014884428997 | 0.963402802164 |
| C | -1.925465576468 | -0.241631434204 | 3.198284457393 |
| C | -1.398130997691 | 1.140680185062 | 3.066386109999 |
| C | -1.553535454300 | 2.112333821506 | 1.596383923825 |
| H | -1.570880258066 | 4.290093480010 | -0.186098982222 |
| H | -2.995498486999 | -0.392733278055 | 3.316646570427 |
| H | -1.929337177671 | 1.795366699453 | 3.766989321621 |
| H | -0.333310584410 | 1.160347952607 | 3.306934903003 |
| H | -0.722624191082 | 1.745970233038 | 0.986875649246 |
| C | -1.310051370277 | 3.620930415328 | 1.963569361288 |
| H | -0.320076996000 | 3.687348950588 | 2.430305131818 |
| H | -2.022262541018 | 3.887089816049 | 2.753384738887 |
| C | -2.934359877690 | 0.512822237938 | 0.258802906104 |
| H | -3.892479008018 | 0.363806104263 | -0.244848681856 |
| H | -2.126907752172 | 0.350374826856 | -0.463438256375 |
| H | -2.811363217615 | -0.297808022954 | 1.016908987515 |
| C | -4.074932179657 | 2.529962204378 | 1.384386624921 |
| H | -4.925007509823 | 2.281103039854 | 0.744575401847 |
| H | -4.352585672496 | 2.245419051063 | 2.414775572577 |
| H | -3.960309385993 | 3.618065078610 | 1.383359432249 |
| C | -1.070992347647 | 6.680317184619 | 2.293259859807 |

| | | | |
|---|---|---|---|
| H | -1.871942952636 | 7.383571497463 | 2.539899169909 |
| H | -1.019048314852 | 5.942053596801 | 3.091067111445 |
| H | -0.130911253217 | 7.241607755020 | 2.294102508025 |
| O | -1.326672475777 | 6.865440204923 | -0.162823249509 |
| O | -1.143135415395 | -1.291415347625 | 3.676634783510 |
| C | -1.575917384007 | 8.292975965320 | 0.005576450003 |
| H | -0.884412388366 | 8.724778930725 | 0.732817120083 |
| H | -2.597898217996 | 8.451174718512 | 0.365318239102 |
| C | -1.392193761453 | 9.088950112257 | -1.321992197119 |
| H | -0.378951929291 | 8.921670453853 | -1.711599324693 |
| H | -2.112947676173 | 8.742564152835 | -2.074350882793 |
| C | -1.844009373778 | -2.552773453583 | 3.893664607916 |
| H | -2.114482126450 | -2.985200310226 | 2.924676665416 |
| H | -2.764865060307 | -2.363628403817 | 4.456349075540 |
| C | -0.994371750963 | -3.588479626124 | 4.690694292781 |
| H | -0.074589957048 | -3.820849943354 | 4.137230391274 |
| H | -0.712561209681 | -3.160794611514 | 5.662512273369 |
| O | -1.602834404322 | 10.479737472520 | -1.005890376648 |
| C | -1.493411369769 | 11.483493199634 | -2.060462915831 |
| H | -0.487112131063 | 11.493036389206 | -2.496606542899 |
| H | -2.231428768827 | 11.309352190615 | -2.852862782737 |
| H | -1.693281585167 | 12.436843528518 | -1.573890530344 |
| O | -1.805888646026 | -4.766422410188 | 4.860457639239 |
| C | -1.261353402433 | -5.912642763049 | 5.583440845453 |
| H | -0.369969244418 | -6.310176420130 | 5.083236874822 |
| H | -1.010296800394 | -5.646558370450 | 6.617410340953 |

| | | | |
|---|---|---|---|
| H | -2.052334442362 | -6.660755938477 | 5.576870330515 |

Cam_TS2C-F2500

($E_F$ = -929.76455870 a.u.; $G_F$ = -929.38644387 a.u.)

0 1

| | | | |
|---|---|---|---|
| C | -1.200454790888 | 1.563772228832 | -2.723142087807 |
| C | -0.501223487509 | 2.418868652381 | -3.624259329506 |
| C | -3.380402624182 | 1.813339024476 | 1.015657687478 |
| C | -2.789097329404 | 1.332150256233 | -0.312673083978 |
| C | -1.308390026635 | 0.799884198511 | -0.222715746022 |
| C | -0.435160520323 | 1.261139589533 | -1.423044822863 |
| H | 0.166078653322 | 3.190749861955 | -3.250175151630 |
| H | -2.962569102792 | 2.696444972915 | 1.496050553471 |
| H | -3.421460900201 | 0.498958514555 | -0.635659594402 |
| H | -0.842319939390 | 1.139856550683 | 0.706087099987 |
| H | -1.333292152475 | -0.291306435590 | -0.166261644857 |
| H | 0.322539341143 | 0.493715393635 | -1.628231344745 |
| H | 0.123181128127 | 2.158505751991 | -1.140622402982 |
| C | -2.803669822798 | 2.390656581691 | -1.449815207930 |
| C | -4.052481676170 | 2.440493144358 | -2.289691998314 |
| H | -3.904498733657 | 3.072391878719 | -3.171032798696 |
| H | -4.359056067224 | 1.447651815416 | -2.627587172269 |
| H | -4.890437592906 | 2.863927237100 | -1.716065747704 |
| C | -2.276725532517 | 3.757127482062 | -1.077234536872 |
| H | -2.052061313942 | 4.339709209462 | -1.975320680528 |
| H | -3.023096377556 | 4.317253968617 | -0.497089941407 |

| | | | |
|---|---|---|---|
| H | -1.369046153027 | 3.704512361616 | -0.470909663117 |
| O | -0.532679304879 | 2.296112120578 | -5.012595377949 |
| O | -3.999486761954 | 0.909558667442 | 1.880504951267 |
| C | -1.883677075419 | 0.370751075651 | -3.367642489846 |
| H | -1.137334383394 | -0.309572524239 | -3.795697667572 |
| H | -2.473842546566 | -0.195174813475 | -2.645089173191 |
| H | -2.543297396889 | 0.682375466550 | -4.179452790845 |
| C | 0.418769311016 | 3.115703116860 | -5.764138059640 |
| H | 1.409431259217 | 3.029232387192 | -5.306732575700 |
| H | 0.104944511969 | 4.163577303526 | -5.720193819008 |
| C | 0.535285206983 | 2.688129250901 | -7.263287260000 |
| H | 0.848040499153 | 1.636852134854 | -7.318081169787 |
| H | -0.443764030219 | 2.782162795478 | -7.751594122980 |
| C | -4.478576441196 | 1.456308622340 | 3.150882331999 |
| H | -5.205304414693 | 2.248958018269 | 2.947628919367 |
| H | -3.633736348631 | 1.891836149393 | 3.695550530928 |
| C | -5.152563988671 | 0.381142377846 | 4.063427492201 |
| H | -6.014808803541 | -0.054999442295 | 3.541657277644 |
| H | -4.435027463778 | -0.423111183023 | 4.274497304149 |
| O | 1.508589784883 | 3.550470303558 | -7.887998379945 |
| C | 1.833851585937 | 3.374862499174 | -9.306390030953 |
| H | 2.249763904829 | 2.378343917029 | -9.496252333647 |
| H | 0.950171966601 | 3.528499689195 | -9.936905428038 |
| H | 2.581098121933 | 4.135857104384 | -9.524205802186 |
| O | -5.564227632217 | 1.037663359585 | 5.280780924506 |
| C | -6.241409797212 | 0.270121007335 | 6.329499966912 |

| | | | |
|---|---|---|---|
| H | -7.184396268926 | -0.153177149182 | 5.963794775206 |
| H | -5.600879304704 | -0.536012277427 | 6.706432857678 |
| H | -6.440938694855 | 0.986688384142 | 7.124318438689 |

Cam_TS2C-F2000

($E_F$ = -929.56042494 a.u.; $G_F$ = -929.18201689 a.u.)

0 1

| | | | |
|---|---|---|---|
| C | -1.168254593137 | 1.524125234389 | -2.703590619175 |
| C | -0.479043359561 | 2.384464936507 | -3.586506426078 |
| C | -3.384752507891 | 1.839528398772 | 0.983227203631 |
| C | -2.790172710765 | 1.340778729153 | -0.325703717996 |
| C | -1.322870136780 | 0.781613540842 | -0.207623995408 |
| C | -0.425951844075 | 1.223663765750 | -1.393745333643 |
| H | 0.196165748792 | 3.147924971118 | -3.209748936670 |
| H | -2.969844365395 | 2.727128141906 | 1.458093964099 |
| H | -3.432650911559 | 0.516274082731 | -0.651459454140 |
| H | -0.866782765799 | 1.115279348005 | 0.728411658864 |
| H | -1.369458550045 | -0.308767048116 | -0.148890457214 |
| H | 0.324459395410 | 0.445235873583 | -1.583544408401 |
| H | 0.139630289244 | 2.115846915014 | -1.109449300435 |
| C | -2.787665480034 | 2.398342032834 | -1.453452300733 |
| C | -4.019239646212 | 2.460886790671 | -2.316059133406 |
| H | -3.841710886985 | 3.077052661604 | -3.203288379617 |
| H | -4.339717015454 | 1.469835292619 | -2.646120959430 |
| H | -4.859648223004 | 2.910092623044 | -1.765782470095 |
| C | -2.237825623848 | 3.756478908154 | -1.087534752592 |

| | | | |
|---|---|---|---|
| H | -1.986752145027 | 4.323867629223 | -1.988514082514 |
| H | -2.980612645375 | 4.340385989884 | -0.526296579999 |
| H | -1.340918041271 | 3.691732902496 | -0.466539514547 |
| O | -0.545487055024 | 2.290114768147 | -4.970297594631 |
| O | -3.987729756454 | 0.933954833253 | 1.848098557171 |
| C | -1.868640191857 | 0.342615659903 | -3.348963540438 |
| H | -1.131128288028 | -0.356060196376 | -3.762658281852 |
| H | -2.479381983970 | -0.206863404921 | -2.630911409501 |
| H | -2.510654341030 | 0.663123497906 | -4.171064828028 |
| C | 0.370272691372 | 3.138032085351 | -5.717085984224 |
| H | 1.364722593002 | 3.094781764220 | -5.261005143794 |
| H | 0.017745876413 | 4.174326058073 | -5.684652224939 |
| C | 0.496189482850 | 2.692395687667 | -7.199072063348 |
| H | 0.845531903674 | 1.651599660522 | -7.239231726754 |
| H | -0.485367578375 | 2.744316883553 | -7.689556864257 |
| C | -4.475991170229 | 1.487735796434 | 3.101753490591 |
| H | -5.218874329710 | 2.263589859085 | 2.890401216620 |
| H | -3.642566291473 | 1.946032545511 | 3.646171225715 |
| C | -5.122929631557 | 0.406069148455 | 4.007684794982 |
| H | -5.974462051250 | -0.052710347970 | 3.486841889644 |
| H | -4.388746936669 | -0.381826742068 | 4.225424232658 |
| O | 1.435456526732 | 3.577891136174 | -7.829081431865 |
| C | 1.752483073626 | 3.368912272635 | -9.232220280545 |
| H | 2.197441164736 | 2.379606132979 | -9.396010564909 |
| H | 0.859885504883 | 3.472753803912 | -9.861486054411 |
| H | 2.474187219458 | 4.142828809563 | -9.488986767011 |

| | | | |
|---|---|---|---|
| O | -5.548175721853 | 1.062390402625 | 5.212997054838 |
| C | -6.203252140046 | 0.270513483465 | 6.240552107575 |
| H | -7.130790210460 | -0.179424395160 | 5.865156825893 |
| H | -5.542573629374 | -0.521753689858 | 6.614179298841 |
| H | -6.433198236829 | 0.966243367520 | 7.046123736025 |

Cam_TS2C-F1500

($E_F$ = -929.35858975 a.u.; $G_F$ = -928.97966773 a.u.)

0 1

| | | | |
|---|---|---|---|
| C | -1.129597695457 | 1.474598945564 | -2.678865719503 |
| C | -0.449854524859 | 2.339750956526 | -3.545159823297 |
| C | -3.388996029690 | 1.872025602378 | 0.947618737626 |
| C | -2.787354594313 | 1.353883661535 | -0.339987865089 |
| C | -1.336371836230 | 0.765596142045 | -0.185937847869 |
| C | -0.412035878347 | 1.175579163563 | -1.359305832750 |
| H | 0.235474353297 | 3.093367270072 | -3.167427799824 |
| H | -2.975987876545 | 2.763044087576 | 1.418017206678 |
| H | -3.439977006223 | 0.539219624878 | -0.670706024262 |
| H | -0.891966581132 | 1.100687893352 | 0.755220929751 |
| H | -1.407725686078 | -0.322746746328 | -0.115338622389 |
| H | 0.324269718848 | 0.379648911027 | -1.530583251905 |
| H | 0.167720818511 | 2.059005192338 | -1.076321460920 |
| C | -2.760857274980 | 2.409191039563 | -1.459616592623 |
| C | -3.972101248319 | 2.488937183583 | -2.347683308640 |
| H | -3.759017612713 | 3.083900473213 | -3.241821681656 |
| H | -4.312633787720 | 1.501218028769 | -2.667382823347 |

| | | | |
|---|---|---|---|
| H | -4.811890624012 | 2.971459726946 | -1.825062401718 |
| C | -2.181951073235 | 3.755755190360 | -1.100122375595 |
| H | -1.902127384960 | 4.306122528676 | -2.003379355635 |
| H | -2.917463440691 | 4.366683214214 | -0.558133472207 |
| H | -1.296619655415 | 3.675167633263 | -0.464512266177 |
| O | -0.557278657939 | 2.276427334271 | -4.922816507565 |
| O | -3.979811114738 | 0.965845044907 | 1.811384608197 |
| C | -1.851890167263 | 0.307091785268 | -3.323608991882 |
| H | -1.126756794416 | -0.414050282944 | -3.720411877761 |
| H | -2.485902458364 | -0.221112821843 | -2.609847908938 |
| H | -2.473544912548 | 0.637156767679 | -4.157154023564 |
| C | 0.301670721346 | 3.168445864000 | -5.669353185706 |
| H | 1.296276047527 | 3.198155131210 | -5.211880759499 |
| H | -0.116208246308 | 4.180901776473 | -5.657579198432 |
| C | 0.454093415889 | 2.695417448604 | -7.130129015131 |
| H | 0.865569101555 | 1.676422270881 | -7.143941683279 |
| H | -0.527395026788 | 2.674840397262 | -7.624023684606 |
| C | -4.475232563304 | 1.524608127125 | 3.050638206217 |
| H | -5.230707454914 | 2.287140877562 | 2.833749527563 |
| H | -3.651437706821 | 1.999805753941 | 3.596037656706 |
| C | -5.099440711575 | 0.434943755697 | 3.945878353901 |
| H | -5.941814798586 | -0.039902145373 | 3.423671058117 |
| H | -4.351691102383 | -0.340322153244 | 4.164963378495 |
| O | 1.336873390090 | 3.618212026049 | -7.774926063105 |
| C | 1.657686565881 | 3.366150401197 | -9.158846047464 |
| H | 2.156908171314 | 2.396342199387 | -9.282491811534 |

| | | | |
|---|---|---|---|
| H | 0.758559381395 | 3.389019883938 | -9.788157396471 |
| H | 2.333878036087 | 4.164478280057 | -9.462227075167 |
| O | -5.535541647421 | 1.084405944118 | 5.144120928989 |
| C | -6.169704048996 | 0.263478476910 | 6.146415435897 |
| H | -7.082960557051 | -0.206533064424 | 5.758763414192 |
| H | -5.491084872746 | -0.519105074134 | 6.510193087381 |
| H | -6.425696594869 | 0.932600873126 | 6.967052900427 |

Cam_TS2C-F1000

($E_F$ = -929.15917502 a.u.; $G_F$ = -928.78043433 a.u.)

0 1

| | | | |
|---|---|---|---|
| C | -1.078913330608 | 1.410306581288 | -2.643527324487 |
| C | -0.407067661965 | 2.276935456062 | -3.496811129371 |
| C | -3.398217375958 | 1.916656027043 | 0.904316031484 |
| C | -2.773736414424 | 1.378087903463 | -0.354144865773 |
| C | -1.340663469635 | 0.768823907501 | -0.144700833167 |
| C | -0.386983590764 | 1.113218747171 | -1.314306689272 |
| H | 0.292626807342 | 3.018189088980 | -3.121689787375 |
| H | -2.996792574722 | 2.815418396387 | 1.370386225203 |
| H | -3.428900631091 | 0.569625703416 | -0.695290352009 |
| H | -0.910241201623 | 1.135868912507 | 0.791094504708 |
| H | -1.436267263082 | -0.313633255302 | -0.028339777300 |
| H | 0.317773713010 | 0.284730960668 | -1.461261211371 |
| H | 0.224952719914 | 1.978778457741 | -1.044325305261 |
| C | -2.714010326102 | 2.427073000014 | -1.468280425519 |
| C | -3.899722866170 | 2.529256968308 | -2.386396947436 |

| | | | |
|---|---|---|---|
| H | -3.640053938581 | 3.087434279718 | -3.292258736035 |
| H | -4.273257063588 | 1.547231379055 | -2.685951056136 |
| H | -4.731605389650 | 3.062458663238 | -1.901540313405 |
| C | -2.096362554855 | 3.756978827719 | -1.116269516431 |
| H | -1.786384820431 | 4.287847549591 | -2.021556851507 |
| H | -2.817961896729 | 4.399687094362 | -0.592292721132 |
| H | -1.222501133230 | 3.654779532651 | -0.467909175646 |
| O | -0.567104950025 | 2.250186044158 | -4.865482963518 |
| O | -3.977714231422 | 1.008888058391 | 1.765636113263 |
| C | -1.831672900794 | 0.260542866506 | -3.283350567684 |
| H | -1.125041317670 | -0.487061144346 | -3.664248197582 |
| H | -2.488320656365 | -0.241132038557 | -2.570996876560 |
| H | -2.433729056794 | 0.601252754323 | -4.126583369878 |
| C | 0.223028235322 | 3.191340643519 | -5.612879807032 |
| H | 1.216220665040 | 3.294520478363 | -5.162540506589 |
| H | -0.262607892891 | 4.173402791769 | -5.610580078947 |
| C | 0.393235897034 | 2.701521819713 | -7.056657685894 |
| H | 0.868204095929 | 1.710044078725 | -7.052501434391 |
| H | -0.587967548151 | 2.607346689004 | -7.543350142670 |
| C | -4.494198528222 | 1.570150977222 | 2.986364851263 |
| H | -5.272576788745 | 2.306091797375 | 2.757613634113 |
| H | -3.690205142163 | 2.076552340375 | 3.533883882312 |
| C | -5.081596886529 | 0.465287568782 | 3.872027662364 |
| H | -5.902906550619 | -0.040937600952 | 3.345050363083 |
| H | -4.308254513453 | -0.283152127806 | 4.097204886761 |
| O | 1.208949879267 | 3.661959648677 | -7.722411591675 |

| | | | |
|---|---|---|---|
| C | 1.530285444386 | 3.368495093274 | -9.087905498855 |
| H | 2.086241207062 | 2.425235285258 | -9.174567261843 |
| H | 0.626233266860 | 3.306002786712 | -9.708452308107 |
| H | 2.154209031718 | 4.188689157121 | -9.442000414860 |
| O | -5.545928432890 | 1.100575070327 | 5.060722212282 |
| C | -6.144856219014 | 0.239964894484 | 6.037151758591 |
| H | -7.031945442109 | -0.267387105900 | 5.634542870984 |
| H | -5.434185468448 | -0.517782031188 | 6.393936218308 |
| H | -6.441154459582 | 0.876607623896 | 6.870412184633 |

Cam_TS2C-F500

($E_F$ = -928.96260737 a.u.; $G_F$ = -928.58457049 a.u.)

0 1

| | | | |
|---|---|---|---|
| C | -1.002447814216 | 1.297021532065 | -2.578396914627 |
| C | -0.316181016552 | 2.147155955474 | -3.420712519519 |
| C | -3.417803716973 | 1.990376059422 | 0.842595288356 |
| C | -2.687882584095 | 1.443309257798 | -0.344317646543 |
| C | -1.252328214365 | 0.939157802940 | -0.006629392480 |
| C | -0.323635908910 | 0.957448909953 | -1.252304266357 |
| H | 0.432852458563 | 2.841986279380 | -3.052947916289 |
| H | -3.101421130615 | 2.928432925151 | 1.296842697268 |
| H | -3.273163438255 | 0.587187671000 | -0.697416251511 |
| H | -0.824938208012 | 1.580175448876 | 0.769796889456 |
| H | -1.320233837495 | -0.059497807683 | 0.430698608788 |
| H | 0.164248934620 | -0.018299086708 | -1.364188103357 |
| H | 0.484904383177 | 1.674310683594 | -1.084267703705 |

| | | | |
|---|---|---|---|
| C | -2.610213501146 | 2.457298222222 | -1.477625129527 |
| C | -3.784770369933 | 2.572222462391 | -2.407038878914 |
| H | -3.478864291931 | 3.022996864450 | -3.357828729456 |
| H | -4.237182900784 | 1.601008027529 | -2.620217470454 |
| H | -4.570954452752 | 3.214271892578 | -1.982066739169 |
| C | -1.924349053896 | 3.764872138175 | -1.179902887695 |
| H | -1.644452713699 | 4.271521891858 | -2.108628489511 |
| H | -2.588227447124 | 4.445386739920 | -0.627253420157 |
| H | -1.017349148558 | 3.635551968698 | -0.583655529213 |
| O | -0.567562824294 | 2.199461854968 | -4.770197248509 |
| O | -3.977222976420 | 1.070494845986 | 1.695940029304 |
| C | -1.832629273565 | 0.200598681936 | -3.216502656536 |
| H | -1.174968101477 | -0.587819896075 | -3.602455845937 |
| H | -2.512657259100 | -0.263574644348 | -2.500965821736 |
| H | -2.417427956675 | 0.580486477796 | -4.054631763915 |
| C | 0.177911035956 | 3.172891801248 | -5.509042293310 |
| H | 1.193775711641 | 3.264098088538 | -5.108953894999 |
| H | -0.309545477082 | 4.152296858161 | -5.444564685951 |
| C | 0.265603160452 | 2.725051761686 | -6.964765052656 |
| H | 0.736926257226 | 1.732562582819 | -7.011926998848 |
| H | -0.741094889702 | 2.643709986597 | -7.399759078603 |
| C | -4.600928769006 | 1.622916340109 | 2.861185641007 |
| H | -5.448986831170 | 2.253433987047 | 2.572128723066 |
| H | -3.884493703184 | 2.239931930822 | 3.417213887049 |
| C | -5.082013501468 | 0.484948452345 | 3.754472220124 |
| H | -5.814553563734 | -0.132903810685 | 3.215106501967 |

| | | | |
|---|---|---|---|
| H | -4.232124157398 | -0.157321866632 | 4.028120158933 |
| O | 1.043944638125 | 3.697985644950 | -7.646072099249 |
| C | 1.290250786471 | 3.400218563579 | -9.017165112749 |
| H | 1.841921288583 | 2.456583232890 | -9.130250974316 |
| H | 0.354335596804 | 3.331528050832 | -9.588929848900 |
| H | 1.892746835578 | 4.216897782949 | -9.414906421195 |
| O | -5.662283204967 | 1.087523046811 | 4.902213284261 |
| C | -6.164014820755 | 0.164613407704 | 5.863828493100 |
| H | -6.957553185429 | -0.466575407852 | 5.440210352402 |
| H | -5.367144865305 | -0.485071893687 | 6.251893622364 |
| H | -6.575465503359 | 0.754098901234 | 6.683223063061 |

Cam_Int1-F500

($E_F$ = -928.99139981 a.u.; $G_F$ = -928.60773950 a.u.)

0 1

| | | | |
|---|---|---|---|
| C | -1.211703000816 | 1.509628830178 | -2.381750771390 |
| C | -0.373705956051 | 2.339872551016 | -3.306323816400 |
| C | -3.438304510306 | 1.966432514238 | 0.732470831695 |
| C | -2.664411879787 | 1.361926124432 | -0.392720605645 |
| C | -1.255459644385 | 0.827836435778 | 0.027670492783 |
| C | -0.324938896554 | 1.033373768066 | -1.190008239192 |
| H | 0.249940922622 | 3.146720633523 | -2.933287597051 |
| H | -3.116302533636 | 2.902795548941 | 1.185874015711 |
| H | -3.254597312408 | 0.510330751084 | -0.746956567230 |
| H | -0.887612579294 | 1.383028318277 | 0.894394601405 |
| H | -1.323201463126 | -0.218270283309 | 0.332084658508 |

| | | | |
|---|---|---|---|
| H | 0.222021929707 | 0.128167275725 | -1.462942306759 |
| H | 0.427628451424 | 1.793404457344 | -0.964342393880 |
| C | -2.387466399329 | 2.290646646354 | -1.621907937671 |
| C | -3.634234232204 | 2.487190339124 | -2.490312881234 |
| H | -3.382115105505 | 3.030024021094 | -3.406512208670 |
| H | -4.090388596913 | 1.536542077346 | -2.773256591496 |
| H | -4.386810708176 | 3.064507030527 | -1.945712978732 |
| C | -1.897215755152 | 3.677994786676 | -1.161263432479 |
| H | -1.645143757658 | 4.300485537726 | -2.022470463980 |
| H | -2.682505625755 | 4.194935179341 | -0.605201124666 |
| H | -1.014457371235 | 3.620001250914 | -0.519209369312 |
| O | -0.637336568034 | 2.349679969731 | -4.652680853653 |
| O | -3.997323630095 | 1.061122814512 | 1.603609516829 |
| C | -1.748346414549 | 0.283336222683 | -3.149786287901 |
| H | -0.909740970280 | -0.298724243581 | -3.539512987941 |
| H | -2.337959674621 | -0.368713399412 | -2.502545522258 |
| H | -2.364695986469 | 0.578861419854 | -3.998639313354 |
| C | 0.308876780080 | 3.092712155932 | -5.431902848978 |
| H | 1.328800834756 | 2.807382704431 | -5.152558767516 |
| H | 0.186404338684 | 4.166895419107 | -5.248759556606 |
| C | 0.098437237148 | 2.794246013144 | -6.911851098718 |
| H | 0.213149947049 | 1.715527858747 | -7.091796639782 |
| H | -0.916110759698 | 3.085035434913 | -7.220852962523 |
| C | -4.580432149281 | 1.627495982560 | 2.782243613194 |
| H | -5.412924959634 | 2.287017670646 | 2.512948407710 |
| H | -3.833968583158 | 2.217357612309 | 3.328088871954 |

| | | | |
|---|---|---|---|
| C | -5.082698545484 | 0.500203054131 | 3.677481634695 |
| H | -5.844443531758 | -0.089723291354 | 3.147386707939 |
| H | -4.249546867242 | -0.171337626098 | 3.931662336951 |
| O | 1.084371249126 | 3.541074688656 | -7.610719377714 |
| C | 1.090632510159 | 3.343436556999 | -9.020888916337 |
| H | 1.288871991375 | 2.293800605908 | -9.278778431774 |
| H | 0.135387711254 | 3.642826606236 | -9.474167467882 |
| H | 1.888323064510 | 3.967550708133 | -9.423794240961 |
| O | -5.622477848496 | 1.113324618205 | 4.839189405344 |
| C | -6.137258076313 | 0.200207444618 | 5.803334685668 |
| H | -6.958391199381 | -0.401537593942 | 5.389575671796 |
| H | -5.355418746807 | -0.478029275798 | 6.172660978311 |
| H | -6.514673800395 | 0.796742501719 | 6.633930013145 |

Cam_TS2C-F200

($E_F$ = -928.84708243 a.u.; $G_F$ = -928.46838941 a.u.)

0 1

| | | | |
|---|---|---|---|
| C | -0.966477407026 | 1.027217474471 | -2.566716782053 |
| C | -0.191604879587 | 1.938512605714 | -3.254274498338 |
| C | -3.413614685966 | 2.030644851548 | 0.729960372262 |
| C | -2.622317524616 | 1.383537561780 | -0.358357099454 |
| C | -1.140765437721 | 1.144948842798 | 0.000679953433 |
| C | -0.426772250507 | 0.530927329036 | -1.217068286038 |
| H | 0.563574613309 | 2.548287019522 | -2.765406244795 |
| H | -3.212564388233 | 3.055153801971 | 1.039073204703 |
| H | -3.081028839783 | 0.401300992034 | -0.533070944035 |

| | | | |
|---|---|---|---|
| H | -0.678740137415 | 2.100317904389 | 0.269303930866 |
| H | -1.058287238229 | 0.498778961524 | 0.879109246985 |
| H | -0.550853102218 | -0.556904616381 | -1.191327607067 |
| H | 0.649396018805 | 0.713876287465 | -1.146680365926 |
| C | -2.688315449552 | 2.170689194607 | -1.662266099723 |
| C | -3.923279596152 | 1.993192874143 | -2.505137818585 |
| H | -3.746074057043 | 2.323242252878 | -3.533956993806 |
| H | -4.265790803088 | 0.956713318516 | -2.532493008855 |
| H | -4.752663876577 | 2.599132948934 | -2.110429628096 |
| C | -2.189476174403 | 3.594142081518 | -1.639203978220 |
| H | -2.017619705898 | 3.950903756286 | -2.659128928335 |
| H | -2.930158701515 | 4.264758306328 | -1.179962241199 |
| H | -1.252366471897 | 3.707570468195 | -1.090898817437 |
| O | -0.460321088646 | 2.249859994048 | -4.563020106445 |
| O | -3.878168620627 | 1.181339587375 | 1.699745159059 |
| C | -1.748678647939 | 0.010724783776 | -3.378262450692 |
| H | -1.068172703415 | -0.761537656657 | -3.757541698993 |
| H | -2.502423218902 | -0.489760016941 | -2.767827660450 |
| H | -2.242749123016 | 0.468439339705 | -4.234918759996 |
| C | 0.527212150329 | 3.052714378541 | -5.210423906241 |
| H | 1.527256496186 | 2.632743272070 | -5.053722645837 |
| H | 0.512759972141 | 4.075149113506 | -4.813495220903 |
| C | 0.218833013886 | 3.064370019566 | -6.698601268412 |
| H | 0.213690807391 | 2.032030512467 | -7.077760609784 |
| H | -0.777700749190 | 3.494987267078 | -6.875896464048 |
| C | -4.578803203476 | 1.798322240001 | 2.780588935280 |

| | | | |
|---|---|---|---|
| H | -5.473401654802 | 2.312049050282 | 2.411194485401 |
| H | -3.937510981518 | 2.533562116198 | 3.282277364448 |
| C | -4.970419417644 | 0.706012777314 | 3.762577460122 |
| H | -5.617898723583 | -0.029244668702 | 3.262567357096 |
| H | -4.068372795644 | 0.180646954475 | 4.109781924563 |
| O | 1.231137023887 | 3.835654116458 | -7.321756726292 |
| C | 1.124038094734 | 3.874734453574 | -8.736838515469 |
| H | 1.202596357730 | 2.869328918010 | -9.174029142493 |
| H | 0.172860699490 | 4.322746676401 | -9.057410255899 |
| H | 1.947423094612 | 4.488062862295 | -9.103559200374 |
| O | -5.643581781640 | 1.335745801774 | 4.838463065267 |
| C | -6.069702889775 | 0.430560828720 | 5.845862697572 |
| H | -6.775573546216 | -0.312072920869 | 5.447847785989 |
| H | -5.219097164357 | -0.100710910921 | 6.295899193902 |
| H | -6.569080830919 | 1.019557493977 | 6.615493511960 |

Pin_TS2C-F2500

($E_F$ = -929.80790720 a.u.; $G_F$ = -929.43161500 a.u.)

0 1

| | | | |
|---|---|---|---|
| C | -2.514460679411 | 2.707020626613 | 1.172316625214 |
| C | -1.211522247774 | 4.283743828126 | 0.924913359355 |
| C | -1.476914274965 | 5.681894499854 | 1.033246624136 |
| C | -1.584818779707 | -0.419715475072 | 2.789695644539 |
| C | -1.334068181126 | 1.067518800025 | 3.033589222722 |
| C | -1.254465459719 | 2.060112022475 | 1.798845825137 |
| H | -0.847779528545 | 4.010968132668 | -0.065280336772 |

| | | | |
|---|---|---|---|
| H | -2.366694077977 | -0.745555016317 | 2.104333818357 |
| H | -2.084927404901 | 1.458830043012 | 3.739392544586 |
| H | -0.377690161313 | 1.144171368576 | 3.565962698843 |
| H | -0.706624697672 | 1.546743754580 | 1.000965736536 |
| C | -0.564646894804 | 3.434047977819 | 2.056769054232 |
| H | 0.531527269416 | 3.423406441648 | 2.035153871980 |
| H | -0.860061524472 | 3.811368985064 | 3.039846668567 |
| C | -3.011433842200 | 2.159624414436 | -0.138471848232 |
| H | -3.533184073308 | 1.200045698390 | 0.006207799480 |
| H | -3.720243609390 | 2.847866710089 | -0.609974292399 |
| H | -2.190174151051 | 1.982262140176 | -0.838531936746 |
| C | -3.605658602120 | 3.124682156041 | 2.123615436184 |
| H | -4.217751254183 | 2.260141586455 | 2.421615018841 |
| H | -3.214518263952 | 3.572781086013 | 3.040006772494 |
| H | -4.272599597821 | 3.852065860969 | 1.651522766362 |
| C | -1.839407358601 | 6.354304474485 | 2.322476753851 |
| H | -2.807412514056 | 6.865485756295 | 2.264191379676 |
| H | -1.908193653913 | 5.621933230408 | 3.124589638786 |
| H | -1.099465487151 | 7.105566558252 | 2.626262757701 |
| O | -1.429004596935 | 6.487517643146 | -0.127489410893 |
| O | -1.320774372481 | -1.286701919713 | 3.852560164432 |
| C | -1.818352703079 | 7.895851493806 | -0.063451035817 |
| H | -1.332108701652 | 8.388250887574 | 0.781375464113 |
| H | -2.901797651688 | 7.972103732161 | 0.071316344010 |
| C | -1.427931552056 | 8.700586323010 | -1.345509331828 |
| H | -0.344232863910 | 8.628645490079 | -1.507668664304 |

| | | | |
|---|---|---|---|
| H | -1.937582096045 | 8.283257394007 | -2.223831526314 |
| C | -1.652649283483 | -2.698043771631 | 3.674672066978 |
| H | -1.108562428684 | -3.091344577507 | 2.810345418412 |
| H | -2.726951265892 | -2.796980384869 | 3.483688146965 |
| C | -1.286831901045 | -3.553735256933 | 4.931383395314 |
| H | -0.209692245406 | -3.468242930310 | 5.127867768461 |
| H | -1.827045743172 | -3.166157817767 | 5.805681440376 |
| O | -1.828122812791 | 10.069545529121 | -1.117563679288 |
| C | -1.590471451569 | 11.086881517257 | -2.144621824865 |
| H | -0.519914646969 | 11.193758284600 | -2.356168658409 |
| H | -2.123738144325 | 10.846122294020 | -3.071912210629 |
| H | -1.978863600597 | 12.012955517210 | -1.724254319710 |
| O | -1.654866087496 | -4.922520791591 | 4.663705409514 |
| C | -1.412291519728 | -5.949658390719 | 5.680628241031 |
| H | -0.343514928754 | -6.034129147819 | 5.910427768290 |
| H | -1.968004542241 | -5.734303110632 | 6.601001325305 |
| H | -1.770842483378 | -6.878461338203 | 5.240378002282 |

Pin_TS2C-F2000

($E_F$ = -929.59372096 a.u.; $G_F$ = -929.21660521 a.u.)

0 1

| | | | |
|---|---|---|---|
| C | -2.487768999994 | 2.704411055188 | 1.179449945683 |
| C | -1.179805575058 | 4.282308517303 | 0.947344168552 |
| C | -1.462166755658 | 5.667714072986 | 1.066151100227 |
| C | -1.601413581577 | -0.379641644880 | 2.776352033890 |
| C | -1.310669703631 | 1.087792590962 | 3.035159360646 |

| | | | |
|---|---|---|---|
| C | -1.226142164545 | 2.067002658539 | 1.805934726192 |
| H | -0.819241756720 | 4.017768376419 | -0.046046913120 |
| H | -2.407493768406 | -0.680814366081 | 2.107800029876 |
| H | -2.041835722022 | 1.494861623857 | 3.753195460633 |
| H | -0.345321737584 | 1.134881026403 | 3.554938048295 |
| H | -0.676949823176 | 1.551644258502 | 1.010062147973 |
| C | -0.535212676358 | 3.432952199692 | 2.071333980367 |
| H | 0.560930348309 | 3.420374007440 | 2.048553640071 |
| H | -0.828858547853 | 3.805585311488 | 3.056728310209 |
| C | -2.977424066772 | 2.161348220170 | -0.135481914338 |
| H | -3.500240332513 | 1.201138353115 | 0.001817429704 |
| H | -3.683822254151 | 2.851117765193 | -0.608572373647 |
| H | -2.152415441580 | 1.986599019635 | -0.831835613041 |
| C | -3.582315674900 | 3.120439174833 | 2.127055690239 |
| H | -4.194246179515 | 2.255544468990 | 2.424661156441 |
| H | -3.194734202247 | 3.570721738005 | 3.043891977032 |
| H | -4.249244531871 | 3.846274184081 | 1.652364215390 |
| C | -1.820815470891 | 6.337814519840 | 2.357472349050 |
| H | -2.796314358823 | 6.835476230715 | 2.308413810724 |
| H | -1.870857743131 | 5.606241183107 | 3.161559759558 |
| H | -1.087803947579 | 7.099655300458 | 2.651645994833 |
| O | -1.429619227287 | 6.462048426756 | -0.094503229769 |
| O | -1.329733750713 | -1.253316067606 | 3.823307861558 |
| C | -1.864451488293 | 7.847657171164 | -0.041438012455 |
| H | -1.421251969494 | 8.361067909278 | 0.814679891074 |
| H | -2.954041180266 | 7.892586833379 | 0.057750070243 |

| | | | |
|---|---|---|---|
| C | -1.449953857274 | 8.639876673927 | -1.310910495752 |
| H | -0.359908548883 | 8.587324810771 | -1.437124228767 |
| H | -1.921571169054 | 8.206119708538 | -2.202927156975 |
| C | -1.688759247018 | -2.647846788005 | 3.636477319215 |
| H | -1.171080747184 | -3.045158314366 | 2.757432673699 |
| H | -2.768919240133 | -2.730688792126 | 3.470176830889 |
| C | -1.304492913217 | -3.498956296059 | 4.877303515628 |
| H | -0.221488570216 | -3.428217407729 | 5.048215670652 |
| H | -1.817401621987 | -3.104698872070 | 5.765500897925 |
| O | -1.882981411749 | 9.995467999484 | -1.104594468108 |
| C | -1.612913186308 | 10.982451472788 | -2.135962841905 |
| H | -0.535046584196 | 11.094143353220 | -2.307727716890 |
| H | -2.103607928060 | 10.716370776477 | -3.080484847510 |
| H | -2.023263613256 | 11.918995594501 | -1.761158988256 |
| O | -1.697321851575 | -4.855149608110 | 4.615278149132 |
| C | -1.433033058648 | -5.857685021424 | 5.634093331567 |
| H | -0.358306809926 | -5.944731834022 | 5.836853525095 |
| H | -1.959338621563 | -5.624029243803 | 6.568009627107 |
| H | -1.806665409542 | -6.796330001542 | 5.227625998032 |

Pin_TS2C-F1800

($E_F$ = -929.50869156 a.u.; $G_F$ = -929.13129005 a.u.)

0 1

| | | | |
|---|---|---|---|
| C | -2.474478444853 | 2.704634107058 | 1.183805532639 |
| C | -1.164082462558 | 4.282392614656 | 0.957429986367 |
| C | -1.453892019924 | 5.662624248848 | 1.080262559564 |

| | | | |
|---|---|---|---|
| C | -1.609088869485 | -0.362794331448 | 2.771483124056 |
| C | -1.298845383225 | 1.095825916292 | 3.036297083563 |
| C | -1.212134593890 | 2.070370619818 | 1.809463720353 |
| H | -0.805275710495 | 4.021203902232 | -0.037401198669 |
| H | -2.426918387565 | -0.651735696777 | 2.111835189507 |
| H | -2.020216387592 | 1.510233229206 | 3.760210374735 |
| H | -0.329442514302 | 1.128743890267 | 3.549636601952 |
| H | -0.663069028650 | 1.554140424105 | 1.014002275207 |
| C | -0.519932593790 | 3.432826885210 | 2.077833110712 |
| H | 0.576157263339 | 3.418987371667 | 2.053768423083 |
| H | -0.812067279056 | 3.803562297794 | 3.064386903804 |
| C | -2.962361736768 | 2.163793013674 | -0.132531185054 |
| H | -3.486290272437 | 1.203751417946 | 0.002019809233 |
| H | -3.667532199235 | 2.854777526870 | -0.605770164405 |
| H | -2.136405624676 | 1.989496948056 | -0.827889228637 |
| C | -3.569524363177 | 3.120562553236 | 2.130677951563 |
| H | -4.181646941184 | 2.255849012475 | 2.428504222442 |
| H | -3.182514490351 | 3.571522278615 | 3.047425702581 |
| H | -4.236368233138 | 3.846141264366 | 1.655390346827 |
| C | -1.810921908303 | 6.332223648963 | 2.372109962270 |
| H | -2.791034634760 | 6.821253024144 | 2.327977423530 |
| H | -1.849588290634 | 5.601972617218 | 3.177975929373 |
| H | -1.082600165817 | 7.101018775281 | 2.659860671885 |
| O | -1.426413151254 | 6.452401251522 | -0.080329118510 |
| O | -1.333561211489 | -1.240648998235 | 3.810636860056 |
| C | -1.890649336249 | 7.825070764558 | -0.036323057428 |

| | | | |
|---|---|---|---|
| H | -1.478620586951 | 8.349681829095 | 0.828533740578 |
| H | -2.983220837733 | 7.848200449655 | 0.036733480603 |
| C | -1.458184779101 | 8.615792794945 | -1.295912937112 |
| H | -0.364472438941 | 8.579207418267 | -1.394637383263 |
| H | -1.900240722330 | 8.171565064415 | -2.198055482684 |
| C | -1.707782114086 | -2.627456249526 | 3.621157065482 |
| H | -1.205913871020 | -3.026767979608 | 2.733727206198 |
| H | -2.790971872134 | -2.701017254195 | 3.469935368699 |
| C | -1.312242913670 | -3.478477534227 | 4.853392627878 |
| H | -0.226128080696 | -3.416583836913 | 5.008322396665 |
| H | -1.808323792880 | -3.080230925061 | 5.749540726031 |
| O | -1.916337188779 | 9.962801144035 | -1.105293633521 |
| C | -1.624922580629 | 10.939480864634 | -2.134384559292 |
| H | -0.543207162964 | 11.060753952105 | -2.274242519696 |
| H | -2.082348888412 | 10.659392193991 | -3.091782680983 |
| H | -2.055296832765 | 11.876961356121 | -1.784773324854 |
| O | -1.719859015074 | -4.828664986575 | 4.596242194819 |
| C | -1.443471031625 | -5.822171195845 | 5.614392919711 |
| H | -0.365682511731 | -5.913140453304 | 5.799891519299 |
| H | -1.951697878631 | -5.579494113012 | 6.556312413616 |
| H | -1.827712602411 | -6.763520787224 | 5.223972946089 |

Pin_Int1-F1800

($E_F$ = -929.52497837 a.u.; $G_F$ = -929.14420874 a.u.)

0 1

| | | | |
|---|---|---|---|
| C | -2.355735893138 | 2.900521037204 | 1.180523743988 |

| | | | |
|---|---|---|---|
| C | -1.349781904952 | 4.134833396213 | 0.959189556018 |
| C | -1.770268129663 | 5.581799180360 | 1.060867055919 |
| C | -1.584058147254 | -0.343643313423 | 2.751598755424 |
| C | -1.265123154165 | 1.113265649952 | 3.020123474644 |
| C | -1.147260572889 | 2.086474931712 | 1.803483883737 |
| H | -0.895483425309 | 4.004792681616 | -0.027567910341 |
| H | -2.397506104077 | -0.628594050518 | 2.084884452880 |
| H | -1.985781829941 | 1.529293679707 | 3.742837789890 |
| H | -0.299471968652 | 1.134747602583 | 3.541606917541 |
| H | -0.641087070691 | 1.556069509940 | 0.989769385074 |
| C | -0.430955244215 | 3.450773117127 | 2.036677091392 |
| H | 0.647779416783 | 3.489637202831 | 1.867832664296 |
| H | -0.636620137618 | 3.833489471936 | 3.040133734550 |
| C | -2.950168322201 | 2.334506702014 | -0.104555027113 |
| H | -3.510085519430 | 1.413804373333 | 0.093512177875 |
| H | -3.636406832541 | 3.054756508542 | -0.562677649265 |
| H | -2.167407409512 | 2.104090391246 | -0.832945792315 |
| C | -3.466068246628 | 3.192962331497 | 2.188828692797 |
| H | -4.054414302537 | 2.289348675712 | 2.376284035312 |
| H | -3.082189326645 | 3.536719390999 | 3.151432434902 |
| H | -4.136929406215 | 3.964771602092 | 1.801292341865 |
| C | -1.967965148719 | 6.252506559782 | 2.385032686394 |
| H | -2.786147365397 | 6.979413511706 | 2.377950641482 |
| H | -2.199754236358 | 5.515225693450 | 3.151388259509 |
| H | -1.062877618321 | 6.789684926751 | 2.713388343525 |
| O | -1.431295065994 | 6.395884667846 | -0.034525898123 |

| | | | |
|---|---|---|---|
| O | -1.321825701367 | -1.221220285522 | 3.794215741569 |
| C | -1.890928228679 | 7.771235428846 | -0.029505130610 |
| H | -1.511128278360 | 8.300587572532 | 0.848527498066 |
| H | -2.985707502481 | 7.793643043900 | 0.003725666349 |
| C | -1.414540690882 | 8.552922269863 | -1.277919144783 |
| H | -0.318303430326 | 8.514801956920 | -1.343260353684 |
| H | -1.829649669730 | 8.105775499091 | -2.191395687161 |
| C | -1.698024956962 | -2.607284910378 | 3.603618617506 |
| H | -1.190776775833 | -3.008793207948 | 2.720261933736 |
| H | -2.780289170404 | -2.678587894500 | 3.444927153269 |
| C | -1.312614717798 | -3.457359239296 | 4.839749282047 |
| H | -0.227605496963 | -3.396269943518 | 5.002537181476 |
| H | -1.814834159929 | -3.057497674462 | 5.731747640109 |
| O | -1.876169086245 | 9.901813198419 | -1.106838440980 |
| C | -1.563538035954 | 10.872618406744 | -2.134747935997 |
| H | -0.479198015233 | 10.995118121907 | -2.251679133779 |
| H | -1.999335709966 | 10.586460711584 | -3.100454979715 |
| H | -2.002853418341 | 11.811471805482 | -1.800223093677 |
| O | -1.719628728093 | -4.807422750242 | 4.581314355339 |
| C | -1.451704279443 | -5.799796909849 | 5.602873535631 |
| H | -0.375430658328 | -5.891184106185 | 5.796737783947 |
| H | -1.966982520264 | -5.555518618682 | 6.540529463826 |
| H | -1.833556660827 | -6.741402137505 | 5.210749445727 |

Pin_TS2C-F1500

($E_F$ = -929.38184067 a.u.; $G_F$ = -929.00428929 a.u.)

0 1

| | | | |
|---|---|---|---|
| C | -2.448067151124 | 2.705245307807 | 1.190513000334 |
| C | -1.135475021999 | 4.284085582855 | 0.975219619263 |
| C | -1.439216654224 | 5.655937004009 | 1.103952938525 |
| C | -1.620302798306 | -0.335349677175 | 2.765035083713 |
| C | -1.276840220327 | 1.108600512136 | 3.039598729631 |
| C | -1.185014161621 | 2.076798084213 | 1.816722313552 |
| H | -0.778272251612 | 4.028549468682 | -0.021515873824 |
| H | -2.456323647430 | -0.602744096819 | 2.119197242064 |
| H | -1.982132313015 | 1.535123586257 | 3.772546194318 |
| H | -0.301640675191 | 1.117326720178 | 3.542933038009 |
| H | -0.634615905338 | 1.559646065958 | 1.022698077013 |
| C | -0.492166906009 | 3.434559955169 | 2.090701051233 |
| H | 0.603853240159 | 3.419716182956 | 2.065645754982 |
| H | -0.783254089795 | 3.801958324926 | 3.078794434992 |
| C | -2.930576517193 | 2.167476053809 | -0.128800054581 |
| H | -3.455039758814 | 1.206933967492 | 0.000515129091 |
| H | -3.634057889793 | 2.859486865438 | -0.603183519219 |
| H | -2.101880120681 | 1.995131966783 | -0.821409351877 |
| C | -3.545675107436 | 3.119828966649 | 2.134690806829 |
| H | -4.157429160457 | 2.254699999118 | 2.432179222553 |
| H | -3.161288573611 | 3.572498907736 | 3.051712153512 |
| H | -4.212594440100 | 3.844133398887 | 1.657441378212 |
| C | -1.794488238710 | 6.324644780461 | 2.396445606638 |
| H | -2.782845903773 | 6.797592136495 | 2.360593675973 |
| H | -1.812874494105 | 5.597142729233 | 3.205462344198 |

| | | | |
|---|---|---|---|
| H | -1.075430862161 | 7.106168529784 | 2.673106278841 |
| O | -1.423371310113 | 6.438126099542 | -0.057250318800 |
| O | -1.341156820020 | -1.221372432214 | 3.791370888776 |
| C | -1.930474118496 | 7.790587431673 | -0.026456299856 |
| H | -1.560115475135 | 8.331702781261 | 0.847093486796 |
| H | -3.025279613263 | 7.781286077052 | 0.014342815493 |
| C | -1.478637631843 | 8.577769528567 | -1.274486024853 |
| H | -0.381629599437 | 8.562650806313 | -1.339461294233 |
| H | -1.882972359685 | 8.118386715202 | -2.186974123550 |
| C | -1.740702910643 | -2.595595355889 | 3.597123410016 |
| H | -1.263653065972 | -2.997540966368 | 2.697053437962 |
| H | -2.828031541685 | -2.653190167235 | 3.468901142115 |
| C | -1.329147217422 | -3.448159676797 | 4.815623130973 |
| H | -0.238915730491 | -3.400077143185 | 4.946419051241 |
| H | -1.799435305846 | -3.044373711934 | 5.723429677509 |
| O | -1.968883815497 | 9.912726481955 | -1.107114603842 |
| C | -1.649749362653 | 10.872355709404 | -2.135151478218 |
| H | -0.564533218932 | 11.004659894460 | -2.235374052489 |
| H | -2.064694399823 | 10.571924705956 | -3.106087662234 |
| H | -2.102923908025 | 11.812233988755 | -1.821994618453 |
| O | -1.759205006911 | -4.789024382653 | 4.565890629986 |
| C | -1.465266678469 | -5.769473787927 | 5.582888075472 |
| H | -0.383567226997 | -5.866264610284 | 5.743393393637 |
| H | -1.947050355815 | -5.513521576307 | 6.535611270956 |
| H | -1.864290378249 | -6.714495403005 | 5.216236688465 |

Pin_TS2C-F1000

($E_F$ = -929.17232342 a.u.; $G_F$ = -928.79435668 a.u.)

0 1

| | | | |
|---|---|---|---|
| C | -2.384966758814 | 2.710756456170 | 1.206768439054 |
| C | -1.070473741196 | 4.293181005999 | 1.017083563282 |
| C | -1.404744928197 | 5.649113097532 | 1.154699360476 |
| C | -1.632795176917 | -0.283334238355 | 2.755223833852 |
| C | -1.228898208083 | 1.132099724696 | 3.051787072556 |
| C | -1.122198814874 | 2.093113733672 | 1.837939878511 |
| H | -0.713272599092 | 4.048241475805 | 0.017967350470 |
| H | -2.494414599838 | -0.507499176230 | 2.126796550178 |
| H | -1.907705056610 | 1.578071268987 | 3.798440273392 |
| H | -0.247349503823 | 1.094905078276 | 3.541334517842 |
| H | -0.566022388417 | 1.576004740272 | 1.047816959480 |
| C | -0.430381674873 | 3.443671560707 | 2.125564129362 |
| H | 0.665550107909 | 3.428306644462 | 2.101812922565 |
| H | -0.723066601610 | 3.804098900774 | 3.115706391817 |
| C | -2.853215968053 | 2.179078176453 | -0.119655009121 |
| H | -3.378237214186 | 1.217222922609 | -0.002051225760 |
| H | -3.552596940119 | 2.872829882908 | -0.597789860941 |
| H | -2.017334782413 | 2.011303896639 | -0.804770524625 |
| C | -3.490484432931 | 3.121585687729 | 2.142837534161 |
| H | -4.102469060730 | 2.255366416157 | 2.436754939436 |
| H | -3.113958165067 | 3.576523566302 | 3.062018905159 |
| H | -4.155970689262 | 3.843994345876 | 1.660502713605 |
| C | -1.761385306874 | 6.316386292694 | 2.446943862238 |

| | | | |
|---|---|---|---|
| H | -2.767209210442 | 6.752147088229 | 2.425646328634 |
| H | -1.737075408814 | 5.596876773843 | 3.262893968239 |
| H | -1.066351202452 | 7.126558242469 | 2.701504578029 |
| O | -1.418593688390 | 6.415441891841 | -0.009743827008 |
| O | -1.359568055199 | -1.188351487781 | 3.758383820916 |
| C | -1.995610927001 | 7.731486034593 | -0.000392351007 |
| H | -1.678828829441 | 8.302538467014 | 0.875421108310 |
| H | -3.089818004660 | 7.667911985187 | 0.008216999751 |
| C | -1.534898835915 | 8.504805545037 | -1.242873120993 |
| H | -0.436068062008 | 8.526188672260 | -1.270839749272 |
| H | -1.890491590169 | 8.012691094868 | -2.159005656322 |
| C | -1.805933636449 | -2.538525393558 | 3.550405324385 |
| H | -1.369169359550 | -2.941665055015 | 2.630315616296 |
| H | -2.898331526376 | -2.564922365736 | 3.457211216823 |
| C | -1.374257935425 | -3.399628687671 | 4.744125763680 |
| H | -0.279013845745 | -3.376793868775 | 4.837367975290 |
| H | -1.802354865481 | -2.988511531843 | 5.669764586955 |
| O | -2.074146335261 | 9.819490637979 | -1.120616602054 |
| C | -1.717425652238 | 10.743720435586 | -2.155298600954 |
| H | -0.630415541902 | 10.895114306041 | -2.200189680068 |
| H | -2.068610785884 | 10.401883479816 | -3.138293392270 |
| H | -2.202849754696 | 11.687542711952 | -1.908098171654 |
| O | -1.842586040135 | -4.723465555706 | 4.504642762762 |
| C | -1.521736921686 | -5.685138226938 | 5.517921996433 |
| H | -0.435336526533 | -5.793152802990 | 5.638168845490 |
| H | -1.960119475299 | -5.408455931929 | 6.486316246360 |

| | | | |
|---|---|---|---|
| H | -1.944142152856 | -6.634295589533 | 5.189133333108 |

Pin_TS2C-F500

($E_F$ = -928.96542963 a.u.; $G_F$ = -928.58766836 a.u.)

0 1

| | | | |
|---|---|---|---|
| C | -2.263377927526 | 2.730823726989 | 1.241504231045 |
| C | -0.949834193742 | 4.319187007439 | 1.095050674322 |
| C | -1.330212860860 | 5.654465939461 | 1.236999428342 |
| C | -1.625373510826 | -0.210164506500 | 2.751072788006 |
| C | -1.143846922687 | 1.164264402449 | 3.086281925732 |
| C | -1.005802236443 | 2.125643029294 | 1.888643213572 |
| H | -0.584958652598 | 4.086486920966 | 0.096124169816 |
| H | -2.503733522044 | -0.369845295944 | 2.126211480300 |
| H | -1.794540457980 | 1.629939207346 | 3.846167247780 |
| H | -0.162321330739 | 1.063781759813 | 3.566774344347 |
| H | -0.434961549013 | 1.612254661238 | 1.106522923468 |
| C | -0.320022704041 | 3.469762128663 | 2.200911604016 |
| H | 0.775888576227 | 3.455939630015 | 2.187891966486 |
| H | -0.624199628423 | 3.820359451551 | 3.191048207461 |
| C | -2.703990777092 | 2.208195808045 | -0.097348096315 |
| H | -3.231843088090 | 1.245624985180 | 0.000891920589 |
| H | -3.393285733021 | 2.905645921577 | -0.584958509068 |
| H | -1.854256349640 | 2.045254134205 | -0.766456634155 |
| C | -3.385686519771 | 3.135770560886 | 2.159534417197 |
| H | -3.999992286711 | 2.267955613630 | 2.443608475179 |
| H | -3.025711665977 | 3.592760274801 | 3.084320969637 |

| | | | |
|---|---|---|---|
| H | -4.045593892496 | 3.856297839329 | 1.666543150649 |
| C | -1.695086457012 | 6.323895807084 | 2.524949282756 |
| H | -2.724512150915 | 6.701542259324 | 2.521324802380 |
| H | -1.608806648196 | 5.623484264689 | 3.353330996031 |
| H | -1.041219052921 | 7.177170608171 | 2.745009455150 |
| O | -1.386701403755 | 6.396718343182 | 0.065096876697 |
| O | -1.389075012603 | -1.147646678736 | 3.725319741871 |
| C | -2.063413132763 | 7.656269696940 | 0.043350793505 |
| H | -1.794980133653 | 8.277324761780 | 0.901723026145 |
| H | -3.150338996211 | 7.511461769819 | 0.049060482336 |
| C | -1.639497815669 | 8.400091035949 | -1.219880270310 |
| H | -0.542849055779 | 8.480494457623 | -1.238571670103 |
| H | -1.955022157038 | 7.849495867548 | -2.117638630352 |
| C | -1.895506636845 | -2.463873694456 | 3.483199481695 |
| H | -1.491095173179 | -2.860785350965 | 2.545512263690 |
| H | -2.989873106834 | -2.444890381346 | 3.410357041309 |
| C | -1.470016536238 | -3.351585839366 | 4.648495560221 |
| H | -0.372573958889 | -3.363731459002 | 4.719128440770 |
| H | -1.864511523874 | -2.940129689615 | 5.589200090211 |
| O | -2.248491615293 | 9.682399314377 | -1.161109926098 |
| C | -1.888725530502 | 10.556108945028 | -2.226528839401 |
| H | -0.806742032223 | 10.747989797533 | -2.240698635340 |
| H | -2.185914178339 | 10.146512179582 | -3.201997854875 |
| H | -2.416392392267 | 11.494973171838 | -2.057965631475 |
| O | -1.983103779478 | -4.651589694254 | 4.401660310999 |
| C | -1.653056592961 | -5.607910648399 | 5.404983863190 |

| | | | |
|---|---|---|---|
| H | -0.565453562257 | -5.736052197819 | 5.495720053820 |
| H | -2.056209445144 | -5.317632599694 | 6.385225246380 |
| H | -2.100511359755 | -6.553994917882 | 5.100225647236 |

Pin_TS2C-F200

($E_F$ = -928.84310592 a.u.; $G_F$ = -928.46576119 a.u.)

0 1

| | | | |
|---|---|---|---|
| C | -2.060686700554 | 2.782550415138 | 1.291627006781 |
| C | -0.743694743665 | 4.369334704927 | 1.212950104245 |
| C | -1.168399860636 | 5.686290157251 | 1.352517783030 |
| C | -1.577834976727 | -0.120863167916 | 2.760390102397 |
| C | -1.019899615858 | 1.205623044810 | 3.148949779699 |
| C | -0.824272155850 | 2.176857794784 | 1.974642263717 |
| H | -0.354466739840 | 4.149188881876 | 0.220659939545 |
| H | -2.454015349580 | -0.203842793848 | 2.118018299971 |
| H | -1.653814966567 | 1.687461127536 | 3.913149974392 |
| H | -0.051578568430 | 1.035902559625 | 3.636377205735 |
| H | -0.228657711534 | 1.667878633680 | 1.208315368777 |
| C | -0.144465440431 | 3.512886392140 | 2.325667240529 |
| H | 0.951068655408 | 3.496179181534 | 2.341122868317 |
| H | -0.475399606531 | 3.852933611279 | 3.310756217970 |
| C | -2.453478806417 | 2.278159961919 | -0.068490974310 |
| H | -2.991538763843 | 1.318544532071 | -0.003331214258 |
| H | -3.118741959818 | 2.986696799966 | -0.573437381518 |
| H | -1.580106463007 | 2.116968241429 | -0.706881327803 |
| C | -3.212358995261 | 3.184506656234 | 2.173615100389 |

| | | | |
|---|---|---|---|
| H | -3.838718544795 | 2.317572966729 | 2.432280342732 |
| H | -2.881945091831 | 3.636446039905 | 3.111830377922 |
| H | -3.852909650695 | 3.909962168610 | 1.662392995185 |
| C | -1.558973440391 | 6.358516501760 | 2.630710973184 |
| H | -2.614349558825 | 6.656601715291 | 2.642942458023 |
| H | -1.399462061139 | 5.692279539339 | 3.476547978232 |
| H | -0.967593252181 | 7.265900163951 | 2.805936184079 |
| O | -1.238883615549 | 6.416120528931 | 0.177463672340 |
| O | -1.414903439548 | -1.099410794984 | 3.703711498298 |
| C | -2.102483623551 | 7.548788143422 | 0.095562328646 |
| H | -1.941049086940 | 8.246905362752 | 0.922036184662 |
| H | -3.154428660176 | 7.238173687109 | 0.105123341287 |
| C | -1.772156016005 | 8.266909831365 | -1.203847119283 |
| H | -0.696786960804 | 8.497553909535 | -1.220279220685 |
| H | -1.992712912065 | 7.621973216604 | -2.066766004984 |
| C | -1.995901414133 | -2.369102431380 | 3.405799031553 |
| H | -1.606892581935 | -2.754971467442 | 2.456929271499 |
| H | -3.086762966702 | -2.283500101161 | 3.326890245812 |
| C | -1.628023558082 | -3.310885947808 | 4.541501741177 |
| H | -0.533134282441 | -3.383881774904 | 4.617283650629 |
| H | -2.005381108304 | -2.907461523213 | 5.492878708387 |
| O | -2.550862986407 | 9.451044160795 | -1.234109222364 |
| C | -2.274013940390 | 10.280989480232 | -2.351988957921 |
| H | -1.225080373048 | 10.609803270533 | -2.358632097680 |
| H | -2.485186563450 | 9.765883885120 | -3.299867350939 |
| H | -2.920904355391 | 11.154917096751 | -2.270735739125 |

| | | | |
|---|---|---|---|
| O | -2.207245465560 | -4.570674861538 | 4.252869450819 |
| C | -1.920634657998 | -5.557920147539 | 5.232602213506 |
| H | -0.839697349651 | -5.737590712150 | 5.318695478440 |
| H | -2.307619612248 | -5.269513466548 | 6.220181216981 |
| H | -2.410296774716 | -6.478172845126 | 4.913259908847 |

Camphanediol

(E = -542.51041820 a.u.; H = -542.22856734 a.u.)

0 1

| | | | |
|---|---|---|---|
| C | -1.811229307931 | 0.798024401982 | -2.550895596127 |
| C | -1.160916037528 | 1.985245064328 | -1.766751519597 |
| C | -1.814684955324 | 1.879177066857 | -0.343541445298 |
| C | -2.738232988661 | 0.644397011137 | -0.489010282065 |
| C | -1.803629233544 | -0.587397818986 | -0.533811415981 |
| C | -1.162198424749 | -0.477505831177 | -1.951346863648 |
| H | -0.078644640844 | 1.815933068148 | -1.687717684487 |
| H | -1.035495827990 | 1.680216569619 | 0.404810567206 |
| H | -3.506672591696 | 0.595164947786 | 0.287937423396 |
| H | -1.058777728402 | -0.559254775117 | 0.265644023331 |
| H | -2.362213533142 | -1.516663807335 | -0.414063476936 |
| H | -1.386420261759 | -1.351638462470 | -2.565966462683 |
| H | -0.072655939322 | -0.390158850914 | -1.914631098173 |
| C | -3.261485231704 | 0.738744342293 | -1.952780319877 |
| C | -4.071473644707 | -0.494260880968 | -2.394830152249 |
| H | -4.332887715525 | -0.413226201728 | -3.453918161168 |
| H | -3.558817476138 | -1.445900878473 | -2.256273495953 |

| | | | |
|---|---|---|---|
| H | -5.009597038284 | -0.540787958130 | -1.833434843596 |
| C | -4.136528387222 | 1.969545049548 | -2.251281921326 |
| H | -4.397649671982 | 1.987194236833 | -3.314333826013 |
| H | -5.072500725245 | 1.897476060282 | -1.687245128722 |
| H | -3.659693574121 | 2.910371085894 | -2.000945427243 |
| O | -1.390028860237 | 3.268020445841 | -2.328445961177 |
| O | -2.484121412418 | 3.085540782856 | -0.008392003731 |
| C | -1.677154823809 | 0.918745646858 | -4.058347747953 |
| H | -0.623088602362 | 0.941360033682 | -4.358731308684 |
| H | -2.141305881776 | 0.067615221490 | -4.563496845229 |
| H | -2.155955017960 | 1.829801650049 | -4.424684726920 |
| H | -0.844084926583 | 3.336537047495 | -3.116440477605 |
| H | -2.961791023743 | 2.922168355329 | 0.810239115266 |

Camphanediol_H2

(E = -543.72581137 a.u.; H = -543.42024775 a.u.)

0 1

| | | | |
|---|---|---|---|
| C | -1.602140523483 | 1.804978803351 | -2.553973309713 |
| C | -1.061324174522 | 2.759936160430 | -3.622540492102 |
| C | -3.360807148495 | 2.019733443246 | 0.859625554148 |
| C | -2.686731892766 | 1.509731809716 | -0.403698154642 |
| C | -1.204838419694 | 1.120279849212 | -0.197874005686 |
| C | -0.493369251137 | 1.401659190330 | -1.544561383125 |
| H | -0.620399262549 | 3.645135494670 | -3.150297272091 |
| H | -2.783738812881 | 2.848338412163 | 1.293355922799 |
| H | -3.247273713440 | 0.612924499085 | -0.686207464024 |

| | | | |
|---|---|---|---|
| H | -0.765626667304 | 1.718753187147 | 0.606127507842 |
| H | -1.117804966740 | 0.079253758917 | 0.114885187142 |
| H | 0.074602579871 | 0.546120410326 | -1.911804764989 |
| H | 0.224792552694 | 2.219141146112 | -1.435429703384 |
| C | -2.698873322992 | 2.469956516033 | -1.629807287472 |
| C | -4.088922537949 | 2.549139389024 | -2.279148882347 |
| H | -4.056926495033 | 3.111639861559 | -3.217227404184 |
| H | -4.498254676542 | 1.560277787068 | -2.492113331621 |
| H | -4.793606649932 | 3.061705486502 | -1.618050215208 |
| C | -2.289966496357 | 3.898895414036 | -1.206002774937 |
| H | -2.287660188540 | 4.580582228750 | -2.059736216451 |
| H | -3.005388207578 | 4.303445057666 | -0.485947835893 |
| H | -1.300206061908 | 3.931652928431 | -0.744624625470 |
| O | -0.064297414818 | 2.068641046978 | -4.381363022062 |
| O | -3.421751737403 | 0.919805675128 | 1.772045028237 |
| C | -2.135327727255 | 0.543273314578 | -3.265840666074 |
| H | -1.323135124780 | 0.076086209802 | -3.822516239575 |
| H | -2.528275361138 | -0.189079766358 | -2.557885914414 |
| H | -2.926666862209 | 0.786022785631 | -3.979541749103 |
| H | 0.298310553557 | 2.683092222970 | -5.024767552230 |
| H | -3.781950598720 | 1.239841378644 | 2.603692169378 |
| H | -1.883372079859 | 3.093097228900 | -4.274818033830 |
| H | -4.370845742880 | 2.391354066624 | 0.631356289654 |

Pinanediol

(E = -542.48436261 a.u.; H = -542.20322834 a.u.)

0 1

| | | | |
|---|---|---|---|
| C | -1.717126544362 | 1.750165685129 | -0.240824960907 |
| C | -1.772261221729 | 3.232563753912 | 0.292101799957 |
| C | -2.192719950953 | 3.400172964070 | 1.773026133923 |
| C | -1.621450012724 | 2.220500210401 | 2.629071125428 |
| C | -0.429672440684 | 1.464512967149 | 1.972685620423 |
| C | -0.314519435053 | 1.703573007768 | 0.459038637446 |
| H | -2.314483483800 | 3.955612980994 | -0.328624129299 |
| H | -2.447768683999 | 1.509988297940 | 2.732157232666 |
| H | 0.489585285126 | 1.814502008878 | 2.452218852219 |
| H | -0.523962594857 | 0.394693635336 | 2.191619202259 |
| H | 0.447688116550 | 1.061302611798 | 0.008254012373 |
| C | -0.218999433844 | 3.235045092733 | 0.243631598289 |
| H | 0.184184863796 | 3.517578906692 | -0.727347663355 |
| H | 0.276573563614 | 3.813814592189 | 1.021667678091 |
| C | -1.587797195798 | 1.737512369845 | -1.774935273968 |
| H | -0.856017853992 | 2.450915927405 | -2.154764933431 |
| H | -1.290476498873 | 0.742395561442 | -2.120766540183 |
| H | -2.551583171416 | 1.975881844600 | -2.236205768251 |
| C | -2.782106821742 | 0.709964498557 | 0.118911338174 |
| H | -2.495645144664 | -0.264331742633 | -0.290406445754 |
| H | -2.936412527346 | 0.577610929062 | 1.186955387750 |
| H | -3.746231704217 | 0.978417256235 | -0.324646007493 |
| C | -3.712409053701 | 3.484982292542 | 1.944324329787 |
| H | -4.101012681794 | 4.363939664838 | 1.419648603772 |
| H | -4.221492218041 | 2.605057305810 | 1.545260664503 |

| | | | |
|---|---|---|---|
| H | -3.946114406735 | 3.586205345371 | 3.006137634181 |
| O | -1.615381412638 | 4.611651509913 | 2.278393292790 |
| O | -1.299631582479 | 2.711480431169 | 3.926325544264 |
| H | -1.947078233416 | 5.329488523389 | 1.728722649133 |
| H | -1.098688789056 | 1.946309218588 | 4.473707558563 |

Pinanediol_H2

(E = -543.70031681 a.u.; H = -543.39570535 a.u.)

0 1

| | | | |
|---|---|---|---|
| C | -1.414831787491 | 2.664707691946 | 0.753568010114 |
| C | -1.247760037126 | 4.155724638417 | 1.209460984917 |
| C | -2.263239763269 | 5.244218025003 | 0.862841467939 |
| C | -1.970712669770 | 0.895439008390 | 3.783775799213 |
| C | -0.819742775157 | 0.957192698911 | 2.788739233898 |
| C | -0.608506054159 | 2.282641980858 | 2.047697547786 |
| H | -0.298739078193 | 4.514086745502 | 0.799534887305 |
| H | -2.933068974751 | 1.078697929317 | 3.290784891415 |
| H | 0.086275737511 | 0.706544637724 | 3.350209219207 |
| H | -0.954304126719 | 0.149246704502 | 2.060306795751 |
| H | 0.449953042492 | 2.333568527824 | 1.771472699956 |
| C | -1.001250608698 | 3.658309809526 | 2.655518608855 |
| H | -0.258788334723 | 4.186487159324 | 3.255875642223 |
| H | -1.930401240872 | 3.599724994135 | 3.226521964189 |
| C | -0.748716874339 | 2.299677258101 | -0.571305776241 |
| H | 0.287838559973 | 2.646775741925 | -0.600643871967 |
| H | -0.746837383146 | 1.214832212293 | -0.723187977598 |

| | | | |
|---|---|---|---|
| H | -1.278709527992 | 2.751247809309 | -1.416968833173 |
| C | -2.859888598037 | 2.156798383408 | 0.754316459577 |
| H | -2.880217306032 | 1.069801967111 | 0.632342680617 |
| H | -3.389602262481 | 2.394257443714 | 1.676821817314 |
| H | -3.423548546310 | 2.593519069691 | -0.075708436907 |
| C | -3.498085032482 | 5.329359445567 | 1.758970084897 |
| H | -4.148620261583 | 6.144143624781 | 1.423633111893 |
| H | -4.088113153763 | 4.412319333312 | 1.742045595980 |
| H | -3.198358829059 | 5.539813002104 | 2.787886303550 |
| O | -1.532896802892 | 6.481408545774 | 0.934892125859 |
| O | -1.944976171196 | -0.411475314086 | 4.362468945930 |
| H | -2.142771461877 | 7.199095240307 | 0.737172627429 |
| H | -2.650356745914 | -0.464051888216 | 5.013327556263 |
| H | -2.586781455512 | 5.073865465037 | -0.176911874170 |
| H | -1.834878544617 | 1.666714214315 | 4.555241887771 |

Transcyclohexanediol

(E = -386.44147288 a.u.; H = -386.25335164 a.u.)

0 1

| | | | |
|---|---|---|---|
| C | -0.626160312053 | -0.375628506316 | 0.400284171477 |
| C | 0.533750520978 | -0.361173830914 | -0.599175758244 |
| C | 0.550056469737 | 0.934091814955 | -1.404845355268 |
| C | 0.602313379712 | 2.159676402893 | -0.482096552969 |
| C | -0.552221850567 | 2.145763828249 | 0.532417340763 |
| C | -0.577106938235 | 0.833860151544 | 1.332593072886 |
| H | 1.399007577587 | 0.916039227259 | -2.093369585101 |

| | | | |
|---|---|---|---|
| H | -0.357076252617 | 0.959395697262 | -2.019642522447 |
| H | 0.573322912836 | 3.077764573427 | -1.075914340371 |
| H | 1.556882706858 | 2.168106633405 | 0.059177712066 |
| H | -0.475102664785 | 2.998997321864 | 1.212619984830 |
| H | -1.503341379389 | 2.255990792429 | -0.002821273462 |
| H | -1.429933163707 | 0.797042113094 | 2.015722771004 |
| H | 0.330656622035 | 0.765862571931 | 1.949173644359 |
| H | 1.471639773209 | -0.429563097236 | -0.015338252425 |
| H | -1.559102790126 | -0.368642151366 | -0.172984422575 |
| O | -0.650792328991 | -1.624507821208 | 1.106546319490 |
| H | 0.097945812715 | -1.630377313988 | 1.713914457314 |
| O | 0.436972085298 | -1.458456007471 | -1.494396483894 |
| H | 0.178536143969 | -2.218015459748 | -0.957522963077 |

cyclohexanediol_H2

(E = -387.63229706 a.u.; H = -387.42180155 a.u.)

0 1

| | | | |
|---|---|---|---|
| C | -3.195897022324 | 0.059856101552 | -0.327615320153 |
| C | 3.192931610325 | 0.085210983387 | 0.325917510951 |
| C | 1.901852407088 | 0.079566551695 | -0.476281266392 |
| C | 0.649170564611 | 0.075906331900 | 0.403165408989 |
| C | -0.652083059151 | 0.070081628294 | -0.405156386284 |
| C | -1.904692813844 | 0.066201612189 | 0.474405051836 |
| H | -3.224561014476 | -0.829436452737 | -0.976037382092 |
| H | 1.900987528408 | 0.957468518858 | -1.131598687501 |
| H | 1.907488935341 | -0.799823510854 | -1.129586018585 |

| | | | |
|---|---|---|---|
| H | 0.659250075334 | 0.954017946555 | 1.061331760500 |
| H | 0.665832278001 | -0.800614786311 | 1.063319182214 |
| H | -0.668841983903 | 0.946650293791 | -1.065245783937 |
| H | -0.662120120826 | -0.807989582907 | -1.063384232802 |
| H | -1.910557315787 | 0.945681277123 | 1.127560653264 |
| H | -1.903453550850 | -0.811579743695 | 1.129859073820 |
| O | -4.282959811130 | 0.056591220095 | 0.597017998760 |
| H | 3.228166025918 | -0.801973729074 | 0.976966921754 |
| O | 4.280102181164 | 0.087457673037 | -0.598504503449 |
| H | 5.100689562828 | 0.091417907974 | -0.097908233722 |
| H | 3.222182734497 | 0.974639843661 | 0.974200421389 |
| H | -5.103643161892 | 0.053413967684 | 0.096532174979 |
| H | -3.231943807343 | 0.947113907260 | -0.978470499290 |

Ciscyclohexanediol

(E = -386.44188437 a.u.; H = -386.25373293 a.u.)

0 1

| | | | |
|---|---|---|---|
| C | -4.006268289005 | -1.960699173913 | -2.508526054866 |
| C | -3.409125366444 | -1.226355653119 | -1.301093520013 |
| C | -4.373578079878 | -1.225893456089 | -0.113815276501 |
| C | -4.788089580873 | -2.655980024466 | 0.264714790887 |
| C | -5.397124810853 | -3.385846379866 | -0.940996716281 |
| C | -4.433055820641 | -3.379551003217 | -2.135607135384 |
| H | -3.241425952854 | -1.992344600848 | -3.297951332466 |
| H | -2.473257630061 | -1.731404864036 | -1.017004583639 |
| H | -3.907787199438 | -0.718032946900 | 0.739533339040 |

| | | | |
|---|---|---|---|
| H | -5.256714408955 | -0.647669658297 | -0.401463338622 |
| H | -5.498344153592 | -2.629858199462 | 1.096301601573 |
| H | -3.908618762310 | -3.208764791405 | 0.620498033488 |
| H | -5.653839987556 | -4.414962224460 | -0.672352041030 |
| H | -6.323942091779 | -2.886032888734 | -1.237219182640 |
| H | -4.896748581032 | -3.842081745205 | -3.010862975031 |
| H | -3.535846258262 | -3.963284279596 | -1.896431139217 |
| O | -5.159649622051 | -1.266766161627 | -2.979709322846 |
| O | -3.126576290475 | 0.102164746386 | -1.764049739345 |
| H | -4.906884191062 | -0.336422692420 | -3.026228365197 |
| H | -2.944705748982 | 0.658736051225 | -1.001010584843 |

Ciscyclopropanediol

(E = -268.40579358 a.u.; H = -268.30941431 a.u.)

0 1

| | | | |
|---|---|---|---|
| C | -0.797567847682 | 0.005541794401 | -1.575690773290 |
| C | -1.727800764353 | -0.017702624147 | -0.378964340888 |
| C | -0.266814821369 | 0.271118862389 | -0.181155624521 |
| H | -0.570041838328 | -0.964909207631 | -2.003319807376 |
| H | -0.846700499505 | 0.829139514176 | -2.278981809274 |
| H | -2.441314051451 | 0.790926024893 | -0.262390227531 |
| H | 0.053446174659 | 1.276306176408 | 0.082370930711 |
| O | 0.417957863784 | -0.761345780853 | 0.490672487787 |
| H | 1.211165184600 | -0.976906233050 | -0.008956969358 |
| O | -2.224581023948 | -1.237748876050 | 0.082255837643 |
| H | -1.482879767509 | -1.682736749229 | 0.515185914681 |

Ciscyclopropanediol_H2

(E = -269.65003215 a.u.; H = -269.52893012 a.u.)

0 1

| | | | |
|---|---|---|---|
| C | -1.938715601262 | 2.762233944632 | -0.555124354086 |
| H | -2.989987578768 | 2.752458369458 | -0.227515329929 |
| H | -1.463602954013 | 3.655346499075 | -0.120147622961 |
| C | -1.871700552571 | 2.829438339024 | -2.072201124900 |
| H | -0.823963256461 | 2.827748380724 | -2.384793913982 |
| H | -2.337238669351 | 1.933300342787 | -2.491355209248 |
| C | -2.559033618176 | 4.060097554974 | -2.641045989858 |
| H | -3.618714368687 | 4.068232324089 | -2.341698322695 |
| H | -2.092000161410 | 4.970563585528 | -2.234191548833 |
| O | -1.264345582739 | 1.573776008639 | -0.147838105927 |
| H | -1.300263296720 | 1.522006153125 | 0.811332004960 |
| O | -2.428157951232 | 4.007658390228 | -4.059925246177 |
| H | -2.860245448644 | 4.782459167484 | -4.429985783714 |

Ciscyclobutanediol

(E = -307.74426259 a.u.; H = -307.61763110 a.u.)

0 1

| | | | |
|---|---|---|---|
| C | -2.058069361352 | 0.858672107785 | -1.902161135908 |
| C | -1.104433939681 | 1.099513758497 | -0.705554261587 |
| C | -2.271098479109 | 0.742162225450 | 0.253880397248 |
| C | -3.242801166059 | 1.051026564363 | -0.910747309908 |
| H | -1.993433306644 | -0.179032302124 | -2.234614443673 |

| | | | |
|---|---|---|---|
| H | -1.979737838045 | 1.528810128328 | -2.759935270761 |
| H | -0.827584908474 | 2.157550532908 | -0.617813372072 |
| H | -2.365309761024 | 1.334273289020 | 1.171293880249 |
| H | -3.605777255424 | 2.081015886565 | -0.886997366792 |
| H | -4.084620412254 | 0.364932283229 | -0.997280527959 |
| O | 0.027395325356 | 0.263023908085 | -0.521640549262 |
| H | 0.469921387599 | 0.151033121667 | -1.367783968316 |
| O | -2.281334747400 | -0.644135374456 | 0.542675027583 |
| H | -1.356865756202 | -0.926637645205 | 0.528416919887 |

Ciscyclobutanediol_H2

(E = -308.97928003 a.u.; H = -308.82821611 a.u.)

0 1

| | | | |
|---|---|---|---|
| C | -2.503791518616 | 3.749218505141 | -2.795948548576 |
| H | -1.970418335433 | 2.864132368670 | -2.439220996014 |
| H | -1.956729415206 | 4.630528629311 | -2.426853227202 |
| C | -2.534928469025 | 3.758886722610 | -4.323267676350 |
| H | -3.102946871303 | 2.885104384470 | -4.659942217463 |
| H | -3.091515304502 | 4.644042760811 | -4.660938967958 |
| C | -1.138295899225 | 3.758144299742 | -4.950916399470 |
| H | -0.580078359973 | 2.870775119993 | -4.633965148276 |
| H | -0.571940134590 | 4.629267343147 | -4.593567265828 |
| C | -1.169333624839 | 3.783031985872 | -6.478067892486 |
| H | -1.712508677265 | 2.912166779063 | -6.854467636238 |
| H | -1.706473360709 | 4.678442518379 | -6.827268980686 |
| O | -3.803938604585 | 3.670826465959 | -2.219581006330 |

| | | | |
|---|---|---|---|
| H | -4.291770361511 | 4.456064204012 | -2.486678915313 |
| O | 0.129938000160 | 3.703048702679 | -7.056205237556 |
| H | 0.626489045617 | 4.476545120214 | -6.771478869046 |

$H_2$

(E = -1.17957107 a.u.; H = -1.16619875 a.u.)

0 1

| | | | |
|---|---|---|---|
| H | -0.300405579853 | -0.001261525493 | -1.302215418325 |
| H | 0.136204948853 | 0.448690953493 | -0.901332330675 |

Azoalkane_TS-F4000

($E_F$ = -1375.67759959 a.u.; $G_F$ = -1375.29557182 a.u.)

0 1

| | | | |
|---|---|---|---|
| N | -0.569316893252 | -1.175002294660 | -0.150745983255 |
| N | 0.621696976501 | -1.346630475615 | -0.171075629653 |
| C | -1.528868069266 | -2.738504220303 | 0.009027867306 |
| C | 1.584179950658 | 0.222798609063 | -0.323531475642 |
| C | 1.073680665708 | 0.812228309500 | -1.623690524753 |
| H | 1.383911747231 | 0.207821591205 | -2.479661594341 |
| H | -0.017220005064 | 0.859983081585 | -1.604083401730 |
| H | 1.449702316816 | 1.830616073914 | -1.772542913032 |
| C | 1.174628765306 | 0.991583561517 | 0.917239240703 |
| H | 0.087316549703 | 1.086724968804 | 0.955576420934 |
| H | 1.516540874764 | 0.481964534638 | 1.821522846997 |
| H | 1.610783722812 | 1.995725400501 | 0.911427008789 |
| C | -1.111825579478 | -3.524279787289 | -1.218719513874 |

| | | | |
|---|---|---|---|
| H | -0.024169588510 | -3.617735974776 | -1.250098539834 |
| H | -1.545809978636 | -4.529175223028 | -1.200783279783 |
| H | -1.450030551221 | -3.028332051926 | -2.131920432123 |
| C | -1.028585453073 | -3.312532940831 | 1.320517245135 |
| H | -1.412864000614 | -4.325691120172 | 1.482459018776 |
| H | 0.062004262115 | -3.367680522302 | 1.307553926253 |
| H | -1.339352078725 | -2.693316408793 | 2.165680045976 |
| C | 3.194610133017 | 0.017605406504 | -0.352460854104 |
| O | 3.698726917312 | -1.073033105666 | -0.200993533629 |
| N | 3.992880540163 | 1.186829736859 | -0.516385964732 |
| H | 3.504307000385 | 2.022354419261 | -0.804984398982 |
| C | -3.139754014614 | -2.533142388221 | 0.027687924209 |
| O | -3.643005823017 | -1.442847304742 | -0.128787100715 |
| N | -3.938664160185 | -3.701729477271 | 0.192410712211 |
| H | -3.451010884745 | -4.536696077365 | 0.484141067056 |
| C | -5.443438455355 | -3.705583397968 | 0.458582041338 |
| H | -5.858683406729 | -2.879418213240 | -0.116266223261 |
| H | -5.616238087686 | -3.493697851981 | 1.517346742660 |
| C | -6.214070350122 | -5.056889713181 | 0.098543059130 |
| H | -6.187198972103 | -5.233946930797 | -0.976755175783 |
| H | -5.744712564689 | -5.905345394704 | 0.599796989751 |
| C | 5.496940005213 | 1.191591339115 | -0.786350331298 |
| H | 5.667047409449 | 0.984609114035 | -1.846507107203 |
| H | 5.913343465057 | 0.362615427683 | -0.216434153291 |
| C | 6.268904162031 | 2.540977694952 | -0.421950574420 |
| H | 5.798753306508 | 3.391854022755 | -0.918296591196 |

| | | | |
|---|---|---|---|
| H | 6.244503860559 | 2.713154908242 | 0.654191666374 |
| O | 7.721491516567 | 2.529474594179 | -0.847529509446 |
| O | -7.667554691473 | -5.043986981268 | 0.520878966724 |
| C | 8.489827918081 | 3.683455744698 | -0.626267483843 |
| O | 8.018603398939 | 4.672864818296 | -0.124884010244 |
| C | -8.435200263685 | -6.198966962730 | 0.302313271769 |
| O | -7.962733809033 | -7.190050886476 | -0.194535992793 |
| C | 9.972326134390 | 3.583700918519 | -1.073198108361 |
| H | 10.220049838575 | 2.618164687226 | -1.504514959582 |
| C | 10.959072370774 | 4.531248627215 | -0.988149088637 |
| H | 10.773513699132 | 5.513229859179 | -0.566973120855 |
| H | 11.953889365708 | 4.301877814506 | -1.348494910992 |
| C | -9.918554126137 | -6.097991619355 | 0.746067383265 |
| H | -10.167278283616 | -5.131063351481 | 1.173644463203 |
| C | -10.904930954425 | -7.046046754291 | 0.662413453172 |
| H | -10.718384401864 | -8.029391756755 | 0.244901058230 |
| H | -11.900460510732 | -6.815686904697 | 1.020137426260 |

Azoalkane_R-F4000

($E_F$ = -1375.68034482 a.u.; $G_F$ = -1375.29772992 a.u.)

0 1

| | | | |
|---|---|---|---|
| N | -0.589544527686 | -1.210631270542 | -0.160292266090 |
| N | 0.643857501830 | -1.305196978822 | -0.160313076641 |
| C | -1.367445048348 | -2.624616720985 | 0.014368117659 |
| C | 1.421757730566 | 0.108787615861 | -0.334984208843 |
| C | 0.959019019996 | 0.702408652451 | -1.666889140155 |

| | | | |
|---|---|---|---|
| H | 1.256567083677 | 0.061985324225 | -2.500972480631 |
| H | -0.128625099008 | 0.791743670316 | -1.665679038233 |
| H | 1.384889339998 | 1.695610388247 | -1.829341073555 |
| C | 1.029017456047 | 0.973518714972 | 0.863927762154 |
| H | -0.055542538373 | 1.094902853741 | 0.887315525767 |
| H | 1.349836126727 | 0.505169217505 | 1.797697079180 |
| H | 1.491940134358 | 1.960928817157 | 0.802875865687 |
| C | -0.974722827907 | -3.489324173654 | -1.184567205824 |
| H | 0.109836270252 | -3.610714515941 | -1.207969146280 |
| H | -1.437650541519 | -4.476732859545 | -1.123531953565 |
| H | -1.295548790467 | -3.020952425157 | -2.118322923266 |
| C | -0.904689647995 | -3.218266973833 | 1.346254272113 |
| H | -1.330554500223 | -4.211474338690 | 1.508687258889 |
| H | 0.182954812271 | -3.307597988058 | 1.345030017139 |
| H | -1.202230863287 | -2.577864098835 | 2.180355811758 |
| C | 3.052446225558 | -0.071572000740 | -0.357586949966 |
| O | 3.564975860641 | -1.162039125041 | -0.238677250353 |
| N | 3.843699007636 | 1.103680808620 | -0.496397965308 |
| H | 3.352053629751 | 1.946424457920 | -0.756749139738 |
| C | -2.998132976549 | -2.444253497591 | 0.036997759172 |
| O | -3.510660563852 | -1.353781735704 | -0.081873115512 |
| N | -3.789386935995 | -3.619507567204 | 0.175787739522 |
| H | -3.297739258199 | -4.462259005593 | 0.436107200139 |
| C | -5.289199167228 | -3.631203620320 | 0.465884604283 |
| H | -5.712892354427 | -2.787313750967 | -0.075999705049 |
| H | -5.444658871071 | -3.452078855808 | 1.533406311875 |

| | | | |
|---|---|---|---|
| C | -6.068099273282 | -4.969566994522 | 0.077243349123 |
| H | -6.059663719605 | -5.113525374228 | -1.003283200555 |
| H | -5.592430645172 | -5.833950300058 | 0.544155942904 |
| C | 5.343516923249 | 1.115371411848 | -0.786467645004 |
| H | 5.498997509097 | 0.936204757990 | -1.853979789905 |
| H | 5.767203974340 | 0.271504389657 | -0.244541752396 |
| C | 6.122403582413 | 2.453753800776 | -0.397864251054 |
| H | 5.646742168659 | 3.318116509794 | -0.864823597613 |
| H | 6.113943446430 | 2.597756840739 | 0.682656785378 |
| O | 7.568632990404 | 2.451107518913 | -0.844801088637 |
| O | -7.514318700206 | -4.966934880551 | 0.524212560792 |
| C | 8.343055119307 | 3.596831324836 | -0.603189159470 |
| O | 7.881981743236 | 4.572911619306 | -0.067411713379 |
| C | -8.288750847742 | -6.112644565358 | 0.282568690248 |
| O | -7.827693877228 | -7.088702619702 | -0.253262182208 |
| C | 9.818381080381 | 3.506483376288 | -1.075224605022 |
| H | 10.057088558408 | 2.552846134952 | -1.537030297055 |
| C | 10.808715207140 | 4.449200973168 | -0.978900378520 |
| H | 10.632106877017 | 5.419438178239 | -0.527722712035 |
| H | 11.797331809806 | 4.227827913706 | -1.360667298661 |
| C | -9.764064857213 | -6.022312780875 | 0.754643165523 |
| H | -10.002757136615 | -5.068696680633 | 1.216499061585 |
| C | -10.754405242596 | -6.965021721118 | 0.658299107224 |
| H | -10.577812331283 | -7.935237888422 | 0.207071157020 |
| H | -11.743011374468 | -6.743662996072 | 1.040100970496 |

Azoalkane_TS-F3000

($E_F$ = -1375.11163455 a.u.; $G_F$ = -1374.73213159 a.u.)

0 1

| | | | |
|---|---|---|---|
| N | -0.554223645580 | -1.157934536671 | -0.147728715419 |
| N | 0.608933770382 | -1.354669281489 | -0.169262556449 |
| C | -1.591380094333 | -2.823305784286 | 0.001457639655 |
| C | 1.644735623891 | 0.307936553772 | -0.320205233570 |
| C | 1.106192978496 | 0.873519416746 | -1.612115920300 |
| H | 1.435066738034 | 0.286233735744 | -2.472892633126 |
| H | 0.013888012807 | 0.881178809891 | -1.586760081438 |
| H | 1.436271538147 | 1.909459280031 | -1.762191858066 |
| C | 1.211133026668 | 1.030471352597 | 0.932333583766 |
| H | 0.121360444926 | 1.098397073627 | 0.969982459807 |
| H | 1.565630883789 | 0.512155082328 | 1.826701394992 |
| H | 1.619670174746 | 2.047973425889 | 0.949730402260 |
| C | -1.158835970378 | -3.541362108905 | -1.253900941062 |
| H | -0.069072310837 | -3.608775898664 | -1.293010571587 |
| H | -1.567036992011 | -4.558974767685 | -1.274317804443 |
| H | -1.514541251459 | -3.020166984961 | -2.146120870094 |
| C | -1.050264617957 | -3.391769384291 | 1.290833997884 |
| H | -1.377836590161 | -4.428968888063 | 1.437991534661 |
| H | 0.042050447287 | -3.397102401654 | 1.264137969293 |
| H | -1.379304516925 | -2.808001809795 | 2.153928269085 |
| C | 3.209645269990 | 0.062781900234 | -0.337249431722 |
| O | 3.702030637149 | -1.026151361228 | -0.126746130162 |
| N | 4.004670482155 | 1.206776353705 | -0.554181595541 |

| | | | |
|---|---|---|---|
| H | 3.527108140048 | 2.034141461925 | -0.881316168166 |
| C | -3.156209259342 | -2.578725298053 | 0.020657131437 |
| O | -3.649215602603 | -1.489982478332 | -0.189567924291 |
| N | -3.950727382027 | -3.722980929858 | 0.238258369378 |
| H | -3.472699799110 | -4.550214642941 | 0.565095600053 |
| C | -5.437930798136 | -3.698062634979 | 0.468527287321 |
| H | -5.837990315077 | -2.884953714039 | -0.135695154933 |
| H | -5.643195439954 | -3.462870601895 | 1.517526536402 |
| C | -6.165214361934 | -5.045752783292 | 0.115979750389 |
| H | -6.110348806712 | -5.245466993391 | -0.955166461607 |
| H | -5.701704528709 | -5.883009248565 | 0.643054918565 |
| C | 5.491997626890 | 1.181458692001 | -0.783651762058 |
| H | 5.697782906990 | 0.945305061494 | -1.832323542933 |
| H | 5.891697312551 | 0.368883736428 | -0.178485484819 |
| C | 6.219140450556 | 2.529434303288 | -0.431933997602 |
| H | 5.755809541519 | 3.366239556024 | -0.959867659766 |
| H | 6.163850798092 | 2.730029010667 | 0.639015699415 |
| O | 7.655480761043 | 2.488290874908 | -0.824271194964 |
| O | -7.601424566514 | -5.004873700418 | 0.508897981235 |
| C | 8.408776842331 | 3.639436949466 | -0.633963374198 |
| O | 7.932708173026 | 4.651709901230 | -0.181663739624 |
| C | -8.354791111260 | -6.155898943864 | 0.318172570404 |
| O | -7.878895229572 | -7.167944992221 | -0.134876702088 |
| C | 9.873711305494 | 3.496203973212 | -1.048118360621 |
| H | 10.128053294504 | 2.514121615668 | -1.434542346235 |
| C | 10.835958264149 | 4.453510553099 | -0.972769796007 |

| | | | |
|---|---|---|---|
| H | 10.617185236388 | 5.444881399960 | -0.591129695801 |
| H | 11.846324967977 | 4.236948508570 | -1.296101911626 |
| C | -9.819596165154 | -6.012860504324 | 0.732902506233 |
| H | -10.073793490525 | -5.030977725677 | 1.119981766044 |
| C | -10.781893544261 | -6.970100464929 | 0.657313311499 |
| H | -10.563265560599 | -7.961275204823 | 0.275028205131 |
| H | -11.792152783471 | -6.753685341134 | 0.981108100251 |

Azoalkane_R-F3000

($E_F$ = -1375.12543091 a.u.; $G_F$ = -1374.73928078 a.u.)

0 1

| | | | |
|---|---|---|---|
| N | -0.590782698700 | -1.220866710282 | -0.153712760579 |
| N | 0.644687902172 | -1.294904955601 | -0.163250955590 |
| C | -1.312631924069 | -2.605555861366 | 0.013013713236 |
| C | 1.366537492652 | 0.089784101631 | -0.329982109024 |
| C | 0.897631507368 | 0.698197550679 | -1.658083522794 |
| H | 1.198927437939 | 0.069962862373 | -2.499982643528 |
| H | -0.190596022814 | 0.776050119577 | -1.651820182349 |
| H | 1.311509914481 | 1.697772019258 | -1.809610536500 |
| C | 0.978663592037 | 0.959879138244 | 0.872379085392 |
| H | -0.105811059133 | 1.079273674367 | 0.897964402123 |
| H | 1.302432821876 | 0.491375707510 | 1.805006428156 |
| H | 1.441587692150 | 1.946486357742 | 0.807788466164 |
| C | -0.924767562573 | -3.475639716748 | -1.189358628741 |
| H | 0.159706589328 | -3.595037135989 | -1.214951609433 |
| H | -1.387693934549 | -4.462246225632 | -1.124775359731 |

| | | | |
|---|---|---|---|
| H | -1.248540894479 | -3.007126099846 | -2.121979183567 |
| C | -0.843717255262 | -3.213982986010 | 1.341105526925 |
| H | -1.257594188416 | -4.213559194473 | 1.492624194829 |
| H | 0.244510262660 | -3.291834772747 | 1.334834649773 |
| H | -1.145008230538 | -2.585757628979 | 2.183013080230 |
| C | 2.965469734311 | -0.107796653696 | -0.348101958356 |
| O | 3.477318814167 | -1.196969935609 | -0.197058614462 |
| N | 3.743855654840 | 1.052453942131 | -0.515927896316 |
| H | 3.258321850383 | 1.890165625472 | -0.800633634953 |
| C | -2.911563778771 | -2.407973671716 | 0.031147146309 |
| O | -3.423412776148 | -1.318797915636 | -0.119877656772 |
| N | -3.689949953429 | -3.568225531184 | 0.198963414831 |
| H | -3.204415031354 | -4.405941601847 | 0.483654101493 |
| C | -5.171504429008 | -3.557050231542 | 0.460938339967 |
| H | -5.587194269629 | -2.725656256121 | -0.106549936845 |
| H | -5.355571335477 | -3.357747029044 | 1.521340478415 |
| C | -5.904188098786 | -4.893279194646 | 0.079058114604 |
| H | -5.870220367287 | -5.058586594198 | -0.998795831992 |
| H | -5.431042730080 | -5.746994430320 | 0.570007803388 |
| C | 5.225412396992 | 1.041276880410 | -0.777889760025 |
| H | 5.409489039263 | 0.841955430289 | -1.838286841834 |
| H | 5.641098963389 | 0.209893649009 | -0.210383202874 |
| C | 5.958089953729 | 2.377514053760 | -0.396026480983 |
| H | 5.484949300164 | 3.231219619222 | -0.886997624054 |
| H | 5.924109801369 | 2.542841550230 | 0.681824076061 |
| O | 7.386327377759 | 2.348849946782 | -0.817996503363 |

| | | | |
|---|---|---|---|
| O | -7.332420547509 | -4.864621404507 | 0.501045199457 |
| C | 8.146152617436 | 3.490770831621 | -0.600866291320 |
| O | 7.682178362193 | 4.486392488956 | -0.101483704352 |
| C | -8.092251318713 | -6.006534863233 | 0.283896048324 |
| O | -7.628286002472 | -7.002144889697 | -0.215517784785 |
| C | 9.601723667712 | 3.360431324171 | -1.050905649685 |
| H | 9.845009851686 | 2.393257439901 | -1.479731576657 |
| C | 10.568151916176 | 4.312140045070 | -0.959798538736 |
| H | 10.360442645818 | 5.288775638286 | -0.536276229639 |
| H | 11.570731939437 | 4.105870313719 | -1.312727864806 |
| C | -9.547816226959 | -5.876203421307 | 0.733957641312 |
| H | -9.791094681547 | -4.909039750465 | 1.162810772319 |
| C | -10.514247754238 | -6.827907752711 | 0.642839622827 |
| H | -10.306546037400 | -7.804533407823 | 0.219291126325 |
| H | -11.516822842277 | -6.621644367613 | 0.995786425921 |

Azoalkane_TS-F2500

($E_F$ = -1374.83180495 a.u.; $G_F$ = -1374.45071493 a.u.)

0 1

| | | | |
|---|---|---|---|
| N | -0.545630845887 | -1.174753249532 | -0.070646560893 |
| N | 0.599607028169 | -1.341358711382 | -0.246896902508 |
| C | -1.573084969971 | -2.892915410009 | -0.135989265794 |
| C | 1.627172286117 | 0.377320127445 | -0.181831596791 |
| C | 1.074757699089 | 1.075242868531 | -1.397082957442 |
| H | 1.420989176167 | 0.612479090106 | -2.324306896659 |
| H | -0.017128026646 | 1.047169754239 | -1.378193496404 |

| | | | |
|---|---|---|---|
| H | 1.364298611820 | 2.135017768226 | -1.409614020174 |
| C | 1.161062670411 | 0.916023340070 | 1.146796867328 |
| H | 0.069138007199 | 0.932829839909 | 1.187039031944 |
| H | 1.541931322669 | 0.303241273906 | 1.966398210157 |
| H | 1.527146914844 | 1.940918841003 | 1.287130711120 |
| C | -1.107881756404 | -3.431057119636 | -1.465189012396 |
| H | -0.015990139411 | -3.448344473787 | -1.505995912664 |
| H | -1.474536088749 | -4.455684085649 | -1.605945356690 |
| H | -1.488877419255 | -2.817605839740 | -2.284232927944 |
| C | -1.020204933376 | -3.591715664206 | 1.078562163199 |
| H | -1.309908009540 | -4.651445657006 | 1.090542483968 |
| H | 0.071675170200 | -3.563788353054 | 1.059174527816 |
| H | -1.365937451579 | -3.129489889023 | 2.006238093143 |
| C | 3.169661311480 | 0.132379094977 | -0.141875522590 |
| O | 3.641839407449 | -0.836700777039 | 0.422885231208 |
| N | 3.975315640624 | 1.156878488149 | -0.653420119524 |
| H | 3.530337442969 | 1.846227995232 | -1.240914215452 |
| C | -3.115600068863 | -2.647738527692 | -0.174946282186 |
| O | -3.587956958289 | -1.677966957959 | -0.738357167925 |
| N | -3.921074656362 | -3.672758168500 | 0.335840372267 |
| H | -3.475839440614 | -4.362798335307 | 0.922334733909 |
| C | -5.412902501417 | -3.628857150619 | 0.388350553913 |
| H | -5.739702033105 | -2.974169609194 | -0.418642106019 |
| H | -5.739481858483 | -3.179965613626 | 1.331891175873 |
| C | -6.078748349820 | -5.035034773061 | 0.254154283899 |
| H | -5.898788987449 | -5.457062278730 | -0.736091741290 |

| | | | |
|---|---|---|---|
| H | -5.675606673420 | -5.728009846945 | 0.996937282210 |
| C | 5.467174694684 | 1.113077788236 | -0.705019267968 |
| H | 5.794352091624 | 0.663196098364 | -1.647882349020 |
| H | 5.793571082132 | 0.459297715662 | 0.102873322607 |
| C | 6.132800290741 | 2.519467049062 | -0.571958047546 |
| H | 5.730116795424 | 3.211566399872 | -1.315804259159 |
| H | 5.952110445875 | 2.942592668441 | 0.417690464239 |
| O | 7.594080494581 | 2.423460457899 | -0.782969448487 |
| O | -7.539865878288 | -4.939141382549 | 0.466320648037 |
| C | 8.316490589648 | 3.601350956860 | -0.768079109687 |
| O | 7.795633412426 | 4.677944548418 | -0.603288166153 |
| C | -8.262440470933 | -6.116913768771 | 0.450182611075 |
| O | -7.741849999299 | -7.193310212902 | 0.283310054323 |
| C | 9.807307131235 | 3.395007353651 | -0.978172112097 |
| H | 10.102826974380 | 2.359099954699 | -1.110136249642 |
| C | 10.742798509335 | 4.373634456896 | -1.007609488773 |
| H | 10.469479835378 | 5.415063560474 | -0.876882689010 |
| H | 11.786632906929 | 4.131266934118 | -1.162721300273 |
| C | -9.753060151634 | -5.910714877392 | 0.661792506184 |
| H | -10.048339796126 | -4.874984041342 | 0.795637454501 |
| C | -10.688650682481 | -6.889269400005 | 0.690450494037 |
| H | -10.415571533761 | -7.930521213538 | 0.557853732654 |
| H | -11.732327347017 | -6.647019864199 | 0.846794904417 |

Azoalkane_R-F2500

($E_F$ = -1374.85245358 a.u.; $G_F$ = -1374.46641589 a.u.)

0 1

| | | | |
|---|---|---|---|
| N | -0.582476312497 | -1.231271843349 | -0.058073752115 |
| N | 0.636505313839 | -1.284309682563 | -0.259367993232 |
| C | -1.277656105480 | -2.619685657109 | -0.033198411302 |
| C | 1.331695299439 | 0.104098906869 | -0.284203610720 |
| C | 0.855277229025 | 0.799420506228 | -1.567379080836 |
| H | 1.167591458443 | 0.244637523632 | -2.455302039205 |
| H | -0.234486671113 | 0.856255716139 | -1.556493988642 |
| H | 1.240477008331 | 1.819617779001 | -1.639213254047 |
| C | 0.945377144617 | 0.898397611985 | 0.971495607117 |
| H | -0.137694478049 | 1.020790620161 | 1.008659444626 |
| H | 1.270278155849 | 0.367346973499 | 1.869365895339 |
| H | 1.420106170686 | 1.881505238232 | 0.960968293122 |
| C | -0.891214464951 | -3.414105529428 | -1.288781592356 |
| H | 0.191862055547 | -3.536482612795 | -1.325842196898 |
| H | -1.365929226700 | -4.397219380840 | -1.278188202302 |
| H | -1.216050379925 | -2.883152126944 | -2.186732076213 |
| C | -0.801349937987 | -3.314877802141 | 1.250087911528 |
| H | -1.186552834186 | -4.335069499752 | 1.321984764896 |
| H | 0.288414695659 | -3.371716626727 | 1.239303719487 |
| H | -1.113741213679 | -2.760009145093 | 2.137929790544 |
| C | 2.914022841043 | -0.095050545418 | -0.247526299627 |
| O | 3.409226924351 | -1.126015219493 | 0.161858889422 |
| N | 3.696293814860 | 1.008341364666 | -0.601160855848 |
| H | 3.236124379693 | 1.759610479192 | -1.092715011916 |
| C | -2.859977317233 | -2.420555868783 | -0.070068203943 |

| | | | |
|---|---|---|---|
| O | -3.355135514718 | -1.389688475829 | -0.479756172366 |
| N | -3.642283776540 | -3.523866503284 | 0.283733784808 |
| H | -3.182171370178 | -4.275015655681 | 0.775524141686 |
| C | -5.131723740408 | -3.486405192652 | 0.394406725372 |
| H | -5.490174737892 | -2.776156511789 | -0.349538713325 |
| H | -5.423403857422 | -3.107518484322 | 1.379328533043 |
| C | -5.804347052283 | -4.879008476438 | 0.184486805435 |
| H | -5.660957677441 | -5.230773240175 | -0.838631620280 |
| H | -5.378604062374 | -5.623199913283 | 0.862315538211 |
| C | 5.185717964901 | 0.970889089804 | -0.712049443932 |
| H | 5.477255250640 | 0.592266092571 | -1.697114891213 |
| H | 5.544260299615 | 0.260432529855 | 0.031652889042 |
| C | 5.858395883068 | 2.363423342010 | -0.501845076156 |
| H | 5.432566800983 | 3.107808236825 | -1.179406857274 |
| H | 5.715164106463 | 2.714910134946 | 0.521390521399 |
| O | 7.310704998161 | 2.280147726003 | -0.771538008915 |
| O | -7.256697564139 | -4.795684955704 | 0.453942626055 |
| C | 8.038604515156 | 3.452462740011 | -0.698998781883 |
| O | 7.528953787815 | 4.515633235818 | -0.439519537254 |
| C | -7.984563408070 | -5.968035506197 | 0.381630438157 |
| O | -7.474852513235 | -7.031270822268 | 0.122532941440 |
| C | 9.519923427202 | 3.258129175192 | -0.976842070656 |
| H | 9.805296694305 | 2.233550076054 | -1.192765842234 |
| C | 10.458746113739 | 4.233971116079 | -0.970310037080 |
| H | 10.195629299385 | 5.264156208553 | -0.756185355471 |
| H | 11.495093266315 | 4.000601987787 | -1.179831363051 |

| | | | |
|---|---|---|---|
| C | -9.465928394922 | -5.773651530598 | 0.659193416836 |
| H | -9.751356111827 | -4.749015115510 | 0.874776179285 |
| C | -10.404729367809 | -6.749515395209 | 0.652802329098 |
| H | -10.141558382288 | -7.779756970921 | 0.439014271685 |
| H | -11.441112896941 | -6.516107529199 | 0.862100833725 |

Azoalkane_TS-F2000

($E_F$ = -1374.55461051 a.u.; $G_F$ = -1374.17203340 a.u.)

0 1

| | | | |
|---|---|---|---|
| N | -0.542598467715 | -1.190953441085 | -0.060471406217 |
| N | 0.596644090578 | -1.324733414481 | -0.257127280206 |
| C | -1.536642908903 | -2.943583225208 | -0.187234472321 |
| C | 1.590681931934 | 0.427909577919 | -0.130414157454 |
| C | 1.040602445757 | 1.137844901924 | -1.338291642096 |
| H | 1.397006033089 | 0.692842132820 | -2.270518925548 |
| H | -0.050942870893 | 1.096974473878 | -1.329485126266 |
| H | 1.318728446512 | 2.200972885236 | -1.331040376108 |
| C | 1.088131252363 | 0.916517384229 | 1.202629330804 |
| H | -0.004551118884 | 0.906623269282 | 1.224149450535 |
| H | 1.471695928194 | 0.290370161187 | 2.010132624573 |
| H | 1.425108757502 | 1.946565236083 | 1.376841088675 |
| C | -1.034165199580 | -3.432096310910 | -1.520341469567 |
| H | 0.058516309356 | -3.422228355516 | -1.541910957927 |
| H | -1.371177485860 | -4.462121208977 | -1.694622314916 |
| H | -1.417750662767 | -2.805873563101 | -2.327777260952 |
| C | -0.986503220750 | -3.653608655992 | 1.020562888089 |

| | | | |
|---|---|---|---|
| H | -1.264618493957 | -4.716739194567 | 1.013242086138 |
| H | 0.105041462909 | -3.612726449507 | 1.011710381012 |
| H | -1.342869529111 | -3.208681752153 | 1.952840742440 |
| C | 3.114313824373 | 0.172326556063 | -0.067173356300 |
| O | 3.573018382514 | -0.739353776548 | 0.599647012563 |
| N | 3.926967024492 | 1.119739630759 | -0.684846839891 |
| H | 3.492215397012 | 1.761453331084 | -1.330415767353 |
| C | -3.060278670141 | -2.687990749887 | -0.250375394439 |
| O | -3.519016253182 | -1.776243390538 | -0.917080692317 |
| N | -3.872900419547 | -3.635464222784 | 0.367247132587 |
| H | -3.438111633177 | -4.277249853237 | 1.012719422870 |
| C | -5.355407538891 | -3.585097289552 | 0.382717993756 |
| H | -5.662839110949 | -2.960297012108 | -0.454973459996 |
| H | -5.706759529593 | -3.104967450681 | 1.301915143793 |
| C | -6.002645800560 | -4.992580665137 | 0.280701149000 |
| H | -5.797013609345 | -5.447947295209 | -0.689922737060 |
| H | -5.615417537193 | -5.658621591914 | 1.056227149547 |
| C | 5.409475644849 | 1.069381876638 | -0.700211967851 |
| H | 5.760895197201 | 0.589115860847 | -1.619311917827 |
| H | 5.716857222331 | 0.444711383127 | 0.137594522188 |
| C | 6.056690879737 | 2.476887286426 | -0.598362533045 |
| H | 5.669531011423 | 3.142798179563 | -1.374032781948 |
| H | 5.850959968753 | 2.932407826374 | 0.372167850538 |
| O | 7.512746362488 | 2.366529721766 | -0.772136016276 |
| O | -7.458683390743 | -4.882234334031 | 0.454636165287 |
| C | 8.231505488114 | 3.538897556160 | -0.791172261802 |

| | | | |
|---|---|---|---|
| O | 7.711360098118 | 4.623531120589 | -0.682202872054 |
| C | -8.177459562674 | -6.054595083451 | 0.473496122324 |
| O | -7.657343333356 | -7.139215708582 | 0.364234042670 |
| C | 9.715572625418 | 3.311401365701 | -0.961674536590 |
| H | 10.011959635521 | 2.270996982663 | -1.044210458729 |
| C | 10.640513588278 | 4.292254738918 | -1.011378386506 |
| H | 10.352807452489 | 5.334703659983 | -0.928541334449 |
| H | 11.690363740304 | 4.057527234605 | -1.134771647518 |
| C | -9.661504904183 | -5.827109634078 | 0.644212138676 |
| H | -9.957865878012 | -4.786716277641 | 0.727009349575 |
| C | -10.586456474812 | -6.807959031817 | 0.693806232659 |
| H | -10.298776361920 | -7.850396662477 | 0.610708587862 |
| H | -11.636289319505 | -6.573239560590 | 0.817369108237 |

Azoalkane_R-F2000

($E_F$ = -1374.58252574 a.u.; $G_F$ = -1374.19303677 a.u.)

0 1

| | | | |
|---|---|---|---|
| N | -0.571608357797 | -1.251543851188 | -0.007387611999 |
| N | 0.625653741876 | -1.264145029930 | -0.310227442877 |
| C | -1.239741653735 | -2.636689036825 | -0.048954009347 |
| C | 1.293787070030 | 0.121000498294 | -0.268660925891 |
| C | 0.819831463872 | 0.847345509017 | -1.536630983410 |
| H | 1.141669311936 | 0.320761391074 | -2.438098190145 |
| H | -0.270549735135 | 0.893721670320 | -1.531422620098 |
| H | 1.193957461048 | 1.873574545461 | -1.574707114724 |
| C | 0.895834764768 | 0.884592618569 | 1.002970833920 |

| | | | |
|---|---|---|---|
| H | -0.187028122655 | 1.005322429313 | 1.037568444239 |
| H | 1.216381847714 | 0.331289451981 | 1.888549042913 |
| H | 1.370658071833 | 1.867728311816 | 1.016423293200 |
| C | -0.841789973967 | -3.400281783252 | -1.320585581829 |
| H | 0.241072831639 | -3.521012351486 | -1.355183308901 |
| H | -1.316613794311 | -4.383417241591 | -1.334037595562 |
| H | -1.162336786421 | -2.846978867905 | -2.206164001480 |
| C | -0.765786015750 | -3.363034076118 | 1.219016166928 |
| H | -1.139912246067 | -4.389263046029 | 1.257092462971 |
| H | 0.324595207558 | -3.409410480477 | 1.213807664014 |
| H | -1.087623640233 | -2.836449774763 | 2.120483352033 |
| C | 2.861051081326 | -0.083157379767 | -0.208071453291 |
| O | 3.339810115252 | -1.081191502681 | 0.296243728721 |
| N | 3.649261376864 | 0.977334557112 | -0.639142188424 |
| H | 3.204529121133 | 1.687913952869 | -1.199913858426 |
| C | -2.807005784165 | -2.432530301542 | -0.109542351730 |
| O | -3.285764453615 | -1.434495528495 | -0.613856336041 |
| N | -3.595216285918 | -3.493021918792 | 0.321528758563 |
| H | -3.150483365229 | -4.203602191889 | 0.882298678760 |
| C | -5.078171552241 | -3.445566816455 | 0.377803952946 |
| H | -5.410301213207 | -2.775251722827 | -0.413974801805 |
| H | -5.406818461563 | -3.018653005093 | 1.331192444955 |
| C | -5.727248773256 | -4.845010718383 | 0.213925011969 |
| H | -5.546784227580 | -5.246719162789 | -0.784992096990 |
| H | -5.322878614831 | -5.553269447230 | 0.942027387593 |
| C | 5.132217110509 | 0.929881583556 | -0.695411731469 |

| | | | |
|---|---|---|---|
| H | 5.460868367373 | 0.502960745013 | -1.648795865727 |
| H | 5.464345085909 | 0.259573037941 | 0.096373619115 |
| C | 5.781290601322 | 2.329328205780 | -0.531541077548 |
| H | 5.376925266223 | 3.037579738138 | -1.259653490352 |
| H | 5.600817313877 | 2.731045630343 | 0.467371166932 |
| O | 7.232119704588 | 2.226975957526 | -0.747173301409 |
| O | -7.178075932852 | -4.742658667334 | 0.429570360590 |
| C | 7.953567747556 | 3.397617571000 | -0.720313689128 |
| O | 7.439379789847 | 4.475391242936 | -0.539266290428 |
| C | -7.899526985485 | -5.913297489524 | 0.402695319439 |
| O | -7.385343497834 | -6.991067958713 | 0.221622248684 |
| C | 9.432132738343 | 3.177084424952 | -0.940317378732 |
| H | 9.722945544206 | 2.142159382788 | -1.086808101854 |
| C | 10.358747792493 | 4.157414197587 | -0.960106207568 |
| H | 10.076725280817 | 5.194400827090 | -0.813571440352 |
| H | 11.404365592207 | 3.927672645508 | -1.122598810124 |
| C | -9.378089300551 | -5.692766116562 | 0.622717416073 |
| H | -9.668898238011 | -4.657844288599 | 0.769230847072 |
| C | -10.304706253825 | -6.673094101469 | 0.642496638526 |
| H | -10.022687315463 | -7.710077628266 | 0.495939596219 |
| H | -11.350321967367 | -6.443354088549 | 0.805003160065 |

Azoalkane_TS-F1800

($E_F$ = -1374.44455675 a.u.; $G_F$ = -1374.06170686 a.u.)

0 1

| | | | |
|---|---|---|---|
| N | -0.541639221722 | -1.198501653775 | -0.058045300040 |

| | | | |
|---|---|---|---|
| N | 0.595687830074 | -1.317357095131 | -0.259091259097 |
| C | -1.517890476617 | -2.966972858774 | -0.204425343809 |
| C | 1.571927992970 | 0.451623401572 | -0.113221501289 |
| C | 1.021892323686 | 1.159134093675 | -1.321924645578 |
| H | 1.381248108052 | 0.715194860605 | -2.253602512770 |
| H | -0.069485840113 | 1.113667632649 | -1.315806671415 |
| H | 1.295929574140 | 2.223312681235 | -1.314295382107 |
| C | 1.053805505294 | 0.924486952172 | 1.218657352645 |
| H | -0.038866001497 | 0.900460286565 | 1.232772599613 |
| H | 1.440686933193 | 0.298410211968 | 2.024377806679 |
| H | 1.375322082138 | 1.958072486582 | 1.401640790775 |
| C | -1.000822804068 | -3.438926526507 | -1.537062517224 |
| H | 0.091836368417 | -3.415474871945 | -1.551820184794 |
| H | -1.323024730960 | -4.472160815592 | -1.720769244718 |
| H | -1.387821139188 | -2.811913581575 | -2.342012343352 |
| C | -0.967235625187 | -3.675647594898 | 1.003326272033 |
| H | -1.241306332538 | -4.739809957126 | 0.994861779038 |
| H | 0.124139742089 | -3.630203485494 | 0.996658603602 |
| H | -1.326091458800 | -3.232561998253 | 1.935599359094 |
| C | 3.088243441051 | 0.191103163252 | -0.042702015671 |
| O | 3.542151808700 | -0.700921291392 | 0.654848642746 |
| N | 3.903317554220 | 1.107453289629 | -0.696825900536 |
| H | 3.470848577688 | 1.739038213362 | -1.353634636453 |
| C | -3.034259875721 | -2.706193447686 | -0.273650447199 |
| O | -3.488517357212 | -1.812913936659 | -0.969206514816 |
| N | -3.849000738325 | -3.623640896878 | 0.379208322263 |

| | | | |
|---|---|---|---|
| H | -3.416156268727 | -4.256397928223 | 1.034595474490 |
| C | -5.327673499743 | -3.568707422728 | 0.389178497288 |
| H | -5.631055426266 | -2.944053529794 | -0.450069293846 |
| H | -5.682239245943 | -3.088458477497 | 1.307136406043 |
| C | -5.971071822886 | -4.974042122391 | 0.284612197142 |
| H | -5.762165007751 | -5.429455617104 | -0.685416618352 |
| H | -5.586790191802 | -5.640542208493 | 1.061357778686 |
| C | 5.382000254443 | 1.052695596083 | -0.705647544628 |
| H | 5.737296586220 | 0.571202833632 | -1.622676584445 |
| H | 5.684847443238 | 0.429258058051 | 0.134703542265 |
| C | 6.025154484289 | 2.458255750223 | -0.602585032308 |
| H | 5.641361195265 | 3.123617629386 | -1.380549328938 |
| H | 5.815475068489 | 2.915001808481 | 0.366657425846 |
| O | 7.478063466908 | 2.342279683193 | -0.770470116101 |
| O | -7.423832412119 | -4.858130162722 | 0.453730736782 |
| C | 8.197376559984 | 3.511275316097 | -0.793196939786 |
| O | 7.680137930797 | 4.598196194099 | -0.690961876416 |
| C | -8.143294363955 | -6.027054532334 | 0.475176592484 |
| O | -7.626286721139 | -7.113866237493 | 0.370842273474 |
| C | 9.677449083604 | 3.275401468849 | -0.958638325965 |
| H | 9.971911687030 | 2.234127119970 | -1.035098804601 |
| C | 10.599490586000 | 4.256177435254 | -1.010509159197 |
| H | 10.308341503013 | 5.298095482773 | -0.933241426219 |
| H | 11.650512573963 | 4.024565272647 | -1.130011655450 |
| C | -9.623201278107 | -5.791252906104 | 0.642112864732 |
| H | -9.917460771405 | -4.750073295497 | 0.720400403592 |

| | | | |
|---|---|---|---|
| C | -10.545332681518 | -6.771982275292 | 0.693203960689 |
| H | -10.254388324085 | -7.813804468323 | 0.614114630783 |
| H | -11.596225732159 | -6.540426554268 | 0.813882179217 |

Azoalkane_R-F1800

($E_F$ = -1374.47534590 a.u.; $G_F$ = -1374.08598648 a.u.)

0 1

| | | | |
|---|---|---|---|
| N | -0.565840612791 | -1.260924903577 | 0.012778128979 |
| N | 0.619871032725 | -1.254623437133 | -0.330181949205 |
| C | -1.223858280482 | -2.644144300870 | -0.051103439237 |
| C | 1.277882704013 | 0.128600671435 | -0.266325453176 |
| C | 0.806685460445 | 0.862499731573 | -1.531484019348 |
| H | 1.132777207977 | 0.342959419210 | -2.435463044599 |
| H | -0.283863088515 | 0.905369499393 | -1.529852993239 |
| H | 1.177446916636 | 1.890261520567 | -1.560595942518 |
| C | 0.873890942779 | 0.884019496601 | 1.008352956325 |
| H | -0.208666057385 | 1.006811778818 | 1.039257239199 |
| H | 1.189112434280 | 0.323676404456 | 1.891292509984 |
| H | 1.350735926918 | 1.866096662824 | 1.030314302527 |
| C | -0.819952573545 | -3.399481339207 | -1.325857922234 |
| H | 0.262602247531 | -3.522275951006 | -1.356839110538 |
| H | -1.296802645137 | -4.381554893167 | -1.347855212574 |
| H | -1.135228503501 | -2.839079209175 | -2.208740849839 |
| C | -0.752582086439 | -3.378124294237 | 1.213977975149 |
| H | -1.123338179064 | -4.405889216525 | 1.243049079855 |
| H | 0.337966629046 | -3.420987383053 | 1.212277415035 |

| | | | |
|---|---|---|---|
| H | -1.078622121751 | -2.858643273800 | 2.118009600465 |
| C | 2.839441664630 | -0.078742764473 | -0.197616700556 |
| O | 3.311687849963 | -1.067043084311 | 0.332723629652 |
| N | 3.630075178356 | 0.966156411481 | -0.651691027517 |
| H | 3.189626088513 | 1.666434500446 | -1.228333546929 |
| C | -2.785422571439 | -2.436790888381 | -0.119690132257 |
| O | -3.257706122805 | -1.448421077591 | -0.649864510100 |
| N | -3.576026070366 | -3.481750927835 | 0.334298572348 |
| H | -3.135535909922 | -4.182112943118 | 0.910807762237 |
| C | -5.055795202931 | -3.430642213818 | 0.377599659081 |
| H | -5.380931951856 | -2.766592130591 | -0.422335946428 |
| H | -5.393123728063 | -2.996767580310 | 1.324885176971 |
| C | -5.698182797199 | -4.829860259798 | 0.218517489348 |
| H | -5.509807435443 | -5.238584699255 | -0.776208741017 |
| H | -5.298768734480 | -5.532882554396 | 0.954573241443 |
| C | 5.109848046457 | 0.915054631267 | -0.694863432971 |
| H | 5.447259680322 | 0.481049205246 | -1.642060041396 |
| H | 5.434924210088 | 0.251120622167 | 0.105193610979 |
| C | 5.752206796831 | 2.314303434148 | -0.535926456460 |
| H | 5.352848072249 | 3.017216529849 | -1.272116514296 |
| H | 5.563740163252 | 2.723166768954 | 0.458725600214 |
| O | 7.200821564195 | 2.206590932838 | -0.740256820152 |
| O | -7.146779178649 | -4.722157914840 | 0.422988462292 |
| C | 7.921229423051 | 3.375113019380 | -0.721661954954 |
| O | 7.407844577334 | 4.455838212752 | -0.555123945705 |
| C | -7.867204646646 | -5.890667482352 | 0.404268040092 |

| | | | |
|---|---|---|---|
| O | -7.353848640000 | -6.971376466048 | 0.237508142976 |
| C | 9.396835615835 | 3.146474244817 | -0.930956381320 |
| H | 9.687477043451 | 2.109924036873 | -1.064713798064 |
| C | 10.319477112585 | 4.127751441972 | -0.955385289121 |
| H | 10.032304601804 | 5.164970358729 | -0.821040894873 |
| H | 11.367109828545 | 3.901192255387 | -1.109328181915 |
| C | -9.342790374300 | -5.662037645296 | 0.613733950379 |
| H | -9.633406383136 | -4.625500391614 | 0.747694558697 |
| C | -10.265443600402 | -6.643306823755 | 0.638080175066 |
| H | -9.978297018064 | -7.680512402419 | 0.503534333099 |
| H | -11.313060423477 | -6.416754069810 | 0.792156668412 |

Azoalkane_TS-F1500

($E_F$ = -1374.28045955 a.u.; $G_F$ = -1373.89788958 a.u.)

0 1

| | | | |
|---|---|---|---|
| N | -0.538690443887 | -1.224234091742 | -0.051264158253 |
| N | 0.595444984738 | -1.312079753463 | -0.262966621548 |
| C | -1.477610244694 | -3.007042055916 | -0.245477176905 |
| C | 1.533675109513 | 0.496498049562 | -0.076958925767 |
| C | 0.991293151112 | 1.191250304394 | -1.295300709116 |
| H | 1.363368715904 | 0.744723643863 | -2.220970643403 |
| H | -0.099598484657 | 1.135643440593 | -1.302197811373 |
| H | 1.256836790116 | 2.257726365708 | -1.290563907733 |
| C | 0.970414889419 | 0.941964071194 | 1.244007403710 |
| H | -0.120940879141 | 0.878654283082 | 1.234650717888 |
| H | 1.363938003030 | 0.326959304163 | 2.054483388805 |

| | | | |
|---|---|---|---|
| H | 1.248209056364 | 1.986440319859 | 1.437903247632 |
| C | -0.946915555149 | -3.438610109757 | -1.585517843952 |
| H | 0.145362352830 | -3.398424644174 | -1.594374594174 |
| H | -1.251427835652 | -4.472465565211 | -1.794407067455 |
| H | -1.340896672312 | -2.799181002191 | -2.376811736374 |
| C | -0.921886819635 | -3.732785286562 | 0.949205017989 |
| H | -1.195265969197 | -4.796808092777 | 0.924854756967 |
| H | 0.169319974768 | -3.684888541420 | 0.940313254752 |
| H | -1.276767147958 | -3.303923276736 | 1.889747367379 |
| C | 3.038626368707 | 0.237291333416 | 0.021706470588 |
| O | 3.484062865767 | -0.592737532075 | 0.799411338867 |
| N | 3.860283012630 | 1.074495683657 | -0.716130843088 |
| H | 3.435088001099 | 1.665478963575 | -1.413789483227 |
| C | -2.983837280612 | -2.735507135168 | -0.312007611352 |
| O | -3.435059772463 | -1.872247506883 | -1.048349311652 |
| N | -3.798085038608 | -3.599405153603 | 0.402399988311 |
| H | -3.365158385056 | -4.217212236143 | 1.071496602778 |
| C | -5.271003161951 | -3.536005561989 | 0.411596106043 |
| H | -5.571057217601 | -2.904759486580 | -0.423897112700 |
| H | -5.624991618377 | -3.062107596210 | 1.333105004621 |
| C | -5.911383135758 | -4.936579407855 | 0.295346177454 |
| H | -5.701362021215 | -5.385327940282 | -0.677773896295 |
| H | -5.529883916520 | -5.609789906245 | 1.067917285325 |
| C | 5.333167707136 | 1.017455728944 | -0.699888789156 |
| H | 5.703797026026 | 0.515478023416 | -1.599727516857 |
| H | 5.621833836696 | 0.414907142074 | 0.160407895504 |

| | | | |
|---|---|---|---|
| C | 5.967002951588 | 2.423493662362 | -0.618672048214 |
| H | 5.597282372603 | 3.069468388748 | -1.419564260143 |
| H | 5.738364467326 | 2.903110005015 | 0.335329889790 |
| O | 7.417573758732 | 2.298315227849 | -0.756133511828 |
| O | -7.358703048882 | -4.811561495673 | 0.462781085998 |
| C | 8.136301278175 | 3.462484580162 | -0.804115379228 |
| O | 7.620744891504 | 4.553833597607 | -0.746372425581 |
| C | -8.080734195059 | -5.974636386469 | 0.482100581472 |
| O | -7.570120075923 | -7.064641079415 | 0.375541159372 |
| C | 9.612889967738 | 3.212989400744 | -0.935935736084 |
| H | 9.906838539168 | 2.169637953033 | -0.972974958519 |
| C | 10.529905319902 | 4.193561285707 | -1.003604537297 |
| H | 10.231374446058 | 5.235537147210 | -0.965008462122 |
| H | 11.584381838472 | 3.965621979028 | -1.097442977696 |
| C | -9.553709639096 | -5.725762200793 | 0.650191576929 |
| H | -9.843258743898 | -4.683557509506 | 0.730057546620 |
| C | -10.472705620572 | -6.705567072806 | 0.699949365364 |
| H | -10.178552035684 | -7.746346538124 | 0.618548243588 |
| H | -11.524455802159 | -6.478166547137 | 0.821635948218 |

Azoalkane_R-F1500

($E_F$ = -1374.31547436 a.u.; $G_F$ = -1373.92454611 a.u.)

0 1

| | | | |
|---|---|---|---|
| N | -0.555083434582 | -1.279577318758 | 0.044644672278 |
| N | 0.609963643246 | -1.232257472658 | -0.356811052036 |
| C | -1.194549974850 | -2.660115000820 | -0.072753544020 |

| | | | |
|---|---|---|---|
| C | 1.249428738575 | 0.148280060073 | -0.239411936004 |
| C | 0.781095499216 | 0.916013725478 | -1.485471679517 |
| H | 1.117867465959 | 0.428339273968 | -2.403245385681 |
| H | -0.309778045989 | 0.947493661650 | -1.491684178205 |
| H | 1.141374191349 | 1.948117611542 | -1.475527954209 |
| C | 0.834904578263 | 0.868333700247 | 1.052376459829 |
| H | -0.246883158808 | 0.996771926271 | 1.077702663317 |
| H | 1.139371728558 | 0.280483466192 | 1.920625795046 |
| H | 1.316943068799 | 1.846890753472 | 1.105558699210 |
| C | -0.780025004044 | -3.380169522011 | -1.364541035332 |
| H | 0.301762784373 | -3.508606984235 | -1.389866533989 |
| H | -1.262062910773 | -4.358726897606 | -1.417722692227 |
| H | -1.084492052615 | -2.792320140301 | -2.232791021092 |
| C | -0.726217771178 | -3.427847464594 | 1.173307102373 |
| H | -1.086497040517 | -4.459951120959 | 1.163364389978 |
| H | 0.364655708488 | -3.459327967107 | 1.179520091285 |
| H | -1.062989879022 | -2.940171792687 | 2.091080168420 |
| C | 2.802566324554 | -0.062353600022 | -0.156462062063 |
| O | 3.261470133270 | -1.018016366564 | 0.443263727369 |
| N | 3.599666012159 | 0.939784243224 | -0.675906282555 |
| H | 3.169514945658 | 1.601589726904 | -1.303217494022 |
| C | -2.747687123457 | -2.449480122764 | -0.155704687811 |
| O | -3.206590143801 | -1.493818119990 | -0.755432415529 |
| N | -3.544787722154 | -3.451616773037 | 0.363740730845 |
| H | -3.114637599867 | -4.113421353924 | 0.991053654095 |
| C | -5.019905796260 | -3.397555984665 | 0.372967628948 |

| | | | |
|---|---|---|---|
| H | -5.327372382013 | -2.760484903482 | -0.455385474699 |
| H | -5.379538030947 | -2.934636361856 | 1.298081961095 |
| C | -5.648561493251 | -4.800665574610 | 0.243653887075 |
| H | -5.434277351605 | -5.240810059057 | -0.732441051437 |
| H | -5.265722293356 | -5.478331930537 | 1.011757510350 |
| C | 5.074784018789 | 0.885724278964 | -0.685134265729 |
| H | 5.434415772077 | 0.422805901092 | -1.610249284317 |
| H | 5.382251470200 | 0.248652426808 | 0.143217757400 |
| C | 5.703438877766 | 2.288833784737 | -0.555819328141 |
| H | 5.320599342317 | 2.966500804928 | -1.323922047291 |
| H | 5.489155177955 | 2.728977245108 | 0.420276053502 |
| O | 7.151413456104 | 2.172047234772 | -0.721025429227 |
| O | -7.096536101260 | -4.683878411885 | 0.408859285594 |
| C | 7.868147394259 | 3.338629631979 | -0.734250916000 |
| O | 7.352225009748 | 4.425693345713 | -0.624304307147 |
| C | -7.813269958256 | -5.850461078980 | 0.422085467143 |
| O | -7.297347360323 | -6.937524845311 | 0.312140100760 |
| C | 9.342406389460 | 3.096521513177 | -0.900368482141 |
| H | 9.636228442232 | 2.055819786187 | -0.983667819298 |
| C | 10.257659040280 | 4.080074493405 | -0.944601201292 |
| H | 9.959375023560 | 5.119392818475 | -0.859949027728 |
| H | 11.310491969257 | 3.857192478276 | -1.065104726457 |
| C | -9.287529086849 | -5.608352691267 | 0.588202081708 |
| H | -9.581351236877 | -4.567650742486 | 0.671500191966 |
| C | -10.202781704438 | -6.591905663078 | 0.632435377565 |
| H | -9.904497566964 | -7.631224123544 | 0.547784445171 |

H -11.255614704047 -6.369023463939 0.752938150119

Azoalkane_TS-F1000

($E_F$ = -1374.01007837 a.u.; $G_F$ = -1373.62752923 a.u.)

0 1

| | | | |
|---|---|---|---|
| N | -0.537047256857 | -1.246099201362 | -0.045662033523 |
| N | 0.590974281696 | -1.269483650121 | -0.271848151052 |
| C | -1.388163603791 | -3.092711357979 | -0.309586053511 |
| C | 1.441910035162 | 0.577460512521 | -0.008978558314 |
| C | 0.891867531645 | 1.277722527184 | -1.219887877050 |
| H | 1.261012642492 | 0.839482044184 | -2.150798998092 |
| H | -0.198468796370 | 1.213151902954 | -1.225154582806 |
| H | 1.151095542050 | 2.345491571255 | -1.208391298274 |
| C | 0.840246366766 | 0.961447662383 | 1.311848349966 |
| H | -0.247086838077 | 0.850118764448 | 1.283928127607 |
| H | 1.250696477248 | 0.345321982389 | 2.112255126080 |
| H | 1.065857577399 | 2.012611793002 | 1.535205527445 |
| C | -0.787940245574 | -3.474955963122 | -1.631593253863 |
| H | 0.299459002256 | -3.364077065319 | -1.604578071234 |
| H | -1.014173397511 | -4.525698631649 | -1.856297467513 |
| H | -1.198946002776 | -2.857494233080 | -2.430692224839 |
| C | -0.836838331555 | -3.794637610395 | 0.899771527684 |
| H | -1.095894036329 | -4.862435579765 | 0.886985142743 |
| H | 0.253494450367 | -3.729886359211 | 0.904062918297 |
| H | -1.205144434133 | -3.357807505527 | 1.831673055757 |
| C | 2.931651834817 | 0.307397239682 | 0.093347214668 |

| | | | |
|---|---|---|---|
| O | 3.377105159332 | -0.449041327028 | 0.945866958350 |
| N | 3.748693751211 | 1.022553850953 | -0.757155299672 |
| H | 3.322773466462 | 1.585703066584 | -1.476252132880 |
| C | -2.878018151109 | -2.822487834987 | -0.409978684480 |
| O | -3.324328822015 | -2.064728586105 | -1.260870530307 |
| N | -3.694196730595 | -3.538937481068 | 0.440263280490 |
| H | -3.267539793635 | -4.103302183066 | 1.157967045687 |
| C | -5.157972270131 | -3.465389015207 | 0.429372787585 |
| H | -5.443992532430 | -2.841631016644 | -0.416330778452 |
| H | -5.521133061206 | -2.984218824573 | 1.343202189652 |
| C | -5.791415023692 | -4.861265587958 | 0.314345086356 |
| H | -5.563266653801 | -5.319941974333 | -0.650316635316 |
| H | -5.427298159159 | -5.527831493299 | 1.101019317607 |
| C | 5.212463972761 | 0.949157934436 | -0.744538911258 |
| H | 5.576684214923 | 0.465991986639 | -1.656894774183 |
| H | 5.497640884075 | 0.327311448769 | 0.102858447280 |
| C | 5.845590688957 | 2.345372567022 | -0.631921961676 |
| H | 5.482332684202 | 3.010107100471 | -1.420540551794 |
| H | 5.616212255082 | 2.806198065592 | 0.331425284317 |
| O | 7.287246623582 | 2.202726011399 | -0.767123283810 |
| O | -7.232886162616 | -4.718713401352 | 0.451618092756 |
| C | 8.013134693910 | 3.355881752391 | -0.803987166864 |
| O | 7.510908145529 | 4.453309862999 | -0.735182122671 |
| C | -7.958934925244 | -5.871824605489 | 0.486511033955 |
| O | -7.456994103915 | -6.969165744253 | 0.414346539644 |
| C | 9.477856307925 | 3.082321473323 | -0.939945477987 |

| | | | |
|---|---|---|---|
| H | 9.761519972590 | 2.036841541283 | -0.985684426844 |
| C | 10.392043696050 | 4.059638573343 | -1.001068245113 |
| H | 10.091063165017 | 5.100500545235 | -0.952744542007 |
| H | 11.447234925936 | 3.836347257746 | -1.098998073410 |
| C | -9.423435862639 | -5.598342044951 | 0.624980139994 |
| H | -9.706846049629 | -4.552929631705 | 0.673691036687 |
| C | -10.337729003329 | -6.575639448256 | 0.684811328016 |
| H | -10.037005095219 | -7.616431283966 | 0.633505416630 |
| H | -11.392754090702 | -6.352401224350 | 0.784628558405 |

Azoalkane_R-F1000

($E_F$ = -1374.05136271 a.u.; $G_F$ = -1373.66025679 a.u.)

0 1

| | | | |
|---|---|---|---|
| N | -0.522246857256 | -1.323195630087 | 0.111420467273 |
| N | 0.576228115725 | -1.192402329876 | -0.428741578611 |
| C | -1.134977169022 | -2.696757344427 | -0.057372956912 |
| C | 1.188957555654 | 0.181159333609 | -0.259948442915 |
| C | 0.735433072023 | 0.959478607207 | -1.505766134091 |
| H | 1.085665003363 | 0.481279448964 | -2.423266901244 |
| H | -0.355525164177 | 0.986315083519 | -1.528495524925 |
| H | 1.090805480323 | 1.992894510138 | -1.478704677055 |
| C | 0.752404229089 | 0.888650874885 | 1.030597339874 |
| H | -0.327710579094 | 1.030724391019 | 1.037875990250 |
| H | 1.031185024319 | 0.287577157691 | 1.897833459650 |
| H | 1.244724568099 | 1.861120403536 | 1.103148885003 |
| C | -0.698428481912 | -3.404245532432 | -1.347922045715 |

| | | | |
|---|---|---|---|
| H | 0.381686410556 | -3.546317929787 | -1.355205216527 |
| H | -1.190748224492 | -4.376715337343 | -1.420473984658 |
| H | -0.977213276404 | -2.803169986772 | -2.215155623537 |
| C | -0.681448031175 | -3.475079292287 | 1.188441283238 |
| H | -1.036819875929 | -4.508495384012 | 1.161378711113 |
| H | 0.409510322204 | -3.501915037456 | 1.211166873780 |
| H | -1.031677253677 | -2.996882480469 | 2.105944380672 |
| C | 2.729365417990 | -0.040938655373 | -0.154908725138 |
| O | 3.169215069063 | -0.969926821729 | 0.501153416608 |
| N | 3.535983130714 | 0.910735317867 | -0.731739964406 |
| H | 3.116030695036 | 1.553695840705 | -1.384590387102 |
| C | -2.675385483098 | -2.474658760582 | -0.162406207847 |
| O | -3.115237685806 | -1.545667344202 | -0.818462049648 |
| N | -3.482000827890 | -3.426335798527 | 0.414423502386 |
| H | -3.062045200300 | -4.069300426540 | 1.067267866582 |
| C | -4.948593602051 | -3.360395938290 | 0.399309934276 |
| H | -5.239087921879 | -2.720806214450 | -0.433124167784 |
| H | -5.322904021205 | -2.899692676586 | 1.319750396034 |
| C | -5.567278454305 | -4.757980298845 | 0.254693071154 |
| H | -5.334319338434 | -5.196626804385 | -0.718090506100 |
| H | -5.201209740050 | -5.438231863411 | 1.028947842967 |
| C | 5.002575889357 | 0.844796107649 | -0.716617952914 |
| H | 5.376891624332 | 0.384081025721 | -1.637050311966 |
| H | 5.293066235660 | 0.205217407443 | 0.115826008510 |
| C | 5.621258791686 | 2.242382787455 | -0.572015770253 |
| H | 5.255197091521 | 2.922622913830 | -1.346283804499 |

| | | | |
|---|---|---|---|
| H | 5.388290209907 | 2.681043147600 | 0.400759311234 |
| O | 7.063256239785 | 2.111185725592 | -0.709389852276 |
| O | -7.009274517717 | -4.626784297754 | 0.392083137091 |
| C | 7.782339247946 | 3.269392119630 | -0.729568308093 |
| O | 7.273368907756 | 4.362368193272 | -0.643526904179 |
| C | -7.728358795186 | -5.784990171758 | 0.412243173170 |
| O | -7.219390752996 | -6.877965007262 | 0.326171552678 |
| C | 9.248186660156 | 3.005798662871 | -0.871360142649 |
| H | 9.537240961077 | 1.962634740015 | -0.933027961019 |
| C | 10.157132742943 | 3.988711983615 | -0.919412219843 |
| H | 9.850860277641 | 5.027173740308 | -0.855509886174 |
| H | 11.213292715682 | 3.772348355335 | -1.022203757568 |
| C | -9.194204327538 | -5.521397965368 | 0.554057110394 |
| H | -9.483256512241 | -4.478235023320 | 0.615752372887 |
| C | -10.103151242778 | -6.504311136578 | 0.602096691544 |
| H | -9.796880935369 | -7.542771893387 | 0.538166828515 |
| H | -11.159309772318 | -6.287948377112 | 0.704905115037 |

Azoalkane_TS-F800

($E_F$ = -1373.90322434 a.u.; $G_F$ = -1373.52157252 a.u.)

0 1

| | | | |
|---|---|---|---|
| N | -0.534315412513 | -1.265880813352 | -0.038251719703 |
| N | 0.588382636522 | -1.249824884851 | -0.279300993043 |
| C | -1.332160881081 | -3.136127760289 | -0.339689116182 |
| C | 1.386135692665 | 0.620701945567 | 0.021860479357 |
| C | 0.835342493043 | 1.313751860826 | -1.192603205371 |

| | | | |
|---|---|---|---|
| H | 1.202147690307 | 0.870139552476 | -2.121974253865 |
| H | -0.254878297610 | 1.248328147902 | -1.197511889549 |
| H | 1.095624672207 | 2.381142905086 | -1.187476051830 |
| C | 0.756875550276 | 0.978622838966 | 1.335549515318 |
| H | -0.325936919253 | 0.831972794960 | 1.294088914110 |
| H | 1.178262955813 | 0.374050074893 | 2.138592197020 |
| H | 0.943909700001 | 2.036030361685 | 1.564894591709 |
| C | -0.703491534802 | -3.493589184366 | -1.653800960285 |
| H | 0.379374722411 | -3.347239925023 | -1.612682606732 |
| H | -0.890897965931 | -4.550836739345 | -1.883581600138 |
| H | -1.125028761159 | -2.888523603053 | -2.456398769255 |
| C | -0.780945817940 | -3.829760789927 | 0.874253923901 |
| H | -1.041199632810 | -4.897156353469 | 0.868694425017 |
| H | 0.309275921309 | -3.764315800684 | 0.878823997397 |
| H | -1.147454807126 | -3.386605793131 | 1.803958766809 |
| C | 2.870614174412 | 0.354193394778 | 0.133661584523 |
| O | 3.319572120454 | -0.345878472889 | 1.032750369780 |
| N | 3.682508376385 | 0.990502596416 | -0.778404287463 |
| H | 3.255571762314 | 1.525421752276 | -1.517828901753 |
| C | -2.816684393531 | -2.869491193892 | -0.450801362725 |
| O | -3.265904138480 | -2.168704077758 | -1.349202669690 |
| N | -3.628287950191 | -3.506510404278 | 0.461029409226 |
| H | -3.201114338582 | -4.042004346903 | 1.199908546659 |
| C | -5.088534352215 | -3.425854500714 | 0.451445315605 |
| H | -5.373376161490 | -2.789818518275 | -0.385275031412 |
| H | -5.447354062642 | -2.957642048498 | 1.373330104939 |

| | | | |
|---|---|---|---|
| C | -5.724137223021 | -4.816071756626 | 0.315589235205 |
| H | -5.496631137451 | -5.261222447442 | -0.655647266062 |
| H | -5.363326090306 | -5.495239077806 | 1.092830018618 |
| C | 5.142752855368 | 0.909914623746 | -0.768252969088 |
| H | 5.501916661626 | 0.440960291213 | -1.689628320543 |
| H | 5.427336949940 | 0.274580321270 | 0.069089193707 |
| C | 5.778234035814 | 2.300277297536 | -0.633312464098 |
| H | 5.417699340433 | 2.978768438680 | -1.411274807009 |
| H | 5.550309118655 | 2.746233482709 | 0.337458392375 |
| O | 7.216188402011 | 2.149540892680 | -0.770322482329 |
| O | -7.162025755406 | -4.665372550155 | 0.453314010985 |
| C | 7.946636365190 | 3.297312091640 | -0.797283988761 |
| O | 7.451679489074 | 4.397429506518 | -0.716664599459 |
| C | -7.892531820334 | -5.813122825129 | 0.479588212448 |
| O | -7.397675845144 | -6.913196613428 | 0.397804156237 |
| C | 9.406000527856 | 3.012845283716 | -0.939579177934 |
| H | 9.683784412685 | 1.966433297567 | -0.995437146313 |
| C | 10.320574326566 | 3.987707317347 | -0.994504437847 |
| H | 10.020576839450 | 5.028309856759 | -0.935698998287 |
| H | 11.375446888544 | 3.765061452593 | -1.097651191376 |
| C | -9.351816526305 | -5.528692228379 | 0.622750802775 |
| H | -9.629509058911 | -4.482313965737 | 0.679636526096 |
| C | -10.266428759352 | -6.503543275465 | 0.677223318876 |
| H | -9.966524236430 | -7.544111168866 | 0.617386246987 |
| H | -11.321241885908 | -6.280922088009 | 0.781016377282 |

Azoalkane_R-F800

($E_F$ = -1373.94659685 a.u.; $G_F$ = -1373.55543222 a.u.)

0 1

| | | | |
|---|---|---|---|
| N | -0.506268113185 | -1.343017794726 | 0.135668679850 |
| N | 0.560308838229 | -1.172614264387 | -0.453188119512 |
| C | -1.105492208561 | -2.714965966241 | -0.054816785292 |
| C | 1.159532871638 | 0.199333909867 | -0.262702726455 |
| C | 0.711130428670 | 0.984967983788 | -1.505722700781 |
| H | 1.066886351131 | 0.513547817832 | -2.424531814952 |
| H | -0.379786967028 | 1.008310333411 | -1.534678122498 |
| H | 1.063133049329 | 2.019198664803 | -1.468904574341 |
| C | 0.712143002924 | 0.896404591472 | 1.029358065158 |
| H | -0.367648623380 | 1.041649303700 | 1.029069565576 |
| H | 0.981660065984 | 0.288189896595 | 1.894194462949 |
| H | 1.205853129141 | 1.867439012819 | 1.113040201001 |
| C | -0.658103826419 | -3.412035692385 | -1.346878592471 |
| H | 0.421687813208 | -3.557280209552 | -1.346591498588 |
| H | -1.151813884643 | -4.383070138434 | -1.430560820759 |
| H | -0.927622039227 | -2.803820449441 | -2.211714243401 |
| C | -0.657088137451 | -3.500600796562 | 1.188202120818 |
| H | -1.009090417148 | -4.534831584115 | 1.151383651142 |
| H | 0.433829297348 | -3.523942754056 | 1.217156323795 |
| H | -1.012843252550 | -3.029181441660 | 2.107011960392 |
| C | 2.695602516175 | -0.026065286056 | -0.148679520354 |
| O | 3.128195830186 | -0.939146748397 | 0.534907907057 |
| N | 3.505578922459 | 0.898699563368 | -0.756672532757 |

| | | | |
|---|---|---|---|
| H | 3.088224247471 | 1.533271494780 | -1.419080012920 |
| C | -2.641562024066 | -2.489566826073 | -0.168837833653 |
| O | -3.074156337041 | -1.576484138069 | -0.852422946157 |
| N | -3.451537509251 | -3.414332997296 | 0.439154415691 |
| H | -3.034181755991 | -4.048906391069 | 1.101559807530 |
| C | -4.914757276239 | -3.343089318675 | 0.418230773335 |
| H | -5.199780293770 | -2.694394942365 | -0.408937727567 |
| H | -5.292004523433 | -2.892000329159 | 1.342172968146 |
| C | -5.531280993715 | -4.736539699073 | 0.255621150242 |
| H | -5.293936064022 | -5.165311540187 | -0.720601899396 |
| H | -5.170530266797 | -5.425590070315 | 1.024650164585 |
| C | 4.968798648991 | 0.827455949783 | -0.735746206851 |
| H | 5.346047495036 | 0.376363397434 | -1.659685881006 |
| H | 5.253820276909 | 0.178764876620 | 0.091425226550 |
| C | 5.585321999229 | 2.220906895776 | -0.573140907551 |
| H | 5.224573248157 | 2.909953974290 | -1.342173610270 |
| H | 5.347974600667 | 2.649682729115 | 0.403079654725 |
| O | 7.024463301661 | 2.083771857795 | -0.704707250198 |
| O | -6.970421983099 | -4.599405070662 | 0.387191757225 |
| C | 7.745967548951 | 3.237908826074 | -0.718096013487 |
| O | 7.241577458209 | 4.332886676173 | -0.628309139756 |
| C | -7.691926454945 | -5.753541881931 | 0.400576199673 |
| O | -7.187536847266 | -6.848519266879 | 0.310782107915 |
| C | 9.207607180960 | 2.965430890493 | -0.858510095036 |
| H | 9.493025872006 | 1.921615652040 | -0.923459925306 |
| C | 10.115274745468 | 3.947302556070 | -0.901832615855 |

| | | | |
|---|---|---|---|
| H | 9.807742016790 | 4.985166276358 | -0.834434443199 |
| H | 11.171956806586 | 3.733061102750 | -1.004143606453 |
| C | -9.153565658163 | -5.481064403700 | 0.540995610828 |
| H | -9.438983928367 | -4.437249455175 | 0.605951910878 |
| C | -10.061233337334 | -6.462936097452 | 0.584315193064 |
| H | -9.753701038217 | -7.500799526470 | 0.516910521832 |
| H | -11.117915066366 | -6.248694958883 | 0.686630165538 |

Azoalkane_TS-F600

($E_F$ = -1373.79738522 a.u.; $G_F$ = -1373.41545887 a.u.)

0 1

| | | | |
|---|---|---|---|
| N | -0.529392916855 | -1.293129361878 | -0.025807391534 |
| N | 0.583486214742 | -1.222632223642 | -0.291401848392 |
| C | -1.251661511993 | -3.192254993784 | -0.363841444737 |
| C | 1.305716504839 | 0.676557589903 | 0.046549775699 |
| C | 0.749031181715 | 1.351912344854 | -1.175166334634 |
| H | 1.111977851123 | 0.896973129394 | -2.100722157098 |
| H | -0.340846287921 | 1.282175539519 | -1.176598995967 |
| H | 1.007363554961 | 2.419500988737 | -1.185236770428 |
| C | 0.645676145244 | 1.007069151382 | 1.350425165146 |
| H | -0.429176391841 | 0.812990022335 | 1.295617442675 |
| H | 1.083448232755 | 0.423765961830 | 2.159970732983 |
| H | 0.781820042993 | 2.072143414695 | 1.579706337721 |
| C | -0.591779217923 | -3.522642914076 | -1.667830432706 |
| H | 0.483091061954 | -3.328635016580 | -1.613112224739 |
| H | -0.728012837787 | -4.587680840406 | -1.897233601538 |

| | | | |
|---|---|---|---|
| H | -1.029604502864 | -2.939209169354 | -2.477255146989 |
| C | -0.694856494482 | -3.867764651818 | 0.857736666018 |
| H | -0.953154667078 | -4.935364019077 | 0.867682908559 |
| H | 0.395019600029 | -3.798001574177 | 0.859085217117 |
| H | -1.057739098801 | -3.412960063056 | 1.783383635429 |
| C | 2.787031379492 | 0.425107487187 | 0.167638987160 |
| O | 3.248691457375 | -0.187871208086 | 1.123702427377 |
| N | 3.585775563703 | 0.950974995550 | -0.819626056592 |
| H | 3.154780834546 | 1.442826577937 | -1.585693208370 |
| C | -2.732987544241 | -2.940756230765 | -0.484739056785 |
| O | -3.194729271876 | -2.327536051729 | -1.440608146417 |
| N | -3.531639874421 | -3.466842537662 | 0.502481108091 |
| H | -3.100583720578 | -3.958974025528 | 1.268333664388 |
| C | -4.988392694988 | -3.376727329222 | 0.499574797379 |
| H | -5.274319140768 | -2.703599744902 | -0.307122835476 |
| H | -5.336745923899 | -2.949332354811 | 1.444314654450 |
| C | -5.632573442026 | -4.753334056209 | 0.303321429213 |
| H | -5.414408753589 | -5.153498636079 | -0.689531017219 |
| H | -5.272402657122 | -5.470509455541 | 1.045521171550 |
| C | 5.042533296929 | 0.860927723851 | -0.816505377705 |
| H | 5.391037856033 | 0.432640404475 | -1.760791844432 |
| H | 5.328399655242 | 0.188587939091 | -0.009126652033 |
| C | 5.686577679979 | 2.237769774328 | -0.621488054655 |
| H | 5.326655074079 | 2.954140034861 | -1.364588146586 |
| H | 5.468016124045 | 2.638981064381 | 0.370857709824 |
| O | 7.119462055322 | 2.077382463285 | -0.774131741194 |

| | | | |
|---|---|---|---|
| O | -7.065391676879 | -4.593030438695 | 0.456696611729 |
| C | 7.858987562736 | 3.216813413218 | -0.772521700537 |
| O | 7.377005928381 | 4.319218954009 | -0.651893075210 |
| C | -7.805007308239 | -5.732401790899 | 0.453926483512 |
| O | -7.323169391412 | -6.834690749411 | 0.331620061981 |
| C | 9.310381537994 | 2.918839819533 | -0.940637023236 |
| H | 9.577069239983 | 1.872105031609 | -1.031142434202 |
| C | 10.229188117425 | 3.888119336240 | -0.977586079457 |
| H | 9.935571492194 | 4.927979101333 | -0.883837418804 |
| H | 11.281668511906 | 3.663926017754 | -1.100449348340 |
| C | -9.256297970519 | -5.434526663665 | 0.623128400604 |
| H | -9.522855458075 | -4.387883539945 | 0.715123121050 |
| C | -10.175165530244 | -6.403779096651 | 0.659291706422 |
| H | -9.881682056663 | -7.443545953970 | 0.564052169142 |
| H | -11.227566499218 | -6.179654417618 | 0.782970545649 |

Azoalkane_R-F600

($E_F$ = -1373.84240219 a.u.; $G_F$ = -1373.45196656 a.u.)

0 1

| | | | |
|---|---|---|---|
| N | -0.484419350514 | -1.365653796109 | 0.164575285741 |
| N | 0.538619908620 | -1.150210065828 | -0.481932673944 |
| C | -1.066739990701 | -2.738941756490 | -0.038038799065 |
| C | 1.120922196741 | 0.223090048569 | -0.279293000645 |
| C | 0.671473839213 | 1.008862204712 | -1.521620953916 |
| H | 1.028692062486 | 0.539455353321 | -2.440855027445 |
| H | -0.419518731121 | 1.027600533570 | -1.552245382428 |

| | | | |
|---|---|---|---|
| H | 1.019296522334 | 2.044405007350 | -1.482110199454 |
| C | 0.665135589339 | 0.914331739373 | 1.012336133180 |
| H | -0.415168217274 | 1.057277520766 | 1.008337789631 |
| H | 0.932543391226 | 0.305283957238 | 1.876780996198 |
| H | 1.155265188901 | 1.887074672058 | 1.099630532933 |
| C | -0.610368807262 | -3.430551790429 | -1.329260798597 |
| H | 0.469926417030 | -3.573566148968 | -1.324700576767 |
| H | -1.100519952348 | -4.403286456194 | -1.416525103044 |
| H | -0.877319643376 | -2.821719458276 | -2.193994769925 |
| C | -0.617986865465 | -3.524437152133 | 1.204710583354 |
| H | -0.965972302406 | -4.559928790324 | 1.165328720330 |
| H | 0.472988980253 | -3.543358235264 | 1.235841702876 |
| H | -0.975538553831 | -3.054708378838 | 2.123649612456 |
| C | 2.654155314622 | -0.002372920326 | -0.160609982447 |
| O | 3.083313566379 | -0.887316800416 | 0.561833303265 |
| N | 3.463657824396 | 0.881684358146 | -0.820996544448 |
| H | 3.043513893241 | 1.504754560784 | -1.492192797155 |
| C | -2.599899056877 | -2.513401122496 | -0.157563058984 |
| O | -3.028625357233 | -1.628808608419 | -0.880699442477 |
| N | -3.409811280894 | -3.397035178794 | 0.502881077219 |
| H | -2.990102782718 | -4.019661507795 | 1.174767474589 |
| C | -4.869681825644 | -3.321031630678 | 0.487386392702 |
| H | -5.152735836133 | -2.615839638315 | -0.292642564713 |
| H | -5.242760636888 | -2.935644015614 | 1.441926354423 |
| C | -5.488558042827 | -4.696465600223 | 0.229192874731 |
| H | -5.263248751888 | -5.052568700811 | -0.778715652343 |

| | | | |
|---|---|---|---|
| H | -5.121686964460 | -5.441026119867 | 0.941095372212 |
| C | 4.923535147065 | 0.805694799681 | -0.806518242490 |
| H | 5.295978751975 | 0.421885004667 | -1.761938071100 |
| H | 5.207066949329 | 0.099217182856 | -0.027829576569 |
| C | 5.542650556564 | 2.180680558081 | -0.546468315541 |
| H | 5.175100819600 | 2.926493666798 | -1.256711696126 |
| H | 5.318313515084 | 2.535016704273 | 0.462279820592 |
| O | 6.976678035960 | 2.044849814182 | -0.705400879263 |
| O | -6.922737400080 | -4.560382647467 | 0.386475871896 |
| C | 7.702313919158 | 3.193119241335 | -0.668323554464 |
| O | 7.206838900379 | 4.284633382672 | -0.510492400834 |
| C | -7.648255562445 | -5.708794075051 | 0.351299426421 |
| O | -7.152539304358 | -6.800642605235 | 0.196535070709 |
| C | 9.156299119204 | 2.917372703238 | -0.849722822529 |
| H | 9.434413631010 | 1.877143728187 | -0.974245656534 |
| C | 10.063989258418 | 3.897710938150 | -0.858832021889 |
| H | 9.759092933642 | 4.930725443472 | -0.731603429726 |
| H | 11.118461041917 | 3.689351718017 | -0.991937804669 |
| C | -9.102452786285 | -5.432732740462 | 0.530493639616 |
| H | -9.380783725532 | -4.392230698772 | 0.652271542257 |
| C | -10.010073860784 | -6.413125847751 | 0.540899900418 |
| H | -9.704956081922 | -7.446417154173 | 0.416427065463 |
| H | -11.064707251797 | -6.204539272025 | 0.672363905499 |

Azoalkane_TS-F400

($E_F$ = -1373.69284982 a.u.; $G_F$ = -1373.31167909 a.u.)

0 1

| | | | |
|---|---|---|---|
| N | -0.514686904353 | -1.333765340646 | 0.008877065849 |
| N | 0.568743492094 | -1.181932928391 | -0.326512199735 |
| C | -1.149379531534 | -3.258169797749 | -0.382722215859 |
| C | 1.203441086023 | 0.742464630381 | 0.065107126011 |
| C | 0.631435363925 | 1.404887628884 | -1.156703779372 |
| H | 0.975531471064 | 0.935943211866 | -2.082540776536 |
| H | -0.458551284966 | 1.340932025960 | -1.141944713463 |
| H | 0.897945076294 | 2.469776835664 | -1.185176587318 |
| C | 0.523030533182 | 1.035916244068 | 1.365047016695 |
| H | -0.538095825045 | 0.776204553908 | 1.308533566639 |
| H | 0.993442803038 | 0.484777793997 | 2.178323432403 |
| H | 0.591486338599 | 2.108377883788 | 1.589431288188 |
| C | -0.468926770220 | -3.551658360627 | -1.682631672001 |
| H | 0.592196431161 | -3.291940056864 | -1.626092846991 |
| H | -0.537370225711 | -4.624127634645 | -1.906984715809 |
| H | -0.939315548809 | -3.000546927600 | -2.495940275374 |
| C | -0.577416582740 | -3.920562252464 | 0.839125602947 |
| H | -0.843936959878 | -4.985448316156 | 0.867621460613 |
| H | 0.512570921858 | -3.856617130418 | 0.824398037953 |
| H | -0.921536568958 | -3.451587667276 | 1.764938867450 |
| C | 2.682592993113 | 0.506738190278 | 0.180030158580 |
| O | 3.169556210946 | -0.010018587948 | 1.181005279612 |
| N | 3.455542551605 | 0.906728250904 | -0.880706672217 |
| H | 3.015436388378 | 1.352903739683 | -1.668784279363 |
| C | -2.628526622802 | -3.022441957847 | -0.497700095208 |

| | | | |
|---|---|---|---|
| O | -3.115454948136 | -2.505715598642 | -1.498707491054 |
| N | -3.401512033088 | -3.422396972621 | 0.563024006361 |
| H | -2.961431321085 | -3.868538439455 | 1.351134634089 |
| C | -4.854749860000 | -3.319874158674 | 0.573292231287 |
| H | -5.144471505789 | -2.609328949642 | -0.199322962325 |
| H | -5.188069783781 | -2.935382645948 | 1.540725898242 |
| C | -5.510116750464 | -4.678260023815 | 0.315271068656 |
| H | -5.289845059715 | -5.036204407189 | -0.693153601965 |
| H | -5.162614461267 | -5.430097937992 | 1.028320465957 |
| C | 4.908780021542 | 0.804204632039 | -0.891026589247 |
| H | 5.242068061579 | 0.419812635372 | -1.858514492807 |
| H | 5.198525899426 | 0.093575609637 | -0.118492108190 |
| C | 5.564158768010 | 2.162565734184 | -0.632882210737 |
| H | 5.216622123397 | 2.914486945255 | -1.345835525911 |
| H | 5.343934188844 | 2.520399691692 | 0.375597571302 |
| O | 6.993050991768 | 1.987393250785 | -0.783927868254 |
| O | -6.939015099582 | -4.503077214244 | 0.466226526895 |
| C | 7.745813203229 | 3.114599207936 | -0.743090205166 |
| O | 7.279471477703 | 4.219587652438 | -0.587701698295 |
| C | -7.691772344403 | -5.630285400005 | 0.425523555616 |
| O | -7.225420812203 | -6.735284311881 | 0.270333499721 |
| C | 9.190128828002 | 2.798689617847 | -0.918431591357 |
| H | 9.443238030059 | 1.751706859800 | -1.038773238252 |
| C | 10.116862991976 | 3.758645009439 | -0.926999560437 |
| H | 9.832002725555 | 4.797852626003 | -0.803468627752 |
| H | 11.167709352270 | 3.529675538562 | -1.055449092055 |

| | | | |
|---|---|---|---|
| C | -9.136096705493 | -5.314363399873 | 0.600734261433 |
| H | -9.389214427345 | -4.267373807604 | 0.720899404406 |
| C | -10.062828509394 | -6.274316713610 | 0.609394943873 |
| H | -9.777959412441 | -7.313531404708 | 0.486040541703 |
| H | -11.113681550007 | -6.045338483756 | 0.737747545433 |

Azoalkane_R-F400

($E_F$ = -1373.73895359 a.u.; $G_F$ = -1373.34844668 a.u.)

0 1

| | | | |
|---|---|---|---|
| N | -0.452061154280 | -1.399774372495 | 0.198334098327 |
| N | 0.506128322907 | -1.115945351884 | -0.515915750021 |
| C | -1.014413753425 | -2.772544250791 | -0.035832564643 |
| C | 1.068477798617 | 0.256828013443 | -0.281751209562 |
| C | 0.606597465848 | 1.064775658860 | -1.504406174183 |
| H | 0.953944454597 | 0.612460382952 | -2.435939928052 |
| H | -0.484703305541 | 1.081177969048 | -1.525559109796 |
| H | 0.953506732659 | 2.099824056236 | -1.447206941094 |
| C | 0.612391026865 | 0.916843803456 | 1.025053764653 |
| H | -0.469704169204 | 1.047841821025 | 1.030049530103 |
| H | 0.892122597269 | 0.295740676071 | 1.876187907821 |
| H | 1.090892208635 | 1.894236076174 | 1.126558050091 |
| C | -0.558292549100 | -3.432581243002 | -1.342614671987 |
| H | 0.523802387642 | -3.563585633085 | -1.347576681545 |
| H | -1.036795813681 | -4.409972412596 | -1.444118355592 |
| H | -0.837995729464 | -2.811489506922 | -2.193766202890 |
| C | -0.552580134370 | -3.580482016582 | 1.186846785408 |

| | | | |
|---|---|---|---|
| H | -0.899501372248 | -4.615526393247 | 1.129650506698 |
| H | 0.538719837888 | -3.596899339026 | 1.208032671441 |
| H | -0.899948592479 | -3.128150034643 | 2.118364046065 |
| C | 2.601134683698 | 0.032348535066 | -0.174360152216 |
| O | 3.036029761790 | -0.812151984837 | 0.592523722019 |
| N | 3.402205218728 | 0.857654895550 | -0.910079272738 |
| H | 2.973661654868 | 1.463391214185 | -1.591306494839 |
| C | -2.547065868692 | -2.548052024876 | -0.143274307853 |
| O | -2.981928229643 | -1.703563475769 | -0.910189931743 |
| N | -3.348167937790 | -3.373331914976 | 0.592440820332 |
| H | -2.919653209827 | -3.979057946450 | 1.273695032997 |
| C | -4.804989875901 | -3.294282881657 | 0.588156858999 |
| H | -5.090238327079 | -2.547019281737 | -0.150716422031 |
| H | -5.169877641197 | -2.963300032502 | 1.565446945660 |
| C | -5.426022337754 | -4.649953794846 | 0.254813810780 |
| H | -5.197232000754 | -4.952589056592 | -0.769775307706 |
| H | -5.066672929876 | -5.432839840024 | 0.928099111778 |
| C | 4.859028236684 | 0.778612497209 | -0.905852077451 |
| H | 5.223879024274 | 0.447692815530 | -1.883177219384 |
| H | 5.144308295279 | 0.031303106053 | -0.167036544241 |
| C | 5.480071014425 | 2.134261990229 | -0.572445340452 |
| H | 5.120655225300 | 2.917199808651 | -1.245637468161 |
| H | 5.251364048955 | 2.436813116109 | 0.452186677534 |
| O | 6.911329761243 | 1.994009519683 | -0.729992334019 |
| O | -6.857293469951 | -4.509675827858 | 0.412221120630 |
| C | 7.642923828386 | 3.133279573469 | -0.645223567634 |

| | | | |
|---|---|---|---|
| O | 7.155593889718 | 4.221617851915 | -0.444727690801 |
| C | -7.588879839094 | -5.648966556851 | 0.327650688826 |
| O | -7.101531784942 | -6.737351354507 | 0.127467623326 |
| C | 9.091978191843 | 2.850856389574 | -0.835738150744 |
| H | 9.363461384318 | 1.814181538707 | -0.997662730272 |
| C | 10.001236637376 | 3.827038197904 | -0.809123997889 |
| H | 9.698212253212 | 4.855335481629 | -0.644671007463 |
| H | 11.055569468890 | 3.622197822452 | -0.949014770132 |
| C | -9.037954037049 | -5.366498019585 | 0.517942400232 |
| H | -9.309453747309 | -4.329782522076 | 0.679593521612 |
| C | -9.947210262489 | -6.342688510415 | 0.491449001122 |
| H | -9.644168262068 | -7.371026735544 | 0.327269381029 |
| H | -11.001558674415 | -6.137815128756 | 0.631169694134 |

Azoalkane_TS-F200

($E_F$ = -1373.59029850 a.u.; $G_F$ = -1373.21011810 a.u.)

0 1

| | | | |
|---|---|---|---|
| N | -0.465757714848 | -1.418346922873 | 0.081632772147 |
| N | 0.519611916702 | -1.097283825223 | -0.399518991241 |
| C | -0.959693480941 | -3.360082103662 | -0.426376000508 |
| C | 1.013686924899 | 0.844341933087 | 0.108697617582 |
| C | 0.413914540839 | 1.512209856398 | -1.097724384260 |
| H | 0.717687063166 | 1.037837042746 | -2.034991468860 |
| H | -0.675910957379 | 1.466864330201 | -1.051208291432 |
| H | 0.703476242834 | 2.569937201004 | -1.138732782465 |
| C | 0.310668328142 | 1.054967376176 | 1.409745462172 |

| | | | |
|---|---|---|---|
| H | -0.721887447880 | 0.698269214689 | 1.347342123788 |
| H | 0.828814682805 | 0.541396947980 | 2.217953676163 |
| H | 0.277751023571 | 2.125754328186 | 1.647978725340 |
| C | -0.256560778253 | -3.570877012686 | -1.727330739748 |
| H | 0.775943185955 | -3.214026552279 | -1.664923211711 |
| H | -0.223481128760 | -4.641709196780 | -1.965343496502 |
| H | -0.774721641035 | -3.057550008791 | -2.535684418730 |
| C | -0.359943048574 | -4.027675142630 | 0.780211023040 |
| H | -0.649508941272 | -5.085390726133 | 0.821447809424 |
| H | 0.729882249078 | -3.982325501866 | 0.733717392041 |
| H | -0.663738837077 | -3.553102761131 | 1.717369830892 |
| C | 2.496102456075 | 0.660860510482 | 0.208015845707 |
| O | 3.040810930649 | 0.362555832767 | 1.268917721491 |
| N | 3.216289283185 | 0.823781171146 | -0.944207062778 |
| H | 2.747700487346 | 1.100591951081 | -1.790314705214 |
| C | -2.442124789793 | -3.176810596242 | -0.525821867718 |
| O | -2.986868545194 | -2.879191260363 | -1.586912931499 |
| N | -3.162313770552 | -3.339161228977 | 0.626484215480 |
| H | -2.693703696490 | -3.615333964942 | 1.472802782446 |
| C | -4.611605377124 | -3.216316047819 | 0.655467411188 |
| H | -4.911559328291 | -2.484567782221 | -0.094437232806 |
| H | -4.919937986351 | -2.853961423164 | 1.638231790190 |
| C | -5.285059149952 | -4.556214680105 | 0.365865035641 |
| H | -5.049642807264 | -4.898484913937 | -0.644142822619 |
| H | -4.966851632699 | -5.324303027000 | 1.074950152539 |
| C | 4.665569255113 | 0.700824336739 | -0.973303434767 |

| | | | |
|---|---|---|---|
| H | 4.973833644756 | 0.338711748514 | -1.956184000780 |
| H | 4.965484202115 | -0.031160852551 | -0.223611385805 |
| C | 5.339140544440 | 2.040595082327 | -0.683367295408 |
| H | 5.020948314871 | 2.808907236235 | -1.392222384170 |
| H | 5.103810287161 | 2.382611767526 | 0.326751042562 |
| O | 6.764083565820 | 1.832561895412 | -0.806560194319 |
| O | -6.710024598574 | -4.348251281872 | 0.488919875223 |
| C | 7.541236077142 | 2.937315889600 | -0.720054334037 |
| O | 7.100252861646 | 4.050250077632 | -0.547251649959 |
| C | -7.487102191267 | -5.453063045513 | 0.402532854563 |
| O | -7.046038331841 | -6.565991254340 | 0.229934178143 |
| C | 8.977643591410 | 2.585444213778 | -0.871871726470 |
| H | 9.207403476280 | 1.535595302426 | -1.011293042370 |
| C | 9.923250227683 | 3.523771652217 | -0.837999417521 |
| H | 9.656709700810 | 4.565443955176 | -0.696032633936 |
| H | 10.972047732246 | 3.276696421918 | -0.948925179469 |
| C | -8.923542286600 | -5.101260422536 | 0.554197759612 |
| H | -9.153375284700 | -4.051410170259 | 0.693448006451 |
| C | -9.869090402653 | -6.039646689682 | 0.520399100272 |
| H | -9.602477165565 | -7.081318303380 | 0.378604435909 |
| H | -10.917910560414 | -5.792621404457 | 0.631215811999 |

Azoalkane_R-F200

($E_F$ = -1373.63654442 a.u.; $G_F$ = -1373.24837690 a.u.)

0 1

| | | | |
|---|---|---|---|
| N | -0.385004335740 | -1.447790552801 | 0.254694103017 |

| | | | |
|---|---|---|---|
| N | 0.439041417672 | -1.067908648554 | -0.572342521607 |
| C | -0.923154246488 | -2.824185478250 | 0.007233246295 |
| C | 0.977193812711 | 0.308485322755 | -0.324880749019 |
| C | 0.467274450885 | 1.130676991091 | -1.517645928075 |
| H | 0.781212587039 | 0.692322495893 | -2.467466933501 |
| H | -0.624291509096 | 1.139907831441 | -1.500417449461 |
| H | 0.810276234891 | 2.166880827165 | -1.458112152426 |
| C | 0.550155466126 | 0.936401261874 | 1.005996782364 |
| H | -0.534850057405 | 1.036073691354 | 1.049395972926 |
| H | 0.876010548776 | 0.314241848113 | 1.838760815498 |
| H | 1.002615738788 | 1.926727759139 | 1.103230637108 |
| C | -0.496122270785 | -3.452098358703 | -1.323648079519 |
| H | 0.588882873818 | -3.551770651965 | -1.367053133749 |
| H | -0.948582588600 | -4.442424778151 | -1.420882187327 |
| H | -0.821981640257 | -2.829936741735 | -2.156408878367 |
| C | -0.413224227905 | -3.646377263425 | 1.199994119483 |
| H | -0.756217278665 | -4.682583942815 | 1.140458150690 |
| H | 0.678341634058 | -3.655599356069 | 1.182761851335 |
| H | -0.727163155115 | -3.208028427215 | 2.149817751409 |
| C | 2.513971246065 | 0.094763905987 | -0.270022364684 |
| O | 2.985617383191 | -0.698804916978 | 0.529374913381 |
| N | 3.276351918425 | 0.850124630675 | -1.108602766738 |
| H | 2.819961782181 | 1.453803106613 | -1.772570318839 |
| C | -2.459932239121 | -2.610470487110 | -0.047615243058 |
| O | -2.931585609288 | -1.816887213632 | -0.846992907837 |
| N | -3.222305342834 | -3.365857409732 | 0.790948337335 |

| | | | |
|---|---|---|---|
| H | -2.765908790521 | -3.969542367218 | 1.454904948663 |
| C | -4.675802782978 | -3.287290246010 | 0.835885116792 |
| H | -4.980293360802 | -2.426799461122 | 0.242046325057 |
| H | -5.005561183705 | -3.130013831426 | 1.866334575001 |
| C | -5.314294374090 | -4.561768234383 | 0.290749659164 |
| H | -5.102280555556 | -4.688804968120 | -0.773569618829 |
| H | -4.955488662849 | -5.448896546607 | 0.818804491158 |
| C | 4.729849474385 | 0.771547705065 | -1.153531328852 |
| H | 5.059611995167 | 0.614262787784 | -2.183979731991 |
| H | 5.034333036016 | -0.088942372294 | -0.559684866889 |
| C | 5.368344615688 | 2.046026774438 | -0.608400004993 |
| H | 5.009547778042 | 2.933154690418 | -1.136464752896 |
| H | 5.156323359117 | 2.173072634601 | 0.455918482814 |
| O | 6.792707344580 | 1.913374454106 | -0.808727304356 |
| O | -6.738655684999 | -4.429127471879 | 0.491089221859 |
| C | 7.541893404382 | 3.013551207693 | -0.559192316840 |
| O | 7.077026944652 | 4.063151029217 | -0.178751762255 |
| C | -7.487839501589 | -5.529300315641 | 0.241536124689 |
| O | -7.022972563222 | -6.578886503765 | -0.138932142447 |
| C | 8.979000106196 | 2.742064869752 | -0.824192368971 |
| H | 9.230638173824 | 1.739276198416 | -1.148885862992 |
| C | 9.899303863469 | 3.693426908339 | -0.670263762949 |
| H | 9.611638584016 | 4.686863444996 | -0.343813662722 |
| H | 10.948196307362 | 3.504481498680 | -0.863687080314 |
| C | -8.924944305817 | -5.257828968319 | 0.506555372708 |
| H | -9.176582393153 | -4.255053221529 | 0.831279486631 |

| | | | |
|---|---|---|---|
| C | -9.845246401391 | -6.209188766893 | 0.352607222525 |
| H | -9.557581147424 | -7.202612044277 | 0.026125959658 |
| H | -10.894137410803 | -6.020254536325 | 0.546044710608 |
